# Application of Microbiome Feedback Theory to Animals: Can Parasites Drive Coexistence in Ungulate Communities?

**DOI:** 10.1093/icb/icaf087

**Published:** 2025-06-10

**Authors:** James D Bever, James S Adelman, Maarten B Eppinga, Elizabeth A Archie, Vanessa O Ezenwa

**Affiliations:** Department of Ecology and Evolutionary Biology, and Kansas Biological Survey, University of Kansas, Lawrence, KS, 66045, USA; Department of Biological Sciences, University of Memphis, Memphis, TN, 38152, USA; Department of Geography, University of Zurich, Zurich, Switzerland; Department of Biological Sciences, University of Notre Dame, South Bend, IN 46556, USA; Department of Ecology and Evolutionary Biology, Yale University, New Haven, CT 06511, USA

## Abstract

Parasites can have large impacts on host populations, but the extent to which parasite dynamics impact or respond to multi-species community structure remains uncertain. Empirical and theoretical studies within the host–microbiome feedback framework (often called plant–soil feedback) has provided strong evidence of the importance of soil pathogens to plant community structure and function. We adapt this framework to herd animals by extending the mathematics of host–microbiome feedback theory to accommodate increased likelihood of exposure to microbiomes from conspecific hosts rather than heterospecific hosts. We then integrate this framework with a model of interguild frequency dependence. Coupling this model with empirical observations, we estimate the host-specific fitness of gastro-intestinal nematodes living on ungulate species of Western USA. We find evidence that host-specific differences in nematode fitness could generate negative feedback on host fitness and contribute to coexistence of ungulates. Moreover, we find that this is more likely to be the case for pairs of ungulate species with high habitat overlap. If nematodes can indeed drive such negative feedbacks, then negative impacts of nematodes on their ungulate hosts should decline, i.e., be diluted, with increasing host diversity. While more work is necessary to confirm the underlying assumptions driving these conclusions, our work highlights the possibility that parasites play under appreciated roles in structuring animal communities.

## Introduction

Disease-causing organisms (henceforth “parasites”) can have large impacts on host populations, a reality being increasingly appreciated in studies of both animals and plants. While most work has focused on interactions of individual host and parasite species in isolation, these interactions are occurring in community settings and have potential to impact and respond to host community structure (Johnson et al. 2015). However, the extent to which host–parasite dynamics impact or respond to multi-species community structure remains uncertain, as illustrated by the controversy over diversity-disease relationships in ecological communities (Rohr et al. 2020). This controversy in part reflects a reliance of the discipline on a modeling framework that emphasizes the dynamics of diseased individuals in populations, often called the SIR framework (Anderson and May 1979). While this framework has proven value in describing the conditions for disease spread within individual populations, tracking the dynamics of multiple host and parasite species can be challenging (Buhnerkempe et al. 2015; Jiang et al. 2020), which limits inference on interconnections of disease incidence and host community structure. An alternative approach, the microbiome feedback framework, has proven useful in demonstrating the influence of parasites on coexistence of plant species (Bever et al. 2015). However, this approach has not yet been applied to animal hosts.

**Fig. 1:**
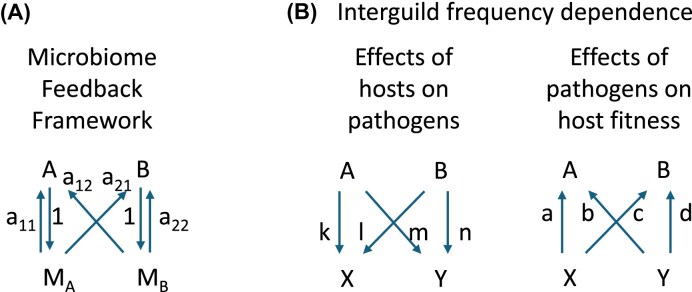
Panel A presents the fitness relations between two host species (A and B) and the microbiomes influenced by these two species (M_A_ and M_B_, respectively) within the microbiome feedback model [modified from (Bever et al. 1997)]. The arrows represent the direction of fitness impacts with a_11_ and a_21_ representing the fitness effects on plant A and B due to changes in microbiome by plant A, and a_12_ and a_22_ representing the fitness effects on plant and B due to changes in microbiome by plant B. Microbiome mediated coexistence is possible when I_s_ = a_11_–a_12_–a_21_ + a_22_ < 0. Panel B presents the fitness relations within the interguild frequency dependence model [modified from (Bever 1999)] in which arrows represent direction of the fitness effects between host A and B and parasites X and Y. Parameters k, l, m and n represent the magnitude of fitness effects of hosts on parasites and a, b, c and d represent the fitness effects of parasites on hosts. Coexistence is possible when *I*_H_ * *I_M_* < 0, where I_H_ = a—b—c + d and I_M_ = k—l—m + n.

The microbiome feedback framework explicitly considers the potential interdependence of host community structure with the net impacts of the collection of microbes, symbionts, and parasites interacting with the hosts (i.e., the host microbiome *sensu lato*). It was developed to test the importance of the soil microbiome on plant–plant interactions (Bever 1994; Bever et al. 1997). Microbiome feedback theory identifies that microbiome changes can drive coexistence of competing host species when host-specific changes in microbiome composition impedes the focal host species more than competitor host species (Fig. 1A). This stabilizing negative feedback would be expected if parasite specialization results in higher virulence on focal versus non-focal hosts (Bever 2003). The microbiome feedback framework can be applied to the entirety or any subset of organisms interacting with a host. Studies using this framework have provided evidence that soil parasites can have important impacts on plant community structure, including facilitating coexistence among plant species (Bever et al. 2015; Crawford et al. 2019), generating patterns of native plant diversity (Eppinga et al. 2018), driving relative abundance of plant species (Mangan et al. 2010), contributing to plant species invasions (Eppinga et al. 2006; Crawford et al. 2019), and enabling species turnover during succession (Van der Putten et al. 1993; Bauer et al. 2015). Recently, the framework has been used to show the generality of parasite dilution with plant diversity (Collins et al. 2020; Wang et al. 2023). That is, parasite impacts on plant hosts will generally decline with host diversity when parasites drive negative feedback on host fitness.

Microbiome feedback has been commonly measured using a straightforward experimental assay, where a common microbiome (e.g., a mixture of parasites) is exposed to, and trained on, individual host species, thereby allowing host-specific differentiation of the microbiome, and then the consequences of host-specific differentiation is tested in a separate full factorial assay of host fitness [(Bever 1994), Fig. 2]. This experimental approach integrates the differential growth rates of parasites with the differential impacts of those parasites on hosts into a single condition for the stabilizing impact of parasites on host coexistence, as represented by *I_S_* (Bever et al. 1997).

**Fig. 2:**
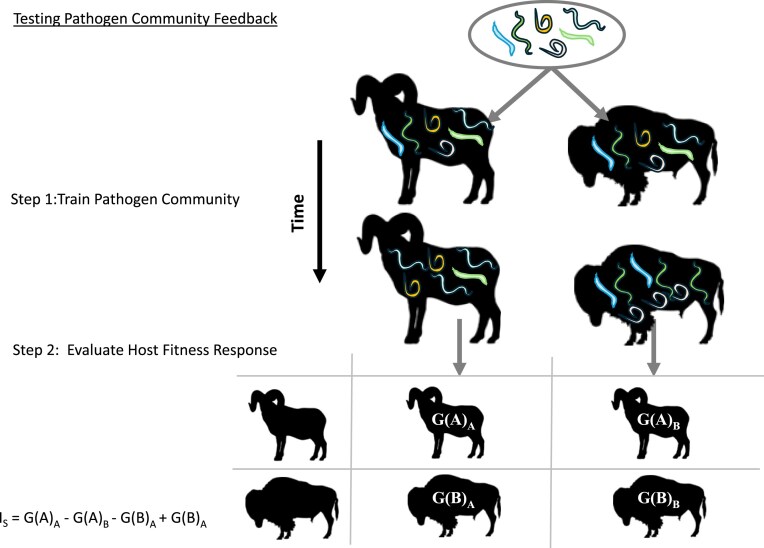
The stability condition for host–microbiome feedback could be tested by exposing uninfected hosts (e.g., sheep and bison as illustrated here) to a common microbiome, allowing the microbiome to differentiate on the host, and then testing the fitness consequences of the differentiation in a separate assay in which all hosts were grown with exposure to the two trained microbiomes. The differential effects on fitness proxies (labelled G(i)_j_) to represent growth response of host i to microbiome trained on host j) will determine the interaction coefficient (I**_s_**) as described in (Bever et al. 1997). Sheep and bison silhouettes are credited to Gabriela Palomo-Munoz and T. Michael Keesey, respectively.

**Fig. 3:**
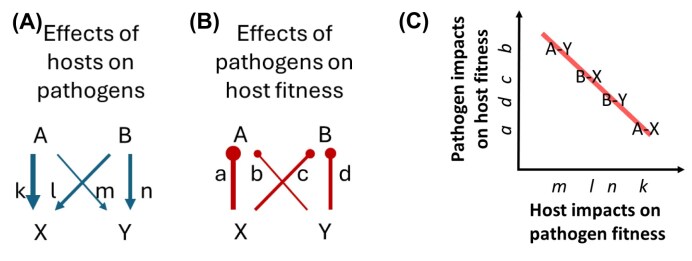
Host pathogen fitness effects are likely to be negatively correlated. (A) Positive effects of hosts on parasites (arrows) are assumed to be proportional to (B) negative effects of parasites on hosts (clubs). When this is true, (C) the relationship between host and parasite should be negative across multiple host–parasite pairings. This relationship is consistent with parasite-driven negative feedback, as I_H_ * I_M_ < 0, where I_H_ = a—b—c + d and I_M_ = k—l—m + n (Bever 1999).

To our knowledge, the microbiome feedback theoretical or experimental approach has not been previously applied to interactions between animal hosts and their parasites. However, there is no fundamental reason preventing such application (Abbott et al. 2021). The host specificity of parasites provides evidence that parasite fitness depends, and hence parasite community composition changes, with animal host identity. We note that host differences in parasite fitness starts with exposure and integrates through parasite infectivity (), and as such includes host specialists (i.e., narrow host range) as well as fitness differences in host generalists. However, the consequences of these changes on host fitness are difficult to generalize. In the absence of this experimental approach to assessment of net microbiome feedback (Fig. 2), one could infer feedback dynamics from the product of differential fitness effects of hosts and parasites (Fig. 1B), as represented within a model of interguild frequency dependence (Bever 1999). Negative microbiome feedback occurs when host effects on microbes are negatively correlated with microbe effects on hosts (Bever 1999). Such a negative correlation is expected between hosts and most parasites because parasite fitness generally increases as detrimental effects on hosts increase (Fig. 3).

Here, we adapt the feedback framework to evaluate the potential for parasitic nematodes to contribute to the coexistence of sympatric ungulate species in western North America. We focused on gastrointestinal (GI) nematodes which are common parasites of wild and domestic ungulates. These parasites are transmitted via a fecal–oral route, where infected hosts shed parasite propagules into the environment that develop into infective stages and are ingested by susceptible hosts during grazing (Bowman 2021). We used measures of nematode egg abundance in host feces to estimate host-specific fitness of nematodes.

We use a general model of interdependence of host and parasite fitness (Bever 1999) to project feedback and coexistence. A core assumption of this model is that all hosts and parasites associate at random, as expected under density-dependent mass action (Bever 1999). However, all of our focal ungulate hosts are social species that live in groups, with quantifiably different habitat niches (McCullough 1980); consequently, we expect higher rates of exposure to microbiomes from conspecific hosts than would be expected by chance, and that the enhanced exposure to conspecific parasite communities will depend upon the degree of habitat overlap between two species. Application of the feedback model to this system requires an modification of the theory to accommodate an increased likelihood of within-species exposure. Therefore, we (1) extend host microbiome feedback theory (Fig. 1A) to accommodate increased within species exposure, (2) translate new results from microbiome feedback theory to parameterization of the interguild frequency dependence (Fig. 1B), (3) use these theoretical results and the assumption of negative correlation of host and pathogen fitness effects (Fig. 3) to infer the potential importance of nematode dynamics on ungulate coexistence from observations of nematode abundance in ungulate hosts.

Our theory development is limited to interactions of two hosts and two parasites (See theory development below). For each pair of hosts and pair of parasites, this exercise yields two metrics of the potential for nematode-mediated feedback to drive ungulate coexistence: (1) a feasible equilibrium identifies that coexistence is possible and (2) when it is possible, the strength of the stabilization force identifies stability of coexistence. We extract these metrics for many host-pair/parasite-pair combinations and then test for patterns in these measures across ecological and evolutionary dimensions of hosts and parasites. Specifically, we test whether parasite-mediated coexistence is more likely for host pairs with a high level of resource competition (habitat overlap), as habitat overlap should increase the likelihood of exposure to microbiomes from heterospecific hosts. We also test whether host phylogenetic relationships structure parasite feedbacks, as such feedbacks have been found to be more negative between phylogenetically disparate plant hosts (Crawford et al. 2019). Finally, we test whether variation in nematode host ranges influence the likelihood of mediation of host coexistence, as parasites with narrow host ranges may be more likely to generate negative feedbacks due to their specialization. Together these analyses provide a first look at patterns and potential consequences of parasite-mediated negative feedback in animal hosts.

## Theory development

### Theory overview

Microbiome feedback models (Fig. 1A), i.e., Bever et al. (1997) and models that build on this framework [e.g., (Bever 2003; Revilla et al. 2013; Eppinga et al. 2018; Kandlikar et al. 2019; Mack et al. 2019; Ke and Wan 2020; Abbott et al. 2021; Miller et al. 2022)], as well as the host–microbe interguild frequency dependence model (Fig. 1B) of Bever (1999) share an assumption of random association of hosts and microbiome components. That is, these mean field models assume wide dispersal and density-dependent association of host and microbes. Local dispersal of “microbes” would generate greater probability of conspecific spread as is represented in spatially explicit simulations of plant–soil feedback dynamics (Mangan et al. 2010; Mack and Bever 2014). Compared to plants, the high mobility of animals will increase interspecific transmission. However, conspecific herding, as supported the aggregation behavior of many ungulate species, suggests that conspecific exposure will be greater than expected by chance. Note that this perspective is coming from that of the host, as pathogen movement is dependent on host behavior. This host-centric perspective is consistent with the microbiome feedback model (Fig 1A), not with model of interguild frequency dependence. Therefore consideration of greater transmission between conspecifics needs to be made explicit by modifying exposure assumptions in the microbiome feedback model. We will then need to convert between the parameters of the microbiome feedback model and the host–microbe interguild frequency dependence in order to interpret data on nematode abundance. We do that in the steps below.

### Accommodation of greater probability of exposure to microbiomes of conspecific hosts

In the microbiome feedback model differential fitness effects of hosts A and B on microbes is implicitly represented with changes in respective microbiomes (*M_A_* and *M_B_*, in Fig. 1A). We can accommodate increased exposure to microbiomes of conspecific hosts, whether resulting from herding behavior of animals or limited dispersal of sessile organisms such as plants, by allowing the parameter φ to modulate the effects of spatial proximity (Bever and Simms 2000). We assume that φ proportion of the time a host would be infected with its own microbiome, while (1−φ) of the time hosts are exposed to microbiomes at random (i.e., in proportion to host density). With this we can modify the representation for average fitness of plant A and B, *W_A_* and *W_B_*, respectively, from the general modeling feedback framework (Fig. 1A) (Bever et al. 1997), as follows:


\begin{eqnarray*}
{{W}_A} = \varphi {{a}_{11}} + \left( {1 - \varphi } \right)\left( {{{a}_{11}}{{p}_{{{M}_A}}} + \ {{a}_{12}}{{p}_{{{M}_B}}}} \right),
\end{eqnarray*}

\begin{eqnarray*}
{{W}_B} = \varphi {{a}_{22}} + \left( {1 - \varphi } \right)\left( {{{a}_{21}}{{p}_{{{M}_A}}} + \ {{a}_{22}}{{p}_{{{M}_B}}}} \right),
\end{eqnarray*}

where *a_ij_* is the impact of the change in microbiome due to host j on host i, and ${{p}_{{{M}_A}}}$ and ${{p}_{{{M}_B}}}$ represent the proportion of the microbiome influenced by host A and B, respectively. That is, φ proportion of the time a host’s fitness is impacted by its own microbiome, while (1−φ) of the time host fitness is impacted by microbiomes according to the average microbiome composition at that moment, as assumed in the standard feedback model. With these fitness we can describe the exponential growth of host A and B as $\frac{{dA}}{{dt}} = \ {{W}_A}A$ and $\frac{{dB}}{{dt}} = \ {{W}_B}B$. Application of the chain rule as in (Bever et al. 1997) allows reduction of the dimensionality of these equations into a single equation for the rate of change in proportion of host A among all hosts as


\begin{eqnarray*}
\frac{{d{{p}_A}}}{{dt}} = \ {{p}_A}{{p}_B}\left( {{{W}_A} - {{W}_B}} \right)
\end{eqnarray*}

where *p_A_* and *p_B_* represent the proportion of hosts A and B respectively in the host community. As proportions, there is an implicit assumption of competition (i.e., *p_B_ =* 1- *p_A_*). As proportion Further analysis requires assuming that microbiome dynamics are fast relative to the dynamics of the host [as outlined in (Eppinga et al. 2018)], an assumption that will be true for most host–microbe interactions, including nematodes and ungulates. We can then write the equation for equilibrium of the proportion of host A as


(1) \begin{eqnarray*}
\widehat {{{p}_A}} &=& \ \frac{{\varphi \left( {{{a}_{22}} - {{a}_{11}}} \right) + \left( {1 - \varphi } \right)\left( {{{a}_{22}} - {{a}_{12}}} \right)}}{{\left( {1 - \varphi } \right){{I}_S}}} \\
&=& \ \frac{{{{a}_{22}} - {{a}_{12}}\ + \ \varphi \left( {{{a}_{12}} - {{a}_{11}}} \right)}}{{\left( {1 - \varphi } \right){{I}_S}}}
\end{eqnarray*}

The equilibrium is feasible if


(2) \begin{eqnarray*}
0{\mathrm{\ }} < {\mathrm{\ }}{{\hat{p}}_A} < {\mathrm{\ }}1),
\end{eqnarray*}

and it is stable when:


(3) \begin{eqnarray*}
\left( {{\mathrm{1 - \varphi }}} \right){{{\mathrm{I}}}_{\mathrm{s}}}{\mathrm{ < 0}}
\end{eqnarray*}

(see derivation in Appendix 1), where


(4) \begin{eqnarray*}
{{{\mathrm{I}}}_{\mathrm{s}}}{\mathrm{ = }}{{{\mathrm{a}}}_{{\mathrm{11}}}}{\mathrm{ - }}{{{\mathrm{a}}}_{{\mathrm{12}}}}{\mathrm{ - }}{{{\mathrm{a}}}_{{\mathrm{21}}}}{\mathrm{ + }}{{{\mathrm{a}}}_{{\mathrm{22}}}}
\end{eqnarray*}

and describes the stabilization force for the general feedback model (Bever et al. 1997). Biologically, this condition means that coexistence is possible when the direct effects of microbiome change on fitness of conspecifics (a_11_ and a_22_) are more negative or less positive than the effects of microbiome change on heterospecifics (a_12_ and a_21_), a condition that may be named negative microbiome feedback.

Interestingly, the stabilization force of microbiome feedback decreases with φ, indicating that elevated exposure to conspecific parasites reduces the likelihood of coexistence via parasite mediated negative feedbacks. This is not a result that would have been obvious in the absence of modeling. For example, the result is surprising from the perspective that greater conspecific transmission would hinder conspecifics, hence enhancing fitness differences with heterospecifics. However, it highlights that stabilization from microbiome feedback always stems from the differential microbial responses to, and impacts on, hosts, and these differential impacts are only realized when microbiomes are shared between host species.

### Translating parameters between feedback theory and model of interguild frequency dependence

The modeling of interguild frequency dependence [(Bever 1999), Fig. 1B] shares basic assumptions with microbiome feedback theory (Fig. 1A) in that it considers exponentially growing populations of hosts (here identified as host A and B) and microbes (e.g., symbionts or parasites) in which relative fitnesses are dependent on the host or microbe with which they are associated. In the model of interguild frequency dependence, the change in abundance of individual microbes (X and Y) are explicitly monitored as state variables (Fig. 1B). The model of interguild frequency dependence (Fig. 1B) shares with the basic feedback model (1) the core biological assumption of exponential growth of hosts and parasites that are dependent on each other, and (2) the implicit assumption of saturating dynamics embedded in the modeling of proportions (i.e., proportions are bound between 0 and 1; hence p_B_ = 1–p_A_). Within this framework, Bever (1999) identified that coexistence is possible when:


(5) \begin{eqnarray*}
{{{\mathrm{I}}}_{\mathrm{H}}}{\mathrm{* }}{{{\mathrm{I}}}_{\mathrm{M}}}{\mathrm{ < 0}},
\end{eqnarray*}

where I_H_ is the interaction coefficient of host responses to microbes (I_H_ = a—b—c + d) and I_M_ is the interaction coefficient of microbe responses to hosts (I_M_ = k—l—m + n). As I_H_ and I_M_ are measures of specificity in fitness effects of microbes on hosts and hosts on microbes, respectively, this stability condition identifies that host–microbe dynamics can contribute to coexistence when there is a negative correlation between the differential fitness effects and responses of microbes and hosts. This stability condition shares similarities to the stability condition for microbiome feedback (Eq. 3, when φ = 0), perhaps not surprising given the common assumptions underlying the models. However, to date, these two modeling approaches have not been formally equated. We do so here.

As the feedback parameters *a_ij_* represent net results of differential microbial responses and impacts on hosts, they must be composed of the individual pairwise fitness (*a.d, k.n*). We construct these relationships by following paths from host abundance to changed microbial fitness back to host fitness. The parameter *a_11_* will then be composed of all paths that begin and end with host A. This includes two direct paths to and from individual microbes, represented by k*a through microbe X and m*b through microbe Y. There are also two indirect paths looping back to host A. These connect through the implicit competitive effect between the microbes, as an increased fitness of X leads to a decreased proportion of Y. These indirect paths are from host A to microbe X to microbe Y back to A as represented by k*(−1)*b = −kb, and from host A to microbe Y to microbe X back to host A as represented by m*(−1)*a. This corresponds to the product of differences in host and microbe fitnesses associated with host A and the two microbes as follows:


(6) \begin{eqnarray*}
{{{\mathrm{a}}}_{{\mathrm{11}}}}{\mathrm{ = ak - am - bk + bm = }}\left( {{\mathrm{a - b}}} \right)\left( {{\mathrm{k - m}}} \right),
\end{eqnarray*}

Using the same logic, we identify that


(7) \begin{eqnarray*}
{{{\mathrm{a}}}_{{\mathrm{12}}}}{\mathrm{ = }}\left( {{\mathrm{d - c}}} \right)\left( {{\mathrm{m - k}}} \right){\mathrm{ = dm - kd - cm + ck}},
\end{eqnarray*}

(8) \begin{eqnarray*}
{{{\mathrm{a}}}_{{\mathrm{21}}}}{\mathrm{ = }}\left( {{\mathrm{b - a}}} \right)\left( {{\mathrm{n - l}}} \right){\mathrm{ = bn - an - bl + al}},
\end{eqnarray*}

and


(9) \begin{eqnarray*}
{{{\mathrm{a}}}_{{\mathrm{22}}}}{\mathrm{ = }}\left( {{\mathrm{d - c}}} \right)\left( {{\mathrm{n - l}}} \right){\mathrm{ = dn - cn - dl + cl}}.
\end{eqnarray*}

Using these parameter conversions, we show that


(10) \begin{eqnarray*}
{{{\mathrm{I}}}_{\mathrm{s}}}&=&{{{\mathrm{I}}}_{\mathrm{H}}}{\mathrm{* }}{{{\mathrm{I}}}_{\mathrm{M}}}{\mathrm{ = }}\left( {{\mathrm{a -- b -- c + d}}} \right)\left( {{\mathrm{k -- l -- m + n}}} \right)\\
&=&{\mathrm{ \ ak --al - am + an -- bk + bl}}\\
&&{\mathrm{ + bm -- bn -- ck + cl}}\\
&&+{\mathrm{ cm -- cn + dk -- dl -- dm + dn}}.
\end{eqnarray*}

That is, in the case of two microbes and two hosts, the stability condition for microbiome feedback equals the stability condition for interguild frequency dependence. This provides a formal demonstration that stabilizing potential of microbiome feedback emerges from a negative correlation of differential fitness impacts of hosts on microbes and of microbes on hosts, as illustrated in Fig. 3.

### Derivation of expected values of parasite abundance in the field

Aggregation of ungulate species will likely increase the rate of exposure of nematodes to conspecific hosts relative to stochastic expectations. This greater conspecific exposure will influence the realized abundance of nematodes in the field and this effect will need to be accounted for in use of field data for estimation of nematode fitness responses to individual hosts. We use the results of the previous two sections to estimate the expected abundance as a function of φ and fitness parameters (*a.d* and *k.n*). This was accomplished through a recursive consideration of individual transmission events as described in 2 This exercise generated four equations representing the expected abundance of parasites X and Y in host A and B as represented by *PX_A_, PY_A_, PX_B_*, and *PY_B_* in equations 11–14:


(11) \begin{eqnarray*}
P{{X}_A} = \frac{{k\left( {{{{\hat{P}}}_A} - 1} \right)\left( {\varphi - 1} \right)}}{{1 - k\left( {\varphi + \left( {1 - \varphi } \right){{{\hat{P}}}_A}} \right)}},
\end{eqnarray*}

(12) \begin{eqnarray*}
P{{Y}_A} = \frac{{m\left( {{{{\hat{P}}}_A} - 1} \right)\left( {\varphi - 1} \right)}}{{1 - m\left( {\varphi + \left( {1 - \varphi } \right){{{\hat{P}}}_A}} \right)}},
\end{eqnarray*}

(13) \begin{eqnarray*}
P{{X}_B} = \frac{{l{{{\hat{P}}}_A}\left( {\varphi - 1} \right)}}{{1 - l\left( {\varphi + \left( {1 - \varphi } \right)\left( {1 - {{{\hat{P}}}_A}} \right)} \right)}},
\end{eqnarray*}

(14) \begin{eqnarray*}
P{{Y}_B} = \frac{{n{{{\hat{P}}}_A}\left( {\varphi - 1} \right)}}{{1 - n\left( {\varphi + \left( {1 - \varphi } \right)\left( {1 - {{{\hat{P}}}_A}} \right)} \right)}},
\end{eqnarray*}

where ${{\hat{p}}_A}$ is the equilibrium proportion of host A (Eq. 1). Substituting the equations for the feedback parameters (Eq. 6–9) gives four equations with 8 unknowns (Eq. 4a–d in Appendix 3).

### Derivation of parasite fitness values

To solve equations 11–14 for parasite fitness estimates (*k, l, m, n*), we need to assume a relationship between host and parasite fitness effects. We assumed that parasite fitness impacts on hosts are negatively related to parasite fitness gains on that host (e.g., *a* = −*k, b = −m*, etc.), as depicted in Fig. 3. This inverse relationship between host and parasite fitness is supported by experimental studies showing that host fitness tends to decline with increasing parasite replication [e.g., (Råberg et al. 2007; de Roode et al. 2008)]. Substituting these relationships and rearranging these equations by solving for the fitness responses of nematodes yielded four equations with four unknowns (*k, l, m*, and *n*) as functions of the expected abundance of nematodes and φ (Appendix 3). These equations were solved with the aid of Mathematica yielding solutions that required more than 10,000 lines to write (Electronic supplementary material). We tried non-linear relationships between parasite impacts and parasite responses to that host (e.g., *a* = 1/*k, etc*.), but the equations could not be solved. However, we note that variation in the slope of the linear relationship between host and microbial fitness (e.g., *a* = *−μ k*, where *μ* is the slope) proportionally alters the stabilizing force [= *μ* (1*−*φ) *I_s_*^]^, but does not change the equilibrium proportion of the hosts (i.e., ${{\hat{P}}_A}$).

## Estimating nematode fitness across ungulate hosts

### Study system and sampling

We collected data on GI nematodes infecting six sympatric ungulate species at the Confederated Salish and Kootenai Tribes Bison Range, MT, USA, formerly the National Bison Range (NBR). The Bison Range is a bunchgrass prairie ecosystem (Belovsky and Slade 2020) that is home to a semi-managed population of American bison (*Bison bison*) and unmanaged populations of bighorn sheep (*Ovis canadensis*), elk (*Cervus canadensis*), mule deer (*Odocoileus hemionus*), white-tailed deer (*Odocoileus virginianus*), and pronghorn (*Antilocapra americana*). At the time of data collection for this study, the Bison Range maintained approximately 350 bison which were rotated seasonally among eight fenced pastures within the 86 km^2^ NBR boundary. Movement of the other five ungulate species was unrestricted. To characterize the GI nematode species infecting our focal ungulate host species and quantify parasite abundance, we collected fecal samples opportunistically from all host species between July and August 2007. Focal species were located by driving the NBR road network and samples were collected either from individual animals observed defecating or by searching areas recently used by focal animals for fresh fecal samples as described in Archie and Ezenwa (2011). Samples were stored on ice in a cooler in the field and then at 4°C in the lab before processing.

### Parasitological analysis

To estimate nematode abundance, we used a sugar flotation method to quantify nematode eggs in host fecal samples as described in Foreyt (2013). Briefly, we homogenized 1g of each fecal sample in 10 ml of water, centrifuged the fecal slurry, and resuspended the pellet in a 1.27 specific gravity sugar solution. The sample was then re-spun with a glass coverslip atop the centrifuge tube and nematode eggs attached to the glass slide were counted under the 10× objective of a compound microscope. Egg counts were performed on a total of 104 fecal samples (*n* = 14–20 host per species).

To characterize nematode species composition we collected an average of 25.5 fecal samples (range = 22 to 32 samples) per host species (*N* = 153 samples) between July and December 2007. Following methods described by Archie and Ezenwa (2011), each sample consisted of 10–20 g of feces, which was cultured for 10 days during which nematode eggs developed to the infective larval stage. After 10 days, we isolated as many live larvae as possible from each sample using a modification of the Baermann technique. Larvae were exsheathed in sodium hypochlorite, washed in deionized water, and stored individually at 80°C in a lysis buffer (Redman et al. 2008; Archie and Ezenwa 2011). To extract DNA from individual larvae, each larvae was incubated in lysis buffer at 60°C for 98 min, then at 94°C for 20 min. DNA extracts were diluted 1:5 and stored at −80°C. Larval species were identified by amplifying and sequencing ∼250 base pairs of ITS2 rRNA (Gasser et al. 1993) from larvae using primers NC1-ACGTCTGGTTCAGGGTTGTT and NC2-TTAGTTTCTTTT CCTCCGCT). Positive PCR reactions were cleaned using ExoSAP and sequenced in one direction using Dye Terminator Cycle Sequencing (Applied Biosystems). We identified 2428 larvae from their ITS2 sequences by comparing the sequences to template sequences on GenBank (average number of larvae per host species = 404.67; range = 322 to 507 larvae). Finally, we estimated species-specific nematode abundance for each host and parasite pair (Table 1) by multiplying the proportional abundance of each nematode species estimated from larval sequencing by the average fecal egg count per host.

**Table 1:** Nematode parasite abundance (estimated as eggs shed per gram of host feces) by host species and nematode host range, i.e., number of host species in which a particular parasite is found

Parasite	Host range	Host					
		Bighorn sheep	Bison	Elk	Mule deer	Pronghorn	White-tailed deer
*Cooperia oncophora*	6	1.65	13.56	0.05	0.16	0.33	0.04
*Haemonchus contortus*	6	0.22	0.55	0.75	1.3	137.42	1.38
*Oesophagostomum venulosum*	6	7.59	0.14	2.21	9.12	2.89	1.57
*Ostertagia leptospicularis*	6	1.21	1.39	2.07	1.77	5.15	1.72
*Ostertagia ostertagi*	6	4.29	19.65	0.05	0.21	2.89	0.11
*Trichostrongylus axei*	6	31.46	12.46	4.6	5.63	5.79	0.84
*Spiculopteragia* sp.	4	0.33	0.42	0.67	0	0	1.14
*Nematodirus spathiger*	2	0.44	0	0	0	0.96	0
Unknown sp. A	2	0.11	0	0.03	0	0	0
*Chabertia ovina*	1	0	0.14	0	0	0	0
*Marshallagia* sp.	1	5.83	0	0	0	0	0
*Trichostrongylus* sp.	1	0	0	0	0	8.04	0
Unknown sp. B	1	0	0	0	0	0	0.02

### Estimating ungulate habitat overlap and the likelihood of within- vs between-species exposure

**Fig. 4:**
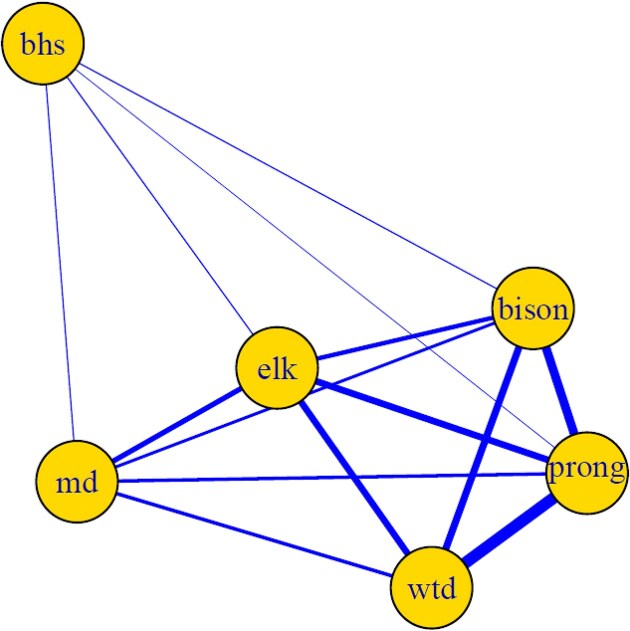
Network showing the likelihood of habitat overlap between all pairs of ungulate hosts. The thickness of the lines displays differences in overlap probabilities, estimated using the simple ratio index (SRI). We assumed that SRI represents the probability of parasite exposure between pairs of species, and then used 1-SRI as serve as a proxy for the probability of within-species exposure (phi). Abbreviations used: bhs: bighorn sheep, md: mountain deer, prong: pronghorn, wtd: white-tailed deer.

To establish a metric quantifying the differential exposure to nematodes from conspecifics, we first estimated the degree of habitat overlap between pairs of host species. To generate habitat overlap information, we used data of ungulate habitat occupancy derived from systematic road transects performed at NBR in June-July of 2006 and 2007. When a focal group of animals was observed during a transect, the species, time of day, size and composition of the group, and a GPS coordinate were recorded. We overlaid the GPS coordinates onto a map of NBR divided into 1km × 1km grid cells using ArcGIS, and then used the presence or absence of all six host species in each of 110 grid cells to estimate the likelihood of any two host species overlapping in space. We used the Simple Ratio Index (SRI) to represent this likelihood as calculated in the R package *asnipe* (Farine 2013) and visualized using *igraph* (Csárdi et al. 2025) (Fig. 4). If we assume that SRI represents the *probability* of between-species parasite exposure between hosts X and Y, then we expect that 1-SRI can serve as a proxy for the probability of within-species exposure. This is likely an overestimate of within-species transmission since it does not account for the presence of other members of the host community beyond the two focal host species, and it does not appropriately account for host densities. Nevertheless, we believe 1-SRI provides a reasonable initial estimate of the relative degree of within- vs. between-species exposure across different pairs of hosts. We therefore explore the sensitivity of estimates of parasite relative fitness to phi by using the 1-SRI value as the high estimate of phi and comparing results for phi = 1−0.5 SRI and 1−0.25 SRI.

### Estimating feasibility and stability of pairwise coexistence from nematode abundance and parasite connectivity

For each ungulate pair-nematode pair combination we used the estimates of nematode abundance in hosts (Table 1), estimates of phi (Fig. 2), and the solution to Eqs. 11–14 to estimate host-specific parasite fitness sets, from which we determined (1) whether parasite-mediated coexistence was possible (i.e., equilibrium feasible using Eq. 2) and (2) the strength of stabilization if it was feasible (i.e., strength of negative feedback using Eq. 3). As illustration, if we were to consider Bighorn sheep-Bison pair of ungulates and the *Ostertagia ostertagi-Trichostrongylus axei* pair of nematodes and φ = 0, then observed abundances of nematodes would be estimates of nematode fitness (*k = 4.29, l = 19.65, m = 31.46*, and *n = 12.46*) and with the assumption that parasite fitness impacts on hosts are negatively related to parasite fitness gains on that host (Fig. 3), we would estimate that parasite-mediated coexistence was possible with an equilibrium proportion of Bighorn sheep ($\widehat {{{p}_A}}$) of 0.21 and a stabilizing negative feedback (*I_S_*) of −1,180. Adjustment of fitness estimates for amplification from exposure to conspecific microbiomes can yield non-feasible equilibrium and greatly reduce the stabilizing effect.

There are 15 ungulate pairs [(6*5)/2] and 78 GI nematode pairs [(13*12)/2], yielding 1170 total combinations. We created a loop in Matlab (Appendix 4) that extracted the corresponding φ values and nematode loads from each of these groups and solved the nematode fitness response equations (*k, l, m*, and *n*) described in Appendix 3. For final analyses, we retained solutions only when the estimate of P_A_ for a given host–parasite group was a real (non-complex) number between 0 and 1, i.e., that had a feasible equilibrium.

### Statistical analysis of patterns of feasibility and stability of pairwise coexistence

To examine if characteristics of either host or parasite pairs predicted coexistence (as determined by whether viable solutions could or could not be reached), we performed a generalized linear mixed effects model with binomial errors (Brooks et al. 2017). Because multiple solutions were possible for any given two-host-two-parasite group, the identity of the host pair and the parasite pair were included as random effects. In our initial, maximal model, we included the following fixed effects as predictors: φ assumption (a factor with three levels: as estimated, 0.5*estimated, or 0.25*estimated), host phylogenetic distance, host habitat overlap, and the parasite pair's host range, as well as all two-way interactions with φ assumption. Parasite pair’s host range was a factor with the levels of “narrow-narrow,” “narrow-broad,” or “broad-broad,” with narrow and broad defined for each member of the parasite pair based upon being found in <3 hosts or >3 host species, respectively (see Table 1). Final fixed effects were retained based upon AICc comparison of all possible combinations of fixed effects.

To assess how both habitat overlap between hosts and phylogenetic distance between hosts predicted the estimates of parasite mediated feedback [(1−φ)*I_s_], we constructed linear mixed effects models in R (Team 2010; Bates et al. 2015; Kuznetsova et al. 2017). Because estimates of parasite mediated feedback were skewed and generally negative, but always less than 1, we used a transformation to ensure normality of residuals from these models. Specifically, our response variable was ln(estimated feedback + 1). We reverse-transformed model predictions for graphing (-e^raw predictions + 1). Initial, maximal models included habitat overlap, φ assumption (as estimated φ, 0.5*estimated φ, or 0.25*estimated φ) and their interaction as fixed effects. These models also included random effects of intercepts for both host pair ID and parasite pair ID as well as a random slope for parasite pair ID. This accounted for non-independence of data since each host and parasite pair generated multiple data points. We compared maximal models with less complicated random effects structures using AICc. The initial structure was most supported for models with habitat overlap as a fixed effect; a model with only random intercepts for host and parasite pair IDs was most supported when phylogenetic distance was a fixed effect. After assessing the random effect structure, we simplified fixed effects by removing the interaction term, which yielded lower AICc values in both cases.

Finally, to assess whether the host-range of a given parasite pair predicted the strength of parasite-mediated feedback, we performed a linear mixed effects model with the dependent variable of mean estimated parasite-mediated feedback [(1−φ)*I_s_] across all host pairs for a given parasite pair. The model included parasite pair as a random effect and fixed effects of φ assumption (defined above) and the parasite pair's host range (defined above) and their interaction.

## Results

### Estimation of feedback strength and potential for coexistence

Of the 1170 possible host pair-parasite pair combinations, 397 (∼34%) yielded viable, real solutions, consistent with feasible parasite-mediated ungulate coexistence. Lower values of φ increased the probability of finding a real solution (likelihood ratio test: χ^2^ = 7.58, df = 2, *P* = 0.02; parameter estimate (φ = 0.5*estimated) = 0.15, SE = 0.097, odds-ratio compared to φ as estimated = 1.17; parameter estimate (φ = 0.25*estimated) = 0.26, SE = 0.096, odds-ratio compared to φ as estimated = 1.30). Increasing host divergence time increased the probability of finding a real solution (χ^2^ = 8.94, df = 1, *P* = 0.003; parameter estimate = 0.04, SE = 0.01, odds-ratio per MY = 1.04). Increasing habitat overlap predicted a lower probability of finding a real solution, although with lower precision than the other variables (χ^2^ = 3.37, df = 1, *P* = 0.067; parameter estimate = −1.13, SE = 0.58, odds-ratio per 0.01 units overlap = 0.99). A parasite’s host range was not retained in the top model, though was present in the second-most supported model (ΔAICc < 0.1), with narrower host ranges tending to decrease the probability of obtaining a real solution, though this was estimated with low precision (χ^2^ = 4.00, df = 2, *P* = 0.14; parameter estimate (“narrow-broad”) = −0.06, SE = 0.30, odds-ratio compared to “broad-broad” = 0.94; parameter estimate (“narrow-narrow”) = −0.69, SE = 0.38, odds-ratio compared to “broad-broad” = 0.50).

**Fig. 5:**
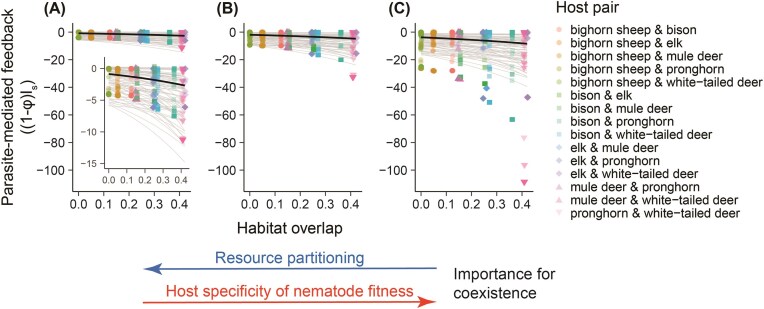
Estimated strength of parasite-mediated feedback, (1−φ)I_s_, becomes more pronounced as habitat overlap between host pairs increases. The (1−φ)I_s_ estimates were generated under current estimates of φ (A) and under the assumptions that true φ = 0.5*estimated (B), or true φ = 0.25*estimated (C). Although mean values of parasite mediated feedback declined, i.e., became more stabilizing, with decreasing φ, the relationship with habitat overlap is negative in each scenario. Black lines show predictions based solely on fixed effects from linear mixed effects models, gray lines show predictions including random effects for all parasite pairings for pronghorn & white-tailed deer hosts, the host pair with the widest range of (1−φ)I_s_. Including predictions for all possible host–parasite combinations made lines indistinguishable. Points were jittered slightly along the *x*-axis to show separation.

**Fig. 6:**
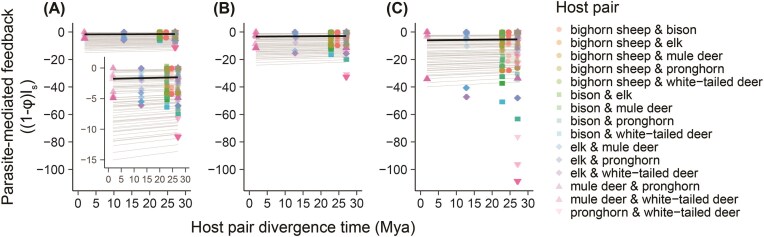
Estimated strength of parasite-mediated feedback, (1−φ)I_s_, showed no strong relationship with phylogenetic distance between host pairs (divergence time in millions of years). (1−φ)I_s_ estimates were generated under current estimates of φ (A) and under the assumptions that true φ = 0.5*estimated (B), or true φ = 0.25*estimated (C). Black lines show predictions based solely on fixed effects from linear mixed effects models, gray lines show predictions including random effects for all parasite pairings for pronghorn and white-tailed deer hosts, the host pair with the widest range of (1−φ)I_s_. Including predictions for all possible host–parasite combinations made lines indistinguishable. Points were jittered slightly along the *x*-axis to show separation.

As expected, parasite-mediated feedback was stronger (i.e., “estimated value of (1−φ)*I_s_“ became more negative) when we assumed that the true value of φ was lower than we had estimated (Fig. 5, F_2,1719.1_ = 148.2, *P* < 0.001), mimicking situations in which exposure to parasites from heterospecific hosts is relatively higher. Regardless of our choice of φ, however, feedback became stronger as habitat overlap increased (parameter estimate (for transformed (1−φ)*I_s_ values) = 1.61, SE = 0.52; F_1,19.2_ = 9.6; *P* = 0.006), with considerable variation in the intercept of this pattern among pathogen and host pairs (Fig 5). In contrast, we did not find strong evidence for a relationship between parasite-mediated feedback and phylogenetic distance between hosts, regardless of the assumed value of φ (Fig. 6; F_1,13.4_ = 0.80, *P* = 0.78, parameter estimate (for transformed (1−φ)*I_s_ values) = −0.003, SE = 0.013).

**Fig. 7:**
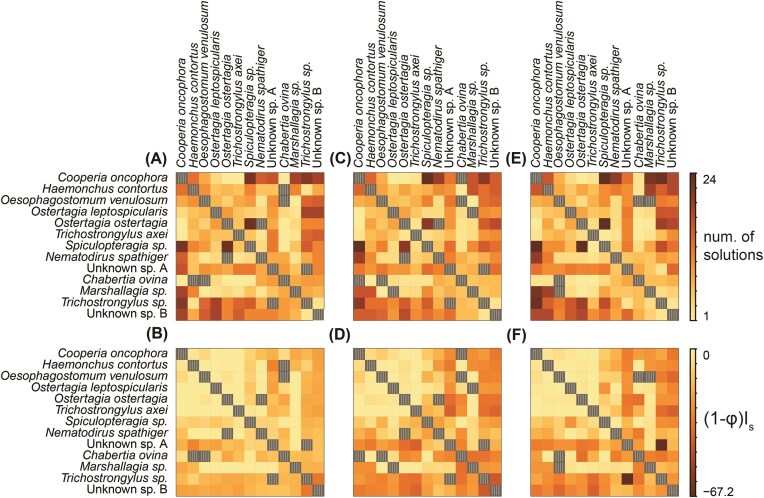
The number of real solutions (A, C, E) and mean estimated parasite mediate feedback, (1−φ)I_s_, (B, D, F) obtained for a given parasite pair across all host pairs under current estimates of φ (A, B), true φ = 0.5*estimated (C, D), or true φ = 0.25*estimated (E, F). Hatched squares indicate pairs for which we obtained no real solutions across any host pairs. Species are organized from broadest host ranges (upper left corners) to narrowest host ranges.

We found that the mean strength of parasite mediated feedback varied with the combined host range of a given pair of parasites (Fig. 7). Specifically, mean feedback tended to be stronger when one or both parasites of the pair showed a relatively narrow host range (i.e., only infecting 1–2 host species, Table 1; F_2, 54.9_ = 13.7, *P* < 0.001, parameter estimate(“narrow-broad”) = −1.85, SE = 2.07; parameter estimate(“narrow-narrow”) = −4.36, SE = 2.63). This pattern was most pronounced at lower values of φ (φ category x host range category interaction: F_4,120.3_ = 14.91, *P* < 0.001; parameter estimate(0.5*φ × “narrow-broad”) = −3.53, SE = 1.97; parameter estimate (0.5*φ × “narrow-narrow”) = −3.48, SE = 2.50, parameter estimate (0.25*φ × “narrow-broad”) = −13.70, SE = 1.96; parameter estimate (0.25*φ × “narrow-narrow”) = −14.34, SE = 2.49).

## Discussion

We provide a first attempt to apply microbiome feedback theory to animal communities. Feedback theory and tests have proven productive in elucidation of the importance of soil pathogens in plant community structure and function (Mangan et al. 2010; Bever et al. 2015; Eppinga et al. 2018; Crawford et al. 2019; Wang et al. 2023). Plant–soil feedback practice has relied heavily on an experimental approach (Fig. 2) that integrates parasite responses to hosts and host responses to evaluation of the potential for microbiome feedback to drive coexistence (Bever 1994; Bever et al. 1997). While we were not able to use this experimental approach in the current study, we utilized observational data of nematode species abundance among ungulate hosts to estimate fitness differences among hosts, and the potential for host–parasite feedbacks to drive community dynamics. We find evidence that nematode dynamics can generate negative feedback, potentially contributing to coexistence of ungulates in western North America. Moreover, we find that nematode-mediated coexistence should increase in importance for host species that share habitat more frequently.

**Fig. 8:**
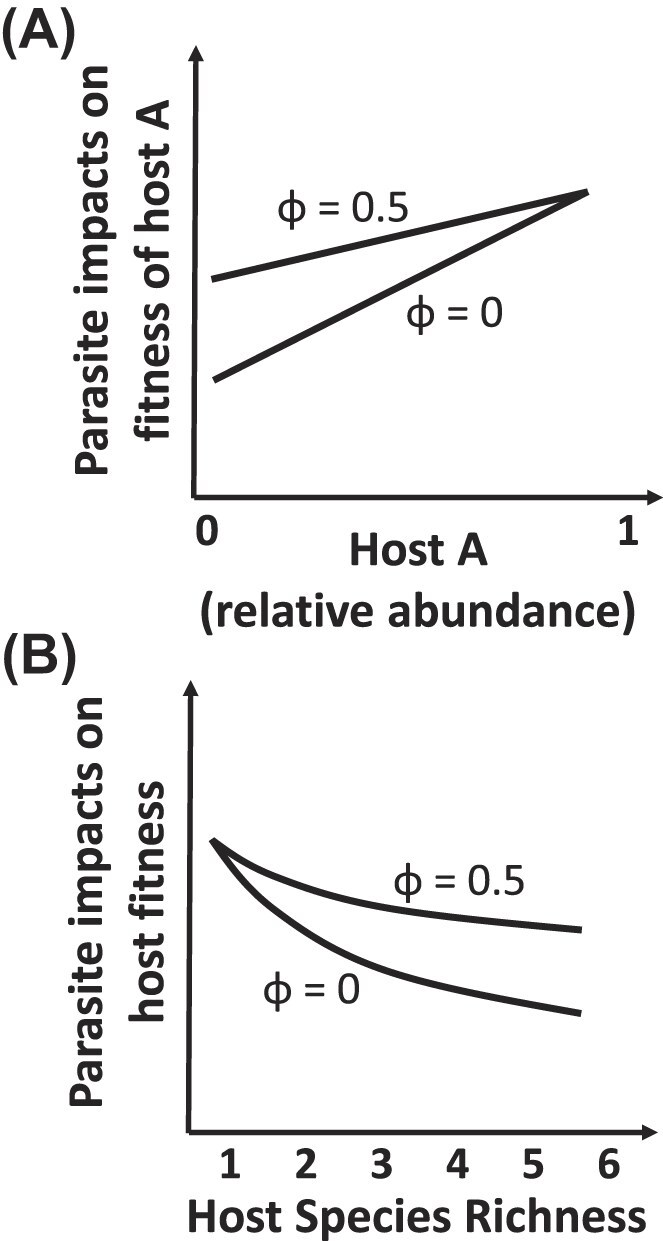
When changes in parasite composition drive negative feedback, negative impacts of the parasites on a given host increases with the relative abundance of that species (A). With greater exposure to conspecific microbiome than expected by chance (i.e., Φ>0), then the release from parasites with declining abundance of host A is diminished, as represented in (A) by Φ =0.5. A basic consequence of this relationship is that with increasing richness of competing hosts is that the average abundance of each host declines, thereby diluting the negative effects of parasites. This parasite dilution is diminished by greater exposure to conspecific microbiomes than expected by chance, as illustrated in (B) by Φ =0.5.

We modified classic microbiome feedback theory to accommodate an increased likelihood of exposure to conspecific parasites, which might be typical of animals that live in groups, like the ungulate species in our study. Interestingly, we find that enhanced exposure to parasites from conspecific hosts decreases the stabilizing force of negative feedback (Eq. 2). As exposure to parasites from different hosts, as our framework considers, is a critical and necessary step in disease transmission, inference from our model may seem contrary to analysis of models of two hosts-one parasite which find that dynamics of a single parasite population can drive coexistence of two host species when within-species parasite transmission exceeds between-species parasite transmission (Holt and Pickering 1985). One distinction is that exposure does not necessarily result in transmission, and the host-specific differences in net fitness responses of parasites to hosts could result from failure to infect (i.e., failure to transmit), or reduced virulence after infection. A second qualitative difference between Holt and Pickering results and ours stem from our consideration of multiple parasites on competing host species. Parasite specialization, as reflected in differential fitness on hosts, drives coexistence in multi-parasite, multi-host systems (Bever 1999). With such parasite specialization, the negative impact of parasites on a host species increases with an increasing proportion of that host species (Fig. 8A). When it is the only host species (i.e., 100% of the host community), the host is only exposed to microbiomes from conspecifics which are enriched in parasites to which it is most vulnerable. As the host species becomes rarer, it is increasingly exposed to microbiomes of heterospecific species that have less deleterious parasites. However, when hosts are exposed to conspecific microbiomes more than expected by chance (i.e., φ>0), then the benefit of rarity is reduced, as the exposure to microbiomes from heterospecific species and their less deleterious parasites is diminished (Fig. 8A), thereby diminishing the stabilizing force of negative feedback.

Our model assumes that hosts are competing so that increases in one host species lead to a decrease in another host species [in contrast to assumptions of independent carrying capacities in other models of multi-host dynamics, e.g., (Dobson 1985)]. As a result, when parasite mediated negative feedbacks exist, then net parasite impacts will be reduced, i.e., diluted, by increasing host species richness, as the average relative abundance of hosts species declines with 1/S, where S is the host species richness (Fig. 8B). This dilution of pathogen impacts was shown empirically and theoretically for plant communities (Wang et al. 2023). Here, we find that the release from impacts of specialist parasites with decreasing host proportion, and hence host richness, will be diminished when hosts have greater likelihood of exposure to conspecific microbiomes (i.e., φ>0, Fig. 8B).

An interesting consequence of the reduced strength of parasite mediated negative feedback with increased exposure to conspecific microbiomes is that parasite dynamics may become a stronger driver of coexistence of host animals as resource partitioning becomes weaker. In our ungulate system, habitat overlap as measured by species co-occurrence, may reflect competition for common food resources, thereby reducing the likelihood of coexistence. However, increasing habitat overlap will also increase the likelihood of exposure to parasite communities from heterospecific rather than conspecific hosts, which can provide release from deleterious nematodes. Thus, high habitat overlap generates a greater likelihood of parasite-driven negative feedback contributing to coexistence (Fig. 5). We find that species with substantial levels of habitat overlap in our study, such as white-tailed deer and elk, which have the potential for moderate to high resource competition (Jenkins and Wright 1988; Jenkins et al. 2007), also have a greater likelihood of coexistence through parasite driven negative feedbacks. In this case, competition provides release from enemies. In contrast, for species such as white-tail deer and bighorn sheep with negligible habitat overlap and very low exposure to each other's parasitic communities, the likelihood of coexistence through parasite driven negative feedback was extremely low. Our analyses could be extended through integration of the Phi modification of the microbiome feedback model with explicit consideration of competition between host species [e.g., (Bever 2003)] to evaluate potential interactive effects of resource partitioning and negative microbiome feedback on coexistence.

Within the microbiome feedback framework, host-specificity of parasite fitness, as measured by the interaction coefficient I_M_ (Fig. 1B), is the critical metric determining nematode differentiation with host species, and the strength of resulting feedback. We note that this metric could be large because of a narrow host-range (i.e., classic host specialists). But the metric could also be large for nematodes with wide host ranges (i.e., generalists). The host ranges for nematodes in this study varied from infecting all six ungulate host species (complete generalists) to infecting only one host species (complete specialists, see Table 1). We generally see that the strongest estimates of negative feedback involve nematodes with narrow host ranges (e.g., *Trichostrongylus* sp. and Unknown sp. B), but we also see some degree of negative feedback generated between pairs of generalists (e.g., *Cooperia oncophora* and *Oesophagostomum venulosum*, Fig. 7), suggesting that both specialist and generalist nematodes can contribute to the consistent parasite-driven negative feedback that we predict between ungulate species. Feedback involving host-specialist and generalists are likely to generate negative feedback between the most pairs of hosts (Fig. 7A, C, E, lower-left and upper-right quadrants) and the estimated feedback becomes increasingly negative with decreasing φ (Fig. 7B, D, F)

Our estimate of the enhanced probability of exposure to conspecific parasites has a large impact on the estimation of parasite fitness and predicted dynamics (Figs. 5–8). In this study, we used visual observations of host species co-occurrence in space as a proxy for the likelihood of microbiome exposure. We acknowledge that this is an imperfect measure for environmentally-transmitted parasites such as GI nematodes. First, there will be a time lag between introduction of nematode eggs into the environment via defecation, and the ingestion of infective larvae (Bowman 2021). Second, the habitat use data were collected in the summer when these ungulates are at the maximal spatial separation, as ungulates that occupy high elevations in the summer (e.g., bighorn sheep) overwinter at lower elevations. Finally, our proxy for exposure to parasites from conspecifics does not capture the nuances of within-species contact in animals that form social groups. We therefore expect that habitat overlap measured over the summer underestimates interspecific exposure. In fact, population genetic analyses of nematodes are consistent with high levels of transmission between ungulate species (Cerutti et al. 2010, Archie and Ezenwa 2011). Thus, assuming our estimated Φ is an upper bound, our analyses (Figs. 5, 6, 7) represent a conservative description of the potential contribution of nematode feedback to ungulate coexistence.

Our analyses assumed a negative relationship between parasite and host fitness effects (Fig 3) and our inference of the potential for GI nematode dynamics to contribute to coexistence of ungulates depends upon this assumption. While there is direct evidence in support of this relationship in other host–parasite systems [plant–fungal pathogens (Thrall and Burdon 2003), plant–bacterial pathogens (Platt et al. 2012), mice–protozoan parasite (Råberg et al. 2007); insect–protozoan parasite (de Roode et al. 2008)], there is less information for GI nematode-ungulate interactions. There is evidence of deleterious impacts of GI nematodes on wild ungulate hosts (Koltz et al. 2022), but tests of the reciprocal effects of the host–nematode interaction on both host and parasite are rare. While it is generally observed that GI nematode load is negatively correlated with host health (Mavrot et al. 2015), there is also variation in this relationship within species consistent with genetic variation in host tolerance (Hayward et al. 2014; Budischak et al. 2018), and variance in tolerance can weaken the relationship between host and parasite fitness effects [e.g., (Råberg et al. 2007)]. We note however, that variation in slope of the relationship between host and pathogen fitness effects (e.g., Fig. 3) will not change qualitative expectations for coexistence, though the strength of the stabilizing effect will change proportionately with the slope (e.g., a slope of ½ will reduce the stabilizing effect by ½). It is quite possible, however, that non-linearity in this relationship would have larger effects on stability of nematode community feedbacks. Further work is necessary to test the strength and linearity of the dependence of nematode and host fitness of individual host–nematode combinations.

### Implications for management and a changing climate

Inferring parasite fitness responses to hosts required us to assume that GI nematode abundances were at equilibrium for each pair of nematodes and pair of ungulate hosts (Fig. 1B). Given that each host–nematode combination was used in 60 pairwise comparisons, this assumption could not be simultaneously true for each of the 1170 combinations of ungulate and GI nematode pairs. Hence inference from our analyses must be viewed with caution. Eppinga et al. (2018) extended the two species host–microbiome feedback model to multi-species communities. However, our preliminary exploration of whether the model of interguild frequency dependence (Bever 1999) could also be expanded to multi-hosts and multi-parasite was not productive [but see (Golubski 2002) for some mathematical progress in this direction]. It is also possible that our estimates using pairwise microbiome feedback could be projected using the multispecies feedback framework (Eppinga et al. 2018). This might be a productive avenue for further work.

Specifically within the context of projecting multi-host dynamics, our evidence of GI nematode-driven negative feedback and the connection between parasite driven negative feedback and parasite dilution, as illustrated in Fig. 8B and demonstrated in plants (Wang et al. 2023), implies that parasite fitness effects on hosts (measured as nematode egg abundance in feces in this study) should increase with the loss of ungulate diversity in North America. Given that native ungulate diversity has been significantly reduced relative to pre-colonization in most regions of North America, our work predicts that impacts of GI nematodes on ungulate hosts should be more severe in regions with less remaining ungulate diversity. Such a prediction is testable in white-tailed deer, the only remaining ungulate in the many parts of North America. After controlling for relevant abiotic and biotic factors (e.g., rainfall, humidity, host density), GI nematode species that affect deer throughout their range (i.e., their native parasites) should have more severe impacts on individuals in regions with lower compared to higher ungulate diversity.

To the extent that parasite dynamics influence host ecology, then understanding the dependence of these dynamics on climate is critical to efforts to project the stability of host populations. The strength of the microbiome feedback approach is to describe the potential of a parasite community to influence host community stability, as illustrated in this study. Repeating the measurements and analyses that we developed here across climatic gradients would offer the comparisons to test for climatic dependence of parasite-driven feedback. This comparative approach could be used to test specific hypotheses motivated by functional analyses of nematode–ungulate interactions. Climate dependence of parasite-driven feedbacks, for example, could be informed by natural history of the environmental transmission (e.g., tolerance of individual nematode species to UV radiation, desiccation, or freezing). Functional modelling of ungulate–nematode interactions could also provide foundations for predicting climate sensitivities.

## Conclusion

Classically, coexistence of animal competitors is thought to result from reduced interspecific competition due to resource partitioning. Ungulates illustrate this resource partitioning beautifully, with bighorn sheep adapted to difficult to access montane habitats, while elk and deer browse shrubs and trees, and bison and pronghorn specialize on the grasslands. However, these resource divisions are incomplete—e.g., all the ungulates are concentrated into lowland habitats during the winter—and all compete to some degree. We find evidence that host-specific differences in nematode fitness could generate negative feedback and contribute to coexistence of ungulate hosts, and that this is more likely to be the case for ungulates that have higher levels of habitat overlap. While more work is necessary to test the underlying assumptions and mechanism of parasite-mediated negative feedback, our work highlights another pathway by which parasites could structure animal communities (Wood et al. 2007; Fenton and Brockhurst 2008; Friesen et al. 2021). Moreover, should specificity of nematode fitness relationships drive negative feedbacks, then nematode impacts on hosts would decline, i.e., be diluted, with increasing ungulate diversity, and conversely, declines of ungulate diversity could lead to increases in increasing detrimental impacts of GI nematodes on the remaining host-species.

## Supplementary Material

icaf087_Supplemental_Files

## Data Availability

All data are incorporated into the article and its online supplementary material.

## Notes

From the symposium “Paddling Together: navigating the crosscurrents of plant and animal biology to explore uncharted waters in disease ecology” presented at the annual meeting of the Society for Integrative and Comparative Biology, January 3-7th, 2025.
