# Supplementary material for: Application of Microbiome Feedback Theory to Animals: Can Parasites Drive Coexistence in Ungulate Communities?
Source: Integr Comp Biol. 2025 Jun 10;65(2):445–60. doi: 10.1093/icb/icaf087 (PMC12448209; doi:10.1093/icb/icaf087)
Supplement: icaf087_Supplemental_Files [file icaf087_supplemental_files.zip › icb-2025-0058-File012.docx]

**Appendix 1**

In the main text section ‘*Modification of the microbiome feedback model to accommodate greater probability of transmission to conspecifics*’, we described how non-random transmission of microbiome components modified the equilibrium and stability conditions of the microbiome feedback model. In this Appendix, we provide the mathematical derivations leading to these findings.

As noted in the main text, the feedback model with non-random transmission can be written as:

$\frac{dP_{A}}{dt}=P_{A}P_{B}\left( w_{A}-w_{B} \right)$ (1)

In which $P_{A}$ and $P_{B}$ are the proportions of ungulate species *A* and *B* in the community, and $w_{A}$ and $w_{b}$ describe their relative fitness:

$w_{A}=\varphi\alpha_{11}+\left( 1-\varphi\right)\left( \alpha_{11}p_{M_{A}}+\alpha_{12}p_{M_{B}} \right)$ (2)

$w_{B}=\varphi\alpha_{22}+\left( 1-\varphi\right)\left( \alpha_{21}p_{M_{A}}+\alpha_{22}p_{M_{B}} \right)$ (3)

In which $p_{M_{A}}$ and $p_{M_{B}}$ are the proportions of the microbiomes depending on hosts *A* and *B* in the microbiome community. In Mack and others (2019), it was shown that when assuming that microbiome dynamics are relatively fast, and the ratio between per capita impacts of microbiomes and host carrying capacity is constant across species, microbiome effects are proportional to host densities, and equations (2) and (3) can be written as:

$w_{A}=\varphi\alpha_{11}+\left( 1-\varphi\right)\left( \alpha_{11}P_{A}+\alpha_{12}P_{B} \right)=\varphi\alpha_{11}+\left( 1-\varphi\right)\left( \alpha_{11}P_{A}+\alpha_{12}\left( 1-P_{A} \right) \right)$ (4)

$w_{B}=\varphi\alpha_{22}+\left( 1-\varphi\right)\left( \alpha_{21}P_{A}+\alpha_{22}P_{B} \right)=\varphi\alpha_{11}+\left( 1-\varphi\right)\left( \alpha_{11}P_{A}+\alpha_{12}\left( 1-P_{A} \right) \right)$ (5)

Where we also used that in a community with two hosts $P_{A}+P_{B}=1$. Substituting equations (4) and (5) into equation (1), we obtain:

$\frac{dP_{A}}{dt}=-\frac{dP_{B}}{dt}{=P}_{A}\left( w_{A}-w_{A}P_{A}-w_{B}\left( 1-P_{A} \right) \right)$ (6)

To find the system’s equilibrium points, we set $\frac{dP_{A}}{dt}=0$, and solve for $P_{A}$. Equation (6) directly shows that $\hat{P}_{A}$= 0 is a solution (where the hat indicates an equilibrium density). The second term on the right hand side is a quadratic equation in $P_{A}$, from which two additional solutions can be obtained:

$\hat{P}_{A}=\frac{I_{S}^{*}+E_{A,B}+\sqrt{\left( I_{S}^{*}+E_{A,B} \right)^{2}-4I_{S}^{*}E_{A,B}}}{2I_{S}^{*}}=1$ (7)

$\hat{P}_{A}=\frac{I_{S}^{*}+E_{A,B}-\sqrt{\left( I_{S}^{*}+E_{A,B} \right)^{2}-4I_{S}^{*}E_{A,B}}}{2I_{S}^{*}}=\frac{E_{A,B}}{I_{S}^{*}}$ (8)

Following the notation in Eppinga and others (2018), in equations (7) and (8) $E_{A,B}$ represents the microbiome effects on host species A, in an environment dominated by host species B. Technically, this state is referred to as the invasion condition for host species A (e.g. (Eppinga and others 2006)). In contrast to previous model formulations, however, $E_{A,B}$ is now dependent on the degree of non-random transmission between hosts:

$E_{A,B}=\alpha_{22}-\alpha_{12}+ \varphi\left( \alpha_{12}-\alpha_{11} \right)$ (9)

We note that under random transmission (i.e. $\varphi=0$), equation (9) reduces to previously obtained results for the microbiome feedback model (Eppinga and others 2018). In similar vein, we can derive the invasion condition for host B:

$E_{B,A}=\alpha_{11}-\alpha_{21}+ \varphi\left( \alpha_{21}-\alpha_{22} \right)$ (10)

This invasion condition also determines the equilibrium density of host B:

$\hat{P}_{B}=\frac{E_{B,A}}{I_{S}^{*}}$ (11)

In equations (8) and (11), $I_{S}^{*}$ represents the interaction coefficient between the two host populations. Due to the assumption of non-random transmission, this is a modified version of the interaction coefficient $I_{S}$as previously presented (Bever and others 1997). Similarly to the original feedback model, however, the interaction coefficient is defined by the sum of the invasion conditions for both species. This observation can be verified given that $\hat{P}_{A}+\hat{P}_{B}=1$, hence the right-hand sides of equations (8) and (11) need to sum up to 1 as well. Using the right hand sides of equations (9) and (10) we can then express $I_{S}^{*}$ as:

$I_{S}^{*}=E_{A,B}+E_{B,A}=\left( 1-\varphi\right)\left( \alpha_{11}-\alpha_{12}-\alpha_{21}+\alpha_{22} \right)=\left( 1-\varphi\right)I_{S}$ (12)

An equilibrium point in which hosts A and B are both present in non-zero densities requires that $\hat{P}_{A}$ and $\hat{P}_{B}$ are between 0 and 1. This requirement is also referred to as the feasibility condition, and hence meeting the feasibility condition is a necessary requirement for host coexistence (e.g. (Eppinga and others 2018)). From equations (8), (11) and (12), it is clear that the feasibility condition can only be met if the values for $E_{A,B}$, $E_{B,A}$ and $I_{S}^{*}$ are all positive, or if they are all negative. For feasible equilibrium points, it is of interest to identify whether these are unstable or stable equilibrium points. Stability requires negative density dependence of both hosts around the equilibrium point. For example, if the density of host A is increased beyond the equilibrium level, a subsequent decline in density would be needed to return to the equilibrium point. If this indeed occurs, the system can be considered (locally) stable. We can thus evaluate the stability of an internal equilibrium point by evaluating how the growth of host A responds to changes in host A density around the equilibrium point. This change is defined as:

$\frac{d}{dP_{A}}\left( {\frac{dP_{A}}{dt}}_{|P_{A}=\hat{P}_{A}} \right)=\frac{E_{A,B}E_{B,A}}{I_{S}^{*}}$ (13)

Considering the two cases enabling feasible equilibrium points, it is clear that when $E_{A,B}$, $E_{B,A}$ and $I_{S}^{*}$ are all positive, the growth of host A when perturbed above the equilibrium point is positive as well. This means that the perturbation (i.e. the increase in proportion above the equilibrium point) is amplified, and there is a positive feedback that will lead to exclusion of host B. Note that this positive feedback would also work the other way around: if the density of host A would be reduced below the equilibrium point, its growth rate would become negative, and the system would develop to a state where only host B persists.

Alternatively, when $E_{A,B}$, $E_{B,A}$ and $I_{S}^{*}$ are all negative, the growth of host A when perturbed above the equilibrium point is negative as well, and the system would return to the equilibrium point. Similarly, a perturbation pushing host A below the equilibrium point would lead to a positive growth rate, and the system would again return to the equilibrium point.

Finally, we note that in this latter case, there are only three equilibrium points, the other two equilibria are the corner points with $\hat{P}_{A}=0$ and $\hat{P}_{A}=1$. Hence, if the internal equilibrium point is stable, the corner equilibria are unstable. This means that if there is a feasible equilibrium point, this is a globally stable equilibrium point if and only if $I_{S}^{*}<0$.

**Appendix 2**

In the main text section ‘*Derivation of expected values of parasite abundance in the field*’ we described how non-random transmission of microbiome components over longer timescales will approach an equilibrium level of pathogens within each host. In this Appendix, we derive these equilibrium levels of pathogens by tracking pathogen dynamics over recursive transmission events.

We will first consider the accumulation of one species of pathogen (labeled pathogen X, ${PX}_{A}$) that grows on one host species (labeled host A, $P_{A}$). The relative fitness level of the pathogen within this host is described by the parameter *k*. As described in the main text, the model framework assumes exponential pathogen growth in hosts over time, and assumes in general that microbiome dynamics occur on a faster timescale than host dynamics. As mentioned in Appendix 1, it was previously shown that this latter assumption means that pathogen densities become proportional to current host densities (Eppinga and others 2018; Mack and others 2019). In the case of random transmission and without consideration of history, the proportional dependence of pathogen X on host A would be determined by the relative fitness parameter *k* (see Fig. 1 in the main text). Considering the slow timescale associated with host dynamics, and a situation where host A is in equilibrium, the density of pathogen X would then simply be given by ${PX}_{A}=k\hat{P}_{A}$, where the hat indicates equilibrium. However, for the animal-parasite systems considered in this study, we need to consider non-random transmission dynamics and their cumulative impacts over time. Non-random transmission implies that a pathogen is able to accumulate in a host over multiple transmission cycles, leading to accelerated growth and an increase in relative fitness. The stronger the tendency for transmission within conspecific hosts, the stronger this effect will be. Transmission within hosts depends on the rate of non-random transmission, $\varphi$, and the within host-transmission that occurs randomly, and therefore depends on the density of host A in the community, $P_{A}$. We can combine these two processes to describe a specific conspecific transmission rate $r$:

$r=\varphi+\left( 1-\varphi\right)P_{A}$ (1)

Hence, instead of a constant proportionality $k$ we need to derive a specific function of proportionality that takes into account the degree of within-host transmission and its cumulative impacts on exponential pathogen accumulation, i.e. ${PX}_{A}=G\left( r \right)$. In the following derivation for $G\left( r \right)$, we assume that if host A is exposed to a heterospecific microbiome, the exponential growth process of pathogen X is broken, and its relative fitness returns to the background fitness *k*.

In the following, we approach the phenomenon by considering a current system state, at time *t*, that was preceded by many preceding transmission cycles. As a result, it can be assumed that the relative fitness of pathogen X in host A has reached an equilibrium level. Focusing on the four preceding cycles, Figure A1 illustrates all the possible trajectories towards the current system state. In this Figure, the bottom row indicates the trajectory for which the fitness for pathogen X in host A would be lowest, namely the background fitness *k*. This occurs when there was heterospecific microbiome transmission in the previous cycle, i.e. at *t*-1 (Fig. A1). Note that in this case, the transmission events before *t*-1 are no longer relevant, as those preceding dynamics have been reset by the transmission event at *t*-1. Given that the probability of heterospecific transmission in any transmission cycle is $\left( 1-r \right)$, the above scenario applies to a proportion $P_{0}=\left( 1-r \right)$ of the population of host A, and fitness of pathogen X in this proportion is the background fitness $F_{0}=k$. Hence, the fitness contribution of this part of the host population to pathogen X is:

$G_{0}\left( r \right)=P_{0}F_{0}=\left( 1-r \right)k$ (2)

Where $G_{0}$ indicates the case where there were 0 previous cycles of conspecific transmission. We can then continue with quantifying the relative fitness of pathogen X in the remaining proportion of the host A population, i.e. $1-P_{0}=r$. All hosts within this remaining proportion of the population experienced a conspecific transmission event at *t*-1. The exponential growth process then implies that growth over these two time steps is $k^{2}$. Analogous to the above derivation of $G_{0}\left( r \right)$, we consider the proportion of the population that experienced heterospecific transmission in the preceding timestep, in this case *t*-2 (Fig. 1). Again, this proportion is $\left( 1-r \right)$. Given that we considered a remaining proportion of the population $\varphi$ at this timestep, we have $P_{1}=\left( 1-r \right)r$. As noted above, the relative fitness for pathogen X within this group is $F_{1}=k^{2}$. Hence, the fitness contribution of this part of the host population to pathogen X is:

$G_{1}\left( r \right)=P_{1}F_{1}=\left( 1-r \right)rk^{2}$ (3)

The next step then considered the proportion of the population that experienced self-transmission in the preceding two transmission cycles (Fig. 1). The remaining proportion of the host population to consider at this point is: $1-P_{0}-P_{1}=r^{2}$. Hence, the proportion experiencing heterospecific transmission in the preceding timestep *t*-3 is $P_{2}=\left( 1-r \right)r^{2}$ and the relative fitness of pathogen X in this group is $F_{2}=k^{3}$. Hence, the fitness contribution of this part of the host population to pathogen X is:

$G_{2}\left( r \right)=P_{2}F_{2}=\left( 1-r \right)r^{2}k^{3}$ (4)

Hence, it follows that we can formulate a generalized fitness contribution function as:

$G_{n}\left( r \right)=P_{n}F_{n}=\left( 1-r \right)r^{n}k^{n+1}$ (5)

Subsequently, we can sum the fitness contributions of all trajectories to describe pathogen population X:

$G\left( r \right)=\sum_{i=0}^{\infty} G_{i}\left( r \right)=\sum_{i=0}^{\infty} \left( 1-r \right)r^{i}k^{i+1}=\frac{k\left( 1-r \right)}{1-rk}$ (6)

Substituting equation (1) into equation (6) then yields:

${PX}_{A}=G\left( r \right)=G\left( \varphi,p_{A} \right)=\frac{k\left( \hat{P}_{A}-1 \right)\left( \varphi-1 \right)}{1-k\left( \varphi+\left( 1-\varphi\right)\hat{P}_{A} \right)}$ (7)

Where the right hand side of equation (7) is the expression presented in the main text. We can then derive a similar equation for the accumulation of pathogen Y on host A. This pathogen will have the same conspecific transmission rate (i.e. that of host A), as described by equation (1). The only difference is that its relative fitness level is described by the parameter *m* (instead of *k*). Hence, it follows that for the accumulation of pathogen Y on host A, we can write:

${PY}_{A}=\frac{m\left( \hat{P}_{A}-1 \right)\left( \varphi-1 \right)}{1-m\left( \varphi+\left( 1-\varphi\right)\hat{P}_{A} \right)}$ (8)

For the accumulation of pathogen X on host B, its relative fitness level is described by the parameter *l.* From equation (6), it then follows that we can write for this case:

$G\left( r \right)=\frac{l\left( 1-r \right)}{1-rl}$ (9)

In addition, the conspecific transmission rate is now given by:

$r=\varphi+\left( 1-\varphi\right)\left( {1-P}_{A} \right)$ (10)

Inserting equation (10) into equation (9) then yields:

${PX}_{B}=\frac{l\hat{P}_{A}\left( \varphi-1 \right)}{1-l\left( \varphi+\left( 1-\varphi\right)\left( 1-\hat{P}_{A} \right) \right)}$ (11)

For accumulation of pathogen Y on host B, the only difference is then that its relative fitness level is described by the parameter *n* (instead of *l*). Hence, it follows that we can modify equation (11) to obtain:

${PY}_{B}=\frac{n\hat{P}_{A}\left( \varphi-1 \right)}{1-n\left( \varphi+\left( 1-\varphi\right)\left( 1-\hat{P}_{A} \right) \right)}$ (12)

Where equations (7), (8), (11) and (12) correspond to the results reported in the main text.


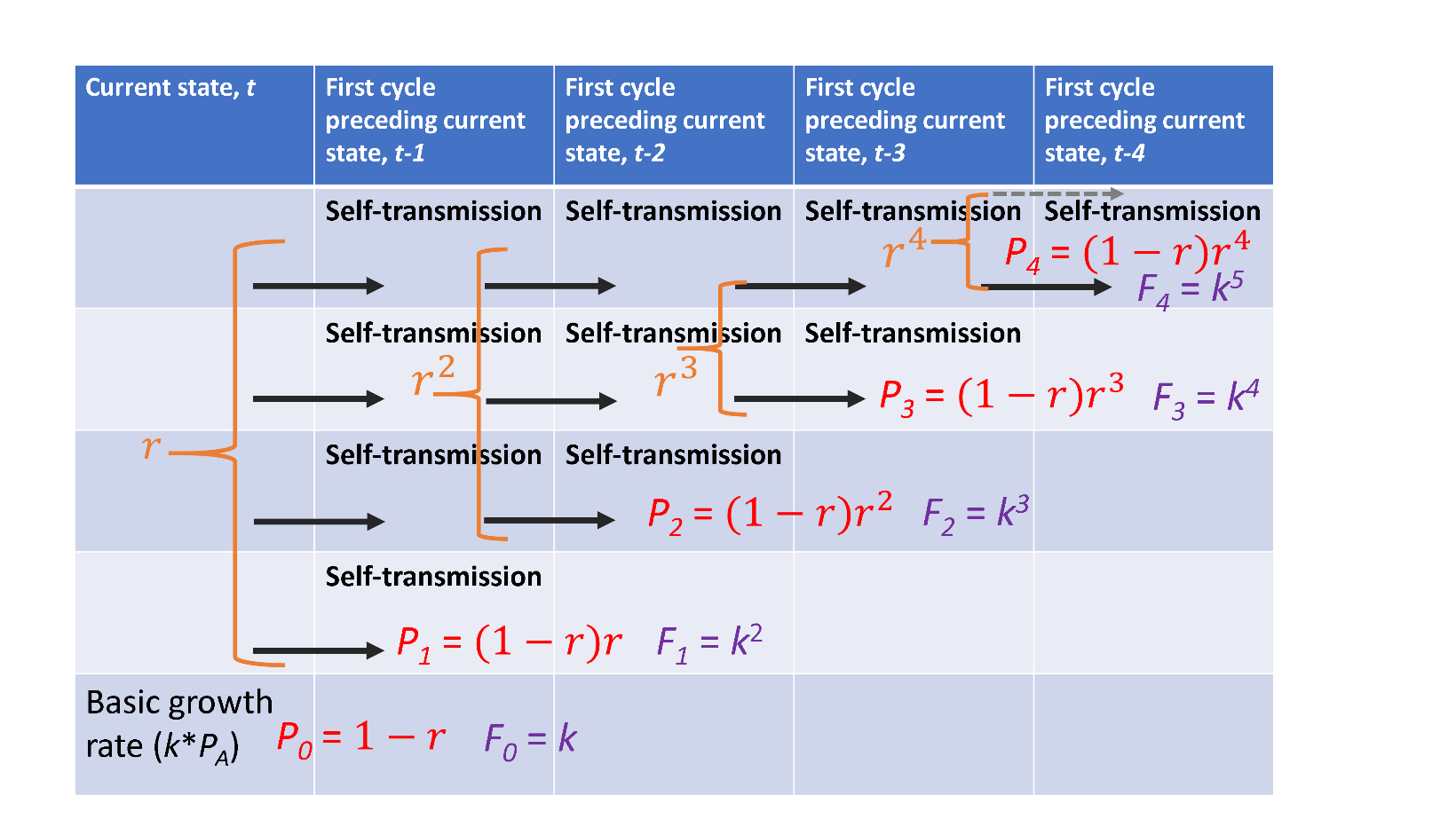


*Figure A1: Illustration of accumulated pathogen effects due to self-transmission within hosts. Given a transmission rate r, specific proportions of the populations will each contain an amount of pathogens based on historical transmission events. These proportions can be calculated using a backcasting approach, here illustrated for the four transmission cycles preceding the current system state.*

**Appendix 3**

In the main text section ‘*Derivation of parasite fitness values*’ we explain how observed abundances of pathogens and estimations of niche overlap between hosts could be utilized to calculate the fitness values of the pathogens. In this Appendix, we provide the analytical details of this process.

In the following, we focus on the case of two animal hosts, and two pathogens that are part of their microbiome. We also assume that empirical observations are made when the system has reached an equilibrium state. We will analyze the system described by the phenomenological feedback model (Bever and others 1997), but utilizing the pathogen effects as inferred from the model including non-random transmission (Bever 1999). We first recall that the equilibrium density of animal host A is then described by:

$\hat{P}_{A}=\frac{E_{A,B}}{I_{S}^{*}}=\frac{\alpha_{22}-\alpha_{12}+ \varphi\left( \alpha_{12}-\alpha_{11} \right)}{\left( 1- \varphi\right)\left( \alpha_{11}-\alpha_{12}-\alpha_{21}+\alpha_{12} \right)}$ (1)

As described in the main text, the phenomenological effects in equation (1) can be written as functions of pathogen and host effects:

$\alpha_{11}=\left( a-b \right)\left( k-m \right)$ (2a)

$\alpha_{12}=\left( a-b \right)\left( l-n \right)$ (2b)

$\alpha_{21}=\left( c-d \right)\left( k-m \right)$ (2c)

$\alpha_{22}=\left( c-d \right)\left( l-n \right)$ (2d)

Substituting equations (2) into equation (1), we obtain:

$\hat{P}_{A}=\frac{E_{A,B}}{I_{S}^{*}}=\frac{n-l+\left( m-k \right)\varphi}{\left( k-l-m+n \right)\left( 1-\varphi\right)}$ (3)

In Appendix 2, we derived the densities of the pathogens within both hosts. These expressions were functions of the equilibrium density of host A. Substituting equation (3) into these expressions now yields the following equilibrium pathogen densities:

$\hat{P}_{X,A}=\frac{k\left( k-m+\left( l-n \right)\varphi\right)}{k-l-m+n+k\left( l-n \right)\left( 1+\varphi\right)}$ (4a)

$\hat{P}_{Y,A}=\frac{m\left( k-m+\left( l-n \right)\varphi\right)}{k-l-m+n+m\left( l-n \right)\left( 1+\varphi\right)}$ (4b)

$\hat{P}_{X,B}=\frac{l\left( n-l+\left( m-k \right)\varphi\right)}{k-l-m+n+l\left( k-m \right)\left( 1+\varphi\right)}$ (4c)

$\hat{P}_{Y,B}=\frac{n\left( n-l+\left( m-k \right)\varphi\right)}{k-l-m+n+n\left( k-m \right)\left( 1+\varphi\right)}$ (4d)

The equilibrium pathogen densities described by equations (4), are then equivalent to the observed pathogen densities in both hosts. In other words, the equilibrium densities $\left( \hat{P}_{X,A},\hat{P}_{Y,A},\hat{P}_{X,B},P_{Y,B} \right)$ can be related to observed pathogen densities $\left( O_{X,A},O_{Y,A},O_{X,B},O_{Y,B} \right)$. This yields four equations $\left( \hat{P}_{X,A}=O_{X,A},\hat{P}_{Y,A}=O_{Y,A},P_{X,B}=O_{X,B},\hat{P}_{Y,B}=O_{Y,B} \right)$, from which we aim to solve four unknowns, namely the pathogen fitness parameters *k*, *l*, *m* and *n*. This procedure only leaves the parameter $\varphi$ unconstrained, which could be estimated from estimations of ungulate habitat overlap as described in the main text.

We approached this problem by sequentially solving these four equations. Specifically, when first solving for parameter *m*, we obtain an expression that only depends on the observables and on the parameter *k*:

$m=\frac{k\left( O_{X,A}-1 \right) O_{Y,A}}{-kO_{Y,A}+O_{X,A}\left( O_{Y,A}+k-1 \right)}$ (5)

Then solving for parameter *l* yields a similarly simple expression, although this expression not only depends on the observables and *k* but also on $\varphi$:

$l=\frac{k\left( O_{X,A}-1 \right) O_{X,B}}{O_{X,A}\left( 1-O_{X,B} \right)+k\left( \varphi\left( O_{X,A}-1 \right)\left( O_{X,B}-1 \right)+O_{X,A}O_{X,B}-1 \right)}$ (6)

Subsequently solving for *n* also yields an expression that depends on the observables, *k* and $\varphi$. The dependency on *k* in particular now becomes more complicated:

$n=\frac{\theta_{1}-\theta_{2}}{k\left( \varphi\left( O_{X,A}-1 \right)+O_{X,A} \right)-O_{X,A}}$ (7)

In which:

$\theta_{1}=\frac{k\left( k+k\varphi\left( O_{X,A}-1 \right)-O_{X,A} \right)\left( O_{X,A}-1 \right)}{O_{X,A}\left( 1-O_{X,B} \right)+k\left( \varphi\left( O_{X,A}-1 \right)\left( O_{X,B}-1 \right)+O_{X,A}O_{X,B}-1 \right)}$ (8)

and

$\theta_{2}=\frac{k\left( \left( k-1 \right)\left( k+1-2O_{X,A} \right)O_{X,A}+\left( k-O_{X,A} \right)^{2}O_{Y,A} \right)}{O_{X,A}\left( O_{Y,A}+k-1 \right)-kO_{Y,A}}$ (9)

The final step solves for parameter *k*, as a function of the observables and $\varphi$ only. Then, substituting this expression for *k* back into equations (5), (6) and (7), yields expressions for parameters *m*, *l* and *n* as a function of only observables and $\varphi$ as well. Given that the expressions in equations (4) contain products of the pathogen fitness parameters *m*, *l* and *n*, and that these parameters are higher order functions of the parameter *k*, solving this last equation is not straightforward. Specifically, we find four possible solutions for *k*, with expressions becoming too cumbersome to display here. These expressions are included in the scripts added as Electronic Supplementary Material. Importantly, these expressions allow for a near-instant calculation of each solution, when the observables $\left( O_{X,A},O_{Y,A},O_{X,B},O_{Y,B} \right)$ and $\varphi$ are provided as an input. We followed this procedure for each host pair-pathogen pair described in the main text.

**References cited in Appendix 1-3**

Bever JD. 1999. Dynamics within mutualism and the maintenance of diversity: inference from a model of interguild frequency dependence. Ecology Letters 2:52-61.

Bever JD, Westover KM, Antonovics J. 1997. Incorporating the soil community into plant population dynamics: the utility of the feedback approach. Journal of Ecology 85:561-573.

Eppinga MB, Baudena M, Johnson DJ, Jiang J, Mack KML, Strand AE, Bever JD. 2018. Frequency-dependent feedback constrains plant community coexistence. Nature Ecology & Evolution 2(9):1403-1407.

Eppinga MB, Rietkerk M, Dekker SC, De Ruiter PC, Van der Putten WH. 2006. Accumulation of local pathogens: a new hypothesis to explain exotic plant invasions. Oikos 114(1):168-176.

Mack KML, Eppinga MB, Bever JD. 2019. Plant-soil feedbacks promote coexistence and resilience in multi-species communities. Plos One 14(2).
