# Supplementary material for: Application of Microbiome Feedback Theory to Animals: Can Parasites Drive Coexistence in Ungulate Communities?
Source: Integr Comp Biol. 2025 Jun 10;65(2):445–60. doi: 10.1093/icb/icaf087 (PMC12448209; doi:10.1093/icb/icaf087)
Supplement: icaf087_Supplemental_Files [file icaf087_supplemental_files.zip › icb-2025-0058-File013.docx]

Appendix 4. Matlab code used to estimate parasitic nematode feedback

%% Read in data on helminth intensity and spatial overlap

% NOTE: before attempting, run long code for 'obtain_parameters2' function

% and preliminary values from Maarten Eppinga beginning on on line 225

ung_hel = readtable('Ezenwa ungulate helminth MT intensity cleaned 20250131.csv');

% note: keeps loads of 0 == 0 instead of converting to 0.001 because it

% yields more solutions that are real numbers AND not NaN

ung_hel_2 = ung_hel(:,2:end);

phis = readtable('overlap_metrics_JSA_20241230.csv');

% select just a small example (bison and pronghorn, cooperia and haemonchus)

ung_hel_2(2:3,[2,5])

%% parasite and host names

parasite_row_names = ung_hel{:,1};

host_col_names = ung_hel_2.Properties.VariableNames;

%% Compute all pairwise combinations of rows and columns

row_nums = 1:size(ung_hel_2, 1);

col_nums = 1:size(ung_hel_2, 2);

row_combos = nchoosek(row_nums, 2);

col_combos = nchoosek(col_nums, 2);

%% Initialize output table

num_combos = size(row_combos, 1) * size(col_combos, 1);

output = table('Size', [num_combos 37], ...

'VariableTypes',{'string', 'string', 'string', 'string', ...

'double', 'double', 'double', 'double', 'double', 'double', 'double', ...

'double', 'double', 'double', 'double', 'double', 'double', 'double', ...

'double', 'double', 'double', 'double', 'double', 'double', 'double', ...

'double', 'double', 'double', 'double', 'double', 'double', 'double', ...

'double', 'double', 'double', 'double', 'double'}, ...

'VariableNames', {'host_col_1_name', 'host_col_2_name', 'parasite_row_1_name', 'parasite_row_2_name', ...

'S1_K', 'S1_L', 'S1_M', 'S1_N', 'S1_Pa', 'S1_Is', 'S1_Paphi0', ...

'S2_K', 'S2_L', 'S2_M', 'S2_N', 'S2_Pa', 'S2_Is', 'S2_Paphi0', ...

'S3_K', 'S3_L', 'S3_M', 'S3_N', 'S3_Pa', 'S3_Is', 'S3_Paphi0', ...

'S4_K', 'S4_L', 'S4_M', 'S4_N', 'S4_Pa', 'S4_Is', 'S4_Paphi0', ...

'WW', 'XX', 'YY', 'ZZ', 'phi'});

%% walk through a single example

i = 14;

j = 8;

temp_test_table = ung_hel_2(row_combos(i, :), col_combos(j, :));

WW = temp_test_table{1,1};

XX = temp_test_table{2,1};

YY = temp_test_table{1,2};

ZZ = temp_test_table{2,2};

phi = phis.phi_2(find((strcmp(phis.sp_1, host_col_names{col_combos(j,1)}) & ...

strcmp(phis.sp_2, host_col_names{col_combos(j,2)})) | ...

(strcmp(phis.sp_2, host_col_names{col_combos(j,1)}) & ...

strcmp(phis.sp_1, host_col_names{col_combos(j,2)}))));

[temp_test_S1, temp_test_S2, temp_test_S3, temp_test_S4] = ObtainParameters2(...

WW, XX, YY, ZZ, phi);

output(1,1:2) = host_col_names(col_combos(j,1:2));

output(1,3:4) = parasite_row_names(row_combos(i,1:2))';

output(1,5:11) = num2cell(temp_test_S1);

output(1,12:18) = num2cell(temp_test_S2);

output(1,19:25) = num2cell(temp_test_S3);

output(1,26:32) = num2cell(temp_test_S4);

output(1,33) = num2cell(WW);

output(1,34) = num2cell(XX);

output(1,35) = num2cell(YY);

output(1,36) = num2cell(ZZ);

output(1,37) = num2cell(phi);

%% in the next sectios, there will be three loops to get solutions from all possible combos

% each will assume a different phi value 1) phi = as estiamted, 2) phi =

% 0.5*estimate, 3) phi = 0.25*estimate

% I'll combine these after the fact in R

%% loop to solve all possible combos, using phi = raw estimate

%Initialize output table

num_combos = size(row_combos, 1) * size(col_combos, 1);

output_raw_phi = table('Size', [num_combos 37], ...

'VariableTypes',{'string', 'string', 'string', 'string', ...

'double', 'double', 'double', 'double', 'double', 'double', 'double', ...

'double', 'double', 'double', 'double', 'double', 'double', 'double', ...

'double', 'double', 'double', 'double', 'double', 'double', 'double', ...

'double', 'double', 'double', 'double', 'double', 'double', 'double', ...

'double', 'double', 'double', 'double', 'double'}, ...

'VariableNames', {'host_col_1_name', 'host_col_2_name', 'parasite_row_1_name', 'parasite_row_2_name', ...

'S1_K', 'S1_L', 'S1_M', 'S1_N', 'S1_Pa', 'S1_Is', 'S1_Paphi0', ...

'S2_K', 'S2_L', 'S2_M', 'S2_N', 'S2_Pa', 'S2_Is', 'S2_Paphi0', ...

'S3_K', 'S3_L', 'S3_M', 'S3_N', 'S3_Pa', 'S3_Is', 'S3_Paphi0', ...

'S4_K', 'S4_L', 'S4_M', 'S4_N', 'S4_Pa', 'S4_Is', 'S4_Paphi0', ...

'WW', 'XX', 'YY', 'ZZ', 'phi'});

for i = 1:size(row_combos, 1)

for j = 1:size(col_combos, 1)

temp_test_table = ung_hel_2(row_combos(i, :), col_combos(j, :));

WW = temp_test_table{1,1};

XX = temp_test_table{2,1};

YY = temp_test_table{1,2};

ZZ = temp_test_table{2,2};

phi = phis.phi_2(find((strcmp(phis.sp_1, host_col_names{col_combos(j,1)}) & ...

strcmp(phis.sp_2, host_col_names{col_combos(j,2)})) | ...

(strcmp(phis.sp_2, host_col_names{col_combos(j,1)}) & ...

strcmp(phis.sp_1, host_col_names{col_combos(j,2)}))));

[temp_test_S1, temp_test_S2, temp_test_S3, temp_test_S4] = ObtainParameters2(...

WW, XX, YY, ZZ, phi);

output_raw_phi((i-1)*15+j,1:2) = host_col_names(col_combos(j,1:2));

output_raw_phi((i-1)*15+j,3:4) = parasite_row_names(row_combos(i,1:2))';

output_raw_phi((i-1)*15+j,5:11) = num2cell(temp_test_S1);

output_raw_phi((i-1)*15+j,12:18) = num2cell(temp_test_S2);

output_raw_phi((i-1)*15+j,19:25) = num2cell(temp_test_S3);

output_raw_phi((i-1)*15+j,26:32) = num2cell(temp_test_S4);

output_raw_phi((i-1)*15+j,33) = num2cell(WW);

output_raw_phi((i-1)*15+j,34) = num2cell(XX);

output_raw_phi((i-1)*15+j,35) = num2cell(YY);

output_raw_phi((i-1)*15+j,36) = num2cell(ZZ);

output_raw_phi((i-1)*15+j,37) = num2cell(phi);

end

end

% Write to csv

%writetable(output_raw_phi,'output raw phi 20250131.csv')

%% loop to solve all possible combos, using phi = 0.5*estimate

%Initialize output table

num_combos = size(row_combos, 1) * size(col_combos, 1);

output_half_phi = table('Size', [num_combos 37], ...

'VariableTypes',{'string', 'string', 'string', 'string', ...

'double', 'double', 'double', 'double', 'double', 'double', 'double', ...

'double', 'double', 'double', 'double', 'double', 'double', 'double', ...

'double', 'double', 'double', 'double', 'double', 'double', 'double', ...

'double', 'double', 'double', 'double', 'double', 'double', 'double', ...

'double', 'double', 'double', 'double', 'double'}, ...

'VariableNames', {'host_col_1_name', 'host_col_2_name', 'parasite_row_1_name', 'parasite_row_2_name', ...

'S1_K', 'S1_L', 'S1_M', 'S1_N', 'S1_Pa', 'S1_Is', 'S1_Paphi0', ...

'S2_K', 'S2_L', 'S2_M', 'S2_N', 'S2_Pa', 'S2_Is', 'S2_Paphi0', ...

'S3_K', 'S3_L', 'S3_M', 'S3_N', 'S3_Pa', 'S3_Is', 'S3_Paphi0', ...

'S4_K', 'S4_L', 'S4_M', 'S4_N', 'S4_Pa', 'S4_Is', 'S4_Paphi0', ...

'WW', 'XX', 'YY', 'ZZ', 'phi'});

for i = 1:size(row_combos, 1)

for j = 1:size(col_combos, 1)

temp_test_table = ung_hel_2(row_combos(i, :), col_combos(j, :));

WW = temp_test_table{1,1};

XX = temp_test_table{2,1};

YY = temp_test_table{1,2};

ZZ = temp_test_table{2,2};

phi = 0.5*phis.phi_2(find((strcmp(phis.sp_1, host_col_names{col_combos(j,1)}) & ...

strcmp(phis.sp_2, host_col_names{col_combos(j,2)})) | ...

(strcmp(phis.sp_2, host_col_names{col_combos(j,1)}) & ...

strcmp(phis.sp_1, host_col_names{col_combos(j,2)}))));

[temp_test_S1, temp_test_S2, temp_test_S3, temp_test_S4] = ObtainParameters2(...

WW, XX, YY, ZZ, phi);

output_half_phi((i-1)*15+j,1:2) = host_col_names(col_combos(j,1:2));

output_half_phi((i-1)*15+j,3:4) = parasite_row_names(row_combos(i,1:2))';

output_half_phi((i-1)*15+j,5:11) = num2cell(temp_test_S1);

output_half_phi((i-1)*15+j,12:18) = num2cell(temp_test_S2);

output_half_phi((i-1)*15+j,19:25) = num2cell(temp_test_S3);

output_half_phi((i-1)*15+j,26:32) = num2cell(temp_test_S4);

output_half_phi((i-1)*15+j,33) = num2cell(WW);

output_half_phi((i-1)*15+j,34) = num2cell(XX);

output_half_phi((i-1)*15+j,35) = num2cell(YY);

output_half_phi((i-1)*15+j,36) = num2cell(ZZ);

output_half_phi((i-1)*15+j,37) = num2cell(phi);

end

end

% Write to csv

%writetable(output_half_phi,'output half phi 20250131.csv')

%% loop to solve all possible combos, using phi = 0.25*estimate

%Initialize output table

num_combos = size(row_combos, 1) * size(col_combos, 1);

output_quarter_phi = table('Size', [num_combos 37], ...

'VariableTypes',{'string', 'string', 'string', 'string', ...

'double', 'double', 'double', 'double', 'double', 'double', 'double', ...

'double', 'double', 'double', 'double', 'double', 'double', 'double', ...

'double', 'double', 'double', 'double', 'double', 'double', 'double', ...

'double', 'double', 'double', 'double', 'double', 'double', 'double', ...

'double', 'double', 'double', 'double', 'double'}, ...

'VariableNames', {'host_col_1_name', 'host_col_2_name', 'parasite_row_1_name', 'parasite_row_2_name', ...

'S1_K', 'S1_L', 'S1_M', 'S1_N', 'S1_Pa', 'S1_Is', 'S1_Paphi0', ...

'S2_K', 'S2_L', 'S2_M', 'S2_N', 'S2_Pa', 'S2_Is', 'S2_Paphi0', ...

'S3_K', 'S3_L', 'S3_M', 'S3_N', 'S3_Pa', 'S3_Is', 'S3_Paphi0', ...

'S4_K', 'S4_L', 'S4_M', 'S4_N', 'S4_Pa', 'S4_Is', 'S4_Paphi0', ...

'WW', 'XX', 'YY', 'ZZ', 'phi'});

for i = 1:size(row_combos, 1)

for j = 1:size(col_combos, 1)

temp_test_table = ung_hel_2(row_combos(i, :), col_combos(j, :));

WW = temp_test_table{1,1};

XX = temp_test_table{2,1};

YY = temp_test_table{1,2};

ZZ = temp_test_table{2,2};

phi = 0.25*phis.phi_2(find((strcmp(phis.sp_1, host_col_names{col_combos(j,1)}) & ...

strcmp(phis.sp_2, host_col_names{col_combos(j,2)})) | ...

(strcmp(phis.sp_2, host_col_names{col_combos(j,1)}) & ...

strcmp(phis.sp_1, host_col_names{col_combos(j,2)}))));

[temp_test_S1, temp_test_S2, temp_test_S3, temp_test_S4] = ObtainParameters2(...

WW, XX, YY, ZZ, phi);

output_quarter_phi((i-1)*15+j,1:2) = host_col_names(col_combos(j,1:2));

output_quarter_phi((i-1)*15+j,3:4) = parasite_row_names(row_combos(i,1:2))';

output_quarter_phi((i-1)*15+j,5:11) = num2cell(temp_test_S1);

output_quarter_phi((i-1)*15+j,12:18) = num2cell(temp_test_S2);

output_quarter_phi((i-1)*15+j,19:25) = num2cell(temp_test_S3);

output_quarter_phi((i-1)*15+j,26:32) = num2cell(temp_test_S4);

output_quarter_phi((i-1)*15+j,33) = num2cell(WW);

output_quarter_phi((i-1)*15+j,34) = num2cell(XX);

output_quarter_phi((i-1)*15+j,35) = num2cell(YY);

output_quarter_phi((i-1)*15+j,36) = num2cell(ZZ);

output_quarter_phi((i-1)*15+j,37) = num2cell(phi);

end

end

% Write to csv

%writetable(output_quarter_phi,'output quarter phi 20250131.csv')

%% Maarten's solution code

%% Matlab Function to obtain 4 solutions of host-pathogen model based on observational data

function [S1, S2, S3, S4] = ObtainParameters2(WW,XX,YY,ZZ,phi)

ImagT = exp(-20); % Ignore very small complex parts < E-9

%% Solution 1

k1 = (-1/4).*((-1)+phi+(-1).*phi.*WW+(-1).*phi.*YY+WW.*YY+phi.*WW.*YY).^(-1) ...

.*((-1)+phi+(-1).*phi.*WW+(-1).*phi.*ZZ+WW.*ZZ+phi.*WW.*ZZ).^(-1).*((-1) ...

+2.*phi+(-1).*phi.^2+(-3).*WW+2.*phi.*WW+phi.^2.*WW+(-4).*phi.*WW.^2+ ...

phi.^2.*WW.^2+(-1).*phi.^2.*WW.^3+WW.*YY+(-4).*phi.*WW.*YY+2.*phi.^2.* ...

WW.*YY+3.*WW.^2.*YY+2.*phi.*WW.^2.*YY+(-4).*phi.^2.*WW.^2.*YY+2.*phi.* ...

WW.^3.*YY+2.*phi.^2.*WW.^3.*YY+(-2).*phi.*ZZ+2.*phi.^2.*ZZ+3.*WW.*ZZ+( ...

-2).*phi.*WW.*ZZ+(-4).*phi.^2.*WW.*ZZ+WW.^2.*ZZ+4.*phi.*WW.^2.*ZZ+2.* ...

phi.^2.*WW.^2.*ZZ+(-1).*phi.^2.*YY.*ZZ+4.*phi.*WW.*YY.*ZZ+phi.^2.*WW.* ...

YY.*ZZ+(-3).*WW.^2.*YY.*ZZ+(-2).*phi.*WW.^2.*YY.*ZZ+phi.^2.*WW.^2.*YY.* ...

ZZ+(-1).*WW.^3.*YY.*ZZ+(-2).*phi.*WW.^3.*YY.*ZZ+(-1).*phi.^2.*WW.^3.* ...

YY.*ZZ)+(-1/2).*((1/4).*((-1)+phi+(-1).*phi.*WW+(-1).*phi.*YY+WW.*YY+ ...

phi.*WW.*YY).^(-2).*((-1)+phi+(-1).*phi.*WW+(-1).*phi.*ZZ+WW.*ZZ+phi.* ...

WW.*ZZ).^(-2).*((-1)+2.*phi+(-1).*phi.^2+(-3).*WW+2.*phi.*WW+phi.^2.*WW+ ...

(-4).*phi.*WW.^2+phi.^2.*WW.^2+(-1).*phi.^2.*WW.^3+WW.*YY+(-4).*phi.* ...

WW.*YY+2.*phi.^2.*WW.*YY+3.*WW.^2.*YY+2.*phi.*WW.^2.*YY+(-4).*phi.^2.* ...

WW.^2.*YY+2.*phi.*WW.^3.*YY+2.*phi.^2.*WW.^3.*YY+(-2).*phi.*ZZ+2.* ...

phi.^2.*ZZ+3.*WW.*ZZ+(-2).*phi.*WW.*ZZ+(-4).*phi.^2.*WW.*ZZ+WW.^2.*ZZ+ ...

4.*phi.*WW.^2.*ZZ+2.*phi.^2.*WW.^2.*ZZ+(-1).*phi.^2.*YY.*ZZ+4.*phi.*WW.* ...

YY.*ZZ+phi.^2.*WW.*YY.*ZZ+(-3).*WW.^2.*YY.*ZZ+(-2).*phi.*WW.^2.*YY.*ZZ+ ...

phi.^2.*WW.^2.*YY.*ZZ+(-1).*WW.^3.*YY.*ZZ+(-2).*phi.*WW.^3.*YY.*ZZ+(-1) ...

.*phi.^2.*WW.^3.*YY.*ZZ).^2+(-1).*(WW+(-1).*XX).^(-1).*((-1)+phi+(-1).* ...

phi.*WW+(-1).*phi.*YY+WW.*YY+phi.*WW.*YY).^(-1).*((-1)+phi+(-1).*phi.* ...

WW+(-1).*phi.*ZZ+WW.*ZZ+phi.*WW.*ZZ).^(-1).*(3.*WW.^2+(-4).*phi.*WW.^2+ ...

phi.^2.*WW.^2+3.*WW.^3+2.*phi.*WW.^3+(-2).*phi.^2.*WW.^3+2.*phi.*WW.^4+ ...

phi.^2.*WW.^4+(-3).*WW.*XX+4.*phi.*WW.*XX+(-1).*phi.^2.*WW.*XX+(-3).* ...

WW.^2.*XX+(-2).*phi.*WW.^2.*XX+2.*phi.^2.*WW.^2.*XX+(-2).*phi.*WW.^3.* ...

XX+(-1).*phi.^2.*WW.^3.*XX+(-1).*phi.*WW.*YY+phi.^2.*WW.*YY+WW.^2.*YY+ ...

2.*phi.*WW.^2.*YY+(-4).*phi.^2.*WW.^2.*YY+(-5).*WW.^3.*YY+5.*phi.* ...

WW.^3.*YY+5.*phi.^2.*WW.^3.*YY+(-2).*WW.^4.*YY+(-6).*phi.*WW.^4.*YY+(-2) ...

.*phi.^2.*WW.^4.*YY+3.*WW.^2.*XX.*YY+(-5).*phi.*WW.^2.*XX.*YY+phi.^2.* ...

WW.^2.*XX.*YY+3.*WW.^3.*XX.*YY+4.*phi.*WW.^3.*XX.*YY+(-2).*phi.^2.* ...

WW.^3.*XX.*YY+phi.*WW.^4.*XX.*YY+phi.^2.*WW.^4.*XX.*YY+phi.*WW.*ZZ+(-1) ...

.*phi.^2.*WW.*ZZ+(-3).*WW.^2.*ZZ+4.*phi.*WW.^2.*ZZ+2.*phi.^2.*WW.^2.*ZZ+ ...

(-3).*WW.^3.*ZZ+(-5).*phi.*WW.^3.*ZZ+(-1).*phi.^2.*WW.^3.*ZZ+2.*WW.*XX.* ...

ZZ+(-6).*phi.*WW.*XX.*ZZ+2.*phi.^2.*WW.*XX.*ZZ+5.*WW.^2.*XX.*ZZ+5.*phi.* ...

WW.^2.*XX.*ZZ+(-5).*phi.^2.*WW.^2.*XX.*ZZ+(-1).*WW.^3.*XX.*ZZ+2.*phi.* ...

WW.^3.*XX.*ZZ+4.*phi.^2.*WW.^3.*XX.*ZZ+(-1).*phi.*WW.^4.*XX.*ZZ+(-1).* ...

phi.^2.*WW.^4.*XX.*ZZ+(-2).*phi.*WW.^2.*YY.*ZZ+phi.^2.*WW.^2.*YY.*ZZ+3.* ...

WW.^3.*YY.*ZZ+(-2).*phi.*WW.^3.*YY.*ZZ+(-2).*phi.^2.*WW.^3.*YY.*ZZ+3.* ...

WW.^4.*YY.*ZZ+4.*phi.*WW.^4.*YY.*ZZ+phi.^2.*WW.^4.*YY.*ZZ+2.*phi.*WW.* ...

XX.*YY.*ZZ+(-1).*phi.^2.*WW.*XX.*YY.*ZZ+(-3).*WW.^2.*XX.*YY.*ZZ+2.*phi.* ...

WW.^2.*XX.*YY.*ZZ+2.*phi.^2.*WW.^2.*XX.*YY.*ZZ+(-3).*WW.^3.*XX.*YY.*ZZ+( ...

-4).*phi.*WW.^3.*XX.*YY.*ZZ+(-1).*phi.^2.*WW.^3.*XX.*YY.*ZZ)+(1/3).*(WW+ ...

(-2).*phi.*WW+phi.^2.*WW+2.*phi.*WW.^2+(-2).*phi.^2.*WW.^2+phi.^2.* ...

WW.^3+(-1).*XX+2.*phi.*XX+(-1).*phi.^2.*XX+(-2).*phi.*WW.*XX+2.*phi.^2.* ...

WW.*XX+(-1).*phi.^2.*WW.^2.*XX+phi.*WW.*YY+(-1).*phi.^2.*WW.*YY+(-1).* ...

WW.^2.*YY+2.*phi.^2.*WW.^2.*YY+(-1).*phi.*WW.^3.*YY+(-1).*phi.^2.* ...

WW.^3.*YY+(-1).*phi.*XX.*YY+phi.^2.*XX.*YY+WW.*XX.*YY+(-2).*phi.^2.*WW.* ...

XX.*YY+phi.*WW.^2.*XX.*YY+phi.^2.*WW.^2.*XX.*YY+phi.*WW.*ZZ+(-1).* ...

phi.^2.*WW.*ZZ+(-1).*WW.^2.*ZZ+2.*phi.^2.*WW.^2.*ZZ+(-1).*phi.*WW.^3.* ...

ZZ+(-1).*phi.^2.*WW.^3.*ZZ+(-1).*phi.*XX.*ZZ+phi.^2.*XX.*ZZ+WW.*XX.*ZZ+( ...

-2).*phi.^2.*WW.*XX.*ZZ+phi.*WW.^2.*XX.*ZZ+phi.^2.*WW.^2.*XX.*ZZ+ ...

phi.^2.*WW.*YY.*ZZ+(-2).*phi.*WW.^2.*YY.*ZZ+(-2).*phi.^2.*WW.^2.*YY.*ZZ+ ...

WW.^3.*YY.*ZZ+2.*phi.*WW.^3.*YY.*ZZ+phi.^2.*WW.^3.*YY.*ZZ+(-1).*phi.^2.* ...

XX.*YY.*ZZ+2.*phi.*WW.*XX.*YY.*ZZ+2.*phi.^2.*WW.*XX.*YY.*ZZ+(-1).* ...

WW.^2.*XX.*YY.*ZZ+(-2).*phi.*WW.^2.*XX.*YY.*ZZ+(-1).*phi.^2.*WW.^2.*XX.* ...

YY.*ZZ).^(-1).*(3.*WW.^2+(-4).*phi.*WW.^2+phi.^2.*WW.^2+3.*WW.^3+2.* ...

phi.*WW.^3+(-2).*phi.^2.*WW.^3+2.*phi.*WW.^4+phi.^2.*WW.^4+(-3).*WW.*XX+ ...

4.*phi.*WW.*XX+(-1).*phi.^2.*WW.*XX+(-3).*WW.^2.*XX+(-2).*phi.*WW.^2.* ...

XX+2.*phi.^2.*WW.^2.*XX+(-2).*phi.*WW.^3.*XX+(-1).*phi.^2.*WW.^3.*XX+( ...

-1).*phi.*WW.*YY+phi.^2.*WW.*YY+WW.^2.*YY+2.*phi.*WW.^2.*YY+(-4).* ...

phi.^2.*WW.^2.*YY+(-5).*WW.^3.*YY+5.*phi.*WW.^3.*YY+5.*phi.^2.*WW.^3.* ...

YY+(-2).*WW.^4.*YY+(-6).*phi.*WW.^4.*YY+(-2).*phi.^2.*WW.^4.*YY+3.* ...

WW.^2.*XX.*YY+(-5).*phi.*WW.^2.*XX.*YY+phi.^2.*WW.^2.*XX.*YY+3.*WW.^3.* ...

XX.*YY+4.*phi.*WW.^3.*XX.*YY+(-2).*phi.^2.*WW.^3.*XX.*YY+phi.*WW.^4.* ...

XX.*YY+phi.^2.*WW.^4.*XX.*YY+phi.*WW.*ZZ+(-1).*phi.^2.*WW.*ZZ+(-3).* ...

WW.^2.*ZZ+4.*phi.*WW.^2.*ZZ+2.*phi.^2.*WW.^2.*ZZ+(-3).*WW.^3.*ZZ+(-5).* ...

phi.*WW.^3.*ZZ+(-1).*phi.^2.*WW.^3.*ZZ+2.*WW.*XX.*ZZ+(-6).*phi.*WW.*XX.* ...

ZZ+2.*phi.^2.*WW.*XX.*ZZ+5.*WW.^2.*XX.*ZZ+5.*phi.*WW.^2.*XX.*ZZ+(-5).* ...

phi.^2.*WW.^2.*XX.*ZZ+(-1).*WW.^3.*XX.*ZZ+2.*phi.*WW.^3.*XX.*ZZ+4.* ...

phi.^2.*WW.^3.*XX.*ZZ+(-1).*phi.*WW.^4.*XX.*ZZ+(-1).*phi.^2.*WW.^4.*XX.* ...

ZZ+(-2).*phi.*WW.^2.*YY.*ZZ+phi.^2.*WW.^2.*YY.*ZZ+3.*WW.^3.*YY.*ZZ+(-2) ...

.*phi.*WW.^3.*YY.*ZZ+(-2).*phi.^2.*WW.^3.*YY.*ZZ+3.*WW.^4.*YY.*ZZ+4.* ...

phi.*WW.^4.*YY.*ZZ+phi.^2.*WW.^4.*YY.*ZZ+2.*phi.*WW.*XX.*YY.*ZZ+(-1).* ...

phi.^2.*WW.*XX.*YY.*ZZ+(-3).*WW.^2.*XX.*YY.*ZZ+2.*phi.*WW.^2.*XX.*YY.* ...

ZZ+2.*phi.^2.*WW.^2.*XX.*YY.*ZZ+(-3).*WW.^3.*XX.*YY.*ZZ+(-4).*phi.* ...

WW.^3.*XX.*YY.*ZZ+(-1).*phi.^2.*WW.^3.*XX.*YY.*ZZ)+(1/3).*2.^(1/3).*(WW+ ...

(-1).*XX).^(-1).*((-1)+phi+(-1).*phi.*WW+(-1).*phi.*YY+WW.*YY+phi.*WW.* ...

YY).^(-1).*((-1)+phi+(-1).*phi.*WW+(-1).*phi.*ZZ+WW.*ZZ+phi.*WW.*ZZ).^( ...

-1).*(12.*(WW+(-2).*phi.*WW+phi.^2.*WW+2.*phi.*WW.^2+(-2).*phi.^2.* ...

WW.^2+phi.^2.*WW.^3+(-1).*XX+2.*phi.*XX+(-1).*phi.^2.*XX+(-2).*phi.*WW.* ...

XX+2.*phi.^2.*WW.*XX+(-1).*phi.^2.*WW.^2.*XX+phi.*WW.*YY+(-1).*phi.^2.* ...

WW.*YY+(-1).*WW.^2.*YY+2.*phi.^2.*WW.^2.*YY+(-1).*phi.*WW.^3.*YY+(-1).* ...

phi.^2.*WW.^3.*YY+(-1).*phi.*XX.*YY+phi.^2.*XX.*YY+WW.*XX.*YY+(-2).* ...

phi.^2.*WW.*XX.*YY+phi.*WW.^2.*XX.*YY+phi.^2.*WW.^2.*XX.*YY+phi.*WW.*ZZ+ ...

(-1).*phi.^2.*WW.*ZZ+(-1).*WW.^2.*ZZ+2.*phi.^2.*WW.^2.*ZZ+(-1).*phi.* ...

WW.^3.*ZZ+(-1).*phi.^2.*WW.^3.*ZZ+(-1).*phi.*XX.*ZZ+phi.^2.*XX.*ZZ+WW.* ...

XX.*ZZ+(-2).*phi.^2.*WW.*XX.*ZZ+phi.*WW.^2.*XX.*ZZ+phi.^2.*WW.^2.*XX.* ...

ZZ+phi.^2.*WW.*YY.*ZZ+(-2).*phi.*WW.^2.*YY.*ZZ+(-2).*phi.^2.*WW.^2.*YY.* ...

ZZ+WW.^3.*YY.*ZZ+2.*phi.*WW.^3.*YY.*ZZ+phi.^2.*WW.^3.*YY.*ZZ+(-1).* ...

phi.^2.*XX.*YY.*ZZ+2.*phi.*WW.*XX.*YY.*ZZ+2.*phi.^2.*WW.*XX.*YY.*ZZ+(-1) ...

.*WW.^2.*XX.*YY.*ZZ+(-2).*phi.*WW.^2.*XX.*YY.*ZZ+(-1).*phi.^2.*WW.^2.* ...

XX.*YY.*ZZ).*(WW.^4+(-1).*WW.^3.*XX+WW.^3.*YY+(-2).*WW.^4.*YY+WW.^4.* ...

XX.*YY+(-1).*WW.^3.*ZZ+2.*WW.^3.*XX.*ZZ+(-1).*WW.^4.*XX.*ZZ+WW.^4.*YY.* ...

ZZ+(-1).*WW.^3.*XX.*YY.*ZZ)+(3.*WW.^2+(-4).*phi.*WW.^2+phi.^2.*WW.^2+3.* ...

WW.^3+2.*phi.*WW.^3+(-2).*phi.^2.*WW.^3+2.*phi.*WW.^4+phi.^2.*WW.^4+(-3) ...

.*WW.*XX+4.*phi.*WW.*XX+(-1).*phi.^2.*WW.*XX+(-3).*WW.^2.*XX+(-2).*phi.* ...

WW.^2.*XX+2.*phi.^2.*WW.^2.*XX+(-2).*phi.*WW.^3.*XX+(-1).*phi.^2.* ...

WW.^3.*XX+(-1).*phi.*WW.*YY+phi.^2.*WW.*YY+WW.^2.*YY+2.*phi.*WW.^2.*YY+( ...

-4).*phi.^2.*WW.^2.*YY+(-5).*WW.^3.*YY+5.*phi.*WW.^3.*YY+5.*phi.^2.* ...

WW.^3.*YY+(-2).*WW.^4.*YY+(-6).*phi.*WW.^4.*YY+(-2).*phi.^2.*WW.^4.*YY+ ...

3.*WW.^2.*XX.*YY+(-5).*phi.*WW.^2.*XX.*YY+phi.^2.*WW.^2.*XX.*YY+3.* ...

WW.^3.*XX.*YY+4.*phi.*WW.^3.*XX.*YY+(-2).*phi.^2.*WW.^3.*XX.*YY+phi.* ...

WW.^4.*XX.*YY+phi.^2.*WW.^4.*XX.*YY+phi.*WW.*ZZ+(-1).*phi.^2.*WW.*ZZ+( ...

-3).*WW.^2.*ZZ+4.*phi.*WW.^2.*ZZ+2.*phi.^2.*WW.^2.*ZZ+(-3).*WW.^3.*ZZ+( ...

-5).*phi.*WW.^3.*ZZ+(-1).*phi.^2.*WW.^3.*ZZ+2.*WW.*XX.*ZZ+(-6).*phi.* ...

WW.*XX.*ZZ+2.*phi.^2.*WW.*XX.*ZZ+5.*WW.^2.*XX.*ZZ+5.*phi.*WW.^2.*XX.*ZZ+ ...

(-5).*phi.^2.*WW.^2.*XX.*ZZ+(-1).*WW.^3.*XX.*ZZ+2.*phi.*WW.^3.*XX.*ZZ+ ...

4.*phi.^2.*WW.^3.*XX.*ZZ+(-1).*phi.*WW.^4.*XX.*ZZ+(-1).*phi.^2.*WW.^4.* ...

XX.*ZZ+(-2).*phi.*WW.^2.*YY.*ZZ+phi.^2.*WW.^2.*YY.*ZZ+3.*WW.^3.*YY.*ZZ+( ...

-2).*phi.*WW.^3.*YY.*ZZ+(-2).*phi.^2.*WW.^3.*YY.*ZZ+3.*WW.^4.*YY.*ZZ+4.* ...

phi.*WW.^4.*YY.*ZZ+phi.^2.*WW.^4.*YY.*ZZ+2.*phi.*WW.*XX.*YY.*ZZ+(-1).* ...

phi.^2.*WW.*XX.*YY.*ZZ+(-3).*WW.^2.*XX.*YY.*ZZ+2.*phi.*WW.^2.*XX.*YY.* ...

ZZ+2.*phi.^2.*WW.^2.*XX.*YY.*ZZ+(-3).*WW.^3.*XX.*YY.*ZZ+(-4).*phi.* ...

WW.^3.*XX.*YY.*ZZ+(-1).*phi.^2.*WW.^3.*XX.*YY.*ZZ).^2+(-3).*((-3).* ...

WW.^3+2.*phi.*WW.^3+(-1).*WW.^4+(-2).*phi.*WW.^4+3.*WW.^2.*XX+(-2).* ...

phi.*WW.^2.*XX+WW.^3.*XX+2.*phi.*WW.^3.*XX+(-1).*WW.^2.*YY+2.*phi.* ...

WW.^2.*YY+WW.^3.*YY+(-6).*phi.*WW.^3.*YY+4.*WW.^4.*YY+4.*phi.*WW.^4.*YY+ ...

(-3).*WW.^3.*XX.*YY+2.*phi.*WW.^3.*XX.*YY+(-1).*WW.^4.*XX.*YY+(-2).* ...

phi.*WW.^4.*XX.*YY+WW.^2.*ZZ+(-2).*phi.*WW.^2.*ZZ+3.*WW.^3.*ZZ+2.*phi.* ...

WW.^3.*ZZ+(-4).*WW.^2.*XX.*ZZ+4.*phi.*WW.^2.*XX.*ZZ+(-1).*WW.^3.*XX.*ZZ+ ...

(-6).*phi.*WW.^3.*XX.*ZZ+WW.^4.*XX.*ZZ+2.*phi.*WW.^4.*XX.*ZZ+(-1).* ...

WW.^3.*YY.*ZZ+2.*phi.*WW.^3.*YY.*ZZ+(-3).*WW.^4.*YY.*ZZ+(-2).*phi.* ...

WW.^4.*YY.*ZZ+WW.^2.*XX.*YY.*ZZ+(-2).*phi.*WW.^2.*XX.*YY.*ZZ+3.*WW.^3.* ...

XX.*YY.*ZZ+2.*phi.*WW.^3.*XX.*YY.*ZZ).*((-1).*WW+2.*phi.*WW+(-1).* ...

phi.^2.*WW+(-3).*WW.^2+2.*phi.*WW.^2+phi.^2.*WW.^2+(-4).*phi.*WW.^3+ ...

phi.^2.*WW.^3+(-1).*phi.^2.*WW.^4+XX+(-2).*phi.*XX+phi.^2.*XX+3.*WW.*XX+ ...

(-2).*phi.*WW.*XX+(-1).*phi.^2.*WW.*XX+4.*phi.*WW.^2.*XX+(-1).*phi.^2.* ...

WW.^2.*XX+phi.^2.*WW.^3.*XX+WW.^2.*YY+(-4).*phi.*WW.^2.*YY+2.*phi.^2.* ...

WW.^2.*YY+3.*WW.^3.*YY+2.*phi.*WW.^3.*YY+(-4).*phi.^2.*WW.^3.*YY+2.* ...

phi.*WW.^4.*YY+2.*phi.^2.*WW.^4.*YY+(-1).*WW.*XX.*YY+4.*phi.*WW.*XX.*YY+ ...

(-2).*phi.^2.*WW.*XX.*YY+(-3).*WW.^2.*XX.*YY+(-2).*phi.*WW.^2.*XX.*YY+ ...

4.*phi.^2.*WW.^2.*XX.*YY+(-2).*phi.*WW.^3.*XX.*YY+(-2).*phi.^2.*WW.^3.* ...

XX.*YY+(-2).*phi.*WW.*ZZ+2.*phi.^2.*WW.*ZZ+3.*WW.^2.*ZZ+(-2).*phi.* ...

WW.^2.*ZZ+(-4).*phi.^2.*WW.^2.*ZZ+WW.^3.*ZZ+4.*phi.*WW.^3.*ZZ+2.* ...

phi.^2.*WW.^3.*ZZ+2.*phi.*XX.*ZZ+(-2).*phi.^2.*XX.*ZZ+(-3).*WW.*XX.*ZZ+ ...

2.*phi.*WW.*XX.*ZZ+4.*phi.^2.*WW.*XX.*ZZ+(-1).*WW.^2.*XX.*ZZ+(-4).*phi.* ...

WW.^2.*XX.*ZZ+(-2).*phi.^2.*WW.^2.*XX.*ZZ+(-1).*phi.^2.*WW.*YY.*ZZ+4.* ...

phi.*WW.^2.*YY.*ZZ+phi.^2.*WW.^2.*YY.*ZZ+(-3).*WW.^3.*YY.*ZZ+(-2).*phi.* ...

WW.^3.*YY.*ZZ+phi.^2.*WW.^3.*YY.*ZZ+(-1).*WW.^4.*YY.*ZZ+(-2).*phi.* ...

WW.^4.*YY.*ZZ+(-1).*phi.^2.*WW.^4.*YY.*ZZ+phi.^2.*XX.*YY.*ZZ+(-4).*phi.* ...

WW.*XX.*YY.*ZZ+(-1).*phi.^2.*WW.*XX.*YY.*ZZ+3.*WW.^2.*XX.*YY.*ZZ+2.* ...

phi.*WW.^2.*XX.*YY.*ZZ+(-1).*phi.^2.*WW.^2.*XX.*YY.*ZZ+WW.^3.*XX.*YY.* ...

ZZ+2.*phi.*WW.^3.*XX.*YY.*ZZ+phi.^2.*WW.^3.*XX.*YY.*ZZ)).*(27.*(WW+(-2) ...

.*phi.*WW+phi.^2.*WW+2.*phi.*WW.^2+(-2).*phi.^2.*WW.^2+phi.^2.*WW.^3+( ...

-1).*XX+2.*phi.*XX+(-1).*phi.^2.*XX+(-2).*phi.*WW.*XX+2.*phi.^2.*WW.*XX+ ...

(-1).*phi.^2.*WW.^2.*XX+phi.*WW.*YY+(-1).*phi.^2.*WW.*YY+(-1).*WW.^2.* ...

YY+2.*phi.^2.*WW.^2.*YY+(-1).*phi.*WW.^3.*YY+(-1).*phi.^2.*WW.^3.*YY+( ...

-1).*phi.*XX.*YY+phi.^2.*XX.*YY+WW.*XX.*YY+(-2).*phi.^2.*WW.*XX.*YY+ ...

phi.*WW.^2.*XX.*YY+phi.^2.*WW.^2.*XX.*YY+phi.*WW.*ZZ+(-1).*phi.^2.*WW.* ...

ZZ+(-1).*WW.^2.*ZZ+2.*phi.^2.*WW.^2.*ZZ+(-1).*phi.*WW.^3.*ZZ+(-1).* ...

phi.^2.*WW.^3.*ZZ+(-1).*phi.*XX.*ZZ+phi.^2.*XX.*ZZ+WW.*XX.*ZZ+(-2).* ...

phi.^2.*WW.*XX.*ZZ+phi.*WW.^2.*XX.*ZZ+phi.^2.*WW.^2.*XX.*ZZ+phi.^2.*WW.* ...

YY.*ZZ+(-2).*phi.*WW.^2.*YY.*ZZ+(-2).*phi.^2.*WW.^2.*YY.*ZZ+WW.^3.*YY.* ...

ZZ+2.*phi.*WW.^3.*YY.*ZZ+phi.^2.*WW.^3.*YY.*ZZ+(-1).*phi.^2.*XX.*YY.*ZZ+ ...

2.*phi.*WW.*XX.*YY.*ZZ+2.*phi.^2.*WW.*XX.*YY.*ZZ+(-1).*WW.^2.*XX.*YY.* ...

ZZ+(-2).*phi.*WW.^2.*XX.*YY.*ZZ+(-1).*phi.^2.*WW.^2.*XX.*YY.*ZZ).*((-3) ...

.*WW.^3+2.*phi.*WW.^3+(-1).*WW.^4+(-2).*phi.*WW.^4+3.*WW.^2.*XX+(-2).* ...

phi.*WW.^2.*XX+WW.^3.*XX+2.*phi.*WW.^3.*XX+(-1).*WW.^2.*YY+2.*phi.* ...

WW.^2.*YY+WW.^3.*YY+(-6).*phi.*WW.^3.*YY+4.*WW.^4.*YY+4.*phi.*WW.^4.*YY+ ...

(-3).*WW.^3.*XX.*YY+2.*phi.*WW.^3.*XX.*YY+(-1).*WW.^4.*XX.*YY+(-2).* ...

phi.*WW.^4.*XX.*YY+WW.^2.*ZZ+(-2).*phi.*WW.^2.*ZZ+3.*WW.^3.*ZZ+2.*phi.* ...

WW.^3.*ZZ+(-4).*WW.^2.*XX.*ZZ+4.*phi.*WW.^2.*XX.*ZZ+(-1).*WW.^3.*XX.*ZZ+ ...

(-6).*phi.*WW.^3.*XX.*ZZ+WW.^4.*XX.*ZZ+2.*phi.*WW.^4.*XX.*ZZ+(-1).* ...

WW.^3.*YY.*ZZ+2.*phi.*WW.^3.*YY.*ZZ+(-3).*WW.^4.*YY.*ZZ+(-2).*phi.* ...

WW.^4.*YY.*ZZ+WW.^2.*XX.*YY.*ZZ+(-2).*phi.*WW.^2.*XX.*YY.*ZZ+3.*WW.^3.* ...

XX.*YY.*ZZ+2.*phi.*WW.^3.*XX.*YY.*ZZ).^2+(-72).*(WW+(-2).*phi.*WW+ ...

phi.^2.*WW+2.*phi.*WW.^2+(-2).*phi.^2.*WW.^2+phi.^2.*WW.^3+(-1).*XX+2.* ...

phi.*XX+(-1).*phi.^2.*XX+(-2).*phi.*WW.*XX+2.*phi.^2.*WW.*XX+(-1).* ...

phi.^2.*WW.^2.*XX+phi.*WW.*YY+(-1).*phi.^2.*WW.*YY+(-1).*WW.^2.*YY+2.* ...

phi.^2.*WW.^2.*YY+(-1).*phi.*WW.^3.*YY+(-1).*phi.^2.*WW.^3.*YY+(-1).* ...

phi.*XX.*YY+phi.^2.*XX.*YY+WW.*XX.*YY+(-2).*phi.^2.*WW.*XX.*YY+phi.* ...

WW.^2.*XX.*YY+phi.^2.*WW.^2.*XX.*YY+phi.*WW.*ZZ+(-1).*phi.^2.*WW.*ZZ+( ...

-1).*WW.^2.*ZZ+2.*phi.^2.*WW.^2.*ZZ+(-1).*phi.*WW.^3.*ZZ+(-1).*phi.^2.* ...

WW.^3.*ZZ+(-1).*phi.*XX.*ZZ+phi.^2.*XX.*ZZ+WW.*XX.*ZZ+(-2).*phi.^2.*WW.* ...

XX.*ZZ+phi.*WW.^2.*XX.*ZZ+phi.^2.*WW.^2.*XX.*ZZ+phi.^2.*WW.*YY.*ZZ+(-2) ...

.*phi.*WW.^2.*YY.*ZZ+(-2).*phi.^2.*WW.^2.*YY.*ZZ+WW.^3.*YY.*ZZ+2.*phi.* ...

WW.^3.*YY.*ZZ+phi.^2.*WW.^3.*YY.*ZZ+(-1).*phi.^2.*XX.*YY.*ZZ+2.*phi.* ...

WW.*XX.*YY.*ZZ+2.*phi.^2.*WW.*XX.*YY.*ZZ+(-1).*WW.^2.*XX.*YY.*ZZ+(-2).* ...

phi.*WW.^2.*XX.*YY.*ZZ+(-1).*phi.^2.*WW.^2.*XX.*YY.*ZZ).*(WW.^4+(-1).* ...

WW.^3.*XX+WW.^3.*YY+(-2).*WW.^4.*YY+WW.^4.*XX.*YY+(-1).*WW.^3.*ZZ+2.* ...

WW.^3.*XX.*ZZ+(-1).*WW.^4.*XX.*ZZ+WW.^4.*YY.*ZZ+(-1).*WW.^3.*XX.*YY.*ZZ) ...

.*(3.*WW.^2+(-4).*phi.*WW.^2+phi.^2.*WW.^2+3.*WW.^3+2.*phi.*WW.^3+(-2).* ...

phi.^2.*WW.^3+2.*phi.*WW.^4+phi.^2.*WW.^4+(-3).*WW.*XX+4.*phi.*WW.*XX+( ...

-1).*phi.^2.*WW.*XX+(-3).*WW.^2.*XX+(-2).*phi.*WW.^2.*XX+2.*phi.^2.* ...

WW.^2.*XX+(-2).*phi.*WW.^3.*XX+(-1).*phi.^2.*WW.^3.*XX+(-1).*phi.*WW.* ...

YY+phi.^2.*WW.*YY+WW.^2.*YY+2.*phi.*WW.^2.*YY+(-4).*phi.^2.*WW.^2.*YY+( ...

-5).*WW.^3.*YY+5.*phi.*WW.^3.*YY+5.*phi.^2.*WW.^3.*YY+(-2).*WW.^4.*YY+( ...

-6).*phi.*WW.^4.*YY+(-2).*phi.^2.*WW.^4.*YY+3.*WW.^2.*XX.*YY+(-5).*phi.* ...

WW.^2.*XX.*YY+phi.^2.*WW.^2.*XX.*YY+3.*WW.^3.*XX.*YY+4.*phi.*WW.^3.*XX.* ...

YY+(-2).*phi.^2.*WW.^3.*XX.*YY+phi.*WW.^4.*XX.*YY+phi.^2.*WW.^4.*XX.*YY+ ...

phi.*WW.*ZZ+(-1).*phi.^2.*WW.*ZZ+(-3).*WW.^2.*ZZ+4.*phi.*WW.^2.*ZZ+2.* ...

phi.^2.*WW.^2.*ZZ+(-3).*WW.^3.*ZZ+(-5).*phi.*WW.^3.*ZZ+(-1).*phi.^2.* ...

WW.^3.*ZZ+2.*WW.*XX.*ZZ+(-6).*phi.*WW.*XX.*ZZ+2.*phi.^2.*WW.*XX.*ZZ+5.* ...

WW.^2.*XX.*ZZ+5.*phi.*WW.^2.*XX.*ZZ+(-5).*phi.^2.*WW.^2.*XX.*ZZ+(-1).* ...

WW.^3.*XX.*ZZ+2.*phi.*WW.^3.*XX.*ZZ+4.*phi.^2.*WW.^3.*XX.*ZZ+(-1).*phi.* ...

WW.^4.*XX.*ZZ+(-1).*phi.^2.*WW.^4.*XX.*ZZ+(-2).*phi.*WW.^2.*YY.*ZZ+ ...

phi.^2.*WW.^2.*YY.*ZZ+3.*WW.^3.*YY.*ZZ+(-2).*phi.*WW.^3.*YY.*ZZ+(-2).* ...

phi.^2.*WW.^3.*YY.*ZZ+3.*WW.^4.*YY.*ZZ+4.*phi.*WW.^4.*YY.*ZZ+phi.^2.* ...

WW.^4.*YY.*ZZ+2.*phi.*WW.*XX.*YY.*ZZ+(-1).*phi.^2.*WW.*XX.*YY.*ZZ+(-3).* ...

WW.^2.*XX.*YY.*ZZ+2.*phi.*WW.^2.*XX.*YY.*ZZ+2.*phi.^2.*WW.^2.*XX.*YY.* ...

ZZ+(-3).*WW.^3.*XX.*YY.*ZZ+(-4).*phi.*WW.^3.*XX.*YY.*ZZ+(-1).*phi.^2.* ...

WW.^3.*XX.*YY.*ZZ)+2.*(3.*WW.^2+(-4).*phi.*WW.^2+phi.^2.*WW.^2+3.*WW.^3+ ...

2.*phi.*WW.^3+(-2).*phi.^2.*WW.^3+2.*phi.*WW.^4+phi.^2.*WW.^4+(-3).*WW.* ...

XX+4.*phi.*WW.*XX+(-1).*phi.^2.*WW.*XX+(-3).*WW.^2.*XX+(-2).*phi.* ...

WW.^2.*XX+2.*phi.^2.*WW.^2.*XX+(-2).*phi.*WW.^3.*XX+(-1).*phi.^2.* ...

WW.^3.*XX+(-1).*phi.*WW.*YY+phi.^2.*WW.*YY+WW.^2.*YY+2.*phi.*WW.^2.*YY+( ...

-4).*phi.^2.*WW.^2.*YY+(-5).*WW.^3.*YY+5.*phi.*WW.^3.*YY+5.*phi.^2.* ...

WW.^3.*YY+(-2).*WW.^4.*YY+(-6).*phi.*WW.^4.*YY+(-2).*phi.^2.*WW.^4.*YY+ ...

3.*WW.^2.*XX.*YY+(-5).*phi.*WW.^2.*XX.*YY+phi.^2.*WW.^2.*XX.*YY+3.* ...

WW.^3.*XX.*YY+4.*phi.*WW.^3.*XX.*YY+(-2).*phi.^2.*WW.^3.*XX.*YY+phi.* ...

WW.^4.*XX.*YY+phi.^2.*WW.^4.*XX.*YY+phi.*WW.*ZZ+(-1).*phi.^2.*WW.*ZZ+( ...

-3).*WW.^2.*ZZ+4.*phi.*WW.^2.*ZZ+2.*phi.^2.*WW.^2.*ZZ+(-3).*WW.^3.*ZZ+( ...

-5).*phi.*WW.^3.*ZZ+(-1).*phi.^2.*WW.^3.*ZZ+2.*WW.*XX.*ZZ+(-6).*phi.* ...

WW.*XX.*ZZ+2.*phi.^2.*WW.*XX.*ZZ+5.*WW.^2.*XX.*ZZ+5.*phi.*WW.^2.*XX.*ZZ+ ...

(-5).*phi.^2.*WW.^2.*XX.*ZZ+(-1).*WW.^3.*XX.*ZZ+2.*phi.*WW.^3.*XX.*ZZ+ ...

4.*phi.^2.*WW.^3.*XX.*ZZ+(-1).*phi.*WW.^4.*XX.*ZZ+(-1).*phi.^2.*WW.^4.* ...

XX.*ZZ+(-2).*phi.*WW.^2.*YY.*ZZ+phi.^2.*WW.^2.*YY.*ZZ+3.*WW.^3.*YY.*ZZ+( ...

-2).*phi.*WW.^3.*YY.*ZZ+(-2).*phi.^2.*WW.^3.*YY.*ZZ+3.*WW.^4.*YY.*ZZ+4.* ...

phi.*WW.^4.*YY.*ZZ+phi.^2.*WW.^4.*YY.*ZZ+2.*phi.*WW.*XX.*YY.*ZZ+(-1).* ...

phi.^2.*WW.*XX.*YY.*ZZ+(-3).*WW.^2.*XX.*YY.*ZZ+2.*phi.*WW.^2.*XX.*YY.* ...

ZZ+2.*phi.^2.*WW.^2.*XX.*YY.*ZZ+(-3).*WW.^3.*XX.*YY.*ZZ+(-4).*phi.* ...

WW.^3.*XX.*YY.*ZZ+(-1).*phi.^2.*WW.^3.*XX.*YY.*ZZ).^3+(-9).*((-3).* ...

WW.^3+2.*phi.*WW.^3+(-1).*WW.^4+(-2).*phi.*WW.^4+3.*WW.^2.*XX+(-2).* ...

phi.*WW.^2.*XX+WW.^3.*XX+2.*phi.*WW.^3.*XX+(-1).*WW.^2.*YY+2.*phi.* ...

WW.^2.*YY+WW.^3.*YY+(-6).*phi.*WW.^3.*YY+4.*WW.^4.*YY+4.*phi.*WW.^4.*YY+ ...

(-3).*WW.^3.*XX.*YY+2.*phi.*WW.^3.*XX.*YY+(-1).*WW.^4.*XX.*YY+(-2).* ...

phi.*WW.^4.*XX.*YY+WW.^2.*ZZ+(-2).*phi.*WW.^2.*ZZ+3.*WW.^3.*ZZ+2.*phi.* ...

WW.^3.*ZZ+(-4).*WW.^2.*XX.*ZZ+4.*phi.*WW.^2.*XX.*ZZ+(-1).*WW.^3.*XX.*ZZ+ ...

(-6).*phi.*WW.^3.*XX.*ZZ+WW.^4.*XX.*ZZ+2.*phi.*WW.^4.*XX.*ZZ+(-1).* ...

WW.^3.*YY.*ZZ+2.*phi.*WW.^3.*YY.*ZZ+(-3).*WW.^4.*YY.*ZZ+(-2).*phi.* ...

WW.^4.*YY.*ZZ+WW.^2.*XX.*YY.*ZZ+(-2).*phi.*WW.^2.*XX.*YY.*ZZ+3.*WW.^3.* ...

XX.*YY.*ZZ+2.*phi.*WW.^3.*XX.*YY.*ZZ).*(3.*WW.^2+(-4).*phi.*WW.^2+ ...

phi.^2.*WW.^2+3.*WW.^3+2.*phi.*WW.^3+(-2).*phi.^2.*WW.^3+2.*phi.*WW.^4+ ...

phi.^2.*WW.^4+(-3).*WW.*XX+4.*phi.*WW.*XX+(-1).*phi.^2.*WW.*XX+(-3).* ...

WW.^2.*XX+(-2).*phi.*WW.^2.*XX+2.*phi.^2.*WW.^2.*XX+(-2).*phi.*WW.^3.* ...

XX+(-1).*phi.^2.*WW.^3.*XX+(-1).*phi.*WW.*YY+phi.^2.*WW.*YY+WW.^2.*YY+ ...

2.*phi.*WW.^2.*YY+(-4).*phi.^2.*WW.^2.*YY+(-5).*WW.^3.*YY+5.*phi.* ...

WW.^3.*YY+5.*phi.^2.*WW.^3.*YY+(-2).*WW.^4.*YY+(-6).*phi.*WW.^4.*YY+(-2) ...

.*phi.^2.*WW.^4.*YY+3.*WW.^2.*XX.*YY+(-5).*phi.*WW.^2.*XX.*YY+phi.^2.* ...

WW.^2.*XX.*YY+3.*WW.^3.*XX.*YY+4.*phi.*WW.^3.*XX.*YY+(-2).*phi.^2.* ...

WW.^3.*XX.*YY+phi.*WW.^4.*XX.*YY+phi.^2.*WW.^4.*XX.*YY+phi.*WW.*ZZ+(-1) ...

.*phi.^2.*WW.*ZZ+(-3).*WW.^2.*ZZ+4.*phi.*WW.^2.*ZZ+2.*phi.^2.*WW.^2.*ZZ+ ...

(-3).*WW.^3.*ZZ+(-5).*phi.*WW.^3.*ZZ+(-1).*phi.^2.*WW.^3.*ZZ+2.*WW.*XX.* ...

ZZ+(-6).*phi.*WW.*XX.*ZZ+2.*phi.^2.*WW.*XX.*ZZ+5.*WW.^2.*XX.*ZZ+5.*phi.* ...

WW.^2.*XX.*ZZ+(-5).*phi.^2.*WW.^2.*XX.*ZZ+(-1).*WW.^3.*XX.*ZZ+2.*phi.* ...

WW.^3.*XX.*ZZ+4.*phi.^2.*WW.^3.*XX.*ZZ+(-1).*phi.*WW.^4.*XX.*ZZ+(-1).* ...

phi.^2.*WW.^4.*XX.*ZZ+(-2).*phi.*WW.^2.*YY.*ZZ+phi.^2.*WW.^2.*YY.*ZZ+3.* ...

WW.^3.*YY.*ZZ+(-2).*phi.*WW.^3.*YY.*ZZ+(-2).*phi.^2.*WW.^3.*YY.*ZZ+3.* ...

WW.^4.*YY.*ZZ+4.*phi.*WW.^4.*YY.*ZZ+phi.^2.*WW.^4.*YY.*ZZ+2.*phi.*WW.* ...

XX.*YY.*ZZ+(-1).*phi.^2.*WW.*XX.*YY.*ZZ+(-3).*WW.^2.*XX.*YY.*ZZ+2.*phi.* ...

WW.^2.*XX.*YY.*ZZ+2.*phi.^2.*WW.^2.*XX.*YY.*ZZ+(-3).*WW.^3.*XX.*YY.*ZZ+( ...

-4).*phi.*WW.^3.*XX.*YY.*ZZ+(-1).*phi.^2.*WW.^3.*XX.*YY.*ZZ).*((-1).*WW+ ...

2.*phi.*WW+(-1).*phi.^2.*WW+(-3).*WW.^2+2.*phi.*WW.^2+phi.^2.*WW.^2+(-4) ...

.*phi.*WW.^3+phi.^2.*WW.^3+(-1).*phi.^2.*WW.^4+XX+(-2).*phi.*XX+phi.^2.* ...

XX+3.*WW.*XX+(-2).*phi.*WW.*XX+(-1).*phi.^2.*WW.*XX+4.*phi.*WW.^2.*XX+( ...

-1).*phi.^2.*WW.^2.*XX+phi.^2.*WW.^3.*XX+WW.^2.*YY+(-4).*phi.*WW.^2.*YY+ ...

2.*phi.^2.*WW.^2.*YY+3.*WW.^3.*YY+2.*phi.*WW.^3.*YY+(-4).*phi.^2.* ...

WW.^3.*YY+2.*phi.*WW.^4.*YY+2.*phi.^2.*WW.^4.*YY+(-1).*WW.*XX.*YY+4.* ...

phi.*WW.*XX.*YY+(-2).*phi.^2.*WW.*XX.*YY+(-3).*WW.^2.*XX.*YY+(-2).*phi.* ...

WW.^2.*XX.*YY+4.*phi.^2.*WW.^2.*XX.*YY+(-2).*phi.*WW.^3.*XX.*YY+(-2).* ...

phi.^2.*WW.^3.*XX.*YY+(-2).*phi.*WW.*ZZ+2.*phi.^2.*WW.*ZZ+3.*WW.^2.*ZZ+( ...

-2).*phi.*WW.^2.*ZZ+(-4).*phi.^2.*WW.^2.*ZZ+WW.^3.*ZZ+4.*phi.*WW.^3.*ZZ+ ...

2.*phi.^2.*WW.^3.*ZZ+2.*phi.*XX.*ZZ+(-2).*phi.^2.*XX.*ZZ+(-3).*WW.*XX.* ...

ZZ+2.*phi.*WW.*XX.*ZZ+4.*phi.^2.*WW.*XX.*ZZ+(-1).*WW.^2.*XX.*ZZ+(-4).* ...

phi.*WW.^2.*XX.*ZZ+(-2).*phi.^2.*WW.^2.*XX.*ZZ+(-1).*phi.^2.*WW.*YY.*ZZ+ ...

4.*phi.*WW.^2.*YY.*ZZ+phi.^2.*WW.^2.*YY.*ZZ+(-3).*WW.^3.*YY.*ZZ+(-2).* ...

phi.*WW.^3.*YY.*ZZ+phi.^2.*WW.^3.*YY.*ZZ+(-1).*WW.^4.*YY.*ZZ+(-2).*phi.* ...

WW.^4.*YY.*ZZ+(-1).*phi.^2.*WW.^4.*YY.*ZZ+phi.^2.*XX.*YY.*ZZ+(-4).*phi.* ...

WW.*XX.*YY.*ZZ+(-1).*phi.^2.*WW.*XX.*YY.*ZZ+3.*WW.^2.*XX.*YY.*ZZ+2.* ...

phi.*WW.^2.*XX.*YY.*ZZ+(-1).*phi.^2.*WW.^2.*XX.*YY.*ZZ+WW.^3.*XX.*YY.* ...

ZZ+2.*phi.*WW.^3.*XX.*YY.*ZZ+phi.^2.*WW.^3.*XX.*YY.*ZZ)+27.*(WW.^4+(-1) ...

.*WW.^3.*XX+WW.^3.*YY+(-2).*WW.^4.*YY+WW.^4.*XX.*YY+(-1).*WW.^3.*ZZ+2.* ...

WW.^3.*XX.*ZZ+(-1).*WW.^4.*XX.*ZZ+WW.^4.*YY.*ZZ+(-1).*WW.^3.*XX.*YY.*ZZ) ...

.*((-1).*WW+2.*phi.*WW+(-1).*phi.^2.*WW+(-3).*WW.^2+2.*phi.*WW.^2+ ...

phi.^2.*WW.^2+(-4).*phi.*WW.^3+phi.^2.*WW.^3+(-1).*phi.^2.*WW.^4+XX+(-2) ...

.*phi.*XX+phi.^2.*XX+3.*WW.*XX+(-2).*phi.*WW.*XX+(-1).*phi.^2.*WW.*XX+ ...

4.*phi.*WW.^2.*XX+(-1).*phi.^2.*WW.^2.*XX+phi.^2.*WW.^3.*XX+WW.^2.*YY+( ...

-4).*phi.*WW.^2.*YY+2.*phi.^2.*WW.^2.*YY+3.*WW.^3.*YY+2.*phi.*WW.^3.*YY+ ...

(-4).*phi.^2.*WW.^3.*YY+2.*phi.*WW.^4.*YY+2.*phi.^2.*WW.^4.*YY+(-1).* ...

WW.*XX.*YY+4.*phi.*WW.*XX.*YY+(-2).*phi.^2.*WW.*XX.*YY+(-3).*WW.^2.*XX.* ...

YY+(-2).*phi.*WW.^2.*XX.*YY+4.*phi.^2.*WW.^2.*XX.*YY+(-2).*phi.*WW.^3.* ...

XX.*YY+(-2).*phi.^2.*WW.^3.*XX.*YY+(-2).*phi.*WW.*ZZ+2.*phi.^2.*WW.*ZZ+ ...

3.*WW.^2.*ZZ+(-2).*phi.*WW.^2.*ZZ+(-4).*phi.^2.*WW.^2.*ZZ+WW.^3.*ZZ+4.* ...

phi.*WW.^3.*ZZ+2.*phi.^2.*WW.^3.*ZZ+2.*phi.*XX.*ZZ+(-2).*phi.^2.*XX.*ZZ+ ...

(-3).*WW.*XX.*ZZ+2.*phi.*WW.*XX.*ZZ+4.*phi.^2.*WW.*XX.*ZZ+(-1).*WW.^2.* ...

XX.*ZZ+(-4).*phi.*WW.^2.*XX.*ZZ+(-2).*phi.^2.*WW.^2.*XX.*ZZ+(-1).* ...

phi.^2.*WW.*YY.*ZZ+4.*phi.*WW.^2.*YY.*ZZ+phi.^2.*WW.^2.*YY.*ZZ+(-3).* ...

WW.^3.*YY.*ZZ+(-2).*phi.*WW.^3.*YY.*ZZ+phi.^2.*WW.^3.*YY.*ZZ+(-1).* ...

WW.^4.*YY.*ZZ+(-2).*phi.*WW.^4.*YY.*ZZ+(-1).*phi.^2.*WW.^4.*YY.*ZZ+ ...

phi.^2.*XX.*YY.*ZZ+(-4).*phi.*WW.*XX.*YY.*ZZ+(-1).*phi.^2.*WW.*XX.*YY.* ...

ZZ+3.*WW.^2.*XX.*YY.*ZZ+2.*phi.*WW.^2.*XX.*YY.*ZZ+(-1).*phi.^2.*WW.^2.* ...

XX.*YY.*ZZ+WW.^3.*XX.*YY.*ZZ+2.*phi.*WW.^3.*XX.*YY.*ZZ+phi.^2.*WW.^3.* ...

XX.*YY.*ZZ).^2+((-4).*(12.*(WW+(-2).*phi.*WW+phi.^2.*WW+2.*phi.*WW.^2+( ...

-2).*phi.^2.*WW.^2+phi.^2.*WW.^3+(-1).*XX+2.*phi.*XX+(-1).*phi.^2.*XX+( ...

-2).*phi.*WW.*XX+2.*phi.^2.*WW.*XX+(-1).*phi.^2.*WW.^2.*XX+phi.*WW.*YY+( ...

-1).*phi.^2.*WW.*YY+(-1).*WW.^2.*YY+2.*phi.^2.*WW.^2.*YY+(-1).*phi.* ...

WW.^3.*YY+(-1).*phi.^2.*WW.^3.*YY+(-1).*phi.*XX.*YY+phi.^2.*XX.*YY+WW.* ...

XX.*YY+(-2).*phi.^2.*WW.*XX.*YY+phi.*WW.^2.*XX.*YY+phi.^2.*WW.^2.*XX.* ...

YY+phi.*WW.*ZZ+(-1).*phi.^2.*WW.*ZZ+(-1).*WW.^2.*ZZ+2.*phi.^2.*WW.^2.* ...

ZZ+(-1).*phi.*WW.^3.*ZZ+(-1).*phi.^2.*WW.^3.*ZZ+(-1).*phi.*XX.*ZZ+ ...

phi.^2.*XX.*ZZ+WW.*XX.*ZZ+(-2).*phi.^2.*WW.*XX.*ZZ+phi.*WW.^2.*XX.*ZZ+ ...

phi.^2.*WW.^2.*XX.*ZZ+phi.^2.*WW.*YY.*ZZ+(-2).*phi.*WW.^2.*YY.*ZZ+(-2).* ...

phi.^2.*WW.^2.*YY.*ZZ+WW.^3.*YY.*ZZ+2.*phi.*WW.^3.*YY.*ZZ+phi.^2.* ...

WW.^3.*YY.*ZZ+(-1).*phi.^2.*XX.*YY.*ZZ+2.*phi.*WW.*XX.*YY.*ZZ+2.* ...

phi.^2.*WW.*XX.*YY.*ZZ+(-1).*WW.^2.*XX.*YY.*ZZ+(-2).*phi.*WW.^2.*XX.* ...

YY.*ZZ+(-1).*phi.^2.*WW.^2.*XX.*YY.*ZZ).*(WW.^4+(-1).*WW.^3.*XX+WW.^3.* ...

YY+(-2).*WW.^4.*YY+WW.^4.*XX.*YY+(-1).*WW.^3.*ZZ+2.*WW.^3.*XX.*ZZ+(-1).* ...

WW.^4.*XX.*ZZ+WW.^4.*YY.*ZZ+(-1).*WW.^3.*XX.*YY.*ZZ)+(3.*WW.^2+(-4).* ...

phi.*WW.^2+phi.^2.*WW.^2+3.*WW.^3+2.*phi.*WW.^3+(-2).*phi.^2.*WW.^3+2.* ...

phi.*WW.^4+phi.^2.*WW.^4+(-3).*WW.*XX+4.*phi.*WW.*XX+(-1).*phi.^2.*WW.* ...

XX+(-3).*WW.^2.*XX+(-2).*phi.*WW.^2.*XX+2.*phi.^2.*WW.^2.*XX+(-2).*phi.* ...

WW.^3.*XX+(-1).*phi.^2.*WW.^3.*XX+(-1).*phi.*WW.*YY+phi.^2.*WW.*YY+ ...

WW.^2.*YY+2.*phi.*WW.^2.*YY+(-4).*phi.^2.*WW.^2.*YY+(-5).*WW.^3.*YY+5.* ...

phi.*WW.^3.*YY+5.*phi.^2.*WW.^3.*YY+(-2).*WW.^4.*YY+(-6).*phi.*WW.^4.* ...

YY+(-2).*phi.^2.*WW.^4.*YY+3.*WW.^2.*XX.*YY+(-5).*phi.*WW.^2.*XX.*YY+ ...

phi.^2.*WW.^2.*XX.*YY+3.*WW.^3.*XX.*YY+4.*phi.*WW.^3.*XX.*YY+(-2).* ...

phi.^2.*WW.^3.*XX.*YY+phi.*WW.^4.*XX.*YY+phi.^2.*WW.^4.*XX.*YY+phi.*WW.* ...

ZZ+(-1).*phi.^2.*WW.*ZZ+(-3).*WW.^2.*ZZ+4.*phi.*WW.^2.*ZZ+2.*phi.^2.* ...

WW.^2.*ZZ+(-3).*WW.^3.*ZZ+(-5).*phi.*WW.^3.*ZZ+(-1).*phi.^2.*WW.^3.*ZZ+ ...

2.*WW.*XX.*ZZ+(-6).*phi.*WW.*XX.*ZZ+2.*phi.^2.*WW.*XX.*ZZ+5.*WW.^2.*XX.* ...

ZZ+5.*phi.*WW.^2.*XX.*ZZ+(-5).*phi.^2.*WW.^2.*XX.*ZZ+(-1).*WW.^3.*XX.* ...

ZZ+2.*phi.*WW.^3.*XX.*ZZ+4.*phi.^2.*WW.^3.*XX.*ZZ+(-1).*phi.*WW.^4.*XX.* ...

ZZ+(-1).*phi.^2.*WW.^4.*XX.*ZZ+(-2).*phi.*WW.^2.*YY.*ZZ+phi.^2.*WW.^2.* ...

YY.*ZZ+3.*WW.^3.*YY.*ZZ+(-2).*phi.*WW.^3.*YY.*ZZ+(-2).*phi.^2.*WW.^3.* ...

YY.*ZZ+3.*WW.^4.*YY.*ZZ+4.*phi.*WW.^4.*YY.*ZZ+phi.^2.*WW.^4.*YY.*ZZ+2.* ...

phi.*WW.*XX.*YY.*ZZ+(-1).*phi.^2.*WW.*XX.*YY.*ZZ+(-3).*WW.^2.*XX.*YY.* ...

ZZ+2.*phi.*WW.^2.*XX.*YY.*ZZ+2.*phi.^2.*WW.^2.*XX.*YY.*ZZ+(-3).*WW.^3.* ...

XX.*YY.*ZZ+(-4).*phi.*WW.^3.*XX.*YY.*ZZ+(-1).*phi.^2.*WW.^3.*XX.*YY.*ZZ) ...

.^2+(-3).*((-3).*WW.^3+2.*phi.*WW.^3+(-1).*WW.^4+(-2).*phi.*WW.^4+3.* ...

WW.^2.*XX+(-2).*phi.*WW.^2.*XX+WW.^3.*XX+2.*phi.*WW.^3.*XX+(-1).*WW.^2.* ...

YY+2.*phi.*WW.^2.*YY+WW.^3.*YY+(-6).*phi.*WW.^3.*YY+4.*WW.^4.*YY+4.* ...

phi.*WW.^4.*YY+(-3).*WW.^3.*XX.*YY+2.*phi.*WW.^3.*XX.*YY+(-1).*WW.^4.* ...

XX.*YY+(-2).*phi.*WW.^4.*XX.*YY+WW.^2.*ZZ+(-2).*phi.*WW.^2.*ZZ+3.* ...

WW.^3.*ZZ+2.*phi.*WW.^3.*ZZ+(-4).*WW.^2.*XX.*ZZ+4.*phi.*WW.^2.*XX.*ZZ+( ...

-1).*WW.^3.*XX.*ZZ+(-6).*phi.*WW.^3.*XX.*ZZ+WW.^4.*XX.*ZZ+2.*phi.* ...

WW.^4.*XX.*ZZ+(-1).*WW.^3.*YY.*ZZ+2.*phi.*WW.^3.*YY.*ZZ+(-3).*WW.^4.* ...

YY.*ZZ+(-2).*phi.*WW.^4.*YY.*ZZ+WW.^2.*XX.*YY.*ZZ+(-2).*phi.*WW.^2.*XX.* ...

YY.*ZZ+3.*WW.^3.*XX.*YY.*ZZ+2.*phi.*WW.^3.*XX.*YY.*ZZ).*((-1).*WW+2.* ...

phi.*WW+(-1).*phi.^2.*WW+(-3).*WW.^2+2.*phi.*WW.^2+phi.^2.*WW.^2+(-4).* ...

phi.*WW.^3+phi.^2.*WW.^3+(-1).*phi.^2.*WW.^4+XX+(-2).*phi.*XX+phi.^2.* ...

XX+3.*WW.*XX+(-2).*phi.*WW.*XX+(-1).*phi.^2.*WW.*XX+4.*phi.*WW.^2.*XX+( ...

-1).*phi.^2.*WW.^2.*XX+phi.^2.*WW.^3.*XX+WW.^2.*YY+(-4).*phi.*WW.^2.*YY+ ...

2.*phi.^2.*WW.^2.*YY+3.*WW.^3.*YY+2.*phi.*WW.^3.*YY+(-4).*phi.^2.* ...

WW.^3.*YY+2.*phi.*WW.^4.*YY+2.*phi.^2.*WW.^4.*YY+(-1).*WW.*XX.*YY+4.* ...

phi.*WW.*XX.*YY+(-2).*phi.^2.*WW.*XX.*YY+(-3).*WW.^2.*XX.*YY+(-2).*phi.* ...

WW.^2.*XX.*YY+4.*phi.^2.*WW.^2.*XX.*YY+(-2).*phi.*WW.^3.*XX.*YY+(-2).* ...

phi.^2.*WW.^3.*XX.*YY+(-2).*phi.*WW.*ZZ+2.*phi.^2.*WW.*ZZ+3.*WW.^2.*ZZ+( ...

-2).*phi.*WW.^2.*ZZ+(-4).*phi.^2.*WW.^2.*ZZ+WW.^3.*ZZ+4.*phi.*WW.^3.*ZZ+ ...

2.*phi.^2.*WW.^3.*ZZ+2.*phi.*XX.*ZZ+(-2).*phi.^2.*XX.*ZZ+(-3).*WW.*XX.* ...

ZZ+2.*phi.*WW.*XX.*ZZ+4.*phi.^2.*WW.*XX.*ZZ+(-1).*WW.^2.*XX.*ZZ+(-4).* ...

phi.*WW.^2.*XX.*ZZ+(-2).*phi.^2.*WW.^2.*XX.*ZZ+(-1).*phi.^2.*WW.*YY.*ZZ+ ...

4.*phi.*WW.^2.*YY.*ZZ+phi.^2.*WW.^2.*YY.*ZZ+(-3).*WW.^3.*YY.*ZZ+(-2).* ...

phi.*WW.^3.*YY.*ZZ+phi.^2.*WW.^3.*YY.*ZZ+(-1).*WW.^4.*YY.*ZZ+(-2).*phi.* ...

WW.^4.*YY.*ZZ+(-1).*phi.^2.*WW.^4.*YY.*ZZ+phi.^2.*XX.*YY.*ZZ+(-4).*phi.* ...

WW.*XX.*YY.*ZZ+(-1).*phi.^2.*WW.*XX.*YY.*ZZ+3.*WW.^2.*XX.*YY.*ZZ+2.* ...

phi.*WW.^2.*XX.*YY.*ZZ+(-1).*phi.^2.*WW.^2.*XX.*YY.*ZZ+WW.^3.*XX.*YY.* ...

ZZ+2.*phi.*WW.^3.*XX.*YY.*ZZ+phi.^2.*WW.^3.*XX.*YY.*ZZ)).^3+(27.*(WW+( ...

-2).*phi.*WW+phi.^2.*WW+2.*phi.*WW.^2+(-2).*phi.^2.*WW.^2+phi.^2.*WW.^3+ ...

(-1).*XX+2.*phi.*XX+(-1).*phi.^2.*XX+(-2).*phi.*WW.*XX+2.*phi.^2.*WW.* ...

XX+(-1).*phi.^2.*WW.^2.*XX+phi.*WW.*YY+(-1).*phi.^2.*WW.*YY+(-1).* ...

WW.^2.*YY+2.*phi.^2.*WW.^2.*YY+(-1).*phi.*WW.^3.*YY+(-1).*phi.^2.* ...

WW.^3.*YY+(-1).*phi.*XX.*YY+phi.^2.*XX.*YY+WW.*XX.*YY+(-2).*phi.^2.*WW.* ...

XX.*YY+phi.*WW.^2.*XX.*YY+phi.^2.*WW.^2.*XX.*YY+phi.*WW.*ZZ+(-1).* ...

phi.^2.*WW.*ZZ+(-1).*WW.^2.*ZZ+2.*phi.^2.*WW.^2.*ZZ+(-1).*phi.*WW.^3.* ...

ZZ+(-1).*phi.^2.*WW.^3.*ZZ+(-1).*phi.*XX.*ZZ+phi.^2.*XX.*ZZ+WW.*XX.*ZZ+( ...

-2).*phi.^2.*WW.*XX.*ZZ+phi.*WW.^2.*XX.*ZZ+phi.^2.*WW.^2.*XX.*ZZ+ ...

phi.^2.*WW.*YY.*ZZ+(-2).*phi.*WW.^2.*YY.*ZZ+(-2).*phi.^2.*WW.^2.*YY.*ZZ+ ...

WW.^3.*YY.*ZZ+2.*phi.*WW.^3.*YY.*ZZ+phi.^2.*WW.^3.*YY.*ZZ+(-1).*phi.^2.* ...

XX.*YY.*ZZ+2.*phi.*WW.*XX.*YY.*ZZ+2.*phi.^2.*WW.*XX.*YY.*ZZ+(-1).* ...

WW.^2.*XX.*YY.*ZZ+(-2).*phi.*WW.^2.*XX.*YY.*ZZ+(-1).*phi.^2.*WW.^2.*XX.* ...

YY.*ZZ).*((-3).*WW.^3+2.*phi.*WW.^3+(-1).*WW.^4+(-2).*phi.*WW.^4+3.* ...

WW.^2.*XX+(-2).*phi.*WW.^2.*XX+WW.^3.*XX+2.*phi.*WW.^3.*XX+(-1).*WW.^2.* ...

YY+2.*phi.*WW.^2.*YY+WW.^3.*YY+(-6).*phi.*WW.^3.*YY+4.*WW.^4.*YY+4.* ...

phi.*WW.^4.*YY+(-3).*WW.^3.*XX.*YY+2.*phi.*WW.^3.*XX.*YY+(-1).*WW.^4.* ...

XX.*YY+(-2).*phi.*WW.^4.*XX.*YY+WW.^2.*ZZ+(-2).*phi.*WW.^2.*ZZ+3.* ...

WW.^3.*ZZ+2.*phi.*WW.^3.*ZZ+(-4).*WW.^2.*XX.*ZZ+4.*phi.*WW.^2.*XX.*ZZ+( ...

-1).*WW.^3.*XX.*ZZ+(-6).*phi.*WW.^3.*XX.*ZZ+WW.^4.*XX.*ZZ+2.*phi.* ...

WW.^4.*XX.*ZZ+(-1).*WW.^3.*YY.*ZZ+2.*phi.*WW.^3.*YY.*ZZ+(-3).*WW.^4.* ...

YY.*ZZ+(-2).*phi.*WW.^4.*YY.*ZZ+WW.^2.*XX.*YY.*ZZ+(-2).*phi.*WW.^2.*XX.* ...

YY.*ZZ+3.*WW.^3.*XX.*YY.*ZZ+2.*phi.*WW.^3.*XX.*YY.*ZZ).^2+(-72).*(WW+( ...

-2).*phi.*WW+phi.^2.*WW+2.*phi.*WW.^2+(-2).*phi.^2.*WW.^2+phi.^2.*WW.^3+ ...

(-1).*XX+2.*phi.*XX+(-1).*phi.^2.*XX+(-2).*phi.*WW.*XX+2.*phi.^2.*WW.* ...

XX+(-1).*phi.^2.*WW.^2.*XX+phi.*WW.*YY+(-1).*phi.^2.*WW.*YY+(-1).* ...

WW.^2.*YY+2.*phi.^2.*WW.^2.*YY+(-1).*phi.*WW.^3.*YY+(-1).*phi.^2.* ...

WW.^3.*YY+(-1).*phi.*XX.*YY+phi.^2.*XX.*YY+WW.*XX.*YY+(-2).*phi.^2.*WW.* ...

XX.*YY+phi.*WW.^2.*XX.*YY+phi.^2.*WW.^2.*XX.*YY+phi.*WW.*ZZ+(-1).* ...

phi.^2.*WW.*ZZ+(-1).*WW.^2.*ZZ+2.*phi.^2.*WW.^2.*ZZ+(-1).*phi.*WW.^3.* ...

ZZ+(-1).*phi.^2.*WW.^3.*ZZ+(-1).*phi.*XX.*ZZ+phi.^2.*XX.*ZZ+WW.*XX.*ZZ+( ...

-2).*phi.^2.*WW.*XX.*ZZ+phi.*WW.^2.*XX.*ZZ+phi.^2.*WW.^2.*XX.*ZZ+ ...

phi.^2.*WW.*YY.*ZZ+(-2).*phi.*WW.^2.*YY.*ZZ+(-2).*phi.^2.*WW.^2.*YY.*ZZ+ ...

WW.^3.*YY.*ZZ+2.*phi.*WW.^3.*YY.*ZZ+phi.^2.*WW.^3.*YY.*ZZ+(-1).*phi.^2.* ...

XX.*YY.*ZZ+2.*phi.*WW.*XX.*YY.*ZZ+2.*phi.^2.*WW.*XX.*YY.*ZZ+(-1).* ...

WW.^2.*XX.*YY.*ZZ+(-2).*phi.*WW.^2.*XX.*YY.*ZZ+(-1).*phi.^2.*WW.^2.*XX.* ...

YY.*ZZ).*(WW.^4+(-1).*WW.^3.*XX+WW.^3.*YY+(-2).*WW.^4.*YY+WW.^4.*XX.*YY+ ...

(-1).*WW.^3.*ZZ+2.*WW.^3.*XX.*ZZ+(-1).*WW.^4.*XX.*ZZ+WW.^4.*YY.*ZZ+(-1) ...

.*WW.^3.*XX.*YY.*ZZ).*(3.*WW.^2+(-4).*phi.*WW.^2+phi.^2.*WW.^2+3.*WW.^3+ ...

2.*phi.*WW.^3+(-2).*phi.^2.*WW.^3+2.*phi.*WW.^4+phi.^2.*WW.^4+(-3).*WW.* ...

XX+4.*phi.*WW.*XX+(-1).*phi.^2.*WW.*XX+(-3).*WW.^2.*XX+(-2).*phi.* ...

WW.^2.*XX+2.*phi.^2.*WW.^2.*XX+(-2).*phi.*WW.^3.*XX+(-1).*phi.^2.* ...

WW.^3.*XX+(-1).*phi.*WW.*YY+phi.^2.*WW.*YY+WW.^2.*YY+2.*phi.*WW.^2.*YY+( ...

-4).*phi.^2.*WW.^2.*YY+(-5).*WW.^3.*YY+5.*phi.*WW.^3.*YY+5.*phi.^2.* ...

WW.^3.*YY+(-2).*WW.^4.*YY+(-6).*phi.*WW.^4.*YY+(-2).*phi.^2.*WW.^4.*YY+ ...

3.*WW.^2.*XX.*YY+(-5).*phi.*WW.^2.*XX.*YY+phi.^2.*WW.^2.*XX.*YY+3.* ...

WW.^3.*XX.*YY+4.*phi.*WW.^3.*XX.*YY+(-2).*phi.^2.*WW.^3.*XX.*YY+phi.* ...

WW.^4.*XX.*YY+phi.^2.*WW.^4.*XX.*YY+phi.*WW.*ZZ+(-1).*phi.^2.*WW.*ZZ+( ...

-3).*WW.^2.*ZZ+4.*phi.*WW.^2.*ZZ+2.*phi.^2.*WW.^2.*ZZ+(-3).*WW.^3.*ZZ+( ...

-5).*phi.*WW.^3.*ZZ+(-1).*phi.^2.*WW.^3.*ZZ+2.*WW.*XX.*ZZ+(-6).*phi.* ...

WW.*XX.*ZZ+2.*phi.^2.*WW.*XX.*ZZ+5.*WW.^2.*XX.*ZZ+5.*phi.*WW.^2.*XX.*ZZ+ ...

(-5).*phi.^2.*WW.^2.*XX.*ZZ+(-1).*WW.^3.*XX.*ZZ+2.*phi.*WW.^3.*XX.*ZZ+ ...

4.*phi.^2.*WW.^3.*XX.*ZZ+(-1).*phi.*WW.^4.*XX.*ZZ+(-1).*phi.^2.*WW.^4.* ...

XX.*ZZ+(-2).*phi.*WW.^2.*YY.*ZZ+phi.^2.*WW.^2.*YY.*ZZ+3.*WW.^3.*YY.*ZZ+( ...

-2).*phi.*WW.^3.*YY.*ZZ+(-2).*phi.^2.*WW.^3.*YY.*ZZ+3.*WW.^4.*YY.*ZZ+4.* ...

phi.*WW.^4.*YY.*ZZ+phi.^2.*WW.^4.*YY.*ZZ+2.*phi.*WW.*XX.*YY.*ZZ+(-1).* ...

phi.^2.*WW.*XX.*YY.*ZZ+(-3).*WW.^2.*XX.*YY.*ZZ+2.*phi.*WW.^2.*XX.*YY.* ...

ZZ+2.*phi.^2.*WW.^2.*XX.*YY.*ZZ+(-3).*WW.^3.*XX.*YY.*ZZ+(-4).*phi.* ...

WW.^3.*XX.*YY.*ZZ+(-1).*phi.^2.*WW.^3.*XX.*YY.*ZZ)+2.*(3.*WW.^2+(-4).* ...

phi.*WW.^2+phi.^2.*WW.^2+3.*WW.^3+2.*phi.*WW.^3+(-2).*phi.^2.*WW.^3+2.* ...

phi.*WW.^4+phi.^2.*WW.^4+(-3).*WW.*XX+4.*phi.*WW.*XX+(-1).*phi.^2.*WW.* ...

XX+(-3).*WW.^2.*XX+(-2).*phi.*WW.^2.*XX+2.*phi.^2.*WW.^2.*XX+(-2).*phi.* ...

WW.^3.*XX+(-1).*phi.^2.*WW.^3.*XX+(-1).*phi.*WW.*YY+phi.^2.*WW.*YY+ ...

WW.^2.*YY+2.*phi.*WW.^2.*YY+(-4).*phi.^2.*WW.^2.*YY+(-5).*WW.^3.*YY+5.* ...

phi.*WW.^3.*YY+5.*phi.^2.*WW.^3.*YY+(-2).*WW.^4.*YY+(-6).*phi.*WW.^4.* ...

YY+(-2).*phi.^2.*WW.^4.*YY+3.*WW.^2.*XX.*YY+(-5).*phi.*WW.^2.*XX.*YY+ ...

phi.^2.*WW.^2.*XX.*YY+3.*WW.^3.*XX.*YY+4.*phi.*WW.^3.*XX.*YY+(-2).* ...

phi.^2.*WW.^3.*XX.*YY+phi.*WW.^4.*XX.*YY+phi.^2.*WW.^4.*XX.*YY+phi.*WW.* ...

ZZ+(-1).*phi.^2.*WW.*ZZ+(-3).*WW.^2.*ZZ+4.*phi.*WW.^2.*ZZ+2.*phi.^2.* ...

WW.^2.*ZZ+(-3).*WW.^3.*ZZ+(-5).*phi.*WW.^3.*ZZ+(-1).*phi.^2.*WW.^3.*ZZ+ ...

2.*WW.*XX.*ZZ+(-6).*phi.*WW.*XX.*ZZ+2.*phi.^2.*WW.*XX.*ZZ+5.*WW.^2.*XX.* ...

ZZ+5.*phi.*WW.^2.*XX.*ZZ+(-5).*phi.^2.*WW.^2.*XX.*ZZ+(-1).*WW.^3.*XX.* ...

ZZ+2.*phi.*WW.^3.*XX.*ZZ+4.*phi.^2.*WW.^3.*XX.*ZZ+(-1).*phi.*WW.^4.*XX.* ...

ZZ+(-1).*phi.^2.*WW.^4.*XX.*ZZ+(-2).*phi.*WW.^2.*YY.*ZZ+phi.^2.*WW.^2.* ...

YY.*ZZ+3.*WW.^3.*YY.*ZZ+(-2).*phi.*WW.^3.*YY.*ZZ+(-2).*phi.^2.*WW.^3.* ...

YY.*ZZ+3.*WW.^4.*YY.*ZZ+4.*phi.*WW.^4.*YY.*ZZ+phi.^2.*WW.^4.*YY.*ZZ+2.* ...

phi.*WW.*XX.*YY.*ZZ+(-1).*phi.^2.*WW.*XX.*YY.*ZZ+(-3).*WW.^2.*XX.*YY.* ...

ZZ+2.*phi.*WW.^2.*XX.*YY.*ZZ+2.*phi.^2.*WW.^2.*XX.*YY.*ZZ+(-3).*WW.^3.* ...

XX.*YY.*ZZ+(-4).*phi.*WW.^3.*XX.*YY.*ZZ+(-1).*phi.^2.*WW.^3.*XX.*YY.*ZZ) ...

.^3+(-9).*((-3).*WW.^3+2.*phi.*WW.^3+(-1).*WW.^4+(-2).*phi.*WW.^4+3.* ...

WW.^2.*XX+(-2).*phi.*WW.^2.*XX+WW.^3.*XX+2.*phi.*WW.^3.*XX+(-1).*WW.^2.* ...

YY+2.*phi.*WW.^2.*YY+WW.^3.*YY+(-6).*phi.*WW.^3.*YY+4.*WW.^4.*YY+4.* ...

phi.*WW.^4.*YY+(-3).*WW.^3.*XX.*YY+2.*phi.*WW.^3.*XX.*YY+(-1).*WW.^4.* ...

XX.*YY+(-2).*phi.*WW.^4.*XX.*YY+WW.^2.*ZZ+(-2).*phi.*WW.^2.*ZZ+3.* ...

WW.^3.*ZZ+2.*phi.*WW.^3.*ZZ+(-4).*WW.^2.*XX.*ZZ+4.*phi.*WW.^2.*XX.*ZZ+( ...

-1).*WW.^3.*XX.*ZZ+(-6).*phi.*WW.^3.*XX.*ZZ+WW.^4.*XX.*ZZ+2.*phi.* ...

WW.^4.*XX.*ZZ+(-1).*WW.^3.*YY.*ZZ+2.*phi.*WW.^3.*YY.*ZZ+(-3).*WW.^4.* ...

YY.*ZZ+(-2).*phi.*WW.^4.*YY.*ZZ+WW.^2.*XX.*YY.*ZZ+(-2).*phi.*WW.^2.*XX.* ...

YY.*ZZ+3.*WW.^3.*XX.*YY.*ZZ+2.*phi.*WW.^3.*XX.*YY.*ZZ).*(3.*WW.^2+(-4).* ...

phi.*WW.^2+phi.^2.*WW.^2+3.*WW.^3+2.*phi.*WW.^3+(-2).*phi.^2.*WW.^3+2.* ...

phi.*WW.^4+phi.^2.*WW.^4+(-3).*WW.*XX+4.*phi.*WW.*XX+(-1).*phi.^2.*WW.* ...

XX+(-3).*WW.^2.*XX+(-2).*phi.*WW.^2.*XX+2.*phi.^2.*WW.^2.*XX+(-2).*phi.* ...

WW.^3.*XX+(-1).*phi.^2.*WW.^3.*XX+(-1).*phi.*WW.*YY+phi.^2.*WW.*YY+ ...

WW.^2.*YY+2.*phi.*WW.^2.*YY+(-4).*phi.^2.*WW.^2.*YY+(-5).*WW.^3.*YY+5.* ...

phi.*WW.^3.*YY+5.*phi.^2.*WW.^3.*YY+(-2).*WW.^4.*YY+(-6).*phi.*WW.^4.* ...

YY+(-2).*phi.^2.*WW.^4.*YY+3.*WW.^2.*XX.*YY+(-5).*phi.*WW.^2.*XX.*YY+ ...

phi.^2.*WW.^2.*XX.*YY+3.*WW.^3.*XX.*YY+4.*phi.*WW.^3.*XX.*YY+(-2).* ...

phi.^2.*WW.^3.*XX.*YY+phi.*WW.^4.*XX.*YY+phi.^2.*WW.^4.*XX.*YY+phi.*WW.* ...

ZZ+(-1).*phi.^2.*WW.*ZZ+(-3).*WW.^2.*ZZ+4.*phi.*WW.^2.*ZZ+2.*phi.^2.* ...

WW.^2.*ZZ+(-3).*WW.^3.*ZZ+(-5).*phi.*WW.^3.*ZZ+(-1).*phi.^2.*WW.^3.*ZZ+ ...

2.*WW.*XX.*ZZ+(-6).*phi.*WW.*XX.*ZZ+2.*phi.^2.*WW.*XX.*ZZ+5.*WW.^2.*XX.* ...

ZZ+5.*phi.*WW.^2.*XX.*ZZ+(-5).*phi.^2.*WW.^2.*XX.*ZZ+(-1).*WW.^3.*XX.* ...

ZZ+2.*phi.*WW.^3.*XX.*ZZ+4.*phi.^2.*WW.^3.*XX.*ZZ+(-1).*phi.*WW.^4.*XX.* ...

ZZ+(-1).*phi.^2.*WW.^4.*XX.*ZZ+(-2).*phi.*WW.^2.*YY.*ZZ+phi.^2.*WW.^2.* ...

YY.*ZZ+3.*WW.^3.*YY.*ZZ+(-2).*phi.*WW.^3.*YY.*ZZ+(-2).*phi.^2.*WW.^3.* ...

YY.*ZZ+3.*WW.^4.*YY.*ZZ+4.*phi.*WW.^4.*YY.*ZZ+phi.^2.*WW.^4.*YY.*ZZ+2.* ...

phi.*WW.*XX.*YY.*ZZ+(-1).*phi.^2.*WW.*XX.*YY.*ZZ+(-3).*WW.^2.*XX.*YY.* ...

ZZ+2.*phi.*WW.^2.*XX.*YY.*ZZ+2.*phi.^2.*WW.^2.*XX.*YY.*ZZ+(-3).*WW.^3.* ...

XX.*YY.*ZZ+(-4).*phi.*WW.^3.*XX.*YY.*ZZ+(-1).*phi.^2.*WW.^3.*XX.*YY.*ZZ) ...

.*((-1).*WW+2.*phi.*WW+(-1).*phi.^2.*WW+(-3).*WW.^2+2.*phi.*WW.^2+ ...

phi.^2.*WW.^2+(-4).*phi.*WW.^3+phi.^2.*WW.^3+(-1).*phi.^2.*WW.^4+XX+(-2) ...

.*phi.*XX+phi.^2.*XX+3.*WW.*XX+(-2).*phi.*WW.*XX+(-1).*phi.^2.*WW.*XX+ ...

4.*phi.*WW.^2.*XX+(-1).*phi.^2.*WW.^2.*XX+phi.^2.*WW.^3.*XX+WW.^2.*YY+( ...

-4).*phi.*WW.^2.*YY+2.*phi.^2.*WW.^2.*YY+3.*WW.^3.*YY+2.*phi.*WW.^3.*YY+ ...

(-4).*phi.^2.*WW.^3.*YY+2.*phi.*WW.^4.*YY+2.*phi.^2.*WW.^4.*YY+(-1).* ...

WW.*XX.*YY+4.*phi.*WW.*XX.*YY+(-2).*phi.^2.*WW.*XX.*YY+(-3).*WW.^2.*XX.* ...

YY+(-2).*phi.*WW.^2.*XX.*YY+4.*phi.^2.*WW.^2.*XX.*YY+(-2).*phi.*WW.^3.* ...

XX.*YY+(-2).*phi.^2.*WW.^3.*XX.*YY+(-2).*phi.*WW.*ZZ+2.*phi.^2.*WW.*ZZ+ ...

3.*WW.^2.*ZZ+(-2).*phi.*WW.^2.*ZZ+(-4).*phi.^2.*WW.^2.*ZZ+WW.^3.*ZZ+4.* ...

phi.*WW.^3.*ZZ+2.*phi.^2.*WW.^3.*ZZ+2.*phi.*XX.*ZZ+(-2).*phi.^2.*XX.*ZZ+ ...

(-3).*WW.*XX.*ZZ+2.*phi.*WW.*XX.*ZZ+4.*phi.^2.*WW.*XX.*ZZ+(-1).*WW.^2.* ...

XX.*ZZ+(-4).*phi.*WW.^2.*XX.*ZZ+(-2).*phi.^2.*WW.^2.*XX.*ZZ+(-1).* ...

phi.^2.*WW.*YY.*ZZ+4.*phi.*WW.^2.*YY.*ZZ+phi.^2.*WW.^2.*YY.*ZZ+(-3).* ...

WW.^3.*YY.*ZZ+(-2).*phi.*WW.^3.*YY.*ZZ+phi.^2.*WW.^3.*YY.*ZZ+(-1).* ...

WW.^4.*YY.*ZZ+(-2).*phi.*WW.^4.*YY.*ZZ+(-1).*phi.^2.*WW.^4.*YY.*ZZ+ ...

phi.^2.*XX.*YY.*ZZ+(-4).*phi.*WW.*XX.*YY.*ZZ+(-1).*phi.^2.*WW.*XX.*YY.* ...

ZZ+3.*WW.^2.*XX.*YY.*ZZ+2.*phi.*WW.^2.*XX.*YY.*ZZ+(-1).*phi.^2.*WW.^2.* ...

XX.*YY.*ZZ+WW.^3.*XX.*YY.*ZZ+2.*phi.*WW.^3.*XX.*YY.*ZZ+phi.^2.*WW.^3.* ...

XX.*YY.*ZZ)+27.*(WW.^4+(-1).*WW.^3.*XX+WW.^3.*YY+(-2).*WW.^4.*YY+WW.^4.* ...

XX.*YY+(-1).*WW.^3.*ZZ+2.*WW.^3.*XX.*ZZ+(-1).*WW.^4.*XX.*ZZ+WW.^4.*YY.* ...

ZZ+(-1).*WW.^3.*XX.*YY.*ZZ).*((-1).*WW+2.*phi.*WW+(-1).*phi.^2.*WW+(-3) ...

.*WW.^2+2.*phi.*WW.^2+phi.^2.*WW.^2+(-4).*phi.*WW.^3+phi.^2.*WW.^3+(-1) ...

.*phi.^2.*WW.^4+XX+(-2).*phi.*XX+phi.^2.*XX+3.*WW.*XX+(-2).*phi.*WW.*XX+ ...

(-1).*phi.^2.*WW.*XX+4.*phi.*WW.^2.*XX+(-1).*phi.^2.*WW.^2.*XX+phi.^2.* ...

WW.^3.*XX+WW.^2.*YY+(-4).*phi.*WW.^2.*YY+2.*phi.^2.*WW.^2.*YY+3.*WW.^3.* ...

YY+2.*phi.*WW.^3.*YY+(-4).*phi.^2.*WW.^3.*YY+2.*phi.*WW.^4.*YY+2.* ...

phi.^2.*WW.^4.*YY+(-1).*WW.*XX.*YY+4.*phi.*WW.*XX.*YY+(-2).*phi.^2.*WW.* ...

XX.*YY+(-3).*WW.^2.*XX.*YY+(-2).*phi.*WW.^2.*XX.*YY+4.*phi.^2.*WW.^2.* ...

XX.*YY+(-2).*phi.*WW.^3.*XX.*YY+(-2).*phi.^2.*WW.^3.*XX.*YY+(-2).*phi.* ...

WW.*ZZ+2.*phi.^2.*WW.*ZZ+3.*WW.^2.*ZZ+(-2).*phi.*WW.^2.*ZZ+(-4).* ...

phi.^2.*WW.^2.*ZZ+WW.^3.*ZZ+4.*phi.*WW.^3.*ZZ+2.*phi.^2.*WW.^3.*ZZ+2.* ...

phi.*XX.*ZZ+(-2).*phi.^2.*XX.*ZZ+(-3).*WW.*XX.*ZZ+2.*phi.*WW.*XX.*ZZ+4.* ...

phi.^2.*WW.*XX.*ZZ+(-1).*WW.^2.*XX.*ZZ+(-4).*phi.*WW.^2.*XX.*ZZ+(-2).* ...

phi.^2.*WW.^2.*XX.*ZZ+(-1).*phi.^2.*WW.*YY.*ZZ+4.*phi.*WW.^2.*YY.*ZZ+ ...

phi.^2.*WW.^2.*YY.*ZZ+(-3).*WW.^3.*YY.*ZZ+(-2).*phi.*WW.^3.*YY.*ZZ+ ...

phi.^2.*WW.^3.*YY.*ZZ+(-1).*WW.^4.*YY.*ZZ+(-2).*phi.*WW.^4.*YY.*ZZ+(-1) ...

.*phi.^2.*WW.^4.*YY.*ZZ+phi.^2.*XX.*YY.*ZZ+(-4).*phi.*WW.*XX.*YY.*ZZ+( ...

-1).*phi.^2.*WW.*XX.*YY.*ZZ+3.*WW.^2.*XX.*YY.*ZZ+2.*phi.*WW.^2.*XX.*YY.* ...

ZZ+(-1).*phi.^2.*WW.^2.*XX.*YY.*ZZ+WW.^3.*XX.*YY.*ZZ+2.*phi.*WW.^3.*XX.* ...

YY.*ZZ+phi.^2.*WW.^3.*XX.*YY.*ZZ).^2).^2).^(1/2)).^(-1/3)+(1/3).*2.^( ...

-1/3).*(WW+(-2).*phi.*WW+phi.^2.*WW+2.*phi.*WW.^2+(-2).*phi.^2.*WW.^2+ ...

phi.^2.*WW.^3+(-1).*XX+2.*phi.*XX+(-1).*phi.^2.*XX+(-2).*phi.*WW.*XX+2.* ...

phi.^2.*WW.*XX+(-1).*phi.^2.*WW.^2.*XX+phi.*WW.*YY+(-1).*phi.^2.*WW.*YY+ ...

(-1).*WW.^2.*YY+2.*phi.^2.*WW.^2.*YY+(-1).*phi.*WW.^3.*YY+(-1).*phi.^2.* ...

WW.^3.*YY+(-1).*phi.*XX.*YY+phi.^2.*XX.*YY+WW.*XX.*YY+(-2).*phi.^2.*WW.* ...

XX.*YY+phi.*WW.^2.*XX.*YY+phi.^2.*WW.^2.*XX.*YY+phi.*WW.*ZZ+(-1).* ...

phi.^2.*WW.*ZZ+(-1).*WW.^2.*ZZ+2.*phi.^2.*WW.^2.*ZZ+(-1).*phi.*WW.^3.* ...

ZZ+(-1).*phi.^2.*WW.^3.*ZZ+(-1).*phi.*XX.*ZZ+phi.^2.*XX.*ZZ+WW.*XX.*ZZ+( ...

-2).*phi.^2.*WW.*XX.*ZZ+phi.*WW.^2.*XX.*ZZ+phi.^2.*WW.^2.*XX.*ZZ+ ...

phi.^2.*WW.*YY.*ZZ+(-2).*phi.*WW.^2.*YY.*ZZ+(-2).*phi.^2.*WW.^2.*YY.*ZZ+ ...

WW.^3.*YY.*ZZ+2.*phi.*WW.^3.*YY.*ZZ+phi.^2.*WW.^3.*YY.*ZZ+(-1).*phi.^2.* ...

XX.*YY.*ZZ+2.*phi.*WW.*XX.*YY.*ZZ+2.*phi.^2.*WW.*XX.*YY.*ZZ+(-1).* ...

WW.^2.*XX.*YY.*ZZ+(-2).*phi.*WW.^2.*XX.*YY.*ZZ+(-1).*phi.^2.*WW.^2.*XX.* ...

YY.*ZZ).^(-1).*(27.*(WW+(-2).*phi.*WW+phi.^2.*WW+2.*phi.*WW.^2+(-2).* ...

phi.^2.*WW.^2+phi.^2.*WW.^3+(-1).*XX+2.*phi.*XX+(-1).*phi.^2.*XX+(-2).* ...

phi.*WW.*XX+2.*phi.^2.*WW.*XX+(-1).*phi.^2.*WW.^2.*XX+phi.*WW.*YY+(-1).* ...

phi.^2.*WW.*YY+(-1).*WW.^2.*YY+2.*phi.^2.*WW.^2.*YY+(-1).*phi.*WW.^3.* ...

YY+(-1).*phi.^2.*WW.^3.*YY+(-1).*phi.*XX.*YY+phi.^2.*XX.*YY+WW.*XX.*YY+( ...

-2).*phi.^2.*WW.*XX.*YY+phi.*WW.^2.*XX.*YY+phi.^2.*WW.^2.*XX.*YY+phi.* ...

WW.*ZZ+(-1).*phi.^2.*WW.*ZZ+(-1).*WW.^2.*ZZ+2.*phi.^2.*WW.^2.*ZZ+(-1).* ...

phi.*WW.^3.*ZZ+(-1).*phi.^2.*WW.^3.*ZZ+(-1).*phi.*XX.*ZZ+phi.^2.*XX.*ZZ+ ...

WW.*XX.*ZZ+(-2).*phi.^2.*WW.*XX.*ZZ+phi.*WW.^2.*XX.*ZZ+phi.^2.*WW.^2.* ...

XX.*ZZ+phi.^2.*WW.*YY.*ZZ+(-2).*phi.*WW.^2.*YY.*ZZ+(-2).*phi.^2.*WW.^2.* ...

YY.*ZZ+WW.^3.*YY.*ZZ+2.*phi.*WW.^3.*YY.*ZZ+phi.^2.*WW.^3.*YY.*ZZ+(-1).* ...

phi.^2.*XX.*YY.*ZZ+2.*phi.*WW.*XX.*YY.*ZZ+2.*phi.^2.*WW.*XX.*YY.*ZZ+(-1) ...

.*WW.^2.*XX.*YY.*ZZ+(-2).*phi.*WW.^2.*XX.*YY.*ZZ+(-1).*phi.^2.*WW.^2.* ...

XX.*YY.*ZZ).*((-3).*WW.^3+2.*phi.*WW.^3+(-1).*WW.^4+(-2).*phi.*WW.^4+3.* ...

WW.^2.*XX+(-2).*phi.*WW.^2.*XX+WW.^3.*XX+2.*phi.*WW.^3.*XX+(-1).*WW.^2.* ...

YY+2.*phi.*WW.^2.*YY+WW.^3.*YY+(-6).*phi.*WW.^3.*YY+4.*WW.^4.*YY+4.* ...

phi.*WW.^4.*YY+(-3).*WW.^3.*XX.*YY+2.*phi.*WW.^3.*XX.*YY+(-1).*WW.^4.* ...

XX.*YY+(-2).*phi.*WW.^4.*XX.*YY+WW.^2.*ZZ+(-2).*phi.*WW.^2.*ZZ+3.* ...

WW.^3.*ZZ+2.*phi.*WW.^3.*ZZ+(-4).*WW.^2.*XX.*ZZ+4.*phi.*WW.^2.*XX.*ZZ+( ...

-1).*WW.^3.*XX.*ZZ+(-6).*phi.*WW.^3.*XX.*ZZ+WW.^4.*XX.*ZZ+2.*phi.* ...

WW.^4.*XX.*ZZ+(-1).*WW.^3.*YY.*ZZ+2.*phi.*WW.^3.*YY.*ZZ+(-3).*WW.^4.* ...

YY.*ZZ+(-2).*phi.*WW.^4.*YY.*ZZ+WW.^2.*XX.*YY.*ZZ+(-2).*phi.*WW.^2.*XX.* ...

YY.*ZZ+3.*WW.^3.*XX.*YY.*ZZ+2.*phi.*WW.^3.*XX.*YY.*ZZ).^2+(-72).*(WW+( ...

-2).*phi.*WW+phi.^2.*WW+2.*phi.*WW.^2+(-2).*phi.^2.*WW.^2+phi.^2.*WW.^3+ ...

(-1).*XX+2.*phi.*XX+(-1).*phi.^2.*XX+(-2).*phi.*WW.*XX+2.*phi.^2.*WW.* ...

XX+(-1).*phi.^2.*WW.^2.*XX+phi.*WW.*YY+(-1).*phi.^2.*WW.*YY+(-1).* ...

WW.^2.*YY+2.*phi.^2.*WW.^2.*YY+(-1).*phi.*WW.^3.*YY+(-1).*phi.^2.* ...

WW.^3.*YY+(-1).*phi.*XX.*YY+phi.^2.*XX.*YY+WW.*XX.*YY+(-2).*phi.^2.*WW.* ...

XX.*YY+phi.*WW.^2.*XX.*YY+phi.^2.*WW.^2.*XX.*YY+phi.*WW.*ZZ+(-1).* ...

phi.^2.*WW.*ZZ+(-1).*WW.^2.*ZZ+2.*phi.^2.*WW.^2.*ZZ+(-1).*phi.*WW.^3.* ...

ZZ+(-1).*phi.^2.*WW.^3.*ZZ+(-1).*phi.*XX.*ZZ+phi.^2.*XX.*ZZ+WW.*XX.*ZZ+( ...

-2).*phi.^2.*WW.*XX.*ZZ+phi.*WW.^2.*XX.*ZZ+phi.^2.*WW.^2.*XX.*ZZ+ ...

phi.^2.*WW.*YY.*ZZ+(-2).*phi.*WW.^2.*YY.*ZZ+(-2).*phi.^2.*WW.^2.*YY.*ZZ+ ...

WW.^3.*YY.*ZZ+2.*phi.*WW.^3.*YY.*ZZ+phi.^2.*WW.^3.*YY.*ZZ+(-1).*phi.^2.* ...

XX.*YY.*ZZ+2.*phi.*WW.*XX.*YY.*ZZ+2.*phi.^2.*WW.*XX.*YY.*ZZ+(-1).* ...

WW.^2.*XX.*YY.*ZZ+(-2).*phi.*WW.^2.*XX.*YY.*ZZ+(-1).*phi.^2.*WW.^2.*XX.* ...

YY.*ZZ).*(WW.^4+(-1).*WW.^3.*XX+WW.^3.*YY+(-2).*WW.^4.*YY+WW.^4.*XX.*YY+ ...

(-1).*WW.^3.*ZZ+2.*WW.^3.*XX.*ZZ+(-1).*WW.^4.*XX.*ZZ+WW.^4.*YY.*ZZ+(-1) ...

.*WW.^3.*XX.*YY.*ZZ).*(3.*WW.^2+(-4).*phi.*WW.^2+phi.^2.*WW.^2+3.*WW.^3+ ...

2.*phi.*WW.^3+(-2).*phi.^2.*WW.^3+2.*phi.*WW.^4+phi.^2.*WW.^4+(-3).*WW.* ...

XX+4.*phi.*WW.*XX+(-1).*phi.^2.*WW.*XX+(-3).*WW.^2.*XX+(-2).*phi.* ...

WW.^2.*XX+2.*phi.^2.*WW.^2.*XX+(-2).*phi.*WW.^3.*XX+(-1).*phi.^2.* ...

WW.^3.*XX+(-1).*phi.*WW.*YY+phi.^2.*WW.*YY+WW.^2.*YY+2.*phi.*WW.^2.*YY+( ...

-4).*phi.^2.*WW.^2.*YY+(-5).*WW.^3.*YY+5.*phi.*WW.^3.*YY+5.*phi.^2.* ...

WW.^3.*YY+(-2).*WW.^4.*YY+(-6).*phi.*WW.^4.*YY+(-2).*phi.^2.*WW.^4.*YY+ ...

3.*WW.^2.*XX.*YY+(-5).*phi.*WW.^2.*XX.*YY+phi.^2.*WW.^2.*XX.*YY+3.* ...

WW.^3.*XX.*YY+4.*phi.*WW.^3.*XX.*YY+(-2).*phi.^2.*WW.^3.*XX.*YY+phi.* ...

WW.^4.*XX.*YY+phi.^2.*WW.^4.*XX.*YY+phi.*WW.*ZZ+(-1).*phi.^2.*WW.*ZZ+( ...

-3).*WW.^2.*ZZ+4.*phi.*WW.^2.*ZZ+2.*phi.^2.*WW.^2.*ZZ+(-3).*WW.^3.*ZZ+( ...

-5).*phi.*WW.^3.*ZZ+(-1).*phi.^2.*WW.^3.*ZZ+2.*WW.*XX.*ZZ+(-6).*phi.* ...

WW.*XX.*ZZ+2.*phi.^2.*WW.*XX.*ZZ+5.*WW.^2.*XX.*ZZ+5.*phi.*WW.^2.*XX.*ZZ+ ...

(-5).*phi.^2.*WW.^2.*XX.*ZZ+(-1).*WW.^3.*XX.*ZZ+2.*phi.*WW.^3.*XX.*ZZ+ ...

4.*phi.^2.*WW.^3.*XX.*ZZ+(-1).*phi.*WW.^4.*XX.*ZZ+(-1).*phi.^2.*WW.^4.* ...

XX.*ZZ+(-2).*phi.*WW.^2.*YY.*ZZ+phi.^2.*WW.^2.*YY.*ZZ+3.*WW.^3.*YY.*ZZ+( ...

-2).*phi.*WW.^3.*YY.*ZZ+(-2).*phi.^2.*WW.^3.*YY.*ZZ+3.*WW.^4.*YY.*ZZ+4.* ...

phi.*WW.^4.*YY.*ZZ+phi.^2.*WW.^4.*YY.*ZZ+2.*phi.*WW.*XX.*YY.*ZZ+(-1).* ...

phi.^2.*WW.*XX.*YY.*ZZ+(-3).*WW.^2.*XX.*YY.*ZZ+2.*phi.*WW.^2.*XX.*YY.* ...

ZZ+2.*phi.^2.*WW.^2.*XX.*YY.*ZZ+(-3).*WW.^3.*XX.*YY.*ZZ+(-4).*phi.* ...

WW.^3.*XX.*YY.*ZZ+(-1).*phi.^2.*WW.^3.*XX.*YY.*ZZ)+2.*(3.*WW.^2+(-4).* ...

phi.*WW.^2+phi.^2.*WW.^2+3.*WW.^3+2.*phi.*WW.^3+(-2).*phi.^2.*WW.^3+2.* ...

phi.*WW.^4+phi.^2.*WW.^4+(-3).*WW.*XX+4.*phi.*WW.*XX+(-1).*phi.^2.*WW.* ...

XX+(-3).*WW.^2.*XX+(-2).*phi.*WW.^2.*XX+2.*phi.^2.*WW.^2.*XX+(-2).*phi.* ...

WW.^3.*XX+(-1).*phi.^2.*WW.^3.*XX+(-1).*phi.*WW.*YY+phi.^2.*WW.*YY+ ...

WW.^2.*YY+2.*phi.*WW.^2.*YY+(-4).*phi.^2.*WW.^2.*YY+(-5).*WW.^3.*YY+5.* ...

phi.*WW.^3.*YY+5.*phi.^2.*WW.^3.*YY+(-2).*WW.^4.*YY+(-6).*phi.*WW.^4.* ...

YY+(-2).*phi.^2.*WW.^4.*YY+3.*WW.^2.*XX.*YY+(-5).*phi.*WW.^2.*XX.*YY+ ...

phi.^2.*WW.^2.*XX.*YY+3.*WW.^3.*XX.*YY+4.*phi.*WW.^3.*XX.*YY+(-2).* ...

phi.^2.*WW.^3.*XX.*YY+phi.*WW.^4.*XX.*YY+phi.^2.*WW.^4.*XX.*YY+phi.*WW.* ...

ZZ+(-1).*phi.^2.*WW.*ZZ+(-3).*WW.^2.*ZZ+4.*phi.*WW.^2.*ZZ+2.*phi.^2.* ...

WW.^2.*ZZ+(-3).*WW.^3.*ZZ+(-5).*phi.*WW.^3.*ZZ+(-1).*phi.^2.*WW.^3.*ZZ+ ...

2.*WW.*XX.*ZZ+(-6).*phi.*WW.*XX.*ZZ+2.*phi.^2.*WW.*XX.*ZZ+5.*WW.^2.*XX.* ...

ZZ+5.*phi.*WW.^2.*XX.*ZZ+(-5).*phi.^2.*WW.^2.*XX.*ZZ+(-1).*WW.^3.*XX.* ...

ZZ+2.*phi.*WW.^3.*XX.*ZZ+4.*phi.^2.*WW.^3.*XX.*ZZ+(-1).*phi.*WW.^4.*XX.* ...

ZZ+(-1).*phi.^2.*WW.^4.*XX.*ZZ+(-2).*phi.*WW.^2.*YY.*ZZ+phi.^2.*WW.^2.* ...

YY.*ZZ+3.*WW.^3.*YY.*ZZ+(-2).*phi.*WW.^3.*YY.*ZZ+(-2).*phi.^2.*WW.^3.* ...

YY.*ZZ+3.*WW.^4.*YY.*ZZ+4.*phi.*WW.^4.*YY.*ZZ+phi.^2.*WW.^4.*YY.*ZZ+2.* ...

phi.*WW.*XX.*YY.*ZZ+(-1).*phi.^2.*WW.*XX.*YY.*ZZ+(-3).*WW.^2.*XX.*YY.* ...

ZZ+2.*phi.*WW.^2.*XX.*YY.*ZZ+2.*phi.^2.*WW.^2.*XX.*YY.*ZZ+(-3).*WW.^3.* ...

XX.*YY.*ZZ+(-4).*phi.*WW.^3.*XX.*YY.*ZZ+(-1).*phi.^2.*WW.^3.*XX.*YY.*ZZ) ...

.^3+(-9).*((-3).*WW.^3+2.*phi.*WW.^3+(-1).*WW.^4+(-2).*phi.*WW.^4+3.* ...

WW.^2.*XX+(-2).*phi.*WW.^2.*XX+WW.^3.*XX+2.*phi.*WW.^3.*XX+(-1).*WW.^2.* ...

YY+2.*phi.*WW.^2.*YY+WW.^3.*YY+(-6).*phi.*WW.^3.*YY+4.*WW.^4.*YY+4.* ...

phi.*WW.^4.*YY+(-3).*WW.^3.*XX.*YY+2.*phi.*WW.^3.*XX.*YY+(-1).*WW.^4.* ...

XX.*YY+(-2).*phi.*WW.^4.*XX.*YY+WW.^2.*ZZ+(-2).*phi.*WW.^2.*ZZ+3.* ...

WW.^3.*ZZ+2.*phi.*WW.^3.*ZZ+(-4).*WW.^2.*XX.*ZZ+4.*phi.*WW.^2.*XX.*ZZ+( ...

-1).*WW.^3.*XX.*ZZ+(-6).*phi.*WW.^3.*XX.*ZZ+WW.^4.*XX.*ZZ+2.*phi.* ...

WW.^4.*XX.*ZZ+(-1).*WW.^3.*YY.*ZZ+2.*phi.*WW.^3.*YY.*ZZ+(-3).*WW.^4.* ...

YY.*ZZ+(-2).*phi.*WW.^4.*YY.*ZZ+WW.^2.*XX.*YY.*ZZ+(-2).*phi.*WW.^2.*XX.* ...

YY.*ZZ+3.*WW.^3.*XX.*YY.*ZZ+2.*phi.*WW.^3.*XX.*YY.*ZZ).*(3.*WW.^2+(-4).* ...

phi.*WW.^2+phi.^2.*WW.^2+3.*WW.^3+2.*phi.*WW.^3+(-2).*phi.^2.*WW.^3+2.* ...

phi.*WW.^4+phi.^2.*WW.^4+(-3).*WW.*XX+4.*phi.*WW.*XX+(-1).*phi.^2.*WW.* ...

XX+(-3).*WW.^2.*XX+(-2).*phi.*WW.^2.*XX+2.*phi.^2.*WW.^2.*XX+(-2).*phi.* ...

WW.^3.*XX+(-1).*phi.^2.*WW.^3.*XX+(-1).*phi.*WW.*YY+phi.^2.*WW.*YY+ ...

WW.^2.*YY+2.*phi.*WW.^2.*YY+(-4).*phi.^2.*WW.^2.*YY+(-5).*WW.^3.*YY+5.* ...

phi.*WW.^3.*YY+5.*phi.^2.*WW.^3.*YY+(-2).*WW.^4.*YY+(-6).*phi.*WW.^4.* ...

YY+(-2).*phi.^2.*WW.^4.*YY+3.*WW.^2.*XX.*YY+(-5).*phi.*WW.^2.*XX.*YY+ ...

phi.^2.*WW.^2.*XX.*YY+3.*WW.^3.*XX.*YY+4.*phi.*WW.^3.*XX.*YY+(-2).* ...

phi.^2.*WW.^3.*XX.*YY+phi.*WW.^4.*XX.*YY+phi.^2.*WW.^4.*XX.*YY+phi.*WW.* ...

ZZ+(-1).*phi.^2.*WW.*ZZ+(-3).*WW.^2.*ZZ+4.*phi.*WW.^2.*ZZ+2.*phi.^2.* ...

WW.^2.*ZZ+(-3).*WW.^3.*ZZ+(-5).*phi.*WW.^3.*ZZ+(-1).*phi.^2.*WW.^3.*ZZ+ ...

2.*WW.*XX.*ZZ+(-6).*phi.*WW.*XX.*ZZ+2.*phi.^2.*WW.*XX.*ZZ+5.*WW.^2.*XX.* ...

ZZ+5.*phi.*WW.^2.*XX.*ZZ+(-5).*phi.^2.*WW.^2.*XX.*ZZ+(-1).*WW.^3.*XX.* ...

ZZ+2.*phi.*WW.^3.*XX.*ZZ+4.*phi.^2.*WW.^3.*XX.*ZZ+(-1).*phi.*WW.^4.*XX.* ...

ZZ+(-1).*phi.^2.*WW.^4.*XX.*ZZ+(-2).*phi.*WW.^2.*YY.*ZZ+phi.^2.*WW.^2.* ...

YY.*ZZ+3.*WW.^3.*YY.*ZZ+(-2).*phi.*WW.^3.*YY.*ZZ+(-2).*phi.^2.*WW.^3.* ...

YY.*ZZ+3.*WW.^4.*YY.*ZZ+4.*phi.*WW.^4.*YY.*ZZ+phi.^2.*WW.^4.*YY.*ZZ+2.* ...

phi.*WW.*XX.*YY.*ZZ+(-1).*phi.^2.*WW.*XX.*YY.*ZZ+(-3).*WW.^2.*XX.*YY.* ...

ZZ+2.*phi.*WW.^2.*XX.*YY.*ZZ+2.*phi.^2.*WW.^2.*XX.*YY.*ZZ+(-3).*WW.^3.* ...

XX.*YY.*ZZ+(-4).*phi.*WW.^3.*XX.*YY.*ZZ+(-1).*phi.^2.*WW.^3.*XX.*YY.*ZZ) ...

.*((-1).*WW+2.*phi.*WW+(-1).*phi.^2.*WW+(-3).*WW.^2+2.*phi.*WW.^2+ ...

phi.^2.*WW.^2+(-4).*phi.*WW.^3+phi.^2.*WW.^3+(-1).*phi.^2.*WW.^4+XX+(-2) ...

.*phi.*XX+phi.^2.*XX+3.*WW.*XX+(-2).*phi.*WW.*XX+(-1).*phi.^2.*WW.*XX+ ...

4.*phi.*WW.^2.*XX+(-1).*phi.^2.*WW.^2.*XX+phi.^2.*WW.^3.*XX+WW.^2.*YY+( ...

-4).*phi.*WW.^2.*YY+2.*phi.^2.*WW.^2.*YY+3.*WW.^3.*YY+2.*phi.*WW.^3.*YY+ ...

(-4).*phi.^2.*WW.^3.*YY+2.*phi.*WW.^4.*YY+2.*phi.^2.*WW.^4.*YY+(-1).* ...

WW.*XX.*YY+4.*phi.*WW.*XX.*YY+(-2).*phi.^2.*WW.*XX.*YY+(-3).*WW.^2.*XX.* ...

YY+(-2).*phi.*WW.^2.*XX.*YY+4.*phi.^2.*WW.^2.*XX.*YY+(-2).*phi.*WW.^3.* ...

XX.*YY+(-2).*phi.^2.*WW.^3.*XX.*YY+(-2).*phi.*WW.*ZZ+2.*phi.^2.*WW.*ZZ+ ...

3.*WW.^2.*ZZ+(-2).*phi.*WW.^2.*ZZ+(-4).*phi.^2.*WW.^2.*ZZ+WW.^3.*ZZ+4.* ...

phi.*WW.^3.*ZZ+2.*phi.^2.*WW.^3.*ZZ+2.*phi.*XX.*ZZ+(-2).*phi.^2.*XX.*ZZ+ ...

(-3).*WW.*XX.*ZZ+2.*phi.*WW.*XX.*ZZ+4.*phi.^2.*WW.*XX.*ZZ+(-1).*WW.^2.* ...

XX.*ZZ+(-4).*phi.*WW.^2.*XX.*ZZ+(-2).*phi.^2.*WW.^2.*XX.*ZZ+(-1).* ...

phi.^2.*WW.*YY.*ZZ+4.*phi.*WW.^2.*YY.*ZZ+phi.^2.*WW.^2.*YY.*ZZ+(-3).* ...

WW.^3.*YY.*ZZ+(-2).*phi.*WW.^3.*YY.*ZZ+phi.^2.*WW.^3.*YY.*ZZ+(-1).* ...

WW.^4.*YY.*ZZ+(-2).*phi.*WW.^4.*YY.*ZZ+(-1).*phi.^2.*WW.^4.*YY.*ZZ+ ...

phi.^2.*XX.*YY.*ZZ+(-4).*phi.*WW.*XX.*YY.*ZZ+(-1).*phi.^2.*WW.*XX.*YY.* ...

ZZ+3.*WW.^2.*XX.*YY.*ZZ+2.*phi.*WW.^2.*XX.*YY.*ZZ+(-1).*phi.^2.*WW.^2.* ...

XX.*YY.*ZZ+WW.^3.*XX.*YY.*ZZ+2.*phi.*WW.^3.*XX.*YY.*ZZ+phi.^2.*WW.^3.* ...

XX.*YY.*ZZ)+27.*(WW.^4+(-1).*WW.^3.*XX+WW.^3.*YY+(-2).*WW.^4.*YY+WW.^4.* ...

XX.*YY+(-1).*WW.^3.*ZZ+2.*WW.^3.*XX.*ZZ+(-1).*WW.^4.*XX.*ZZ+WW.^4.*YY.* ...

ZZ+(-1).*WW.^3.*XX.*YY.*ZZ).*((-1).*WW+2.*phi.*WW+(-1).*phi.^2.*WW+(-3) ...

.*WW.^2+2.*phi.*WW.^2+phi.^2.*WW.^2+(-4).*phi.*WW.^3+phi.^2.*WW.^3+(-1) ...

.*phi.^2.*WW.^4+XX+(-2).*phi.*XX+phi.^2.*XX+3.*WW.*XX+(-2).*phi.*WW.*XX+ ...

(-1).*phi.^2.*WW.*XX+4.*phi.*WW.^2.*XX+(-1).*phi.^2.*WW.^2.*XX+phi.^2.* ...

WW.^3.*XX+WW.^2.*YY+(-4).*phi.*WW.^2.*YY+2.*phi.^2.*WW.^2.*YY+3.*WW.^3.* ...

YY+2.*phi.*WW.^3.*YY+(-4).*phi.^2.*WW.^3.*YY+2.*phi.*WW.^4.*YY+2.* ...

phi.^2.*WW.^4.*YY+(-1).*WW.*XX.*YY+4.*phi.*WW.*XX.*YY+(-2).*phi.^2.*WW.* ...

XX.*YY+(-3).*WW.^2.*XX.*YY+(-2).*phi.*WW.^2.*XX.*YY+4.*phi.^2.*WW.^2.* ...

XX.*YY+(-2).*phi.*WW.^3.*XX.*YY+(-2).*phi.^2.*WW.^3.*XX.*YY+(-2).*phi.* ...

WW.*ZZ+2.*phi.^2.*WW.*ZZ+3.*WW.^2.*ZZ+(-2).*phi.*WW.^2.*ZZ+(-4).* ...

phi.^2.*WW.^2.*ZZ+WW.^3.*ZZ+4.*phi.*WW.^3.*ZZ+2.*phi.^2.*WW.^3.*ZZ+2.* ...

phi.*XX.*ZZ+(-2).*phi.^2.*XX.*ZZ+(-3).*WW.*XX.*ZZ+2.*phi.*WW.*XX.*ZZ+4.* ...

phi.^2.*WW.*XX.*ZZ+(-1).*WW.^2.*XX.*ZZ+(-4).*phi.*WW.^2.*XX.*ZZ+(-2).* ...

phi.^2.*WW.^2.*XX.*ZZ+(-1).*phi.^2.*WW.*YY.*ZZ+4.*phi.*WW.^2.*YY.*ZZ+ ...

phi.^2.*WW.^2.*YY.*ZZ+(-3).*WW.^3.*YY.*ZZ+(-2).*phi.*WW.^3.*YY.*ZZ+ ...

phi.^2.*WW.^3.*YY.*ZZ+(-1).*WW.^4.*YY.*ZZ+(-2).*phi.*WW.^4.*YY.*ZZ+(-1) ...

.*phi.^2.*WW.^4.*YY.*ZZ+phi.^2.*XX.*YY.*ZZ+(-4).*phi.*WW.*XX.*YY.*ZZ+( ...

-1).*phi.^2.*WW.*XX.*YY.*ZZ+3.*WW.^2.*XX.*YY.*ZZ+2.*phi.*WW.^2.*XX.*YY.* ...

ZZ+(-1).*phi.^2.*WW.^2.*XX.*YY.*ZZ+WW.^3.*XX.*YY.*ZZ+2.*phi.*WW.^3.*XX.* ...

YY.*ZZ+phi.^2.*WW.^3.*XX.*YY.*ZZ).^2+((-4).*(12.*(WW+(-2).*phi.*WW+ ...

phi.^2.*WW+2.*phi.*WW.^2+(-2).*phi.^2.*WW.^2+phi.^2.*WW.^3+(-1).*XX+2.* ...

phi.*XX+(-1).*phi.^2.*XX+(-2).*phi.*WW.*XX+2.*phi.^2.*WW.*XX+(-1).* ...

phi.^2.*WW.^2.*XX+phi.*WW.*YY+(-1).*phi.^2.*WW.*YY+(-1).*WW.^2.*YY+2.* ...

phi.^2.*WW.^2.*YY+(-1).*phi.*WW.^3.*YY+(-1).*phi.^2.*WW.^3.*YY+(-1).* ...

phi.*XX.*YY+phi.^2.*XX.*YY+WW.*XX.*YY+(-2).*phi.^2.*WW.*XX.*YY+phi.* ...

WW.^2.*XX.*YY+phi.^2.*WW.^2.*XX.*YY+phi.*WW.*ZZ+(-1).*phi.^2.*WW.*ZZ+( ...

-1).*WW.^2.*ZZ+2.*phi.^2.*WW.^2.*ZZ+(-1).*phi.*WW.^3.*ZZ+(-1).*phi.^2.* ...

WW.^3.*ZZ+(-1).*phi.*XX.*ZZ+phi.^2.*XX.*ZZ+WW.*XX.*ZZ+(-2).*phi.^2.*WW.* ...

XX.*ZZ+phi.*WW.^2.*XX.*ZZ+phi.^2.*WW.^2.*XX.*ZZ+phi.^2.*WW.*YY.*ZZ+(-2) ...

.*phi.*WW.^2.*YY.*ZZ+(-2).*phi.^2.*WW.^2.*YY.*ZZ+WW.^3.*YY.*ZZ+2.*phi.* ...

WW.^3.*YY.*ZZ+phi.^2.*WW.^3.*YY.*ZZ+(-1).*phi.^2.*XX.*YY.*ZZ+2.*phi.* ...

WW.*XX.*YY.*ZZ+2.*phi.^2.*WW.*XX.*YY.*ZZ+(-1).*WW.^2.*XX.*YY.*ZZ+(-2).* ...

phi.*WW.^2.*XX.*YY.*ZZ+(-1).*phi.^2.*WW.^2.*XX.*YY.*ZZ).*(WW.^4+(-1).* ...

WW.^3.*XX+WW.^3.*YY+(-2).*WW.^4.*YY+WW.^4.*XX.*YY+(-1).*WW.^3.*ZZ+2.* ...

WW.^3.*XX.*ZZ+(-1).*WW.^4.*XX.*ZZ+WW.^4.*YY.*ZZ+(-1).*WW.^3.*XX.*YY.*ZZ) ...

+(3.*WW.^2+(-4).*phi.*WW.^2+phi.^2.*WW.^2+3.*WW.^3+2.*phi.*WW.^3+(-2).* ...

phi.^2.*WW.^3+2.*phi.*WW.^4+phi.^2.*WW.^4+(-3).*WW.*XX+4.*phi.*WW.*XX+( ...

-1).*phi.^2.*WW.*XX+(-3).*WW.^2.*XX+(-2).*phi.*WW.^2.*XX+2.*phi.^2.* ...

WW.^2.*XX+(-2).*phi.*WW.^3.*XX+(-1).*phi.^2.*WW.^3.*XX+(-1).*phi.*WW.* ...

YY+phi.^2.*WW.*YY+WW.^2.*YY+2.*phi.*WW.^2.*YY+(-4).*phi.^2.*WW.^2.*YY+( ...

-5).*WW.^3.*YY+5.*phi.*WW.^3.*YY+5.*phi.^2.*WW.^3.*YY+(-2).*WW.^4.*YY+( ...

-6).*phi.*WW.^4.*YY+(-2).*phi.^2.*WW.^4.*YY+3.*WW.^2.*XX.*YY+(-5).*phi.* ...

WW.^2.*XX.*YY+phi.^2.*WW.^2.*XX.*YY+3.*WW.^3.*XX.*YY+4.*phi.*WW.^3.*XX.* ...

YY+(-2).*phi.^2.*WW.^3.*XX.*YY+phi.*WW.^4.*XX.*YY+phi.^2.*WW.^4.*XX.*YY+ ...

phi.*WW.*ZZ+(-1).*phi.^2.*WW.*ZZ+(-3).*WW.^2.*ZZ+4.*phi.*WW.^2.*ZZ+2.* ...

phi.^2.*WW.^2.*ZZ+(-3).*WW.^3.*ZZ+(-5).*phi.*WW.^3.*ZZ+(-1).*phi.^2.* ...

WW.^3.*ZZ+2.*WW.*XX.*ZZ+(-6).*phi.*WW.*XX.*ZZ+2.*phi.^2.*WW.*XX.*ZZ+5.* ...

WW.^2.*XX.*ZZ+5.*phi.*WW.^2.*XX.*ZZ+(-5).*phi.^2.*WW.^2.*XX.*ZZ+(-1).* ...

WW.^3.*XX.*ZZ+2.*phi.*WW.^3.*XX.*ZZ+4.*phi.^2.*WW.^3.*XX.*ZZ+(-1).*phi.* ...

WW.^4.*XX.*ZZ+(-1).*phi.^2.*WW.^4.*XX.*ZZ+(-2).*phi.*WW.^2.*YY.*ZZ+ ...

phi.^2.*WW.^2.*YY.*ZZ+3.*WW.^3.*YY.*ZZ+(-2).*phi.*WW.^3.*YY.*ZZ+(-2).* ...

phi.^2.*WW.^3.*YY.*ZZ+3.*WW.^4.*YY.*ZZ+4.*phi.*WW.^4.*YY.*ZZ+phi.^2.* ...

WW.^4.*YY.*ZZ+2.*phi.*WW.*XX.*YY.*ZZ+(-1).*phi.^2.*WW.*XX.*YY.*ZZ+(-3).* ...

WW.^2.*XX.*YY.*ZZ+2.*phi.*WW.^2.*XX.*YY.*ZZ+2.*phi.^2.*WW.^2.*XX.*YY.* ...

ZZ+(-3).*WW.^3.*XX.*YY.*ZZ+(-4).*phi.*WW.^3.*XX.*YY.*ZZ+(-1).*phi.^2.* ...

WW.^3.*XX.*YY.*ZZ).^2+(-3).*((-3).*WW.^3+2.*phi.*WW.^3+(-1).*WW.^4+(-2) ...

.*phi.*WW.^4+3.*WW.^2.*XX+(-2).*phi.*WW.^2.*XX+WW.^3.*XX+2.*phi.*WW.^3.* ...

XX+(-1).*WW.^2.*YY+2.*phi.*WW.^2.*YY+WW.^3.*YY+(-6).*phi.*WW.^3.*YY+4.* ...

WW.^4.*YY+4.*phi.*WW.^4.*YY+(-3).*WW.^3.*XX.*YY+2.*phi.*WW.^3.*XX.*YY+( ...

-1).*WW.^4.*XX.*YY+(-2).*phi.*WW.^4.*XX.*YY+WW.^2.*ZZ+(-2).*phi.*WW.^2.* ...

ZZ+3.*WW.^3.*ZZ+2.*phi.*WW.^3.*ZZ+(-4).*WW.^2.*XX.*ZZ+4.*phi.*WW.^2.* ...

XX.*ZZ+(-1).*WW.^3.*XX.*ZZ+(-6).*phi.*WW.^3.*XX.*ZZ+WW.^4.*XX.*ZZ+2.* ...

phi.*WW.^4.*XX.*ZZ+(-1).*WW.^3.*YY.*ZZ+2.*phi.*WW.^3.*YY.*ZZ+(-3).* ...

WW.^4.*YY.*ZZ+(-2).*phi.*WW.^4.*YY.*ZZ+WW.^2.*XX.*YY.*ZZ+(-2).*phi.* ...

WW.^2.*XX.*YY.*ZZ+3.*WW.^3.*XX.*YY.*ZZ+2.*phi.*WW.^3.*XX.*YY.*ZZ).*((-1) ...

.*WW+2.*phi.*WW+(-1).*phi.^2.*WW+(-3).*WW.^2+2.*phi.*WW.^2+phi.^2.* ...

WW.^2+(-4).*phi.*WW.^3+phi.^2.*WW.^3+(-1).*phi.^2.*WW.^4+XX+(-2).*phi.* ...

XX+phi.^2.*XX+3.*WW.*XX+(-2).*phi.*WW.*XX+(-1).*phi.^2.*WW.*XX+4.*phi.* ...

WW.^2.*XX+(-1).*phi.^2.*WW.^2.*XX+phi.^2.*WW.^3.*XX+WW.^2.*YY+(-4).* ...

phi.*WW.^2.*YY+2.*phi.^2.*WW.^2.*YY+3.*WW.^3.*YY+2.*phi.*WW.^3.*YY+(-4) ...

.*phi.^2.*WW.^3.*YY+2.*phi.*WW.^4.*YY+2.*phi.^2.*WW.^4.*YY+(-1).*WW.* ...

XX.*YY+4.*phi.*WW.*XX.*YY+(-2).*phi.^2.*WW.*XX.*YY+(-3).*WW.^2.*XX.*YY+( ...

-2).*phi.*WW.^2.*XX.*YY+4.*phi.^2.*WW.^2.*XX.*YY+(-2).*phi.*WW.^3.*XX.* ...

YY+(-2).*phi.^2.*WW.^3.*XX.*YY+(-2).*phi.*WW.*ZZ+2.*phi.^2.*WW.*ZZ+3.* ...

WW.^2.*ZZ+(-2).*phi.*WW.^2.*ZZ+(-4).*phi.^2.*WW.^2.*ZZ+WW.^3.*ZZ+4.* ...

phi.*WW.^3.*ZZ+2.*phi.^2.*WW.^3.*ZZ+2.*phi.*XX.*ZZ+(-2).*phi.^2.*XX.*ZZ+ ...

(-3).*WW.*XX.*ZZ+2.*phi.*WW.*XX.*ZZ+4.*phi.^2.*WW.*XX.*ZZ+(-1).*WW.^2.* ...

XX.*ZZ+(-4).*phi.*WW.^2.*XX.*ZZ+(-2).*phi.^2.*WW.^2.*XX.*ZZ+(-1).* ...

phi.^2.*WW.*YY.*ZZ+4.*phi.*WW.^2.*YY.*ZZ+phi.^2.*WW.^2.*YY.*ZZ+(-3).* ...

WW.^3.*YY.*ZZ+(-2).*phi.*WW.^3.*YY.*ZZ+phi.^2.*WW.^3.*YY.*ZZ+(-1).* ...

WW.^4.*YY.*ZZ+(-2).*phi.*WW.^4.*YY.*ZZ+(-1).*phi.^2.*WW.^4.*YY.*ZZ+ ...

phi.^2.*XX.*YY.*ZZ+(-4).*phi.*WW.*XX.*YY.*ZZ+(-1).*phi.^2.*WW.*XX.*YY.* ...

ZZ+3.*WW.^2.*XX.*YY.*ZZ+2.*phi.*WW.^2.*XX.*YY.*ZZ+(-1).*phi.^2.*WW.^2.* ...

XX.*YY.*ZZ+WW.^3.*XX.*YY.*ZZ+2.*phi.*WW.^3.*XX.*YY.*ZZ+phi.^2.*WW.^3.* ...

XX.*YY.*ZZ)).^3+(27.*(WW+(-2).*phi.*WW+phi.^2.*WW+2.*phi.*WW.^2+(-2).* ...

phi.^2.*WW.^2+phi.^2.*WW.^3+(-1).*XX+2.*phi.*XX+(-1).*phi.^2.*XX+(-2).* ...

phi.*WW.*XX+2.*phi.^2.*WW.*XX+(-1).*phi.^2.*WW.^2.*XX+phi.*WW.*YY+(-1).* ...

phi.^2.*WW.*YY+(-1).*WW.^2.*YY+2.*phi.^2.*WW.^2.*YY+(-1).*phi.*WW.^3.* ...

YY+(-1).*phi.^2.*WW.^3.*YY+(-1).*phi.*XX.*YY+phi.^2.*XX.*YY+WW.*XX.*YY+( ...

-2).*phi.^2.*WW.*XX.*YY+phi.*WW.^2.*XX.*YY+phi.^2.*WW.^2.*XX.*YY+phi.* ...

WW.*ZZ+(-1).*phi.^2.*WW.*ZZ+(-1).*WW.^2.*ZZ+2.*phi.^2.*WW.^2.*ZZ+(-1).* ...

phi.*WW.^3.*ZZ+(-1).*phi.^2.*WW.^3.*ZZ+(-1).*phi.*XX.*ZZ+phi.^2.*XX.*ZZ+ ...

WW.*XX.*ZZ+(-2).*phi.^2.*WW.*XX.*ZZ+phi.*WW.^2.*XX.*ZZ+phi.^2.*WW.^2.* ...

XX.*ZZ+phi.^2.*WW.*YY.*ZZ+(-2).*phi.*WW.^2.*YY.*ZZ+(-2).*phi.^2.*WW.^2.* ...

YY.*ZZ+WW.^3.*YY.*ZZ+2.*phi.*WW.^3.*YY.*ZZ+phi.^2.*WW.^3.*YY.*ZZ+(-1).* ...

phi.^2.*XX.*YY.*ZZ+2.*phi.*WW.*XX.*YY.*ZZ+2.*phi.^2.*WW.*XX.*YY.*ZZ+(-1) ...

.*WW.^2.*XX.*YY.*ZZ+(-2).*phi.*WW.^2.*XX.*YY.*ZZ+(-1).*phi.^2.*WW.^2.* ...

XX.*YY.*ZZ).*((-3).*WW.^3+2.*phi.*WW.^3+(-1).*WW.^4+(-2).*phi.*WW.^4+3.* ...

WW.^2.*XX+(-2).*phi.*WW.^2.*XX+WW.^3.*XX+2.*phi.*WW.^3.*XX+(-1).*WW.^2.* ...

YY+2.*phi.*WW.^2.*YY+WW.^3.*YY+(-6).*phi.*WW.^3.*YY+4.*WW.^4.*YY+4.* ...

phi.*WW.^4.*YY+(-3).*WW.^3.*XX.*YY+2.*phi.*WW.^3.*XX.*YY+(-1).*WW.^4.* ...

XX.*YY+(-2).*phi.*WW.^4.*XX.*YY+WW.^2.*ZZ+(-2).*phi.*WW.^2.*ZZ+3.* ...

WW.^3.*ZZ+2.*phi.*WW.^3.*ZZ+(-4).*WW.^2.*XX.*ZZ+4.*phi.*WW.^2.*XX.*ZZ+( ...

-1).*WW.^3.*XX.*ZZ+(-6).*phi.*WW.^3.*XX.*ZZ+WW.^4.*XX.*ZZ+2.*phi.* ...

WW.^4.*XX.*ZZ+(-1).*WW.^3.*YY.*ZZ+2.*phi.*WW.^3.*YY.*ZZ+(-3).*WW.^4.* ...

YY.*ZZ+(-2).*phi.*WW.^4.*YY.*ZZ+WW.^2.*XX.*YY.*ZZ+(-2).*phi.*WW.^2.*XX.* ...

YY.*ZZ+3.*WW.^3.*XX.*YY.*ZZ+2.*phi.*WW.^3.*XX.*YY.*ZZ).^2+(-72).*(WW+( ...

-2).*phi.*WW+phi.^2.*WW+2.*phi.*WW.^2+(-2).*phi.^2.*WW.^2+phi.^2.*WW.^3+ ...

(-1).*XX+2.*phi.*XX+(-1).*phi.^2.*XX+(-2).*phi.*WW.*XX+2.*phi.^2.*WW.* ...

XX+(-1).*phi.^2.*WW.^2.*XX+phi.*WW.*YY+(-1).*phi.^2.*WW.*YY+(-1).* ...

WW.^2.*YY+2.*phi.^2.*WW.^2.*YY+(-1).*phi.*WW.^3.*YY+(-1).*phi.^2.* ...

WW.^3.*YY+(-1).*phi.*XX.*YY+phi.^2.*XX.*YY+WW.*XX.*YY+(-2).*phi.^2.*WW.* ...

XX.*YY+phi.*WW.^2.*XX.*YY+phi.^2.*WW.^2.*XX.*YY+phi.*WW.*ZZ+(-1).* ...

phi.^2.*WW.*ZZ+(-1).*WW.^2.*ZZ+2.*phi.^2.*WW.^2.*ZZ+(-1).*phi.*WW.^3.* ...

ZZ+(-1).*phi.^2.*WW.^3.*ZZ+(-1).*phi.*XX.*ZZ+phi.^2.*XX.*ZZ+WW.*XX.*ZZ+( ...

-2).*phi.^2.*WW.*XX.*ZZ+phi.*WW.^2.*XX.*ZZ+phi.^2.*WW.^2.*XX.*ZZ+ ...

phi.^2.*WW.*YY.*ZZ+(-2).*phi.*WW.^2.*YY.*ZZ+(-2).*phi.^2.*WW.^2.*YY.*ZZ+ ...

WW.^3.*YY.*ZZ+2.*phi.*WW.^3.*YY.*ZZ+phi.^2.*WW.^3.*YY.*ZZ+(-1).*phi.^2.* ...

XX.*YY.*ZZ+2.*phi.*WW.*XX.*YY.*ZZ+2.*phi.^2.*WW.*XX.*YY.*ZZ+(-1).* ...

WW.^2.*XX.*YY.*ZZ+(-2).*phi.*WW.^2.*XX.*YY.*ZZ+(-1).*phi.^2.*WW.^2.*XX.* ...

YY.*ZZ).*(WW.^4+(-1).*WW.^3.*XX+WW.^3.*YY+(-2).*WW.^4.*YY+WW.^4.*XX.*YY+ ...

(-1).*WW.^3.*ZZ+2.*WW.^3.*XX.*ZZ+(-1).*WW.^4.*XX.*ZZ+WW.^4.*YY.*ZZ+(-1) ...

.*WW.^3.*XX.*YY.*ZZ).*(3.*WW.^2+(-4).*phi.*WW.^2+phi.^2.*WW.^2+3.*WW.^3+ ...

2.*phi.*WW.^3+(-2).*phi.^2.*WW.^3+2.*phi.*WW.^4+phi.^2.*WW.^4+(-3).*WW.* ...

XX+4.*phi.*WW.*XX+(-1).*phi.^2.*WW.*XX+(-3).*WW.^2.*XX+(-2).*phi.* ...

WW.^2.*XX+2.*phi.^2.*WW.^2.*XX+(-2).*phi.*WW.^3.*XX+(-1).*phi.^2.* ...

WW.^3.*XX+(-1).*phi.*WW.*YY+phi.^2.*WW.*YY+WW.^2.*YY+2.*phi.*WW.^2.*YY+( ...

-4).*phi.^2.*WW.^2.*YY+(-5).*WW.^3.*YY+5.*phi.*WW.^3.*YY+5.*phi.^2.* ...

WW.^3.*YY+(-2).*WW.^4.*YY+(-6).*phi.*WW.^4.*YY+(-2).*phi.^2.*WW.^4.*YY+ ...

3.*WW.^2.*XX.*YY+(-5).*phi.*WW.^2.*XX.*YY+phi.^2.*WW.^2.*XX.*YY+3.* ...

WW.^3.*XX.*YY+4.*phi.*WW.^3.*XX.*YY+(-2).*phi.^2.*WW.^3.*XX.*YY+phi.* ...

WW.^4.*XX.*YY+phi.^2.*WW.^4.*XX.*YY+phi.*WW.*ZZ+(-1).*phi.^2.*WW.*ZZ+( ...

-3).*WW.^2.*ZZ+4.*phi.*WW.^2.*ZZ+2.*phi.^2.*WW.^2.*ZZ+(-3).*WW.^3.*ZZ+( ...

-5).*phi.*WW.^3.*ZZ+(-1).*phi.^2.*WW.^3.*ZZ+2.*WW.*XX.*ZZ+(-6).*phi.* ...

WW.*XX.*ZZ+2.*phi.^2.*WW.*XX.*ZZ+5.*WW.^2.*XX.*ZZ+5.*phi.*WW.^2.*XX.*ZZ+ ...

(-5).*phi.^2.*WW.^2.*XX.*ZZ+(-1).*WW.^3.*XX.*ZZ+2.*phi.*WW.^3.*XX.*ZZ+ ...

4.*phi.^2.*WW.^3.*XX.*ZZ+(-1).*phi.*WW.^4.*XX.*ZZ+(-1).*phi.^2.*WW.^4.* ...

XX.*ZZ+(-2).*phi.*WW.^2.*YY.*ZZ+phi.^2.*WW.^2.*YY.*ZZ+3.*WW.^3.*YY.*ZZ+( ...

-2).*phi.*WW.^3.*YY.*ZZ+(-2).*phi.^2.*WW.^3.*YY.*ZZ+3.*WW.^4.*YY.*ZZ+4.* ...

phi.*WW.^4.*YY.*ZZ+phi.^2.*WW.^4.*YY.*ZZ+2.*phi.*WW.*XX.*YY.*ZZ+(-1).* ...

phi.^2.*WW.*XX.*YY.*ZZ+(-3).*WW.^2.*XX.*YY.*ZZ+2.*phi.*WW.^2.*XX.*YY.* ...

ZZ+2.*phi.^2.*WW.^2.*XX.*YY.*ZZ+(-3).*WW.^3.*XX.*YY.*ZZ+(-4).*phi.* ...

WW.^3.*XX.*YY.*ZZ+(-1).*phi.^2.*WW.^3.*XX.*YY.*ZZ)+2.*(3.*WW.^2+(-4).* ...

phi.*WW.^2+phi.^2.*WW.^2+3.*WW.^3+2.*phi.*WW.^3+(-2).*phi.^2.*WW.^3+2.* ...

phi.*WW.^4+phi.^2.*WW.^4+(-3).*WW.*XX+4.*phi.*WW.*XX+(-1).*phi.^2.*WW.* ...

XX+(-3).*WW.^2.*XX+(-2).*phi.*WW.^2.*XX+2.*phi.^2.*WW.^2.*XX+(-2).*phi.* ...

WW.^3.*XX+(-1).*phi.^2.*WW.^3.*XX+(-1).*phi.*WW.*YY+phi.^2.*WW.*YY+ ...

WW.^2.*YY+2.*phi.*WW.^2.*YY+(-4).*phi.^2.*WW.^2.*YY+(-5).*WW.^3.*YY+5.* ...

phi.*WW.^3.*YY+5.*phi.^2.*WW.^3.*YY+(-2).*WW.^4.*YY+(-6).*phi.*WW.^4.* ...

YY+(-2).*phi.^2.*WW.^4.*YY+3.*WW.^2.*XX.*YY+(-5).*phi.*WW.^2.*XX.*YY+ ...

phi.^2.*WW.^2.*XX.*YY+3.*WW.^3.*XX.*YY+4.*phi.*WW.^3.*XX.*YY+(-2).* ...

phi.^2.*WW.^3.*XX.*YY+phi.*WW.^4.*XX.*YY+phi.^2.*WW.^4.*XX.*YY+phi.*WW.* ...

ZZ+(-1).*phi.^2.*WW.*ZZ+(-3).*WW.^2.*ZZ+4.*phi.*WW.^2.*ZZ+2.*phi.^2.* ...

WW.^2.*ZZ+(-3).*WW.^3.*ZZ+(-5).*phi.*WW.^3.*ZZ+(-1).*phi.^2.*WW.^3.*ZZ+ ...

2.*WW.*XX.*ZZ+(-6).*phi.*WW.*XX.*ZZ+2.*phi.^2.*WW.*XX.*ZZ+5.*WW.^2.*XX.* ...

ZZ+5.*phi.*WW.^2.*XX.*ZZ+(-5).*phi.^2.*WW.^2.*XX.*ZZ+(-1).*WW.^3.*XX.* ...

ZZ+2.*phi.*WW.^3.*XX.*ZZ+4.*phi.^2.*WW.^3.*XX.*ZZ+(-1).*phi.*WW.^4.*XX.* ...

ZZ+(-1).*phi.^2.*WW.^4.*XX.*ZZ+(-2).*phi.*WW.^2.*YY.*ZZ+phi.^2.*WW.^2.* ...

YY.*ZZ+3.*WW.^3.*YY.*ZZ+(-2).*phi.*WW.^3.*YY.*ZZ+(-2).*phi.^2.*WW.^3.* ...

YY.*ZZ+3.*WW.^4.*YY.*ZZ+4.*phi.*WW.^4.*YY.*ZZ+phi.^2.*WW.^4.*YY.*ZZ+2.* ...

phi.*WW.*XX.*YY.*ZZ+(-1).*phi.^2.*WW.*XX.*YY.*ZZ+(-3).*WW.^2.*XX.*YY.* ...

ZZ+2.*phi.*WW.^2.*XX.*YY.*ZZ+2.*phi.^2.*WW.^2.*XX.*YY.*ZZ+(-3).*WW.^3.* ...

XX.*YY.*ZZ+(-4).*phi.*WW.^3.*XX.*YY.*ZZ+(-1).*phi.^2.*WW.^3.*XX.*YY.*ZZ) ...

.^3+(-9).*((-3).*WW.^3+2.*phi.*WW.^3+(-1).*WW.^4+(-2).*phi.*WW.^4+3.* ...

WW.^2.*XX+(-2).*phi.*WW.^2.*XX+WW.^3.*XX+2.*phi.*WW.^3.*XX+(-1).*WW.^2.* ...

YY+2.*phi.*WW.^2.*YY+WW.^3.*YY+(-6).*phi.*WW.^3.*YY+4.*WW.^4.*YY+4.* ...

phi.*WW.^4.*YY+(-3).*WW.^3.*XX.*YY+2.*phi.*WW.^3.*XX.*YY+(-1).*WW.^4.* ...

XX.*YY+(-2).*phi.*WW.^4.*XX.*YY+WW.^2.*ZZ+(-2).*phi.*WW.^2.*ZZ+3.* ...

WW.^3.*ZZ+2.*phi.*WW.^3.*ZZ+(-4).*WW.^2.*XX.*ZZ+4.*phi.*WW.^2.*XX.*ZZ+( ...

-1).*WW.^3.*XX.*ZZ+(-6).*phi.*WW.^3.*XX.*ZZ+WW.^4.*XX.*ZZ+2.*phi.* ...

WW.^4.*XX.*ZZ+(-1).*WW.^3.*YY.*ZZ+2.*phi.*WW.^3.*YY.*ZZ+(-3).*WW.^4.* ...

YY.*ZZ+(-2).*phi.*WW.^4.*YY.*ZZ+WW.^2.*XX.*YY.*ZZ+(-2).*phi.*WW.^2.*XX.* ...

YY.*ZZ+3.*WW.^3.*XX.*YY.*ZZ+2.*phi.*WW.^3.*XX.*YY.*ZZ).*(3.*WW.^2+(-4).* ...

phi.*WW.^2+phi.^2.*WW.^2+3.*WW.^3+2.*phi.*WW.^3+(-2).*phi.^2.*WW.^3+2.* ...

phi.*WW.^4+phi.^2.*WW.^4+(-3).*WW.*XX+4.*phi.*WW.*XX+(-1).*phi.^2.*WW.* ...

XX+(-3).*WW.^2.*XX+(-2).*phi.*WW.^2.*XX+2.*phi.^2.*WW.^2.*XX+(-2).*phi.* ...

WW.^3.*XX+(-1).*phi.^2.*WW.^3.*XX+(-1).*phi.*WW.*YY+phi.^2.*WW.*YY+ ...

WW.^2.*YY+2.*phi.*WW.^2.*YY+(-4).*phi.^2.*WW.^2.*YY+(-5).*WW.^3.*YY+5.* ...

phi.*WW.^3.*YY+5.*phi.^2.*WW.^3.*YY+(-2).*WW.^4.*YY+(-6).*phi.*WW.^4.* ...

YY+(-2).*phi.^2.*WW.^4.*YY+3.*WW.^2.*XX.*YY+(-5).*phi.*WW.^2.*XX.*YY+ ...

phi.^2.*WW.^2.*XX.*YY+3.*WW.^3.*XX.*YY+4.*phi.*WW.^3.*XX.*YY+(-2).* ...

phi.^2.*WW.^3.*XX.*YY+phi.*WW.^4.*XX.*YY+phi.^2.*WW.^4.*XX.*YY+phi.*WW.* ...

ZZ+(-1).*phi.^2.*WW.*ZZ+(-3).*WW.^2.*ZZ+4.*phi.*WW.^2.*ZZ+2.*phi.^2.* ...

WW.^2.*ZZ+(-3).*WW.^3.*ZZ+(-5).*phi.*WW.^3.*ZZ+(-1).*phi.^2.*WW.^3.*ZZ+ ...

2.*WW.*XX.*ZZ+(-6).*phi.*WW.*XX.*ZZ+2.*phi.^2.*WW.*XX.*ZZ+5.*WW.^2.*XX.* ...

ZZ+5.*phi.*WW.^2.*XX.*ZZ+(-5).*phi.^2.*WW.^2.*XX.*ZZ+(-1).*WW.^3.*XX.* ...

ZZ+2.*phi.*WW.^3.*XX.*ZZ+4.*phi.^2.*WW.^3.*XX.*ZZ+(-1).*phi.*WW.^4.*XX.* ...

ZZ+(-1).*phi.^2.*WW.^4.*XX.*ZZ+(-2).*phi.*WW.^2.*YY.*ZZ+phi.^2.*WW.^2.* ...

YY.*ZZ+3.*WW.^3.*YY.*ZZ+(-2).*phi.*WW.^3.*YY.*ZZ+(-2).*phi.^2.*WW.^3.* ...

YY.*ZZ+3.*WW.^4.*YY.*ZZ+4.*phi.*WW.^4.*YY.*ZZ+phi.^2.*WW.^4.*YY.*ZZ+2.* ...

phi.*WW.*XX.*YY.*ZZ+(-1).*phi.^2.*WW.*XX.*YY.*ZZ+(-3).*WW.^2.*XX.*YY.* ...

ZZ+2.*phi.*WW.^2.*XX.*YY.*ZZ+2.*phi.^2.*WW.^2.*XX.*YY.*ZZ+(-3).*WW.^3.* ...

XX.*YY.*ZZ+(-4).*phi.*WW.^3.*XX.*YY.*ZZ+(-1).*phi.^2.*WW.^3.*XX.*YY.*ZZ) ...

.*((-1).*WW+2.*phi.*WW+(-1).*phi.^2.*WW+(-3).*WW.^2+2.*phi.*WW.^2+ ...

phi.^2.*WW.^2+(-4).*phi.*WW.^3+phi.^2.*WW.^3+(-1).*phi.^2.*WW.^4+XX+(-2) ...

.*phi.*XX+phi.^2.*XX+3.*WW.*XX+(-2).*phi.*WW.*XX+(-1).*phi.^2.*WW.*XX+ ...

4.*phi.*WW.^2.*XX+(-1).*phi.^2.*WW.^2.*XX+phi.^2.*WW.^3.*XX+WW.^2.*YY+( ...

-4).*phi.*WW.^2.*YY+2.*phi.^2.*WW.^2.*YY+3.*WW.^3.*YY+2.*phi.*WW.^3.*YY+ ...

(-4).*phi.^2.*WW.^3.*YY+2.*phi.*WW.^4.*YY+2.*phi.^2.*WW.^4.*YY+(-1).* ...

WW.*XX.*YY+4.*phi.*WW.*XX.*YY+(-2).*phi.^2.*WW.*XX.*YY+(-3).*WW.^2.*XX.* ...

YY+(-2).*phi.*WW.^2.*XX.*YY+4.*phi.^2.*WW.^2.*XX.*YY+(-2).*phi.*WW.^3.* ...

XX.*YY+(-2).*phi.^2.*WW.^3.*XX.*YY+(-2).*phi.*WW.*ZZ+2.*phi.^2.*WW.*ZZ+ ...

3.*WW.^2.*ZZ+(-2).*phi.*WW.^2.*ZZ+(-4).*phi.^2.*WW.^2.*ZZ+WW.^3.*ZZ+4.* ...

phi.*WW.^3.*ZZ+2.*phi.^2.*WW.^3.*ZZ+2.*phi.*XX.*ZZ+(-2).*phi.^2.*XX.*ZZ+ ...

(-3).*WW.*XX.*ZZ+2.*phi.*WW.*XX.*ZZ+4.*phi.^2.*WW.*XX.*ZZ+(-1).*WW.^2.* ...

XX.*ZZ+(-4).*phi.*WW.^2.*XX.*ZZ+(-2).*phi.^2.*WW.^2.*XX.*ZZ+(-1).* ...

phi.^2.*WW.*YY.*ZZ+4.*phi.*WW.^2.*YY.*ZZ+phi.^2.*WW.^2.*YY.*ZZ+(-3).* ...

WW.^3.*YY.*ZZ+(-2).*phi.*WW.^3.*YY.*ZZ+phi.^2.*WW.^3.*YY.*ZZ+(-1).* ...

WW.^4.*YY.*ZZ+(-2).*phi.*WW.^4.*YY.*ZZ+(-1).*phi.^2.*WW.^4.*YY.*ZZ+ ...

phi.^2.*XX.*YY.*ZZ+(-4).*phi.*WW.*XX.*YY.*ZZ+(-1).*phi.^2.*WW.*XX.*YY.* ...

ZZ+3.*WW.^2.*XX.*YY.*ZZ+2.*phi.*WW.^2.*XX.*YY.*ZZ+(-1).*phi.^2.*WW.^2.* ...

XX.*YY.*ZZ+WW.^3.*XX.*YY.*ZZ+2.*phi.*WW.^3.*XX.*YY.*ZZ+phi.^2.*WW.^3.* ...

XX.*YY.*ZZ)+27.*(WW.^4+(-1).*WW.^3.*XX+WW.^3.*YY+(-2).*WW.^4.*YY+WW.^4.* ...

XX.*YY+(-1).*WW.^3.*ZZ+2.*WW.^3.*XX.*ZZ+(-1).*WW.^4.*XX.*ZZ+WW.^4.*YY.* ...

ZZ+(-1).*WW.^3.*XX.*YY.*ZZ).*((-1).*WW+2.*phi.*WW+(-1).*phi.^2.*WW+(-3) ...

.*WW.^2+2.*phi.*WW.^2+phi.^2.*WW.^2+(-4).*phi.*WW.^3+phi.^2.*WW.^3+(-1) ...

.*phi.^2.*WW.^4+XX+(-2).*phi.*XX+phi.^2.*XX+3.*WW.*XX+(-2).*phi.*WW.*XX+ ...

(-1).*phi.^2.*WW.*XX+4.*phi.*WW.^2.*XX+(-1).*phi.^2.*WW.^2.*XX+phi.^2.* ...

WW.^3.*XX+WW.^2.*YY+(-4).*phi.*WW.^2.*YY+2.*phi.^2.*WW.^2.*YY+3.*WW.^3.* ...

YY+2.*phi.*WW.^3.*YY+(-4).*phi.^2.*WW.^3.*YY+2.*phi.*WW.^4.*YY+2.* ...

phi.^2.*WW.^4.*YY+(-1).*WW.*XX.*YY+4.*phi.*WW.*XX.*YY+(-2).*phi.^2.*WW.* ...

XX.*YY+(-3).*WW.^2.*XX.*YY+(-2).*phi.*WW.^2.*XX.*YY+4.*phi.^2.*WW.^2.* ...

XX.*YY+(-2).*phi.*WW.^3.*XX.*YY+(-2).*phi.^2.*WW.^3.*XX.*YY+(-2).*phi.* ...

WW.*ZZ+2.*phi.^2.*WW.*ZZ+3.*WW.^2.*ZZ+(-2).*phi.*WW.^2.*ZZ+(-4).* ...

phi.^2.*WW.^2.*ZZ+WW.^3.*ZZ+4.*phi.*WW.^3.*ZZ+2.*phi.^2.*WW.^3.*ZZ+2.* ...

phi.*XX.*ZZ+(-2).*phi.^2.*XX.*ZZ+(-3).*WW.*XX.*ZZ+2.*phi.*WW.*XX.*ZZ+4.* ...

phi.^2.*WW.*XX.*ZZ+(-1).*WW.^2.*XX.*ZZ+(-4).*phi.*WW.^2.*XX.*ZZ+(-2).* ...

phi.^2.*WW.^2.*XX.*ZZ+(-1).*phi.^2.*WW.*YY.*ZZ+4.*phi.*WW.^2.*YY.*ZZ+ ...

phi.^2.*WW.^2.*YY.*ZZ+(-3).*WW.^3.*YY.*ZZ+(-2).*phi.*WW.^3.*YY.*ZZ+ ...

phi.^2.*WW.^3.*YY.*ZZ+(-1).*WW.^4.*YY.*ZZ+(-2).*phi.*WW.^4.*YY.*ZZ+(-1) ...

.*phi.^2.*WW.^4.*YY.*ZZ+phi.^2.*XX.*YY.*ZZ+(-4).*phi.*WW.*XX.*YY.*ZZ+( ...

-1).*phi.^2.*WW.*XX.*YY.*ZZ+3.*WW.^2.*XX.*YY.*ZZ+2.*phi.*WW.^2.*XX.*YY.* ...

ZZ+(-1).*phi.^2.*WW.^2.*XX.*YY.*ZZ+WW.^3.*XX.*YY.*ZZ+2.*phi.*WW.^3.*XX.* ...

YY.*ZZ+phi.^2.*WW.^3.*XX.*YY.*ZZ).^2).^2).^(1/2)).^(1/3)).^(1/2)+(-1/2) ...

.*((1/2).*((-1)+phi+(-1).*phi.*WW+(-1).*phi.*YY+WW.*YY+phi.*WW.*YY).^( ...

-2).*((-1)+phi+(-1).*phi.*WW+(-1).*phi.*ZZ+WW.*ZZ+phi.*WW.*ZZ).^(-2).*(( ...

-1)+2.*phi+(-1).*phi.^2+(-3).*WW+2.*phi.*WW+phi.^2.*WW+(-4).*phi.*WW.^2+ ...

phi.^2.*WW.^2+(-1).*phi.^2.*WW.^3+WW.*YY+(-4).*phi.*WW.*YY+2.*phi.^2.* ...

WW.*YY+3.*WW.^2.*YY+2.*phi.*WW.^2.*YY+(-4).*phi.^2.*WW.^2.*YY+2.*phi.* ...

WW.^3.*YY+2.*phi.^2.*WW.^3.*YY+(-2).*phi.*ZZ+2.*phi.^2.*ZZ+3.*WW.*ZZ+( ...

-2).*phi.*WW.*ZZ+(-4).*phi.^2.*WW.*ZZ+WW.^2.*ZZ+4.*phi.*WW.^2.*ZZ+2.* ...

phi.^2.*WW.^2.*ZZ+(-1).*phi.^2.*YY.*ZZ+4.*phi.*WW.*YY.*ZZ+phi.^2.*WW.* ...

YY.*ZZ+(-3).*WW.^2.*YY.*ZZ+(-2).*phi.*WW.^2.*YY.*ZZ+phi.^2.*WW.^2.*YY.* ...

ZZ+(-1).*WW.^3.*YY.*ZZ+(-2).*phi.*WW.^3.*YY.*ZZ+(-1).*phi.^2.*WW.^3.* ...

YY.*ZZ).^2+(-1).*(WW+(-1).*XX).^(-1).*((-1)+phi+(-1).*phi.*WW+(-1).* ...

phi.*YY+WW.*YY+phi.*WW.*YY).^(-1).*((-1)+phi+(-1).*phi.*WW+(-1).*phi.* ...

ZZ+WW.*ZZ+phi.*WW.*ZZ).^(-1).*(3.*WW.^2+(-4).*phi.*WW.^2+phi.^2.*WW.^2+ ...

3.*WW.^3+2.*phi.*WW.^3+(-2).*phi.^2.*WW.^3+2.*phi.*WW.^4+phi.^2.*WW.^4+( ...

-3).*WW.*XX+4.*phi.*WW.*XX+(-1).*phi.^2.*WW.*XX+(-3).*WW.^2.*XX+(-2).* ...

phi.*WW.^2.*XX+2.*phi.^2.*WW.^2.*XX+(-2).*phi.*WW.^3.*XX+(-1).*phi.^2.* ...

WW.^3.*XX+(-1).*phi.*WW.*YY+phi.^2.*WW.*YY+WW.^2.*YY+2.*phi.*WW.^2.*YY+( ...

-4).*phi.^2.*WW.^2.*YY+(-5).*WW.^3.*YY+5.*phi.*WW.^3.*YY+5.*phi.^2.* ...

WW.^3.*YY+(-2).*WW.^4.*YY+(-6).*phi.*WW.^4.*YY+(-2).*phi.^2.*WW.^4.*YY+ ...

3.*WW.^2.*XX.*YY+(-5).*phi.*WW.^2.*XX.*YY+phi.^2.*WW.^2.*XX.*YY+3.* ...

WW.^3.*XX.*YY+4.*phi.*WW.^3.*XX.*YY+(-2).*phi.^2.*WW.^3.*XX.*YY+phi.* ...

WW.^4.*XX.*YY+phi.^2.*WW.^4.*XX.*YY+phi.*WW.*ZZ+(-1).*phi.^2.*WW.*ZZ+( ...

-3).*WW.^2.*ZZ+4.*phi.*WW.^2.*ZZ+2.*phi.^2.*WW.^2.*ZZ+(-3).*WW.^3.*ZZ+( ...

-5).*phi.*WW.^3.*ZZ+(-1).*phi.^2.*WW.^3.*ZZ+2.*WW.*XX.*ZZ+(-6).*phi.* ...

WW.*XX.*ZZ+2.*phi.^2.*WW.*XX.*ZZ+5.*WW.^2.*XX.*ZZ+5.*phi.*WW.^2.*XX.*ZZ+ ...

(-5).*phi.^2.*WW.^2.*XX.*ZZ+(-1).*WW.^3.*XX.*ZZ+2.*phi.*WW.^3.*XX.*ZZ+ ...

4.*phi.^2.*WW.^3.*XX.*ZZ+(-1).*phi.*WW.^4.*XX.*ZZ+(-1).*phi.^2.*WW.^4.* ...

XX.*ZZ+(-2).*phi.*WW.^2.*YY.*ZZ+phi.^2.*WW.^2.*YY.*ZZ+3.*WW.^3.*YY.*ZZ+( ...

-2).*phi.*WW.^3.*YY.*ZZ+(-2).*phi.^2.*WW.^3.*YY.*ZZ+3.*WW.^4.*YY.*ZZ+4.* ...

phi.*WW.^4.*YY.*ZZ+phi.^2.*WW.^4.*YY.*ZZ+2.*phi.*WW.*XX.*YY.*ZZ+(-1).* ...

phi.^2.*WW.*XX.*YY.*ZZ+(-3).*WW.^2.*XX.*YY.*ZZ+2.*phi.*WW.^2.*XX.*YY.* ...

ZZ+2.*phi.^2.*WW.^2.*XX.*YY.*ZZ+(-3).*WW.^3.*XX.*YY.*ZZ+(-4).*phi.* ...

WW.^3.*XX.*YY.*ZZ+(-1).*phi.^2.*WW.^3.*XX.*YY.*ZZ)+(-1/3).*(WW+(-2).* ...

phi.*WW+phi.^2.*WW+2.*phi.*WW.^2+(-2).*phi.^2.*WW.^2+phi.^2.*WW.^3+(-1) ...

.*XX+2.*phi.*XX+(-1).*phi.^2.*XX+(-2).*phi.*WW.*XX+2.*phi.^2.*WW.*XX+( ...

-1).*phi.^2.*WW.^2.*XX+phi.*WW.*YY+(-1).*phi.^2.*WW.*YY+(-1).*WW.^2.*YY+ ...

2.*phi.^2.*WW.^2.*YY+(-1).*phi.*WW.^3.*YY+(-1).*phi.^2.*WW.^3.*YY+(-1).* ...

phi.*XX.*YY+phi.^2.*XX.*YY+WW.*XX.*YY+(-2).*phi.^2.*WW.*XX.*YY+phi.* ...

WW.^2.*XX.*YY+phi.^2.*WW.^2.*XX.*YY+phi.*WW.*ZZ+(-1).*phi.^2.*WW.*ZZ+( ...

-1).*WW.^2.*ZZ+2.*phi.^2.*WW.^2.*ZZ+(-1).*phi.*WW.^3.*ZZ+(-1).*phi.^2.* ...

WW.^3.*ZZ+(-1).*phi.*XX.*ZZ+phi.^2.*XX.*ZZ+WW.*XX.*ZZ+(-2).*phi.^2.*WW.* ...

XX.*ZZ+phi.*WW.^2.*XX.*ZZ+phi.^2.*WW.^2.*XX.*ZZ+phi.^2.*WW.*YY.*ZZ+(-2) ...

.*phi.*WW.^2.*YY.*ZZ+(-2).*phi.^2.*WW.^2.*YY.*ZZ+WW.^3.*YY.*ZZ+2.*phi.* ...

WW.^3.*YY.*ZZ+phi.^2.*WW.^3.*YY.*ZZ+(-1).*phi.^2.*XX.*YY.*ZZ+2.*phi.* ...

WW.*XX.*YY.*ZZ+2.*phi.^2.*WW.*XX.*YY.*ZZ+(-1).*WW.^2.*XX.*YY.*ZZ+(-2).* ...

phi.*WW.^2.*XX.*YY.*ZZ+(-1).*phi.^2.*WW.^2.*XX.*YY.*ZZ).^(-1).*(3.* ...

WW.^2+(-4).*phi.*WW.^2+phi.^2.*WW.^2+3.*WW.^3+2.*phi.*WW.^3+(-2).* ...

phi.^2.*WW.^3+2.*phi.*WW.^4+phi.^2.*WW.^4+(-3).*WW.*XX+4.*phi.*WW.*XX+( ...

-1).*phi.^2.*WW.*XX+(-3).*WW.^2.*XX+(-2).*phi.*WW.^2.*XX+2.*phi.^2.* ...

WW.^2.*XX+(-2).*phi.*WW.^3.*XX+(-1).*phi.^2.*WW.^3.*XX+(-1).*phi.*WW.* ...

YY+phi.^2.*WW.*YY+WW.^2.*YY+2.*phi.*WW.^2.*YY+(-4).*phi.^2.*WW.^2.*YY+( ...

-5).*WW.^3.*YY+5.*phi.*WW.^3.*YY+5.*phi.^2.*WW.^3.*YY+(-2).*WW.^4.*YY+( ...

-6).*phi.*WW.^4.*YY+(-2).*phi.^2.*WW.^4.*YY+3.*WW.^2.*XX.*YY+(-5).*phi.* ...

WW.^2.*XX.*YY+phi.^2.*WW.^2.*XX.*YY+3.*WW.^3.*XX.*YY+4.*phi.*WW.^3.*XX.* ...

YY+(-2).*phi.^2.*WW.^3.*XX.*YY+phi.*WW.^4.*XX.*YY+phi.^2.*WW.^4.*XX.*YY+ ...

phi.*WW.*ZZ+(-1).*phi.^2.*WW.*ZZ+(-3).*WW.^2.*ZZ+4.*phi.*WW.^2.*ZZ+2.* ...

phi.^2.*WW.^2.*ZZ+(-3).*WW.^3.*ZZ+(-5).*phi.*WW.^3.*ZZ+(-1).*phi.^2.* ...

WW.^3.*ZZ+2.*WW.*XX.*ZZ+(-6).*phi.*WW.*XX.*ZZ+2.*phi.^2.*WW.*XX.*ZZ+5.* ...

WW.^2.*XX.*ZZ+5.*phi.*WW.^2.*XX.*ZZ+(-5).*phi.^2.*WW.^2.*XX.*ZZ+(-1).* ...

WW.^3.*XX.*ZZ+2.*phi.*WW.^3.*XX.*ZZ+4.*phi.^2.*WW.^3.*XX.*ZZ+(-1).*phi.* ...

WW.^4.*XX.*ZZ+(-1).*phi.^2.*WW.^4.*XX.*ZZ+(-2).*phi.*WW.^2.*YY.*ZZ+ ...

phi.^2.*WW.^2.*YY.*ZZ+3.*WW.^3.*YY.*ZZ+(-2).*phi.*WW.^3.*YY.*ZZ+(-2).* ...

phi.^2.*WW.^3.*YY.*ZZ+3.*WW.^4.*YY.*ZZ+4.*phi.*WW.^4.*YY.*ZZ+phi.^2.* ...

WW.^4.*YY.*ZZ+2.*phi.*WW.*XX.*YY.*ZZ+(-1).*phi.^2.*WW.*XX.*YY.*ZZ+(-3).* ...

WW.^2.*XX.*YY.*ZZ+2.*phi.*WW.^2.*XX.*YY.*ZZ+2.*phi.^2.*WW.^2.*XX.*YY.* ...

ZZ+(-3).*WW.^3.*XX.*YY.*ZZ+(-4).*phi.*WW.^3.*XX.*YY.*ZZ+(-1).*phi.^2.* ...

WW.^3.*XX.*YY.*ZZ)+(-1/3).*2.^(1/3).*(WW+(-1).*XX).^(-1).*((-1)+phi+(-1) ...

.*phi.*WW+(-1).*phi.*YY+WW.*YY+phi.*WW.*YY).^(-1).*((-1)+phi+(-1).*phi.* ...

WW+(-1).*phi.*ZZ+WW.*ZZ+phi.*WW.*ZZ).^(-1).*(12.*(WW+(-2).*phi.*WW+ ...

phi.^2.*WW+2.*phi.*WW.^2+(-2).*phi.^2.*WW.^2+phi.^2.*WW.^3+(-1).*XX+2.* ...

phi.*XX+(-1).*phi.^2.*XX+(-2).*phi.*WW.*XX+2.*phi.^2.*WW.*XX+(-1).* ...

phi.^2.*WW.^2.*XX+phi.*WW.*YY+(-1).*phi.^2.*WW.*YY+(-1).*WW.^2.*YY+2.* ...

phi.^2.*WW.^2.*YY+(-1).*phi.*WW.^3.*YY+(-1).*phi.^2.*WW.^3.*YY+(-1).* ...

phi.*XX.*YY+phi.^2.*XX.*YY+WW.*XX.*YY+(-2).*phi.^2.*WW.*XX.*YY+phi.* ...

WW.^2.*XX.*YY+phi.^2.*WW.^2.*XX.*YY+phi.*WW.*ZZ+(-1).*phi.^2.*WW.*ZZ+( ...

-1).*WW.^2.*ZZ+2.*phi.^2.*WW.^2.*ZZ+(-1).*phi.*WW.^3.*ZZ+(-1).*phi.^2.* ...

WW.^3.*ZZ+(-1).*phi.*XX.*ZZ+phi.^2.*XX.*ZZ+WW.*XX.*ZZ+(-2).*phi.^2.*WW.* ...

XX.*ZZ+phi.*WW.^2.*XX.*ZZ+phi.^2.*WW.^2.*XX.*ZZ+phi.^2.*WW.*YY.*ZZ+(-2) ...

.*phi.*WW.^2.*YY.*ZZ+(-2).*phi.^2.*WW.^2.*YY.*ZZ+WW.^3.*YY.*ZZ+2.*phi.* ...

WW.^3.*YY.*ZZ+phi.^2.*WW.^3.*YY.*ZZ+(-1).*phi.^2.*XX.*YY.*ZZ+2.*phi.* ...

WW.*XX.*YY.*ZZ+2.*phi.^2.*WW.*XX.*YY.*ZZ+(-1).*WW.^2.*XX.*YY.*ZZ+(-2).* ...

phi.*WW.^2.*XX.*YY.*ZZ+(-1).*phi.^2.*WW.^2.*XX.*YY.*ZZ).*(WW.^4+(-1).* ...

WW.^3.*XX+WW.^3.*YY+(-2).*WW.^4.*YY+WW.^4.*XX.*YY+(-1).*WW.^3.*ZZ+2.* ...

WW.^3.*XX.*ZZ+(-1).*WW.^4.*XX.*ZZ+WW.^4.*YY.*ZZ+(-1).*WW.^3.*XX.*YY.*ZZ) ...

+(3.*WW.^2+(-4).*phi.*WW.^2+phi.^2.*WW.^2+3.*WW.^3+2.*phi.*WW.^3+(-2).* ...

phi.^2.*WW.^3+2.*phi.*WW.^4+phi.^2.*WW.^4+(-3).*WW.*XX+4.*phi.*WW.*XX+( ...

-1).*phi.^2.*WW.*XX+(-3).*WW.^2.*XX+(-2).*phi.*WW.^2.*XX+2.*phi.^2.* ...

WW.^2.*XX+(-2).*phi.*WW.^3.*XX+(-1).*phi.^2.*WW.^3.*XX+(-1).*phi.*WW.* ...

YY+phi.^2.*WW.*YY+WW.^2.*YY+2.*phi.*WW.^2.*YY+(-4).*phi.^2.*WW.^2.*YY+( ...

-5).*WW.^3.*YY+5.*phi.*WW.^3.*YY+5.*phi.^2.*WW.^3.*YY+(-2).*WW.^4.*YY+( ...

-6).*phi.*WW.^4.*YY+(-2).*phi.^2.*WW.^4.*YY+3.*WW.^2.*XX.*YY+(-5).*phi.* ...

WW.^2.*XX.*YY+phi.^2.*WW.^2.*XX.*YY+3.*WW.^3.*XX.*YY+4.*phi.*WW.^3.*XX.* ...

YY+(-2).*phi.^2.*WW.^3.*XX.*YY+phi.*WW.^4.*XX.*YY+phi.^2.*WW.^4.*XX.*YY+ ...

phi.*WW.*ZZ+(-1).*phi.^2.*WW.*ZZ+(-3).*WW.^2.*ZZ+4.*phi.*WW.^2.*ZZ+2.* ...

phi.^2.*WW.^2.*ZZ+(-3).*WW.^3.*ZZ+(-5).*phi.*WW.^3.*ZZ+(-1).*phi.^2.* ...

WW.^3.*ZZ+2.*WW.*XX.*ZZ+(-6).*phi.*WW.*XX.*ZZ+2.*phi.^2.*WW.*XX.*ZZ+5.* ...

WW.^2.*XX.*ZZ+5.*phi.*WW.^2.*XX.*ZZ+(-5).*phi.^2.*WW.^2.*XX.*ZZ+(-1).* ...

WW.^3.*XX.*ZZ+2.*phi.*WW.^3.*XX.*ZZ+4.*phi.^2.*WW.^3.*XX.*ZZ+(-1).*phi.* ...

WW.^4.*XX.*ZZ+(-1).*phi.^2.*WW.^4.*XX.*ZZ+(-2).*phi.*WW.^2.*YY.*ZZ+ ...

phi.^2.*WW.^2.*YY.*ZZ+3.*WW.^3.*YY.*ZZ+(-2).*phi.*WW.^3.*YY.*ZZ+(-2).* ...

phi.^2.*WW.^3.*YY.*ZZ+3.*WW.^4.*YY.*ZZ+4.*phi.*WW.^4.*YY.*ZZ+phi.^2.* ...

WW.^4.*YY.*ZZ+2.*phi.*WW.*XX.*YY.*ZZ+(-1).*phi.^2.*WW.*XX.*YY.*ZZ+(-3).* ...

WW.^2.*XX.*YY.*ZZ+2.*phi.*WW.^2.*XX.*YY.*ZZ+2.*phi.^2.*WW.^2.*XX.*YY.* ...

ZZ+(-3).*WW.^3.*XX.*YY.*ZZ+(-4).*phi.*WW.^3.*XX.*YY.*ZZ+(-1).*phi.^2.* ...

WW.^3.*XX.*YY.*ZZ).^2+(-3).*((-3).*WW.^3+2.*phi.*WW.^3+(-1).*WW.^4+(-2) ...

.*phi.*WW.^4+3.*WW.^2.*XX+(-2).*phi.*WW.^2.*XX+WW.^3.*XX+2.*phi.*WW.^3.* ...

XX+(-1).*WW.^2.*YY+2.*phi.*WW.^2.*YY+WW.^3.*YY+(-6).*phi.*WW.^3.*YY+4.* ...

WW.^4.*YY+4.*phi.*WW.^4.*YY+(-3).*WW.^3.*XX.*YY+2.*phi.*WW.^3.*XX.*YY+( ...

-1).*WW.^4.*XX.*YY+(-2).*phi.*WW.^4.*XX.*YY+WW.^2.*ZZ+(-2).*phi.*WW.^2.* ...

ZZ+3.*WW.^3.*ZZ+2.*phi.*WW.^3.*ZZ+(-4).*WW.^2.*XX.*ZZ+4.*phi.*WW.^2.* ...

XX.*ZZ+(-1).*WW.^3.*XX.*ZZ+(-6).*phi.*WW.^3.*XX.*ZZ+WW.^4.*XX.*ZZ+2.* ...

phi.*WW.^4.*XX.*ZZ+(-1).*WW.^3.*YY.*ZZ+2.*phi.*WW.^3.*YY.*ZZ+(-3).* ...

WW.^4.*YY.*ZZ+(-2).*phi.*WW.^4.*YY.*ZZ+WW.^2.*XX.*YY.*ZZ+(-2).*phi.* ...

WW.^2.*XX.*YY.*ZZ+3.*WW.^3.*XX.*YY.*ZZ+2.*phi.*WW.^3.*XX.*YY.*ZZ).*((-1) ...

.*WW+2.*phi.*WW+(-1).*phi.^2.*WW+(-3).*WW.^2+2.*phi.*WW.^2+phi.^2.* ...

WW.^2+(-4).*phi.*WW.^3+phi.^2.*WW.^3+(-1).*phi.^2.*WW.^4+XX+(-2).*phi.* ...

XX+phi.^2.*XX+3.*WW.*XX+(-2).*phi.*WW.*XX+(-1).*phi.^2.*WW.*XX+4.*phi.* ...

WW.^2.*XX+(-1).*phi.^2.*WW.^2.*XX+phi.^2.*WW.^3.*XX+WW.^2.*YY+(-4).* ...

phi.*WW.^2.*YY+2.*phi.^2.*WW.^2.*YY+3.*WW.^3.*YY+2.*phi.*WW.^3.*YY+(-4) ...

.*phi.^2.*WW.^3.*YY+2.*phi.*WW.^4.*YY+2.*phi.^2.*WW.^4.*YY+(-1).*WW.* ...

XX.*YY+4.*phi.*WW.*XX.*YY+(-2).*phi.^2.*WW.*XX.*YY+(-3).*WW.^2.*XX.*YY+( ...

-2).*phi.*WW.^2.*XX.*YY+4.*phi.^2.*WW.^2.*XX.*YY+(-2).*phi.*WW.^3.*XX.* ...

YY+(-2).*phi.^2.*WW.^3.*XX.*YY+(-2).*phi.*WW.*ZZ+2.*phi.^2.*WW.*ZZ+3.* ...

WW.^2.*ZZ+(-2).*phi.*WW.^2.*ZZ+(-4).*phi.^2.*WW.^2.*ZZ+WW.^3.*ZZ+4.* ...

phi.*WW.^3.*ZZ+2.*phi.^2.*WW.^3.*ZZ+2.*phi.*XX.*ZZ+(-2).*phi.^2.*XX.*ZZ+ ...

(-3).*WW.*XX.*ZZ+2.*phi.*WW.*XX.*ZZ+4.*phi.^2.*WW.*XX.*ZZ+(-1).*WW.^2.* ...

XX.*ZZ+(-4).*phi.*WW.^2.*XX.*ZZ+(-2).*phi.^2.*WW.^2.*XX.*ZZ+(-1).* ...

phi.^2.*WW.*YY.*ZZ+4.*phi.*WW.^2.*YY.*ZZ+phi.^2.*WW.^2.*YY.*ZZ+(-3).* ...

WW.^3.*YY.*ZZ+(-2).*phi.*WW.^3.*YY.*ZZ+phi.^2.*WW.^3.*YY.*ZZ+(-1).* ...

WW.^4.*YY.*ZZ+(-2).*phi.*WW.^4.*YY.*ZZ+(-1).*phi.^2.*WW.^4.*YY.*ZZ+ ...

phi.^2.*XX.*YY.*ZZ+(-4).*phi.*WW.*XX.*YY.*ZZ+(-1).*phi.^2.*WW.*XX.*YY.* ...

ZZ+3.*WW.^2.*XX.*YY.*ZZ+2.*phi.*WW.^2.*XX.*YY.*ZZ+(-1).*phi.^2.*WW.^2.* ...

XX.*YY.*ZZ+WW.^3.*XX.*YY.*ZZ+2.*phi.*WW.^3.*XX.*YY.*ZZ+phi.^2.*WW.^3.* ...

XX.*YY.*ZZ)).*(27.*(WW+(-2).*phi.*WW+phi.^2.*WW+2.*phi.*WW.^2+(-2).* ...

phi.^2.*WW.^2+phi.^2.*WW.^3+(-1).*XX+2.*phi.*XX+(-1).*phi.^2.*XX+(-2).* ...

phi.*WW.*XX+2.*phi.^2.*WW.*XX+(-1).*phi.^2.*WW.^2.*XX+phi.*WW.*YY+(-1).* ...

phi.^2.*WW.*YY+(-1).*WW.^2.*YY+2.*phi.^2.*WW.^2.*YY+(-1).*phi.*WW.^3.* ...

YY+(-1).*phi.^2.*WW.^3.*YY+(-1).*phi.*XX.*YY+phi.^2.*XX.*YY+WW.*XX.*YY+( ...

-2).*phi.^2.*WW.*XX.*YY+phi.*WW.^2.*XX.*YY+phi.^2.*WW.^2.*XX.*YY+phi.* ...

WW.*ZZ+(-1).*phi.^2.*WW.*ZZ+(-1).*WW.^2.*ZZ+2.*phi.^2.*WW.^2.*ZZ+(-1).* ...

phi.*WW.^3.*ZZ+(-1).*phi.^2.*WW.^3.*ZZ+(-1).*phi.*XX.*ZZ+phi.^2.*XX.*ZZ+ ...

WW.*XX.*ZZ+(-2).*phi.^2.*WW.*XX.*ZZ+phi.*WW.^2.*XX.*ZZ+phi.^2.*WW.^2.* ...

XX.*ZZ+phi.^2.*WW.*YY.*ZZ+(-2).*phi.*WW.^2.*YY.*ZZ+(-2).*phi.^2.*WW.^2.* ...

YY.*ZZ+WW.^3.*YY.*ZZ+2.*phi.*WW.^3.*YY.*ZZ+phi.^2.*WW.^3.*YY.*ZZ+(-1).* ...

phi.^2.*XX.*YY.*ZZ+2.*phi.*WW.*XX.*YY.*ZZ+2.*phi.^2.*WW.*XX.*YY.*ZZ+(-1) ...

.*WW.^2.*XX.*YY.*ZZ+(-2).*phi.*WW.^2.*XX.*YY.*ZZ+(-1).*phi.^2.*WW.^2.* ...

XX.*YY.*ZZ).*((-3).*WW.^3+2.*phi.*WW.^3+(-1).*WW.^4+(-2).*phi.*WW.^4+3.* ...

WW.^2.*XX+(-2).*phi.*WW.^2.*XX+WW.^3.*XX+2.*phi.*WW.^3.*XX+(-1).*WW.^2.* ...

YY+2.*phi.*WW.^2.*YY+WW.^3.*YY+(-6).*phi.*WW.^3.*YY+4.*WW.^4.*YY+4.* ...

phi.*WW.^4.*YY+(-3).*WW.^3.*XX.*YY+2.*phi.*WW.^3.*XX.*YY+(-1).*WW.^4.* ...

XX.*YY+(-2).*phi.*WW.^4.*XX.*YY+WW.^2.*ZZ+(-2).*phi.*WW.^2.*ZZ+3.* ...

WW.^3.*ZZ+2.*phi.*WW.^3.*ZZ+(-4).*WW.^2.*XX.*ZZ+4.*phi.*WW.^2.*XX.*ZZ+( ...

-1).*WW.^3.*XX.*ZZ+(-6).*phi.*WW.^3.*XX.*ZZ+WW.^4.*XX.*ZZ+2.*phi.* ...

WW.^4.*XX.*ZZ+(-1).*WW.^3.*YY.*ZZ+2.*phi.*WW.^3.*YY.*ZZ+(-3).*WW.^4.* ...

YY.*ZZ+(-2).*phi.*WW.^4.*YY.*ZZ+WW.^2.*XX.*YY.*ZZ+(-2).*phi.*WW.^2.*XX.* ...

YY.*ZZ+3.*WW.^3.*XX.*YY.*ZZ+2.*phi.*WW.^3.*XX.*YY.*ZZ).^2+(-72).*(WW+( ...

-2).*phi.*WW+phi.^2.*WW+2.*phi.*WW.^2+(-2).*phi.^2.*WW.^2+phi.^2.*WW.^3+ ...

(-1).*XX+2.*phi.*XX+(-1).*phi.^2.*XX+(-2).*phi.*WW.*XX+2.*phi.^2.*WW.* ...

XX+(-1).*phi.^2.*WW.^2.*XX+phi.*WW.*YY+(-1).*phi.^2.*WW.*YY+(-1).* ...

WW.^2.*YY+2.*phi.^2.*WW.^2.*YY+(-1).*phi.*WW.^3.*YY+(-1).*phi.^2.* ...

WW.^3.*YY+(-1).*phi.*XX.*YY+phi.^2.*XX.*YY+WW.*XX.*YY+(-2).*phi.^2.*WW.* ...

XX.*YY+phi.*WW.^2.*XX.*YY+phi.^2.*WW.^2.*XX.*YY+phi.*WW.*ZZ+(-1).* ...

phi.^2.*WW.*ZZ+(-1).*WW.^2.*ZZ+2.*phi.^2.*WW.^2.*ZZ+(-1).*phi.*WW.^3.* ...

ZZ+(-1).*phi.^2.*WW.^3.*ZZ+(-1).*phi.*XX.*ZZ+phi.^2.*XX.*ZZ+WW.*XX.*ZZ+( ...

-2).*phi.^2.*WW.*XX.*ZZ+phi.*WW.^2.*XX.*ZZ+phi.^2.*WW.^2.*XX.*ZZ+ ...

phi.^2.*WW.*YY.*ZZ+(-2).*phi.*WW.^2.*YY.*ZZ+(-2).*phi.^2.*WW.^2.*YY.*ZZ+ ...

WW.^3.*YY.*ZZ+2.*phi.*WW.^3.*YY.*ZZ+phi.^2.*WW.^3.*YY.*ZZ+(-1).*phi.^2.* ...

XX.*YY.*ZZ+2.*phi.*WW.*XX.*YY.*ZZ+2.*phi.^2.*WW.*XX.*YY.*ZZ+(-1).* ...

WW.^2.*XX.*YY.*ZZ+(-2).*phi.*WW.^2.*XX.*YY.*ZZ+(-1).*phi.^2.*WW.^2.*XX.* ...

YY.*ZZ).*(WW.^4+(-1).*WW.^3.*XX+WW.^3.*YY+(-2).*WW.^4.*YY+WW.^4.*XX.*YY+ ...

(-1).*WW.^3.*ZZ+2.*WW.^3.*XX.*ZZ+(-1).*WW.^4.*XX.*ZZ+WW.^4.*YY.*ZZ+(-1) ...

.*WW.^3.*XX.*YY.*ZZ).*(3.*WW.^2+(-4).*phi.*WW.^2+phi.^2.*WW.^2+3.*WW.^3+ ...

2.*phi.*WW.^3+(-2).*phi.^2.*WW.^3+2.*phi.*WW.^4+phi.^2.*WW.^4+(-3).*WW.* ...

XX+4.*phi.*WW.*XX+(-1).*phi.^2.*WW.*XX+(-3).*WW.^2.*XX+(-2).*phi.* ...

WW.^2.*XX+2.*phi.^2.*WW.^2.*XX+(-2).*phi.*WW.^3.*XX+(-1).*phi.^2.* ...

WW.^3.*XX+(-1).*phi.*WW.*YY+phi.^2.*WW.*YY+WW.^2.*YY+2.*phi.*WW.^2.*YY+( ...

-4).*phi.^2.*WW.^2.*YY+(-5).*WW.^3.*YY+5.*phi.*WW.^3.*YY+5.*phi.^2.* ...

WW.^3.*YY+(-2).*WW.^4.*YY+(-6).*phi.*WW.^4.*YY+(-2).*phi.^2.*WW.^4.*YY+ ...

3.*WW.^2.*XX.*YY+(-5).*phi.*WW.^2.*XX.*YY+phi.^2.*WW.^2.*XX.*YY+3.* ...

WW.^3.*XX.*YY+4.*phi.*WW.^3.*XX.*YY+(-2).*phi.^2.*WW.^3.*XX.*YY+phi.* ...

WW.^4.*XX.*YY+phi.^2.*WW.^4.*XX.*YY+phi.*WW.*ZZ+(-1).*phi.^2.*WW.*ZZ+( ...

-3).*WW.^2.*ZZ+4.*phi.*WW.^2.*ZZ+2.*phi.^2.*WW.^2.*ZZ+(-3).*WW.^3.*ZZ+( ...

-5).*phi.*WW.^3.*ZZ+(-1).*phi.^2.*WW.^3.*ZZ+2.*WW.*XX.*ZZ+(-6).*phi.* ...

WW.*XX.*ZZ+2.*phi.^2.*WW.*XX.*ZZ+5.*WW.^2.*XX.*ZZ+5.*phi.*WW.^2.*XX.*ZZ+ ...

(-5).*phi.^2.*WW.^2.*XX.*ZZ+(-1).*WW.^3.*XX.*ZZ+2.*phi.*WW.^3.*XX.*ZZ+ ...

4.*phi.^2.*WW.^3.*XX.*ZZ+(-1).*phi.*WW.^4.*XX.*ZZ+(-1).*phi.^2.*WW.^4.* ...

XX.*ZZ+(-2).*phi.*WW.^2.*YY.*ZZ+phi.^2.*WW.^2.*YY.*ZZ+3.*WW.^3.*YY.*ZZ+( ...

-2).*phi.*WW.^3.*YY.*ZZ+(-2).*phi.^2.*WW.^3.*YY.*ZZ+3.*WW.^4.*YY.*ZZ+4.* ...

phi.*WW.^4.*YY.*ZZ+phi.^2.*WW.^4.*YY.*ZZ+2.*phi.*WW.*XX.*YY.*ZZ+(-1).* ...

phi.^2.*WW.*XX.*YY.*ZZ+(-3).*WW.^2.*XX.*YY.*ZZ+2.*phi.*WW.^2.*XX.*YY.* ...

ZZ+2.*phi.^2.*WW.^2.*XX.*YY.*ZZ+(-3).*WW.^3.*XX.*YY.*ZZ+(-4).*phi.* ...

WW.^3.*XX.*YY.*ZZ+(-1).*phi.^2.*WW.^3.*XX.*YY.*ZZ)+2.*(3.*WW.^2+(-4).* ...

phi.*WW.^2+phi.^2.*WW.^2+3.*WW.^3+2.*phi.*WW.^3+(-2).*phi.^2.*WW.^3+2.* ...

phi.*WW.^4+phi.^2.*WW.^4+(-3).*WW.*XX+4.*phi.*WW.*XX+(-1).*phi.^2.*WW.* ...

XX+(-3).*WW.^2.*XX+(-2).*phi.*WW.^2.*XX+2.*phi.^2.*WW.^2.*XX+(-2).*phi.* ...

WW.^3.*XX+(-1).*phi.^2.*WW.^3.*XX+(-1).*phi.*WW.*YY+phi.^2.*WW.*YY+ ...

WW.^2.*YY+2.*phi.*WW.^2.*YY+(-4).*phi.^2.*WW.^2.*YY+(-5).*WW.^3.*YY+5.* ...

phi.*WW.^3.*YY+5.*phi.^2.*WW.^3.*YY+(-2).*WW.^4.*YY+(-6).*phi.*WW.^4.* ...

YY+(-2).*phi.^2.*WW.^4.*YY+3.*WW.^2.*XX.*YY+(-5).*phi.*WW.^2.*XX.*YY+ ...

phi.^2.*WW.^2.*XX.*YY+3.*WW.^3.*XX.*YY+4.*phi.*WW.^3.*XX.*YY+(-2).* ...

phi.^2.*WW.^3.*XX.*YY+phi.*WW.^4.*XX.*YY+phi.^2.*WW.^4.*XX.*YY+phi.*WW.* ...

ZZ+(-1).*phi.^2.*WW.*ZZ+(-3).*WW.^2.*ZZ+4.*phi.*WW.^2.*ZZ+2.*phi.^2.* ...

WW.^2.*ZZ+(-3).*WW.^3.*ZZ+(-5).*phi.*WW.^3.*ZZ+(-1).*phi.^2.*WW.^3.*ZZ+ ...

2.*WW.*XX.*ZZ+(-6).*phi.*WW.*XX.*ZZ+2.*phi.^2.*WW.*XX.*ZZ+5.*WW.^2.*XX.* ...

ZZ+5.*phi.*WW.^2.*XX.*ZZ+(-5).*phi.^2.*WW.^2.*XX.*ZZ+(-1).*WW.^3.*XX.* ...

ZZ+2.*phi.*WW.^3.*XX.*ZZ+4.*phi.^2.*WW.^3.*XX.*ZZ+(-1).*phi.*WW.^4.*XX.* ...

ZZ+(-1).*phi.^2.*WW.^4.*XX.*ZZ+(-2).*phi.*WW.^2.*YY.*ZZ+phi.^2.*WW.^2.* ...

YY.*ZZ+3.*WW.^3.*YY.*ZZ+(-2).*phi.*WW.^3.*YY.*ZZ+(-2).*phi.^2.*WW.^3.* ...

YY.*ZZ+3.*WW.^4.*YY.*ZZ+4.*phi.*WW.^4.*YY.*ZZ+phi.^2.*WW.^4.*YY.*ZZ+2.* ...

phi.*WW.*XX.*YY.*ZZ+(-1).*phi.^2.*WW.*XX.*YY.*ZZ+(-3).*WW.^2.*XX.*YY.* ...

ZZ+2.*phi.*WW.^2.*XX.*YY.*ZZ+2.*phi.^2.*WW.^2.*XX.*YY.*ZZ+(-3).*WW.^3.* ...

XX.*YY.*ZZ+(-4).*phi.*WW.^3.*XX.*YY.*ZZ+(-1).*phi.^2.*WW.^3.*XX.*YY.*ZZ) ...

.^3+(-9).*((-3).*WW.^3+2.*phi.*WW.^3+(-1).*WW.^4+(-2).*phi.*WW.^4+3.* ...

WW.^2.*XX+(-2).*phi.*WW.^2.*XX+WW.^3.*XX+2.*phi.*WW.^3.*XX+(-1).*WW.^2.* ...

YY+2.*phi.*WW.^2.*YY+WW.^3.*YY+(-6).*phi.*WW.^3.*YY+4.*WW.^4.*YY+4.* ...

phi.*WW.^4.*YY+(-3).*WW.^3.*XX.*YY+2.*phi.*WW.^3.*XX.*YY+(-1).*WW.^4.* ...

XX.*YY+(-2).*phi.*WW.^4.*XX.*YY+WW.^2.*ZZ+(-2).*phi.*WW.^2.*ZZ+3.* ...

WW.^3.*ZZ+2.*phi.*WW.^3.*ZZ+(-4).*WW.^2.*XX.*ZZ+4.*phi.*WW.^2.*XX.*ZZ+( ...

-1).*WW.^3.*XX.*ZZ+(-6).*phi.*WW.^3.*XX.*ZZ+WW.^4.*XX.*ZZ+2.*phi.* ...

WW.^4.*XX.*ZZ+(-1).*WW.^3.*YY.*ZZ+2.*phi.*WW.^3.*YY.*ZZ+(-3).*WW.^4.* ...

YY.*ZZ+(-2).*phi.*WW.^4.*YY.*ZZ+WW.^2.*XX.*YY.*ZZ+(-2).*phi.*WW.^2.*XX.* ...

YY.*ZZ+3.*WW.^3.*XX.*YY.*ZZ+2.*phi.*WW.^3.*XX.*YY.*ZZ).*(3.*WW.^2+(-4).* ...

phi.*WW.^2+phi.^2.*WW.^2+3.*WW.^3+2.*phi.*WW.^3+(-2).*phi.^2.*WW.^3+2.* ...

phi.*WW.^4+phi.^2.*WW.^4+(-3).*WW.*XX+4.*phi.*WW.*XX+(-1).*phi.^2.*WW.* ...

XX+(-3).*WW.^2.*XX+(-2).*phi.*WW.^2.*XX+2.*phi.^2.*WW.^2.*XX+(-2).*phi.* ...

WW.^3.*XX+(-1).*phi.^2.*WW.^3.*XX+(-1).*phi.*WW.*YY+phi.^2.*WW.*YY+ ...

WW.^2.*YY+2.*phi.*WW.^2.*YY+(-4).*phi.^2.*WW.^2.*YY+(-5).*WW.^3.*YY+5.* ...

phi.*WW.^3.*YY+5.*phi.^2.*WW.^3.*YY+(-2).*WW.^4.*YY+(-6).*phi.*WW.^4.* ...

YY+(-2).*phi.^2.*WW.^4.*YY+3.*WW.^2.*XX.*YY+(-5).*phi.*WW.^2.*XX.*YY+ ...

phi.^2.*WW.^2.*XX.*YY+3.*WW.^3.*XX.*YY+4.*phi.*WW.^3.*XX.*YY+(-2).* ...

phi.^2.*WW.^3.*XX.*YY+phi.*WW.^4.*XX.*YY+phi.^2.*WW.^4.*XX.*YY+phi.*WW.* ...

ZZ+(-1).*phi.^2.*WW.*ZZ+(-3).*WW.^2.*ZZ+4.*phi.*WW.^2.*ZZ+2.*phi.^2.* ...

WW.^2.*ZZ+(-3).*WW.^3.*ZZ+(-5).*phi.*WW.^3.*ZZ+(-1).*phi.^2.*WW.^3.*ZZ+ ...

2.*WW.*XX.*ZZ+(-6).*phi.*WW.*XX.*ZZ+2.*phi.^2.*WW.*XX.*ZZ+5.*WW.^2.*XX.* ...

ZZ+5.*phi.*WW.^2.*XX.*ZZ+(-5).*phi.^2.*WW.^2.*XX.*ZZ+(-1).*WW.^3.*XX.* ...

ZZ+2.*phi.*WW.^3.*XX.*ZZ+4.*phi.^2.*WW.^3.*XX.*ZZ+(-1).*phi.*WW.^4.*XX.* ...

ZZ+(-1).*phi.^2.*WW.^4.*XX.*ZZ+(-2).*phi.*WW.^2.*YY.*ZZ+phi.^2.*WW.^2.* ...

YY.*ZZ+3.*WW.^3.*YY.*ZZ+(-2).*phi.*WW.^3.*YY.*ZZ+(-2).*phi.^2.*WW.^3.* ...

YY.*ZZ+3.*WW.^4.*YY.*ZZ+4.*phi.*WW.^4.*YY.*ZZ+phi.^2.*WW.^4.*YY.*ZZ+2.* ...

phi.*WW.*XX.*YY.*ZZ+(-1).*phi.^2.*WW.*XX.*YY.*ZZ+(-3).*WW.^2.*XX.*YY.* ...

ZZ+2.*phi.*WW.^2.*XX.*YY.*ZZ+2.*phi.^2.*WW.^2.*XX.*YY.*ZZ+(-3).*WW.^3.* ...

XX.*YY.*ZZ+(-4).*phi.*WW.^3.*XX.*YY.*ZZ+(-1).*phi.^2.*WW.^3.*XX.*YY.*ZZ) ...

.*((-1).*WW+2.*phi.*WW+(-1).*phi.^2.*WW+(-3).*WW.^2+2.*phi.*WW.^2+ ...

phi.^2.*WW.^2+(-4).*phi.*WW.^3+phi.^2.*WW.^3+(-1).*phi.^2.*WW.^4+XX+(-2) ...

.*phi.*XX+phi.^2.*XX+3.*WW.*XX+(-2).*phi.*WW.*XX+(-1).*phi.^2.*WW.*XX+ ...

4.*phi.*WW.^2.*XX+(-1).*phi.^2.*WW.^2.*XX+phi.^2.*WW.^3.*XX+WW.^2.*YY+( ...

-4).*phi.*WW.^2.*YY+2.*phi.^2.*WW.^2.*YY+3.*WW.^3.*YY+2.*phi.*WW.^3.*YY+ ...

(-4).*phi.^2.*WW.^3.*YY+2.*phi.*WW.^4.*YY+2.*phi.^2.*WW.^4.*YY+(-1).* ...

WW.*XX.*YY+4.*phi.*WW.*XX.*YY+(-2).*phi.^2.*WW.*XX.*YY+(-3).*WW.^2.*XX.* ...

YY+(-2).*phi.*WW.^2.*XX.*YY+4.*phi.^2.*WW.^2.*XX.*YY+(-2).*phi.*WW.^3.* ...

XX.*YY+(-2).*phi.^2.*WW.^3.*XX.*YY+(-2).*phi.*WW.*ZZ+2.*phi.^2.*WW.*ZZ+ ...

3.*WW.^2.*ZZ+(-2).*phi.*WW.^2.*ZZ+(-4).*phi.^2.*WW.^2.*ZZ+WW.^3.*ZZ+4.* ...

phi.*WW.^3.*ZZ+2.*phi.^2.*WW.^3.*ZZ+2.*phi.*XX.*ZZ+(-2).*phi.^2.*XX.*ZZ+ ...

(-3).*WW.*XX.*ZZ+2.*phi.*WW.*XX.*ZZ+4.*phi.^2.*WW.*XX.*ZZ+(-1).*WW.^2.* ...

XX.*ZZ+(-4).*phi.*WW.^2.*XX.*ZZ+(-2).*phi.^2.*WW.^2.*XX.*ZZ+(-1).* ...

phi.^2.*WW.*YY.*ZZ+4.*phi.*WW.^2.*YY.*ZZ+phi.^2.*WW.^2.*YY.*ZZ+(-3).* ...

WW.^3.*YY.*ZZ+(-2).*phi.*WW.^3.*YY.*ZZ+phi.^2.*WW.^3.*YY.*ZZ+(-1).* ...

WW.^4.*YY.*ZZ+(-2).*phi.*WW.^4.*YY.*ZZ+(-1).*phi.^2.*WW.^4.*YY.*ZZ+ ...

phi.^2.*XX.*YY.*ZZ+(-4).*phi.*WW.*XX.*YY.*ZZ+(-1).*phi.^2.*WW.*XX.*YY.* ...

ZZ+3.*WW.^2.*XX.*YY.*ZZ+2.*phi.*WW.^2.*XX.*YY.*ZZ+(-1).*phi.^2.*WW.^2.* ...

XX.*YY.*ZZ+WW.^3.*XX.*YY.*ZZ+2.*phi.*WW.^3.*XX.*YY.*ZZ+phi.^2.*WW.^3.* ...

XX.*YY.*ZZ)+27.*(WW.^4+(-1).*WW.^3.*XX+WW.^3.*YY+(-2).*WW.^4.*YY+WW.^4.* ...

XX.*YY+(-1).*WW.^3.*ZZ+2.*WW.^3.*XX.*ZZ+(-1).*WW.^4.*XX.*ZZ+WW.^4.*YY.* ...

ZZ+(-1).*WW.^3.*XX.*YY.*ZZ).*((-1).*WW+2.*phi.*WW+(-1).*phi.^2.*WW+(-3) ...

.*WW.^2+2.*phi.*WW.^2+phi.^2.*WW.^2+(-4).*phi.*WW.^3+phi.^2.*WW.^3+(-1) ...

.*phi.^2.*WW.^4+XX+(-2).*phi.*XX+phi.^2.*XX+3.*WW.*XX+(-2).*phi.*WW.*XX+ ...

(-1).*phi.^2.*WW.*XX+4.*phi.*WW.^2.*XX+(-1).*phi.^2.*WW.^2.*XX+phi.^2.* ...

WW.^3.*XX+WW.^2.*YY+(-4).*phi.*WW.^2.*YY+2.*phi.^2.*WW.^2.*YY+3.*WW.^3.* ...

YY+2.*phi.*WW.^3.*YY+(-4).*phi.^2.*WW.^3.*YY+2.*phi.*WW.^4.*YY+2.* ...

phi.^2.*WW.^4.*YY+(-1).*WW.*XX.*YY+4.*phi.*WW.*XX.*YY+(-2).*phi.^2.*WW.* ...

XX.*YY+(-3).*WW.^2.*XX.*YY+(-2).*phi.*WW.^2.*XX.*YY+4.*phi.^2.*WW.^2.* ...

XX.*YY+(-2).*phi.*WW.^3.*XX.*YY+(-2).*phi.^2.*WW.^3.*XX.*YY+(-2).*phi.* ...

WW.*ZZ+2.*phi.^2.*WW.*ZZ+3.*WW.^2.*ZZ+(-2).*phi.*WW.^2.*ZZ+(-4).* ...

phi.^2.*WW.^2.*ZZ+WW.^3.*ZZ+4.*phi.*WW.^3.*ZZ+2.*phi.^2.*WW.^3.*ZZ+2.* ...

phi.*XX.*ZZ+(-2).*phi.^2.*XX.*ZZ+(-3).*WW.*XX.*ZZ+2.*phi.*WW.*XX.*ZZ+4.* ...

phi.^2.*WW.*XX.*ZZ+(-1).*WW.^2.*XX.*ZZ+(-4).*phi.*WW.^2.*XX.*ZZ+(-2).* ...

phi.^2.*WW.^2.*XX.*ZZ+(-1).*phi.^2.*WW.*YY.*ZZ+4.*phi.*WW.^2.*YY.*ZZ+ ...

phi.^2.*WW.^2.*YY.*ZZ+(-3).*WW.^3.*YY.*ZZ+(-2).*phi.*WW.^3.*YY.*ZZ+ ...

phi.^2.*WW.^3.*YY.*ZZ+(-1).*WW.^4.*YY.*ZZ+(-2).*phi.*WW.^4.*YY.*ZZ+(-1) ...

.*phi.^2.*WW.^4.*YY.*ZZ+phi.^2.*XX.*YY.*ZZ+(-4).*phi.*WW.*XX.*YY.*ZZ+( ...

-1).*phi.^2.*WW.*XX.*YY.*ZZ+3.*WW.^2.*XX.*YY.*ZZ+2.*phi.*WW.^2.*XX.*YY.* ...

ZZ+(-1).*phi.^2.*WW.^2.*XX.*YY.*ZZ+WW.^3.*XX.*YY.*ZZ+2.*phi.*WW.^3.*XX.* ...

YY.*ZZ+phi.^2.*WW.^3.*XX.*YY.*ZZ).^2+((-4).*(12.*(WW+(-2).*phi.*WW+ ...

phi.^2.*WW+2.*phi.*WW.^2+(-2).*phi.^2.*WW.^2+phi.^2.*WW.^3+(-1).*XX+2.* ...

phi.*XX+(-1).*phi.^2.*XX+(-2).*phi.*WW.*XX+2.*phi.^2.*WW.*XX+(-1).* ...

phi.^2.*WW.^2.*XX+phi.*WW.*YY+(-1).*phi.^2.*WW.*YY+(-1).*WW.^2.*YY+2.* ...

phi.^2.*WW.^2.*YY+(-1).*phi.*WW.^3.*YY+(-1).*phi.^2.*WW.^3.*YY+(-1).* ...

phi.*XX.*YY+phi.^2.*XX.*YY+WW.*XX.*YY+(-2).*phi.^2.*WW.*XX.*YY+phi.* ...

WW.^2.*XX.*YY+phi.^2.*WW.^2.*XX.*YY+phi.*WW.*ZZ+(-1).*phi.^2.*WW.*ZZ+( ...

-1).*WW.^2.*ZZ+2.*phi.^2.*WW.^2.*ZZ+(-1).*phi.*WW.^3.*ZZ+(-1).*phi.^2.* ...

WW.^3.*ZZ+(-1).*phi.*XX.*ZZ+phi.^2.*XX.*ZZ+WW.*XX.*ZZ+(-2).*phi.^2.*WW.* ...

XX.*ZZ+phi.*WW.^2.*XX.*ZZ+phi.^2.*WW.^2.*XX.*ZZ+phi.^2.*WW.*YY.*ZZ+(-2) ...

.*phi.*WW.^2.*YY.*ZZ+(-2).*phi.^2.*WW.^2.*YY.*ZZ+WW.^3.*YY.*ZZ+2.*phi.* ...

WW.^3.*YY.*ZZ+phi.^2.*WW.^3.*YY.*ZZ+(-1).*phi.^2.*XX.*YY.*ZZ+2.*phi.* ...

WW.*XX.*YY.*ZZ+2.*phi.^2.*WW.*XX.*YY.*ZZ+(-1).*WW.^2.*XX.*YY.*ZZ+(-2).* ...

phi.*WW.^2.*XX.*YY.*ZZ+(-1).*phi.^2.*WW.^2.*XX.*YY.*ZZ).*(WW.^4+(-1).* ...

WW.^3.*XX+WW.^3.*YY+(-2).*WW.^4.*YY+WW.^4.*XX.*YY+(-1).*WW.^3.*ZZ+2.* ...

WW.^3.*XX.*ZZ+(-1).*WW.^4.*XX.*ZZ+WW.^4.*YY.*ZZ+(-1).*WW.^3.*XX.*YY.*ZZ) ...

+(3.*WW.^2+(-4).*phi.*WW.^2+phi.^2.*WW.^2+3.*WW.^3+2.*phi.*WW.^3+(-2).* ...

phi.^2.*WW.^3+2.*phi.*WW.^4+phi.^2.*WW.^4+(-3).*WW.*XX+4.*phi.*WW.*XX+( ...

-1).*phi.^2.*WW.*XX+(-3).*WW.^2.*XX+(-2).*phi.*WW.^2.*XX+2.*phi.^2.* ...

WW.^2.*XX+(-2).*phi.*WW.^3.*XX+(-1).*phi.^2.*WW.^3.*XX+(-1).*phi.*WW.* ...

YY+phi.^2.*WW.*YY+WW.^2.*YY+2.*phi.*WW.^2.*YY+(-4).*phi.^2.*WW.^2.*YY+( ...

-5).*WW.^3.*YY+5.*phi.*WW.^3.*YY+5.*phi.^2.*WW.^3.*YY+(-2).*WW.^4.*YY+( ...

-6).*phi.*WW.^4.*YY+(-2).*phi.^2.*WW.^4.*YY+3.*WW.^2.*XX.*YY+(-5).*phi.* ...

WW.^2.*XX.*YY+phi.^2.*WW.^2.*XX.*YY+3.*WW.^3.*XX.*YY+4.*phi.*WW.^3.*XX.* ...

YY+(-2).*phi.^2.*WW.^3.*XX.*YY+phi.*WW.^4.*XX.*YY+phi.^2.*WW.^4.*XX.*YY+ ...

phi.*WW.*ZZ+(-1).*phi.^2.*WW.*ZZ+(-3).*WW.^2.*ZZ+4.*phi.*WW.^2.*ZZ+2.* ...

phi.^2.*WW.^2.*ZZ+(-3).*WW.^3.*ZZ+(-5).*phi.*WW.^3.*ZZ+(-1).*phi.^2.* ...

WW.^3.*ZZ+2.*WW.*XX.*ZZ+(-6).*phi.*WW.*XX.*ZZ+2.*phi.^2.*WW.*XX.*ZZ+5.* ...

WW.^2.*XX.*ZZ+5.*phi.*WW.^2.*XX.*ZZ+(-5).*phi.^2.*WW.^2.*XX.*ZZ+(-1).* ...

WW.^3.*XX.*ZZ+2.*phi.*WW.^3.*XX.*ZZ+4.*phi.^2.*WW.^3.*XX.*ZZ+(-1).*phi.* ...

WW.^4.*XX.*ZZ+(-1).*phi.^2.*WW.^4.*XX.*ZZ+(-2).*phi.*WW.^2.*YY.*ZZ+ ...

phi.^2.*WW.^2.*YY.*ZZ+3.*WW.^3.*YY.*ZZ+(-2).*phi.*WW.^3.*YY.*ZZ+(-2).* ...

phi.^2.*WW.^3.*YY.*ZZ+3.*WW.^4.*YY.*ZZ+4.*phi.*WW.^4.*YY.*ZZ+phi.^2.* ...

WW.^4.*YY.*ZZ+2.*phi.*WW.*XX.*YY.*ZZ+(-1).*phi.^2.*WW.*XX.*YY.*ZZ+(-3).* ...

WW.^2.*XX.*YY.*ZZ+2.*phi.*WW.^2.*XX.*YY.*ZZ+2.*phi.^2.*WW.^2.*XX.*YY.* ...

ZZ+(-3).*WW.^3.*XX.*YY.*ZZ+(-4).*phi.*WW.^3.*XX.*YY.*ZZ+(-1).*phi.^2.* ...

WW.^3.*XX.*YY.*ZZ).^2+(-3).*((-3).*WW.^3+2.*phi.*WW.^3+(-1).*WW.^4+(-2) ...

.*phi.*WW.^4+3.*WW.^2.*XX+(-2).*phi.*WW.^2.*XX+WW.^3.*XX+2.*phi.*WW.^3.* ...

XX+(-1).*WW.^2.*YY+2.*phi.*WW.^2.*YY+WW.^3.*YY+(-6).*phi.*WW.^3.*YY+4.* ...

WW.^4.*YY+4.*phi.*WW.^4.*YY+(-3).*WW.^3.*XX.*YY+2.*phi.*WW.^3.*XX.*YY+( ...

-1).*WW.^4.*XX.*YY+(-2).*phi.*WW.^4.*XX.*YY+WW.^2.*ZZ+(-2).*phi.*WW.^2.* ...

ZZ+3.*WW.^3.*ZZ+2.*phi.*WW.^3.*ZZ+(-4).*WW.^2.*XX.*ZZ+4.*phi.*WW.^2.* ...

XX.*ZZ+(-1).*WW.^3.*XX.*ZZ+(-6).*phi.*WW.^3.*XX.*ZZ+WW.^4.*XX.*ZZ+2.* ...

phi.*WW.^4.*XX.*ZZ+(-1).*WW.^3.*YY.*ZZ+2.*phi.*WW.^3.*YY.*ZZ+(-3).* ...

WW.^4.*YY.*ZZ+(-2).*phi.*WW.^4.*YY.*ZZ+WW.^2.*XX.*YY.*ZZ+(-2).*phi.* ...

WW.^2.*XX.*YY.*ZZ+3.*WW.^3.*XX.*YY.*ZZ+2.*phi.*WW.^3.*XX.*YY.*ZZ).*((-1) ...

.*WW+2.*phi.*WW+(-1).*phi.^2.*WW+(-3).*WW.^2+2.*phi.*WW.^2+phi.^2.* ...

WW.^2+(-4).*phi.*WW.^3+phi.^2.*WW.^3+(-1).*phi.^2.*WW.^4+XX+(-2).*phi.* ...

XX+phi.^2.*XX+3.*WW.*XX+(-2).*phi.*WW.*XX+(-1).*phi.^2.*WW.*XX+4.*phi.* ...

WW.^2.*XX+(-1).*phi.^2.*WW.^2.*XX+phi.^2.*WW.^3.*XX+WW.^2.*YY+(-4).* ...

phi.*WW.^2.*YY+2.*phi.^2.*WW.^2.*YY+3.*WW.^3.*YY+2.*phi.*WW.^3.*YY+(-4) ...

.*phi.^2.*WW.^3.*YY+2.*phi.*WW.^4.*YY+2.*phi.^2.*WW.^4.*YY+(-1).*WW.* ...

XX.*YY+4.*phi.*WW.*XX.*YY+(-2).*phi.^2.*WW.*XX.*YY+(-3).*WW.^2.*XX.*YY+( ...

-2).*phi.*WW.^2.*XX.*YY+4.*phi.^2.*WW.^2.*XX.*YY+(-2).*phi.*WW.^3.*XX.* ...

YY+(-2).*phi.^2.*WW.^3.*XX.*YY+(-2).*phi.*WW.*ZZ+2.*phi.^2.*WW.*ZZ+3.* ...

WW.^2.*ZZ+(-2).*phi.*WW.^2.*ZZ+(-4).*phi.^2.*WW.^2.*ZZ+WW.^3.*ZZ+4.* ...

phi.*WW.^3.*ZZ+2.*phi.^2.*WW.^3.*ZZ+2.*phi.*XX.*ZZ+(-2).*phi.^2.*XX.*ZZ+ ...

(-3).*WW.*XX.*ZZ+2.*phi.*WW.*XX.*ZZ+4.*phi.^2.*WW.*XX.*ZZ+(-1).*WW.^2.* ...

XX.*ZZ+(-4).*phi.*WW.^2.*XX.*ZZ+(-2).*phi.^2.*WW.^2.*XX.*ZZ+(-1).* ...

phi.^2.*WW.*YY.*ZZ+4.*phi.*WW.^2.*YY.*ZZ+phi.^2.*WW.^2.*YY.*ZZ+(-3).* ...

WW.^3.*YY.*ZZ+(-2).*phi.*WW.^3.*YY.*ZZ+phi.^2.*WW.^3.*YY.*ZZ+(-1).* ...

WW.^4.*YY.*ZZ+(-2).*phi.*WW.^4.*YY.*ZZ+(-1).*phi.^2.*WW.^4.*YY.*ZZ+ ...

phi.^2.*XX.*YY.*ZZ+(-4).*phi.*WW.*XX.*YY.*ZZ+(-1).*phi.^2.*WW.*XX.*YY.* ...

ZZ+3.*WW.^2.*XX.*YY.*ZZ+2.*phi.*WW.^2.*XX.*YY.*ZZ+(-1).*phi.^2.*WW.^2.* ...

XX.*YY.*ZZ+WW.^3.*XX.*YY.*ZZ+2.*phi.*WW.^3.*XX.*YY.*ZZ+phi.^2.*WW.^3.* ...

XX.*YY.*ZZ)).^3+(27.*(WW+(-2).*phi.*WW+phi.^2.*WW+2.*phi.*WW.^2+(-2).* ...

phi.^2.*WW.^2+phi.^2.*WW.^3+(-1).*XX+2.*phi.*XX+(-1).*phi.^2.*XX+(-2).* ...

phi.*WW.*XX+2.*phi.^2.*WW.*XX+(-1).*phi.^2.*WW.^2.*XX+phi.*WW.*YY+(-1).* ...

phi.^2.*WW.*YY+(-1).*WW.^2.*YY+2.*phi.^2.*WW.^2.*YY+(-1).*phi.*WW.^3.* ...

YY+(-1).*phi.^2.*WW.^3.*YY+(-1).*phi.*XX.*YY+phi.^2.*XX.*YY+WW.*XX.*YY+( ...

-2).*phi.^2.*WW.*XX.*YY+phi.*WW.^2.*XX.*YY+phi.^2.*WW.^2.*XX.*YY+phi.* ...

WW.*ZZ+(-1).*phi.^2.*WW.*ZZ+(-1).*WW.^2.*ZZ+2.*phi.^2.*WW.^2.*ZZ+(-1).* ...

phi.*WW.^3.*ZZ+(-1).*phi.^2.*WW.^3.*ZZ+(-1).*phi.*XX.*ZZ+phi.^2.*XX.*ZZ+ ...

WW.*XX.*ZZ+(-2).*phi.^2.*WW.*XX.*ZZ+phi.*WW.^2.*XX.*ZZ+phi.^2.*WW.^2.* ...

XX.*ZZ+phi.^2.*WW.*YY.*ZZ+(-2).*phi.*WW.^2.*YY.*ZZ+(-2).*phi.^2.*WW.^2.* ...

YY.*ZZ+WW.^3.*YY.*ZZ+2.*phi.*WW.^3.*YY.*ZZ+phi.^2.*WW.^3.*YY.*ZZ+(-1).* ...

phi.^2.*XX.*YY.*ZZ+2.*phi.*WW.*XX.*YY.*ZZ+2.*phi.^2.*WW.*XX.*YY.*ZZ+(-1) ...

.*WW.^2.*XX.*YY.*ZZ+(-2).*phi.*WW.^2.*XX.*YY.*ZZ+(-1).*phi.^2.*WW.^2.* ...

XX.*YY.*ZZ).*((-3).*WW.^3+2.*phi.*WW.^3+(-1).*WW.^4+(-2).*phi.*WW.^4+3.* ...

WW.^2.*XX+(-2).*phi.*WW.^2.*XX+WW.^3.*XX+2.*phi.*WW.^3.*XX+(-1).*WW.^2.* ...

YY+2.*phi.*WW.^2.*YY+WW.^3.*YY+(-6).*phi.*WW.^3.*YY+4.*WW.^4.*YY+4.* ...

phi.*WW.^4.*YY+(-3).*WW.^3.*XX.*YY+2.*phi.*WW.^3.*XX.*YY+(-1).*WW.^4.* ...

XX.*YY+(-2).*phi.*WW.^4.*XX.*YY+WW.^2.*ZZ+(-2).*phi.*WW.^2.*ZZ+3.* ...

WW.^3.*ZZ+2.*phi.*WW.^3.*ZZ+(-4).*WW.^2.*XX.*ZZ+4.*phi.*WW.^2.*XX.*ZZ+( ...

-1).*WW.^3.*XX.*ZZ+(-6).*phi.*WW.^3.*XX.*ZZ+WW.^4.*XX.*ZZ+2.*phi.* ...

WW.^4.*XX.*ZZ+(-1).*WW.^3.*YY.*ZZ+2.*phi.*WW.^3.*YY.*ZZ+(-3).*WW.^4.* ...

YY.*ZZ+(-2).*phi.*WW.^4.*YY.*ZZ+WW.^2.*XX.*YY.*ZZ+(-2).*phi.*WW.^2.*XX.* ...

YY.*ZZ+3.*WW.^3.*XX.*YY.*ZZ+2.*phi.*WW.^3.*XX.*YY.*ZZ).^2+(-72).*(WW+( ...

-2).*phi.*WW+phi.^2.*WW+2.*phi.*WW.^2+(-2).*phi.^2.*WW.^2+phi.^2.*WW.^3+ ...

(-1).*XX+2.*phi.*XX+(-1).*phi.^2.*XX+(-2).*phi.*WW.*XX+2.*phi.^2.*WW.* ...

XX+(-1).*phi.^2.*WW.^2.*XX+phi.*WW.*YY+(-1).*phi.^2.*WW.*YY+(-1).* ...

WW.^2.*YY+2.*phi.^2.*WW.^2.*YY+(-1).*phi.*WW.^3.*YY+(-1).*phi.^2.* ...

WW.^3.*YY+(-1).*phi.*XX.*YY+phi.^2.*XX.*YY+WW.*XX.*YY+(-2).*phi.^2.*WW.* ...

XX.*YY+phi.*WW.^2.*XX.*YY+phi.^2.*WW.^2.*XX.*YY+phi.*WW.*ZZ+(-1).* ...

phi.^2.*WW.*ZZ+(-1).*WW.^2.*ZZ+2.*phi.^2.*WW.^2.*ZZ+(-1).*phi.*WW.^3.* ...

ZZ+(-1).*phi.^2.*WW.^3.*ZZ+(-1).*phi.*XX.*ZZ+phi.^2.*XX.*ZZ+WW.*XX.*ZZ+( ...

-2).*phi.^2.*WW.*XX.*ZZ+phi.*WW.^2.*XX.*ZZ+phi.^2.*WW.^2.*XX.*ZZ+ ...

phi.^2.*WW.*YY.*ZZ+(-2).*phi.*WW.^2.*YY.*ZZ+(-2).*phi.^2.*WW.^2.*YY.*ZZ+ ...

WW.^3.*YY.*ZZ+2.*phi.*WW.^3.*YY.*ZZ+phi.^2.*WW.^3.*YY.*ZZ+(-1).*phi.^2.* ...

XX.*YY.*ZZ+2.*phi.*WW.*XX.*YY.*ZZ+2.*phi.^2.*WW.*XX.*YY.*ZZ+(-1).* ...

WW.^2.*XX.*YY.*ZZ+(-2).*phi.*WW.^2.*XX.*YY.*ZZ+(-1).*phi.^2.*WW.^2.*XX.* ...

YY.*ZZ).*(WW.^4+(-1).*WW.^3.*XX+WW.^3.*YY+(-2).*WW.^4.*YY+WW.^4.*XX.*YY+ ...

(-1).*WW.^3.*ZZ+2.*WW.^3.*XX.*ZZ+(-1).*WW.^4.*XX.*ZZ+WW.^4.*YY.*ZZ+(-1) ...

.*WW.^3.*XX.*YY.*ZZ).*(3.*WW.^2+(-4).*phi.*WW.^2+phi.^2.*WW.^2+3.*WW.^3+ ...

2.*phi.*WW.^3+(-2).*phi.^2.*WW.^3+2.*phi.*WW.^4+phi.^2.*WW.^4+(-3).*WW.* ...

XX+4.*phi.*WW.*XX+(-1).*phi.^2.*WW.*XX+(-3).*WW.^2.*XX+(-2).*phi.* ...

WW.^2.*XX+2.*phi.^2.*WW.^2.*XX+(-2).*phi.*WW.^3.*XX+(-1).*phi.^2.* ...

WW.^3.*XX+(-1).*phi.*WW.*YY+phi.^2.*WW.*YY+WW.^2.*YY+2.*phi.*WW.^2.*YY+( ...

-4).*phi.^2.*WW.^2.*YY+(-5).*WW.^3.*YY+5.*phi.*WW.^3.*YY+5.*phi.^2.* ...

WW.^3.*YY+(-2).*WW.^4.*YY+(-6).*phi.*WW.^4.*YY+(-2).*phi.^2.*WW.^4.*YY+ ...

3.*WW.^2.*XX.*YY+(-5).*phi.*WW.^2.*XX.*YY+phi.^2.*WW.^2.*XX.*YY+3.* ...

WW.^3.*XX.*YY+4.*phi.*WW.^3.*XX.*YY+(-2).*phi.^2.*WW.^3.*XX.*YY+phi.* ...

WW.^4.*XX.*YY+phi.^2.*WW.^4.*XX.*YY+phi.*WW.*ZZ+(-1).*phi.^2.*WW.*ZZ+( ...

-3).*WW.^2.*ZZ+4.*phi.*WW.^2.*ZZ+2.*phi.^2.*WW.^2.*ZZ+(-3).*WW.^3.*ZZ+( ...

-5).*phi.*WW.^3.*ZZ+(-1).*phi.^2.*WW.^3.*ZZ+2.*WW.*XX.*ZZ+(-6).*phi.* ...

WW.*XX.*ZZ+2.*phi.^2.*WW.*XX.*ZZ+5.*WW.^2.*XX.*ZZ+5.*phi.*WW.^2.*XX.*ZZ+ ...

(-5).*phi.^2.*WW.^2.*XX.*ZZ+(-1).*WW.^3.*XX.*ZZ+2.*phi.*WW.^3.*XX.*ZZ+ ...

4.*phi.^2.*WW.^3.*XX.*ZZ+(-1).*phi.*WW.^4.*XX.*ZZ+(-1).*phi.^2.*WW.^4.* ...

XX.*ZZ+(-2).*phi.*WW.^2.*YY.*ZZ+phi.^2.*WW.^2.*YY.*ZZ+3.*WW.^3.*YY.*ZZ+( ...

-2).*phi.*WW.^3.*YY.*ZZ+(-2).*phi.^2.*WW.^3.*YY.*ZZ+3.*WW.^4.*YY.*ZZ+4.* ...

phi.*WW.^4.*YY.*ZZ+phi.^2.*WW.^4.*YY.*ZZ+2.*phi.*WW.*XX.*YY.*ZZ+(-1).* ...

phi.^2.*WW.*XX.*YY.*ZZ+(-3).*WW.^2.*XX.*YY.*ZZ+2.*phi.*WW.^2.*XX.*YY.* ...

ZZ+2.*phi.^2.*WW.^2.*XX.*YY.*ZZ+(-3).*WW.^3.*XX.*YY.*ZZ+(-4).*phi.* ...

WW.^3.*XX.*YY.*ZZ+(-1).*phi.^2.*WW.^3.*XX.*YY.*ZZ)+2.*(3.*WW.^2+(-4).* ...

phi.*WW.^2+phi.^2.*WW.^2+3.*WW.^3+2.*phi.*WW.^3+(-2).*phi.^2.*WW.^3+2.* ...

phi.*WW.^4+phi.^2.*WW.^4+(-3).*WW.*XX+4.*phi.*WW.*XX+(-1).*phi.^2.*WW.* ...

XX+(-3).*WW.^2.*XX+(-2).*phi.*WW.^2.*XX+2.*phi.^2.*WW.^2.*XX+(-2).*phi.* ...

WW.^3.*XX+(-1).*phi.^2.*WW.^3.*XX+(-1).*phi.*WW.*YY+phi.^2.*WW.*YY+ ...

WW.^2.*YY+2.*phi.*WW.^2.*YY+(-4).*phi.^2.*WW.^2.*YY+(-5).*WW.^3.*YY+5.* ...

phi.*WW.^3.*YY+5.*phi.^2.*WW.^3.*YY+(-2).*WW.^4.*YY+(-6).*phi.*WW.^4.* ...

YY+(-2).*phi.^2.*WW.^4.*YY+3.*WW.^2.*XX.*YY+(-5).*phi.*WW.^2.*XX.*YY+ ...

phi.^2.*WW.^2.*XX.*YY+3.*WW.^3.*XX.*YY+4.*phi.*WW.^3.*XX.*YY+(-2).* ...

phi.^2.*WW.^3.*XX.*YY+phi.*WW.^4.*XX.*YY+phi.^2.*WW.^4.*XX.*YY+phi.*WW.* ...

ZZ+(-1).*phi.^2.*WW.*ZZ+(-3).*WW.^2.*ZZ+4.*phi.*WW.^2.*ZZ+2.*phi.^2.* ...

WW.^2.*ZZ+(-3).*WW.^3.*ZZ+(-5).*phi.*WW.^3.*ZZ+(-1).*phi.^2.*WW.^3.*ZZ+ ...

2.*WW.*XX.*ZZ+(-6).*phi.*WW.*XX.*ZZ+2.*phi.^2.*WW.*XX.*ZZ+5.*WW.^2.*XX.* ...

ZZ+5.*phi.*WW.^2.*XX.*ZZ+(-5).*phi.^2.*WW.^2.*XX.*ZZ+(-1).*WW.^3.*XX.* ...

ZZ+2.*phi.*WW.^3.*XX.*ZZ+4.*phi.^2.*WW.^3.*XX.*ZZ+(-1).*phi.*WW.^4.*XX.* ...

ZZ+(-1).*phi.^2.*WW.^4.*XX.*ZZ+(-2).*phi.*WW.^2.*YY.*ZZ+phi.^2.*WW.^2.* ...

YY.*ZZ+3.*WW.^3.*YY.*ZZ+(-2).*phi.*WW.^3.*YY.*ZZ+(-2).*phi.^2.*WW.^3.* ...

YY.*ZZ+3.*WW.^4.*YY.*ZZ+4.*phi.*WW.^4.*YY.*ZZ+phi.^2.*WW.^4.*YY.*ZZ+2.* ...

phi.*WW.*XX.*YY.*ZZ+(-1).*phi.^2.*WW.*XX.*YY.*ZZ+(-3).*WW.^2.*XX.*YY.* ...

ZZ+2.*phi.*WW.^2.*XX.*YY.*ZZ+2.*phi.^2.*WW.^2.*XX.*YY.*ZZ+(-3).*WW.^3.* ...

XX.*YY.*ZZ+(-4).*phi.*WW.^3.*XX.*YY.*ZZ+(-1).*phi.^2.*WW.^3.*XX.*YY.*ZZ) ...

.^3+(-9).*((-3).*WW.^3+2.*phi.*WW.^3+(-1).*WW.^4+(-2).*phi.*WW.^4+3.* ...

WW.^2.*XX+(-2).*phi.*WW.^2.*XX+WW.^3.*XX+2.*phi.*WW.^3.*XX+(-1).*WW.^2.* ...

YY+2.*phi.*WW.^2.*YY+WW.^3.*YY+(-6).*phi.*WW.^3.*YY+4.*WW.^4.*YY+4.* ...

phi.*WW.^4.*YY+(-3).*WW.^3.*XX.*YY+2.*phi.*WW.^3.*XX.*YY+(-1).*WW.^4.* ...

XX.*YY+(-2).*phi.*WW.^4.*XX.*YY+WW.^2.*ZZ+(-2).*phi.*WW.^2.*ZZ+3.* ...

WW.^3.*ZZ+2.*phi.*WW.^3.*ZZ+(-4).*WW.^2.*XX.*ZZ+4.*phi.*WW.^2.*XX.*ZZ+( ...

-1).*WW.^3.*XX.*ZZ+(-6).*phi.*WW.^3.*XX.*ZZ+WW.^4.*XX.*ZZ+2.*phi.* ...

WW.^4.*XX.*ZZ+(-1).*WW.^3.*YY.*ZZ+2.*phi.*WW.^3.*YY.*ZZ+(-3).*WW.^4.* ...

YY.*ZZ+(-2).*phi.*WW.^4.*YY.*ZZ+WW.^2.*XX.*YY.*ZZ+(-2).*phi.*WW.^2.*XX.* ...

YY.*ZZ+3.*WW.^3.*XX.*YY.*ZZ+2.*phi.*WW.^3.*XX.*YY.*ZZ).*(3.*WW.^2+(-4).* ...

phi.*WW.^2+phi.^2.*WW.^2+3.*WW.^3+2.*phi.*WW.^3+(-2).*phi.^2.*WW.^3+2.* ...

phi.*WW.^4+phi.^2.*WW.^4+(-3).*WW.*XX+4.*phi.*WW.*XX+(-1).*phi.^2.*WW.* ...

XX+(-3).*WW.^2.*XX+(-2).*phi.*WW.^2.*XX+2.*phi.^2.*WW.^2.*XX+(-2).*phi.* ...

WW.^3.*XX+(-1).*phi.^2.*WW.^3.*XX+(-1).*phi.*WW.*YY+phi.^2.*WW.*YY+ ...

WW.^2.*YY+2.*phi.*WW.^2.*YY+(-4).*phi.^2.*WW.^2.*YY+(-5).*WW.^3.*YY+5.* ...

phi.*WW.^3.*YY+5.*phi.^2.*WW.^3.*YY+(-2).*WW.^4.*YY+(-6).*phi.*WW.^4.* ...

YY+(-2).*phi.^2.*WW.^4.*YY+3.*WW.^2.*XX.*YY+(-5).*phi.*WW.^2.*XX.*YY+ ...

phi.^2.*WW.^2.*XX.*YY+3.*WW.^3.*XX.*YY+4.*phi.*WW.^3.*XX.*YY+(-2).* ...

phi.^2.*WW.^3.*XX.*YY+phi.*WW.^4.*XX.*YY+phi.^2.*WW.^4.*XX.*YY+phi.*WW.* ...

ZZ+(-1).*phi.^2.*WW.*ZZ+(-3).*WW.^2.*ZZ+4.*phi.*WW.^2.*ZZ+2.*phi.^2.* ...

WW.^2.*ZZ+(-3).*WW.^3.*ZZ+(-5).*phi.*WW.^3.*ZZ+(-1).*phi.^2.*WW.^3.*ZZ+ ...

2.*WW.*XX.*ZZ+(-6).*phi.*WW.*XX.*ZZ+2.*phi.^2.*WW.*XX.*ZZ+5.*WW.^2.*XX.* ...

ZZ+5.*phi.*WW.^2.*XX.*ZZ+(-5).*phi.^2.*WW.^2.*XX.*ZZ+(-1).*WW.^3.*XX.* ...

ZZ+2.*phi.*WW.^3.*XX.*ZZ+4.*phi.^2.*WW.^3.*XX.*ZZ+(-1).*phi.*WW.^4.*XX.* ...

ZZ+(-1).*phi.^2.*WW.^4.*XX.*ZZ+(-2).*phi.*WW.^2.*YY.*ZZ+phi.^2.*WW.^2.* ...

YY.*ZZ+3.*WW.^3.*YY.*ZZ+(-2).*phi.*WW.^3.*YY.*ZZ+(-2).*phi.^2.*WW.^3.* ...

YY.*ZZ+3.*WW.^4.*YY.*ZZ+4.*phi.*WW.^4.*YY.*ZZ+phi.^2.*WW.^4.*YY.*ZZ+2.* ...

phi.*WW.*XX.*YY.*ZZ+(-1).*phi.^2.*WW.*XX.*YY.*ZZ+(-3).*WW.^2.*XX.*YY.* ...

ZZ+2.*phi.*WW.^2.*XX.*YY.*ZZ+2.*phi.^2.*WW.^2.*XX.*YY.*ZZ+(-3).*WW.^3.* ...

XX.*YY.*ZZ+(-4).*phi.*WW.^3.*XX.*YY.*ZZ+(-1).*phi.^2.*WW.^3.*XX.*YY.*ZZ) ...

.*((-1).*WW+2.*phi.*WW+(-1).*phi.^2.*WW+(-3).*WW.^2+2.*phi.*WW.^2+ ...

phi.^2.*WW.^2+(-4).*phi.*WW.^3+phi.^2.*WW.^3+(-1).*phi.^2.*WW.^4+XX+(-2) ...

.*phi.*XX+phi.^2.*XX+3.*WW.*XX+(-2).*phi.*WW.*XX+(-1).*phi.^2.*WW.*XX+ ...

4.*phi.*WW.^2.*XX+(-1).*phi.^2.*WW.^2.*XX+phi.^2.*WW.^3.*XX+WW.^2.*YY+( ...

-4).*phi.*WW.^2.*YY+2.*phi.^2.*WW.^2.*YY+3.*WW.^3.*YY+2.*phi.*WW.^3.*YY+ ...

(-4).*phi.^2.*WW.^3.*YY+2.*phi.*WW.^4.*YY+2.*phi.^2.*WW.^4.*YY+(-1).* ...

WW.*XX.*YY+4.*phi.*WW.*XX.*YY+(-2).*phi.^2.*WW.*XX.*YY+(-3).*WW.^2.*XX.* ...

YY+(-2).*phi.*WW.^2.*XX.*YY+4.*phi.^2.*WW.^2.*XX.*YY+(-2).*phi.*WW.^3.* ...

XX.*YY+(-2).*phi.^2.*WW.^3.*XX.*YY+(-2).*phi.*WW.*ZZ+2.*phi.^2.*WW.*ZZ+ ...

3.*WW.^2.*ZZ+(-2).*phi.*WW.^2.*ZZ+(-4).*phi.^2.*WW.^2.*ZZ+WW.^3.*ZZ+4.* ...

phi.*WW.^3.*ZZ+2.*phi.^2.*WW.^3.*ZZ+2.*phi.*XX.*ZZ+(-2).*phi.^2.*XX.*ZZ+ ...

(-3).*WW.*XX.*ZZ+2.*phi.*WW.*XX.*ZZ+4.*phi.^2.*WW.*XX.*ZZ+(-1).*WW.^2.* ...

XX.*ZZ+(-4).*phi.*WW.^2.*XX.*ZZ+(-2).*phi.^2.*WW.^2.*XX.*ZZ+(-1).* ...

phi.^2.*WW.*YY.*ZZ+4.*phi.*WW.^2.*YY.*ZZ+phi.^2.*WW.^2.*YY.*ZZ+(-3).* ...

WW.^3.*YY.*ZZ+(-2).*phi.*WW.^3.*YY.*ZZ+phi.^2.*WW.^3.*YY.*ZZ+(-1).* ...

WW.^4.*YY.*ZZ+(-2).*phi.*WW.^4.*YY.*ZZ+(-1).*phi.^2.*WW.^4.*YY.*ZZ+ ...

phi.^2.*XX.*YY.*ZZ+(-4).*phi.*WW.*XX.*YY.*ZZ+(-1).*phi.^2.*WW.*XX.*YY.* ...

ZZ+3.*WW.^2.*XX.*YY.*ZZ+2.*phi.*WW.^2.*XX.*YY.*ZZ+(-1).*phi.^2.*WW.^2.* ...

XX.*YY.*ZZ+WW.^3.*XX.*YY.*ZZ+2.*phi.*WW.^3.*XX.*YY.*ZZ+phi.^2.*WW.^3.* ...

XX.*YY.*ZZ)+27.*(WW.^4+(-1).*WW.^3.*XX+WW.^3.*YY+(-2).*WW.^4.*YY+WW.^4.* ...

XX.*YY+(-1).*WW.^3.*ZZ+2.*WW.^3.*XX.*ZZ+(-1).*WW.^4.*XX.*ZZ+WW.^4.*YY.* ...

ZZ+(-1).*WW.^3.*XX.*YY.*ZZ).*((-1).*WW+2.*phi.*WW+(-1).*phi.^2.*WW+(-3) ...

.*WW.^2+2.*phi.*WW.^2+phi.^2.*WW.^2+(-4).*phi.*WW.^3+phi.^2.*WW.^3+(-1) ...

.*phi.^2.*WW.^4+XX+(-2).*phi.*XX+phi.^2.*XX+3.*WW.*XX+(-2).*phi.*WW.*XX+ ...

(-1).*phi.^2.*WW.*XX+4.*phi.*WW.^2.*XX+(-1).*phi.^2.*WW.^2.*XX+phi.^2.* ...

WW.^3.*XX+WW.^2.*YY+(-4).*phi.*WW.^2.*YY+2.*phi.^2.*WW.^2.*YY+3.*WW.^3.* ...

YY+2.*phi.*WW.^3.*YY+(-4).*phi.^2.*WW.^3.*YY+2.*phi.*WW.^4.*YY+2.* ...

phi.^2.*WW.^4.*YY+(-1).*WW.*XX.*YY+4.*phi.*WW.*XX.*YY+(-2).*phi.^2.*WW.* ...

XX.*YY+(-3).*WW.^2.*XX.*YY+(-2).*phi.*WW.^2.*XX.*YY+4.*phi.^2.*WW.^2.* ...

XX.*YY+(-2).*phi.*WW.^3.*XX.*YY+(-2).*phi.^2.*WW.^3.*XX.*YY+(-2).*phi.* ...

WW.*ZZ+2.*phi.^2.*WW.*ZZ+3.*WW.^2.*ZZ+(-2).*phi.*WW.^2.*ZZ+(-4).* ...

phi.^2.*WW.^2.*ZZ+WW.^3.*ZZ+4.*phi.*WW.^3.*ZZ+2.*phi.^2.*WW.^3.*ZZ+2.* ...

phi.*XX.*ZZ+(-2).*phi.^2.*XX.*ZZ+(-3).*WW.*XX.*ZZ+2.*phi.*WW.*XX.*ZZ+4.* ...

phi.^2.*WW.*XX.*ZZ+(-1).*WW.^2.*XX.*ZZ+(-4).*phi.*WW.^2.*XX.*ZZ+(-2).* ...

phi.^2.*WW.^2.*XX.*ZZ+(-1).*phi.^2.*WW.*YY.*ZZ+4.*phi.*WW.^2.*YY.*ZZ+ ...

phi.^2.*WW.^2.*YY.*ZZ+(-3).*WW.^3.*YY.*ZZ+(-2).*phi.*WW.^3.*YY.*ZZ+ ...

phi.^2.*WW.^3.*YY.*ZZ+(-1).*WW.^4.*YY.*ZZ+(-2).*phi.*WW.^4.*YY.*ZZ+(-1) ...

.*phi.^2.*WW.^4.*YY.*ZZ+phi.^2.*XX.*YY.*ZZ+(-4).*phi.*WW.*XX.*YY.*ZZ+( ...

-1).*phi.^2.*WW.*XX.*YY.*ZZ+3.*WW.^2.*XX.*YY.*ZZ+2.*phi.*WW.^2.*XX.*YY.* ...

ZZ+(-1).*phi.^2.*WW.^2.*XX.*YY.*ZZ+WW.^3.*XX.*YY.*ZZ+2.*phi.*WW.^3.*XX.* ...

YY.*ZZ+phi.^2.*WW.^3.*XX.*YY.*ZZ).^2).^2).^(1/2)).^(-1/3)+(-1/3).*2.^( ...

-1/3).*(WW+(-2).*phi.*WW+phi.^2.*WW+2.*phi.*WW.^2+(-2).*phi.^2.*WW.^2+ ...

phi.^2.*WW.^3+(-1).*XX+2.*phi.*XX+(-1).*phi.^2.*XX+(-2).*phi.*WW.*XX+2.* ...

phi.^2.*WW.*XX+(-1).*phi.^2.*WW.^2.*XX+phi.*WW.*YY+(-1).*phi.^2.*WW.*YY+ ...

(-1).*WW.^2.*YY+2.*phi.^2.*WW.^2.*YY+(-1).*phi.*WW.^3.*YY+(-1).*phi.^2.* ...

WW.^3.*YY+(-1).*phi.*XX.*YY+phi.^2.*XX.*YY+WW.*XX.*YY+(-2).*phi.^2.*WW.* ...

XX.*YY+phi.*WW.^2.*XX.*YY+phi.^2.*WW.^2.*XX.*YY+phi.*WW.*ZZ+(-1).* ...

phi.^2.*WW.*ZZ+(-1).*WW.^2.*ZZ+2.*phi.^2.*WW.^2.*ZZ+(-1).*phi.*WW.^3.* ...

ZZ+(-1).*phi.^2.*WW.^3.*ZZ+(-1).*phi.*XX.*ZZ+phi.^2.*XX.*ZZ+WW.*XX.*ZZ+( ...

-2).*phi.^2.*WW.*XX.*ZZ+phi.*WW.^2.*XX.*ZZ+phi.^2.*WW.^2.*XX.*ZZ+ ...

phi.^2.*WW.*YY.*ZZ+(-2).*phi.*WW.^2.*YY.*ZZ+(-2).*phi.^2.*WW.^2.*YY.*ZZ+ ...

WW.^3.*YY.*ZZ+2.*phi.*WW.^3.*YY.*ZZ+phi.^2.*WW.^3.*YY.*ZZ+(-1).*phi.^2.* ...

XX.*YY.*ZZ+2.*phi.*WW.*XX.*YY.*ZZ+2.*phi.^2.*WW.*XX.*YY.*ZZ+(-1).* ...

WW.^2.*XX.*YY.*ZZ+(-2).*phi.*WW.^2.*XX.*YY.*ZZ+(-1).*phi.^2.*WW.^2.*XX.* ...

YY.*ZZ).^(-1).*(27.*(WW+(-2).*phi.*WW+phi.^2.*WW+2.*phi.*WW.^2+(-2).* ...

phi.^2.*WW.^2+phi.^2.*WW.^3+(-1).*XX+2.*phi.*XX+(-1).*phi.^2.*XX+(-2).* ...

phi.*WW.*XX+2.*phi.^2.*WW.*XX+(-1).*phi.^2.*WW.^2.*XX+phi.*WW.*YY+(-1).* ...

phi.^2.*WW.*YY+(-1).*WW.^2.*YY+2.*phi.^2.*WW.^2.*YY+(-1).*phi.*WW.^3.* ...

YY+(-1).*phi.^2.*WW.^3.*YY+(-1).*phi.*XX.*YY+phi.^2.*XX.*YY+WW.*XX.*YY+( ...

-2).*phi.^2.*WW.*XX.*YY+phi.*WW.^2.*XX.*YY+phi.^2.*WW.^2.*XX.*YY+phi.* ...

WW.*ZZ+(-1).*phi.^2.*WW.*ZZ+(-1).*WW.^2.*ZZ+2.*phi.^2.*WW.^2.*ZZ+(-1).* ...

phi.*WW.^3.*ZZ+(-1).*phi.^2.*WW.^3.*ZZ+(-1).*phi.*XX.*ZZ+phi.^2.*XX.*ZZ+ ...

WW.*XX.*ZZ+(-2).*phi.^2.*WW.*XX.*ZZ+phi.*WW.^2.*XX.*ZZ+phi.^2.*WW.^2.* ...

XX.*ZZ+phi.^2.*WW.*YY.*ZZ+(-2).*phi.*WW.^2.*YY.*ZZ+(-2).*phi.^2.*WW.^2.* ...

YY.*ZZ+WW.^3.*YY.*ZZ+2.*phi.*WW.^3.*YY.*ZZ+phi.^2.*WW.^3.*YY.*ZZ+(-1).* ...

phi.^2.*XX.*YY.*ZZ+2.*phi.*WW.*XX.*YY.*ZZ+2.*phi.^2.*WW.*XX.*YY.*ZZ+(-1) ...

.*WW.^2.*XX.*YY.*ZZ+(-2).*phi.*WW.^2.*XX.*YY.*ZZ+(-1).*phi.^2.*WW.^2.* ...

XX.*YY.*ZZ).*((-3).*WW.^3+2.*phi.*WW.^3+(-1).*WW.^4+(-2).*phi.*WW.^4+3.* ...

WW.^2.*XX+(-2).*phi.*WW.^2.*XX+WW.^3.*XX+2.*phi.*WW.^3.*XX+(-1).*WW.^2.* ...

YY+2.*phi.*WW.^2.*YY+WW.^3.*YY+(-6).*phi.*WW.^3.*YY+4.*WW.^4.*YY+4.* ...

phi.*WW.^4.*YY+(-3).*WW.^3.*XX.*YY+2.*phi.*WW.^3.*XX.*YY+(-1).*WW.^4.* ...

XX.*YY+(-2).*phi.*WW.^4.*XX.*YY+WW.^2.*ZZ+(-2).*phi.*WW.^2.*ZZ+3.* ...

WW.^3.*ZZ+2.*phi.*WW.^3.*ZZ+(-4).*WW.^2.*XX.*ZZ+4.*phi.*WW.^2.*XX.*ZZ+( ...

-1).*WW.^3.*XX.*ZZ+(-6).*phi.*WW.^3.*XX.*ZZ+WW.^4.*XX.*ZZ+2.*phi.* ...

WW.^4.*XX.*ZZ+(-1).*WW.^3.*YY.*ZZ+2.*phi.*WW.^3.*YY.*ZZ+(-3).*WW.^4.* ...

YY.*ZZ+(-2).*phi.*WW.^4.*YY.*ZZ+WW.^2.*XX.*YY.*ZZ+(-2).*phi.*WW.^2.*XX.* ...

YY.*ZZ+3.*WW.^3.*XX.*YY.*ZZ+2.*phi.*WW.^3.*XX.*YY.*ZZ).^2+(-72).*(WW+( ...

-2).*phi.*WW+phi.^2.*WW+2.*phi.*WW.^2+(-2).*phi.^2.*WW.^2+phi.^2.*WW.^3+ ...

(-1).*XX+2.*phi.*XX+(-1).*phi.^2.*XX+(-2).*phi.*WW.*XX+2.*phi.^2.*WW.* ...

XX+(-1).*phi.^2.*WW.^2.*XX+phi.*WW.*YY+(-1).*phi.^2.*WW.*YY+(-1).* ...

WW.^2.*YY+2.*phi.^2.*WW.^2.*YY+(-1).*phi.*WW.^3.*YY+(-1).*phi.^2.* ...

WW.^3.*YY+(-1).*phi.*XX.*YY+phi.^2.*XX.*YY+WW.*XX.*YY+(-2).*phi.^2.*WW.* ...

XX.*YY+phi.*WW.^2.*XX.*YY+phi.^2.*WW.^2.*XX.*YY+phi.*WW.*ZZ+(-1).* ...

phi.^2.*WW.*ZZ+(-1).*WW.^2.*ZZ+2.*phi.^2.*WW.^2.*ZZ+(-1).*phi.*WW.^3.* ...

ZZ+(-1).*phi.^2.*WW.^3.*ZZ+(-1).*phi.*XX.*ZZ+phi.^2.*XX.*ZZ+WW.*XX.*ZZ+( ...

-2).*phi.^2.*WW.*XX.*ZZ+phi.*WW.^2.*XX.*ZZ+phi.^2.*WW.^2.*XX.*ZZ+ ...

phi.^2.*WW.*YY.*ZZ+(-2).*phi.*WW.^2.*YY.*ZZ+(-2).*phi.^2.*WW.^2.*YY.*ZZ+ ...

WW.^3.*YY.*ZZ+2.*phi.*WW.^3.*YY.*ZZ+phi.^2.*WW.^3.*YY.*ZZ+(-1).*phi.^2.* ...

XX.*YY.*ZZ+2.*phi.*WW.*XX.*YY.*ZZ+2.*phi.^2.*WW.*XX.*YY.*ZZ+(-1).* ...

WW.^2.*XX.*YY.*ZZ+(-2).*phi.*WW.^2.*XX.*YY.*ZZ+(-1).*phi.^2.*WW.^2.*XX.* ...

YY.*ZZ).*(WW.^4+(-1).*WW.^3.*XX+WW.^3.*YY+(-2).*WW.^4.*YY+WW.^4.*XX.*YY+ ...

(-1).*WW.^3.*ZZ+2.*WW.^3.*XX.*ZZ+(-1).*WW.^4.*XX.*ZZ+WW.^4.*YY.*ZZ+(-1) ...

.*WW.^3.*XX.*YY.*ZZ).*(3.*WW.^2+(-4).*phi.*WW.^2+phi.^2.*WW.^2+3.*WW.^3+ ...

2.*phi.*WW.^3+(-2).*phi.^2.*WW.^3+2.*phi.*WW.^4+phi.^2.*WW.^4+(-3).*WW.* ...

XX+4.*phi.*WW.*XX+(-1).*phi.^2.*WW.*XX+(-3).*WW.^2.*XX+(-2).*phi.* ...

WW.^2.*XX+2.*phi.^2.*WW.^2.*XX+(-2).*phi.*WW.^3.*XX+(-1).*phi.^2.* ...

WW.^3.*XX+(-1).*phi.*WW.*YY+phi.^2.*WW.*YY+WW.^2.*YY+2.*phi.*WW.^2.*YY+( ...

-4).*phi.^2.*WW.^2.*YY+(-5).*WW.^3.*YY+5.*phi.*WW.^3.*YY+5.*phi.^2.* ...

WW.^3.*YY+(-2).*WW.^4.*YY+(-6).*phi.*WW.^4.*YY+(-2).*phi.^2.*WW.^4.*YY+ ...

3.*WW.^2.*XX.*YY+(-5).*phi.*WW.^2.*XX.*YY+phi.^2.*WW.^2.*XX.*YY+3.* ...

WW.^3.*XX.*YY+4.*phi.*WW.^3.*XX.*YY+(-2).*phi.^2.*WW.^3.*XX.*YY+phi.* ...

WW.^4.*XX.*YY+phi.^2.*WW.^4.*XX.*YY+phi.*WW.*ZZ+(-1).*phi.^2.*WW.*ZZ+( ...

-3).*WW.^2.*ZZ+4.*phi.*WW.^2.*ZZ+2.*phi.^2.*WW.^2.*ZZ+(-3).*WW.^3.*ZZ+( ...

-5).*phi.*WW.^3.*ZZ+(-1).*phi.^2.*WW.^3.*ZZ+2.*WW.*XX.*ZZ+(-6).*phi.* ...

WW.*XX.*ZZ+2.*phi.^2.*WW.*XX.*ZZ+5.*WW.^2.*XX.*ZZ+5.*phi.*WW.^2.*XX.*ZZ+ ...

(-5).*phi.^2.*WW.^2.*XX.*ZZ+(-1).*WW.^3.*XX.*ZZ+2.*phi.*WW.^3.*XX.*ZZ+ ...

4.*phi.^2.*WW.^3.*XX.*ZZ+(-1).*phi.*WW.^4.*XX.*ZZ+(-1).*phi.^2.*WW.^4.* ...

XX.*ZZ+(-2).*phi.*WW.^2.*YY.*ZZ+phi.^2.*WW.^2.*YY.*ZZ+3.*WW.^3.*YY.*ZZ+( ...

-2).*phi.*WW.^3.*YY.*ZZ+(-2).*phi.^2.*WW.^3.*YY.*ZZ+3.*WW.^4.*YY.*ZZ+4.* ...

phi.*WW.^4.*YY.*ZZ+phi.^2.*WW.^4.*YY.*ZZ+2.*phi.*WW.*XX.*YY.*ZZ+(-1).* ...

phi.^2.*WW.*XX.*YY.*ZZ+(-3).*WW.^2.*XX.*YY.*ZZ+2.*phi.*WW.^2.*XX.*YY.* ...

ZZ+2.*phi.^2.*WW.^2.*XX.*YY.*ZZ+(-3).*WW.^3.*XX.*YY.*ZZ+(-4).*phi.* ...

WW.^3.*XX.*YY.*ZZ+(-1).*phi.^2.*WW.^3.*XX.*YY.*ZZ)+2.*(3.*WW.^2+(-4).* ...

phi.*WW.^2+phi.^2.*WW.^2+3.*WW.^3+2.*phi.*WW.^3+(-2).*phi.^2.*WW.^3+2.* ...

phi.*WW.^4+phi.^2.*WW.^4+(-3).*WW.*XX+4.*phi.*WW.*XX+(-1).*phi.^2.*WW.* ...

XX+(-3).*WW.^2.*XX+(-2).*phi.*WW.^2.*XX+2.*phi.^2.*WW.^2.*XX+(-2).*phi.* ...

WW.^3.*XX+(-1).*phi.^2.*WW.^3.*XX+(-1).*phi.*WW.*YY+phi.^2.*WW.*YY+ ...

WW.^2.*YY+2.*phi.*WW.^2.*YY+(-4).*phi.^2.*WW.^2.*YY+(-5).*WW.^3.*YY+5.* ...

phi.*WW.^3.*YY+5.*phi.^2.*WW.^3.*YY+(-2).*WW.^4.*YY+(-6).*phi.*WW.^4.* ...

YY+(-2).*phi.^2.*WW.^4.*YY+3.*WW.^2.*XX.*YY+(-5).*phi.*WW.^2.*XX.*YY+ ...

phi.^2.*WW.^2.*XX.*YY+3.*WW.^3.*XX.*YY+4.*phi.*WW.^3.*XX.*YY+(-2).* ...

phi.^2.*WW.^3.*XX.*YY+phi.*WW.^4.*XX.*YY+phi.^2.*WW.^4.*XX.*YY+phi.*WW.* ...

ZZ+(-1).*phi.^2.*WW.*ZZ+(-3).*WW.^2.*ZZ+4.*phi.*WW.^2.*ZZ+2.*phi.^2.* ...

WW.^2.*ZZ+(-3).*WW.^3.*ZZ+(-5).*phi.*WW.^3.*ZZ+(-1).*phi.^2.*WW.^3.*ZZ+ ...

2.*WW.*XX.*ZZ+(-6).*phi.*WW.*XX.*ZZ+2.*phi.^2.*WW.*XX.*ZZ+5.*WW.^2.*XX.* ...

ZZ+5.*phi.*WW.^2.*XX.*ZZ+(-5).*phi.^2.*WW.^2.*XX.*ZZ+(-1).*WW.^3.*XX.* ...

ZZ+2.*phi.*WW.^3.*XX.*ZZ+4.*phi.^2.*WW.^3.*XX.*ZZ+(-1).*phi.*WW.^4.*XX.* ...

ZZ+(-1).*phi.^2.*WW.^4.*XX.*ZZ+(-2).*phi.*WW.^2.*YY.*ZZ+phi.^2.*WW.^2.* ...

YY.*ZZ+3.*WW.^3.*YY.*ZZ+(-2).*phi.*WW.^3.*YY.*ZZ+(-2).*phi.^2.*WW.^3.* ...

YY.*ZZ+3.*WW.^4.*YY.*ZZ+4.*phi.*WW.^4.*YY.*ZZ+phi.^2.*WW.^4.*YY.*ZZ+2.* ...

phi.*WW.*XX.*YY.*ZZ+(-1).*phi.^2.*WW.*XX.*YY.*ZZ+(-3).*WW.^2.*XX.*YY.* ...

ZZ+2.*phi.*WW.^2.*XX.*YY.*ZZ+2.*phi.^2.*WW.^2.*XX.*YY.*ZZ+(-3).*WW.^3.* ...

XX.*YY.*ZZ+(-4).*phi.*WW.^3.*XX.*YY.*ZZ+(-1).*phi.^2.*WW.^3.*XX.*YY.*ZZ) ...

.^3+(-9).*((-3).*WW.^3+2.*phi.*WW.^3+(-1).*WW.^4+(-2).*phi.*WW.^4+3.* ...

WW.^2.*XX+(-2).*phi.*WW.^2.*XX+WW.^3.*XX+2.*phi.*WW.^3.*XX+(-1).*WW.^2.* ...

YY+2.*phi.*WW.^2.*YY+WW.^3.*YY+(-6).*phi.*WW.^3.*YY+4.*WW.^4.*YY+4.* ...

phi.*WW.^4.*YY+(-3).*WW.^3.*XX.*YY+2.*phi.*WW.^3.*XX.*YY+(-1).*WW.^4.* ...

XX.*YY+(-2).*phi.*WW.^4.*XX.*YY+WW.^2.*ZZ+(-2).*phi.*WW.^2.*ZZ+3.* ...

WW.^3.*ZZ+2.*phi.*WW.^3.*ZZ+(-4).*WW.^2.*XX.*ZZ+4.*phi.*WW.^2.*XX.*ZZ+( ...

-1).*WW.^3.*XX.*ZZ+(-6).*phi.*WW.^3.*XX.*ZZ+WW.^4.*XX.*ZZ+2.*phi.* ...

WW.^4.*XX.*ZZ+(-1).*WW.^3.*YY.*ZZ+2.*phi.*WW.^3.*YY.*ZZ+(-3).*WW.^4.* ...

YY.*ZZ+(-2).*phi.*WW.^4.*YY.*ZZ+WW.^2.*XX.*YY.*ZZ+(-2).*phi.*WW.^2.*XX.* ...

YY.*ZZ+3.*WW.^3.*XX.*YY.*ZZ+2.*phi.*WW.^3.*XX.*YY.*ZZ).*(3.*WW.^2+(-4).* ...

phi.*WW.^2+phi.^2.*WW.^2+3.*WW.^3+2.*phi.*WW.^3+(-2).*phi.^2.*WW.^3+2.* ...

phi.*WW.^4+phi.^2.*WW.^4+(-3).*WW.*XX+4.*phi.*WW.*XX+(-1).*phi.^2.*WW.* ...

XX+(-3).*WW.^2.*XX+(-2).*phi.*WW.^2.*XX+2.*phi.^2.*WW.^2.*XX+(-2).*phi.* ...

WW.^3.*XX+(-1).*phi.^2.*WW.^3.*XX+(-1).*phi.*WW.*YY+phi.^2.*WW.*YY+ ...

WW.^2.*YY+2.*phi.*WW.^2.*YY+(-4).*phi.^2.*WW.^2.*YY+(-5).*WW.^3.*YY+5.* ...

phi.*WW.^3.*YY+5.*phi.^2.*WW.^3.*YY+(-2).*WW.^4.*YY+(-6).*phi.*WW.^4.* ...

YY+(-2).*phi.^2.*WW.^4.*YY+3.*WW.^2.*XX.*YY+(-5).*phi.*WW.^2.*XX.*YY+ ...

phi.^2.*WW.^2.*XX.*YY+3.*WW.^3.*XX.*YY+4.*phi.*WW.^3.*XX.*YY+(-2).* ...

phi.^2.*WW.^3.*XX.*YY+phi.*WW.^4.*XX.*YY+phi.^2.*WW.^4.*XX.*YY+phi.*WW.* ...

ZZ+(-1).*phi.^2.*WW.*ZZ+(-3).*WW.^2.*ZZ+4.*phi.*WW.^2.*ZZ+2.*phi.^2.* ...

WW.^2.*ZZ+(-3).*WW.^3.*ZZ+(-5).*phi.*WW.^3.*ZZ+(-1).*phi.^2.*WW.^3.*ZZ+ ...

2.*WW.*XX.*ZZ+(-6).*phi.*WW.*XX.*ZZ+2.*phi.^2.*WW.*XX.*ZZ+5.*WW.^2.*XX.* ...

ZZ+5.*phi.*WW.^2.*XX.*ZZ+(-5).*phi.^2.*WW.^2.*XX.*ZZ+(-1).*WW.^3.*XX.* ...

ZZ+2.*phi.*WW.^3.*XX.*ZZ+4.*phi.^2.*WW.^3.*XX.*ZZ+(-1).*phi.*WW.^4.*XX.* ...

ZZ+(-1).*phi.^2.*WW.^4.*XX.*ZZ+(-2).*phi.*WW.^2.*YY.*ZZ+phi.^2.*WW.^2.* ...

YY.*ZZ+3.*WW.^3.*YY.*ZZ+(-2).*phi.*WW.^3.*YY.*ZZ+(-2).*phi.^2.*WW.^3.* ...

YY.*ZZ+3.*WW.^4.*YY.*ZZ+4.*phi.*WW.^4.*YY.*ZZ+phi.^2.*WW.^4.*YY.*ZZ+2.* ...

phi.*WW.*XX.*YY.*ZZ+(-1).*phi.^2.*WW.*XX.*YY.*ZZ+(-3).*WW.^2.*XX.*YY.* ...

ZZ+2.*phi.*WW.^2.*XX.*YY.*ZZ+2.*phi.^2.*WW.^2.*XX.*YY.*ZZ+(-3).*WW.^3.* ...

XX.*YY.*ZZ+(-4).*phi.*WW.^3.*XX.*YY.*ZZ+(-1).*phi.^2.*WW.^3.*XX.*YY.*ZZ) ...

.*((-1).*WW+2.*phi.*WW+(-1).*phi.^2.*WW+(-3).*WW.^2+2.*phi.*WW.^2+ ...

phi.^2.*WW.^2+(-4).*phi.*WW.^3+phi.^2.*WW.^3+(-1).*phi.^2.*WW.^4+XX+(-2) ...

.*phi.*XX+phi.^2.*XX+3.*WW.*XX+(-2).*phi.*WW.*XX+(-1).*phi.^2.*WW.*XX+ ...

4.*phi.*WW.^2.*XX+(-1).*phi.^2.*WW.^2.*XX+phi.^2.*WW.^3.*XX+WW.^2.*YY+( ...

-4).*phi.*WW.^2.*YY+2.*phi.^2.*WW.^2.*YY+3.*WW.^3.*YY+2.*phi.*WW.^3.*YY+ ...

(-4).*phi.^2.*WW.^3.*YY+2.*phi.*WW.^4.*YY+2.*phi.^2.*WW.^4.*YY+(-1).* ...

WW.*XX.*YY+4.*phi.*WW.*XX.*YY+(-2).*phi.^2.*WW.*XX.*YY+(-3).*WW.^2.*XX.* ...

YY+(-2).*phi.*WW.^2.*XX.*YY+4.*phi.^2.*WW.^2.*XX.*YY+(-2).*phi.*WW.^3.* ...

XX.*YY+(-2).*phi.^2.*WW.^3.*XX.*YY+(-2).*phi.*WW.*ZZ+2.*phi.^2.*WW.*ZZ+ ...

3.*WW.^2.*ZZ+(-2).*phi.*WW.^2.*ZZ+(-4).*phi.^2.*WW.^2.*ZZ+WW.^3.*ZZ+4.* ...

phi.*WW.^3.*ZZ+2.*phi.^2.*WW.^3.*ZZ+2.*phi.*XX.*ZZ+(-2).*phi.^2.*XX.*ZZ+ ...

(-3).*WW.*XX.*ZZ+2.*phi.*WW.*XX.*ZZ+4.*phi.^2.*WW.*XX.*ZZ+(-1).*WW.^2.* ...

XX.*ZZ+(-4).*phi.*WW.^2.*XX.*ZZ+(-2).*phi.^2.*WW.^2.*XX.*ZZ+(-1).* ...

phi.^2.*WW.*YY.*ZZ+4.*phi.*WW.^2.*YY.*ZZ+phi.^2.*WW.^2.*YY.*ZZ+(-3).* ...

WW.^3.*YY.*ZZ+(-2).*phi.*WW.^3.*YY.*ZZ+phi.^2.*WW.^3.*YY.*ZZ+(-1).* ...

WW.^4.*YY.*ZZ+(-2).*phi.*WW.^4.*YY.*ZZ+(-1).*phi.^2.*WW.^4.*YY.*ZZ+ ...

phi.^2.*XX.*YY.*ZZ+(-4).*phi.*WW.*XX.*YY.*ZZ+(-1).*phi.^2.*WW.*XX.*YY.* ...

ZZ+3.*WW.^2.*XX.*YY.*ZZ+2.*phi.*WW.^2.*XX.*YY.*ZZ+(-1).*phi.^2.*WW.^2.* ...

XX.*YY.*ZZ+WW.^3.*XX.*YY.*ZZ+2.*phi.*WW.^3.*XX.*YY.*ZZ+phi.^2.*WW.^3.* ...

XX.*YY.*ZZ)+27.*(WW.^4+(-1).*WW.^3.*XX+WW.^3.*YY+(-2).*WW.^4.*YY+WW.^4.* ...

XX.*YY+(-1).*WW.^3.*ZZ+2.*WW.^3.*XX.*ZZ+(-1).*WW.^4.*XX.*ZZ+WW.^4.*YY.* ...

ZZ+(-1).*WW.^3.*XX.*YY.*ZZ).*((-1).*WW+2.*phi.*WW+(-1).*phi.^2.*WW+(-3) ...

.*WW.^2+2.*phi.*WW.^2+phi.^2.*WW.^2+(-4).*phi.*WW.^3+phi.^2.*WW.^3+(-1) ...

.*phi.^2.*WW.^4+XX+(-2).*phi.*XX+phi.^2.*XX+3.*WW.*XX+(-2).*phi.*WW.*XX+ ...

(-1).*phi.^2.*WW.*XX+4.*phi.*WW.^2.*XX+(-1).*phi.^2.*WW.^2.*XX+phi.^2.* ...

WW.^3.*XX+WW.^2.*YY+(-4).*phi.*WW.^2.*YY+2.*phi.^2.*WW.^2.*YY+3.*WW.^3.* ...

YY+2.*phi.*WW.^3.*YY+(-4).*phi.^2.*WW.^3.*YY+2.*phi.*WW.^4.*YY+2.* ...

phi.^2.*WW.^4.*YY+(-1).*WW.*XX.*YY+4.*phi.*WW.*XX.*YY+(-2).*phi.^2.*WW.* ...

XX.*YY+(-3).*WW.^2.*XX.*YY+(-2).*phi.*WW.^2.*XX.*YY+4.*phi.^2.*WW.^2.* ...

XX.*YY+(-2).*phi.*WW.^3.*XX.*YY+(-2).*phi.^2.*WW.^3.*XX.*YY+(-2).*phi.* ...

WW.*ZZ+2.*phi.^2.*WW.*ZZ+3.*WW.^2.*ZZ+(-2).*phi.*WW.^2.*ZZ+(-4).* ...

phi.^2.*WW.^2.*ZZ+WW.^3.*ZZ+4.*phi.*WW.^3.*ZZ+2.*phi.^2.*WW.^3.*ZZ+2.* ...

phi.*XX.*ZZ+(-2).*phi.^2.*XX.*ZZ+(-3).*WW.*XX.*ZZ+2.*phi.*WW.*XX.*ZZ+4.* ...

phi.^2.*WW.*XX.*ZZ+(-1).*WW.^2.*XX.*ZZ+(-4).*phi.*WW.^2.*XX.*ZZ+(-2).* ...

phi.^2.*WW.^2.*XX.*ZZ+(-1).*phi.^2.*WW.*YY.*ZZ+4.*phi.*WW.^2.*YY.*ZZ+ ...

phi.^2.*WW.^2.*YY.*ZZ+(-3).*WW.^3.*YY.*ZZ+(-2).*phi.*WW.^3.*YY.*ZZ+ ...

phi.^2.*WW.^3.*YY.*ZZ+(-1).*WW.^4.*YY.*ZZ+(-2).*phi.*WW.^4.*YY.*ZZ+(-1) ...

.*phi.^2.*WW.^4.*YY.*ZZ+phi.^2.*XX.*YY.*ZZ+(-4).*phi.*WW.*XX.*YY.*ZZ+( ...

-1).*phi.^2.*WW.*XX.*YY.*ZZ+3.*WW.^2.*XX.*YY.*ZZ+2.*phi.*WW.^2.*XX.*YY.* ...

ZZ+(-1).*phi.^2.*WW.^2.*XX.*YY.*ZZ+WW.^3.*XX.*YY.*ZZ+2.*phi.*WW.^3.*XX.* ...

YY.*ZZ+phi.^2.*WW.^3.*XX.*YY.*ZZ).^2+((-4).*(12.*(WW+(-2).*phi.*WW+ ...

phi.^2.*WW+2.*phi.*WW.^2+(-2).*phi.^2.*WW.^2+phi.^2.*WW.^3+(-1).*XX+2.* ...

phi.*XX+(-1).*phi.^2.*XX+(-2).*phi.*WW.*XX+2.*phi.^2.*WW.*XX+(-1).* ...

phi.^2.*WW.^2.*XX+phi.*WW.*YY+(-1).*phi.^2.*WW.*YY+(-1).*WW.^2.*YY+2.* ...

phi.^2.*WW.^2.*YY+(-1).*phi.*WW.^3.*YY+(-1).*phi.^2.*WW.^3.*YY+(-1).* ...

phi.*XX.*YY+phi.^2.*XX.*YY+WW.*XX.*YY+(-2).*phi.^2.*WW.*XX.*YY+phi.* ...

WW.^2.*XX.*YY+phi.^2.*WW.^2.*XX.*YY+phi.*WW.*ZZ+(-1).*phi.^2.*WW.*ZZ+( ...

-1).*WW.^2.*ZZ+2.*phi.^2.*WW.^2.*ZZ+(-1).*phi.*WW.^3.*ZZ+(-1).*phi.^2.* ...

WW.^3.*ZZ+(-1).*phi.*XX.*ZZ+phi.^2.*XX.*ZZ+WW.*XX.*ZZ+(-2).*phi.^2.*WW.* ...

XX.*ZZ+phi.*WW.^2.*XX.*ZZ+phi.^2.*WW.^2.*XX.*ZZ+phi.^2.*WW.*YY.*ZZ+(-2) ...

.*phi.*WW.^2.*YY.*ZZ+(-2).*phi.^2.*WW.^2.*YY.*ZZ+WW.^3.*YY.*ZZ+2.*phi.* ...

WW.^3.*YY.*ZZ+phi.^2.*WW.^3.*YY.*ZZ+(-1).*phi.^2.*XX.*YY.*ZZ+2.*phi.* ...

WW.*XX.*YY.*ZZ+2.*phi.^2.*WW.*XX.*YY.*ZZ+(-1).*WW.^2.*XX.*YY.*ZZ+(-2).* ...

phi.*WW.^2.*XX.*YY.*ZZ+(-1).*phi.^2.*WW.^2.*XX.*YY.*ZZ).*(WW.^4+(-1).* ...

WW.^3.*XX+WW.^3.*YY+(-2).*WW.^4.*YY+WW.^4.*XX.*YY+(-1).*WW.^3.*ZZ+2.* ...

WW.^3.*XX.*ZZ+(-1).*WW.^4.*XX.*ZZ+WW.^4.*YY.*ZZ+(-1).*WW.^3.*XX.*YY.*ZZ) ...

+(3.*WW.^2+(-4).*phi.*WW.^2+phi.^2.*WW.^2+3.*WW.^3+2.*phi.*WW.^3+(-2).* ...

phi.^2.*WW.^3+2.*phi.*WW.^4+phi.^2.*WW.^4+(-3).*WW.*XX+4.*phi.*WW.*XX+( ...

-1).*phi.^2.*WW.*XX+(-3).*WW.^2.*XX+(-2).*phi.*WW.^2.*XX+2.*phi.^2.* ...

WW.^2.*XX+(-2).*phi.*WW.^3.*XX+(-1).*phi.^2.*WW.^3.*XX+(-1).*phi.*WW.* ...

YY+phi.^2.*WW.*YY+WW.^2.*YY+2.*phi.*WW.^2.*YY+(-4).*phi.^2.*WW.^2.*YY+( ...

-5).*WW.^3.*YY+5.*phi.*WW.^3.*YY+5.*phi.^2.*WW.^3.*YY+(-2).*WW.^4.*YY+( ...

-6).*phi.*WW.^4.*YY+(-2).*phi.^2.*WW.^4.*YY+3.*WW.^2.*XX.*YY+(-5).*phi.* ...

WW.^2.*XX.*YY+phi.^2.*WW.^2.*XX.*YY+3.*WW.^3.*XX.*YY+4.*phi.*WW.^3.*XX.* ...

YY+(-2).*phi.^2.*WW.^3.*XX.*YY+phi.*WW.^4.*XX.*YY+phi.^2.*WW.^4.*XX.*YY+ ...

phi.*WW.*ZZ+(-1).*phi.^2.*WW.*ZZ+(-3).*WW.^2.*ZZ+4.*phi.*WW.^2.*ZZ+2.* ...

phi.^2.*WW.^2.*ZZ+(-3).*WW.^3.*ZZ+(-5).*phi.*WW.^3.*ZZ+(-1).*phi.^2.* ...

WW.^3.*ZZ+2.*WW.*XX.*ZZ+(-6).*phi.*WW.*XX.*ZZ+2.*phi.^2.*WW.*XX.*ZZ+5.* ...

WW.^2.*XX.*ZZ+5.*phi.*WW.^2.*XX.*ZZ+(-5).*phi.^2.*WW.^2.*XX.*ZZ+(-1).* ...

WW.^3.*XX.*ZZ+2.*phi.*WW.^3.*XX.*ZZ+4.*phi.^2.*WW.^3.*XX.*ZZ+(-1).*phi.* ...

WW.^4.*XX.*ZZ+(-1).*phi.^2.*WW.^4.*XX.*ZZ+(-2).*phi.*WW.^2.*YY.*ZZ+ ...

phi.^2.*WW.^2.*YY.*ZZ+3.*WW.^3.*YY.*ZZ+(-2).*phi.*WW.^3.*YY.*ZZ+(-2).* ...

phi.^2.*WW.^3.*YY.*ZZ+3.*WW.^4.*YY.*ZZ+4.*phi.*WW.^4.*YY.*ZZ+phi.^2.* ...

WW.^4.*YY.*ZZ+2.*phi.*WW.*XX.*YY.*ZZ+(-1).*phi.^2.*WW.*XX.*YY.*ZZ+(-3).* ...

WW.^2.*XX.*YY.*ZZ+2.*phi.*WW.^2.*XX.*YY.*ZZ+2.*phi.^2.*WW.^2.*XX.*YY.* ...

ZZ+(-3).*WW.^3.*XX.*YY.*ZZ+(-4).*phi.*WW.^3.*XX.*YY.*ZZ+(-1).*phi.^2.* ...

WW.^3.*XX.*YY.*ZZ).^2+(-3).*((-3).*WW.^3+2.*phi.*WW.^3+(-1).*WW.^4+(-2) ...

.*phi.*WW.^4+3.*WW.^2.*XX+(-2).*phi.*WW.^2.*XX+WW.^3.*XX+2.*phi.*WW.^3.* ...

XX+(-1).*WW.^2.*YY+2.*phi.*WW.^2.*YY+WW.^3.*YY+(-6).*phi.*WW.^3.*YY+4.* ...

WW.^4.*YY+4.*phi.*WW.^4.*YY+(-3).*WW.^3.*XX.*YY+2.*phi.*WW.^3.*XX.*YY+( ...

-1).*WW.^4.*XX.*YY+(-2).*phi.*WW.^4.*XX.*YY+WW.^2.*ZZ+(-2).*phi.*WW.^2.* ...

ZZ+3.*WW.^3.*ZZ+2.*phi.*WW.^3.*ZZ+(-4).*WW.^2.*XX.*ZZ+4.*phi.*WW.^2.* ...

XX.*ZZ+(-1).*WW.^3.*XX.*ZZ+(-6).*phi.*WW.^3.*XX.*ZZ+WW.^4.*XX.*ZZ+2.* ...

phi.*WW.^4.*XX.*ZZ+(-1).*WW.^3.*YY.*ZZ+2.*phi.*WW.^3.*YY.*ZZ+(-3).* ...

WW.^4.*YY.*ZZ+(-2).*phi.*WW.^4.*YY.*ZZ+WW.^2.*XX.*YY.*ZZ+(-2).*phi.* ...

WW.^2.*XX.*YY.*ZZ+3.*WW.^3.*XX.*YY.*ZZ+2.*phi.*WW.^3.*XX.*YY.*ZZ).*((-1) ...

.*WW+2.*phi.*WW+(-1).*phi.^2.*WW+(-3).*WW.^2+2.*phi.*WW.^2+phi.^2.* ...

WW.^2+(-4).*phi.*WW.^3+phi.^2.*WW.^3+(-1).*phi.^2.*WW.^4+XX+(-2).*phi.* ...

XX+phi.^2.*XX+3.*WW.*XX+(-2).*phi.*WW.*XX+(-1).*phi.^2.*WW.*XX+4.*phi.* ...

WW.^2.*XX+(-1).*phi.^2.*WW.^2.*XX+phi.^2.*WW.^3.*XX+WW.^2.*YY+(-4).* ...

phi.*WW.^2.*YY+2.*phi.^2.*WW.^2.*YY+3.*WW.^3.*YY+2.*phi.*WW.^3.*YY+(-4) ...

.*phi.^2.*WW.^3.*YY+2.*phi.*WW.^4.*YY+2.*phi.^2.*WW.^4.*YY+(-1).*WW.* ...

XX.*YY+4.*phi.*WW.*XX.*YY+(-2).*phi.^2.*WW.*XX.*YY+(-3).*WW.^2.*XX.*YY+( ...

-2).*phi.*WW.^2.*XX.*YY+4.*phi.^2.*WW.^2.*XX.*YY+(-2).*phi.*WW.^3.*XX.* ...

YY+(-2).*phi.^2.*WW.^3.*XX.*YY+(-2).*phi.*WW.*ZZ+2.*phi.^2.*WW.*ZZ+3.* ...

WW.^2.*ZZ+(-2).*phi.*WW.^2.*ZZ+(-4).*phi.^2.*WW.^2.*ZZ+WW.^3.*ZZ+4.* ...

phi.*WW.^3.*ZZ+2.*phi.^2.*WW.^3.*ZZ+2.*phi.*XX.*ZZ+(-2).*phi.^2.*XX.*ZZ+ ...

(-3).*WW.*XX.*ZZ+2.*phi.*WW.*XX.*ZZ+4.*phi.^2.*WW.*XX.*ZZ+(-1).*WW.^2.* ...

XX.*ZZ+(-4).*phi.*WW.^2.*XX.*ZZ+(-2).*phi.^2.*WW.^2.*XX.*ZZ+(-1).* ...

phi.^2.*WW.*YY.*ZZ+4.*phi.*WW.^2.*YY.*ZZ+phi.^2.*WW.^2.*YY.*ZZ+(-3).* ...

WW.^3.*YY.*ZZ+(-2).*phi.*WW.^3.*YY.*ZZ+phi.^2.*WW.^3.*YY.*ZZ+(-1).* ...

WW.^4.*YY.*ZZ+(-2).*phi.*WW.^4.*YY.*ZZ+(-1).*phi.^2.*WW.^4.*YY.*ZZ+ ...

phi.^2.*XX.*YY.*ZZ+(-4).*phi.*WW.*XX.*YY.*ZZ+(-1).*phi.^2.*WW.*XX.*YY.* ...

ZZ+3.*WW.^2.*XX.*YY.*ZZ+2.*phi.*WW.^2.*XX.*YY.*ZZ+(-1).*phi.^2.*WW.^2.* ...

XX.*YY.*ZZ+WW.^3.*XX.*YY.*ZZ+2.*phi.*WW.^3.*XX.*YY.*ZZ+phi.^2.*WW.^3.* ...

XX.*YY.*ZZ)).^3+(27.*(WW+(-2).*phi.*WW+phi.^2.*WW+2.*phi.*WW.^2+(-2).* ...

phi.^2.*WW.^2+phi.^2.*WW.^3+(-1).*XX+2.*phi.*XX+(-1).*phi.^2.*XX+(-2).* ...

phi.*WW.*XX+2.*phi.^2.*WW.*XX+(-1).*phi.^2.*WW.^2.*XX+phi.*WW.*YY+(-1).* ...

phi.^2.*WW.*YY+(-1).*WW.^2.*YY+2.*phi.^2.*WW.^2.*YY+(-1).*phi.*WW.^3.* ...

YY+(-1).*phi.^2.*WW.^3.*YY+(-1).*phi.*XX.*YY+phi.^2.*XX.*YY+WW.*XX.*YY+( ...

-2).*phi.^2.*WW.*XX.*YY+phi.*WW.^2.*XX.*YY+phi.^2.*WW.^2.*XX.*YY+phi.* ...

WW.*ZZ+(-1).*phi.^2.*WW.*ZZ+(-1).*WW.^2.*ZZ+2.*phi.^2.*WW.^2.*ZZ+(-1).* ...

phi.*WW.^3.*ZZ+(-1).*phi.^2.*WW.^3.*ZZ+(-1).*phi.*XX.*ZZ+phi.^2.*XX.*ZZ+ ...

WW.*XX.*ZZ+(-2).*phi.^2.*WW.*XX.*ZZ+phi.*WW.^2.*XX.*ZZ+phi.^2.*WW.^2.* ...

XX.*ZZ+phi.^2.*WW.*YY.*ZZ+(-2).*phi.*WW.^2.*YY.*ZZ+(-2).*phi.^2.*WW.^2.* ...

YY.*ZZ+WW.^3.*YY.*ZZ+2.*phi.*WW.^3.*YY.*ZZ+phi.^2.*WW.^3.*YY.*ZZ+(-1).* ...

phi.^2.*XX.*YY.*ZZ+2.*phi.*WW.*XX.*YY.*ZZ+2.*phi.^2.*WW.*XX.*YY.*ZZ+(-1) ...

.*WW.^2.*XX.*YY.*ZZ+(-2).*phi.*WW.^2.*XX.*YY.*ZZ+(-1).*phi.^2.*WW.^2.* ...

XX.*YY.*ZZ).*((-3).*WW.^3+2.*phi.*WW.^3+(-1).*WW.^4+(-2).*phi.*WW.^4+3.* ...

WW.^2.*XX+(-2).*phi.*WW.^2.*XX+WW.^3.*XX+2.*phi.*WW.^3.*XX+(-1).*WW.^2.* ...

YY+2.*phi.*WW.^2.*YY+WW.^3.*YY+(-6).*phi.*WW.^3.*YY+4.*WW.^4.*YY+4.* ...

phi.*WW.^4.*YY+(-3).*WW.^3.*XX.*YY+2.*phi.*WW.^3.*XX.*YY+(-1).*WW.^4.* ...

XX.*YY+(-2).*phi.*WW.^4.*XX.*YY+WW.^2.*ZZ+(-2).*phi.*WW.^2.*ZZ+3.* ...

WW.^3.*ZZ+2.*phi.*WW.^3.*ZZ+(-4).*WW.^2.*XX.*ZZ+4.*phi.*WW.^2.*XX.*ZZ+( ...

-1).*WW.^3.*XX.*ZZ+(-6).*phi.*WW.^3.*XX.*ZZ+WW.^4.*XX.*ZZ+2.*phi.* ...

WW.^4.*XX.*ZZ+(-1).*WW.^3.*YY.*ZZ+2.*phi.*WW.^3.*YY.*ZZ+(-3).*WW.^4.* ...

YY.*ZZ+(-2).*phi.*WW.^4.*YY.*ZZ+WW.^2.*XX.*YY.*ZZ+(-2).*phi.*WW.^2.*XX.* ...

YY.*ZZ+3.*WW.^3.*XX.*YY.*ZZ+2.*phi.*WW.^3.*XX.*YY.*ZZ).^2+(-72).*(WW+( ...

-2).*phi.*WW+phi.^2.*WW+2.*phi.*WW.^2+(-2).*phi.^2.*WW.^2+phi.^2.*WW.^3+ ...

(-1).*XX+2.*phi.*XX+(-1).*phi.^2.*XX+(-2).*phi.*WW.*XX+2.*phi.^2.*WW.* ...

XX+(-1).*phi.^2.*WW.^2.*XX+phi.*WW.*YY+(-1).*phi.^2.*WW.*YY+(-1).* ...

WW.^2.*YY+2.*phi.^2.*WW.^2.*YY+(-1).*phi.*WW.^3.*YY+(-1).*phi.^2.* ...

WW.^3.*YY+(-1).*phi.*XX.*YY+phi.^2.*XX.*YY+WW.*XX.*YY+(-2).*phi.^2.*WW.* ...

XX.*YY+phi.*WW.^2.*XX.*YY+phi.^2.*WW.^2.*XX.*YY+phi.*WW.*ZZ+(-1).* ...

phi.^2.*WW.*ZZ+(-1).*WW.^2.*ZZ+2.*phi.^2.*WW.^2.*ZZ+(-1).*phi.*WW.^3.* ...

ZZ+(-1).*phi.^2.*WW.^3.*ZZ+(-1).*phi.*XX.*ZZ+phi.^2.*XX.*ZZ+WW.*XX.*ZZ+( ...

-2).*phi.^2.*WW.*XX.*ZZ+phi.*WW.^2.*XX.*ZZ+phi.^2.*WW.^2.*XX.*ZZ+ ...

phi.^2.*WW.*YY.*ZZ+(-2).*phi.*WW.^2.*YY.*ZZ+(-2).*phi.^2.*WW.^2.*YY.*ZZ+ ...

WW.^3.*YY.*ZZ+2.*phi.*WW.^3.*YY.*ZZ+phi.^2.*WW.^3.*YY.*ZZ+(-1).*phi.^2.* ...

XX.*YY.*ZZ+2.*phi.*WW.*XX.*YY.*ZZ+2.*phi.^2.*WW.*XX.*YY.*ZZ+(-1).* ...

WW.^2.*XX.*YY.*ZZ+(-2).*phi.*WW.^2.*XX.*YY.*ZZ+(-1).*phi.^2.*WW.^2.*XX.* ...

YY.*ZZ).*(WW.^4+(-1).*WW.^3.*XX+WW.^3.*YY+(-2).*WW.^4.*YY+WW.^4.*XX.*YY+ ...

(-1).*WW.^3.*ZZ+2.*WW.^3.*XX.*ZZ+(-1).*WW.^4.*XX.*ZZ+WW.^4.*YY.*ZZ+(-1) ...

.*WW.^3.*XX.*YY.*ZZ).*(3.*WW.^2+(-4).*phi.*WW.^2+phi.^2.*WW.^2+3.*WW.^3+ ...

2.*phi.*WW.^3+(-2).*phi.^2.*WW.^3+2.*phi.*WW.^4+phi.^2.*WW.^4+(-3).*WW.* ...

XX+4.*phi.*WW.*XX+(-1).*phi.^2.*WW.*XX+(-3).*WW.^2.*XX+(-2).*phi.* ...

WW.^2.*XX+2.*phi.^2.*WW.^2.*XX+(-2).*phi.*WW.^3.*XX+(-1).*phi.^2.* ...

WW.^3.*XX+(-1).*phi.*WW.*YY+phi.^2.*WW.*YY+WW.^2.*YY+2.*phi.*WW.^2.*YY+( ...

-4).*phi.^2.*WW.^2.*YY+(-5).*WW.^3.*YY+5.*phi.*WW.^3.*YY+5.*phi.^2.* ...

WW.^3.*YY+(-2).*WW.^4.*YY+(-6).*phi.*WW.^4.*YY+(-2).*phi.^2.*WW.^4.*YY+ ...

3.*WW.^2.*XX.*YY+(-5).*phi.*WW.^2.*XX.*YY+phi.^2.*WW.^2.*XX.*YY+3.* ...

WW.^3.*XX.*YY+4.*phi.*WW.^3.*XX.*YY+(-2).*phi.^2.*WW.^3.*XX.*YY+phi.* ...

WW.^4.*XX.*YY+phi.^2.*WW.^4.*XX.*YY+phi.*WW.*ZZ+(-1).*phi.^2.*WW.*ZZ+( ...

-3).*WW.^2.*ZZ+4.*phi.*WW.^2.*ZZ+2.*phi.^2.*WW.^2.*ZZ+(-3).*WW.^3.*ZZ+( ...

-5).*phi.*WW.^3.*ZZ+(-1).*phi.^2.*WW.^3.*ZZ+2.*WW.*XX.*ZZ+(-6).*phi.* ...

WW.*XX.*ZZ+2.*phi.^2.*WW.*XX.*ZZ+5.*WW.^2.*XX.*ZZ+5.*phi.*WW.^2.*XX.*ZZ+ ...

(-5).*phi.^2.*WW.^2.*XX.*ZZ+(-1).*WW.^3.*XX.*ZZ+2.*phi.*WW.^3.*XX.*ZZ+ ...

4.*phi.^2.*WW.^3.*XX.*ZZ+(-1).*phi.*WW.^4.*XX.*ZZ+(-1).*phi.^2.*WW.^4.* ...

XX.*ZZ+(-2).*phi.*WW.^2.*YY.*ZZ+phi.^2.*WW.^2.*YY.*ZZ+3.*WW.^3.*YY.*ZZ+( ...

-2).*phi.*WW.^3.*YY.*ZZ+(-2).*phi.^2.*WW.^3.*YY.*ZZ+3.*WW.^4.*YY.*ZZ+4.* ...

phi.*WW.^4.*YY.*ZZ+phi.^2.*WW.^4.*YY.*ZZ+2.*phi.*WW.*XX.*YY.*ZZ+(-1).* ...

phi.^2.*WW.*XX.*YY.*ZZ+(-3).*WW.^2.*XX.*YY.*ZZ+2.*phi.*WW.^2.*XX.*YY.* ...

ZZ+2.*phi.^2.*WW.^2.*XX.*YY.*ZZ+(-3).*WW.^3.*XX.*YY.*ZZ+(-4).*phi.* ...

WW.^3.*XX.*YY.*ZZ+(-1).*phi.^2.*WW.^3.*XX.*YY.*ZZ)+2.*(3.*WW.^2+(-4).* ...

phi.*WW.^2+phi.^2.*WW.^2+3.*WW.^3+2.*phi.*WW.^3+(-2).*phi.^2.*WW.^3+2.* ...

phi.*WW.^4+phi.^2.*WW.^4+(-3).*WW.*XX+4.*phi.*WW.*XX+(-1).*phi.^2.*WW.* ...

XX+(-3).*WW.^2.*XX+(-2).*phi.*WW.^2.*XX+2.*phi.^2.*WW.^2.*XX+(-2).*phi.* ...

WW.^3.*XX+(-1).*phi.^2.*WW.^3.*XX+(-1).*phi.*WW.*YY+phi.^2.*WW.*YY+ ...

WW.^2.*YY+2.*phi.*WW.^2.*YY+(-4).*phi.^2.*WW.^2.*YY+(-5).*WW.^3.*YY+5.* ...

phi.*WW.^3.*YY+5.*phi.^2.*WW.^3.*YY+(-2).*WW.^4.*YY+(-6).*phi.*WW.^4.* ...

YY+(-2).*phi.^2.*WW.^4.*YY+3.*WW.^2.*XX.*YY+(-5).*phi.*WW.^2.*XX.*YY+ ...

phi.^2.*WW.^2.*XX.*YY+3.*WW.^3.*XX.*YY+4.*phi.*WW.^3.*XX.*YY+(-2).* ...

phi.^2.*WW.^3.*XX.*YY+phi.*WW.^4.*XX.*YY+phi.^2.*WW.^4.*XX.*YY+phi.*WW.* ...

ZZ+(-1).*phi.^2.*WW.*ZZ+(-3).*WW.^2.*ZZ+4.*phi.*WW.^2.*ZZ+2.*phi.^2.* ...

WW.^2.*ZZ+(-3).*WW.^3.*ZZ+(-5).*phi.*WW.^3.*ZZ+(-1).*phi.^2.*WW.^3.*ZZ+ ...

2.*WW.*XX.*ZZ+(-6).*phi.*WW.*XX.*ZZ+2.*phi.^2.*WW.*XX.*ZZ+5.*WW.^2.*XX.* ...

ZZ+5.*phi.*WW.^2.*XX.*ZZ+(-5).*phi.^2.*WW.^2.*XX.*ZZ+(-1).*WW.^3.*XX.* ...

ZZ+2.*phi.*WW.^3.*XX.*ZZ+4.*phi.^2.*WW.^3.*XX.*ZZ+(-1).*phi.*WW.^4.*XX.* ...

ZZ+(-1).*phi.^2.*WW.^4.*XX.*ZZ+(-2).*phi.*WW.^2.*YY.*ZZ+phi.^2.*WW.^2.* ...

YY.*ZZ+3.*WW.^3.*YY.*ZZ+(-2).*phi.*WW.^3.*YY.*ZZ+(-2).*phi.^2.*WW.^3.* ...

YY.*ZZ+3.*WW.^4.*YY.*ZZ+4.*phi.*WW.^4.*YY.*ZZ+phi.^2.*WW.^4.*YY.*ZZ+2.* ...

phi.*WW.*XX.*YY.*ZZ+(-1).*phi.^2.*WW.*XX.*YY.*ZZ+(-3).*WW.^2.*XX.*YY.* ...

ZZ+2.*phi.*WW.^2.*XX.*YY.*ZZ+2.*phi.^2.*WW.^2.*XX.*YY.*ZZ+(-3).*WW.^3.* ...

XX.*YY.*ZZ+(-4).*phi.*WW.^3.*XX.*YY.*ZZ+(-1).*phi.^2.*WW.^3.*XX.*YY.*ZZ) ...

.^3+(-9).*((-3).*WW.^3+2.*phi.*WW.^3+(-1).*WW.^4+(-2).*phi.*WW.^4+3.* ...

WW.^2.*XX+(-2).*phi.*WW.^2.*XX+WW.^3.*XX+2.*phi.*WW.^3.*XX+(-1).*WW.^2.* ...

YY+2.*phi.*WW.^2.*YY+WW.^3.*YY+(-6).*phi.*WW.^3.*YY+4.*WW.^4.*YY+4.* ...

phi.*WW.^4.*YY+(-3).*WW.^3.*XX.*YY+2.*phi.*WW.^3.*XX.*YY+(-1).*WW.^4.* ...

XX.*YY+(-2).*phi.*WW.^4.*XX.*YY+WW.^2.*ZZ+(-2).*phi.*WW.^2.*ZZ+3.* ...

WW.^3.*ZZ+2.*phi.*WW.^3.*ZZ+(-4).*WW.^2.*XX.*ZZ+4.*phi.*WW.^2.*XX.*ZZ+( ...

-1).*WW.^3.*XX.*ZZ+(-6).*phi.*WW.^3.*XX.*ZZ+WW.^4.*XX.*ZZ+2.*phi.* ...

WW.^4.*XX.*ZZ+(-1).*WW.^3.*YY.*ZZ+2.*phi.*WW.^3.*YY.*ZZ+(-3).*WW.^4.* ...

YY.*ZZ+(-2).*phi.*WW.^4.*YY.*ZZ+WW.^2.*XX.*YY.*ZZ+(-2).*phi.*WW.^2.*XX.* ...

YY.*ZZ+3.*WW.^3.*XX.*YY.*ZZ+2.*phi.*WW.^3.*XX.*YY.*ZZ).*(3.*WW.^2+(-4).* ...

phi.*WW.^2+phi.^2.*WW.^2+3.*WW.^3+2.*phi.*WW.^3+(-2).*phi.^2.*WW.^3+2.* ...

phi.*WW.^4+phi.^2.*WW.^4+(-3).*WW.*XX+4.*phi.*WW.*XX+(-1).*phi.^2.*WW.* ...

XX+(-3).*WW.^2.*XX+(-2).*phi.*WW.^2.*XX+2.*phi.^2.*WW.^2.*XX+(-2).*phi.* ...

WW.^3.*XX+(-1).*phi.^2.*WW.^3.*XX+(-1).*phi.*WW.*YY+phi.^2.*WW.*YY+ ...

WW.^2.*YY+2.*phi.*WW.^2.*YY+(-4).*phi.^2.*WW.^2.*YY+(-5).*WW.^3.*YY+5.* ...

phi.*WW.^3.*YY+5.*phi.^2.*WW.^3.*YY+(-2).*WW.^4.*YY+(-6).*phi.*WW.^4.* ...

YY+(-2).*phi.^2.*WW.^4.*YY+3.*WW.^2.*XX.*YY+(-5).*phi.*WW.^2.*XX.*YY+ ...

phi.^2.*WW.^2.*XX.*YY+3.*WW.^3.*XX.*YY+4.*phi.*WW.^3.*XX.*YY+(-2).* ...

phi.^2.*WW.^3.*XX.*YY+phi.*WW.^4.*XX.*YY+phi.^2.*WW.^4.*XX.*YY+phi.*WW.* ...

ZZ+(-1).*phi.^2.*WW.*ZZ+(-3).*WW.^2.*ZZ+4.*phi.*WW.^2.*ZZ+2.*phi.^2.* ...

WW.^2.*ZZ+(-3).*WW.^3.*ZZ+(-5).*phi.*WW.^3.*ZZ+(-1).*phi.^2.*WW.^3.*ZZ+ ...

2.*WW.*XX.*ZZ+(-6).*phi.*WW.*XX.*ZZ+2.*phi.^2.*WW.*XX.*ZZ+5.*WW.^2.*XX.* ...

ZZ+5.*phi.*WW.^2.*XX.*ZZ+(-5).*phi.^2.*WW.^2.*XX.*ZZ+(-1).*WW.^3.*XX.* ...

ZZ+2.*phi.*WW.^3.*XX.*ZZ+4.*phi.^2.*WW.^3.*XX.*ZZ+(-1).*phi.*WW.^4.*XX.* ...

ZZ+(-1).*phi.^2.*WW.^4.*XX.*ZZ+(-2).*phi.*WW.^2.*YY.*ZZ+phi.^2.*WW.^2.* ...

YY.*ZZ+3.*WW.^3.*YY.*ZZ+(-2).*phi.*WW.^3.*YY.*ZZ+(-2).*phi.^2.*WW.^3.* ...

YY.*ZZ+3.*WW.^4.*YY.*ZZ+4.*phi.*WW.^4.*YY.*ZZ+phi.^2.*WW.^4.*YY.*ZZ+2.* ...

phi.*WW.*XX.*YY.*ZZ+(-1).*phi.^2.*WW.*XX.*YY.*ZZ+(-3).*WW.^2.*XX.*YY.* ...

ZZ+2.*phi.*WW.^2.*XX.*YY.*ZZ+2.*phi.^2.*WW.^2.*XX.*YY.*ZZ+(-3).*WW.^3.* ...

XX.*YY.*ZZ+(-4).*phi.*WW.^3.*XX.*YY.*ZZ+(-1).*phi.^2.*WW.^3.*XX.*YY.*ZZ) ...

.*((-1).*WW+2.*phi.*WW+(-1).*phi.^2.*WW+(-3).*WW.^2+2.*phi.*WW.^2+ ...

phi.^2.*WW.^2+(-4).*phi.*WW.^3+phi.^2.*WW.^3+(-1).*phi.^2.*WW.^4+XX+(-2) ...

.*phi.*XX+phi.^2.*XX+3.*WW.*XX+(-2).*phi.*WW.*XX+(-1).*phi.^2.*WW.*XX+ ...

4.*phi.*WW.^2.*XX+(-1).*phi.^2.*WW.^2.*XX+phi.^2.*WW.^3.*XX+WW.^2.*YY+( ...

-4).*phi.*WW.^2.*YY+2.*phi.^2.*WW.^2.*YY+3.*WW.^3.*YY+2.*phi.*WW.^3.*YY+ ...

(-4).*phi.^2.*WW.^3.*YY+2.*phi.*WW.^4.*YY+2.*phi.^2.*WW.^4.*YY+(-1).* ...

WW.*XX.*YY+4.*phi.*WW.*XX.*YY+(-2).*phi.^2.*WW.*XX.*YY+(-3).*WW.^2.*XX.* ...

YY+(-2).*phi.*WW.^2.*XX.*YY+4.*phi.^2.*WW.^2.*XX.*YY+(-2).*phi.*WW.^3.* ...

XX.*YY+(-2).*phi.^2.*WW.^3.*XX.*YY+(-2).*phi.*WW.*ZZ+2.*phi.^2.*WW.*ZZ+ ...

3.*WW.^2.*ZZ+(-2).*phi.*WW.^2.*ZZ+(-4).*phi.^2.*WW.^2.*ZZ+WW.^3.*ZZ+4.* ...

phi.*WW.^3.*ZZ+2.*phi.^2.*WW.^3.*ZZ+2.*phi.*XX.*ZZ+(-2).*phi.^2.*XX.*ZZ+ ...

(-3).*WW.*XX.*ZZ+2.*phi.*WW.*XX.*ZZ+4.*phi.^2.*WW.*XX.*ZZ+(-1).*WW.^2.* ...

XX.*ZZ+(-4).*phi.*WW.^2.*XX.*ZZ+(-2).*phi.^2.*WW.^2.*XX.*ZZ+(-1).* ...

phi.^2.*WW.*YY.*ZZ+4.*phi.*WW.^2.*YY.*ZZ+phi.^2.*WW.^2.*YY.*ZZ+(-3).* ...

WW.^3.*YY.*ZZ+(-2).*phi.*WW.^3.*YY.*ZZ+phi.^2.*WW.^3.*YY.*ZZ+(-1).* ...

WW.^4.*YY.*ZZ+(-2).*phi.*WW.^4.*YY.*ZZ+(-1).*phi.^2.*WW.^4.*YY.*ZZ+ ...

phi.^2.*XX.*YY.*ZZ+(-4).*phi.*WW.*XX.*YY.*ZZ+(-1).*phi.^2.*WW.*XX.*YY.* ...

ZZ+3.*WW.^2.*XX.*YY.*ZZ+2.*phi.*WW.^2.*XX.*YY.*ZZ+(-1).*phi.^2.*WW.^2.* ...

XX.*YY.*ZZ+WW.^3.*XX.*YY.*ZZ+2.*phi.*WW.^3.*XX.*YY.*ZZ+phi.^2.*WW.^3.* ...

XX.*YY.*ZZ)+27.*(WW.^4+(-1).*WW.^3.*XX+WW.^3.*YY+(-2).*WW.^4.*YY+WW.^4.* ...

XX.*YY+(-1).*WW.^3.*ZZ+2.*WW.^3.*XX.*ZZ+(-1).*WW.^4.*XX.*ZZ+WW.^4.*YY.* ...

ZZ+(-1).*WW.^3.*XX.*YY.*ZZ).*((-1).*WW+2.*phi.*WW+(-1).*phi.^2.*WW+(-3) ...

.*WW.^2+2.*phi.*WW.^2+phi.^2.*WW.^2+(-4).*phi.*WW.^3+phi.^2.*WW.^3+(-1) ...

.*phi.^2.*WW.^4+XX+(-2).*phi.*XX+phi.^2.*XX+3.*WW.*XX+(-2).*phi.*WW.*XX+ ...

(-1).*phi.^2.*WW.*XX+4.*phi.*WW.^2.*XX+(-1).*phi.^2.*WW.^2.*XX+phi.^2.* ...

WW.^3.*XX+WW.^2.*YY+(-4).*phi.*WW.^2.*YY+2.*phi.^2.*WW.^2.*YY+3.*WW.^3.* ...

YY+2.*phi.*WW.^3.*YY+(-4).*phi.^2.*WW.^3.*YY+2.*phi.*WW.^4.*YY+2.* ...

phi.^2.*WW.^4.*YY+(-1).*WW.*XX.*YY+4.*phi.*WW.*XX.*YY+(-2).*phi.^2.*WW.* ...

XX.*YY+(-3).*WW.^2.*XX.*YY+(-2).*phi.*WW.^2.*XX.*YY+4.*phi.^2.*WW.^2.* ...

XX.*YY+(-2).*phi.*WW.^3.*XX.*YY+(-2).*phi.^2.*WW.^3.*XX.*YY+(-2).*phi.* ...

WW.*ZZ+2.*phi.^2.*WW.*ZZ+3.*WW.^2.*ZZ+(-2).*phi.*WW.^2.*ZZ+(-4).* ...

phi.^2.*WW.^2.*ZZ+WW.^3.*ZZ+4.*phi.*WW.^3.*ZZ+2.*phi.^2.*WW.^3.*ZZ+2.* ...

phi.*XX.*ZZ+(-2).*phi.^2.*XX.*ZZ+(-3).*WW.*XX.*ZZ+2.*phi.*WW.*XX.*ZZ+4.* ...

phi.^2.*WW.*XX.*ZZ+(-1).*WW.^2.*XX.*ZZ+(-4).*phi.*WW.^2.*XX.*ZZ+(-2).* ...

phi.^2.*WW.^2.*XX.*ZZ+(-1).*phi.^2.*WW.*YY.*ZZ+4.*phi.*WW.^2.*YY.*ZZ+ ...

phi.^2.*WW.^2.*YY.*ZZ+(-3).*WW.^3.*YY.*ZZ+(-2).*phi.*WW.^3.*YY.*ZZ+ ...

phi.^2.*WW.^3.*YY.*ZZ+(-1).*WW.^4.*YY.*ZZ+(-2).*phi.*WW.^4.*YY.*ZZ+(-1) ...

.*phi.^2.*WW.^4.*YY.*ZZ+phi.^2.*XX.*YY.*ZZ+(-4).*phi.*WW.*XX.*YY.*ZZ+( ...

-1).*phi.^2.*WW.*XX.*YY.*ZZ+3.*WW.^2.*XX.*YY.*ZZ+2.*phi.*WW.^2.*XX.*YY.* ...

ZZ+(-1).*phi.^2.*WW.^2.*XX.*YY.*ZZ+WW.^3.*XX.*YY.*ZZ+2.*phi.*WW.^3.*XX.* ...

YY.*ZZ+phi.^2.*WW.^3.*XX.*YY.*ZZ).^2).^2).^(1/2)).^(1/3)+(-1/4).*((-1).* ...

((-1)+phi+(-1).*phi.*WW+(-1).*phi.*YY+WW.*YY+phi.*WW.*YY).^(-3).*((-1)+ ...

phi+(-1).*phi.*WW+(-1).*phi.*ZZ+WW.*ZZ+phi.*WW.*ZZ).^(-3).*((-1)+2.*phi+ ...

(-1).*phi.^2+(-3).*WW+2.*phi.*WW+phi.^2.*WW+(-4).*phi.*WW.^2+phi.^2.* ...

WW.^2+(-1).*phi.^2.*WW.^3+WW.*YY+(-4).*phi.*WW.*YY+2.*phi.^2.*WW.*YY+3.* ...

WW.^2.*YY+2.*phi.*WW.^2.*YY+(-4).*phi.^2.*WW.^2.*YY+2.*phi.*WW.^3.*YY+ ...

2.*phi.^2.*WW.^3.*YY+(-2).*phi.*ZZ+2.*phi.^2.*ZZ+3.*WW.*ZZ+(-2).*phi.* ...

WW.*ZZ+(-4).*phi.^2.*WW.*ZZ+WW.^2.*ZZ+4.*phi.*WW.^2.*ZZ+2.*phi.^2.* ...

WW.^2.*ZZ+(-1).*phi.^2.*YY.*ZZ+4.*phi.*WW.*YY.*ZZ+phi.^2.*WW.*YY.*ZZ+( ...

-3).*WW.^2.*YY.*ZZ+(-2).*phi.*WW.^2.*YY.*ZZ+phi.^2.*WW.^2.*YY.*ZZ+(-1).* ...

WW.^3.*YY.*ZZ+(-2).*phi.*WW.^3.*YY.*ZZ+(-1).*phi.^2.*WW.^3.*YY.*ZZ).^3+( ...

-8).*(WW+(-1).*XX).^(-1).*((-1)+phi+(-1).*phi.*WW+(-1).*phi.*YY+WW.*YY+ ...

phi.*WW.*YY).^(-1).*((-1)+phi+(-1).*phi.*WW+(-1).*phi.*ZZ+WW.*ZZ+phi.* ...

WW.*ZZ).^(-1).*((-3).*WW.^3+2.*phi.*WW.^3+(-1).*WW.^4+(-2).*phi.*WW.^4+ ...

3.*WW.^2.*XX+(-2).*phi.*WW.^2.*XX+WW.^3.*XX+2.*phi.*WW.^3.*XX+(-1).* ...

WW.^2.*YY+2.*phi.*WW.^2.*YY+WW.^3.*YY+(-6).*phi.*WW.^3.*YY+4.*WW.^4.*YY+ ...

4.*phi.*WW.^4.*YY+(-3).*WW.^3.*XX.*YY+2.*phi.*WW.^3.*XX.*YY+(-1).* ...

WW.^4.*XX.*YY+(-2).*phi.*WW.^4.*XX.*YY+WW.^2.*ZZ+(-2).*phi.*WW.^2.*ZZ+ ...

3.*WW.^3.*ZZ+2.*phi.*WW.^3.*ZZ+(-4).*WW.^2.*XX.*ZZ+4.*phi.*WW.^2.*XX.* ...

ZZ+(-1).*WW.^3.*XX.*ZZ+(-6).*phi.*WW.^3.*XX.*ZZ+WW.^4.*XX.*ZZ+2.*phi.* ...

WW.^4.*XX.*ZZ+(-1).*WW.^3.*YY.*ZZ+2.*phi.*WW.^3.*YY.*ZZ+(-3).*WW.^4.* ...

YY.*ZZ+(-2).*phi.*WW.^4.*YY.*ZZ+WW.^2.*XX.*YY.*ZZ+(-2).*phi.*WW.^2.*XX.* ...

YY.*ZZ+3.*WW.^3.*XX.*YY.*ZZ+2.*phi.*WW.^3.*XX.*YY.*ZZ)+4.*(WW+(-1).*XX) ...

.^(-1).*((-1)+phi+(-1).*phi.*WW+(-1).*phi.*YY+WW.*YY+phi.*WW.*YY).^(-2) ...

.*((-1)+phi+(-1).*phi.*WW+(-1).*phi.*ZZ+WW.*ZZ+phi.*WW.*ZZ).^(-2).*((-1) ...

+2.*phi+(-1).*phi.^2+(-3).*WW+2.*phi.*WW+phi.^2.*WW+(-4).*phi.*WW.^2+ ...

phi.^2.*WW.^2+(-1).*phi.^2.*WW.^3+WW.*YY+(-4).*phi.*WW.*YY+2.*phi.^2.* ...

WW.*YY+3.*WW.^2.*YY+2.*phi.*WW.^2.*YY+(-4).*phi.^2.*WW.^2.*YY+2.*phi.* ...

WW.^3.*YY+2.*phi.^2.*WW.^3.*YY+(-2).*phi.*ZZ+2.*phi.^2.*ZZ+3.*WW.*ZZ+( ...

-2).*phi.*WW.*ZZ+(-4).*phi.^2.*WW.*ZZ+WW.^2.*ZZ+4.*phi.*WW.^2.*ZZ+2.* ...

phi.^2.*WW.^2.*ZZ+(-1).*phi.^2.*YY.*ZZ+4.*phi.*WW.*YY.*ZZ+phi.^2.*WW.* ...

YY.*ZZ+(-3).*WW.^2.*YY.*ZZ+(-2).*phi.*WW.^2.*YY.*ZZ+phi.^2.*WW.^2.*YY.* ...

ZZ+(-1).*WW.^3.*YY.*ZZ+(-2).*phi.*WW.^3.*YY.*ZZ+(-1).*phi.^2.*WW.^3.* ...

YY.*ZZ).*(3.*WW.^2+(-4).*phi.*WW.^2+phi.^2.*WW.^2+3.*WW.^3+2.*phi.* ...

WW.^3+(-2).*phi.^2.*WW.^3+2.*phi.*WW.^4+phi.^2.*WW.^4+(-3).*WW.*XX+4.* ...

phi.*WW.*XX+(-1).*phi.^2.*WW.*XX+(-3).*WW.^2.*XX+(-2).*phi.*WW.^2.*XX+ ...

2.*phi.^2.*WW.^2.*XX+(-2).*phi.*WW.^3.*XX+(-1).*phi.^2.*WW.^3.*XX+(-1).* ...

phi.*WW.*YY+phi.^2.*WW.*YY+WW.^2.*YY+2.*phi.*WW.^2.*YY+(-4).*phi.^2.* ...

WW.^2.*YY+(-5).*WW.^3.*YY+5.*phi.*WW.^3.*YY+5.*phi.^2.*WW.^3.*YY+(-2).* ...

WW.^4.*YY+(-6).*phi.*WW.^4.*YY+(-2).*phi.^2.*WW.^4.*YY+3.*WW.^2.*XX.*YY+ ...

(-5).*phi.*WW.^2.*XX.*YY+phi.^2.*WW.^2.*XX.*YY+3.*WW.^3.*XX.*YY+4.*phi.* ...

WW.^3.*XX.*YY+(-2).*phi.^2.*WW.^3.*XX.*YY+phi.*WW.^4.*XX.*YY+phi.^2.* ...

WW.^4.*XX.*YY+phi.*WW.*ZZ+(-1).*phi.^2.*WW.*ZZ+(-3).*WW.^2.*ZZ+4.*phi.* ...

WW.^2.*ZZ+2.*phi.^2.*WW.^2.*ZZ+(-3).*WW.^3.*ZZ+(-5).*phi.*WW.^3.*ZZ+(-1) ...

.*phi.^2.*WW.^3.*ZZ+2.*WW.*XX.*ZZ+(-6).*phi.*WW.*XX.*ZZ+2.*phi.^2.*WW.* ...

XX.*ZZ+5.*WW.^2.*XX.*ZZ+5.*phi.*WW.^2.*XX.*ZZ+(-5).*phi.^2.*WW.^2.*XX.* ...

ZZ+(-1).*WW.^3.*XX.*ZZ+2.*phi.*WW.^3.*XX.*ZZ+4.*phi.^2.*WW.^3.*XX.*ZZ+( ...

-1).*phi.*WW.^4.*XX.*ZZ+(-1).*phi.^2.*WW.^4.*XX.*ZZ+(-2).*phi.*WW.^2.* ...

YY.*ZZ+phi.^2.*WW.^2.*YY.*ZZ+3.*WW.^3.*YY.*ZZ+(-2).*phi.*WW.^3.*YY.*ZZ+( ...

-2).*phi.^2.*WW.^3.*YY.*ZZ+3.*WW.^4.*YY.*ZZ+4.*phi.*WW.^4.*YY.*ZZ+ ...

phi.^2.*WW.^4.*YY.*ZZ+2.*phi.*WW.*XX.*YY.*ZZ+(-1).*phi.^2.*WW.*XX.*YY.* ...

ZZ+(-3).*WW.^2.*XX.*YY.*ZZ+2.*phi.*WW.^2.*XX.*YY.*ZZ+2.*phi.^2.*WW.^2.* ...

XX.*YY.*ZZ+(-3).*WW.^3.*XX.*YY.*ZZ+(-4).*phi.*WW.^3.*XX.*YY.*ZZ+(-1).* ...

phi.^2.*WW.^3.*XX.*YY.*ZZ)).*((1/4).*((-1)+phi+(-1).*phi.*WW+(-1).*phi.* ...

YY+WW.*YY+phi.*WW.*YY).^(-2).*((-1)+phi+(-1).*phi.*WW+(-1).*phi.*ZZ+WW.* ...

ZZ+phi.*WW.*ZZ).^(-2).*((-1)+2.*phi+(-1).*phi.^2+(-3).*WW+2.*phi.*WW+ ...

phi.^2.*WW+(-4).*phi.*WW.^2+phi.^2.*WW.^2+(-1).*phi.^2.*WW.^3+WW.*YY+( ...

-4).*phi.*WW.*YY+2.*phi.^2.*WW.*YY+3.*WW.^2.*YY+2.*phi.*WW.^2.*YY+(-4).* ...

phi.^2.*WW.^2.*YY+2.*phi.*WW.^3.*YY+2.*phi.^2.*WW.^3.*YY+(-2).*phi.*ZZ+ ...

2.*phi.^2.*ZZ+3.*WW.*ZZ+(-2).*phi.*WW.*ZZ+(-4).*phi.^2.*WW.*ZZ+WW.^2.* ...

ZZ+4.*phi.*WW.^2.*ZZ+2.*phi.^2.*WW.^2.*ZZ+(-1).*phi.^2.*YY.*ZZ+4.*phi.* ...

WW.*YY.*ZZ+phi.^2.*WW.*YY.*ZZ+(-3).*WW.^2.*YY.*ZZ+(-2).*phi.*WW.^2.*YY.* ...

ZZ+phi.^2.*WW.^2.*YY.*ZZ+(-1).*WW.^3.*YY.*ZZ+(-2).*phi.*WW.^3.*YY.*ZZ+( ...

-1).*phi.^2.*WW.^3.*YY.*ZZ).^2+(-1).*(WW+(-1).*XX).^(-1).*((-1)+phi+(-1) ...

.*phi.*WW+(-1).*phi.*YY+WW.*YY+phi.*WW.*YY).^(-1).*((-1)+phi+(-1).*phi.* ...

WW+(-1).*phi.*ZZ+WW.*ZZ+phi.*WW.*ZZ).^(-1).*(3.*WW.^2+(-4).*phi.*WW.^2+ ...

phi.^2.*WW.^2+3.*WW.^3+2.*phi.*WW.^3+(-2).*phi.^2.*WW.^3+2.*phi.*WW.^4+ ...

phi.^2.*WW.^4+(-3).*WW.*XX+4.*phi.*WW.*XX+(-1).*phi.^2.*WW.*XX+(-3).* ...

WW.^2.*XX+(-2).*phi.*WW.^2.*XX+2.*phi.^2.*WW.^2.*XX+(-2).*phi.*WW.^3.* ...

XX+(-1).*phi.^2.*WW.^3.*XX+(-1).*phi.*WW.*YY+phi.^2.*WW.*YY+WW.^2.*YY+ ...

2.*phi.*WW.^2.*YY+(-4).*phi.^2.*WW.^2.*YY+(-5).*WW.^3.*YY+5.*phi.* ...

WW.^3.*YY+5.*phi.^2.*WW.^3.*YY+(-2).*WW.^4.*YY+(-6).*phi.*WW.^4.*YY+(-2) ...

.*phi.^2.*WW.^4.*YY+3.*WW.^2.*XX.*YY+(-5).*phi.*WW.^2.*XX.*YY+phi.^2.* ...

WW.^2.*XX.*YY+3.*WW.^3.*XX.*YY+4.*phi.*WW.^3.*XX.*YY+(-2).*phi.^2.* ...

WW.^3.*XX.*YY+phi.*WW.^4.*XX.*YY+phi.^2.*WW.^4.*XX.*YY+phi.*WW.*ZZ+(-1) ...

.*phi.^2.*WW.*ZZ+(-3).*WW.^2.*ZZ+4.*phi.*WW.^2.*ZZ+2.*phi.^2.*WW.^2.*ZZ+ ...

(-3).*WW.^3.*ZZ+(-5).*phi.*WW.^3.*ZZ+(-1).*phi.^2.*WW.^3.*ZZ+2.*WW.*XX.* ...

ZZ+(-6).*phi.*WW.*XX.*ZZ+2.*phi.^2.*WW.*XX.*ZZ+5.*WW.^2.*XX.*ZZ+5.*phi.* ...

WW.^2.*XX.*ZZ+(-5).*phi.^2.*WW.^2.*XX.*ZZ+(-1).*WW.^3.*XX.*ZZ+2.*phi.* ...

WW.^3.*XX.*ZZ+4.*phi.^2.*WW.^3.*XX.*ZZ+(-1).*phi.*WW.^4.*XX.*ZZ+(-1).* ...

phi.^2.*WW.^4.*XX.*ZZ+(-2).*phi.*WW.^2.*YY.*ZZ+phi.^2.*WW.^2.*YY.*ZZ+3.* ...

WW.^3.*YY.*ZZ+(-2).*phi.*WW.^3.*YY.*ZZ+(-2).*phi.^2.*WW.^3.*YY.*ZZ+3.* ...

WW.^4.*YY.*ZZ+4.*phi.*WW.^4.*YY.*ZZ+phi.^2.*WW.^4.*YY.*ZZ+2.*phi.*WW.* ...

XX.*YY.*ZZ+(-1).*phi.^2.*WW.*XX.*YY.*ZZ+(-3).*WW.^2.*XX.*YY.*ZZ+2.*phi.* ...

WW.^2.*XX.*YY.*ZZ+2.*phi.^2.*WW.^2.*XX.*YY.*ZZ+(-3).*WW.^3.*XX.*YY.*ZZ+( ...

-4).*phi.*WW.^3.*XX.*YY.*ZZ+(-1).*phi.^2.*WW.^3.*XX.*YY.*ZZ)+(1/3).*(WW+ ...

(-2).*phi.*WW+phi.^2.*WW+2.*phi.*WW.^2+(-2).*phi.^2.*WW.^2+phi.^2.* ...

WW.^3+(-1).*XX+2.*phi.*XX+(-1).*phi.^2.*XX+(-2).*phi.*WW.*XX+2.*phi.^2.* ...

WW.*XX+(-1).*phi.^2.*WW.^2.*XX+phi.*WW.*YY+(-1).*phi.^2.*WW.*YY+(-1).* ...

WW.^2.*YY+2.*phi.^2.*WW.^2.*YY+(-1).*phi.*WW.^3.*YY+(-1).*phi.^2.* ...

WW.^3.*YY+(-1).*phi.*XX.*YY+phi.^2.*XX.*YY+WW.*XX.*YY+(-2).*phi.^2.*WW.* ...

XX.*YY+phi.*WW.^2.*XX.*YY+phi.^2.*WW.^2.*XX.*YY+phi.*WW.*ZZ+(-1).* ...

phi.^2.*WW.*ZZ+(-1).*WW.^2.*ZZ+2.*phi.^2.*WW.^2.*ZZ+(-1).*phi.*WW.^3.* ...

ZZ+(-1).*phi.^2.*WW.^3.*ZZ+(-1).*phi.*XX.*ZZ+phi.^2.*XX.*ZZ+WW.*XX.*ZZ+( ...

-2).*phi.^2.*WW.*XX.*ZZ+phi.*WW.^2.*XX.*ZZ+phi.^2.*WW.^2.*XX.*ZZ+ ...

phi.^2.*WW.*YY.*ZZ+(-2).*phi.*WW.^2.*YY.*ZZ+(-2).*phi.^2.*WW.^2.*YY.*ZZ+ ...

WW.^3.*YY.*ZZ+2.*phi.*WW.^3.*YY.*ZZ+phi.^2.*WW.^3.*YY.*ZZ+(-1).*phi.^2.* ...

XX.*YY.*ZZ+2.*phi.*WW.*XX.*YY.*ZZ+2.*phi.^2.*WW.*XX.*YY.*ZZ+(-1).* ...

WW.^2.*XX.*YY.*ZZ+(-2).*phi.*WW.^2.*XX.*YY.*ZZ+(-1).*phi.^2.*WW.^2.*XX.* ...

YY.*ZZ).^(-1).*(3.*WW.^2+(-4).*phi.*WW.^2+phi.^2.*WW.^2+3.*WW.^3+2.* ...

phi.*WW.^3+(-2).*phi.^2.*WW.^3+2.*phi.*WW.^4+phi.^2.*WW.^4+(-3).*WW.*XX+ ...

4.*phi.*WW.*XX+(-1).*phi.^2.*WW.*XX+(-3).*WW.^2.*XX+(-2).*phi.*WW.^2.* ...

XX+2.*phi.^2.*WW.^2.*XX+(-2).*phi.*WW.^3.*XX+(-1).*phi.^2.*WW.^3.*XX+( ...

-1).*phi.*WW.*YY+phi.^2.*WW.*YY+WW.^2.*YY+2.*phi.*WW.^2.*YY+(-4).* ...

phi.^2.*WW.^2.*YY+(-5).*WW.^3.*YY+5.*phi.*WW.^3.*YY+5.*phi.^2.*WW.^3.* ...

YY+(-2).*WW.^4.*YY+(-6).*phi.*WW.^4.*YY+(-2).*phi.^2.*WW.^4.*YY+3.* ...

WW.^2.*XX.*YY+(-5).*phi.*WW.^2.*XX.*YY+phi.^2.*WW.^2.*XX.*YY+3.*WW.^3.* ...

XX.*YY+4.*phi.*WW.^3.*XX.*YY+(-2).*phi.^2.*WW.^3.*XX.*YY+phi.*WW.^4.* ...

XX.*YY+phi.^2.*WW.^4.*XX.*YY+phi.*WW.*ZZ+(-1).*phi.^2.*WW.*ZZ+(-3).* ...

WW.^2.*ZZ+4.*phi.*WW.^2.*ZZ+2.*phi.^2.*WW.^2.*ZZ+(-3).*WW.^3.*ZZ+(-5).* ...

phi.*WW.^3.*ZZ+(-1).*phi.^2.*WW.^3.*ZZ+2.*WW.*XX.*ZZ+(-6).*phi.*WW.*XX.* ...

ZZ+2.*phi.^2.*WW.*XX.*ZZ+5.*WW.^2.*XX.*ZZ+5.*phi.*WW.^2.*XX.*ZZ+(-5).* ...

phi.^2.*WW.^2.*XX.*ZZ+(-1).*WW.^3.*XX.*ZZ+2.*phi.*WW.^3.*XX.*ZZ+4.* ...

phi.^2.*WW.^3.*XX.*ZZ+(-1).*phi.*WW.^4.*XX.*ZZ+(-1).*phi.^2.*WW.^4.*XX.* ...

ZZ+(-2).*phi.*WW.^2.*YY.*ZZ+phi.^2.*WW.^2.*YY.*ZZ+3.*WW.^3.*YY.*ZZ+(-2) ...

.*phi.*WW.^3.*YY.*ZZ+(-2).*phi.^2.*WW.^3.*YY.*ZZ+3.*WW.^4.*YY.*ZZ+4.* ...

phi.*WW.^4.*YY.*ZZ+phi.^2.*WW.^4.*YY.*ZZ+2.*phi.*WW.*XX.*YY.*ZZ+(-1).* ...

phi.^2.*WW.*XX.*YY.*ZZ+(-3).*WW.^2.*XX.*YY.*ZZ+2.*phi.*WW.^2.*XX.*YY.* ...

ZZ+2.*phi.^2.*WW.^2.*XX.*YY.*ZZ+(-3).*WW.^3.*XX.*YY.*ZZ+(-4).*phi.* ...

WW.^3.*XX.*YY.*ZZ+(-1).*phi.^2.*WW.^3.*XX.*YY.*ZZ)+(1/3).*2.^(1/3).*(WW+ ...

(-1).*XX).^(-1).*((-1)+phi+(-1).*phi.*WW+(-1).*phi.*YY+WW.*YY+phi.*WW.* ...

YY).^(-1).*((-1)+phi+(-1).*phi.*WW+(-1).*phi.*ZZ+WW.*ZZ+phi.*WW.*ZZ).^( ...

-1).*(12.*(WW+(-2).*phi.*WW+phi.^2.*WW+2.*phi.*WW.^2+(-2).*phi.^2.* ...

WW.^2+phi.^2.*WW.^3+(-1).*XX+2.*phi.*XX+(-1).*phi.^2.*XX+(-2).*phi.*WW.* ...

XX+2.*phi.^2.*WW.*XX+(-1).*phi.^2.*WW.^2.*XX+phi.*WW.*YY+(-1).*phi.^2.* ...

WW.*YY+(-1).*WW.^2.*YY+2.*phi.^2.*WW.^2.*YY+(-1).*phi.*WW.^3.*YY+(-1).* ...

phi.^2.*WW.^3.*YY+(-1).*phi.*XX.*YY+phi.^2.*XX.*YY+WW.*XX.*YY+(-2).* ...

phi.^2.*WW.*XX.*YY+phi.*WW.^2.*XX.*YY+phi.^2.*WW.^2.*XX.*YY+phi.*WW.*ZZ+ ...

(-1).*phi.^2.*WW.*ZZ+(-1).*WW.^2.*ZZ+2.*phi.^2.*WW.^2.*ZZ+(-1).*phi.* ...

WW.^3.*ZZ+(-1).*phi.^2.*WW.^3.*ZZ+(-1).*phi.*XX.*ZZ+phi.^2.*XX.*ZZ+WW.* ...

XX.*ZZ+(-2).*phi.^2.*WW.*XX.*ZZ+phi.*WW.^2.*XX.*ZZ+phi.^2.*WW.^2.*XX.* ...

ZZ+phi.^2.*WW.*YY.*ZZ+(-2).*phi.*WW.^2.*YY.*ZZ+(-2).*phi.^2.*WW.^2.*YY.* ...

ZZ+WW.^3.*YY.*ZZ+2.*phi.*WW.^3.*YY.*ZZ+phi.^2.*WW.^3.*YY.*ZZ+(-1).* ...

phi.^2.*XX.*YY.*ZZ+2.*phi.*WW.*XX.*YY.*ZZ+2.*phi.^2.*WW.*XX.*YY.*ZZ+(-1) ...

.*WW.^2.*XX.*YY.*ZZ+(-2).*phi.*WW.^2.*XX.*YY.*ZZ+(-1).*phi.^2.*WW.^2.* ...

XX.*YY.*ZZ).*(WW.^4+(-1).*WW.^3.*XX+WW.^3.*YY+(-2).*WW.^4.*YY+WW.^4.* ...

XX.*YY+(-1).*WW.^3.*ZZ+2.*WW.^3.*XX.*ZZ+(-1).*WW.^4.*XX.*ZZ+WW.^4.*YY.* ...

ZZ+(-1).*WW.^3.*XX.*YY.*ZZ)+(3.*WW.^2+(-4).*phi.*WW.^2+phi.^2.*WW.^2+3.* ...

WW.^3+2.*phi.*WW.^3+(-2).*phi.^2.*WW.^3+2.*phi.*WW.^4+phi.^2.*WW.^4+(-3) ...

.*WW.*XX+4.*phi.*WW.*XX+(-1).*phi.^2.*WW.*XX+(-3).*WW.^2.*XX+(-2).*phi.* ...

WW.^2.*XX+2.*phi.^2.*WW.^2.*XX+(-2).*phi.*WW.^3.*XX+(-1).*phi.^2.* ...

WW.^3.*XX+(-1).*phi.*WW.*YY+phi.^2.*WW.*YY+WW.^2.*YY+2.*phi.*WW.^2.*YY+( ...

-4).*phi.^2.*WW.^2.*YY+(-5).*WW.^3.*YY+5.*phi.*WW.^3.*YY+5.*phi.^2.* ...

WW.^3.*YY+(-2).*WW.^4.*YY+(-6).*phi.*WW.^4.*YY+(-2).*phi.^2.*WW.^4.*YY+ ...

3.*WW.^2.*XX.*YY+(-5).*phi.*WW.^2.*XX.*YY+phi.^2.*WW.^2.*XX.*YY+3.* ...

WW.^3.*XX.*YY+4.*phi.*WW.^3.*XX.*YY+(-2).*phi.^2.*WW.^3.*XX.*YY+phi.* ...

WW.^4.*XX.*YY+phi.^2.*WW.^4.*XX.*YY+phi.*WW.*ZZ+(-1).*phi.^2.*WW.*ZZ+( ...

-3).*WW.^2.*ZZ+4.*phi.*WW.^2.*ZZ+2.*phi.^2.*WW.^2.*ZZ+(-3).*WW.^3.*ZZ+( ...

-5).*phi.*WW.^3.*ZZ+(-1).*phi.^2.*WW.^3.*ZZ+2.*WW.*XX.*ZZ+(-6).*phi.* ...

WW.*XX.*ZZ+2.*phi.^2.*WW.*XX.*ZZ+5.*WW.^2.*XX.*ZZ+5.*phi.*WW.^2.*XX.*ZZ+ ...

(-5).*phi.^2.*WW.^2.*XX.*ZZ+(-1).*WW.^3.*XX.*ZZ+2.*phi.*WW.^3.*XX.*ZZ+ ...

4.*phi.^2.*WW.^3.*XX.*ZZ+(-1).*phi.*WW.^4.*XX.*ZZ+(-1).*phi.^2.*WW.^4.* ...

XX.*ZZ+(-2).*phi.*WW.^2.*YY.*ZZ+phi.^2.*WW.^2.*YY.*ZZ+3.*WW.^3.*YY.*ZZ+( ...

-2).*phi.*WW.^3.*YY.*ZZ+(-2).*phi.^2.*WW.^3.*YY.*ZZ+3.*WW.^4.*YY.*ZZ+4.* ...

phi.*WW.^4.*YY.*ZZ+phi.^2.*WW.^4.*YY.*ZZ+2.*phi.*WW.*XX.*YY.*ZZ+(-1).* ...

phi.^2.*WW.*XX.*YY.*ZZ+(-3).*WW.^2.*XX.*YY.*ZZ+2.*phi.*WW.^2.*XX.*YY.* ...

ZZ+2.*phi.^2.*WW.^2.*XX.*YY.*ZZ+(-3).*WW.^3.*XX.*YY.*ZZ+(-4).*phi.* ...

WW.^3.*XX.*YY.*ZZ+(-1).*phi.^2.*WW.^3.*XX.*YY.*ZZ).^2+(-3).*((-3).* ...

WW.^3+2.*phi.*WW.^3+(-1).*WW.^4+(-2).*phi.*WW.^4+3.*WW.^2.*XX+(-2).* ...

phi.*WW.^2.*XX+WW.^3.*XX+2.*phi.*WW.^3.*XX+(-1).*WW.^2.*YY+2.*phi.* ...

WW.^2.*YY+WW.^3.*YY+(-6).*phi.*WW.^3.*YY+4.*WW.^4.*YY+4.*phi.*WW.^4.*YY+ ...

(-3).*WW.^3.*XX.*YY+2.*phi.*WW.^3.*XX.*YY+(-1).*WW.^4.*XX.*YY+(-2).* ...

phi.*WW.^4.*XX.*YY+WW.^2.*ZZ+(-2).*phi.*WW.^2.*ZZ+3.*WW.^3.*ZZ+2.*phi.* ...

WW.^3.*ZZ+(-4).*WW.^2.*XX.*ZZ+4.*phi.*WW.^2.*XX.*ZZ+(-1).*WW.^3.*XX.*ZZ+ ...

(-6).*phi.*WW.^3.*XX.*ZZ+WW.^4.*XX.*ZZ+2.*phi.*WW.^4.*XX.*ZZ+(-1).* ...

WW.^3.*YY.*ZZ+2.*phi.*WW.^3.*YY.*ZZ+(-3).*WW.^4.*YY.*ZZ+(-2).*phi.* ...

WW.^4.*YY.*ZZ+WW.^2.*XX.*YY.*ZZ+(-2).*phi.*WW.^2.*XX.*YY.*ZZ+3.*WW.^3.* ...

XX.*YY.*ZZ+2.*phi.*WW.^3.*XX.*YY.*ZZ).*((-1).*WW+2.*phi.*WW+(-1).* ...

phi.^2.*WW+(-3).*WW.^2+2.*phi.*WW.^2+phi.^2.*WW.^2+(-4).*phi.*WW.^3+ ...

phi.^2.*WW.^3+(-1).*phi.^2.*WW.^4+XX+(-2).*phi.*XX+phi.^2.*XX+3.*WW.*XX+ ...

(-2).*phi.*WW.*XX+(-1).*phi.^2.*WW.*XX+4.*phi.*WW.^2.*XX+(-1).*phi.^2.* ...

WW.^2.*XX+phi.^2.*WW.^3.*XX+WW.^2.*YY+(-4).*phi.*WW.^2.*YY+2.*phi.^2.* ...

WW.^2.*YY+3.*WW.^3.*YY+2.*phi.*WW.^3.*YY+(-4).*phi.^2.*WW.^3.*YY+2.* ...

phi.*WW.^4.*YY+2.*phi.^2.*WW.^4.*YY+(-1).*WW.*XX.*YY+4.*phi.*WW.*XX.*YY+ ...

(-2).*phi.^2.*WW.*XX.*YY+(-3).*WW.^2.*XX.*YY+(-2).*phi.*WW.^2.*XX.*YY+ ...

4.*phi.^2.*WW.^2.*XX.*YY+(-2).*phi.*WW.^3.*XX.*YY+(-2).*phi.^2.*WW.^3.* ...

XX.*YY+(-2).*phi.*WW.*ZZ+2.*phi.^2.*WW.*ZZ+3.*WW.^2.*ZZ+(-2).*phi.* ...

WW.^2.*ZZ+(-4).*phi.^2.*WW.^2.*ZZ+WW.^3.*ZZ+4.*phi.*WW.^3.*ZZ+2.* ...

phi.^2.*WW.^3.*ZZ+2.*phi.*XX.*ZZ+(-2).*phi.^2.*XX.*ZZ+(-3).*WW.*XX.*ZZ+ ...

2.*phi.*WW.*XX.*ZZ+4.*phi.^2.*WW.*XX.*ZZ+(-1).*WW.^2.*XX.*ZZ+(-4).*phi.* ...

WW.^2.*XX.*ZZ+(-2).*phi.^2.*WW.^2.*XX.*ZZ+(-1).*phi.^2.*WW.*YY.*ZZ+4.* ...

phi.*WW.^2.*YY.*ZZ+phi.^2.*WW.^2.*YY.*ZZ+(-3).*WW.^3.*YY.*ZZ+(-2).*phi.* ...

WW.^3.*YY.*ZZ+phi.^2.*WW.^3.*YY.*ZZ+(-1).*WW.^4.*YY.*ZZ+(-2).*phi.* ...

WW.^4.*YY.*ZZ+(-1).*phi.^2.*WW.^4.*YY.*ZZ+phi.^2.*XX.*YY.*ZZ+(-4).*phi.* ...

WW.*XX.*YY.*ZZ+(-1).*phi.^2.*WW.*XX.*YY.*ZZ+3.*WW.^2.*XX.*YY.*ZZ+2.* ...

phi.*WW.^2.*XX.*YY.*ZZ+(-1).*phi.^2.*WW.^2.*XX.*YY.*ZZ+WW.^3.*XX.*YY.* ...

ZZ+2.*phi.*WW.^3.*XX.*YY.*ZZ+phi.^2.*WW.^3.*XX.*YY.*ZZ)).*(27.*(WW+(-2) ...

.*phi.*WW+phi.^2.*WW+2.*phi.*WW.^2+(-2).*phi.^2.*WW.^2+phi.^2.*WW.^3+( ...

-1).*XX+2.*phi.*XX+(-1).*phi.^2.*XX+(-2).*phi.*WW.*XX+2.*phi.^2.*WW.*XX+ ...

(-1).*phi.^2.*WW.^2.*XX+phi.*WW.*YY+(-1).*phi.^2.*WW.*YY+(-1).*WW.^2.* ...

YY+2.*phi.^2.*WW.^2.*YY+(-1).*phi.*WW.^3.*YY+(-1).*phi.^2.*WW.^3.*YY+( ...

-1).*phi.*XX.*YY+phi.^2.*XX.*YY+WW.*XX.*YY+(-2).*phi.^2.*WW.*XX.*YY+ ...

phi.*WW.^2.*XX.*YY+phi.^2.*WW.^2.*XX.*YY+phi.*WW.*ZZ+(-1).*phi.^2.*WW.* ...

ZZ+(-1).*WW.^2.*ZZ+2.*phi.^2.*WW.^2.*ZZ+(-1).*phi.*WW.^3.*ZZ+(-1).* ...

phi.^2.*WW.^3.*ZZ+(-1).*phi.*XX.*ZZ+phi.^2.*XX.*ZZ+WW.*XX.*ZZ+(-2).* ...

phi.^2.*WW.*XX.*ZZ+phi.*WW.^2.*XX.*ZZ+phi.^2.*WW.^2.*XX.*ZZ+phi.^2.*WW.* ...

YY.*ZZ+(-2).*phi.*WW.^2.*YY.*ZZ+(-2).*phi.^2.*WW.^2.*YY.*ZZ+WW.^3.*YY.* ...

ZZ+2.*phi.*WW.^3.*YY.*ZZ+phi.^2.*WW.^3.*YY.*ZZ+(-1).*phi.^2.*XX.*YY.*ZZ+ ...

2.*phi.*WW.*XX.*YY.*ZZ+2.*phi.^2.*WW.*XX.*YY.*ZZ+(-1).*WW.^2.*XX.*YY.* ...

ZZ+(-2).*phi.*WW.^2.*XX.*YY.*ZZ+(-1).*phi.^2.*WW.^2.*XX.*YY.*ZZ).*((-3) ...

.*WW.^3+2.*phi.*WW.^3+(-1).*WW.^4+(-2).*phi.*WW.^4+3.*WW.^2.*XX+(-2).* ...

phi.*WW.^2.*XX+WW.^3.*XX+2.*phi.*WW.^3.*XX+(-1).*WW.^2.*YY+2.*phi.* ...

WW.^2.*YY+WW.^3.*YY+(-6).*phi.*WW.^3.*YY+4.*WW.^4.*YY+4.*phi.*WW.^4.*YY+ ...

(-3).*WW.^3.*XX.*YY+2.*phi.*WW.^3.*XX.*YY+(-1).*WW.^4.*XX.*YY+(-2).* ...

phi.*WW.^4.*XX.*YY+WW.^2.*ZZ+(-2).*phi.*WW.^2.*ZZ+3.*WW.^3.*ZZ+2.*phi.* ...

WW.^3.*ZZ+(-4).*WW.^2.*XX.*ZZ+4.*phi.*WW.^2.*XX.*ZZ+(-1).*WW.^3.*XX.*ZZ+ ...

(-6).*phi.*WW.^3.*XX.*ZZ+WW.^4.*XX.*ZZ+2.*phi.*WW.^4.*XX.*ZZ+(-1).* ...

WW.^3.*YY.*ZZ+2.*phi.*WW.^3.*YY.*ZZ+(-3).*WW.^4.*YY.*ZZ+(-2).*phi.* ...

WW.^4.*YY.*ZZ+WW.^2.*XX.*YY.*ZZ+(-2).*phi.*WW.^2.*XX.*YY.*ZZ+3.*WW.^3.* ...

XX.*YY.*ZZ+2.*phi.*WW.^3.*XX.*YY.*ZZ).^2+(-72).*(WW+(-2).*phi.*WW+ ...

phi.^2.*WW+2.*phi.*WW.^2+(-2).*phi.^2.*WW.^2+phi.^2.*WW.^3+(-1).*XX+2.* ...

phi.*XX+(-1).*phi.^2.*XX+(-2).*phi.*WW.*XX+2.*phi.^2.*WW.*XX+(-1).* ...

phi.^2.*WW.^2.*XX+phi.*WW.*YY+(-1).*phi.^2.*WW.*YY+(-1).*WW.^2.*YY+2.* ...

phi.^2.*WW.^2.*YY+(-1).*phi.*WW.^3.*YY+(-1).*phi.^2.*WW.^3.*YY+(-1).* ...

phi.*XX.*YY+phi.^2.*XX.*YY+WW.*XX.*YY+(-2).*phi.^2.*WW.*XX.*YY+phi.* ...

WW.^2.*XX.*YY+phi.^2.*WW.^2.*XX.*YY+phi.*WW.*ZZ+(-1).*phi.^2.*WW.*ZZ+( ...

-1).*WW.^2.*ZZ+2.*phi.^2.*WW.^2.*ZZ+(-1).*phi.*WW.^3.*ZZ+(-1).*phi.^2.* ...

WW.^3.*ZZ+(-1).*phi.*XX.*ZZ+phi.^2.*XX.*ZZ+WW.*XX.*ZZ+(-2).*phi.^2.*WW.* ...

XX.*ZZ+phi.*WW.^2.*XX.*ZZ+phi.^2.*WW.^2.*XX.*ZZ+phi.^2.*WW.*YY.*ZZ+(-2) ...

.*phi.*WW.^2.*YY.*ZZ+(-2).*phi.^2.*WW.^2.*YY.*ZZ+WW.^3.*YY.*ZZ+2.*phi.* ...

WW.^3.*YY.*ZZ+phi.^2.*WW.^3.*YY.*ZZ+(-1).*phi.^2.*XX.*YY.*ZZ+2.*phi.* ...

WW.*XX.*YY.*ZZ+2.*phi.^2.*WW.*XX.*YY.*ZZ+(-1).*WW.^2.*XX.*YY.*ZZ+(-2).* ...

phi.*WW.^2.*XX.*YY.*ZZ+(-1).*phi.^2.*WW.^2.*XX.*YY.*ZZ).*(WW.^4+(-1).* ...

WW.^3.*XX+WW.^3.*YY+(-2).*WW.^4.*YY+WW.^4.*XX.*YY+(-1).*WW.^3.*ZZ+2.* ...

WW.^3.*XX.*ZZ+(-1).*WW.^4.*XX.*ZZ+WW.^4.*YY.*ZZ+(-1).*WW.^3.*XX.*YY.*ZZ) ...

.*(3.*WW.^2+(-4).*phi.*WW.^2+phi.^2.*WW.^2+3.*WW.^3+2.*phi.*WW.^3+(-2).* ...

phi.^2.*WW.^3+2.*phi.*WW.^4+phi.^2.*WW.^4+(-3).*WW.*XX+4.*phi.*WW.*XX+( ...

-1).*phi.^2.*WW.*XX+(-3).*WW.^2.*XX+(-2).*phi.*WW.^2.*XX+2.*phi.^2.* ...

WW.^2.*XX+(-2).*phi.*WW.^3.*XX+(-1).*phi.^2.*WW.^3.*XX+(-1).*phi.*WW.* ...

YY+phi.^2.*WW.*YY+WW.^2.*YY+2.*phi.*WW.^2.*YY+(-4).*phi.^2.*WW.^2.*YY+( ...

-5).*WW.^3.*YY+5.*phi.*WW.^3.*YY+5.*phi.^2.*WW.^3.*YY+(-2).*WW.^4.*YY+( ...

-6).*phi.*WW.^4.*YY+(-2).*phi.^2.*WW.^4.*YY+3.*WW.^2.*XX.*YY+(-5).*phi.* ...

WW.^2.*XX.*YY+phi.^2.*WW.^2.*XX.*YY+3.*WW.^3.*XX.*YY+4.*phi.*WW.^3.*XX.* ...

YY+(-2).*phi.^2.*WW.^3.*XX.*YY+phi.*WW.^4.*XX.*YY+phi.^2.*WW.^4.*XX.*YY+ ...

phi.*WW.*ZZ+(-1).*phi.^2.*WW.*ZZ+(-3).*WW.^2.*ZZ+4.*phi.*WW.^2.*ZZ+2.* ...

phi.^2.*WW.^2.*ZZ+(-3).*WW.^3.*ZZ+(-5).*phi.*WW.^3.*ZZ+(-1).*phi.^2.* ...

WW.^3.*ZZ+2.*WW.*XX.*ZZ+(-6).*phi.*WW.*XX.*ZZ+2.*phi.^2.*WW.*XX.*ZZ+5.* ...

WW.^2.*XX.*ZZ+5.*phi.*WW.^2.*XX.*ZZ+(-5).*phi.^2.*WW.^2.*XX.*ZZ+(-1).* ...

WW.^3.*XX.*ZZ+2.*phi.*WW.^3.*XX.*ZZ+4.*phi.^2.*WW.^3.*XX.*ZZ+(-1).*phi.* ...

WW.^4.*XX.*ZZ+(-1).*phi.^2.*WW.^4.*XX.*ZZ+(-2).*phi.*WW.^2.*YY.*ZZ+ ...

phi.^2.*WW.^2.*YY.*ZZ+3.*WW.^3.*YY.*ZZ+(-2).*phi.*WW.^3.*YY.*ZZ+(-2).* ...

phi.^2.*WW.^3.*YY.*ZZ+3.*WW.^4.*YY.*ZZ+4.*phi.*WW.^4.*YY.*ZZ+phi.^2.* ...

WW.^4.*YY.*ZZ+2.*phi.*WW.*XX.*YY.*ZZ+(-1).*phi.^2.*WW.*XX.*YY.*ZZ+(-3).* ...

WW.^2.*XX.*YY.*ZZ+2.*phi.*WW.^2.*XX.*YY.*ZZ+2.*phi.^2.*WW.^2.*XX.*YY.* ...

ZZ+(-3).*WW.^3.*XX.*YY.*ZZ+(-4).*phi.*WW.^3.*XX.*YY.*ZZ+(-1).*phi.^2.* ...

WW.^3.*XX.*YY.*ZZ)+2.*(3.*WW.^2+(-4).*phi.*WW.^2+phi.^2.*WW.^2+3.*WW.^3+ ...

2.*phi.*WW.^3+(-2).*phi.^2.*WW.^3+2.*phi.*WW.^4+phi.^2.*WW.^4+(-3).*WW.* ...

XX+4.*phi.*WW.*XX+(-1).*phi.^2.*WW.*XX+(-3).*WW.^2.*XX+(-2).*phi.* ...

WW.^2.*XX+2.*phi.^2.*WW.^2.*XX+(-2).*phi.*WW.^3.*XX+(-1).*phi.^2.* ...

WW.^3.*XX+(-1).*phi.*WW.*YY+phi.^2.*WW.*YY+WW.^2.*YY+2.*phi.*WW.^2.*YY+( ...

-4).*phi.^2.*WW.^2.*YY+(-5).*WW.^3.*YY+5.*phi.*WW.^3.*YY+5.*phi.^2.* ...

WW.^3.*YY+(-2).*WW.^4.*YY+(-6).*phi.*WW.^4.*YY+(-2).*phi.^2.*WW.^4.*YY+ ...

3.*WW.^2.*XX.*YY+(-5).*phi.*WW.^2.*XX.*YY+phi.^2.*WW.^2.*XX.*YY+3.* ...

WW.^3.*XX.*YY+4.*phi.*WW.^3.*XX.*YY+(-2).*phi.^2.*WW.^3.*XX.*YY+phi.* ...

WW.^4.*XX.*YY+phi.^2.*WW.^4.*XX.*YY+phi.*WW.*ZZ+(-1).*phi.^2.*WW.*ZZ+( ...

-3).*WW.^2.*ZZ+4.*phi.*WW.^2.*ZZ+2.*phi.^2.*WW.^2.*ZZ+(-3).*WW.^3.*ZZ+( ...

-5).*phi.*WW.^3.*ZZ+(-1).*phi.^2.*WW.^3.*ZZ+2.*WW.*XX.*ZZ+(-6).*phi.* ...

WW.*XX.*ZZ+2.*phi.^2.*WW.*XX.*ZZ+5.*WW.^2.*XX.*ZZ+5.*phi.*WW.^2.*XX.*ZZ+ ...

(-5).*phi.^2.*WW.^2.*XX.*ZZ+(-1).*WW.^3.*XX.*ZZ+2.*phi.*WW.^3.*XX.*ZZ+ ...

4.*phi.^2.*WW.^3.*XX.*ZZ+(-1).*phi.*WW.^4.*XX.*ZZ+(-1).*phi.^2.*WW.^4.* ...

XX.*ZZ+(-2).*phi.*WW.^2.*YY.*ZZ+phi.^2.*WW.^2.*YY.*ZZ+3.*WW.^3.*YY.*ZZ+( ...

-2).*phi.*WW.^3.*YY.*ZZ+(-2).*phi.^2.*WW.^3.*YY.*ZZ+3.*WW.^4.*YY.*ZZ+4.* ...

phi.*WW.^4.*YY.*ZZ+phi.^2.*WW.^4.*YY.*ZZ+2.*phi.*WW.*XX.*YY.*ZZ+(-1).* ...

phi.^2.*WW.*XX.*YY.*ZZ+(-3).*WW.^2.*XX.*YY.*ZZ+2.*phi.*WW.^2.*XX.*YY.* ...

ZZ+2.*phi.^2.*WW.^2.*XX.*YY.*ZZ+(-3).*WW.^3.*XX.*YY.*ZZ+(-4).*phi.* ...

WW.^3.*XX.*YY.*ZZ+(-1).*phi.^2.*WW.^3.*XX.*YY.*ZZ).^3+(-9).*((-3).* ...

WW.^3+2.*phi.*WW.^3+(-1).*WW.^4+(-2).*phi.*WW.^4+3.*WW.^2.*XX+(-2).* ...

phi.*WW.^2.*XX+WW.^3.*XX+2.*phi.*WW.^3.*XX+(-1).*WW.^2.*YY+2.*phi.* ...

WW.^2.*YY+WW.^3.*YY+(-6).*phi.*WW.^3.*YY+4.*WW.^4.*YY+4.*phi.*WW.^4.*YY+ ...

(-3).*WW.^3.*XX.*YY+2.*phi.*WW.^3.*XX.*YY+(-1).*WW.^4.*XX.*YY+(-2).* ...

phi.*WW.^4.*XX.*YY+WW.^2.*ZZ+(-2).*phi.*WW.^2.*ZZ+3.*WW.^3.*ZZ+2.*phi.* ...

WW.^3.*ZZ+(-4).*WW.^2.*XX.*ZZ+4.*phi.*WW.^2.*XX.*ZZ+(-1).*WW.^3.*XX.*ZZ+ ...

(-6).*phi.*WW.^3.*XX.*ZZ+WW.^4.*XX.*ZZ+2.*phi.*WW.^4.*XX.*ZZ+(-1).* ...

WW.^3.*YY.*ZZ+2.*phi.*WW.^3.*YY.*ZZ+(-3).*WW.^4.*YY.*ZZ+(-2).*phi.* ...

WW.^4.*YY.*ZZ+WW.^2.*XX.*YY.*ZZ+(-2).*phi.*WW.^2.*XX.*YY.*ZZ+3.*WW.^3.* ...

XX.*YY.*ZZ+2.*phi.*WW.^3.*XX.*YY.*ZZ).*(3.*WW.^2+(-4).*phi.*WW.^2+ ...

phi.^2.*WW.^2+3.*WW.^3+2.*phi.*WW.^3+(-2).*phi.^2.*WW.^3+2.*phi.*WW.^4+ ...

phi.^2.*WW.^4+(-3).*WW.*XX+4.*phi.*WW.*XX+(-1).*phi.^2.*WW.*XX+(-3).* ...

WW.^2.*XX+(-2).*phi.*WW.^2.*XX+2.*phi.^2.*WW.^2.*XX+(-2).*phi.*WW.^3.* ...

XX+(-1).*phi.^2.*WW.^3.*XX+(-1).*phi.*WW.*YY+phi.^2.*WW.*YY+WW.^2.*YY+ ...

2.*phi.*WW.^2.*YY+(-4).*phi.^2.*WW.^2.*YY+(-5).*WW.^3.*YY+5.*phi.* ...

WW.^3.*YY+5.*phi.^2.*WW.^3.*YY+(-2).*WW.^4.*YY+(-6).*phi.*WW.^4.*YY+(-2) ...

.*phi.^2.*WW.^4.*YY+3.*WW.^2.*XX.*YY+(-5).*phi.*WW.^2.*XX.*YY+phi.^2.* ...

WW.^2.*XX.*YY+3.*WW.^3.*XX.*YY+4.*phi.*WW.^3.*XX.*YY+(-2).*phi.^2.* ...

WW.^3.*XX.*YY+phi.*WW.^4.*XX.*YY+phi.^2.*WW.^4.*XX.*YY+phi.*WW.*ZZ+(-1) ...

.*phi.^2.*WW.*ZZ+(-3).*WW.^2.*ZZ+4.*phi.*WW.^2.*ZZ+2.*phi.^2.*WW.^2.*ZZ+ ...

(-3).*WW.^3.*ZZ+(-5).*phi.*WW.^3.*ZZ+(-1).*phi.^2.*WW.^3.*ZZ+2.*WW.*XX.* ...

ZZ+(-6).*phi.*WW.*XX.*ZZ+2.*phi.^2.*WW.*XX.*ZZ+5.*WW.^2.*XX.*ZZ+5.*phi.* ...

WW.^2.*XX.*ZZ+(-5).*phi.^2.*WW.^2.*XX.*ZZ+(-1).*WW.^3.*XX.*ZZ+2.*phi.* ...

WW.^3.*XX.*ZZ+4.*phi.^2.*WW.^3.*XX.*ZZ+(-1).*phi.*WW.^4.*XX.*ZZ+(-1).* ...

phi.^2.*WW.^4.*XX.*ZZ+(-2).*phi.*WW.^2.*YY.*ZZ+phi.^2.*WW.^2.*YY.*ZZ+3.* ...

WW.^3.*YY.*ZZ+(-2).*phi.*WW.^3.*YY.*ZZ+(-2).*phi.^2.*WW.^3.*YY.*ZZ+3.* ...

WW.^4.*YY.*ZZ+4.*phi.*WW.^4.*YY.*ZZ+phi.^2.*WW.^4.*YY.*ZZ+2.*phi.*WW.* ...

XX.*YY.*ZZ+(-1).*phi.^2.*WW.*XX.*YY.*ZZ+(-3).*WW.^2.*XX.*YY.*ZZ+2.*phi.* ...

WW.^2.*XX.*YY.*ZZ+2.*phi.^2.*WW.^2.*XX.*YY.*ZZ+(-3).*WW.^3.*XX.*YY.*ZZ+( ...

-4).*phi.*WW.^3.*XX.*YY.*ZZ+(-1).*phi.^2.*WW.^3.*XX.*YY.*ZZ).*((-1).*WW+ ...

2.*phi.*WW+(-1).*phi.^2.*WW+(-3).*WW.^2+2.*phi.*WW.^2+phi.^2.*WW.^2+(-4) ...

.*phi.*WW.^3+phi.^2.*WW.^3+(-1).*phi.^2.*WW.^4+XX+(-2).*phi.*XX+phi.^2.* ...

XX+3.*WW.*XX+(-2).*phi.*WW.*XX+(-1).*phi.^2.*WW.*XX+4.*phi.*WW.^2.*XX+( ...

-1).*phi.^2.*WW.^2.*XX+phi.^2.*WW.^3.*XX+WW.^2.*YY+(-4).*phi.*WW.^2.*YY+ ...

2.*phi.^2.*WW.^2.*YY+3.*WW.^3.*YY+2.*phi.*WW.^3.*YY+(-4).*phi.^2.* ...

WW.^3.*YY+2.*phi.*WW.^4.*YY+2.*phi.^2.*WW.^4.*YY+(-1).*WW.*XX.*YY+4.* ...

phi.*WW.*XX.*YY+(-2).*phi.^2.*WW.*XX.*YY+(-3).*WW.^2.*XX.*YY+(-2).*phi.* ...

WW.^2.*XX.*YY+4.*phi.^2.*WW.^2.*XX.*YY+(-2).*phi.*WW.^3.*XX.*YY+(-2).* ...

phi.^2.*WW.^3.*XX.*YY+(-2).*phi.*WW.*ZZ+2.*phi.^2.*WW.*ZZ+3.*WW.^2.*ZZ+( ...

-2).*phi.*WW.^2.*ZZ+(-4).*phi.^2.*WW.^2.*ZZ+WW.^3.*ZZ+4.*phi.*WW.^3.*ZZ+ ...

2.*phi.^2.*WW.^3.*ZZ+2.*phi.*XX.*ZZ+(-2).*phi.^2.*XX.*ZZ+(-3).*WW.*XX.* ...

ZZ+2.*phi.*WW.*XX.*ZZ+4.*phi.^2.*WW.*XX.*ZZ+(-1).*WW.^2.*XX.*ZZ+(-4).* ...

phi.*WW.^2.*XX.*ZZ+(-2).*phi.^2.*WW.^2.*XX.*ZZ+(-1).*phi.^2.*WW.*YY.*ZZ+ ...

4.*phi.*WW.^2.*YY.*ZZ+phi.^2.*WW.^2.*YY.*ZZ+(-3).*WW.^3.*YY.*ZZ+(-2).* ...

phi.*WW.^3.*YY.*ZZ+phi.^2.*WW.^3.*YY.*ZZ+(-1).*WW.^4.*YY.*ZZ+(-2).*phi.* ...

WW.^4.*YY.*ZZ+(-1).*phi.^2.*WW.^4.*YY.*ZZ+phi.^2.*XX.*YY.*ZZ+(-4).*phi.* ...

WW.*XX.*YY.*ZZ+(-1).*phi.^2.*WW.*XX.*YY.*ZZ+3.*WW.^2.*XX.*YY.*ZZ+2.* ...

phi.*WW.^2.*XX.*YY.*ZZ+(-1).*phi.^2.*WW.^2.*XX.*YY.*ZZ+WW.^3.*XX.*YY.* ...

ZZ+2.*phi.*WW.^3.*XX.*YY.*ZZ+phi.^2.*WW.^3.*XX.*YY.*ZZ)+27.*(WW.^4+(-1) ...

.*WW.^3.*XX+WW.^3.*YY+(-2).*WW.^4.*YY+WW.^4.*XX.*YY+(-1).*WW.^3.*ZZ+2.* ...

WW.^3.*XX.*ZZ+(-1).*WW.^4.*XX.*ZZ+WW.^4.*YY.*ZZ+(-1).*WW.^3.*XX.*YY.*ZZ) ...

.*((-1).*WW+2.*phi.*WW+(-1).*phi.^2.*WW+(-3).*WW.^2+2.*phi.*WW.^2+ ...

phi.^2.*WW.^2+(-4).*phi.*WW.^3+phi.^2.*WW.^3+(-1).*phi.^2.*WW.^4+XX+(-2) ...

.*phi.*XX+phi.^2.*XX+3.*WW.*XX+(-2).*phi.*WW.*XX+(-1).*phi.^2.*WW.*XX+ ...

4.*phi.*WW.^2.*XX+(-1).*phi.^2.*WW.^2.*XX+phi.^2.*WW.^3.*XX+WW.^2.*YY+( ...

-4).*phi.*WW.^2.*YY+2.*phi.^2.*WW.^2.*YY+3.*WW.^3.*YY+2.*phi.*WW.^3.*YY+ ...

(-4).*phi.^2.*WW.^3.*YY+2.*phi.*WW.^4.*YY+2.*phi.^2.*WW.^4.*YY+(-1).* ...

WW.*XX.*YY+4.*phi.*WW.*XX.*YY+(-2).*phi.^2.*WW.*XX.*YY+(-3).*WW.^2.*XX.* ...

YY+(-2).*phi.*WW.^2.*XX.*YY+4.*phi.^2.*WW.^2.*XX.*YY+(-2).*phi.*WW.^3.* ...

XX.*YY+(-2).*phi.^2.*WW.^3.*XX.*YY+(-2).*phi.*WW.*ZZ+2.*phi.^2.*WW.*ZZ+ ...

3.*WW.^2.*ZZ+(-2).*phi.*WW.^2.*ZZ+(-4).*phi.^2.*WW.^2.*ZZ+WW.^3.*ZZ+4.* ...

phi.*WW.^3.*ZZ+2.*phi.^2.*WW.^3.*ZZ+2.*phi.*XX.*ZZ+(-2).*phi.^2.*XX.*ZZ+ ...

(-3).*WW.*XX.*ZZ+2.*phi.*WW.*XX.*ZZ+4.*phi.^2.*WW.*XX.*ZZ+(-1).*WW.^2.* ...

XX.*ZZ+(-4).*phi.*WW.^2.*XX.*ZZ+(-2).*phi.^2.*WW.^2.*XX.*ZZ+(-1).* ...

phi.^2.*WW.*YY.*ZZ+4.*phi.*WW.^2.*YY.*ZZ+phi.^2.*WW.^2.*YY.*ZZ+(-3).* ...

WW.^3.*YY.*ZZ+(-2).*phi.*WW.^3.*YY.*ZZ+phi.^2.*WW.^3.*YY.*ZZ+(-1).* ...

WW.^4.*YY.*ZZ+(-2).*phi.*WW.^4.*YY.*ZZ+(-1).*phi.^2.*WW.^4.*YY.*ZZ+ ...

phi.^2.*XX.*YY.*ZZ+(-4).*phi.*WW.*XX.*YY.*ZZ+(-1).*phi.^2.*WW.*XX.*YY.* ...

ZZ+3.*WW.^2.*XX.*YY.*ZZ+2.*phi.*WW.^2.*XX.*YY.*ZZ+(-1).*phi.^2.*WW.^2.* ...

XX.*YY.*ZZ+WW.^3.*XX.*YY.*ZZ+2.*phi.*WW.^3.*XX.*YY.*ZZ+phi.^2.*WW.^3.* ...

XX.*YY.*ZZ).^2+((-4).*(12.*(WW+(-2).*phi.*WW+phi.^2.*WW+2.*phi.*WW.^2+( ...

-2).*phi.^2.*WW.^2+phi.^2.*WW.^3+(-1).*XX+2.*phi.*XX+(-1).*phi.^2.*XX+( ...

-2).*phi.*WW.*XX+2.*phi.^2.*WW.*XX+(-1).*phi.^2.*WW.^2.*XX+phi.*WW.*YY+( ...

-1).*phi.^2.*WW.*YY+(-1).*WW.^2.*YY+2.*phi.^2.*WW.^2.*YY+(-1).*phi.* ...

WW.^3.*YY+(-1).*phi.^2.*WW.^3.*YY+(-1).*phi.*XX.*YY+phi.^2.*XX.*YY+WW.* ...

XX.*YY+(-2).*phi.^2.*WW.*XX.*YY+phi.*WW.^2.*XX.*YY+phi.^2.*WW.^2.*XX.* ...

YY+phi.*WW.*ZZ+(-1).*phi.^2.*WW.*ZZ+(-1).*WW.^2.*ZZ+2.*phi.^2.*WW.^2.* ...

ZZ+(-1).*phi.*WW.^3.*ZZ+(-1).*phi.^2.*WW.^3.*ZZ+(-1).*phi.*XX.*ZZ+ ...

phi.^2.*XX.*ZZ+WW.*XX.*ZZ+(-2).*phi.^2.*WW.*XX.*ZZ+phi.*WW.^2.*XX.*ZZ+ ...

phi.^2.*WW.^2.*XX.*ZZ+phi.^2.*WW.*YY.*ZZ+(-2).*phi.*WW.^2.*YY.*ZZ+(-2).* ...

phi.^2.*WW.^2.*YY.*ZZ+WW.^3.*YY.*ZZ+2.*phi.*WW.^3.*YY.*ZZ+phi.^2.* ...

WW.^3.*YY.*ZZ+(-1).*phi.^2.*XX.*YY.*ZZ+2.*phi.*WW.*XX.*YY.*ZZ+2.* ...

phi.^2.*WW.*XX.*YY.*ZZ+(-1).*WW.^2.*XX.*YY.*ZZ+(-2).*phi.*WW.^2.*XX.* ...

YY.*ZZ+(-1).*phi.^2.*WW.^2.*XX.*YY.*ZZ).*(WW.^4+(-1).*WW.^3.*XX+WW.^3.* ...

YY+(-2).*WW.^4.*YY+WW.^4.*XX.*YY+(-1).*WW.^3.*ZZ+2.*WW.^3.*XX.*ZZ+(-1).* ...

WW.^4.*XX.*ZZ+WW.^4.*YY.*ZZ+(-1).*WW.^3.*XX.*YY.*ZZ)+(3.*WW.^2+(-4).* ...

phi.*WW.^2+phi.^2.*WW.^2+3.*WW.^3+2.*phi.*WW.^3+(-2).*phi.^2.*WW.^3+2.* ...

phi.*WW.^4+phi.^2.*WW.^4+(-3).*WW.*XX+4.*phi.*WW.*XX+(-1).*phi.^2.*WW.* ...

XX+(-3).*WW.^2.*XX+(-2).*phi.*WW.^2.*XX+2.*phi.^2.*WW.^2.*XX+(-2).*phi.* ...

WW.^3.*XX+(-1).*phi.^2.*WW.^3.*XX+(-1).*phi.*WW.*YY+phi.^2.*WW.*YY+ ...

WW.^2.*YY+2.*phi.*WW.^2.*YY+(-4).*phi.^2.*WW.^2.*YY+(-5).*WW.^3.*YY+5.* ...

phi.*WW.^3.*YY+5.*phi.^2.*WW.^3.*YY+(-2).*WW.^4.*YY+(-6).*phi.*WW.^4.* ...

YY+(-2).*phi.^2.*WW.^4.*YY+3.*WW.^2.*XX.*YY+(-5).*phi.*WW.^2.*XX.*YY+ ...

phi.^2.*WW.^2.*XX.*YY+3.*WW.^3.*XX.*YY+4.*phi.*WW.^3.*XX.*YY+(-2).* ...

phi.^2.*WW.^3.*XX.*YY+phi.*WW.^4.*XX.*YY+phi.^2.*WW.^4.*XX.*YY+phi.*WW.* ...

ZZ+(-1).*phi.^2.*WW.*ZZ+(-3).*WW.^2.*ZZ+4.*phi.*WW.^2.*ZZ+2.*phi.^2.* ...

WW.^2.*ZZ+(-3).*WW.^3.*ZZ+(-5).*phi.*WW.^3.*ZZ+(-1).*phi.^2.*WW.^3.*ZZ+ ...

2.*WW.*XX.*ZZ+(-6).*phi.*WW.*XX.*ZZ+2.*phi.^2.*WW.*XX.*ZZ+5.*WW.^2.*XX.* ...

ZZ+5.*phi.*WW.^2.*XX.*ZZ+(-5).*phi.^2.*WW.^2.*XX.*ZZ+(-1).*WW.^3.*XX.* ...

ZZ+2.*phi.*WW.^3.*XX.*ZZ+4.*phi.^2.*WW.^3.*XX.*ZZ+(-1).*phi.*WW.^4.*XX.* ...

ZZ+(-1).*phi.^2.*WW.^4.*XX.*ZZ+(-2).*phi.*WW.^2.*YY.*ZZ+phi.^2.*WW.^2.* ...

YY.*ZZ+3.*WW.^3.*YY.*ZZ+(-2).*phi.*WW.^3.*YY.*ZZ+(-2).*phi.^2.*WW.^3.* ...

YY.*ZZ+3.*WW.^4.*YY.*ZZ+4.*phi.*WW.^4.*YY.*ZZ+phi.^2.*WW.^4.*YY.*ZZ+2.* ...

phi.*WW.*XX.*YY.*ZZ+(-1).*phi.^2.*WW.*XX.*YY.*ZZ+(-3).*WW.^2.*XX.*YY.* ...

ZZ+2.*phi.*WW.^2.*XX.*YY.*ZZ+2.*phi.^2.*WW.^2.*XX.*YY.*ZZ+(-3).*WW.^3.* ...

XX.*YY.*ZZ+(-4).*phi.*WW.^3.*XX.*YY.*ZZ+(-1).*phi.^2.*WW.^3.*XX.*YY.*ZZ) ...

.^2+(-3).*((-3).*WW.^3+2.*phi.*WW.^3+(-1).*WW.^4+(-2).*phi.*WW.^4+3.* ...

WW.^2.*XX+(-2).*phi.*WW.^2.*XX+WW.^3.*XX+2.*phi.*WW.^3.*XX+(-1).*WW.^2.* ...

YY+2.*phi.*WW.^2.*YY+WW.^3.*YY+(-6).*phi.*WW.^3.*YY+4.*WW.^4.*YY+4.* ...

phi.*WW.^4.*YY+(-3).*WW.^3.*XX.*YY+2.*phi.*WW.^3.*XX.*YY+(-1).*WW.^4.* ...

XX.*YY+(-2).*phi.*WW.^4.*XX.*YY+WW.^2.*ZZ+(-2).*phi.*WW.^2.*ZZ+3.* ...

WW.^3.*ZZ+2.*phi.*WW.^3.*ZZ+(-4).*WW.^2.*XX.*ZZ+4.*phi.*WW.^2.*XX.*ZZ+( ...

-1).*WW.^3.*XX.*ZZ+(-6).*phi.*WW.^3.*XX.*ZZ+WW.^4.*XX.*ZZ+2.*phi.* ...

WW.^4.*XX.*ZZ+(-1).*WW.^3.*YY.*ZZ+2.*phi.*WW.^3.*YY.*ZZ+(-3).*WW.^4.* ...

YY.*ZZ+(-2).*phi.*WW.^4.*YY.*ZZ+WW.^2.*XX.*YY.*ZZ+(-2).*phi.*WW.^2.*XX.* ...

YY.*ZZ+3.*WW.^3.*XX.*YY.*ZZ+2.*phi.*WW.^3.*XX.*YY.*ZZ).*((-1).*WW+2.* ...

phi.*WW+(-1).*phi.^2.*WW+(-3).*WW.^2+2.*phi.*WW.^2+phi.^2.*WW.^2+(-4).* ...

phi.*WW.^3+phi.^2.*WW.^3+(-1).*phi.^2.*WW.^4+XX+(-2).*phi.*XX+phi.^2.* ...

XX+3.*WW.*XX+(-2).*phi.*WW.*XX+(-1).*phi.^2.*WW.*XX+4.*phi.*WW.^2.*XX+( ...

-1).*phi.^2.*WW.^2.*XX+phi.^2.*WW.^3.*XX+WW.^2.*YY+(-4).*phi.*WW.^2.*YY+ ...

2.*phi.^2.*WW.^2.*YY+3.*WW.^3.*YY+2.*phi.*WW.^3.*YY+(-4).*phi.^2.* ...

WW.^3.*YY+2.*phi.*WW.^4.*YY+2.*phi.^2.*WW.^4.*YY+(-1).*WW.*XX.*YY+4.* ...

phi.*WW.*XX.*YY+(-2).*phi.^2.*WW.*XX.*YY+(-3).*WW.^2.*XX.*YY+(-2).*phi.* ...

WW.^2.*XX.*YY+4.*phi.^2.*WW.^2.*XX.*YY+(-2).*phi.*WW.^3.*XX.*YY+(-2).* ...

phi.^2.*WW.^3.*XX.*YY+(-2).*phi.*WW.*ZZ+2.*phi.^2.*WW.*ZZ+3.*WW.^2.*ZZ+( ...

-2).*phi.*WW.^2.*ZZ+(-4).*phi.^2.*WW.^2.*ZZ+WW.^3.*ZZ+4.*phi.*WW.^3.*ZZ+ ...

2.*phi.^2.*WW.^3.*ZZ+2.*phi.*XX.*ZZ+(-2).*phi.^2.*XX.*ZZ+(-3).*WW.*XX.* ...

ZZ+2.*phi.*WW.*XX.*ZZ+4.*phi.^2.*WW.*XX.*ZZ+(-1).*WW.^2.*XX.*ZZ+(-4).* ...

phi.*WW.^2.*XX.*ZZ+(-2).*phi.^2.*WW.^2.*XX.*ZZ+(-1).*phi.^2.*WW.*YY.*ZZ+ ...

4.*phi.*WW.^2.*YY.*ZZ+phi.^2.*WW.^2.*YY.*ZZ+(-3).*WW.^3.*YY.*ZZ+(-2).* ...

phi.*WW.^3.*YY.*ZZ+phi.^2.*WW.^3.*YY.*ZZ+(-1).*WW.^4.*YY.*ZZ+(-2).*phi.* ...

WW.^4.*YY.*ZZ+(-1).*phi.^2.*WW.^4.*YY.*ZZ+phi.^2.*XX.*YY.*ZZ+(-4).*phi.* ...

WW.*XX.*YY.*ZZ+(-1).*phi.^2.*WW.*XX.*YY.*ZZ+3.*WW.^2.*XX.*YY.*ZZ+2.* ...

phi.*WW.^2.*XX.*YY.*ZZ+(-1).*phi.^2.*WW.^2.*XX.*YY.*ZZ+WW.^3.*XX.*YY.* ...

ZZ+2.*phi.*WW.^3.*XX.*YY.*ZZ+phi.^2.*WW.^3.*XX.*YY.*ZZ)).^3+(27.*(WW+( ...

-2).*phi.*WW+phi.^2.*WW+2.*phi.*WW.^2+(-2).*phi.^2.*WW.^2+phi.^2.*WW.^3+ ...

(-1).*XX+2.*phi.*XX+(-1).*phi.^2.*XX+(-2).*phi.*WW.*XX+2.*phi.^2.*WW.* ...

XX+(-1).*phi.^2.*WW.^2.*XX+phi.*WW.*YY+(-1).*phi.^2.*WW.*YY+(-1).* ...

WW.^2.*YY+2.*phi.^2.*WW.^2.*YY+(-1).*phi.*WW.^3.*YY+(-1).*phi.^2.* ...

WW.^3.*YY+(-1).*phi.*XX.*YY+phi.^2.*XX.*YY+WW.*XX.*YY+(-2).*phi.^2.*WW.* ...

XX.*YY+phi.*WW.^2.*XX.*YY+phi.^2.*WW.^2.*XX.*YY+phi.*WW.*ZZ+(-1).* ...

phi.^2.*WW.*ZZ+(-1).*WW.^2.*ZZ+2.*phi.^2.*WW.^2.*ZZ+(-1).*phi.*WW.^3.* ...

ZZ+(-1).*phi.^2.*WW.^3.*ZZ+(-1).*phi.*XX.*ZZ+phi.^2.*XX.*ZZ+WW.*XX.*ZZ+( ...

-2).*phi.^2.*WW.*XX.*ZZ+phi.*WW.^2.*XX.*ZZ+phi.^2.*WW.^2.*XX.*ZZ+ ...

phi.^2.*WW.*YY.*ZZ+(-2).*phi.*WW.^2.*YY.*ZZ+(-2).*phi.^2.*WW.^2.*YY.*ZZ+ ...

WW.^3.*YY.*ZZ+2.*phi.*WW.^3.*YY.*ZZ+phi.^2.*WW.^3.*YY.*ZZ+(-1).*phi.^2.* ...

XX.*YY.*ZZ+2.*phi.*WW.*XX.*YY.*ZZ+2.*phi.^2.*WW.*XX.*YY.*ZZ+(-1).* ...

WW.^2.*XX.*YY.*ZZ+(-2).*phi.*WW.^2.*XX.*YY.*ZZ+(-1).*phi.^2.*WW.^2.*XX.* ...

YY.*ZZ).*((-3).*WW.^3+2.*phi.*WW.^3+(-1).*WW.^4+(-2).*phi.*WW.^4+3.* ...

WW.^2.*XX+(-2).*phi.*WW.^2.*XX+WW.^3.*XX+2.*phi.*WW.^3.*XX+(-1).*WW.^2.* ...

YY+2.*phi.*WW.^2.*YY+WW.^3.*YY+(-6).*phi.*WW.^3.*YY+4.*WW.^4.*YY+4.* ...

phi.*WW.^4.*YY+(-3).*WW.^3.*XX.*YY+2.*phi.*WW.^3.*XX.*YY+(-1).*WW.^4.* ...

XX.*YY+(-2).*phi.*WW.^4.*XX.*YY+WW.^2.*ZZ+(-2).*phi.*WW.^2.*ZZ+3.* ...

WW.^3.*ZZ+2.*phi.*WW.^3.*ZZ+(-4).*WW.^2.*XX.*ZZ+4.*phi.*WW.^2.*XX.*ZZ+( ...

-1).*WW.^3.*XX.*ZZ+(-6).*phi.*WW.^3.*XX.*ZZ+WW.^4.*XX.*ZZ+2.*phi.* ...

WW.^4.*XX.*ZZ+(-1).*WW.^3.*YY.*ZZ+2.*phi.*WW.^3.*YY.*ZZ+(-3).*WW.^4.* ...

YY.*ZZ+(-2).*phi.*WW.^4.*YY.*ZZ+WW.^2.*XX.*YY.*ZZ+(-2).*phi.*WW.^2.*XX.* ...

YY.*ZZ+3.*WW.^3.*XX.*YY.*ZZ+2.*phi.*WW.^3.*XX.*YY.*ZZ).^2+(-72).*(WW+( ...

-2).*phi.*WW+phi.^2.*WW+2.*phi.*WW.^2+(-2).*phi.^2.*WW.^2+phi.^2.*WW.^3+ ...

(-1).*XX+2.*phi.*XX+(-1).*phi.^2.*XX+(-2).*phi.*WW.*XX+2.*phi.^2.*WW.* ...

XX+(-1).*phi.^2.*WW.^2.*XX+phi.*WW.*YY+(-1).*phi.^2.*WW.*YY+(-1).* ...

WW.^2.*YY+2.*phi.^2.*WW.^2.*YY+(-1).*phi.*WW.^3.*YY+(-1).*phi.^2.* ...

WW.^3.*YY+(-1).*phi.*XX.*YY+phi.^2.*XX.*YY+WW.*XX.*YY+(-2).*phi.^2.*WW.* ...

XX.*YY+phi.*WW.^2.*XX.*YY+phi.^2.*WW.^2.*XX.*YY+phi.*WW.*ZZ+(-1).* ...

phi.^2.*WW.*ZZ+(-1).*WW.^2.*ZZ+2.*phi.^2.*WW.^2.*ZZ+(-1).*phi.*WW.^3.* ...

ZZ+(-1).*phi.^2.*WW.^3.*ZZ+(-1).*phi.*XX.*ZZ+phi.^2.*XX.*ZZ+WW.*XX.*ZZ+( ...

-2).*phi.^2.*WW.*XX.*ZZ+phi.*WW.^2.*XX.*ZZ+phi.^2.*WW.^2.*XX.*ZZ+ ...

phi.^2.*WW.*YY.*ZZ+(-2).*phi.*WW.^2.*YY.*ZZ+(-2).*phi.^2.*WW.^2.*YY.*ZZ+ ...

WW.^3.*YY.*ZZ+2.*phi.*WW.^3.*YY.*ZZ+phi.^2.*WW.^3.*YY.*ZZ+(-1).*phi.^2.* ...

XX.*YY.*ZZ+2.*phi.*WW.*XX.*YY.*ZZ+2.*phi.^2.*WW.*XX.*YY.*ZZ+(-1).* ...

WW.^2.*XX.*YY.*ZZ+(-2).*phi.*WW.^2.*XX.*YY.*ZZ+(-1).*phi.^2.*WW.^2.*XX.* ...

YY.*ZZ).*(WW.^4+(-1).*WW.^3.*XX+WW.^3.*YY+(-2).*WW.^4.*YY+WW.^4.*XX.*YY+ ...

(-1).*WW.^3.*ZZ+2.*WW.^3.*XX.*ZZ+(-1).*WW.^4.*XX.*ZZ+WW.^4.*YY.*ZZ+(-1) ...

.*WW.^3.*XX.*YY.*ZZ).*(3.*WW.^2+(-4).*phi.*WW.^2+phi.^2.*WW.^2+3.*WW.^3+ ...

2.*phi.*WW.^3+(-2).*phi.^2.*WW.^3+2.*phi.*WW.^4+phi.^2.*WW.^4+(-3).*WW.* ...

XX+4.*phi.*WW.*XX+(-1).*phi.^2.*WW.*XX+(-3).*WW.^2.*XX+(-2).*phi.* ...

WW.^2.*XX+2.*phi.^2.*WW.^2.*XX+(-2).*phi.*WW.^3.*XX+(-1).*phi.^2.* ...

WW.^3.*XX+(-1).*phi.*WW.*YY+phi.^2.*WW.*YY+WW.^2.*YY+2.*phi.*WW.^2.*YY+( ...

-4).*phi.^2.*WW.^2.*YY+(-5).*WW.^3.*YY+5.*phi.*WW.^3.*YY+5.*phi.^2.* ...

WW.^3.*YY+(-2).*WW.^4.*YY+(-6).*phi.*WW.^4.*YY+(-2).*phi.^2.*WW.^4.*YY+ ...

3.*WW.^2.*XX.*YY+(-5).*phi.*WW.^2.*XX.*YY+phi.^2.*WW.^2.*XX.*YY+3.* ...

WW.^3.*XX.*YY+4.*phi.*WW.^3.*XX.*YY+(-2).*phi.^2.*WW.^3.*XX.*YY+phi.* ...

WW.^4.*XX.*YY+phi.^2.*WW.^4.*XX.*YY+phi.*WW.*ZZ+(-1).*phi.^2.*WW.*ZZ+( ...

-3).*WW.^2.*ZZ+4.*phi.*WW.^2.*ZZ+2.*phi.^2.*WW.^2.*ZZ+(-3).*WW.^3.*ZZ+( ...

-5).*phi.*WW.^3.*ZZ+(-1).*phi.^2.*WW.^3.*ZZ+2.*WW.*XX.*ZZ+(-6).*phi.* ...

WW.*XX.*ZZ+2.*phi.^2.*WW.*XX.*ZZ+5.*WW.^2.*XX.*ZZ+5.*phi.*WW.^2.*XX.*ZZ+ ...

(-5).*phi.^2.*WW.^2.*XX.*ZZ+(-1).*WW.^3.*XX.*ZZ+2.*phi.*WW.^3.*XX.*ZZ+ ...

4.*phi.^2.*WW.^3.*XX.*ZZ+(-1).*phi.*WW.^4.*XX.*ZZ+(-1).*phi.^2.*WW.^4.* ...

XX.*ZZ+(-2).*phi.*WW.^2.*YY.*ZZ+phi.^2.*WW.^2.*YY.*ZZ+3.*WW.^3.*YY.*ZZ+( ...

-2).*phi.*WW.^3.*YY.*ZZ+(-2).*phi.^2.*WW.^3.*YY.*ZZ+3.*WW.^4.*YY.*ZZ+4.* ...

phi.*WW.^4.*YY.*ZZ+phi.^2.*WW.^4.*YY.*ZZ+2.*phi.*WW.*XX.*YY.*ZZ+(-1).* ...

phi.^2.*WW.*XX.*YY.*ZZ+(-3).*WW.^2.*XX.*YY.*ZZ+2.*phi.*WW.^2.*XX.*YY.* ...

ZZ+2.*phi.^2.*WW.^2.*XX.*YY.*ZZ+(-3).*WW.^3.*XX.*YY.*ZZ+(-4).*phi.* ...

WW.^3.*XX.*YY.*ZZ+(-1).*phi.^2.*WW.^3.*XX.*YY.*ZZ)+2.*(3.*WW.^2+(-4).* ...

phi.*WW.^2+phi.^2.*WW.^2+3.*WW.^3+2.*phi.*WW.^3+(-2).*phi.^2.*WW.^3+2.* ...

phi.*WW.^4+phi.^2.*WW.^4+(-3).*WW.*XX+4.*phi.*WW.*XX+(-1).*phi.^2.*WW.* ...

XX+(-3).*WW.^2.*XX+(-2).*phi.*WW.^2.*XX+2.*phi.^2.*WW.^2.*XX+(-2).*phi.* ...

WW.^3.*XX+(-1).*phi.^2.*WW.^3.*XX+(-1).*phi.*WW.*YY+phi.^2.*WW.*YY+ ...

WW.^2.*YY+2.*phi.*WW.^2.*YY+(-4).*phi.^2.*WW.^2.*YY+(-5).*WW.^3.*YY+5.* ...

phi.*WW.^3.*YY+5.*phi.^2.*WW.^3.*YY+(-2).*WW.^4.*YY+(-6).*phi.*WW.^4.* ...

YY+(-2).*phi.^2.*WW.^4.*YY+3.*WW.^2.*XX.*YY+(-5).*phi.*WW.^2.*XX.*YY+ ...

phi.^2.*WW.^2.*XX.*YY+3.*WW.^3.*XX.*YY+4.*phi.*WW.^3.*XX.*YY+(-2).* ...

phi.^2.*WW.^3.*XX.*YY+phi.*WW.^4.*XX.*YY+phi.^2.*WW.^4.*XX.*YY+phi.*WW.* ...

ZZ+(-1).*phi.^2.*WW.*ZZ+(-3).*WW.^2.*ZZ+4.*phi.*WW.^2.*ZZ+2.*phi.^2.* ...

WW.^2.*ZZ+(-3).*WW.^3.*ZZ+(-5).*phi.*WW.^3.*ZZ+(-1).*phi.^2.*WW.^3.*ZZ+ ...

2.*WW.*XX.*ZZ+(-6).*phi.*WW.*XX.*ZZ+2.*phi.^2.*WW.*XX.*ZZ+5.*WW.^2.*XX.* ...

ZZ+5.*phi.*WW.^2.*XX.*ZZ+(-5).*phi.^2.*WW.^2.*XX.*ZZ+(-1).*WW.^3.*XX.* ...

ZZ+2.*phi.*WW.^3.*XX.*ZZ+4.*phi.^2.*WW.^3.*XX.*ZZ+(-1).*phi.*WW.^4.*XX.* ...

ZZ+(-1).*phi.^2.*WW.^4.*XX.*ZZ+(-2).*phi.*WW.^2.*YY.*ZZ+phi.^2.*WW.^2.* ...

YY.*ZZ+3.*WW.^3.*YY.*ZZ+(-2).*phi.*WW.^3.*YY.*ZZ+(-2).*phi.^2.*WW.^3.* ...

YY.*ZZ+3.*WW.^4.*YY.*ZZ+4.*phi.*WW.^4.*YY.*ZZ+phi.^2.*WW.^4.*YY.*ZZ+2.* ...

phi.*WW.*XX.*YY.*ZZ+(-1).*phi.^2.*WW.*XX.*YY.*ZZ+(-3).*WW.^2.*XX.*YY.* ...

ZZ+2.*phi.*WW.^2.*XX.*YY.*ZZ+2.*phi.^2.*WW.^2.*XX.*YY.*ZZ+(-3).*WW.^3.* ...

XX.*YY.*ZZ+(-4).*phi.*WW.^3.*XX.*YY.*ZZ+(-1).*phi.^2.*WW.^3.*XX.*YY.*ZZ) ...

.^3+(-9).*((-3).*WW.^3+2.*phi.*WW.^3+(-1).*WW.^4+(-2).*phi.*WW.^4+3.* ...

WW.^2.*XX+(-2).*phi.*WW.^2.*XX+WW.^3.*XX+2.*phi.*WW.^3.*XX+(-1).*WW.^2.* ...

YY+2.*phi.*WW.^2.*YY+WW.^3.*YY+(-6).*phi.*WW.^3.*YY+4.*WW.^4.*YY+4.* ...

phi.*WW.^4.*YY+(-3).*WW.^3.*XX.*YY+2.*phi.*WW.^3.*XX.*YY+(-1).*WW.^4.* ...

XX.*YY+(-2).*phi.*WW.^4.*XX.*YY+WW.^2.*ZZ+(-2).*phi.*WW.^2.*ZZ+3.* ...

WW.^3.*ZZ+2.*phi.*WW.^3.*ZZ+(-4).*WW.^2.*XX.*ZZ+4.*phi.*WW.^2.*XX.*ZZ+( ...

-1).*WW.^3.*XX.*ZZ+(-6).*phi.*WW.^3.*XX.*ZZ+WW.^4.*XX.*ZZ+2.*phi.* ...

WW.^4.*XX.*ZZ+(-1).*WW.^3.*YY.*ZZ+2.*phi.*WW.^3.*YY.*ZZ+(-3).*WW.^4.* ...

YY.*ZZ+(-2).*phi.*WW.^4.*YY.*ZZ+WW.^2.*XX.*YY.*ZZ+(-2).*phi.*WW.^2.*XX.* ...

YY.*ZZ+3.*WW.^3.*XX.*YY.*ZZ+2.*phi.*WW.^3.*XX.*YY.*ZZ).*(3.*WW.^2+(-4).* ...

phi.*WW.^2+phi.^2.*WW.^2+3.*WW.^3+2.*phi.*WW.^3+(-2).*phi.^2.*WW.^3+2.* ...

phi.*WW.^4+phi.^2.*WW.^4+(-3).*WW.*XX+4.*phi.*WW.*XX+(-1).*phi.^2.*WW.* ...

XX+(-3).*WW.^2.*XX+(-2).*phi.*WW.^2.*XX+2.*phi.^2.*WW.^2.*XX+(-2).*phi.* ...

WW.^3.*XX+(-1).*phi.^2.*WW.^3.*XX+(-1).*phi.*WW.*YY+phi.^2.*WW.*YY+ ...

WW.^2.*YY+2.*phi.*WW.^2.*YY+(-4).*phi.^2.*WW.^2.*YY+(-5).*WW.^3.*YY+5.* ...

phi.*WW.^3.*YY+5.*phi.^2.*WW.^3.*YY+(-2).*WW.^4.*YY+(-6).*phi.*WW.^4.* ...

YY+(-2).*phi.^2.*WW.^4.*YY+3.*WW.^2.*XX.*YY+(-5).*phi.*WW.^2.*XX.*YY+ ...

phi.^2.*WW.^2.*XX.*YY+3.*WW.^3.*XX.*YY+4.*phi.*WW.^3.*XX.*YY+(-2).* ...

phi.^2.*WW.^3.*XX.*YY+phi.*WW.^4.*XX.*YY+phi.^2.*WW.^4.*XX.*YY+phi.*WW.* ...

ZZ+(-1).*phi.^2.*WW.*ZZ+(-3).*WW.^2.*ZZ+4.*phi.*WW.^2.*ZZ+2.*phi.^2.* ...

WW.^2.*ZZ+(-3).*WW.^3.*ZZ+(-5).*phi.*WW.^3.*ZZ+(-1).*phi.^2.*WW.^3.*ZZ+ ...

2.*WW.*XX.*ZZ+(-6).*phi.*WW.*XX.*ZZ+2.*phi.^2.*WW.*XX.*ZZ+5.*WW.^2.*XX.* ...

ZZ+5.*phi.*WW.^2.*XX.*ZZ+(-5).*phi.^2.*WW.^2.*XX.*ZZ+(-1).*WW.^3.*XX.* ...

ZZ+2.*phi.*WW.^3.*XX.*ZZ+4.*phi.^2.*WW.^3.*XX.*ZZ+(-1).*phi.*WW.^4.*XX.* ...

ZZ+(-1).*phi.^2.*WW.^4.*XX.*ZZ+(-2).*phi.*WW.^2.*YY.*ZZ+phi.^2.*WW.^2.* ...

YY.*ZZ+3.*WW.^3.*YY.*ZZ+(-2).*phi.*WW.^3.*YY.*ZZ+(-2).*phi.^2.*WW.^3.* ...

YY.*ZZ+3.*WW.^4.*YY.*ZZ+4.*phi.*WW.^4.*YY.*ZZ+phi.^2.*WW.^4.*YY.*ZZ+2.* ...

phi.*WW.*XX.*YY.*ZZ+(-1).*phi.^2.*WW.*XX.*YY.*ZZ+(-3).*WW.^2.*XX.*YY.* ...

ZZ+2.*phi.*WW.^2.*XX.*YY.*ZZ+2.*phi.^2.*WW.^2.*XX.*YY.*ZZ+(-3).*WW.^3.* ...

XX.*YY.*ZZ+(-4).*phi.*WW.^3.*XX.*YY.*ZZ+(-1).*phi.^2.*WW.^3.*XX.*YY.*ZZ) ...

.*((-1).*WW+2.*phi.*WW+(-1).*phi.^2.*WW+(-3).*WW.^2+2.*phi.*WW.^2+ ...

phi.^2.*WW.^2+(-4).*phi.*WW.^3+phi.^2.*WW.^3+(-1).*phi.^2.*WW.^4+XX+(-2) ...

.*phi.*XX+phi.^2.*XX+3.*WW.*XX+(-2).*phi.*WW.*XX+(-1).*phi.^2.*WW.*XX+ ...

4.*phi.*WW.^2.*XX+(-1).*phi.^2.*WW.^2.*XX+phi.^2.*WW.^3.*XX+WW.^2.*YY+( ...

-4).*phi.*WW.^2.*YY+2.*phi.^2.*WW.^2.*YY+3.*WW.^3.*YY+2.*phi.*WW.^3.*YY+ ...

(-4).*phi.^2.*WW.^3.*YY+2.*phi.*WW.^4.*YY+2.*phi.^2.*WW.^4.*YY+(-1).* ...

WW.*XX.*YY+4.*phi.*WW.*XX.*YY+(-2).*phi.^2.*WW.*XX.*YY+(-3).*WW.^2.*XX.* ...

YY+(-2).*phi.*WW.^2.*XX.*YY+4.*phi.^2.*WW.^2.*XX.*YY+(-2).*phi.*WW.^3.* ...

XX.*YY+(-2).*phi.^2.*WW.^3.*XX.*YY+(-2).*phi.*WW.*ZZ+2.*phi.^2.*WW.*ZZ+ ...

3.*WW.^2.*ZZ+(-2).*phi.*WW.^2.*ZZ+(-4).*phi.^2.*WW.^2.*ZZ+WW.^3.*ZZ+4.* ...

phi.*WW.^3.*ZZ+2.*phi.^2.*WW.^3.*ZZ+2.*phi.*XX.*ZZ+(-2).*phi.^2.*XX.*ZZ+ ...

(-3).*WW.*XX.*ZZ+2.*phi.*WW.*XX.*ZZ+4.*phi.^2.*WW.*XX.*ZZ+(-1).*WW.^2.* ...

XX.*ZZ+(-4).*phi.*WW.^2.*XX.*ZZ+(-2).*phi.^2.*WW.^2.*XX.*ZZ+(-1).* ...

phi.^2.*WW.*YY.*ZZ+4.*phi.*WW.^2.*YY.*ZZ+phi.^2.*WW.^2.*YY.*ZZ+(-3).* ...

WW.^3.*YY.*ZZ+(-2).*phi.*WW.^3.*YY.*ZZ+phi.^2.*WW.^3.*YY.*ZZ+(-1).* ...

WW.^4.*YY.*ZZ+(-2).*phi.*WW.^4.*YY.*ZZ+(-1).*phi.^2.*WW.^4.*YY.*ZZ+ ...

phi.^2.*XX.*YY.*ZZ+(-4).*phi.*WW.*XX.*YY.*ZZ+(-1).*phi.^2.*WW.*XX.*YY.* ...

ZZ+3.*WW.^2.*XX.*YY.*ZZ+2.*phi.*WW.^2.*XX.*YY.*ZZ+(-1).*phi.^2.*WW.^2.* ...

XX.*YY.*ZZ+WW.^3.*XX.*YY.*ZZ+2.*phi.*WW.^3.*XX.*YY.*ZZ+phi.^2.*WW.^3.* ...

XX.*YY.*ZZ)+27.*(WW.^4+(-1).*WW.^3.*XX+WW.^3.*YY+(-2).*WW.^4.*YY+WW.^4.* ...

XX.*YY+(-1).*WW.^3.*ZZ+2.*WW.^3.*XX.*ZZ+(-1).*WW.^4.*XX.*ZZ+WW.^4.*YY.* ...

ZZ+(-1).*WW.^3.*XX.*YY.*ZZ).*((-1).*WW+2.*phi.*WW+(-1).*phi.^2.*WW+(-3) ...

.*WW.^2+2.*phi.*WW.^2+phi.^2.*WW.^2+(-4).*phi.*WW.^3+phi.^2.*WW.^3+(-1) ...

.*phi.^2.*WW.^4+XX+(-2).*phi.*XX+phi.^2.*XX+3.*WW.*XX+(-2).*phi.*WW.*XX+ ...

(-1).*phi.^2.*WW.*XX+4.*phi.*WW.^2.*XX+(-1).*phi.^2.*WW.^2.*XX+phi.^2.* ...

WW.^3.*XX+WW.^2.*YY+(-4).*phi.*WW.^2.*YY+2.*phi.^2.*WW.^2.*YY+3.*WW.^3.* ...

YY+2.*phi.*WW.^3.*YY+(-4).*phi.^2.*WW.^3.*YY+2.*phi.*WW.^4.*YY+2.* ...

phi.^2.*WW.^4.*YY+(-1).*WW.*XX.*YY+4.*phi.*WW.*XX.*YY+(-2).*phi.^2.*WW.* ...

XX.*YY+(-3).*WW.^2.*XX.*YY+(-2).*phi.*WW.^2.*XX.*YY+4.*phi.^2.*WW.^2.* ...

XX.*YY+(-2).*phi.*WW.^3.*XX.*YY+(-2).*phi.^2.*WW.^3.*XX.*YY+(-2).*phi.* ...

WW.*ZZ+2.*phi.^2.*WW.*ZZ+3.*WW.^2.*ZZ+(-2).*phi.*WW.^2.*ZZ+(-4).* ...

phi.^2.*WW.^2.*ZZ+WW.^3.*ZZ+4.*phi.*WW.^3.*ZZ+2.*phi.^2.*WW.^3.*ZZ+2.* ...

phi.*XX.*ZZ+(-2).*phi.^2.*XX.*ZZ+(-3).*WW.*XX.*ZZ+2.*phi.*WW.*XX.*ZZ+4.* ...

phi.^2.*WW.*XX.*ZZ+(-1).*WW.^2.*XX.*ZZ+(-4).*phi.*WW.^2.*XX.*ZZ+(-2).* ...

phi.^2.*WW.^2.*XX.*ZZ+(-1).*phi.^2.*WW.*YY.*ZZ+4.*phi.*WW.^2.*YY.*ZZ+ ...

phi.^2.*WW.^2.*YY.*ZZ+(-3).*WW.^3.*YY.*ZZ+(-2).*phi.*WW.^3.*YY.*ZZ+ ...

phi.^2.*WW.^3.*YY.*ZZ+(-1).*WW.^4.*YY.*ZZ+(-2).*phi.*WW.^4.*YY.*ZZ+(-1) ...

.*phi.^2.*WW.^4.*YY.*ZZ+phi.^2.*XX.*YY.*ZZ+(-4).*phi.*WW.*XX.*YY.*ZZ+( ...

-1).*phi.^2.*WW.*XX.*YY.*ZZ+3.*WW.^2.*XX.*YY.*ZZ+2.*phi.*WW.^2.*XX.*YY.* ...

ZZ+(-1).*phi.^2.*WW.^2.*XX.*YY.*ZZ+WW.^3.*XX.*YY.*ZZ+2.*phi.*WW.^3.*XX.* ...

YY.*ZZ+phi.^2.*WW.^3.*XX.*YY.*ZZ).^2).^2).^(1/2)).^(-1/3)+(1/3).*2.^( ...

-1/3).*(WW+(-2).*phi.*WW+phi.^2.*WW+2.*phi.*WW.^2+(-2).*phi.^2.*WW.^2+ ...

phi.^2.*WW.^3+(-1).*XX+2.*phi.*XX+(-1).*phi.^2.*XX+(-2).*phi.*WW.*XX+2.* ...

phi.^2.*WW.*XX+(-1).*phi.^2.*WW.^2.*XX+phi.*WW.*YY+(-1).*phi.^2.*WW.*YY+ ...

(-1).*WW.^2.*YY+2.*phi.^2.*WW.^2.*YY+(-1).*phi.*WW.^3.*YY+(-1).*phi.^2.* ...

WW.^3.*YY+(-1).*phi.*XX.*YY+phi.^2.*XX.*YY+WW.*XX.*YY+(-2).*phi.^2.*WW.* ...

XX.*YY+phi.*WW.^2.*XX.*YY+phi.^2.*WW.^2.*XX.*YY+phi.*WW.*ZZ+(-1).* ...

phi.^2.*WW.*ZZ+(-1).*WW.^2.*ZZ+2.*phi.^2.*WW.^2.*ZZ+(-1).*phi.*WW.^3.* ...

ZZ+(-1).*phi.^2.*WW.^3.*ZZ+(-1).*phi.*XX.*ZZ+phi.^2.*XX.*ZZ+WW.*XX.*ZZ+( ...

-2).*phi.^2.*WW.*XX.*ZZ+phi.*WW.^2.*XX.*ZZ+phi.^2.*WW.^2.*XX.*ZZ+ ...

phi.^2.*WW.*YY.*ZZ+(-2).*phi.*WW.^2.*YY.*ZZ+(-2).*phi.^2.*WW.^2.*YY.*ZZ+ ...

WW.^3.*YY.*ZZ+2.*phi.*WW.^3.*YY.*ZZ+phi.^2.*WW.^3.*YY.*ZZ+(-1).*phi.^2.* ...

XX.*YY.*ZZ+2.*phi.*WW.*XX.*YY.*ZZ+2.*phi.^2.*WW.*XX.*YY.*ZZ+(-1).* ...

WW.^2.*XX.*YY.*ZZ+(-2).*phi.*WW.^2.*XX.*YY.*ZZ+(-1).*phi.^2.*WW.^2.*XX.* ...

YY.*ZZ).^(-1).*(27.*(WW+(-2).*phi.*WW+phi.^2.*WW+2.*phi.*WW.^2+(-2).* ...

phi.^2.*WW.^2+phi.^2.*WW.^3+(-1).*XX+2.*phi.*XX+(-1).*phi.^2.*XX+(-2).* ...

phi.*WW.*XX+2.*phi.^2.*WW.*XX+(-1).*phi.^2.*WW.^2.*XX+phi.*WW.*YY+(-1).* ...

phi.^2.*WW.*YY+(-1).*WW.^2.*YY+2.*phi.^2.*WW.^2.*YY+(-1).*phi.*WW.^3.* ...

YY+(-1).*phi.^2.*WW.^3.*YY+(-1).*phi.*XX.*YY+phi.^2.*XX.*YY+WW.*XX.*YY+( ...

-2).*phi.^2.*WW.*XX.*YY+phi.*WW.^2.*XX.*YY+phi.^2.*WW.^2.*XX.*YY+phi.* ...

WW.*ZZ+(-1).*phi.^2.*WW.*ZZ+(-1).*WW.^2.*ZZ+2.*phi.^2.*WW.^2.*ZZ+(-1).* ...

phi.*WW.^3.*ZZ+(-1).*phi.^2.*WW.^3.*ZZ+(-1).*phi.*XX.*ZZ+phi.^2.*XX.*ZZ+ ...

WW.*XX.*ZZ+(-2).*phi.^2.*WW.*XX.*ZZ+phi.*WW.^2.*XX.*ZZ+phi.^2.*WW.^2.* ...

XX.*ZZ+phi.^2.*WW.*YY.*ZZ+(-2).*phi.*WW.^2.*YY.*ZZ+(-2).*phi.^2.*WW.^2.* ...

YY.*ZZ+WW.^3.*YY.*ZZ+2.*phi.*WW.^3.*YY.*ZZ+phi.^2.*WW.^3.*YY.*ZZ+(-1).* ...

phi.^2.*XX.*YY.*ZZ+2.*phi.*WW.*XX.*YY.*ZZ+2.*phi.^2.*WW.*XX.*YY.*ZZ+(-1) ...

.*WW.^2.*XX.*YY.*ZZ+(-2).*phi.*WW.^2.*XX.*YY.*ZZ+(-1).*phi.^2.*WW.^2.* ...

XX.*YY.*ZZ).*((-3).*WW.^3+2.*phi.*WW.^3+(-1).*WW.^4+(-2).*phi.*WW.^4+3.* ...

WW.^2.*XX+(-2).*phi.*WW.^2.*XX+WW.^3.*XX+2.*phi.*WW.^3.*XX+(-1).*WW.^2.* ...

YY+2.*phi.*WW.^2.*YY+WW.^3.*YY+(-6).*phi.*WW.^3.*YY+4.*WW.^4.*YY+4.* ...

phi.*WW.^4.*YY+(-3).*WW.^3.*XX.*YY+2.*phi.*WW.^3.*XX.*YY+(-1).*WW.^4.* ...

XX.*YY+(-2).*phi.*WW.^4.*XX.*YY+WW.^2.*ZZ+(-2).*phi.*WW.^2.*ZZ+3.* ...

WW.^3.*ZZ+2.*phi.*WW.^3.*ZZ+(-4).*WW.^2.*XX.*ZZ+4.*phi.*WW.^2.*XX.*ZZ+( ...

-1).*WW.^3.*XX.*ZZ+(-6).*phi.*WW.^3.*XX.*ZZ+WW.^4.*XX.*ZZ+2.*phi.* ...

WW.^4.*XX.*ZZ+(-1).*WW.^3.*YY.*ZZ+2.*phi.*WW.^3.*YY.*ZZ+(-3).*WW.^4.* ...

YY.*ZZ+(-2).*phi.*WW.^4.*YY.*ZZ+WW.^2.*XX.*YY.*ZZ+(-2).*phi.*WW.^2.*XX.* ...

YY.*ZZ+3.*WW.^3.*XX.*YY.*ZZ+2.*phi.*WW.^3.*XX.*YY.*ZZ).^2+(-72).*(WW+( ...

-2).*phi.*WW+phi.^2.*WW+2.*phi.*WW.^2+(-2).*phi.^2.*WW.^2+phi.^2.*WW.^3+ ...

(-1).*XX+2.*phi.*XX+(-1).*phi.^2.*XX+(-2).*phi.*WW.*XX+2.*phi.^2.*WW.* ...

XX+(-1).*phi.^2.*WW.^2.*XX+phi.*WW.*YY+(-1).*phi.^2.*WW.*YY+(-1).* ...

WW.^2.*YY+2.*phi.^2.*WW.^2.*YY+(-1).*phi.*WW.^3.*YY+(-1).*phi.^2.* ...

WW.^3.*YY+(-1).*phi.*XX.*YY+phi.^2.*XX.*YY+WW.*XX.*YY+(-2).*phi.^2.*WW.* ...

XX.*YY+phi.*WW.^2.*XX.*YY+phi.^2.*WW.^2.*XX.*YY+phi.*WW.*ZZ+(-1).* ...

phi.^2.*WW.*ZZ+(-1).*WW.^2.*ZZ+2.*phi.^2.*WW.^2.*ZZ+(-1).*phi.*WW.^3.* ...

ZZ+(-1).*phi.^2.*WW.^3.*ZZ+(-1).*phi.*XX.*ZZ+phi.^2.*XX.*ZZ+WW.*XX.*ZZ+( ...

-2).*phi.^2.*WW.*XX.*ZZ+phi.*WW.^2.*XX.*ZZ+phi.^2.*WW.^2.*XX.*ZZ+ ...

phi.^2.*WW.*YY.*ZZ+(-2).*phi.*WW.^2.*YY.*ZZ+(-2).*phi.^2.*WW.^2.*YY.*ZZ+ ...

WW.^3.*YY.*ZZ+2.*phi.*WW.^3.*YY.*ZZ+phi.^2.*WW.^3.*YY.*ZZ+(-1).*phi.^2.* ...

XX.*YY.*ZZ+2.*phi.*WW.*XX.*YY.*ZZ+2.*phi.^2.*WW.*XX.*YY.*ZZ+(-1).* ...

WW.^2.*XX.*YY.*ZZ+(-2).*phi.*WW.^2.*XX.*YY.*ZZ+(-1).*phi.^2.*WW.^2.*XX.* ...

YY.*ZZ).*(WW.^4+(-1).*WW.^3.*XX+WW.^3.*YY+(-2).*WW.^4.*YY+WW.^4.*XX.*YY+ ...

(-1).*WW.^3.*ZZ+2.*WW.^3.*XX.*ZZ+(-1).*WW.^4.*XX.*ZZ+WW.^4.*YY.*ZZ+(-1) ...

.*WW.^3.*XX.*YY.*ZZ).*(3.*WW.^2+(-4).*phi.*WW.^2+phi.^2.*WW.^2+3.*WW.^3+ ...

2.*phi.*WW.^3+(-2).*phi.^2.*WW.^3+2.*phi.*WW.^4+phi.^2.*WW.^4+(-3).*WW.* ...

XX+4.*phi.*WW.*XX+(-1).*phi.^2.*WW.*XX+(-3).*WW.^2.*XX+(-2).*phi.* ...

WW.^2.*XX+2.*phi.^2.*WW.^2.*XX+(-2).*phi.*WW.^3.*XX+(-1).*phi.^2.* ...

WW.^3.*XX+(-1).*phi.*WW.*YY+phi.^2.*WW.*YY+WW.^2.*YY+2.*phi.*WW.^2.*YY+( ...

-4).*phi.^2.*WW.^2.*YY+(-5).*WW.^3.*YY+5.*phi.*WW.^3.*YY+5.*phi.^2.* ...

WW.^3.*YY+(-2).*WW.^4.*YY+(-6).*phi.*WW.^4.*YY+(-2).*phi.^2.*WW.^4.*YY+ ...

3.*WW.^2.*XX.*YY+(-5).*phi.*WW.^2.*XX.*YY+phi.^2.*WW.^2.*XX.*YY+3.* ...

WW.^3.*XX.*YY+4.*phi.*WW.^3.*XX.*YY+(-2).*phi.^2.*WW.^3.*XX.*YY+phi.* ...

WW.^4.*XX.*YY+phi.^2.*WW.^4.*XX.*YY+phi.*WW.*ZZ+(-1).*phi.^2.*WW.*ZZ+( ...

-3).*WW.^2.*ZZ+4.*phi.*WW.^2.*ZZ+2.*phi.^2.*WW.^2.*ZZ+(-3).*WW.^3.*ZZ+( ...

-5).*phi.*WW.^3.*ZZ+(-1).*phi.^2.*WW.^3.*ZZ+2.*WW.*XX.*ZZ+(-6).*phi.* ...

WW.*XX.*ZZ+2.*phi.^2.*WW.*XX.*ZZ+5.*WW.^2.*XX.*ZZ+5.*phi.*WW.^2.*XX.*ZZ+ ...

(-5).*phi.^2.*WW.^2.*XX.*ZZ+(-1).*WW.^3.*XX.*ZZ+2.*phi.*WW.^3.*XX.*ZZ+ ...

4.*phi.^2.*WW.^3.*XX.*ZZ+(-1).*phi.*WW.^4.*XX.*ZZ+(-1).*phi.^2.*WW.^4.* ...

XX.*ZZ+(-2).*phi.*WW.^2.*YY.*ZZ+phi.^2.*WW.^2.*YY.*ZZ+3.*WW.^3.*YY.*ZZ+( ...

-2).*phi.*WW.^3.*YY.*ZZ+(-2).*phi.^2.*WW.^3.*YY.*ZZ+3.*WW.^4.*YY.*ZZ+4.* ...

phi.*WW.^4.*YY.*ZZ+phi.^2.*WW.^4.*YY.*ZZ+2.*phi.*WW.*XX.*YY.*ZZ+(-1).* ...

phi.^2.*WW.*XX.*YY.*ZZ+(-3).*WW.^2.*XX.*YY.*ZZ+2.*phi.*WW.^2.*XX.*YY.* ...

ZZ+2.*phi.^2.*WW.^2.*XX.*YY.*ZZ+(-3).*WW.^3.*XX.*YY.*ZZ+(-4).*phi.* ...

WW.^3.*XX.*YY.*ZZ+(-1).*phi.^2.*WW.^3.*XX.*YY.*ZZ)+2.*(3.*WW.^2+(-4).* ...

phi.*WW.^2+phi.^2.*WW.^2+3.*WW.^3+2.*phi.*WW.^3+(-2).*phi.^2.*WW.^3+2.* ...

phi.*WW.^4+phi.^2.*WW.^4+(-3).*WW.*XX+4.*phi.*WW.*XX+(-1).*phi.^2.*WW.* ...

XX+(-3).*WW.^2.*XX+(-2).*phi.*WW.^2.*XX+2.*phi.^2.*WW.^2.*XX+(-2).*phi.* ...

WW.^3.*XX+(-1).*phi.^2.*WW.^3.*XX+(-1).*phi.*WW.*YY+phi.^2.*WW.*YY+ ...

WW.^2.*YY+2.*phi.*WW.^2.*YY+(-4).*phi.^2.*WW.^2.*YY+(-5).*WW.^3.*YY+5.* ...

phi.*WW.^3.*YY+5.*phi.^2.*WW.^3.*YY+(-2).*WW.^4.*YY+(-6).*phi.*WW.^4.* ...

YY+(-2).*phi.^2.*WW.^4.*YY+3.*WW.^2.*XX.*YY+(-5).*phi.*WW.^2.*XX.*YY+ ...

phi.^2.*WW.^2.*XX.*YY+3.*WW.^3.*XX.*YY+4.*phi.*WW.^3.*XX.*YY+(-2).* ...

phi.^2.*WW.^3.*XX.*YY+phi.*WW.^4.*XX.*YY+phi.^2.*WW.^4.*XX.*YY+phi.*WW.* ...

ZZ+(-1).*phi.^2.*WW.*ZZ+(-3).*WW.^2.*ZZ+4.*phi.*WW.^2.*ZZ+2.*phi.^2.* ...

WW.^2.*ZZ+(-3).*WW.^3.*ZZ+(-5).*phi.*WW.^3.*ZZ+(-1).*phi.^2.*WW.^3.*ZZ+ ...

2.*WW.*XX.*ZZ+(-6).*phi.*WW.*XX.*ZZ+2.*phi.^2.*WW.*XX.*ZZ+5.*WW.^2.*XX.* ...

ZZ+5.*phi.*WW.^2.*XX.*ZZ+(-5).*phi.^2.*WW.^2.*XX.*ZZ+(-1).*WW.^3.*XX.* ...

ZZ+2.*phi.*WW.^3.*XX.*ZZ+4.*phi.^2.*WW.^3.*XX.*ZZ+(-1).*phi.*WW.^4.*XX.* ...

ZZ+(-1).*phi.^2.*WW.^4.*XX.*ZZ+(-2).*phi.*WW.^2.*YY.*ZZ+phi.^2.*WW.^2.* ...

YY.*ZZ+3.*WW.^3.*YY.*ZZ+(-2).*phi.*WW.^3.*YY.*ZZ+(-2).*phi.^2.*WW.^3.* ...

YY.*ZZ+3.*WW.^4.*YY.*ZZ+4.*phi.*WW.^4.*YY.*ZZ+phi.^2.*WW.^4.*YY.*ZZ+2.* ...

phi.*WW.*XX.*YY.*ZZ+(-1).*phi.^2.*WW.*XX.*YY.*ZZ+(-3).*WW.^2.*XX.*YY.* ...

ZZ+2.*phi.*WW.^2.*XX.*YY.*ZZ+2.*phi.^2.*WW.^2.*XX.*YY.*ZZ+(-3).*WW.^3.* ...

XX.*YY.*ZZ+(-4).*phi.*WW.^3.*XX.*YY.*ZZ+(-1).*phi.^2.*WW.^3.*XX.*YY.*ZZ) ...

.^3+(-9).*((-3).*WW.^3+2.*phi.*WW.^3+(-1).*WW.^4+(-2).*phi.*WW.^4+3.* ...

WW.^2.*XX+(-2).*phi.*WW.^2.*XX+WW.^3.*XX+2.*phi.*WW.^3.*XX+(-1).*WW.^2.* ...

YY+2.*phi.*WW.^2.*YY+WW.^3.*YY+(-6).*phi.*WW.^3.*YY+4.*WW.^4.*YY+4.* ...

phi.*WW.^4.*YY+(-3).*WW.^3.*XX.*YY+2.*phi.*WW.^3.*XX.*YY+(-1).*WW.^4.* ...

XX.*YY+(-2).*phi.*WW.^4.*XX.*YY+WW.^2.*ZZ+(-2).*phi.*WW.^2.*ZZ+3.* ...

WW.^3.*ZZ+2.*phi.*WW.^3.*ZZ+(-4).*WW.^2.*XX.*ZZ+4.*phi.*WW.^2.*XX.*ZZ+( ...

-1).*WW.^3.*XX.*ZZ+(-6).*phi.*WW.^3.*XX.*ZZ+WW.^4.*XX.*ZZ+2.*phi.* ...

WW.^4.*XX.*ZZ+(-1).*WW.^3.*YY.*ZZ+2.*phi.*WW.^3.*YY.*ZZ+(-3).*WW.^4.* ...

YY.*ZZ+(-2).*phi.*WW.^4.*YY.*ZZ+WW.^2.*XX.*YY.*ZZ+(-2).*phi.*WW.^2.*XX.* ...

YY.*ZZ+3.*WW.^3.*XX.*YY.*ZZ+2.*phi.*WW.^3.*XX.*YY.*ZZ).*(3.*WW.^2+(-4).* ...

phi.*WW.^2+phi.^2.*WW.^2+3.*WW.^3+2.*phi.*WW.^3+(-2).*phi.^2.*WW.^3+2.* ...

phi.*WW.^4+phi.^2.*WW.^4+(-3).*WW.*XX+4.*phi.*WW.*XX+(-1).*phi.^2.*WW.* ...

XX+(-3).*WW.^2.*XX+(-2).*phi.*WW.^2.*XX+2.*phi.^2.*WW.^2.*XX+(-2).*phi.* ...

WW.^3.*XX+(-1).*phi.^2.*WW.^3.*XX+(-1).*phi.*WW.*YY+phi.^2.*WW.*YY+ ...

WW.^2.*YY+2.*phi.*WW.^2.*YY+(-4).*phi.^2.*WW.^2.*YY+(-5).*WW.^3.*YY+5.* ...

phi.*WW.^3.*YY+5.*phi.^2.*WW.^3.*YY+(-2).*WW.^4.*YY+(-6).*phi.*WW.^4.* ...

YY+(-2).*phi.^2.*WW.^4.*YY+3.*WW.^2.*XX.*YY+(-5).*phi.*WW.^2.*XX.*YY+ ...

phi.^2.*WW.^2.*XX.*YY+3.*WW.^3.*XX.*YY+4.*phi.*WW.^3.*XX.*YY+(-2).* ...

phi.^2.*WW.^3.*XX.*YY+phi.*WW.^4.*XX.*YY+phi.^2.*WW.^4.*XX.*YY+phi.*WW.* ...

ZZ+(-1).*phi.^2.*WW.*ZZ+(-3).*WW.^2.*ZZ+4.*phi.*WW.^2.*ZZ+2.*phi.^2.* ...

WW.^2.*ZZ+(-3).*WW.^3.*ZZ+(-5).*phi.*WW.^3.*ZZ+(-1).*phi.^2.*WW.^3.*ZZ+ ...

2.*WW.*XX.*ZZ+(-6).*phi.*WW.*XX.*ZZ+2.*phi.^2.*WW.*XX.*ZZ+5.*WW.^2.*XX.* ...

ZZ+5.*phi.*WW.^2.*XX.*ZZ+(-5).*phi.^2.*WW.^2.*XX.*ZZ+(-1).*WW.^3.*XX.* ...

ZZ+2.*phi.*WW.^3.*XX.*ZZ+4.*phi.^2.*WW.^3.*XX.*ZZ+(-1).*phi.*WW.^4.*XX.* ...

ZZ+(-1).*phi.^2.*WW.^4.*XX.*ZZ+(-2).*phi.*WW.^2.*YY.*ZZ+phi.^2.*WW.^2.* ...

YY.*ZZ+3.*WW.^3.*YY.*ZZ+(-2).*phi.*WW.^3.*YY.*ZZ+(-2).*phi.^2.*WW.^3.* ...

YY.*ZZ+3.*WW.^4.*YY.*ZZ+4.*phi.*WW.^4.*YY.*ZZ+phi.^2.*WW.^4.*YY.*ZZ+2.* ...

phi.*WW.*XX.*YY.*ZZ+(-1).*phi.^2.*WW.*XX.*YY.*ZZ+(-3).*WW.^2.*XX.*YY.* ...

ZZ+2.*phi.*WW.^2.*XX.*YY.*ZZ+2.*phi.^2.*WW.^2.*XX.*YY.*ZZ+(-3).*WW.^3.* ...

XX.*YY.*ZZ+(-4).*phi.*WW.^3.*XX.*YY.*ZZ+(-1).*phi.^2.*WW.^3.*XX.*YY.*ZZ) ...

.*((-1).*WW+2.*phi.*WW+(-1).*phi.^2.*WW+(-3).*WW.^2+2.*phi.*WW.^2+ ...

phi.^2.*WW.^2+(-4).*phi.*WW.^3+phi.^2.*WW.^3+(-1).*phi.^2.*WW.^4+XX+(-2) ...

.*phi.*XX+phi.^2.*XX+3.*WW.*XX+(-2).*phi.*WW.*XX+(-1).*phi.^2.*WW.*XX+ ...

4.*phi.*WW.^2.*XX+(-1).*phi.^2.*WW.^2.*XX+phi.^2.*WW.^3.*XX+WW.^2.*YY+( ...

-4).*phi.*WW.^2.*YY+2.*phi.^2.*WW.^2.*YY+3.*WW.^3.*YY+2.*phi.*WW.^3.*YY+ ...

(-4).*phi.^2.*WW.^3.*YY+2.*phi.*WW.^4.*YY+2.*phi.^2.*WW.^4.*YY+(-1).* ...

WW.*XX.*YY+4.*phi.*WW.*XX.*YY+(-2).*phi.^2.*WW.*XX.*YY+(-3).*WW.^2.*XX.* ...

YY+(-2).*phi.*WW.^2.*XX.*YY+4.*phi.^2.*WW.^2.*XX.*YY+(-2).*phi.*WW.^3.* ...

XX.*YY+(-2).*phi.^2.*WW.^3.*XX.*YY+(-2).*phi.*WW.*ZZ+2.*phi.^2.*WW.*ZZ+ ...

3.*WW.^2.*ZZ+(-2).*phi.*WW.^2.*ZZ+(-4).*phi.^2.*WW.^2.*ZZ+WW.^3.*ZZ+4.* ...

phi.*WW.^3.*ZZ+2.*phi.^2.*WW.^3.*ZZ+2.*phi.*XX.*ZZ+(-2).*phi.^2.*XX.*ZZ+ ...

(-3).*WW.*XX.*ZZ+2.*phi.*WW.*XX.*ZZ+4.*phi.^2.*WW.*XX.*ZZ+(-1).*WW.^2.* ...

XX.*ZZ+(-4).*phi.*WW.^2.*XX.*ZZ+(-2).*phi.^2.*WW.^2.*XX.*ZZ+(-1).* ...

phi.^2.*WW.*YY.*ZZ+4.*phi.*WW.^2.*YY.*ZZ+phi.^2.*WW.^2.*YY.*ZZ+(-3).* ...

WW.^3.*YY.*ZZ+(-2).*phi.*WW.^3.*YY.*ZZ+phi.^2.*WW.^3.*YY.*ZZ+(-1).* ...

WW.^4.*YY.*ZZ+(-2).*phi.*WW.^4.*YY.*ZZ+(-1).*phi.^2.*WW.^4.*YY.*ZZ+ ...

phi.^2.*XX.*YY.*ZZ+(-4).*phi.*WW.*XX.*YY.*ZZ+(-1).*phi.^2.*WW.*XX.*YY.* ...

ZZ+3.*WW.^2.*XX.*YY.*ZZ+2.*phi.*WW.^2.*XX.*YY.*ZZ+(-1).*phi.^2.*WW.^2.* ...

XX.*YY.*ZZ+WW.^3.*XX.*YY.*ZZ+2.*phi.*WW.^3.*XX.*YY.*ZZ+phi.^2.*WW.^3.* ...

XX.*YY.*ZZ)+27.*(WW.^4+(-1).*WW.^3.*XX+WW.^3.*YY+(-2).*WW.^4.*YY+WW.^4.* ...

XX.*YY+(-1).*WW.^3.*ZZ+2.*WW.^3.*XX.*ZZ+(-1).*WW.^4.*XX.*ZZ+WW.^4.*YY.* ...

ZZ+(-1).*WW.^3.*XX.*YY.*ZZ).*((-1).*WW+2.*phi.*WW+(-1).*phi.^2.*WW+(-3) ...

.*WW.^2+2.*phi.*WW.^2+phi.^2.*WW.^2+(-4).*phi.*WW.^3+phi.^2.*WW.^3+(-1) ...

.*phi.^2.*WW.^4+XX+(-2).*phi.*XX+phi.^2.*XX+3.*WW.*XX+(-2).*phi.*WW.*XX+ ...

(-1).*phi.^2.*WW.*XX+4.*phi.*WW.^2.*XX+(-1).*phi.^2.*WW.^2.*XX+phi.^2.* ...

WW.^3.*XX+WW.^2.*YY+(-4).*phi.*WW.^2.*YY+2.*phi.^2.*WW.^2.*YY+3.*WW.^3.* ...

YY+2.*phi.*WW.^3.*YY+(-4).*phi.^2.*WW.^3.*YY+2.*phi.*WW.^4.*YY+2.* ...

phi.^2.*WW.^4.*YY+(-1).*WW.*XX.*YY+4.*phi.*WW.*XX.*YY+(-2).*phi.^2.*WW.* ...

XX.*YY+(-3).*WW.^2.*XX.*YY+(-2).*phi.*WW.^2.*XX.*YY+4.*phi.^2.*WW.^2.* ...

XX.*YY+(-2).*phi.*WW.^3.*XX.*YY+(-2).*phi.^2.*WW.^3.*XX.*YY+(-2).*phi.* ...

WW.*ZZ+2.*phi.^2.*WW.*ZZ+3.*WW.^2.*ZZ+(-2).*phi.*WW.^2.*ZZ+(-4).* ...

phi.^2.*WW.^2.*ZZ+WW.^3.*ZZ+4.*phi.*WW.^3.*ZZ+2.*phi.^2.*WW.^3.*ZZ+2.* ...

phi.*XX.*ZZ+(-2).*phi.^2.*XX.*ZZ+(-3).*WW.*XX.*ZZ+2.*phi.*WW.*XX.*ZZ+4.* ...

phi.^2.*WW.*XX.*ZZ+(-1).*WW.^2.*XX.*ZZ+(-4).*phi.*WW.^2.*XX.*ZZ+(-2).* ...

phi.^2.*WW.^2.*XX.*ZZ+(-1).*phi.^2.*WW.*YY.*ZZ+4.*phi.*WW.^2.*YY.*ZZ+ ...

phi.^2.*WW.^2.*YY.*ZZ+(-3).*WW.^3.*YY.*ZZ+(-2).*phi.*WW.^3.*YY.*ZZ+ ...

phi.^2.*WW.^3.*YY.*ZZ+(-1).*WW.^4.*YY.*ZZ+(-2).*phi.*WW.^4.*YY.*ZZ+(-1) ...

.*phi.^2.*WW.^4.*YY.*ZZ+phi.^2.*XX.*YY.*ZZ+(-4).*phi.*WW.*XX.*YY.*ZZ+( ...

-1).*phi.^2.*WW.*XX.*YY.*ZZ+3.*WW.^2.*XX.*YY.*ZZ+2.*phi.*WW.^2.*XX.*YY.* ...

ZZ+(-1).*phi.^2.*WW.^2.*XX.*YY.*ZZ+WW.^3.*XX.*YY.*ZZ+2.*phi.*WW.^3.*XX.* ...

YY.*ZZ+phi.^2.*WW.^3.*XX.*YY.*ZZ).^2+((-4).*(12.*(WW+(-2).*phi.*WW+ ...

phi.^2.*WW+2.*phi.*WW.^2+(-2).*phi.^2.*WW.^2+phi.^2.*WW.^3+(-1).*XX+2.* ...

phi.*XX+(-1).*phi.^2.*XX+(-2).*phi.*WW.*XX+2.*phi.^2.*WW.*XX+(-1).* ...

phi.^2.*WW.^2.*XX+phi.*WW.*YY+(-1).*phi.^2.*WW.*YY+(-1).*WW.^2.*YY+2.* ...

phi.^2.*WW.^2.*YY+(-1).*phi.*WW.^3.*YY+(-1).*phi.^2.*WW.^3.*YY+(-1).* ...

phi.*XX.*YY+phi.^2.*XX.*YY+WW.*XX.*YY+(-2).*phi.^2.*WW.*XX.*YY+phi.* ...

WW.^2.*XX.*YY+phi.^2.*WW.^2.*XX.*YY+phi.*WW.*ZZ+(-1).*phi.^2.*WW.*ZZ+( ...

-1).*WW.^2.*ZZ+2.*phi.^2.*WW.^2.*ZZ+(-1).*phi.*WW.^3.*ZZ+(-1).*phi.^2.* ...

WW.^3.*ZZ+(-1).*phi.*XX.*ZZ+phi.^2.*XX.*ZZ+WW.*XX.*ZZ+(-2).*phi.^2.*WW.* ...

XX.*ZZ+phi.*WW.^2.*XX.*ZZ+phi.^2.*WW.^2.*XX.*ZZ+phi.^2.*WW.*YY.*ZZ+(-2) ...

.*phi.*WW.^2.*YY.*ZZ+(-2).*phi.^2.*WW.^2.*YY.*ZZ+WW.^3.*YY.*ZZ+2.*phi.* ...

WW.^3.*YY.*ZZ+phi.^2.*WW.^3.*YY.*ZZ+(-1).*phi.^2.*XX.*YY.*ZZ+2.*phi.* ...

WW.*XX.*YY.*ZZ+2.*phi.^2.*WW.*XX.*YY.*ZZ+(-1).*WW.^2.*XX.*YY.*ZZ+(-2).* ...

phi.*WW.^2.*XX.*YY.*ZZ+(-1).*phi.^2.*WW.^2.*XX.*YY.*ZZ).*(WW.^4+(-1).* ...

WW.^3.*XX+WW.^3.*YY+(-2).*WW.^4.*YY+WW.^4.*XX.*YY+(-1).*WW.^3.*ZZ+2.* ...

WW.^3.*XX.*ZZ+(-1).*WW.^4.*XX.*ZZ+WW.^4.*YY.*ZZ+(-1).*WW.^3.*XX.*YY.*ZZ) ...

+(3.*WW.^2+(-4).*phi.*WW.^2+phi.^2.*WW.^2+3.*WW.^3+2.*phi.*WW.^3+(-2).* ...

phi.^2.*WW.^3+2.*phi.*WW.^4+phi.^2.*WW.^4+(-3).*WW.*XX+4.*phi.*WW.*XX+( ...

-1).*phi.^2.*WW.*XX+(-3).*WW.^2.*XX+(-2).*phi.*WW.^2.*XX+2.*phi.^2.* ...

WW.^2.*XX+(-2).*phi.*WW.^3.*XX+(-1).*phi.^2.*WW.^3.*XX+(-1).*phi.*WW.* ...

YY+phi.^2.*WW.*YY+WW.^2.*YY+2.*phi.*WW.^2.*YY+(-4).*phi.^2.*WW.^2.*YY+( ...

-5).*WW.^3.*YY+5.*phi.*WW.^3.*YY+5.*phi.^2.*WW.^3.*YY+(-2).*WW.^4.*YY+( ...

-6).*phi.*WW.^4.*YY+(-2).*phi.^2.*WW.^4.*YY+3.*WW.^2.*XX.*YY+(-5).*phi.* ...

WW.^2.*XX.*YY+phi.^2.*WW.^2.*XX.*YY+3.*WW.^3.*XX.*YY+4.*phi.*WW.^3.*XX.* ...

YY+(-2).*phi.^2.*WW.^3.*XX.*YY+phi.*WW.^4.*XX.*YY+phi.^2.*WW.^4.*XX.*YY+ ...

phi.*WW.*ZZ+(-1).*phi.^2.*WW.*ZZ+(-3).*WW.^2.*ZZ+4.*phi.*WW.^2.*ZZ+2.* ...

phi.^2.*WW.^2.*ZZ+(-3).*WW.^3.*ZZ+(-5).*phi.*WW.^3.*ZZ+(-1).*phi.^2.* ...

WW.^3.*ZZ+2.*WW.*XX.*ZZ+(-6).*phi.*WW.*XX.*ZZ+2.*phi.^2.*WW.*XX.*ZZ+5.* ...

WW.^2.*XX.*ZZ+5.*phi.*WW.^2.*XX.*ZZ+(-5).*phi.^2.*WW.^2.*XX.*ZZ+(-1).* ...

WW.^3.*XX.*ZZ+2.*phi.*WW.^3.*XX.*ZZ+4.*phi.^2.*WW.^3.*XX.*ZZ+(-1).*phi.* ...

WW.^4.*XX.*ZZ+(-1).*phi.^2.*WW.^4.*XX.*ZZ+(-2).*phi.*WW.^2.*YY.*ZZ+ ...

phi.^2.*WW.^2.*YY.*ZZ+3.*WW.^3.*YY.*ZZ+(-2).*phi.*WW.^3.*YY.*ZZ+(-2).* ...

phi.^2.*WW.^3.*YY.*ZZ+3.*WW.^4.*YY.*ZZ+4.*phi.*WW.^4.*YY.*ZZ+phi.^2.* ...

WW.^4.*YY.*ZZ+2.*phi.*WW.*XX.*YY.*ZZ+(-1).*phi.^2.*WW.*XX.*YY.*ZZ+(-3).* ...

WW.^2.*XX.*YY.*ZZ+2.*phi.*WW.^2.*XX.*YY.*ZZ+2.*phi.^2.*WW.^2.*XX.*YY.* ...

ZZ+(-3).*WW.^3.*XX.*YY.*ZZ+(-4).*phi.*WW.^3.*XX.*YY.*ZZ+(-1).*phi.^2.* ...

WW.^3.*XX.*YY.*ZZ).^2+(-3).*((-3).*WW.^3+2.*phi.*WW.^3+(-1).*WW.^4+(-2) ...

.*phi.*WW.^4+3.*WW.^2.*XX+(-2).*phi.*WW.^2.*XX+WW.^3.*XX+2.*phi.*WW.^3.* ...

XX+(-1).*WW.^2.*YY+2.*phi.*WW.^2.*YY+WW.^3.*YY+(-6).*phi.*WW.^3.*YY+4.* ...

WW.^4.*YY+4.*phi.*WW.^4.*YY+(-3).*WW.^3.*XX.*YY+2.*phi.*WW.^3.*XX.*YY+( ...

-1).*WW.^4.*XX.*YY+(-2).*phi.*WW.^4.*XX.*YY+WW.^2.*ZZ+(-2).*phi.*WW.^2.* ...

ZZ+3.*WW.^3.*ZZ+2.*phi.*WW.^3.*ZZ+(-4).*WW.^2.*XX.*ZZ+4.*phi.*WW.^2.* ...

XX.*ZZ+(-1).*WW.^3.*XX.*ZZ+(-6).*phi.*WW.^3.*XX.*ZZ+WW.^4.*XX.*ZZ+2.* ...

phi.*WW.^4.*XX.*ZZ+(-1).*WW.^3.*YY.*ZZ+2.*phi.*WW.^3.*YY.*ZZ+(-3).* ...

WW.^4.*YY.*ZZ+(-2).*phi.*WW.^4.*YY.*ZZ+WW.^2.*XX.*YY.*ZZ+(-2).*phi.* ...

WW.^2.*XX.*YY.*ZZ+3.*WW.^3.*XX.*YY.*ZZ+2.*phi.*WW.^3.*XX.*YY.*ZZ).*((-1) ...

.*WW+2.*phi.*WW+(-1).*phi.^2.*WW+(-3).*WW.^2+2.*phi.*WW.^2+phi.^2.* ...

WW.^2+(-4).*phi.*WW.^3+phi.^2.*WW.^3+(-1).*phi.^2.*WW.^4+XX+(-2).*phi.* ...

XX+phi.^2.*XX+3.*WW.*XX+(-2).*phi.*WW.*XX+(-1).*phi.^2.*WW.*XX+4.*phi.* ...

WW.^2.*XX+(-1).*phi.^2.*WW.^2.*XX+phi.^2.*WW.^3.*XX+WW.^2.*YY+(-4).* ...

phi.*WW.^2.*YY+2.*phi.^2.*WW.^2.*YY+3.*WW.^3.*YY+2.*phi.*WW.^3.*YY+(-4) ...

.*phi.^2.*WW.^3.*YY+2.*phi.*WW.^4.*YY+2.*phi.^2.*WW.^4.*YY+(-1).*WW.* ...

XX.*YY+4.*phi.*WW.*XX.*YY+(-2).*phi.^2.*WW.*XX.*YY+(-3).*WW.^2.*XX.*YY+( ...

-2).*phi.*WW.^2.*XX.*YY+4.*phi.^2.*WW.^2.*XX.*YY+(-2).*phi.*WW.^3.*XX.* ...

YY+(-2).*phi.^2.*WW.^3.*XX.*YY+(-2).*phi.*WW.*ZZ+2.*phi.^2.*WW.*ZZ+3.* ...

WW.^2.*ZZ+(-2).*phi.*WW.^2.*ZZ+(-4).*phi.^2.*WW.^2.*ZZ+WW.^3.*ZZ+4.* ...

phi.*WW.^3.*ZZ+2.*phi.^2.*WW.^3.*ZZ+2.*phi.*XX.*ZZ+(-2).*phi.^2.*XX.*ZZ+ ...

(-3).*WW.*XX.*ZZ+2.*phi.*WW.*XX.*ZZ+4.*phi.^2.*WW.*XX.*ZZ+(-1).*WW.^2.* ...

XX.*ZZ+(-4).*phi.*WW.^2.*XX.*ZZ+(-2).*phi.^2.*WW.^2.*XX.*ZZ+(-1).* ...

phi.^2.*WW.*YY.*ZZ+4.*phi.*WW.^2.*YY.*ZZ+phi.^2.*WW.^2.*YY.*ZZ+(-3).* ...

WW.^3.*YY.*ZZ+(-2).*phi.*WW.^3.*YY.*ZZ+phi.^2.*WW.^3.*YY.*ZZ+(-1).* ...

WW.^4.*YY.*ZZ+(-2).*phi.*WW.^4.*YY.*ZZ+(-1).*phi.^2.*WW.^4.*YY.*ZZ+ ...

phi.^2.*XX.*YY.*ZZ+(-4).*phi.*WW.*XX.*YY.*ZZ+(-1).*phi.^2.*WW.*XX.*YY.* ...

ZZ+3.*WW.^2.*XX.*YY.*ZZ+2.*phi.*WW.^2.*XX.*YY.*ZZ+(-1).*phi.^2.*WW.^2.* ...

XX.*YY.*ZZ+WW.^3.*XX.*YY.*ZZ+2.*phi.*WW.^3.*XX.*YY.*ZZ+phi.^2.*WW.^3.* ...

XX.*YY.*ZZ)).^3+(27.*(WW+(-2).*phi.*WW+phi.^2.*WW+2.*phi.*WW.^2+(-2).* ...

phi.^2.*WW.^2+phi.^2.*WW.^3+(-1).*XX+2.*phi.*XX+(-1).*phi.^2.*XX+(-2).* ...

phi.*WW.*XX+2.*phi.^2.*WW.*XX+(-1).*phi.^2.*WW.^2.*XX+phi.*WW.*YY+(-1).* ...

phi.^2.*WW.*YY+(-1).*WW.^2.*YY+2.*phi.^2.*WW.^2.*YY+(-1).*phi.*WW.^3.* ...

YY+(-1).*phi.^2.*WW.^3.*YY+(-1).*phi.*XX.*YY+phi.^2.*XX.*YY+WW.*XX.*YY+( ...

-2).*phi.^2.*WW.*XX.*YY+phi.*WW.^2.*XX.*YY+phi.^2.*WW.^2.*XX.*YY+phi.* ...

WW.*ZZ+(-1).*phi.^2.*WW.*ZZ+(-1).*WW.^2.*ZZ+2.*phi.^2.*WW.^2.*ZZ+(-1).* ...

phi.*WW.^3.*ZZ+(-1).*phi.^2.*WW.^3.*ZZ+(-1).*phi.*XX.*ZZ+phi.^2.*XX.*ZZ+ ...

WW.*XX.*ZZ+(-2).*phi.^2.*WW.*XX.*ZZ+phi.*WW.^2.*XX.*ZZ+phi.^2.*WW.^2.* ...

XX.*ZZ+phi.^2.*WW.*YY.*ZZ+(-2).*phi.*WW.^2.*YY.*ZZ+(-2).*phi.^2.*WW.^2.* ...

YY.*ZZ+WW.^3.*YY.*ZZ+2.*phi.*WW.^3.*YY.*ZZ+phi.^2.*WW.^3.*YY.*ZZ+(-1).* ...

phi.^2.*XX.*YY.*ZZ+2.*phi.*WW.*XX.*YY.*ZZ+2.*phi.^2.*WW.*XX.*YY.*ZZ+(-1) ...

.*WW.^2.*XX.*YY.*ZZ+(-2).*phi.*WW.^2.*XX.*YY.*ZZ+(-1).*phi.^2.*WW.^2.* ...

XX.*YY.*ZZ).*((-3).*WW.^3+2.*phi.*WW.^3+(-1).*WW.^4+(-2).*phi.*WW.^4+3.* ...

WW.^2.*XX+(-2).*phi.*WW.^2.*XX+WW.^3.*XX+2.*phi.*WW.^3.*XX+(-1).*WW.^2.* ...

YY+2.*phi.*WW.^2.*YY+WW.^3.*YY+(-6).*phi.*WW.^3.*YY+4.*WW.^4.*YY+4.* ...

phi.*WW.^4.*YY+(-3).*WW.^3.*XX.*YY+2.*phi.*WW.^3.*XX.*YY+(-1).*WW.^4.* ...

XX.*YY+(-2).*phi.*WW.^4.*XX.*YY+WW.^2.*ZZ+(-2).*phi.*WW.^2.*ZZ+3.* ...

WW.^3.*ZZ+2.*phi.*WW.^3.*ZZ+(-4).*WW.^2.*XX.*ZZ+4.*phi.*WW.^2.*XX.*ZZ+( ...

-1).*WW.^3.*XX.*ZZ+(-6).*phi.*WW.^3.*XX.*ZZ+WW.^4.*XX.*ZZ+2.*phi.* ...

WW.^4.*XX.*ZZ+(-1).*WW.^3.*YY.*ZZ+2.*phi.*WW.^3.*YY.*ZZ+(-3).*WW.^4.* ...

YY.*ZZ+(-2).*phi.*WW.^4.*YY.*ZZ+WW.^2.*XX.*YY.*ZZ+(-2).*phi.*WW.^2.*XX.* ...

YY.*ZZ+3.*WW.^3.*XX.*YY.*ZZ+2.*phi.*WW.^3.*XX.*YY.*ZZ).^2+(-72).*(WW+( ...

-2).*phi.*WW+phi.^2.*WW+2.*phi.*WW.^2+(-2).*phi.^2.*WW.^2+phi.^2.*WW.^3+ ...

(-1).*XX+2.*phi.*XX+(-1).*phi.^2.*XX+(-2).*phi.*WW.*XX+2.*phi.^2.*WW.* ...

XX+(-1).*phi.^2.*WW.^2.*XX+phi.*WW.*YY+(-1).*phi.^2.*WW.*YY+(-1).* ...

WW.^2.*YY+2.*phi.^2.*WW.^2.*YY+(-1).*phi.*WW.^3.*YY+(-1).*phi.^2.* ...

WW.^3.*YY+(-1).*phi.*XX.*YY+phi.^2.*XX.*YY+WW.*XX.*YY+(-2).*phi.^2.*WW.* ...

XX.*YY+phi.*WW.^2.*XX.*YY+phi.^2.*WW.^2.*XX.*YY+phi.*WW.*ZZ+(-1).* ...

phi.^2.*WW.*ZZ+(-1).*WW.^2.*ZZ+2.*phi.^2.*WW.^2.*ZZ+(-1).*phi.*WW.^3.* ...

ZZ+(-1).*phi.^2.*WW.^3.*ZZ+(-1).*phi.*XX.*ZZ+phi.^2.*XX.*ZZ+WW.*XX.*ZZ+( ...

-2).*phi.^2.*WW.*XX.*ZZ+phi.*WW.^2.*XX.*ZZ+phi.^2.*WW.^2.*XX.*ZZ+ ...

phi.^2.*WW.*YY.*ZZ+(-2).*phi.*WW.^2.*YY.*ZZ+(-2).*phi.^2.*WW.^2.*YY.*ZZ+ ...

WW.^3.*YY.*ZZ+2.*phi.*WW.^3.*YY.*ZZ+phi.^2.*WW.^3.*YY.*ZZ+(-1).*phi.^2.* ...

XX.*YY.*ZZ+2.*phi.*WW.*XX.*YY.*ZZ+2.*phi.^2.*WW.*XX.*YY.*ZZ+(-1).* ...

WW.^2.*XX.*YY.*ZZ+(-2).*phi.*WW.^2.*XX.*YY.*ZZ+(-1).*phi.^2.*WW.^2.*XX.* ...

YY.*ZZ).*(WW.^4+(-1).*WW.^3.*XX+WW.^3.*YY+(-2).*WW.^4.*YY+WW.^4.*XX.*YY+ ...

(-1).*WW.^3.*ZZ+2.*WW.^3.*XX.*ZZ+(-1).*WW.^4.*XX.*ZZ+WW.^4.*YY.*ZZ+(-1) ...

.*WW.^3.*XX.*YY.*ZZ).*(3.*WW.^2+(-4).*phi.*WW.^2+phi.^2.*WW.^2+3.*WW.^3+ ...

2.*phi.*WW.^3+(-2).*phi.^2.*WW.^3+2.*phi.*WW.^4+phi.^2.*WW.^4+(-3).*WW.* ...

XX+4.*phi.*WW.*XX+(-1).*phi.^2.*WW.*XX+(-3).*WW.^2.*XX+(-2).*phi.* ...

WW.^2.*XX+2.*phi.^2.*WW.^2.*XX+(-2).*phi.*WW.^3.*XX+(-1).*phi.^2.* ...

WW.^3.*XX+(-1).*phi.*WW.*YY+phi.^2.*WW.*YY+WW.^2.*YY+2.*phi.*WW.^2.*YY+( ...

-4).*phi.^2.*WW.^2.*YY+(-5).*WW.^3.*YY+5.*phi.*WW.^3.*YY+5.*phi.^2.* ...

WW.^3.*YY+(-2).*WW.^4.*YY+(-6).*phi.*WW.^4.*YY+(-2).*phi.^2.*WW.^4.*YY+ ...

3.*WW.^2.*XX.*YY+(-5).*phi.*WW.^2.*XX.*YY+phi.^2.*WW.^2.*XX.*YY+3.* ...

WW.^3.*XX.*YY+4.*phi.*WW.^3.*XX.*YY+(-2).*phi.^2.*WW.^3.*XX.*YY+phi.* ...

WW.^4.*XX.*YY+phi.^2.*WW.^4.*XX.*YY+phi.*WW.*ZZ+(-1).*phi.^2.*WW.*ZZ+( ...

-3).*WW.^2.*ZZ+4.*phi.*WW.^2.*ZZ+2.*phi.^2.*WW.^2.*ZZ+(-3).*WW.^3.*ZZ+( ...

-5).*phi.*WW.^3.*ZZ+(-1).*phi.^2.*WW.^3.*ZZ+2.*WW.*XX.*ZZ+(-6).*phi.* ...

WW.*XX.*ZZ+2.*phi.^2.*WW.*XX.*ZZ+5.*WW.^2.*XX.*ZZ+5.*phi.*WW.^2.*XX.*ZZ+ ...

(-5).*phi.^2.*WW.^2.*XX.*ZZ+(-1).*WW.^3.*XX.*ZZ+2.*phi.*WW.^3.*XX.*ZZ+ ...

4.*phi.^2.*WW.^3.*XX.*ZZ+(-1).*phi.*WW.^4.*XX.*ZZ+(-1).*phi.^2.*WW.^4.* ...

XX.*ZZ+(-2).*phi.*WW.^2.*YY.*ZZ+phi.^2.*WW.^2.*YY.*ZZ+3.*WW.^3.*YY.*ZZ+( ...

-2).*phi.*WW.^3.*YY.*ZZ+(-2).*phi.^2.*WW.^3.*YY.*ZZ+3.*WW.^4.*YY.*ZZ+4.* ...

phi.*WW.^4.*YY.*ZZ+phi.^2.*WW.^4.*YY.*ZZ+2.*phi.*WW.*XX.*YY.*ZZ+(-1).* ...

phi.^2.*WW.*XX.*YY.*ZZ+(-3).*WW.^2.*XX.*YY.*ZZ+2.*phi.*WW.^2.*XX.*YY.* ...

ZZ+2.*phi.^2.*WW.^2.*XX.*YY.*ZZ+(-3).*WW.^3.*XX.*YY.*ZZ+(-4).*phi.* ...

WW.^3.*XX.*YY.*ZZ+(-1).*phi.^2.*WW.^3.*XX.*YY.*ZZ)+2.*(3.*WW.^2+(-4).* ...

phi.*WW.^2+phi.^2.*WW.^2+3.*WW.^3+2.*phi.*WW.^3+(-2).*phi.^2.*WW.^3+2.* ...

phi.*WW.^4+phi.^2.*WW.^4+(-3).*WW.*XX+4.*phi.*WW.*XX+(-1).*phi.^2.*WW.* ...

XX+(-3).*WW.^2.*XX+(-2).*phi.*WW.^2.*XX+2.*phi.^2.*WW.^2.*XX+(-2).*phi.* ...

WW.^3.*XX+(-1).*phi.^2.*WW.^3.*XX+(-1).*phi.*WW.*YY+phi.^2.*WW.*YY+ ...

WW.^2.*YY+2.*phi.*WW.^2.*YY+(-4).*phi.^2.*WW.^2.*YY+(-5).*WW.^3.*YY+5.* ...

phi.*WW.^3.*YY+5.*phi.^2.*WW.^3.*YY+(-2).*WW.^4.*YY+(-6).*phi.*WW.^4.* ...

YY+(-2).*phi.^2.*WW.^4.*YY+3.*WW.^2.*XX.*YY+(-5).*phi.*WW.^2.*XX.*YY+ ...

phi.^2.*WW.^2.*XX.*YY+3.*WW.^3.*XX.*YY+4.*phi.*WW.^3.*XX.*YY+(-2).* ...

phi.^2.*WW.^3.*XX.*YY+phi.*WW.^4.*XX.*YY+phi.^2.*WW.^4.*XX.*YY+phi.*WW.* ...

ZZ+(-1).*phi.^2.*WW.*ZZ+(-3).*WW.^2.*ZZ+4.*phi.*WW.^2.*ZZ+2.*phi.^2.* ...

WW.^2.*ZZ+(-3).*WW.^3.*ZZ+(-5).*phi.*WW.^3.*ZZ+(-1).*phi.^2.*WW.^3.*ZZ+ ...

2.*WW.*XX.*ZZ+(-6).*phi.*WW.*XX.*ZZ+2.*phi.^2.*WW.*XX.*ZZ+5.*WW.^2.*XX.* ...

ZZ+5.*phi.*WW.^2.*XX.*ZZ+(-5).*phi.^2.*WW.^2.*XX.*ZZ+(-1).*WW.^3.*XX.* ...

ZZ+2.*phi.*WW.^3.*XX.*ZZ+4.*phi.^2.*WW.^3.*XX.*ZZ+(-1).*phi.*WW.^4.*XX.* ...

ZZ+(-1).*phi.^2.*WW.^4.*XX.*ZZ+(-2).*phi.*WW.^2.*YY.*ZZ+phi.^2.*WW.^2.* ...

YY.*ZZ+3.*WW.^3.*YY.*ZZ+(-2).*phi.*WW.^3.*YY.*ZZ+(-2).*phi.^2.*WW.^3.* ...

YY.*ZZ+3.*WW.^4.*YY.*ZZ+4.*phi.*WW.^4.*YY.*ZZ+phi.^2.*WW.^4.*YY.*ZZ+2.* ...

phi.*WW.*XX.*YY.*ZZ+(-1).*phi.^2.*WW.*XX.*YY.*ZZ+(-3).*WW.^2.*XX.*YY.* ...

ZZ+2.*phi.*WW.^2.*XX.*YY.*ZZ+2.*phi.^2.*WW.^2.*XX.*YY.*ZZ+(-3).*WW.^3.* ...

XX.*YY.*ZZ+(-4).*phi.*WW.^3.*XX.*YY.*ZZ+(-1).*phi.^2.*WW.^3.*XX.*YY.*ZZ) ...

.^3+(-9).*((-3).*WW.^3+2.*phi.*WW.^3+(-1).*WW.^4+(-2).*phi.*WW.^4+3.* ...

WW.^2.*XX+(-2).*phi.*WW.^2.*XX+WW.^3.*XX+2.*phi.*WW.^3.*XX+(-1).*WW.^2.* ...

YY+2.*phi.*WW.^2.*YY+WW.^3.*YY+(-6).*phi.*WW.^3.*YY+4.*WW.^4.*YY+4.* ...

phi.*WW.^4.*YY+(-3).*WW.^3.*XX.*YY+2.*phi.*WW.^3.*XX.*YY+(-1).*WW.^4.* ...

XX.*YY+(-2).*phi.*WW.^4.*XX.*YY+WW.^2.*ZZ+(-2).*phi.*WW.^2.*ZZ+3.* ...

WW.^3.*ZZ+2.*phi.*WW.^3.*ZZ+(-4).*WW.^2.*XX.*ZZ+4.*phi.*WW.^2.*XX.*ZZ+( ...

-1).*WW.^3.*XX.*ZZ+(-6).*phi.*WW.^3.*XX.*ZZ+WW.^4.*XX.*ZZ+2.*phi.* ...

WW.^4.*XX.*ZZ+(-1).*WW.^3.*YY.*ZZ+2.*phi.*WW.^3.*YY.*ZZ+(-3).*WW.^4.* ...

YY.*ZZ+(-2).*phi.*WW.^4.*YY.*ZZ+WW.^2.*XX.*YY.*ZZ+(-2).*phi.*WW.^2.*XX.* ...

YY.*ZZ+3.*WW.^3.*XX.*YY.*ZZ+2.*phi.*WW.^3.*XX.*YY.*ZZ).*(3.*WW.^2+(-4).* ...

phi.*WW.^2+phi.^2.*WW.^2+3.*WW.^3+2.*phi.*WW.^3+(-2).*phi.^2.*WW.^3+2.* ...

phi.*WW.^4+phi.^2.*WW.^4+(-3).*WW.*XX+4.*phi.*WW.*XX+(-1).*phi.^2.*WW.* ...

XX+(-3).*WW.^2.*XX+(-2).*phi.*WW.^2.*XX+2.*phi.^2.*WW.^2.*XX+(-2).*phi.* ...

WW.^3.*XX+(-1).*phi.^2.*WW.^3.*XX+(-1).*phi.*WW.*YY+phi.^2.*WW.*YY+ ...

WW.^2.*YY+2.*phi.*WW.^2.*YY+(-4).*phi.^2.*WW.^2.*YY+(-5).*WW.^3.*YY+5.* ...

phi.*WW.^3.*YY+5.*phi.^2.*WW.^3.*YY+(-2).*WW.^4.*YY+(-6).*phi.*WW.^4.* ...

YY+(-2).*phi.^2.*WW.^4.*YY+3.*WW.^2.*XX.*YY+(-5).*phi.*WW.^2.*XX.*YY+ ...

phi.^2.*WW.^2.*XX.*YY+3.*WW.^3.*XX.*YY+4.*phi.*WW.^3.*XX.*YY+(-2).* ...

phi.^2.*WW.^3.*XX.*YY+phi.*WW.^4.*XX.*YY+phi.^2.*WW.^4.*XX.*YY+phi.*WW.* ...

ZZ+(-1).*phi.^2.*WW.*ZZ+(-3).*WW.^2.*ZZ+4.*phi.*WW.^2.*ZZ+2.*phi.^2.* ...

WW.^2.*ZZ+(-3).*WW.^3.*ZZ+(-5).*phi.*WW.^3.*ZZ+(-1).*phi.^2.*WW.^3.*ZZ+ ...

2.*WW.*XX.*ZZ+(-6).*phi.*WW.*XX.*ZZ+2.*phi.^2.*WW.*XX.*ZZ+5.*WW.^2.*XX.* ...

ZZ+5.*phi.*WW.^2.*XX.*ZZ+(-5).*phi.^2.*WW.^2.*XX.*ZZ+(-1).*WW.^3.*XX.* ...

ZZ+2.*phi.*WW.^3.*XX.*ZZ+4.*phi.^2.*WW.^3.*XX.*ZZ+(-1).*phi.*WW.^4.*XX.* ...

ZZ+(-1).*phi.^2.*WW.^4.*XX.*ZZ+(-2).*phi.*WW.^2.*YY.*ZZ+phi.^2.*WW.^2.* ...

YY.*ZZ+3.*WW.^3.*YY.*ZZ+(-2).*phi.*WW.^3.*YY.*ZZ+(-2).*phi.^2.*WW.^3.* ...

YY.*ZZ+3.*WW.^4.*YY.*ZZ+4.*phi.*WW.^4.*YY.*ZZ+phi.^2.*WW.^4.*YY.*ZZ+2.* ...

phi.*WW.*XX.*YY.*ZZ+(-1).*phi.^2.*WW.*XX.*YY.*ZZ+(-3).*WW.^2.*XX.*YY.* ...

ZZ+2.*phi.*WW.^2.*XX.*YY.*ZZ+2.*phi.^2.*WW.^2.*XX.*YY.*ZZ+(-3).*WW.^3.* ...

XX.*YY.*ZZ+(-4).*phi.*WW.^3.*XX.*YY.*ZZ+(-1).*phi.^2.*WW.^3.*XX.*YY.*ZZ) ...

.*((-1).*WW+2.*phi.*WW+(-1).*phi.^2.*WW+(-3).*WW.^2+2.*phi.*WW.^2+ ...

phi.^2.*WW.^2+(-4).*phi.*WW.^3+phi.^2.*WW.^3+(-1).*phi.^2.*WW.^4+XX+(-2) ...

.*phi.*XX+phi.^2.*XX+3.*WW.*XX+(-2).*phi.*WW.*XX+(-1).*phi.^2.*WW.*XX+ ...

4.*phi.*WW.^2.*XX+(-1).*phi.^2.*WW.^2.*XX+phi.^2.*WW.^3.*XX+WW.^2.*YY+( ...

-4).*phi.*WW.^2.*YY+2.*phi.^2.*WW.^2.*YY+3.*WW.^3.*YY+2.*phi.*WW.^3.*YY+ ...

(-4).*phi.^2.*WW.^3.*YY+2.*phi.*WW.^4.*YY+2.*phi.^2.*WW.^4.*YY+(-1).* ...

WW.*XX.*YY+4.*phi.*WW.*XX.*YY+(-2).*phi.^2.*WW.*XX.*YY+(-3).*WW.^2.*XX.* ...

YY+(-2).*phi.*WW.^2.*XX.*YY+4.*phi.^2.*WW.^2.*XX.*YY+(-2).*phi.*WW.^3.* ...

XX.*YY+(-2).*phi.^2.*WW.^3.*XX.*YY+(-2).*phi.*WW.*ZZ+2.*phi.^2.*WW.*ZZ+ ...

3.*WW.^2.*ZZ+(-2).*phi.*WW.^2.*ZZ+(-4).*phi.^2.*WW.^2.*ZZ+WW.^3.*ZZ+4.* ...

phi.*WW.^3.*ZZ+2.*phi.^2.*WW.^3.*ZZ+2.*phi.*XX.*ZZ+(-2).*phi.^2.*XX.*ZZ+ ...

(-3).*WW.*XX.*ZZ+2.*phi.*WW.*XX.*ZZ+4.*phi.^2.*WW.*XX.*ZZ+(-1).*WW.^2.* ...

XX.*ZZ+(-4).*phi.*WW.^2.*XX.*ZZ+(-2).*phi.^2.*WW.^2.*XX.*ZZ+(-1).* ...

phi.^2.*WW.*YY.*ZZ+4.*phi.*WW.^2.*YY.*ZZ+phi.^2.*WW.^2.*YY.*ZZ+(-3).* ...

WW.^3.*YY.*ZZ+(-2).*phi.*WW.^3.*YY.*ZZ+phi.^2.*WW.^3.*YY.*ZZ+(-1).* ...

WW.^4.*YY.*ZZ+(-2).*phi.*WW.^4.*YY.*ZZ+(-1).*phi.^2.*WW.^4.*YY.*ZZ+ ...

phi.^2.*XX.*YY.*ZZ+(-4).*phi.*WW.*XX.*YY.*ZZ+(-1).*phi.^2.*WW.*XX.*YY.* ...

ZZ+3.*WW.^2.*XX.*YY.*ZZ+2.*phi.*WW.^2.*XX.*YY.*ZZ+(-1).*phi.^2.*WW.^2.* ...

XX.*YY.*ZZ+WW.^3.*XX.*YY.*ZZ+2.*phi.*WW.^3.*XX.*YY.*ZZ+phi.^2.*WW.^3.* ...

XX.*YY.*ZZ)+27.*(WW.^4+(-1).*WW.^3.*XX+WW.^3.*YY+(-2).*WW.^4.*YY+WW.^4.* ...

XX.*YY+(-1).*WW.^3.*ZZ+2.*WW.^3.*XX.*ZZ+(-1).*WW.^4.*XX.*ZZ+WW.^4.*YY.* ...

ZZ+(-1).*WW.^3.*XX.*YY.*ZZ).*((-1).*WW+2.*phi.*WW+(-1).*phi.^2.*WW+(-3) ...

.*WW.^2+2.*phi.*WW.^2+phi.^2.*WW.^2+(-4).*phi.*WW.^3+phi.^2.*WW.^3+(-1) ...

.*phi.^2.*WW.^4+XX+(-2).*phi.*XX+phi.^2.*XX+3.*WW.*XX+(-2).*phi.*WW.*XX+ ...

(-1).*phi.^2.*WW.*XX+4.*phi.*WW.^2.*XX+(-1).*phi.^2.*WW.^2.*XX+phi.^2.* ...

WW.^3.*XX+WW.^2.*YY+(-4).*phi.*WW.^2.*YY+2.*phi.^2.*WW.^2.*YY+3.*WW.^3.* ...

YY+2.*phi.*WW.^3.*YY+(-4).*phi.^2.*WW.^3.*YY+2.*phi.*WW.^4.*YY+2.* ...

phi.^2.*WW.^4.*YY+(-1).*WW.*XX.*YY+4.*phi.*WW.*XX.*YY+(-2).*phi.^2.*WW.* ...

XX.*YY+(-3).*WW.^2.*XX.*YY+(-2).*phi.*WW.^2.*XX.*YY+4.*phi.^2.*WW.^2.* ...

XX.*YY+(-2).*phi.*WW.^3.*XX.*YY+(-2).*phi.^2.*WW.^3.*XX.*YY+(-2).*phi.* ...

WW.*ZZ+2.*phi.^2.*WW.*ZZ+3.*WW.^2.*ZZ+(-2).*phi.*WW.^2.*ZZ+(-4).* ...

phi.^2.*WW.^2.*ZZ+WW.^3.*ZZ+4.*phi.*WW.^3.*ZZ+2.*phi.^2.*WW.^3.*ZZ+2.* ...

phi.*XX.*ZZ+(-2).*phi.^2.*XX.*ZZ+(-3).*WW.*XX.*ZZ+2.*phi.*WW.*XX.*ZZ+4.* ...

phi.^2.*WW.*XX.*ZZ+(-1).*WW.^2.*XX.*ZZ+(-4).*phi.*WW.^2.*XX.*ZZ+(-2).* ...

phi.^2.*WW.^2.*XX.*ZZ+(-1).*phi.^2.*WW.*YY.*ZZ+4.*phi.*WW.^2.*YY.*ZZ+ ...

phi.^2.*WW.^2.*YY.*ZZ+(-3).*WW.^3.*YY.*ZZ+(-2).*phi.*WW.^3.*YY.*ZZ+ ...

phi.^2.*WW.^3.*YY.*ZZ+(-1).*WW.^4.*YY.*ZZ+(-2).*phi.*WW.^4.*YY.*ZZ+(-1) ...

.*phi.^2.*WW.^4.*YY.*ZZ+phi.^2.*XX.*YY.*ZZ+(-4).*phi.*WW.*XX.*YY.*ZZ+( ...

-1).*phi.^2.*WW.*XX.*YY.*ZZ+3.*WW.^2.*XX.*YY.*ZZ+2.*phi.*WW.^2.*XX.*YY.* ...

ZZ+(-1).*phi.^2.*WW.^2.*XX.*YY.*ZZ+WW.^3.*XX.*YY.*ZZ+2.*phi.*WW.^3.*XX.* ...

YY.*ZZ+phi.^2.*WW.^3.*XX.*YY.*ZZ).^2).^2).^(1/2)).^(1/3)).^(-1/2)).^( ...

1/2);

l1 = k1.*((-1)+WW).*YY.*(WW+(-1).*WW.*YY+k1.*((-1)+phi.*((-1)+WW).*((-1)+YY)+ ...

WW.*YY)).^(-1);

m1 = k1.*((-1)+WW).*XX.*((-1).*k1.*XX+WW.*((-1)+k1+XX)).^(-1);

n1 = ((-1).*WW+k1.*(phi.*((-1)+WW)+WW)).^(-1).*(((1+(-1).*k1).*k1.*(1+k1+(-2) ...

.*WW).*WW+k1.*(k1+(-1).*WW).^2.*XX).*((-1).*k1.*XX+WW.*((-1)+k1+XX)).^( ...

-1)+k1.*(k1+k1.*phi.*((-1)+WW)+(-1).*WW).*((-1)+WW).*(WW+(-1).*WW.*YY+ ...

k1.*((-1)+phi.*((-1)+WW).*((-1)+YY)+WW.*YY)).^(-1));

PA1 = (k1+(-1).*l1+(-1).*m1+n1).^(-1).*((-1)+phi).^(-1).*(l1+(-1).*n1+(k1+(-1).*m1).*phi);

PA1Alt = (k1+(-1).*l1+(-1).*m1+n1).^(-1).*((-1)).^(-1).*(l1+(-1).*n1);

Is1 = (-1).*(k1+(-1).*l1+(-1).*m1+n1).^2;

S1 = [k1 l1 m1 n1 PA1 Is1 PA1Alt];

if max(imag(S1)) < ImagT

S1 = real(S1);

end

%% Second solution

k2=(-1/4).*((-1)+phi+(-1).*phi.*WW+(-1).*phi.*YY+WW.*YY+phi.*WW.*YY).^(-1) ...

.*((-1)+phi+(-1).*phi.*WW+(-1).*phi.*ZZ+WW.*ZZ+phi.*WW.*ZZ).^(-1).*((-1) ...

+2.*phi+(-1).*phi.^2+(-3).*WW+2.*phi.*WW+phi.^2.*WW+(-4).*phi.*WW.^2+ ...

phi.^2.*WW.^2+(-1).*phi.^2.*WW.^3+WW.*YY+(-4).*phi.*WW.*YY+2.*phi.^2.* ...

WW.*YY+3.*WW.^2.*YY+2.*phi.*WW.^2.*YY+(-4).*phi.^2.*WW.^2.*YY+2.*phi.* ...

WW.^3.*YY+2.*phi.^2.*WW.^3.*YY+(-2).*phi.*ZZ+2.*phi.^2.*ZZ+3.*WW.*ZZ+( ...

-2).*phi.*WW.*ZZ+(-4).*phi.^2.*WW.*ZZ+WW.^2.*ZZ+4.*phi.*WW.^2.*ZZ+2.* ...

phi.^2.*WW.^2.*ZZ+(-1).*phi.^2.*YY.*ZZ+4.*phi.*WW.*YY.*ZZ+phi.^2.*WW.* ...

YY.*ZZ+(-3).*WW.^2.*YY.*ZZ+(-2).*phi.*WW.^2.*YY.*ZZ+phi.^2.*WW.^2.*YY.* ...

ZZ+(-1).*WW.^3.*YY.*ZZ+(-2).*phi.*WW.^3.*YY.*ZZ+(-1).*phi.^2.*WW.^3.* ...

YY.*ZZ)+(-1/2).*((1/4).*((-1)+phi+(-1).*phi.*WW+(-1).*phi.*YY+WW.*YY+ ...

phi.*WW.*YY).^(-2).*((-1)+phi+(-1).*phi.*WW+(-1).*phi.*ZZ+WW.*ZZ+phi.* ...

WW.*ZZ).^(-2).*((-1)+2.*phi+(-1).*phi.^2+(-3).*WW+2.*phi.*WW+phi.^2.*WW+ ...

(-4).*phi.*WW.^2+phi.^2.*WW.^2+(-1).*phi.^2.*WW.^3+WW.*YY+(-4).*phi.* ...

WW.*YY+2.*phi.^2.*WW.*YY+3.*WW.^2.*YY+2.*phi.*WW.^2.*YY+(-4).*phi.^2.* ...

WW.^2.*YY+2.*phi.*WW.^3.*YY+2.*phi.^2.*WW.^3.*YY+(-2).*phi.*ZZ+2.* ...

phi.^2.*ZZ+3.*WW.*ZZ+(-2).*phi.*WW.*ZZ+(-4).*phi.^2.*WW.*ZZ+WW.^2.*ZZ+ ...

4.*phi.*WW.^2.*ZZ+2.*phi.^2.*WW.^2.*ZZ+(-1).*phi.^2.*YY.*ZZ+4.*phi.*WW.* ...

YY.*ZZ+phi.^2.*WW.*YY.*ZZ+(-3).*WW.^2.*YY.*ZZ+(-2).*phi.*WW.^2.*YY.*ZZ+ ...

phi.^2.*WW.^2.*YY.*ZZ+(-1).*WW.^3.*YY.*ZZ+(-2).*phi.*WW.^3.*YY.*ZZ+(-1) ...

.*phi.^2.*WW.^3.*YY.*ZZ).^2+(-1).*(WW+(-1).*XX).^(-1).*((-1)+phi+(-1).* ...

phi.*WW+(-1).*phi.*YY+WW.*YY+phi.*WW.*YY).^(-1).*((-1)+phi+(-1).*phi.* ...

WW+(-1).*phi.*ZZ+WW.*ZZ+phi.*WW.*ZZ).^(-1).*(3.*WW.^2+(-4).*phi.*WW.^2+ ...

phi.^2.*WW.^2+3.*WW.^3+2.*phi.*WW.^3+(-2).*phi.^2.*WW.^3+2.*phi.*WW.^4+ ...

phi.^2.*WW.^4+(-3).*WW.*XX+4.*phi.*WW.*XX+(-1).*phi.^2.*WW.*XX+(-3).* ...

WW.^2.*XX+(-2).*phi.*WW.^2.*XX+2.*phi.^2.*WW.^2.*XX+(-2).*phi.*WW.^3.* ...

XX+(-1).*phi.^2.*WW.^3.*XX+(-1).*phi.*WW.*YY+phi.^2.*WW.*YY+WW.^2.*YY+ ...

2.*phi.*WW.^2.*YY+(-4).*phi.^2.*WW.^2.*YY+(-5).*WW.^3.*YY+5.*phi.* ...

WW.^3.*YY+5.*phi.^2.*WW.^3.*YY+(-2).*WW.^4.*YY+(-6).*phi.*WW.^4.*YY+(-2) ...

.*phi.^2.*WW.^4.*YY+3.*WW.^2.*XX.*YY+(-5).*phi.*WW.^2.*XX.*YY+phi.^2.* ...

WW.^2.*XX.*YY+3.*WW.^3.*XX.*YY+4.*phi.*WW.^3.*XX.*YY+(-2).*phi.^2.* ...

WW.^3.*XX.*YY+phi.*WW.^4.*XX.*YY+phi.^2.*WW.^4.*XX.*YY+phi.*WW.*ZZ+(-1) ...

.*phi.^2.*WW.*ZZ+(-3).*WW.^2.*ZZ+4.*phi.*WW.^2.*ZZ+2.*phi.^2.*WW.^2.*ZZ+ ...

(-3).*WW.^3.*ZZ+(-5).*phi.*WW.^3.*ZZ+(-1).*phi.^2.*WW.^3.*ZZ+2.*WW.*XX.* ...

ZZ+(-6).*phi.*WW.*XX.*ZZ+2.*phi.^2.*WW.*XX.*ZZ+5.*WW.^2.*XX.*ZZ+5.*phi.* ...

WW.^2.*XX.*ZZ+(-5).*phi.^2.*WW.^2.*XX.*ZZ+(-1).*WW.^3.*XX.*ZZ+2.*phi.* ...

WW.^3.*XX.*ZZ+4.*phi.^2.*WW.^3.*XX.*ZZ+(-1).*phi.*WW.^4.*XX.*ZZ+(-1).* ...

phi.^2.*WW.^4.*XX.*ZZ+(-2).*phi.*WW.^2.*YY.*ZZ+phi.^2.*WW.^2.*YY.*ZZ+3.* ...

WW.^3.*YY.*ZZ+(-2).*phi.*WW.^3.*YY.*ZZ+(-2).*phi.^2.*WW.^3.*YY.*ZZ+3.* ...

WW.^4.*YY.*ZZ+4.*phi.*WW.^4.*YY.*ZZ+phi.^2.*WW.^4.*YY.*ZZ+2.*phi.*WW.* ...

XX.*YY.*ZZ+(-1).*phi.^2.*WW.*XX.*YY.*ZZ+(-3).*WW.^2.*XX.*YY.*ZZ+2.*phi.* ...

WW.^2.*XX.*YY.*ZZ+2.*phi.^2.*WW.^2.*XX.*YY.*ZZ+(-3).*WW.^3.*XX.*YY.*ZZ+( ...

-4).*phi.*WW.^3.*XX.*YY.*ZZ+(-1).*phi.^2.*WW.^3.*XX.*YY.*ZZ)+(1/3).*(WW+ ...

(-2).*phi.*WW+phi.^2.*WW+2.*phi.*WW.^2+(-2).*phi.^2.*WW.^2+phi.^2.* ...

WW.^3+(-1).*XX+2.*phi.*XX+(-1).*phi.^2.*XX+(-2).*phi.*WW.*XX+2.*phi.^2.* ...

WW.*XX+(-1).*phi.^2.*WW.^2.*XX+phi.*WW.*YY+(-1).*phi.^2.*WW.*YY+(-1).* ...

WW.^2.*YY+2.*phi.^2.*WW.^2.*YY+(-1).*phi.*WW.^3.*YY+(-1).*phi.^2.* ...

WW.^3.*YY+(-1).*phi.*XX.*YY+phi.^2.*XX.*YY+WW.*XX.*YY+(-2).*phi.^2.*WW.* ...

XX.*YY+phi.*WW.^2.*XX.*YY+phi.^2.*WW.^2.*XX.*YY+phi.*WW.*ZZ+(-1).* ...

phi.^2.*WW.*ZZ+(-1).*WW.^2.*ZZ+2.*phi.^2.*WW.^2.*ZZ+(-1).*phi.*WW.^3.* ...

ZZ+(-1).*phi.^2.*WW.^3.*ZZ+(-1).*phi.*XX.*ZZ+phi.^2.*XX.*ZZ+WW.*XX.*ZZ+( ...

-2).*phi.^2.*WW.*XX.*ZZ+phi.*WW.^2.*XX.*ZZ+phi.^2.*WW.^2.*XX.*ZZ+ ...

phi.^2.*WW.*YY.*ZZ+(-2).*phi.*WW.^2.*YY.*ZZ+(-2).*phi.^2.*WW.^2.*YY.*ZZ+ ...

WW.^3.*YY.*ZZ+2.*phi.*WW.^3.*YY.*ZZ+phi.^2.*WW.^3.*YY.*ZZ+(-1).*phi.^2.* ...

XX.*YY.*ZZ+2.*phi.*WW.*XX.*YY.*ZZ+2.*phi.^2.*WW.*XX.*YY.*ZZ+(-1).* ...

WW.^2.*XX.*YY.*ZZ+(-2).*phi.*WW.^2.*XX.*YY.*ZZ+(-1).*phi.^2.*WW.^2.*XX.* ...

YY.*ZZ).^(-1).*(3.*WW.^2+(-4).*phi.*WW.^2+phi.^2.*WW.^2+3.*WW.^3+2.* ...

phi.*WW.^3+(-2).*phi.^2.*WW.^3+2.*phi.*WW.^4+phi.^2.*WW.^4+(-3).*WW.*XX+ ...

4.*phi.*WW.*XX+(-1).*phi.^2.*WW.*XX+(-3).*WW.^2.*XX+(-2).*phi.*WW.^2.* ...

XX+2.*phi.^2.*WW.^2.*XX+(-2).*phi.*WW.^3.*XX+(-1).*phi.^2.*WW.^3.*XX+( ...

-1).*phi.*WW.*YY+phi.^2.*WW.*YY+WW.^2.*YY+2.*phi.*WW.^2.*YY+(-4).* ...

phi.^2.*WW.^2.*YY+(-5).*WW.^3.*YY+5.*phi.*WW.^3.*YY+5.*phi.^2.*WW.^3.* ...

YY+(-2).*WW.^4.*YY+(-6).*phi.*WW.^4.*YY+(-2).*phi.^2.*WW.^4.*YY+3.* ...

WW.^2.*XX.*YY+(-5).*phi.*WW.^2.*XX.*YY+phi.^2.*WW.^2.*XX.*YY+3.*WW.^3.* ...

XX.*YY+4.*phi.*WW.^3.*XX.*YY+(-2).*phi.^2.*WW.^3.*XX.*YY+phi.*WW.^4.* ...

XX.*YY+phi.^2.*WW.^4.*XX.*YY+phi.*WW.*ZZ+(-1).*phi.^2.*WW.*ZZ+(-3).* ...

WW.^2.*ZZ+4.*phi.*WW.^2.*ZZ+2.*phi.^2.*WW.^2.*ZZ+(-3).*WW.^3.*ZZ+(-5).* ...

phi.*WW.^3.*ZZ+(-1).*phi.^2.*WW.^3.*ZZ+2.*WW.*XX.*ZZ+(-6).*phi.*WW.*XX.* ...

ZZ+2.*phi.^2.*WW.*XX.*ZZ+5.*WW.^2.*XX.*ZZ+5.*phi.*WW.^2.*XX.*ZZ+(-5).* ...

phi.^2.*WW.^2.*XX.*ZZ+(-1).*WW.^3.*XX.*ZZ+2.*phi.*WW.^3.*XX.*ZZ+4.* ...

phi.^2.*WW.^3.*XX.*ZZ+(-1).*phi.*WW.^4.*XX.*ZZ+(-1).*phi.^2.*WW.^4.*XX.* ...

ZZ+(-2).*phi.*WW.^2.*YY.*ZZ+phi.^2.*WW.^2.*YY.*ZZ+3.*WW.^3.*YY.*ZZ+(-2) ...

.*phi.*WW.^3.*YY.*ZZ+(-2).*phi.^2.*WW.^3.*YY.*ZZ+3.*WW.^4.*YY.*ZZ+4.* ...

phi.*WW.^4.*YY.*ZZ+phi.^2.*WW.^4.*YY.*ZZ+2.*phi.*WW.*XX.*YY.*ZZ+(-1).* ...

phi.^2.*WW.*XX.*YY.*ZZ+(-3).*WW.^2.*XX.*YY.*ZZ+2.*phi.*WW.^2.*XX.*YY.* ...

ZZ+2.*phi.^2.*WW.^2.*XX.*YY.*ZZ+(-3).*WW.^3.*XX.*YY.*ZZ+(-4).*phi.* ...

WW.^3.*XX.*YY.*ZZ+(-1).*phi.^2.*WW.^3.*XX.*YY.*ZZ)+(1/3).*2.^(1/3).*(WW+ ...

(-1).*XX).^(-1).*((-1)+phi+(-1).*phi.*WW+(-1).*phi.*YY+WW.*YY+phi.*WW.* ...

YY).^(-1).*((-1)+phi+(-1).*phi.*WW+(-1).*phi.*ZZ+WW.*ZZ+phi.*WW.*ZZ).^( ...

-1).*(12.*(WW+(-2).*phi.*WW+phi.^2.*WW+2.*phi.*WW.^2+(-2).*phi.^2.* ...

WW.^2+phi.^2.*WW.^3+(-1).*XX+2.*phi.*XX+(-1).*phi.^2.*XX+(-2).*phi.*WW.* ...

XX+2.*phi.^2.*WW.*XX+(-1).*phi.^2.*WW.^2.*XX+phi.*WW.*YY+(-1).*phi.^2.* ...

WW.*YY+(-1).*WW.^2.*YY+2.*phi.^2.*WW.^2.*YY+(-1).*phi.*WW.^3.*YY+(-1).* ...

phi.^2.*WW.^3.*YY+(-1).*phi.*XX.*YY+phi.^2.*XX.*YY+WW.*XX.*YY+(-2).* ...

phi.^2.*WW.*XX.*YY+phi.*WW.^2.*XX.*YY+phi.^2.*WW.^2.*XX.*YY+phi.*WW.*ZZ+ ...

(-1).*phi.^2.*WW.*ZZ+(-1).*WW.^2.*ZZ+2.*phi.^2.*WW.^2.*ZZ+(-1).*phi.* ...

WW.^3.*ZZ+(-1).*phi.^2.*WW.^3.*ZZ+(-1).*phi.*XX.*ZZ+phi.^2.*XX.*ZZ+WW.* ...

XX.*ZZ+(-2).*phi.^2.*WW.*XX.*ZZ+phi.*WW.^2.*XX.*ZZ+phi.^2.*WW.^2.*XX.* ...

ZZ+phi.^2.*WW.*YY.*ZZ+(-2).*phi.*WW.^2.*YY.*ZZ+(-2).*phi.^2.*WW.^2.*YY.* ...

ZZ+WW.^3.*YY.*ZZ+2.*phi.*WW.^3.*YY.*ZZ+phi.^2.*WW.^3.*YY.*ZZ+(-1).* ...

phi.^2.*XX.*YY.*ZZ+2.*phi.*WW.*XX.*YY.*ZZ+2.*phi.^2.*WW.*XX.*YY.*ZZ+(-1) ...

.*WW.^2.*XX.*YY.*ZZ+(-2).*phi.*WW.^2.*XX.*YY.*ZZ+(-1).*phi.^2.*WW.^2.* ...

XX.*YY.*ZZ).*(WW.^4+(-1).*WW.^3.*XX+WW.^3.*YY+(-2).*WW.^4.*YY+WW.^4.* ...

XX.*YY+(-1).*WW.^3.*ZZ+2.*WW.^3.*XX.*ZZ+(-1).*WW.^4.*XX.*ZZ+WW.^4.*YY.* ...

ZZ+(-1).*WW.^3.*XX.*YY.*ZZ)+(3.*WW.^2+(-4).*phi.*WW.^2+phi.^2.*WW.^2+3.* ...

WW.^3+2.*phi.*WW.^3+(-2).*phi.^2.*WW.^3+2.*phi.*WW.^4+phi.^2.*WW.^4+(-3) ...

.*WW.*XX+4.*phi.*WW.*XX+(-1).*phi.^2.*WW.*XX+(-3).*WW.^2.*XX+(-2).*phi.* ...

WW.^2.*XX+2.*phi.^2.*WW.^2.*XX+(-2).*phi.*WW.^3.*XX+(-1).*phi.^2.* ...

WW.^3.*XX+(-1).*phi.*WW.*YY+phi.^2.*WW.*YY+WW.^2.*YY+2.*phi.*WW.^2.*YY+( ...

-4).*phi.^2.*WW.^2.*YY+(-5).*WW.^3.*YY+5.*phi.*WW.^3.*YY+5.*phi.^2.* ...

WW.^3.*YY+(-2).*WW.^4.*YY+(-6).*phi.*WW.^4.*YY+(-2).*phi.^2.*WW.^4.*YY+ ...

3.*WW.^2.*XX.*YY+(-5).*phi.*WW.^2.*XX.*YY+phi.^2.*WW.^2.*XX.*YY+3.* ...

WW.^3.*XX.*YY+4.*phi.*WW.^3.*XX.*YY+(-2).*phi.^2.*WW.^3.*XX.*YY+phi.* ...

WW.^4.*XX.*YY+phi.^2.*WW.^4.*XX.*YY+phi.*WW.*ZZ+(-1).*phi.^2.*WW.*ZZ+( ...

-3).*WW.^2.*ZZ+4.*phi.*WW.^2.*ZZ+2.*phi.^2.*WW.^2.*ZZ+(-3).*WW.^3.*ZZ+( ...

-5).*phi.*WW.^3.*ZZ+(-1).*phi.^2.*WW.^3.*ZZ+2.*WW.*XX.*ZZ+(-6).*phi.* ...

WW.*XX.*ZZ+2.*phi.^2.*WW.*XX.*ZZ+5.*WW.^2.*XX.*ZZ+5.*phi.*WW.^2.*XX.*ZZ+ ...

(-5).*phi.^2.*WW.^2.*XX.*ZZ+(-1).*WW.^3.*XX.*ZZ+2.*phi.*WW.^3.*XX.*ZZ+ ...

4.*phi.^2.*WW.^3.*XX.*ZZ+(-1).*phi.*WW.^4.*XX.*ZZ+(-1).*phi.^2.*WW.^4.* ...

XX.*ZZ+(-2).*phi.*WW.^2.*YY.*ZZ+phi.^2.*WW.^2.*YY.*ZZ+3.*WW.^3.*YY.*ZZ+( ...

-2).*phi.*WW.^3.*YY.*ZZ+(-2).*phi.^2.*WW.^3.*YY.*ZZ+3.*WW.^4.*YY.*ZZ+4.* ...

phi.*WW.^4.*YY.*ZZ+phi.^2.*WW.^4.*YY.*ZZ+2.*phi.*WW.*XX.*YY.*ZZ+(-1).* ...

phi.^2.*WW.*XX.*YY.*ZZ+(-3).*WW.^2.*XX.*YY.*ZZ+2.*phi.*WW.^2.*XX.*YY.* ...

ZZ+2.*phi.^2.*WW.^2.*XX.*YY.*ZZ+(-3).*WW.^3.*XX.*YY.*ZZ+(-4).*phi.* ...

WW.^3.*XX.*YY.*ZZ+(-1).*phi.^2.*WW.^3.*XX.*YY.*ZZ).^2+(-3).*((-3).* ...

WW.^3+2.*phi.*WW.^3+(-1).*WW.^4+(-2).*phi.*WW.^4+3.*WW.^2.*XX+(-2).* ...

phi.*WW.^2.*XX+WW.^3.*XX+2.*phi.*WW.^3.*XX+(-1).*WW.^2.*YY+2.*phi.* ...

WW.^2.*YY+WW.^3.*YY+(-6).*phi.*WW.^3.*YY+4.*WW.^4.*YY+4.*phi.*WW.^4.*YY+ ...

(-3).*WW.^3.*XX.*YY+2.*phi.*WW.^3.*XX.*YY+(-1).*WW.^4.*XX.*YY+(-2).* ...

phi.*WW.^4.*XX.*YY+WW.^2.*ZZ+(-2).*phi.*WW.^2.*ZZ+3.*WW.^3.*ZZ+2.*phi.* ...

WW.^3.*ZZ+(-4).*WW.^2.*XX.*ZZ+4.*phi.*WW.^2.*XX.*ZZ+(-1).*WW.^3.*XX.*ZZ+ ...

(-6).*phi.*WW.^3.*XX.*ZZ+WW.^4.*XX.*ZZ+2.*phi.*WW.^4.*XX.*ZZ+(-1).* ...

WW.^3.*YY.*ZZ+2.*phi.*WW.^3.*YY.*ZZ+(-3).*WW.^4.*YY.*ZZ+(-2).*phi.* ...

WW.^4.*YY.*ZZ+WW.^2.*XX.*YY.*ZZ+(-2).*phi.*WW.^2.*XX.*YY.*ZZ+3.*WW.^3.* ...

XX.*YY.*ZZ+2.*phi.*WW.^3.*XX.*YY.*ZZ).*((-1).*WW+2.*phi.*WW+(-1).* ...

phi.^2.*WW+(-3).*WW.^2+2.*phi.*WW.^2+phi.^2.*WW.^2+(-4).*phi.*WW.^3+ ...

phi.^2.*WW.^3+(-1).*phi.^2.*WW.^4+XX+(-2).*phi.*XX+phi.^2.*XX+3.*WW.*XX+ ...

(-2).*phi.*WW.*XX+(-1).*phi.^2.*WW.*XX+4.*phi.*WW.^2.*XX+(-1).*phi.^2.* ...

WW.^2.*XX+phi.^2.*WW.^3.*XX+WW.^2.*YY+(-4).*phi.*WW.^2.*YY+2.*phi.^2.* ...

WW.^2.*YY+3.*WW.^3.*YY+2.*phi.*WW.^3.*YY+(-4).*phi.^2.*WW.^3.*YY+2.* ...

phi.*WW.^4.*YY+2.*phi.^2.*WW.^4.*YY+(-1).*WW.*XX.*YY+4.*phi.*WW.*XX.*YY+ ...

(-2).*phi.^2.*WW.*XX.*YY+(-3).*WW.^2.*XX.*YY+(-2).*phi.*WW.^2.*XX.*YY+ ...

4.*phi.^2.*WW.^2.*XX.*YY+(-2).*phi.*WW.^3.*XX.*YY+(-2).*phi.^2.*WW.^3.* ...

XX.*YY+(-2).*phi.*WW.*ZZ+2.*phi.^2.*WW.*ZZ+3.*WW.^2.*ZZ+(-2).*phi.* ...

WW.^2.*ZZ+(-4).*phi.^2.*WW.^2.*ZZ+WW.^3.*ZZ+4.*phi.*WW.^3.*ZZ+2.* ...

phi.^2.*WW.^3.*ZZ+2.*phi.*XX.*ZZ+(-2).*phi.^2.*XX.*ZZ+(-3).*WW.*XX.*ZZ+ ...

2.*phi.*WW.*XX.*ZZ+4.*phi.^2.*WW.*XX.*ZZ+(-1).*WW.^2.*XX.*ZZ+(-4).*phi.* ...

WW.^2.*XX.*ZZ+(-2).*phi.^2.*WW.^2.*XX.*ZZ+(-1).*phi.^2.*WW.*YY.*ZZ+4.* ...

phi.*WW.^2.*YY.*ZZ+phi.^2.*WW.^2.*YY.*ZZ+(-3).*WW.^3.*YY.*ZZ+(-2).*phi.* ...

WW.^3.*YY.*ZZ+phi.^2.*WW.^3.*YY.*ZZ+(-1).*WW.^4.*YY.*ZZ+(-2).*phi.* ...

WW.^4.*YY.*ZZ+(-1).*phi.^2.*WW.^4.*YY.*ZZ+phi.^2.*XX.*YY.*ZZ+(-4).*phi.* ...

WW.*XX.*YY.*ZZ+(-1).*phi.^2.*WW.*XX.*YY.*ZZ+3.*WW.^2.*XX.*YY.*ZZ+2.* ...

phi.*WW.^2.*XX.*YY.*ZZ+(-1).*phi.^2.*WW.^2.*XX.*YY.*ZZ+WW.^3.*XX.*YY.* ...

ZZ+2.*phi.*WW.^3.*XX.*YY.*ZZ+phi.^2.*WW.^3.*XX.*YY.*ZZ)).*(27.*(WW+(-2) ...

.*phi.*WW+phi.^2.*WW+2.*phi.*WW.^2+(-2).*phi.^2.*WW.^2+phi.^2.*WW.^3+( ...

-1).*XX+2.*phi.*XX+(-1).*phi.^2.*XX+(-2).*phi.*WW.*XX+2.*phi.^2.*WW.*XX+ ...

(-1).*phi.^2.*WW.^2.*XX+phi.*WW.*YY+(-1).*phi.^2.*WW.*YY+(-1).*WW.^2.* ...

YY+2.*phi.^2.*WW.^2.*YY+(-1).*phi.*WW.^3.*YY+(-1).*phi.^2.*WW.^3.*YY+( ...

-1).*phi.*XX.*YY+phi.^2.*XX.*YY+WW.*XX.*YY+(-2).*phi.^2.*WW.*XX.*YY+ ...

phi.*WW.^2.*XX.*YY+phi.^2.*WW.^2.*XX.*YY+phi.*WW.*ZZ+(-1).*phi.^2.*WW.* ...

ZZ+(-1).*WW.^2.*ZZ+2.*phi.^2.*WW.^2.*ZZ+(-1).*phi.*WW.^3.*ZZ+(-1).* ...

phi.^2.*WW.^3.*ZZ+(-1).*phi.*XX.*ZZ+phi.^2.*XX.*ZZ+WW.*XX.*ZZ+(-2).* ...

phi.^2.*WW.*XX.*ZZ+phi.*WW.^2.*XX.*ZZ+phi.^2.*WW.^2.*XX.*ZZ+phi.^2.*WW.* ...

YY.*ZZ+(-2).*phi.*WW.^2.*YY.*ZZ+(-2).*phi.^2.*WW.^2.*YY.*ZZ+WW.^3.*YY.* ...

ZZ+2.*phi.*WW.^3.*YY.*ZZ+phi.^2.*WW.^3.*YY.*ZZ+(-1).*phi.^2.*XX.*YY.*ZZ+ ...

2.*phi.*WW.*XX.*YY.*ZZ+2.*phi.^2.*WW.*XX.*YY.*ZZ+(-1).*WW.^2.*XX.*YY.* ...

ZZ+(-2).*phi.*WW.^2.*XX.*YY.*ZZ+(-1).*phi.^2.*WW.^2.*XX.*YY.*ZZ).*((-3) ...

.*WW.^3+2.*phi.*WW.^3+(-1).*WW.^4+(-2).*phi.*WW.^4+3.*WW.^2.*XX+(-2).* ...

phi.*WW.^2.*XX+WW.^3.*XX+2.*phi.*WW.^3.*XX+(-1).*WW.^2.*YY+2.*phi.* ...

WW.^2.*YY+WW.^3.*YY+(-6).*phi.*WW.^3.*YY+4.*WW.^4.*YY+4.*phi.*WW.^4.*YY+ ...

(-3).*WW.^3.*XX.*YY+2.*phi.*WW.^3.*XX.*YY+(-1).*WW.^4.*XX.*YY+(-2).* ...

phi.*WW.^4.*XX.*YY+WW.^2.*ZZ+(-2).*phi.*WW.^2.*ZZ+3.*WW.^3.*ZZ+2.*phi.* ...

WW.^3.*ZZ+(-4).*WW.^2.*XX.*ZZ+4.*phi.*WW.^2.*XX.*ZZ+(-1).*WW.^3.*XX.*ZZ+ ...

(-6).*phi.*WW.^3.*XX.*ZZ+WW.^4.*XX.*ZZ+2.*phi.*WW.^4.*XX.*ZZ+(-1).* ...

WW.^3.*YY.*ZZ+2.*phi.*WW.^3.*YY.*ZZ+(-3).*WW.^4.*YY.*ZZ+(-2).*phi.* ...

WW.^4.*YY.*ZZ+WW.^2.*XX.*YY.*ZZ+(-2).*phi.*WW.^2.*XX.*YY.*ZZ+3.*WW.^3.* ...

XX.*YY.*ZZ+2.*phi.*WW.^3.*XX.*YY.*ZZ).^2+(-72).*(WW+(-2).*phi.*WW+ ...

phi.^2.*WW+2.*phi.*WW.^2+(-2).*phi.^2.*WW.^2+phi.^2.*WW.^3+(-1).*XX+2.* ...

phi.*XX+(-1).*phi.^2.*XX+(-2).*phi.*WW.*XX+2.*phi.^2.*WW.*XX+(-1).* ...

phi.^2.*WW.^2.*XX+phi.*WW.*YY+(-1).*phi.^2.*WW.*YY+(-1).*WW.^2.*YY+2.* ...

phi.^2.*WW.^2.*YY+(-1).*phi.*WW.^3.*YY+(-1).*phi.^2.*WW.^3.*YY+(-1).* ...

phi.*XX.*YY+phi.^2.*XX.*YY+WW.*XX.*YY+(-2).*phi.^2.*WW.*XX.*YY+phi.* ...

WW.^2.*XX.*YY+phi.^2.*WW.^2.*XX.*YY+phi.*WW.*ZZ+(-1).*phi.^2.*WW.*ZZ+( ...

-1).*WW.^2.*ZZ+2.*phi.^2.*WW.^2.*ZZ+(-1).*phi.*WW.^3.*ZZ+(-1).*phi.^2.* ...

WW.^3.*ZZ+(-1).*phi.*XX.*ZZ+phi.^2.*XX.*ZZ+WW.*XX.*ZZ+(-2).*phi.^2.*WW.* ...

XX.*ZZ+phi.*WW.^2.*XX.*ZZ+phi.^2.*WW.^2.*XX.*ZZ+phi.^2.*WW.*YY.*ZZ+(-2) ...

.*phi.*WW.^2.*YY.*ZZ+(-2).*phi.^2.*WW.^2.*YY.*ZZ+WW.^3.*YY.*ZZ+2.*phi.* ...

WW.^3.*YY.*ZZ+phi.^2.*WW.^3.*YY.*ZZ+(-1).*phi.^2.*XX.*YY.*ZZ+2.*phi.* ...

WW.*XX.*YY.*ZZ+2.*phi.^2.*WW.*XX.*YY.*ZZ+(-1).*WW.^2.*XX.*YY.*ZZ+(-2).* ...

phi.*WW.^2.*XX.*YY.*ZZ+(-1).*phi.^2.*WW.^2.*XX.*YY.*ZZ).*(WW.^4+(-1).* ...

WW.^3.*XX+WW.^3.*YY+(-2).*WW.^4.*YY+WW.^4.*XX.*YY+(-1).*WW.^3.*ZZ+2.* ...

WW.^3.*XX.*ZZ+(-1).*WW.^4.*XX.*ZZ+WW.^4.*YY.*ZZ+(-1).*WW.^3.*XX.*YY.*ZZ) ...

.*(3.*WW.^2+(-4).*phi.*WW.^2+phi.^2.*WW.^2+3.*WW.^3+2.*phi.*WW.^3+(-2).* ...

phi.^2.*WW.^3+2.*phi.*WW.^4+phi.^2.*WW.^4+(-3).*WW.*XX+4.*phi.*WW.*XX+( ...

-1).*phi.^2.*WW.*XX+(-3).*WW.^2.*XX+(-2).*phi.*WW.^2.*XX+2.*phi.^2.* ...

WW.^2.*XX+(-2).*phi.*WW.^3.*XX+(-1).*phi.^2.*WW.^3.*XX+(-1).*phi.*WW.* ...

YY+phi.^2.*WW.*YY+WW.^2.*YY+2.*phi.*WW.^2.*YY+(-4).*phi.^2.*WW.^2.*YY+( ...

-5).*WW.^3.*YY+5.*phi.*WW.^3.*YY+5.*phi.^2.*WW.^3.*YY+(-2).*WW.^4.*YY+( ...

-6).*phi.*WW.^4.*YY+(-2).*phi.^2.*WW.^4.*YY+3.*WW.^2.*XX.*YY+(-5).*phi.* ...

WW.^2.*XX.*YY+phi.^2.*WW.^2.*XX.*YY+3.*WW.^3.*XX.*YY+4.*phi.*WW.^3.*XX.* ...

YY+(-2).*phi.^2.*WW.^3.*XX.*YY+phi.*WW.^4.*XX.*YY+phi.^2.*WW.^4.*XX.*YY+ ...

phi.*WW.*ZZ+(-1).*phi.^2.*WW.*ZZ+(-3).*WW.^2.*ZZ+4.*phi.*WW.^2.*ZZ+2.* ...

phi.^2.*WW.^2.*ZZ+(-3).*WW.^3.*ZZ+(-5).*phi.*WW.^3.*ZZ+(-1).*phi.^2.* ...

WW.^3.*ZZ+2.*WW.*XX.*ZZ+(-6).*phi.*WW.*XX.*ZZ+2.*phi.^2.*WW.*XX.*ZZ+5.* ...

WW.^2.*XX.*ZZ+5.*phi.*WW.^2.*XX.*ZZ+(-5).*phi.^2.*WW.^2.*XX.*ZZ+(-1).* ...

WW.^3.*XX.*ZZ+2.*phi.*WW.^3.*XX.*ZZ+4.*phi.^2.*WW.^3.*XX.*ZZ+(-1).*phi.* ...

WW.^4.*XX.*ZZ+(-1).*phi.^2.*WW.^4.*XX.*ZZ+(-2).*phi.*WW.^2.*YY.*ZZ+ ...

phi.^2.*WW.^2.*YY.*ZZ+3.*WW.^3.*YY.*ZZ+(-2).*phi.*WW.^3.*YY.*ZZ+(-2).* ...

phi.^2.*WW.^3.*YY.*ZZ+3.*WW.^4.*YY.*ZZ+4.*phi.*WW.^4.*YY.*ZZ+phi.^2.* ...

WW.^4.*YY.*ZZ+2.*phi.*WW.*XX.*YY.*ZZ+(-1).*phi.^2.*WW.*XX.*YY.*ZZ+(-3).* ...

WW.^2.*XX.*YY.*ZZ+2.*phi.*WW.^2.*XX.*YY.*ZZ+2.*phi.^2.*WW.^2.*XX.*YY.* ...

ZZ+(-3).*WW.^3.*XX.*YY.*ZZ+(-4).*phi.*WW.^3.*XX.*YY.*ZZ+(-1).*phi.^2.* ...

WW.^3.*XX.*YY.*ZZ)+2.*(3.*WW.^2+(-4).*phi.*WW.^2+phi.^2.*WW.^2+3.*WW.^3+ ...

2.*phi.*WW.^3+(-2).*phi.^2.*WW.^3+2.*phi.*WW.^4+phi.^2.*WW.^4+(-3).*WW.* ...

XX+4.*phi.*WW.*XX+(-1).*phi.^2.*WW.*XX+(-3).*WW.^2.*XX+(-2).*phi.* ...

WW.^2.*XX+2.*phi.^2.*WW.^2.*XX+(-2).*phi.*WW.^3.*XX+(-1).*phi.^2.* ...

WW.^3.*XX+(-1).*phi.*WW.*YY+phi.^2.*WW.*YY+WW.^2.*YY+2.*phi.*WW.^2.*YY+( ...

-4).*phi.^2.*WW.^2.*YY+(-5).*WW.^3.*YY+5.*phi.*WW.^3.*YY+5.*phi.^2.* ...

WW.^3.*YY+(-2).*WW.^4.*YY+(-6).*phi.*WW.^4.*YY+(-2).*phi.^2.*WW.^4.*YY+ ...

3.*WW.^2.*XX.*YY+(-5).*phi.*WW.^2.*XX.*YY+phi.^2.*WW.^2.*XX.*YY+3.* ...

WW.^3.*XX.*YY+4.*phi.*WW.^3.*XX.*YY+(-2).*phi.^2.*WW.^3.*XX.*YY+phi.* ...

WW.^4.*XX.*YY+phi.^2.*WW.^4.*XX.*YY+phi.*WW.*ZZ+(-1).*phi.^2.*WW.*ZZ+( ...

-3).*WW.^2.*ZZ+4.*phi.*WW.^2.*ZZ+2.*phi.^2.*WW.^2.*ZZ+(-3).*WW.^3.*ZZ+( ...

-5).*phi.*WW.^3.*ZZ+(-1).*phi.^2.*WW.^3.*ZZ+2.*WW.*XX.*ZZ+(-6).*phi.* ...

WW.*XX.*ZZ+2.*phi.^2.*WW.*XX.*ZZ+5.*WW.^2.*XX.*ZZ+5.*phi.*WW.^2.*XX.*ZZ+ ...

(-5).*phi.^2.*WW.^2.*XX.*ZZ+(-1).*WW.^3.*XX.*ZZ+2.*phi.*WW.^3.*XX.*ZZ+ ...

4.*phi.^2.*WW.^3.*XX.*ZZ+(-1).*phi.*WW.^4.*XX.*ZZ+(-1).*phi.^2.*WW.^4.* ...

XX.*ZZ+(-2).*phi.*WW.^2.*YY.*ZZ+phi.^2.*WW.^2.*YY.*ZZ+3.*WW.^3.*YY.*ZZ+( ...

-2).*phi.*WW.^3.*YY.*ZZ+(-2).*phi.^2.*WW.^3.*YY.*ZZ+3.*WW.^4.*YY.*ZZ+4.* ...

phi.*WW.^4.*YY.*ZZ+phi.^2.*WW.^4.*YY.*ZZ+2.*phi.*WW.*XX.*YY.*ZZ+(-1).* ...

phi.^2.*WW.*XX.*YY.*ZZ+(-3).*WW.^2.*XX.*YY.*ZZ+2.*phi.*WW.^2.*XX.*YY.* ...

ZZ+2.*phi.^2.*WW.^2.*XX.*YY.*ZZ+(-3).*WW.^3.*XX.*YY.*ZZ+(-4).*phi.* ...

WW.^3.*XX.*YY.*ZZ+(-1).*phi.^2.*WW.^3.*XX.*YY.*ZZ).^3+(-9).*((-3).* ...

WW.^3+2.*phi.*WW.^3+(-1).*WW.^4+(-2).*phi.*WW.^4+3.*WW.^2.*XX+(-2).* ...

phi.*WW.^2.*XX+WW.^3.*XX+2.*phi.*WW.^3.*XX+(-1).*WW.^2.*YY+2.*phi.* ...

WW.^2.*YY+WW.^3.*YY+(-6).*phi.*WW.^3.*YY+4.*WW.^4.*YY+4.*phi.*WW.^4.*YY+ ...

(-3).*WW.^3.*XX.*YY+2.*phi.*WW.^3.*XX.*YY+(-1).*WW.^4.*XX.*YY+(-2).* ...

phi.*WW.^4.*XX.*YY+WW.^2.*ZZ+(-2).*phi.*WW.^2.*ZZ+3.*WW.^3.*ZZ+2.*phi.* ...

WW.^3.*ZZ+(-4).*WW.^2.*XX.*ZZ+4.*phi.*WW.^2.*XX.*ZZ+(-1).*WW.^3.*XX.*ZZ+ ...

(-6).*phi.*WW.^3.*XX.*ZZ+WW.^4.*XX.*ZZ+2.*phi.*WW.^4.*XX.*ZZ+(-1).* ...

WW.^3.*YY.*ZZ+2.*phi.*WW.^3.*YY.*ZZ+(-3).*WW.^4.*YY.*ZZ+(-2).*phi.* ...

WW.^4.*YY.*ZZ+WW.^2.*XX.*YY.*ZZ+(-2).*phi.*WW.^2.*XX.*YY.*ZZ+3.*WW.^3.* ...

XX.*YY.*ZZ+2.*phi.*WW.^3.*XX.*YY.*ZZ).*(3.*WW.^2+(-4).*phi.*WW.^2+ ...

phi.^2.*WW.^2+3.*WW.^3+2.*phi.*WW.^3+(-2).*phi.^2.*WW.^3+2.*phi.*WW.^4+ ...

phi.^2.*WW.^4+(-3).*WW.*XX+4.*phi.*WW.*XX+(-1).*phi.^2.*WW.*XX+(-3).* ...

WW.^2.*XX+(-2).*phi.*WW.^2.*XX+2.*phi.^2.*WW.^2.*XX+(-2).*phi.*WW.^3.* ...

XX+(-1).*phi.^2.*WW.^3.*XX+(-1).*phi.*WW.*YY+phi.^2.*WW.*YY+WW.^2.*YY+ ...

2.*phi.*WW.^2.*YY+(-4).*phi.^2.*WW.^2.*YY+(-5).*WW.^3.*YY+5.*phi.* ...

WW.^3.*YY+5.*phi.^2.*WW.^3.*YY+(-2).*WW.^4.*YY+(-6).*phi.*WW.^4.*YY+(-2) ...

.*phi.^2.*WW.^4.*YY+3.*WW.^2.*XX.*YY+(-5).*phi.*WW.^2.*XX.*YY+phi.^2.* ...

WW.^2.*XX.*YY+3.*WW.^3.*XX.*YY+4.*phi.*WW.^3.*XX.*YY+(-2).*phi.^2.* ...

WW.^3.*XX.*YY+phi.*WW.^4.*XX.*YY+phi.^2.*WW.^4.*XX.*YY+phi.*WW.*ZZ+(-1) ...

.*phi.^2.*WW.*ZZ+(-3).*WW.^2.*ZZ+4.*phi.*WW.^2.*ZZ+2.*phi.^2.*WW.^2.*ZZ+ ...

(-3).*WW.^3.*ZZ+(-5).*phi.*WW.^3.*ZZ+(-1).*phi.^2.*WW.^3.*ZZ+2.*WW.*XX.* ...

ZZ+(-6).*phi.*WW.*XX.*ZZ+2.*phi.^2.*WW.*XX.*ZZ+5.*WW.^2.*XX.*ZZ+5.*phi.* ...

WW.^2.*XX.*ZZ+(-5).*phi.^2.*WW.^2.*XX.*ZZ+(-1).*WW.^3.*XX.*ZZ+2.*phi.* ...

WW.^3.*XX.*ZZ+4.*phi.^2.*WW.^3.*XX.*ZZ+(-1).*phi.*WW.^4.*XX.*ZZ+(-1).* ...

phi.^2.*WW.^4.*XX.*ZZ+(-2).*phi.*WW.^2.*YY.*ZZ+phi.^2.*WW.^2.*YY.*ZZ+3.* ...

WW.^3.*YY.*ZZ+(-2).*phi.*WW.^3.*YY.*ZZ+(-2).*phi.^2.*WW.^3.*YY.*ZZ+3.* ...

WW.^4.*YY.*ZZ+4.*phi.*WW.^4.*YY.*ZZ+phi.^2.*WW.^4.*YY.*ZZ+2.*phi.*WW.* ...

XX.*YY.*ZZ+(-1).*phi.^2.*WW.*XX.*YY.*ZZ+(-3).*WW.^2.*XX.*YY.*ZZ+2.*phi.* ...

WW.^2.*XX.*YY.*ZZ+2.*phi.^2.*WW.^2.*XX.*YY.*ZZ+(-3).*WW.^3.*XX.*YY.*ZZ+( ...

-4).*phi.*WW.^3.*XX.*YY.*ZZ+(-1).*phi.^2.*WW.^3.*XX.*YY.*ZZ).*((-1).*WW+ ...

2.*phi.*WW+(-1).*phi.^2.*WW+(-3).*WW.^2+2.*phi.*WW.^2+phi.^2.*WW.^2+(-4) ...

.*phi.*WW.^3+phi.^2.*WW.^3+(-1).*phi.^2.*WW.^4+XX+(-2).*phi.*XX+phi.^2.* ...

XX+3.*WW.*XX+(-2).*phi.*WW.*XX+(-1).*phi.^2.*WW.*XX+4.*phi.*WW.^2.*XX+( ...

-1).*phi.^2.*WW.^2.*XX+phi.^2.*WW.^3.*XX+WW.^2.*YY+(-4).*phi.*WW.^2.*YY+ ...

2.*phi.^2.*WW.^2.*YY+3.*WW.^3.*YY+2.*phi.*WW.^3.*YY+(-4).*phi.^2.* ...

WW.^3.*YY+2.*phi.*WW.^4.*YY+2.*phi.^2.*WW.^4.*YY+(-1).*WW.*XX.*YY+4.* ...

phi.*WW.*XX.*YY+(-2).*phi.^2.*WW.*XX.*YY+(-3).*WW.^2.*XX.*YY+(-2).*phi.* ...

WW.^2.*XX.*YY+4.*phi.^2.*WW.^2.*XX.*YY+(-2).*phi.*WW.^3.*XX.*YY+(-2).* ...

phi.^2.*WW.^3.*XX.*YY+(-2).*phi.*WW.*ZZ+2.*phi.^2.*WW.*ZZ+3.*WW.^2.*ZZ+( ...

-2).*phi.*WW.^2.*ZZ+(-4).*phi.^2.*WW.^2.*ZZ+WW.^3.*ZZ+4.*phi.*WW.^3.*ZZ+ ...

2.*phi.^2.*WW.^3.*ZZ+2.*phi.*XX.*ZZ+(-2).*phi.^2.*XX.*ZZ+(-3).*WW.*XX.* ...

ZZ+2.*phi.*WW.*XX.*ZZ+4.*phi.^2.*WW.*XX.*ZZ+(-1).*WW.^2.*XX.*ZZ+(-4).* ...

phi.*WW.^2.*XX.*ZZ+(-2).*phi.^2.*WW.^2.*XX.*ZZ+(-1).*phi.^2.*WW.*YY.*ZZ+ ...

4.*phi.*WW.^2.*YY.*ZZ+phi.^2.*WW.^2.*YY.*ZZ+(-3).*WW.^3.*YY.*ZZ+(-2).* ...

phi.*WW.^3.*YY.*ZZ+phi.^2.*WW.^3.*YY.*ZZ+(-1).*WW.^4.*YY.*ZZ+(-2).*phi.* ...

WW.^4.*YY.*ZZ+(-1).*phi.^2.*WW.^4.*YY.*ZZ+phi.^2.*XX.*YY.*ZZ+(-4).*phi.* ...

WW.*XX.*YY.*ZZ+(-1).*phi.^2.*WW.*XX.*YY.*ZZ+3.*WW.^2.*XX.*YY.*ZZ+2.* ...

phi.*WW.^2.*XX.*YY.*ZZ+(-1).*phi.^2.*WW.^2.*XX.*YY.*ZZ+WW.^3.*XX.*YY.* ...

ZZ+2.*phi.*WW.^3.*XX.*YY.*ZZ+phi.^2.*WW.^3.*XX.*YY.*ZZ)+27.*(WW.^4+(-1) ...

.*WW.^3.*XX+WW.^3.*YY+(-2).*WW.^4.*YY+WW.^4.*XX.*YY+(-1).*WW.^3.*ZZ+2.* ...

WW.^3.*XX.*ZZ+(-1).*WW.^4.*XX.*ZZ+WW.^4.*YY.*ZZ+(-1).*WW.^3.*XX.*YY.*ZZ) ...

.*((-1).*WW+2.*phi.*WW+(-1).*phi.^2.*WW+(-3).*WW.^2+2.*phi.*WW.^2+ ...

phi.^2.*WW.^2+(-4).*phi.*WW.^3+phi.^2.*WW.^3+(-1).*phi.^2.*WW.^4+XX+(-2) ...

.*phi.*XX+phi.^2.*XX+3.*WW.*XX+(-2).*phi.*WW.*XX+(-1).*phi.^2.*WW.*XX+ ...

4.*phi.*WW.^2.*XX+(-1).*phi.^2.*WW.^2.*XX+phi.^2.*WW.^3.*XX+WW.^2.*YY+( ...

-4).*phi.*WW.^2.*YY+2.*phi.^2.*WW.^2.*YY+3.*WW.^3.*YY+2.*phi.*WW.^3.*YY+ ...

(-4).*phi.^2.*WW.^3.*YY+2.*phi.*WW.^4.*YY+2.*phi.^2.*WW.^4.*YY+(-1).* ...

WW.*XX.*YY+4.*phi.*WW.*XX.*YY+(-2).*phi.^2.*WW.*XX.*YY+(-3).*WW.^2.*XX.* ...

YY+(-2).*phi.*WW.^2.*XX.*YY+4.*phi.^2.*WW.^2.*XX.*YY+(-2).*phi.*WW.^3.* ...

XX.*YY+(-2).*phi.^2.*WW.^3.*XX.*YY+(-2).*phi.*WW.*ZZ+2.*phi.^2.*WW.*ZZ+ ...

3.*WW.^2.*ZZ+(-2).*phi.*WW.^2.*ZZ+(-4).*phi.^2.*WW.^2.*ZZ+WW.^3.*ZZ+4.* ...

phi.*WW.^3.*ZZ+2.*phi.^2.*WW.^3.*ZZ+2.*phi.*XX.*ZZ+(-2).*phi.^2.*XX.*ZZ+ ...

(-3).*WW.*XX.*ZZ+2.*phi.*WW.*XX.*ZZ+4.*phi.^2.*WW.*XX.*ZZ+(-1).*WW.^2.* ...

XX.*ZZ+(-4).*phi.*WW.^2.*XX.*ZZ+(-2).*phi.^2.*WW.^2.*XX.*ZZ+(-1).* ...

phi.^2.*WW.*YY.*ZZ+4.*phi.*WW.^2.*YY.*ZZ+phi.^2.*WW.^2.*YY.*ZZ+(-3).* ...

WW.^3.*YY.*ZZ+(-2).*phi.*WW.^3.*YY.*ZZ+phi.^2.*WW.^3.*YY.*ZZ+(-1).* ...

WW.^4.*YY.*ZZ+(-2).*phi.*WW.^4.*YY.*ZZ+(-1).*phi.^2.*WW.^4.*YY.*ZZ+ ...

phi.^2.*XX.*YY.*ZZ+(-4).*phi.*WW.*XX.*YY.*ZZ+(-1).*phi.^2.*WW.*XX.*YY.* ...

ZZ+3.*WW.^2.*XX.*YY.*ZZ+2.*phi.*WW.^2.*XX.*YY.*ZZ+(-1).*phi.^2.*WW.^2.* ...

XX.*YY.*ZZ+WW.^3.*XX.*YY.*ZZ+2.*phi.*WW.^3.*XX.*YY.*ZZ+phi.^2.*WW.^3.* ...

XX.*YY.*ZZ).^2+((-4).*(12.*(WW+(-2).*phi.*WW+phi.^2.*WW+2.*phi.*WW.^2+( ...

-2).*phi.^2.*WW.^2+phi.^2.*WW.^3+(-1).*XX+2.*phi.*XX+(-1).*phi.^2.*XX+( ...

-2).*phi.*WW.*XX+2.*phi.^2.*WW.*XX+(-1).*phi.^2.*WW.^2.*XX+phi.*WW.*YY+( ...

-1).*phi.^2.*WW.*YY+(-1).*WW.^2.*YY+2.*phi.^2.*WW.^2.*YY+(-1).*phi.* ...

WW.^3.*YY+(-1).*phi.^2.*WW.^3.*YY+(-1).*phi.*XX.*YY+phi.^2.*XX.*YY+WW.* ...

XX.*YY+(-2).*phi.^2.*WW.*XX.*YY+phi.*WW.^2.*XX.*YY+phi.^2.*WW.^2.*XX.* ...

YY+phi.*WW.*ZZ+(-1).*phi.^2.*WW.*ZZ+(-1).*WW.^2.*ZZ+2.*phi.^2.*WW.^2.* ...

ZZ+(-1).*phi.*WW.^3.*ZZ+(-1).*phi.^2.*WW.^3.*ZZ+(-1).*phi.*XX.*ZZ+ ...

phi.^2.*XX.*ZZ+WW.*XX.*ZZ+(-2).*phi.^2.*WW.*XX.*ZZ+phi.*WW.^2.*XX.*ZZ+ ...

phi.^2.*WW.^2.*XX.*ZZ+phi.^2.*WW.*YY.*ZZ+(-2).*phi.*WW.^2.*YY.*ZZ+(-2).* ...

phi.^2.*WW.^2.*YY.*ZZ+WW.^3.*YY.*ZZ+2.*phi.*WW.^3.*YY.*ZZ+phi.^2.* ...

WW.^3.*YY.*ZZ+(-1).*phi.^2.*XX.*YY.*ZZ+2.*phi.*WW.*XX.*YY.*ZZ+2.* ...

phi.^2.*WW.*XX.*YY.*ZZ+(-1).*WW.^2.*XX.*YY.*ZZ+(-2).*phi.*WW.^2.*XX.* ...

YY.*ZZ+(-1).*phi.^2.*WW.^2.*XX.*YY.*ZZ).*(WW.^4+(-1).*WW.^3.*XX+WW.^3.* ...

YY+(-2).*WW.^4.*YY+WW.^4.*XX.*YY+(-1).*WW.^3.*ZZ+2.*WW.^3.*XX.*ZZ+(-1).* ...

WW.^4.*XX.*ZZ+WW.^4.*YY.*ZZ+(-1).*WW.^3.*XX.*YY.*ZZ)+(3.*WW.^2+(-4).* ...

phi.*WW.^2+phi.^2.*WW.^2+3.*WW.^3+2.*phi.*WW.^3+(-2).*phi.^2.*WW.^3+2.* ...

phi.*WW.^4+phi.^2.*WW.^4+(-3).*WW.*XX+4.*phi.*WW.*XX+(-1).*phi.^2.*WW.* ...

XX+(-3).*WW.^2.*XX+(-2).*phi.*WW.^2.*XX+2.*phi.^2.*WW.^2.*XX+(-2).*phi.* ...

WW.^3.*XX+(-1).*phi.^2.*WW.^3.*XX+(-1).*phi.*WW.*YY+phi.^2.*WW.*YY+ ...

WW.^2.*YY+2.*phi.*WW.^2.*YY+(-4).*phi.^2.*WW.^2.*YY+(-5).*WW.^3.*YY+5.* ...

phi.*WW.^3.*YY+5.*phi.^2.*WW.^3.*YY+(-2).*WW.^4.*YY+(-6).*phi.*WW.^4.* ...

YY+(-2).*phi.^2.*WW.^4.*YY+3.*WW.^2.*XX.*YY+(-5).*phi.*WW.^2.*XX.*YY+ ...

phi.^2.*WW.^2.*XX.*YY+3.*WW.^3.*XX.*YY+4.*phi.*WW.^3.*XX.*YY+(-2).* ...

phi.^2.*WW.^3.*XX.*YY+phi.*WW.^4.*XX.*YY+phi.^2.*WW.^4.*XX.*YY+phi.*WW.* ...

ZZ+(-1).*phi.^2.*WW.*ZZ+(-3).*WW.^2.*ZZ+4.*phi.*WW.^2.*ZZ+2.*phi.^2.* ...

WW.^2.*ZZ+(-3).*WW.^3.*ZZ+(-5).*phi.*WW.^3.*ZZ+(-1).*phi.^2.*WW.^3.*ZZ+ ...

2.*WW.*XX.*ZZ+(-6).*phi.*WW.*XX.*ZZ+2.*phi.^2.*WW.*XX.*ZZ+5.*WW.^2.*XX.* ...

ZZ+5.*phi.*WW.^2.*XX.*ZZ+(-5).*phi.^2.*WW.^2.*XX.*ZZ+(-1).*WW.^3.*XX.* ...

ZZ+2.*phi.*WW.^3.*XX.*ZZ+4.*phi.^2.*WW.^3.*XX.*ZZ+(-1).*phi.*WW.^4.*XX.* ...

ZZ+(-1).*phi.^2.*WW.^4.*XX.*ZZ+(-2).*phi.*WW.^2.*YY.*ZZ+phi.^2.*WW.^2.* ...

YY.*ZZ+3.*WW.^3.*YY.*ZZ+(-2).*phi.*WW.^3.*YY.*ZZ+(-2).*phi.^2.*WW.^3.* ...

YY.*ZZ+3.*WW.^4.*YY.*ZZ+4.*phi.*WW.^4.*YY.*ZZ+phi.^2.*WW.^4.*YY.*ZZ+2.* ...

phi.*WW.*XX.*YY.*ZZ+(-1).*phi.^2.*WW.*XX.*YY.*ZZ+(-3).*WW.^2.*XX.*YY.* ...

ZZ+2.*phi.*WW.^2.*XX.*YY.*ZZ+2.*phi.^2.*WW.^2.*XX.*YY.*ZZ+(-3).*WW.^3.* ...

XX.*YY.*ZZ+(-4).*phi.*WW.^3.*XX.*YY.*ZZ+(-1).*phi.^2.*WW.^3.*XX.*YY.*ZZ) ...

.^2+(-3).*((-3).*WW.^3+2.*phi.*WW.^3+(-1).*WW.^4+(-2).*phi.*WW.^4+3.* ...

WW.^2.*XX+(-2).*phi.*WW.^2.*XX+WW.^3.*XX+2.*phi.*WW.^3.*XX+(-1).*WW.^2.* ...

YY+2.*phi.*WW.^2.*YY+WW.^3.*YY+(-6).*phi.*WW.^3.*YY+4.*WW.^4.*YY+4.* ...

phi.*WW.^4.*YY+(-3).*WW.^3.*XX.*YY+2.*phi.*WW.^3.*XX.*YY+(-1).*WW.^4.* ...

XX.*YY+(-2).*phi.*WW.^4.*XX.*YY+WW.^2.*ZZ+(-2).*phi.*WW.^2.*ZZ+3.* ...

WW.^3.*ZZ+2.*phi.*WW.^3.*ZZ+(-4).*WW.^2.*XX.*ZZ+4.*phi.*WW.^2.*XX.*ZZ+( ...

-1).*WW.^3.*XX.*ZZ+(-6).*phi.*WW.^3.*XX.*ZZ+WW.^4.*XX.*ZZ+2.*phi.* ...

WW.^4.*XX.*ZZ+(-1).*WW.^3.*YY.*ZZ+2.*phi.*WW.^3.*YY.*ZZ+(-3).*WW.^4.* ...

YY.*ZZ+(-2).*phi.*WW.^4.*YY.*ZZ+WW.^2.*XX.*YY.*ZZ+(-2).*phi.*WW.^2.*XX.* ...

YY.*ZZ+3.*WW.^3.*XX.*YY.*ZZ+2.*phi.*WW.^3.*XX.*YY.*ZZ).*((-1).*WW+2.* ...

phi.*WW+(-1).*phi.^2.*WW+(-3).*WW.^2+2.*phi.*WW.^2+phi.^2.*WW.^2+(-4).* ...

phi.*WW.^3+phi.^2.*WW.^3+(-1).*phi.^2.*WW.^4+XX+(-2).*phi.*XX+phi.^2.* ...

XX+3.*WW.*XX+(-2).*phi.*WW.*XX+(-1).*phi.^2.*WW.*XX+4.*phi.*WW.^2.*XX+( ...

-1).*phi.^2.*WW.^2.*XX+phi.^2.*WW.^3.*XX+WW.^2.*YY+(-4).*phi.*WW.^2.*YY+ ...

2.*phi.^2.*WW.^2.*YY+3.*WW.^3.*YY+2.*phi.*WW.^3.*YY+(-4).*phi.^2.* ...

WW.^3.*YY+2.*phi.*WW.^4.*YY+2.*phi.^2.*WW.^4.*YY+(-1).*WW.*XX.*YY+4.* ...

phi.*WW.*XX.*YY+(-2).*phi.^2.*WW.*XX.*YY+(-3).*WW.^2.*XX.*YY+(-2).*phi.* ...

WW.^2.*XX.*YY+4.*phi.^2.*WW.^2.*XX.*YY+(-2).*phi.*WW.^3.*XX.*YY+(-2).* ...

phi.^2.*WW.^3.*XX.*YY+(-2).*phi.*WW.*ZZ+2.*phi.^2.*WW.*ZZ+3.*WW.^2.*ZZ+( ...

-2).*phi.*WW.^2.*ZZ+(-4).*phi.^2.*WW.^2.*ZZ+WW.^3.*ZZ+4.*phi.*WW.^3.*ZZ+ ...

2.*phi.^2.*WW.^3.*ZZ+2.*phi.*XX.*ZZ+(-2).*phi.^2.*XX.*ZZ+(-3).*WW.*XX.* ...

ZZ+2.*phi.*WW.*XX.*ZZ+4.*phi.^2.*WW.*XX.*ZZ+(-1).*WW.^2.*XX.*ZZ+(-4).* ...

phi.*WW.^2.*XX.*ZZ+(-2).*phi.^2.*WW.^2.*XX.*ZZ+(-1).*phi.^2.*WW.*YY.*ZZ+ ...

4.*phi.*WW.^2.*YY.*ZZ+phi.^2.*WW.^2.*YY.*ZZ+(-3).*WW.^3.*YY.*ZZ+(-2).* ...

phi.*WW.^3.*YY.*ZZ+phi.^2.*WW.^3.*YY.*ZZ+(-1).*WW.^4.*YY.*ZZ+(-2).*phi.* ...

WW.^4.*YY.*ZZ+(-1).*phi.^2.*WW.^4.*YY.*ZZ+phi.^2.*XX.*YY.*ZZ+(-4).*phi.* ...

WW.*XX.*YY.*ZZ+(-1).*phi.^2.*WW.*XX.*YY.*ZZ+3.*WW.^2.*XX.*YY.*ZZ+2.* ...

phi.*WW.^2.*XX.*YY.*ZZ+(-1).*phi.^2.*WW.^2.*XX.*YY.*ZZ+WW.^3.*XX.*YY.* ...

ZZ+2.*phi.*WW.^3.*XX.*YY.*ZZ+phi.^2.*WW.^3.*XX.*YY.*ZZ)).^3+(27.*(WW+( ...

-2).*phi.*WW+phi.^2.*WW+2.*phi.*WW.^2+(-2).*phi.^2.*WW.^2+phi.^2.*WW.^3+ ...

(-1).*XX+2.*phi.*XX+(-1).*phi.^2.*XX+(-2).*phi.*WW.*XX+2.*phi.^2.*WW.* ...

XX+(-1).*phi.^2.*WW.^2.*XX+phi.*WW.*YY+(-1).*phi.^2.*WW.*YY+(-1).* ...

WW.^2.*YY+2.*phi.^2.*WW.^2.*YY+(-1).*phi.*WW.^3.*YY+(-1).*phi.^2.* ...

WW.^3.*YY+(-1).*phi.*XX.*YY+phi.^2.*XX.*YY+WW.*XX.*YY+(-2).*phi.^2.*WW.* ...

XX.*YY+phi.*WW.^2.*XX.*YY+phi.^2.*WW.^2.*XX.*YY+phi.*WW.*ZZ+(-1).* ...

phi.^2.*WW.*ZZ+(-1).*WW.^2.*ZZ+2.*phi.^2.*WW.^2.*ZZ+(-1).*phi.*WW.^3.* ...

ZZ+(-1).*phi.^2.*WW.^3.*ZZ+(-1).*phi.*XX.*ZZ+phi.^2.*XX.*ZZ+WW.*XX.*ZZ+( ...

-2).*phi.^2.*WW.*XX.*ZZ+phi.*WW.^2.*XX.*ZZ+phi.^2.*WW.^2.*XX.*ZZ+ ...

phi.^2.*WW.*YY.*ZZ+(-2).*phi.*WW.^2.*YY.*ZZ+(-2).*phi.^2.*WW.^2.*YY.*ZZ+ ...

WW.^3.*YY.*ZZ+2.*phi.*WW.^3.*YY.*ZZ+phi.^2.*WW.^3.*YY.*ZZ+(-1).*phi.^2.* ...

XX.*YY.*ZZ+2.*phi.*WW.*XX.*YY.*ZZ+2.*phi.^2.*WW.*XX.*YY.*ZZ+(-1).* ...

WW.^2.*XX.*YY.*ZZ+(-2).*phi.*WW.^2.*XX.*YY.*ZZ+(-1).*phi.^2.*WW.^2.*XX.* ...

YY.*ZZ).*((-3).*WW.^3+2.*phi.*WW.^3+(-1).*WW.^4+(-2).*phi.*WW.^4+3.* ...

WW.^2.*XX+(-2).*phi.*WW.^2.*XX+WW.^3.*XX+2.*phi.*WW.^3.*XX+(-1).*WW.^2.* ...

YY+2.*phi.*WW.^2.*YY+WW.^3.*YY+(-6).*phi.*WW.^3.*YY+4.*WW.^4.*YY+4.* ...

phi.*WW.^4.*YY+(-3).*WW.^3.*XX.*YY+2.*phi.*WW.^3.*XX.*YY+(-1).*WW.^4.* ...

XX.*YY+(-2).*phi.*WW.^4.*XX.*YY+WW.^2.*ZZ+(-2).*phi.*WW.^2.*ZZ+3.* ...

WW.^3.*ZZ+2.*phi.*WW.^3.*ZZ+(-4).*WW.^2.*XX.*ZZ+4.*phi.*WW.^2.*XX.*ZZ+( ...

-1).*WW.^3.*XX.*ZZ+(-6).*phi.*WW.^3.*XX.*ZZ+WW.^4.*XX.*ZZ+2.*phi.* ...

WW.^4.*XX.*ZZ+(-1).*WW.^3.*YY.*ZZ+2.*phi.*WW.^3.*YY.*ZZ+(-3).*WW.^4.* ...

YY.*ZZ+(-2).*phi.*WW.^4.*YY.*ZZ+WW.^2.*XX.*YY.*ZZ+(-2).*phi.*WW.^2.*XX.* ...

YY.*ZZ+3.*WW.^3.*XX.*YY.*ZZ+2.*phi.*WW.^3.*XX.*YY.*ZZ).^2+(-72).*(WW+( ...

-2).*phi.*WW+phi.^2.*WW+2.*phi.*WW.^2+(-2).*phi.^2.*WW.^2+phi.^2.*WW.^3+ ...

(-1).*XX+2.*phi.*XX+(-1).*phi.^2.*XX+(-2).*phi.*WW.*XX+2.*phi.^2.*WW.* ...

XX+(-1).*phi.^2.*WW.^2.*XX+phi.*WW.*YY+(-1).*phi.^2.*WW.*YY+(-1).* ...

WW.^2.*YY+2.*phi.^2.*WW.^2.*YY+(-1).*phi.*WW.^3.*YY+(-1).*phi.^2.* ...

WW.^3.*YY+(-1).*phi.*XX.*YY+phi.^2.*XX.*YY+WW.*XX.*YY+(-2).*phi.^2.*WW.* ...

XX.*YY+phi.*WW.^2.*XX.*YY+phi.^2.*WW.^2.*XX.*YY+phi.*WW.*ZZ+(-1).* ...

phi.^2.*WW.*ZZ+(-1).*WW.^2.*ZZ+2.*phi.^2.*WW.^2.*ZZ+(-1).*phi.*WW.^3.* ...

ZZ+(-1).*phi.^2.*WW.^3.*ZZ+(-1).*phi.*XX.*ZZ+phi.^2.*XX.*ZZ+WW.*XX.*ZZ+( ...

-2).*phi.^2.*WW.*XX.*ZZ+phi.*WW.^2.*XX.*ZZ+phi.^2.*WW.^2.*XX.*ZZ+ ...

phi.^2.*WW.*YY.*ZZ+(-2).*phi.*WW.^2.*YY.*ZZ+(-2).*phi.^2.*WW.^2.*YY.*ZZ+ ...

WW.^3.*YY.*ZZ+2.*phi.*WW.^3.*YY.*ZZ+phi.^2.*WW.^3.*YY.*ZZ+(-1).*phi.^2.* ...

XX.*YY.*ZZ+2.*phi.*WW.*XX.*YY.*ZZ+2.*phi.^2.*WW.*XX.*YY.*ZZ+(-1).* ...

WW.^2.*XX.*YY.*ZZ+(-2).*phi.*WW.^2.*XX.*YY.*ZZ+(-1).*phi.^2.*WW.^2.*XX.* ...

YY.*ZZ).*(WW.^4+(-1).*WW.^3.*XX+WW.^3.*YY+(-2).*WW.^4.*YY+WW.^4.*XX.*YY+ ...

(-1).*WW.^3.*ZZ+2.*WW.^3.*XX.*ZZ+(-1).*WW.^4.*XX.*ZZ+WW.^4.*YY.*ZZ+(-1) ...

.*WW.^3.*XX.*YY.*ZZ).*(3.*WW.^2+(-4).*phi.*WW.^2+phi.^2.*WW.^2+3.*WW.^3+ ...

2.*phi.*WW.^3+(-2).*phi.^2.*WW.^3+2.*phi.*WW.^4+phi.^2.*WW.^4+(-3).*WW.* ...

XX+4.*phi.*WW.*XX+(-1).*phi.^2.*WW.*XX+(-3).*WW.^2.*XX+(-2).*phi.* ...

WW.^2.*XX+2.*phi.^2.*WW.^2.*XX+(-2).*phi.*WW.^3.*XX+(-1).*phi.^2.* ...

WW.^3.*XX+(-1).*phi.*WW.*YY+phi.^2.*WW.*YY+WW.^2.*YY+2.*phi.*WW.^2.*YY+( ...

-4).*phi.^2.*WW.^2.*YY+(-5).*WW.^3.*YY+5.*phi.*WW.^3.*YY+5.*phi.^2.* ...

WW.^3.*YY+(-2).*WW.^4.*YY+(-6).*phi.*WW.^4.*YY+(-2).*phi.^2.*WW.^4.*YY+ ...

3.*WW.^2.*XX.*YY+(-5).*phi.*WW.^2.*XX.*YY+phi.^2.*WW.^2.*XX.*YY+3.* ...

WW.^3.*XX.*YY+4.*phi.*WW.^3.*XX.*YY+(-2).*phi.^2.*WW.^3.*XX.*YY+phi.* ...

WW.^4.*XX.*YY+phi.^2.*WW.^4.*XX.*YY+phi.*WW.*ZZ+(-1).*phi.^2.*WW.*ZZ+( ...

-3).*WW.^2.*ZZ+4.*phi.*WW.^2.*ZZ+2.*phi.^2.*WW.^2.*ZZ+(-3).*WW.^3.*ZZ+( ...

-5).*phi.*WW.^3.*ZZ+(-1).*phi.^2.*WW.^3.*ZZ+2.*WW.*XX.*ZZ+(-6).*phi.* ...

WW.*XX.*ZZ+2.*phi.^2.*WW.*XX.*ZZ+5.*WW.^2.*XX.*ZZ+5.*phi.*WW.^2.*XX.*ZZ+ ...

(-5).*phi.^2.*WW.^2.*XX.*ZZ+(-1).*WW.^3.*XX.*ZZ+2.*phi.*WW.^3.*XX.*ZZ+ ...

4.*phi.^2.*WW.^3.*XX.*ZZ+(-1).*phi.*WW.^4.*XX.*ZZ+(-1).*phi.^2.*WW.^4.* ...

XX.*ZZ+(-2).*phi.*WW.^2.*YY.*ZZ+phi.^2.*WW.^2.*YY.*ZZ+3.*WW.^3.*YY.*ZZ+( ...

-2).*phi.*WW.^3.*YY.*ZZ+(-2).*phi.^2.*WW.^3.*YY.*ZZ+3.*WW.^4.*YY.*ZZ+4.* ...

phi.*WW.^4.*YY.*ZZ+phi.^2.*WW.^4.*YY.*ZZ+2.*phi.*WW.*XX.*YY.*ZZ+(-1).* ...

phi.^2.*WW.*XX.*YY.*ZZ+(-3).*WW.^2.*XX.*YY.*ZZ+2.*phi.*WW.^2.*XX.*YY.* ...

ZZ+2.*phi.^2.*WW.^2.*XX.*YY.*ZZ+(-3).*WW.^3.*XX.*YY.*ZZ+(-4).*phi.* ...

WW.^3.*XX.*YY.*ZZ+(-1).*phi.^2.*WW.^3.*XX.*YY.*ZZ)+2.*(3.*WW.^2+(-4).* ...

phi.*WW.^2+phi.^2.*WW.^2+3.*WW.^3+2.*phi.*WW.^3+(-2).*phi.^2.*WW.^3+2.* ...

phi.*WW.^4+phi.^2.*WW.^4+(-3).*WW.*XX+4.*phi.*WW.*XX+(-1).*phi.^2.*WW.* ...

XX+(-3).*WW.^2.*XX+(-2).*phi.*WW.^2.*XX+2.*phi.^2.*WW.^2.*XX+(-2).*phi.* ...

WW.^3.*XX+(-1).*phi.^2.*WW.^3.*XX+(-1).*phi.*WW.*YY+phi.^2.*WW.*YY+ ...

WW.^2.*YY+2.*phi.*WW.^2.*YY+(-4).*phi.^2.*WW.^2.*YY+(-5).*WW.^3.*YY+5.* ...

phi.*WW.^3.*YY+5.*phi.^2.*WW.^3.*YY+(-2).*WW.^4.*YY+(-6).*phi.*WW.^4.* ...

YY+(-2).*phi.^2.*WW.^4.*YY+3.*WW.^2.*XX.*YY+(-5).*phi.*WW.^2.*XX.*YY+ ...

phi.^2.*WW.^2.*XX.*YY+3.*WW.^3.*XX.*YY+4.*phi.*WW.^3.*XX.*YY+(-2).* ...

phi.^2.*WW.^3.*XX.*YY+phi.*WW.^4.*XX.*YY+phi.^2.*WW.^4.*XX.*YY+phi.*WW.* ...

ZZ+(-1).*phi.^2.*WW.*ZZ+(-3).*WW.^2.*ZZ+4.*phi.*WW.^2.*ZZ+2.*phi.^2.* ...

WW.^2.*ZZ+(-3).*WW.^3.*ZZ+(-5).*phi.*WW.^3.*ZZ+(-1).*phi.^2.*WW.^3.*ZZ+ ...

2.*WW.*XX.*ZZ+(-6).*phi.*WW.*XX.*ZZ+2.*phi.^2.*WW.*XX.*ZZ+5.*WW.^2.*XX.* ...

ZZ+5.*phi.*WW.^2.*XX.*ZZ+(-5).*phi.^2.*WW.^2.*XX.*ZZ+(-1).*WW.^3.*XX.* ...

ZZ+2.*phi.*WW.^3.*XX.*ZZ+4.*phi.^2.*WW.^3.*XX.*ZZ+(-1).*phi.*WW.^4.*XX.* ...

ZZ+(-1).*phi.^2.*WW.^4.*XX.*ZZ+(-2).*phi.*WW.^2.*YY.*ZZ+phi.^2.*WW.^2.* ...

YY.*ZZ+3.*WW.^3.*YY.*ZZ+(-2).*phi.*WW.^3.*YY.*ZZ+(-2).*phi.^2.*WW.^3.* ...

YY.*ZZ+3.*WW.^4.*YY.*ZZ+4.*phi.*WW.^4.*YY.*ZZ+phi.^2.*WW.^4.*YY.*ZZ+2.* ...

phi.*WW.*XX.*YY.*ZZ+(-1).*phi.^2.*WW.*XX.*YY.*ZZ+(-3).*WW.^2.*XX.*YY.* ...

ZZ+2.*phi.*WW.^2.*XX.*YY.*ZZ+2.*phi.^2.*WW.^2.*XX.*YY.*ZZ+(-3).*WW.^3.* ...

XX.*YY.*ZZ+(-4).*phi.*WW.^3.*XX.*YY.*ZZ+(-1).*phi.^2.*WW.^3.*XX.*YY.*ZZ) ...

.^3+(-9).*((-3).*WW.^3+2.*phi.*WW.^3+(-1).*WW.^4+(-2).*phi.*WW.^4+3.* ...

WW.^2.*XX+(-2).*phi.*WW.^2.*XX+WW.^3.*XX+2.*phi.*WW.^3.*XX+(-1).*WW.^2.* ...

YY+2.*phi.*WW.^2.*YY+WW.^3.*YY+(-6).*phi.*WW.^3.*YY+4.*WW.^4.*YY+4.* ...

phi.*WW.^4.*YY+(-3).*WW.^3.*XX.*YY+2.*phi.*WW.^3.*XX.*YY+(-1).*WW.^4.* ...

XX.*YY+(-2).*phi.*WW.^4.*XX.*YY+WW.^2.*ZZ+(-2).*phi.*WW.^2.*ZZ+3.* ...

WW.^3.*ZZ+2.*phi.*WW.^3.*ZZ+(-4).*WW.^2.*XX.*ZZ+4.*phi.*WW.^2.*XX.*ZZ+( ...

-1).*WW.^3.*XX.*ZZ+(-6).*phi.*WW.^3.*XX.*ZZ+WW.^4.*XX.*ZZ+2.*phi.* ...

WW.^4.*XX.*ZZ+(-1).*WW.^3.*YY.*ZZ+2.*phi.*WW.^3.*YY.*ZZ+(-3).*WW.^4.* ...

YY.*ZZ+(-2).*phi.*WW.^4.*YY.*ZZ+WW.^2.*XX.*YY.*ZZ+(-2).*phi.*WW.^2.*XX.* ...

YY.*ZZ+3.*WW.^3.*XX.*YY.*ZZ+2.*phi.*WW.^3.*XX.*YY.*ZZ).*(3.*WW.^2+(-4).* ...

phi.*WW.^2+phi.^2.*WW.^2+3.*WW.^3+2.*phi.*WW.^3+(-2).*phi.^2.*WW.^3+2.* ...

phi.*WW.^4+phi.^2.*WW.^4+(-3).*WW.*XX+4.*phi.*WW.*XX+(-1).*phi.^2.*WW.* ...

XX+(-3).*WW.^2.*XX+(-2).*phi.*WW.^2.*XX+2.*phi.^2.*WW.^2.*XX+(-2).*phi.* ...

WW.^3.*XX+(-1).*phi.^2.*WW.^3.*XX+(-1).*phi.*WW.*YY+phi.^2.*WW.*YY+ ...

WW.^2.*YY+2.*phi.*WW.^2.*YY+(-4).*phi.^2.*WW.^2.*YY+(-5).*WW.^3.*YY+5.* ...

phi.*WW.^3.*YY+5.*phi.^2.*WW.^3.*YY+(-2).*WW.^4.*YY+(-6).*phi.*WW.^4.* ...

YY+(-2).*phi.^2.*WW.^4.*YY+3.*WW.^2.*XX.*YY+(-5).*phi.*WW.^2.*XX.*YY+ ...

phi.^2.*WW.^2.*XX.*YY+3.*WW.^3.*XX.*YY+4.*phi.*WW.^3.*XX.*YY+(-2).* ...

phi.^2.*WW.^3.*XX.*YY+phi.*WW.^4.*XX.*YY+phi.^2.*WW.^4.*XX.*YY+phi.*WW.* ...

ZZ+(-1).*phi.^2.*WW.*ZZ+(-3).*WW.^2.*ZZ+4.*phi.*WW.^2.*ZZ+2.*phi.^2.* ...

WW.^2.*ZZ+(-3).*WW.^3.*ZZ+(-5).*phi.*WW.^3.*ZZ+(-1).*phi.^2.*WW.^3.*ZZ+ ...

2.*WW.*XX.*ZZ+(-6).*phi.*WW.*XX.*ZZ+2.*phi.^2.*WW.*XX.*ZZ+5.*WW.^2.*XX.* ...

ZZ+5.*phi.*WW.^2.*XX.*ZZ+(-5).*phi.^2.*WW.^2.*XX.*ZZ+(-1).*WW.^3.*XX.* ...

ZZ+2.*phi.*WW.^3.*XX.*ZZ+4.*phi.^2.*WW.^3.*XX.*ZZ+(-1).*phi.*WW.^4.*XX.* ...

ZZ+(-1).*phi.^2.*WW.^4.*XX.*ZZ+(-2).*phi.*WW.^2.*YY.*ZZ+phi.^2.*WW.^2.* ...

YY.*ZZ+3.*WW.^3.*YY.*ZZ+(-2).*phi.*WW.^3.*YY.*ZZ+(-2).*phi.^2.*WW.^3.* ...

YY.*ZZ+3.*WW.^4.*YY.*ZZ+4.*phi.*WW.^4.*YY.*ZZ+phi.^2.*WW.^4.*YY.*ZZ+2.* ...

phi.*WW.*XX.*YY.*ZZ+(-1).*phi.^2.*WW.*XX.*YY.*ZZ+(-3).*WW.^2.*XX.*YY.* ...

ZZ+2.*phi.*WW.^2.*XX.*YY.*ZZ+2.*phi.^2.*WW.^2.*XX.*YY.*ZZ+(-3).*WW.^3.* ...

XX.*YY.*ZZ+(-4).*phi.*WW.^3.*XX.*YY.*ZZ+(-1).*phi.^2.*WW.^3.*XX.*YY.*ZZ) ...

.*((-1).*WW+2.*phi.*WW+(-1).*phi.^2.*WW+(-3).*WW.^2+2.*phi.*WW.^2+ ...

phi.^2.*WW.^2+(-4).*phi.*WW.^3+phi.^2.*WW.^3+(-1).*phi.^2.*WW.^4+XX+(-2) ...

.*phi.*XX+phi.^2.*XX+3.*WW.*XX+(-2).*phi.*WW.*XX+(-1).*phi.^2.*WW.*XX+ ...

4.*phi.*WW.^2.*XX+(-1).*phi.^2.*WW.^2.*XX+phi.^2.*WW.^3.*XX+WW.^2.*YY+( ...

-4).*phi.*WW.^2.*YY+2.*phi.^2.*WW.^2.*YY+3.*WW.^3.*YY+2.*phi.*WW.^3.*YY+ ...

(-4).*phi.^2.*WW.^3.*YY+2.*phi.*WW.^4.*YY+2.*phi.^2.*WW.^4.*YY+(-1).* ...

WW.*XX.*YY+4.*phi.*WW.*XX.*YY+(-2).*phi.^2.*WW.*XX.*YY+(-3).*WW.^2.*XX.* ...

YY+(-2).*phi.*WW.^2.*XX.*YY+4.*phi.^2.*WW.^2.*XX.*YY+(-2).*phi.*WW.^3.* ...

XX.*YY+(-2).*phi.^2.*WW.^3.*XX.*YY+(-2).*phi.*WW.*ZZ+2.*phi.^2.*WW.*ZZ+ ...

3.*WW.^2.*ZZ+(-2).*phi.*WW.^2.*ZZ+(-4).*phi.^2.*WW.^2.*ZZ+WW.^3.*ZZ+4.* ...

phi.*WW.^3.*ZZ+2.*phi.^2.*WW.^3.*ZZ+2.*phi.*XX.*ZZ+(-2).*phi.^2.*XX.*ZZ+ ...

(-3).*WW.*XX.*ZZ+2.*phi.*WW.*XX.*ZZ+4.*phi.^2.*WW.*XX.*ZZ+(-1).*WW.^2.* ...

XX.*ZZ+(-4).*phi.*WW.^2.*XX.*ZZ+(-2).*phi.^2.*WW.^2.*XX.*ZZ+(-1).* ...

phi.^2.*WW.*YY.*ZZ+4.*phi.*WW.^2.*YY.*ZZ+phi.^2.*WW.^2.*YY.*ZZ+(-3).* ...

WW.^3.*YY.*ZZ+(-2).*phi.*WW.^3.*YY.*ZZ+phi.^2.*WW.^3.*YY.*ZZ+(-1).* ...

WW.^4.*YY.*ZZ+(-2).*phi.*WW.^4.*YY.*ZZ+(-1).*phi.^2.*WW.^4.*YY.*ZZ+ ...

phi.^2.*XX.*YY.*ZZ+(-4).*phi.*WW.*XX.*YY.*ZZ+(-1).*phi.^2.*WW.*XX.*YY.* ...

ZZ+3.*WW.^2.*XX.*YY.*ZZ+2.*phi.*WW.^2.*XX.*YY.*ZZ+(-1).*phi.^2.*WW.^2.* ...

XX.*YY.*ZZ+WW.^3.*XX.*YY.*ZZ+2.*phi.*WW.^3.*XX.*YY.*ZZ+phi.^2.*WW.^3.* ...

XX.*YY.*ZZ)+27.*(WW.^4+(-1).*WW.^3.*XX+WW.^3.*YY+(-2).*WW.^4.*YY+WW.^4.* ...

XX.*YY+(-1).*WW.^3.*ZZ+2.*WW.^3.*XX.*ZZ+(-1).*WW.^4.*XX.*ZZ+WW.^4.*YY.* ...

ZZ+(-1).*WW.^3.*XX.*YY.*ZZ).*((-1).*WW+2.*phi.*WW+(-1).*phi.^2.*WW+(-3) ...

.*WW.^2+2.*phi.*WW.^2+phi.^2.*WW.^2+(-4).*phi.*WW.^3+phi.^2.*WW.^3+(-1) ...

.*phi.^2.*WW.^4+XX+(-2).*phi.*XX+phi.^2.*XX+3.*WW.*XX+(-2).*phi.*WW.*XX+ ...

(-1).*phi.^2.*WW.*XX+4.*phi.*WW.^2.*XX+(-1).*phi.^2.*WW.^2.*XX+phi.^2.* ...

WW.^3.*XX+WW.^2.*YY+(-4).*phi.*WW.^2.*YY+2.*phi.^2.*WW.^2.*YY+3.*WW.^3.* ...

YY+2.*phi.*WW.^3.*YY+(-4).*phi.^2.*WW.^3.*YY+2.*phi.*WW.^4.*YY+2.* ...

phi.^2.*WW.^4.*YY+(-1).*WW.*XX.*YY+4.*phi.*WW.*XX.*YY+(-2).*phi.^2.*WW.* ...

XX.*YY+(-3).*WW.^2.*XX.*YY+(-2).*phi.*WW.^2.*XX.*YY+4.*phi.^2.*WW.^2.* ...

XX.*YY+(-2).*phi.*WW.^3.*XX.*YY+(-2).*phi.^2.*WW.^3.*XX.*YY+(-2).*phi.* ...

WW.*ZZ+2.*phi.^2.*WW.*ZZ+3.*WW.^2.*ZZ+(-2).*phi.*WW.^2.*ZZ+(-4).* ...

phi.^2.*WW.^2.*ZZ+WW.^3.*ZZ+4.*phi.*WW.^3.*ZZ+2.*phi.^2.*WW.^3.*ZZ+2.* ...

phi.*XX.*ZZ+(-2).*phi.^2.*XX.*ZZ+(-3).*WW.*XX.*ZZ+2.*phi.*WW.*XX.*ZZ+4.* ...

phi.^2.*WW.*XX.*ZZ+(-1).*WW.^2.*XX.*ZZ+(-4).*phi.*WW.^2.*XX.*ZZ+(-2).* ...

phi.^2.*WW.^2.*XX.*ZZ+(-1).*phi.^2.*WW.*YY.*ZZ+4.*phi.*WW.^2.*YY.*ZZ+ ...

phi.^2.*WW.^2.*YY.*ZZ+(-3).*WW.^3.*YY.*ZZ+(-2).*phi.*WW.^3.*YY.*ZZ+ ...

phi.^2.*WW.^3.*YY.*ZZ+(-1).*WW.^4.*YY.*ZZ+(-2).*phi.*WW.^4.*YY.*ZZ+(-1) ...

.*phi.^2.*WW.^4.*YY.*ZZ+phi.^2.*XX.*YY.*ZZ+(-4).*phi.*WW.*XX.*YY.*ZZ+( ...

-1).*phi.^2.*WW.*XX.*YY.*ZZ+3.*WW.^2.*XX.*YY.*ZZ+2.*phi.*WW.^2.*XX.*YY.* ...

ZZ+(-1).*phi.^2.*WW.^2.*XX.*YY.*ZZ+WW.^3.*XX.*YY.*ZZ+2.*phi.*WW.^3.*XX.* ...

YY.*ZZ+phi.^2.*WW.^3.*XX.*YY.*ZZ).^2).^2).^(1/2)).^(-1/3)+(1/3).*2.^( ...

-1/3).*(WW+(-2).*phi.*WW+phi.^2.*WW+2.*phi.*WW.^2+(-2).*phi.^2.*WW.^2+ ...

phi.^2.*WW.^3+(-1).*XX+2.*phi.*XX+(-1).*phi.^2.*XX+(-2).*phi.*WW.*XX+2.* ...

phi.^2.*WW.*XX+(-1).*phi.^2.*WW.^2.*XX+phi.*WW.*YY+(-1).*phi.^2.*WW.*YY+ ...

(-1).*WW.^2.*YY+2.*phi.^2.*WW.^2.*YY+(-1).*phi.*WW.^3.*YY+(-1).*phi.^2.* ...

WW.^3.*YY+(-1).*phi.*XX.*YY+phi.^2.*XX.*YY+WW.*XX.*YY+(-2).*phi.^2.*WW.* ...

XX.*YY+phi.*WW.^2.*XX.*YY+phi.^2.*WW.^2.*XX.*YY+phi.*WW.*ZZ+(-1).* ...

phi.^2.*WW.*ZZ+(-1).*WW.^2.*ZZ+2.*phi.^2.*WW.^2.*ZZ+(-1).*phi.*WW.^3.* ...

ZZ+(-1).*phi.^2.*WW.^3.*ZZ+(-1).*phi.*XX.*ZZ+phi.^2.*XX.*ZZ+WW.*XX.*ZZ+( ...

-2).*phi.^2.*WW.*XX.*ZZ+phi.*WW.^2.*XX.*ZZ+phi.^2.*WW.^2.*XX.*ZZ+ ...

phi.^2.*WW.*YY.*ZZ+(-2).*phi.*WW.^2.*YY.*ZZ+(-2).*phi.^2.*WW.^2.*YY.*ZZ+ ...

WW.^3.*YY.*ZZ+2.*phi.*WW.^3.*YY.*ZZ+phi.^2.*WW.^3.*YY.*ZZ+(-1).*phi.^2.* ...

XX.*YY.*ZZ+2.*phi.*WW.*XX.*YY.*ZZ+2.*phi.^2.*WW.*XX.*YY.*ZZ+(-1).* ...

WW.^2.*XX.*YY.*ZZ+(-2).*phi.*WW.^2.*XX.*YY.*ZZ+(-1).*phi.^2.*WW.^2.*XX.* ...

YY.*ZZ).^(-1).*(27.*(WW+(-2).*phi.*WW+phi.^2.*WW+2.*phi.*WW.^2+(-2).* ...

phi.^2.*WW.^2+phi.^2.*WW.^3+(-1).*XX+2.*phi.*XX+(-1).*phi.^2.*XX+(-2).* ...

phi.*WW.*XX+2.*phi.^2.*WW.*XX+(-1).*phi.^2.*WW.^2.*XX+phi.*WW.*YY+(-1).* ...

phi.^2.*WW.*YY+(-1).*WW.^2.*YY+2.*phi.^2.*WW.^2.*YY+(-1).*phi.*WW.^3.* ...

YY+(-1).*phi.^2.*WW.^3.*YY+(-1).*phi.*XX.*YY+phi.^2.*XX.*YY+WW.*XX.*YY+( ...

-2).*phi.^2.*WW.*XX.*YY+phi.*WW.^2.*XX.*YY+phi.^2.*WW.^2.*XX.*YY+phi.* ...

WW.*ZZ+(-1).*phi.^2.*WW.*ZZ+(-1).*WW.^2.*ZZ+2.*phi.^2.*WW.^2.*ZZ+(-1).* ...

phi.*WW.^3.*ZZ+(-1).*phi.^2.*WW.^3.*ZZ+(-1).*phi.*XX.*ZZ+phi.^2.*XX.*ZZ+ ...

WW.*XX.*ZZ+(-2).*phi.^2.*WW.*XX.*ZZ+phi.*WW.^2.*XX.*ZZ+phi.^2.*WW.^2.* ...

XX.*ZZ+phi.^2.*WW.*YY.*ZZ+(-2).*phi.*WW.^2.*YY.*ZZ+(-2).*phi.^2.*WW.^2.* ...

YY.*ZZ+WW.^3.*YY.*ZZ+2.*phi.*WW.^3.*YY.*ZZ+phi.^2.*WW.^3.*YY.*ZZ+(-1).* ...

phi.^2.*XX.*YY.*ZZ+2.*phi.*WW.*XX.*YY.*ZZ+2.*phi.^2.*WW.*XX.*YY.*ZZ+(-1) ...

.*WW.^2.*XX.*YY.*ZZ+(-2).*phi.*WW.^2.*XX.*YY.*ZZ+(-1).*phi.^2.*WW.^2.* ...

XX.*YY.*ZZ).*((-3).*WW.^3+2.*phi.*WW.^3+(-1).*WW.^4+(-2).*phi.*WW.^4+3.* ...

WW.^2.*XX+(-2).*phi.*WW.^2.*XX+WW.^3.*XX+2.*phi.*WW.^3.*XX+(-1).*WW.^2.* ...

YY+2.*phi.*WW.^2.*YY+WW.^3.*YY+(-6).*phi.*WW.^3.*YY+4.*WW.^4.*YY+4.* ...

phi.*WW.^4.*YY+(-3).*WW.^3.*XX.*YY+2.*phi.*WW.^3.*XX.*YY+(-1).*WW.^4.* ...

XX.*YY+(-2).*phi.*WW.^4.*XX.*YY+WW.^2.*ZZ+(-2).*phi.*WW.^2.*ZZ+3.* ...

WW.^3.*ZZ+2.*phi.*WW.^3.*ZZ+(-4).*WW.^2.*XX.*ZZ+4.*phi.*WW.^2.*XX.*ZZ+( ...

-1).*WW.^3.*XX.*ZZ+(-6).*phi.*WW.^3.*XX.*ZZ+WW.^4.*XX.*ZZ+2.*phi.* ...

WW.^4.*XX.*ZZ+(-1).*WW.^3.*YY.*ZZ+2.*phi.*WW.^3.*YY.*ZZ+(-3).*WW.^4.* ...

YY.*ZZ+(-2).*phi.*WW.^4.*YY.*ZZ+WW.^2.*XX.*YY.*ZZ+(-2).*phi.*WW.^2.*XX.* ...

YY.*ZZ+3.*WW.^3.*XX.*YY.*ZZ+2.*phi.*WW.^3.*XX.*YY.*ZZ).^2+(-72).*(WW+( ...

-2).*phi.*WW+phi.^2.*WW+2.*phi.*WW.^2+(-2).*phi.^2.*WW.^2+phi.^2.*WW.^3+ ...

(-1).*XX+2.*phi.*XX+(-1).*phi.^2.*XX+(-2).*phi.*WW.*XX+2.*phi.^2.*WW.* ...

XX+(-1).*phi.^2.*WW.^2.*XX+phi.*WW.*YY+(-1).*phi.^2.*WW.*YY+(-1).* ...

WW.^2.*YY+2.*phi.^2.*WW.^2.*YY+(-1).*phi.*WW.^3.*YY+(-1).*phi.^2.* ...

WW.^3.*YY+(-1).*phi.*XX.*YY+phi.^2.*XX.*YY+WW.*XX.*YY+(-2).*phi.^2.*WW.* ...

XX.*YY+phi.*WW.^2.*XX.*YY+phi.^2.*WW.^2.*XX.*YY+phi.*WW.*ZZ+(-1).* ...

phi.^2.*WW.*ZZ+(-1).*WW.^2.*ZZ+2.*phi.^2.*WW.^2.*ZZ+(-1).*phi.*WW.^3.* ...

ZZ+(-1).*phi.^2.*WW.^3.*ZZ+(-1).*phi.*XX.*ZZ+phi.^2.*XX.*ZZ+WW.*XX.*ZZ+( ...

-2).*phi.^2.*WW.*XX.*ZZ+phi.*WW.^2.*XX.*ZZ+phi.^2.*WW.^2.*XX.*ZZ+ ...

phi.^2.*WW.*YY.*ZZ+(-2).*phi.*WW.^2.*YY.*ZZ+(-2).*phi.^2.*WW.^2.*YY.*ZZ+ ...

WW.^3.*YY.*ZZ+2.*phi.*WW.^3.*YY.*ZZ+phi.^2.*WW.^3.*YY.*ZZ+(-1).*phi.^2.* ...

XX.*YY.*ZZ+2.*phi.*WW.*XX.*YY.*ZZ+2.*phi.^2.*WW.*XX.*YY.*ZZ+(-1).* ...

WW.^2.*XX.*YY.*ZZ+(-2).*phi.*WW.^2.*XX.*YY.*ZZ+(-1).*phi.^2.*WW.^2.*XX.* ...

YY.*ZZ).*(WW.^4+(-1).*WW.^3.*XX+WW.^3.*YY+(-2).*WW.^4.*YY+WW.^4.*XX.*YY+ ...

(-1).*WW.^3.*ZZ+2.*WW.^3.*XX.*ZZ+(-1).*WW.^4.*XX.*ZZ+WW.^4.*YY.*ZZ+(-1) ...

.*WW.^3.*XX.*YY.*ZZ).*(3.*WW.^2+(-4).*phi.*WW.^2+phi.^2.*WW.^2+3.*WW.^3+ ...

2.*phi.*WW.^3+(-2).*phi.^2.*WW.^3+2.*phi.*WW.^4+phi.^2.*WW.^4+(-3).*WW.* ...

XX+4.*phi.*WW.*XX+(-1).*phi.^2.*WW.*XX+(-3).*WW.^2.*XX+(-2).*phi.* ...

WW.^2.*XX+2.*phi.^2.*WW.^2.*XX+(-2).*phi.*WW.^3.*XX+(-1).*phi.^2.* ...

WW.^3.*XX+(-1).*phi.*WW.*YY+phi.^2.*WW.*YY+WW.^2.*YY+2.*phi.*WW.^2.*YY+( ...

-4).*phi.^2.*WW.^2.*YY+(-5).*WW.^3.*YY+5.*phi.*WW.^3.*YY+5.*phi.^2.* ...

WW.^3.*YY+(-2).*WW.^4.*YY+(-6).*phi.*WW.^4.*YY+(-2).*phi.^2.*WW.^4.*YY+ ...

3.*WW.^2.*XX.*YY+(-5).*phi.*WW.^2.*XX.*YY+phi.^2.*WW.^2.*XX.*YY+3.* ...

WW.^3.*XX.*YY+4.*phi.*WW.^3.*XX.*YY+(-2).*phi.^2.*WW.^3.*XX.*YY+phi.* ...

WW.^4.*XX.*YY+phi.^2.*WW.^4.*XX.*YY+phi.*WW.*ZZ+(-1).*phi.^2.*WW.*ZZ+( ...

-3).*WW.^2.*ZZ+4.*phi.*WW.^2.*ZZ+2.*phi.^2.*WW.^2.*ZZ+(-3).*WW.^3.*ZZ+( ...

-5).*phi.*WW.^3.*ZZ+(-1).*phi.^2.*WW.^3.*ZZ+2.*WW.*XX.*ZZ+(-6).*phi.* ...

WW.*XX.*ZZ+2.*phi.^2.*WW.*XX.*ZZ+5.*WW.^2.*XX.*ZZ+5.*phi.*WW.^2.*XX.*ZZ+ ...

(-5).*phi.^2.*WW.^2.*XX.*ZZ+(-1).*WW.^3.*XX.*ZZ+2.*phi.*WW.^3.*XX.*ZZ+ ...

4.*phi.^2.*WW.^3.*XX.*ZZ+(-1).*phi.*WW.^4.*XX.*ZZ+(-1).*phi.^2.*WW.^4.* ...

XX.*ZZ+(-2).*phi.*WW.^2.*YY.*ZZ+phi.^2.*WW.^2.*YY.*ZZ+3.*WW.^3.*YY.*ZZ+( ...

-2).*phi.*WW.^3.*YY.*ZZ+(-2).*phi.^2.*WW.^3.*YY.*ZZ+3.*WW.^4.*YY.*ZZ+4.* ...

phi.*WW.^4.*YY.*ZZ+phi.^2.*WW.^4.*YY.*ZZ+2.*phi.*WW.*XX.*YY.*ZZ+(-1).* ...

phi.^2.*WW.*XX.*YY.*ZZ+(-3).*WW.^2.*XX.*YY.*ZZ+2.*phi.*WW.^2.*XX.*YY.* ...

ZZ+2.*phi.^2.*WW.^2.*XX.*YY.*ZZ+(-3).*WW.^3.*XX.*YY.*ZZ+(-4).*phi.* ...

WW.^3.*XX.*YY.*ZZ+(-1).*phi.^2.*WW.^3.*XX.*YY.*ZZ)+2.*(3.*WW.^2+(-4).* ...

phi.*WW.^2+phi.^2.*WW.^2+3.*WW.^3+2.*phi.*WW.^3+(-2).*phi.^2.*WW.^3+2.* ...

phi.*WW.^4+phi.^2.*WW.^4+(-3).*WW.*XX+4.*phi.*WW.*XX+(-1).*phi.^2.*WW.* ...

XX+(-3).*WW.^2.*XX+(-2).*phi.*WW.^2.*XX+2.*phi.^2.*WW.^2.*XX+(-2).*phi.* ...

WW.^3.*XX+(-1).*phi.^2.*WW.^3.*XX+(-1).*phi.*WW.*YY+phi.^2.*WW.*YY+ ...

WW.^2.*YY+2.*phi.*WW.^2.*YY+(-4).*phi.^2.*WW.^2.*YY+(-5).*WW.^3.*YY+5.* ...

phi.*WW.^3.*YY+5.*phi.^2.*WW.^3.*YY+(-2).*WW.^4.*YY+(-6).*phi.*WW.^4.* ...

YY+(-2).*phi.^2.*WW.^4.*YY+3.*WW.^2.*XX.*YY+(-5).*phi.*WW.^2.*XX.*YY+ ...

phi.^2.*WW.^2.*XX.*YY+3.*WW.^3.*XX.*YY+4.*phi.*WW.^3.*XX.*YY+(-2).* ...

phi.^2.*WW.^3.*XX.*YY+phi.*WW.^4.*XX.*YY+phi.^2.*WW.^4.*XX.*YY+phi.*WW.* ...

ZZ+(-1).*phi.^2.*WW.*ZZ+(-3).*WW.^2.*ZZ+4.*phi.*WW.^2.*ZZ+2.*phi.^2.* ...

WW.^2.*ZZ+(-3).*WW.^3.*ZZ+(-5).*phi.*WW.^3.*ZZ+(-1).*phi.^2.*WW.^3.*ZZ+ ...

2.*WW.*XX.*ZZ+(-6).*phi.*WW.*XX.*ZZ+2.*phi.^2.*WW.*XX.*ZZ+5.*WW.^2.*XX.* ...

ZZ+5.*phi.*WW.^2.*XX.*ZZ+(-5).*phi.^2.*WW.^2.*XX.*ZZ+(-1).*WW.^3.*XX.* ...

ZZ+2.*phi.*WW.^3.*XX.*ZZ+4.*phi.^2.*WW.^3.*XX.*ZZ+(-1).*phi.*WW.^4.*XX.* ...

ZZ+(-1).*phi.^2.*WW.^4.*XX.*ZZ+(-2).*phi.*WW.^2.*YY.*ZZ+phi.^2.*WW.^2.* ...

YY.*ZZ+3.*WW.^3.*YY.*ZZ+(-2).*phi.*WW.^3.*YY.*ZZ+(-2).*phi.^2.*WW.^3.* ...

YY.*ZZ+3.*WW.^4.*YY.*ZZ+4.*phi.*WW.^4.*YY.*ZZ+phi.^2.*WW.^4.*YY.*ZZ+2.* ...

phi.*WW.*XX.*YY.*ZZ+(-1).*phi.^2.*WW.*XX.*YY.*ZZ+(-3).*WW.^2.*XX.*YY.* ...

ZZ+2.*phi.*WW.^2.*XX.*YY.*ZZ+2.*phi.^2.*WW.^2.*XX.*YY.*ZZ+(-3).*WW.^3.* ...

XX.*YY.*ZZ+(-4).*phi.*WW.^3.*XX.*YY.*ZZ+(-1).*phi.^2.*WW.^3.*XX.*YY.*ZZ) ...

.^3+(-9).*((-3).*WW.^3+2.*phi.*WW.^3+(-1).*WW.^4+(-2).*phi.*WW.^4+3.* ...

WW.^2.*XX+(-2).*phi.*WW.^2.*XX+WW.^3.*XX+2.*phi.*WW.^3.*XX+(-1).*WW.^2.* ...

YY+2.*phi.*WW.^2.*YY+WW.^3.*YY+(-6).*phi.*WW.^3.*YY+4.*WW.^4.*YY+4.* ...

phi.*WW.^4.*YY+(-3).*WW.^3.*XX.*YY+2.*phi.*WW.^3.*XX.*YY+(-1).*WW.^4.* ...

XX.*YY+(-2).*phi.*WW.^4.*XX.*YY+WW.^2.*ZZ+(-2).*phi.*WW.^2.*ZZ+3.* ...

WW.^3.*ZZ+2.*phi.*WW.^3.*ZZ+(-4).*WW.^2.*XX.*ZZ+4.*phi.*WW.^2.*XX.*ZZ+( ...

-1).*WW.^3.*XX.*ZZ+(-6).*phi.*WW.^3.*XX.*ZZ+WW.^4.*XX.*ZZ+2.*phi.* ...

WW.^4.*XX.*ZZ+(-1).*WW.^3.*YY.*ZZ+2.*phi.*WW.^3.*YY.*ZZ+(-3).*WW.^4.* ...

YY.*ZZ+(-2).*phi.*WW.^4.*YY.*ZZ+WW.^2.*XX.*YY.*ZZ+(-2).*phi.*WW.^2.*XX.* ...

YY.*ZZ+3.*WW.^3.*XX.*YY.*ZZ+2.*phi.*WW.^3.*XX.*YY.*ZZ).*(3.*WW.^2+(-4).* ...

phi.*WW.^2+phi.^2.*WW.^2+3.*WW.^3+2.*phi.*WW.^3+(-2).*phi.^2.*WW.^3+2.* ...

phi.*WW.^4+phi.^2.*WW.^4+(-3).*WW.*XX+4.*phi.*WW.*XX+(-1).*phi.^2.*WW.* ...

XX+(-3).*WW.^2.*XX+(-2).*phi.*WW.^2.*XX+2.*phi.^2.*WW.^2.*XX+(-2).*phi.* ...

WW.^3.*XX+(-1).*phi.^2.*WW.^3.*XX+(-1).*phi.*WW.*YY+phi.^2.*WW.*YY+ ...

WW.^2.*YY+2.*phi.*WW.^2.*YY+(-4).*phi.^2.*WW.^2.*YY+(-5).*WW.^3.*YY+5.* ...

phi.*WW.^3.*YY+5.*phi.^2.*WW.^3.*YY+(-2).*WW.^4.*YY+(-6).*phi.*WW.^4.* ...

YY+(-2).*phi.^2.*WW.^4.*YY+3.*WW.^2.*XX.*YY+(-5).*phi.*WW.^2.*XX.*YY+ ...

phi.^2.*WW.^2.*XX.*YY+3.*WW.^3.*XX.*YY+4.*phi.*WW.^3.*XX.*YY+(-2).* ...

phi.^2.*WW.^3.*XX.*YY+phi.*WW.^4.*XX.*YY+phi.^2.*WW.^4.*XX.*YY+phi.*WW.* ...

ZZ+(-1).*phi.^2.*WW.*ZZ+(-3).*WW.^2.*ZZ+4.*phi.*WW.^2.*ZZ+2.*phi.^2.* ...

WW.^2.*ZZ+(-3).*WW.^3.*ZZ+(-5).*phi.*WW.^3.*ZZ+(-1).*phi.^2.*WW.^3.*ZZ+ ...

2.*WW.*XX.*ZZ+(-6).*phi.*WW.*XX.*ZZ+2.*phi.^2.*WW.*XX.*ZZ+5.*WW.^2.*XX.* ...

ZZ+5.*phi.*WW.^2.*XX.*ZZ+(-5).*phi.^2.*WW.^2.*XX.*ZZ+(-1).*WW.^3.*XX.* ...

ZZ+2.*phi.*WW.^3.*XX.*ZZ+4.*phi.^2.*WW.^3.*XX.*ZZ+(-1).*phi.*WW.^4.*XX.* ...

ZZ+(-1).*phi.^2.*WW.^4.*XX.*ZZ+(-2).*phi.*WW.^2.*YY.*ZZ+phi.^2.*WW.^2.* ...

YY.*ZZ+3.*WW.^3.*YY.*ZZ+(-2).*phi.*WW.^3.*YY.*ZZ+(-2).*phi.^2.*WW.^3.* ...

YY.*ZZ+3.*WW.^4.*YY.*ZZ+4.*phi.*WW.^4.*YY.*ZZ+phi.^2.*WW.^4.*YY.*ZZ+2.* ...

phi.*WW.*XX.*YY.*ZZ+(-1).*phi.^2.*WW.*XX.*YY.*ZZ+(-3).*WW.^2.*XX.*YY.* ...

ZZ+2.*phi.*WW.^2.*XX.*YY.*ZZ+2.*phi.^2.*WW.^2.*XX.*YY.*ZZ+(-3).*WW.^3.* ...

XX.*YY.*ZZ+(-4).*phi.*WW.^3.*XX.*YY.*ZZ+(-1).*phi.^2.*WW.^3.*XX.*YY.*ZZ) ...

.*((-1).*WW+2.*phi.*WW+(-1).*phi.^2.*WW+(-3).*WW.^2+2.*phi.*WW.^2+ ...

phi.^2.*WW.^2+(-4).*phi.*WW.^3+phi.^2.*WW.^3+(-1).*phi.^2.*WW.^4+XX+(-2) ...

.*phi.*XX+phi.^2.*XX+3.*WW.*XX+(-2).*phi.*WW.*XX+(-1).*phi.^2.*WW.*XX+ ...

4.*phi.*WW.^2.*XX+(-1).*phi.^2.*WW.^2.*XX+phi.^2.*WW.^3.*XX+WW.^2.*YY+( ...

-4).*phi.*WW.^2.*YY+2.*phi.^2.*WW.^2.*YY+3.*WW.^3.*YY+2.*phi.*WW.^3.*YY+ ...

(-4).*phi.^2.*WW.^3.*YY+2.*phi.*WW.^4.*YY+2.*phi.^2.*WW.^4.*YY+(-1).* ...

WW.*XX.*YY+4.*phi.*WW.*XX.*YY+(-2).*phi.^2.*WW.*XX.*YY+(-3).*WW.^2.*XX.* ...

YY+(-2).*phi.*WW.^2.*XX.*YY+4.*phi.^2.*WW.^2.*XX.*YY+(-2).*phi.*WW.^3.* ...

XX.*YY+(-2).*phi.^2.*WW.^3.*XX.*YY+(-2).*phi.*WW.*ZZ+2.*phi.^2.*WW.*ZZ+ ...

3.*WW.^2.*ZZ+(-2).*phi.*WW.^2.*ZZ+(-4).*phi.^2.*WW.^2.*ZZ+WW.^3.*ZZ+4.* ...

phi.*WW.^3.*ZZ+2.*phi.^2.*WW.^3.*ZZ+2.*phi.*XX.*ZZ+(-2).*phi.^2.*XX.*ZZ+ ...

(-3).*WW.*XX.*ZZ+2.*phi.*WW.*XX.*ZZ+4.*phi.^2.*WW.*XX.*ZZ+(-1).*WW.^2.* ...

XX.*ZZ+(-4).*phi.*WW.^2.*XX.*ZZ+(-2).*phi.^2.*WW.^2.*XX.*ZZ+(-1).* ...

phi.^2.*WW.*YY.*ZZ+4.*phi.*WW.^2.*YY.*ZZ+phi.^2.*WW.^2.*YY.*ZZ+(-3).* ...

WW.^3.*YY.*ZZ+(-2).*phi.*WW.^3.*YY.*ZZ+phi.^2.*WW.^3.*YY.*ZZ+(-1).* ...

WW.^4.*YY.*ZZ+(-2).*phi.*WW.^4.*YY.*ZZ+(-1).*phi.^2.*WW.^4.*YY.*ZZ+ ...

phi.^2.*XX.*YY.*ZZ+(-4).*phi.*WW.*XX.*YY.*ZZ+(-1).*phi.^2.*WW.*XX.*YY.* ...

ZZ+3.*WW.^2.*XX.*YY.*ZZ+2.*phi.*WW.^2.*XX.*YY.*ZZ+(-1).*phi.^2.*WW.^2.* ...

XX.*YY.*ZZ+WW.^3.*XX.*YY.*ZZ+2.*phi.*WW.^3.*XX.*YY.*ZZ+phi.^2.*WW.^3.* ...

XX.*YY.*ZZ)+27.*(WW.^4+(-1).*WW.^3.*XX+WW.^3.*YY+(-2).*WW.^4.*YY+WW.^4.* ...

XX.*YY+(-1).*WW.^3.*ZZ+2.*WW.^3.*XX.*ZZ+(-1).*WW.^4.*XX.*ZZ+WW.^4.*YY.* ...

ZZ+(-1).*WW.^3.*XX.*YY.*ZZ).*((-1).*WW+2.*phi.*WW+(-1).*phi.^2.*WW+(-3) ...

.*WW.^2+2.*phi.*WW.^2+phi.^2.*WW.^2+(-4).*phi.*WW.^3+phi.^2.*WW.^3+(-1) ...

.*phi.^2.*WW.^4+XX+(-2).*phi.*XX+phi.^2.*XX+3.*WW.*XX+(-2).*phi.*WW.*XX+ ...

(-1).*phi.^2.*WW.*XX+4.*phi.*WW.^2.*XX+(-1).*phi.^2.*WW.^2.*XX+phi.^2.* ...

WW.^3.*XX+WW.^2.*YY+(-4).*phi.*WW.^2.*YY+2.*phi.^2.*WW.^2.*YY+3.*WW.^3.* ...

YY+2.*phi.*WW.^3.*YY+(-4).*phi.^2.*WW.^3.*YY+2.*phi.*WW.^4.*YY+2.* ...

phi.^2.*WW.^4.*YY+(-1).*WW.*XX.*YY+4.*phi.*WW.*XX.*YY+(-2).*phi.^2.*WW.* ...

XX.*YY+(-3).*WW.^2.*XX.*YY+(-2).*phi.*WW.^2.*XX.*YY+4.*phi.^2.*WW.^2.* ...

XX.*YY+(-2).*phi.*WW.^3.*XX.*YY+(-2).*phi.^2.*WW.^3.*XX.*YY+(-2).*phi.* ...

WW.*ZZ+2.*phi.^2.*WW.*ZZ+3.*WW.^2.*ZZ+(-2).*phi.*WW.^2.*ZZ+(-4).* ...

phi.^2.*WW.^2.*ZZ+WW.^3.*ZZ+4.*phi.*WW.^3.*ZZ+2.*phi.^2.*WW.^3.*ZZ+2.* ...

phi.*XX.*ZZ+(-2).*phi.^2.*XX.*ZZ+(-3).*WW.*XX.*ZZ+2.*phi.*WW.*XX.*ZZ+4.* ...

phi.^2.*WW.*XX.*ZZ+(-1).*WW.^2.*XX.*ZZ+(-4).*phi.*WW.^2.*XX.*ZZ+(-2).* ...

phi.^2.*WW.^2.*XX.*ZZ+(-1).*phi.^2.*WW.*YY.*ZZ+4.*phi.*WW.^2.*YY.*ZZ+ ...

phi.^2.*WW.^2.*YY.*ZZ+(-3).*WW.^3.*YY.*ZZ+(-2).*phi.*WW.^3.*YY.*ZZ+ ...

phi.^2.*WW.^3.*YY.*ZZ+(-1).*WW.^4.*YY.*ZZ+(-2).*phi.*WW.^4.*YY.*ZZ+(-1) ...

.*phi.^2.*WW.^4.*YY.*ZZ+phi.^2.*XX.*YY.*ZZ+(-4).*phi.*WW.*XX.*YY.*ZZ+( ...

-1).*phi.^2.*WW.*XX.*YY.*ZZ+3.*WW.^2.*XX.*YY.*ZZ+2.*phi.*WW.^2.*XX.*YY.* ...

ZZ+(-1).*phi.^2.*WW.^2.*XX.*YY.*ZZ+WW.^3.*XX.*YY.*ZZ+2.*phi.*WW.^3.*XX.* ...

YY.*ZZ+phi.^2.*WW.^3.*XX.*YY.*ZZ).^2+((-4).*(12.*(WW+(-2).*phi.*WW+ ...

phi.^2.*WW+2.*phi.*WW.^2+(-2).*phi.^2.*WW.^2+phi.^2.*WW.^3+(-1).*XX+2.* ...

phi.*XX+(-1).*phi.^2.*XX+(-2).*phi.*WW.*XX+2.*phi.^2.*WW.*XX+(-1).* ...

phi.^2.*WW.^2.*XX+phi.*WW.*YY+(-1).*phi.^2.*WW.*YY+(-1).*WW.^2.*YY+2.* ...

phi.^2.*WW.^2.*YY+(-1).*phi.*WW.^3.*YY+(-1).*phi.^2.*WW.^3.*YY+(-1).* ...

phi.*XX.*YY+phi.^2.*XX.*YY+WW.*XX.*YY+(-2).*phi.^2.*WW.*XX.*YY+phi.* ...

WW.^2.*XX.*YY+phi.^2.*WW.^2.*XX.*YY+phi.*WW.*ZZ+(-1).*phi.^2.*WW.*ZZ+( ...

-1).*WW.^2.*ZZ+2.*phi.^2.*WW.^2.*ZZ+(-1).*phi.*WW.^3.*ZZ+(-1).*phi.^2.* ...

WW.^3.*ZZ+(-1).*phi.*XX.*ZZ+phi.^2.*XX.*ZZ+WW.*XX.*ZZ+(-2).*phi.^2.*WW.* ...

XX.*ZZ+phi.*WW.^2.*XX.*ZZ+phi.^2.*WW.^2.*XX.*ZZ+phi.^2.*WW.*YY.*ZZ+(-2) ...

.*phi.*WW.^2.*YY.*ZZ+(-2).*phi.^2.*WW.^2.*YY.*ZZ+WW.^3.*YY.*ZZ+2.*phi.* ...

WW.^3.*YY.*ZZ+phi.^2.*WW.^3.*YY.*ZZ+(-1).*phi.^2.*XX.*YY.*ZZ+2.*phi.* ...

WW.*XX.*YY.*ZZ+2.*phi.^2.*WW.*XX.*YY.*ZZ+(-1).*WW.^2.*XX.*YY.*ZZ+(-2).* ...

phi.*WW.^2.*XX.*YY.*ZZ+(-1).*phi.^2.*WW.^2.*XX.*YY.*ZZ).*(WW.^4+(-1).* ...

WW.^3.*XX+WW.^3.*YY+(-2).*WW.^4.*YY+WW.^4.*XX.*YY+(-1).*WW.^3.*ZZ+2.* ...

WW.^3.*XX.*ZZ+(-1).*WW.^4.*XX.*ZZ+WW.^4.*YY.*ZZ+(-1).*WW.^3.*XX.*YY.*ZZ) ...

+(3.*WW.^2+(-4).*phi.*WW.^2+phi.^2.*WW.^2+3.*WW.^3+2.*phi.*WW.^3+(-2).* ...

phi.^2.*WW.^3+2.*phi.*WW.^4+phi.^2.*WW.^4+(-3).*WW.*XX+4.*phi.*WW.*XX+( ...

-1).*phi.^2.*WW.*XX+(-3).*WW.^2.*XX+(-2).*phi.*WW.^2.*XX+2.*phi.^2.* ...

WW.^2.*XX+(-2).*phi.*WW.^3.*XX+(-1).*phi.^2.*WW.^3.*XX+(-1).*phi.*WW.* ...

YY+phi.^2.*WW.*YY+WW.^2.*YY+2.*phi.*WW.^2.*YY+(-4).*phi.^2.*WW.^2.*YY+( ...

-5).*WW.^3.*YY+5.*phi.*WW.^3.*YY+5.*phi.^2.*WW.^3.*YY+(-2).*WW.^4.*YY+( ...

-6).*phi.*WW.^4.*YY+(-2).*phi.^2.*WW.^4.*YY+3.*WW.^2.*XX.*YY+(-5).*phi.* ...

WW.^2.*XX.*YY+phi.^2.*WW.^2.*XX.*YY+3.*WW.^3.*XX.*YY+4.*phi.*WW.^3.*XX.* ...

YY+(-2).*phi.^2.*WW.^3.*XX.*YY+phi.*WW.^4.*XX.*YY+phi.^2.*WW.^4.*XX.*YY+ ...

phi.*WW.*ZZ+(-1).*phi.^2.*WW.*ZZ+(-3).*WW.^2.*ZZ+4.*phi.*WW.^2.*ZZ+2.* ...

phi.^2.*WW.^2.*ZZ+(-3).*WW.^3.*ZZ+(-5).*phi.*WW.^3.*ZZ+(-1).*phi.^2.* ...

WW.^3.*ZZ+2.*WW.*XX.*ZZ+(-6).*phi.*WW.*XX.*ZZ+2.*phi.^2.*WW.*XX.*ZZ+5.* ...

WW.^2.*XX.*ZZ+5.*phi.*WW.^2.*XX.*ZZ+(-5).*phi.^2.*WW.^2.*XX.*ZZ+(-1).* ...

WW.^3.*XX.*ZZ+2.*phi.*WW.^3.*XX.*ZZ+4.*phi.^2.*WW.^3.*XX.*ZZ+(-1).*phi.* ...

WW.^4.*XX.*ZZ+(-1).*phi.^2.*WW.^4.*XX.*ZZ+(-2).*phi.*WW.^2.*YY.*ZZ+ ...

phi.^2.*WW.^2.*YY.*ZZ+3.*WW.^3.*YY.*ZZ+(-2).*phi.*WW.^3.*YY.*ZZ+(-2).* ...

phi.^2.*WW.^3.*YY.*ZZ+3.*WW.^4.*YY.*ZZ+4.*phi.*WW.^4.*YY.*ZZ+phi.^2.* ...

WW.^4.*YY.*ZZ+2.*phi.*WW.*XX.*YY.*ZZ+(-1).*phi.^2.*WW.*XX.*YY.*ZZ+(-3).* ...

WW.^2.*XX.*YY.*ZZ+2.*phi.*WW.^2.*XX.*YY.*ZZ+2.*phi.^2.*WW.^2.*XX.*YY.* ...

ZZ+(-3).*WW.^3.*XX.*YY.*ZZ+(-4).*phi.*WW.^3.*XX.*YY.*ZZ+(-1).*phi.^2.* ...

WW.^3.*XX.*YY.*ZZ).^2+(-3).*((-3).*WW.^3+2.*phi.*WW.^3+(-1).*WW.^4+(-2) ...

.*phi.*WW.^4+3.*WW.^2.*XX+(-2).*phi.*WW.^2.*XX+WW.^3.*XX+2.*phi.*WW.^3.* ...

XX+(-1).*WW.^2.*YY+2.*phi.*WW.^2.*YY+WW.^3.*YY+(-6).*phi.*WW.^3.*YY+4.* ...

WW.^4.*YY+4.*phi.*WW.^4.*YY+(-3).*WW.^3.*XX.*YY+2.*phi.*WW.^3.*XX.*YY+( ...

-1).*WW.^4.*XX.*YY+(-2).*phi.*WW.^4.*XX.*YY+WW.^2.*ZZ+(-2).*phi.*WW.^2.* ...

ZZ+3.*WW.^3.*ZZ+2.*phi.*WW.^3.*ZZ+(-4).*WW.^2.*XX.*ZZ+4.*phi.*WW.^2.* ...

XX.*ZZ+(-1).*WW.^3.*XX.*ZZ+(-6).*phi.*WW.^3.*XX.*ZZ+WW.^4.*XX.*ZZ+2.* ...

phi.*WW.^4.*XX.*ZZ+(-1).*WW.^3.*YY.*ZZ+2.*phi.*WW.^3.*YY.*ZZ+(-3).* ...

WW.^4.*YY.*ZZ+(-2).*phi.*WW.^4.*YY.*ZZ+WW.^2.*XX.*YY.*ZZ+(-2).*phi.* ...

WW.^2.*XX.*YY.*ZZ+3.*WW.^3.*XX.*YY.*ZZ+2.*phi.*WW.^3.*XX.*YY.*ZZ).*((-1) ...

.*WW+2.*phi.*WW+(-1).*phi.^2.*WW+(-3).*WW.^2+2.*phi.*WW.^2+phi.^2.* ...

WW.^2+(-4).*phi.*WW.^3+phi.^2.*WW.^3+(-1).*phi.^2.*WW.^4+XX+(-2).*phi.* ...

XX+phi.^2.*XX+3.*WW.*XX+(-2).*phi.*WW.*XX+(-1).*phi.^2.*WW.*XX+4.*phi.* ...

WW.^2.*XX+(-1).*phi.^2.*WW.^2.*XX+phi.^2.*WW.^3.*XX+WW.^2.*YY+(-4).* ...

phi.*WW.^2.*YY+2.*phi.^2.*WW.^2.*YY+3.*WW.^3.*YY+2.*phi.*WW.^3.*YY+(-4) ...

.*phi.^2.*WW.^3.*YY+2.*phi.*WW.^4.*YY+2.*phi.^2.*WW.^4.*YY+(-1).*WW.* ...

XX.*YY+4.*phi.*WW.*XX.*YY+(-2).*phi.^2.*WW.*XX.*YY+(-3).*WW.^2.*XX.*YY+( ...

-2).*phi.*WW.^2.*XX.*YY+4.*phi.^2.*WW.^2.*XX.*YY+(-2).*phi.*WW.^3.*XX.* ...

YY+(-2).*phi.^2.*WW.^3.*XX.*YY+(-2).*phi.*WW.*ZZ+2.*phi.^2.*WW.*ZZ+3.* ...

WW.^2.*ZZ+(-2).*phi.*WW.^2.*ZZ+(-4).*phi.^2.*WW.^2.*ZZ+WW.^3.*ZZ+4.* ...

phi.*WW.^3.*ZZ+2.*phi.^2.*WW.^3.*ZZ+2.*phi.*XX.*ZZ+(-2).*phi.^2.*XX.*ZZ+ ...

(-3).*WW.*XX.*ZZ+2.*phi.*WW.*XX.*ZZ+4.*phi.^2.*WW.*XX.*ZZ+(-1).*WW.^2.* ...

XX.*ZZ+(-4).*phi.*WW.^2.*XX.*ZZ+(-2).*phi.^2.*WW.^2.*XX.*ZZ+(-1).* ...

phi.^2.*WW.*YY.*ZZ+4.*phi.*WW.^2.*YY.*ZZ+phi.^2.*WW.^2.*YY.*ZZ+(-3).* ...

WW.^3.*YY.*ZZ+(-2).*phi.*WW.^3.*YY.*ZZ+phi.^2.*WW.^3.*YY.*ZZ+(-1).* ...

WW.^4.*YY.*ZZ+(-2).*phi.*WW.^4.*YY.*ZZ+(-1).*phi.^2.*WW.^4.*YY.*ZZ+ ...

phi.^2.*XX.*YY.*ZZ+(-4).*phi.*WW.*XX.*YY.*ZZ+(-1).*phi.^2.*WW.*XX.*YY.* ...

ZZ+3.*WW.^2.*XX.*YY.*ZZ+2.*phi.*WW.^2.*XX.*YY.*ZZ+(-1).*phi.^2.*WW.^2.* ...

XX.*YY.*ZZ+WW.^3.*XX.*YY.*ZZ+2.*phi.*WW.^3.*XX.*YY.*ZZ+phi.^2.*WW.^3.* ...

XX.*YY.*ZZ)).^3+(27.*(WW+(-2).*phi.*WW+phi.^2.*WW+2.*phi.*WW.^2+(-2).* ...

phi.^2.*WW.^2+phi.^2.*WW.^3+(-1).*XX+2.*phi.*XX+(-1).*phi.^2.*XX+(-2).* ...

phi.*WW.*XX+2.*phi.^2.*WW.*XX+(-1).*phi.^2.*WW.^2.*XX+phi.*WW.*YY+(-1).* ...

phi.^2.*WW.*YY+(-1).*WW.^2.*YY+2.*phi.^2.*WW.^2.*YY+(-1).*phi.*WW.^3.* ...

YY+(-1).*phi.^2.*WW.^3.*YY+(-1).*phi.*XX.*YY+phi.^2.*XX.*YY+WW.*XX.*YY+( ...

-2).*phi.^2.*WW.*XX.*YY+phi.*WW.^2.*XX.*YY+phi.^2.*WW.^2.*XX.*YY+phi.* ...

WW.*ZZ+(-1).*phi.^2.*WW.*ZZ+(-1).*WW.^2.*ZZ+2.*phi.^2.*WW.^2.*ZZ+(-1).* ...

phi.*WW.^3.*ZZ+(-1).*phi.^2.*WW.^3.*ZZ+(-1).*phi.*XX.*ZZ+phi.^2.*XX.*ZZ+ ...

WW.*XX.*ZZ+(-2).*phi.^2.*WW.*XX.*ZZ+phi.*WW.^2.*XX.*ZZ+phi.^2.*WW.^2.* ...

XX.*ZZ+phi.^2.*WW.*YY.*ZZ+(-2).*phi.*WW.^2.*YY.*ZZ+(-2).*phi.^2.*WW.^2.* ...

YY.*ZZ+WW.^3.*YY.*ZZ+2.*phi.*WW.^3.*YY.*ZZ+phi.^2.*WW.^3.*YY.*ZZ+(-1).* ...

phi.^2.*XX.*YY.*ZZ+2.*phi.*WW.*XX.*YY.*ZZ+2.*phi.^2.*WW.*XX.*YY.*ZZ+(-1) ...

.*WW.^2.*XX.*YY.*ZZ+(-2).*phi.*WW.^2.*XX.*YY.*ZZ+(-1).*phi.^2.*WW.^2.* ...

XX.*YY.*ZZ).*((-3).*WW.^3+2.*phi.*WW.^3+(-1).*WW.^4+(-2).*phi.*WW.^4+3.* ...

WW.^2.*XX+(-2).*phi.*WW.^2.*XX+WW.^3.*XX+2.*phi.*WW.^3.*XX+(-1).*WW.^2.* ...

YY+2.*phi.*WW.^2.*YY+WW.^3.*YY+(-6).*phi.*WW.^3.*YY+4.*WW.^4.*YY+4.* ...

phi.*WW.^4.*YY+(-3).*WW.^3.*XX.*YY+2.*phi.*WW.^3.*XX.*YY+(-1).*WW.^4.* ...

XX.*YY+(-2).*phi.*WW.^4.*XX.*YY+WW.^2.*ZZ+(-2).*phi.*WW.^2.*ZZ+3.* ...

WW.^3.*ZZ+2.*phi.*WW.^3.*ZZ+(-4).*WW.^2.*XX.*ZZ+4.*phi.*WW.^2.*XX.*ZZ+( ...

-1).*WW.^3.*XX.*ZZ+(-6).*phi.*WW.^3.*XX.*ZZ+WW.^4.*XX.*ZZ+2.*phi.* ...

WW.^4.*XX.*ZZ+(-1).*WW.^3.*YY.*ZZ+2.*phi.*WW.^3.*YY.*ZZ+(-3).*WW.^4.* ...

YY.*ZZ+(-2).*phi.*WW.^4.*YY.*ZZ+WW.^2.*XX.*YY.*ZZ+(-2).*phi.*WW.^2.*XX.* ...

YY.*ZZ+3.*WW.^3.*XX.*YY.*ZZ+2.*phi.*WW.^3.*XX.*YY.*ZZ).^2+(-72).*(WW+( ...

-2).*phi.*WW+phi.^2.*WW+2.*phi.*WW.^2+(-2).*phi.^2.*WW.^2+phi.^2.*WW.^3+ ...

(-1).*XX+2.*phi.*XX+(-1).*phi.^2.*XX+(-2).*phi.*WW.*XX+2.*phi.^2.*WW.* ...

XX+(-1).*phi.^2.*WW.^2.*XX+phi.*WW.*YY+(-1).*phi.^2.*WW.*YY+(-1).* ...

WW.^2.*YY+2.*phi.^2.*WW.^2.*YY+(-1).*phi.*WW.^3.*YY+(-1).*phi.^2.* ...

WW.^3.*YY+(-1).*phi.*XX.*YY+phi.^2.*XX.*YY+WW.*XX.*YY+(-2).*phi.^2.*WW.* ...

XX.*YY+phi.*WW.^2.*XX.*YY+phi.^2.*WW.^2.*XX.*YY+phi.*WW.*ZZ+(-1).* ...

phi.^2.*WW.*ZZ+(-1).*WW.^2.*ZZ+2.*phi.^2.*WW.^2.*ZZ+(-1).*phi.*WW.^3.* ...

ZZ+(-1).*phi.^2.*WW.^3.*ZZ+(-1).*phi.*XX.*ZZ+phi.^2.*XX.*ZZ+WW.*XX.*ZZ+( ...

-2).*phi.^2.*WW.*XX.*ZZ+phi.*WW.^2.*XX.*ZZ+phi.^2.*WW.^2.*XX.*ZZ+ ...

phi.^2.*WW.*YY.*ZZ+(-2).*phi.*WW.^2.*YY.*ZZ+(-2).*phi.^2.*WW.^2.*YY.*ZZ+ ...

WW.^3.*YY.*ZZ+2.*phi.*WW.^3.*YY.*ZZ+phi.^2.*WW.^3.*YY.*ZZ+(-1).*phi.^2.* ...

XX.*YY.*ZZ+2.*phi.*WW.*XX.*YY.*ZZ+2.*phi.^2.*WW.*XX.*YY.*ZZ+(-1).* ...

WW.^2.*XX.*YY.*ZZ+(-2).*phi.*WW.^2.*XX.*YY.*ZZ+(-1).*phi.^2.*WW.^2.*XX.* ...

YY.*ZZ).*(WW.^4+(-1).*WW.^3.*XX+WW.^3.*YY+(-2).*WW.^4.*YY+WW.^4.*XX.*YY+ ...

(-1).*WW.^3.*ZZ+2.*WW.^3.*XX.*ZZ+(-1).*WW.^4.*XX.*ZZ+WW.^4.*YY.*ZZ+(-1) ...

.*WW.^3.*XX.*YY.*ZZ).*(3.*WW.^2+(-4).*phi.*WW.^2+phi.^2.*WW.^2+3.*WW.^3+ ...

2.*phi.*WW.^3+(-2).*phi.^2.*WW.^3+2.*phi.*WW.^4+phi.^2.*WW.^4+(-3).*WW.* ...

XX+4.*phi.*WW.*XX+(-1).*phi.^2.*WW.*XX+(-3).*WW.^2.*XX+(-2).*phi.* ...

WW.^2.*XX+2.*phi.^2.*WW.^2.*XX+(-2).*phi.*WW.^3.*XX+(-1).*phi.^2.* ...

WW.^3.*XX+(-1).*phi.*WW.*YY+phi.^2.*WW.*YY+WW.^2.*YY+2.*phi.*WW.^2.*YY+( ...

-4).*phi.^2.*WW.^2.*YY+(-5).*WW.^3.*YY+5.*phi.*WW.^3.*YY+5.*phi.^2.* ...

WW.^3.*YY+(-2).*WW.^4.*YY+(-6).*phi.*WW.^4.*YY+(-2).*phi.^2.*WW.^4.*YY+ ...

3.*WW.^2.*XX.*YY+(-5).*phi.*WW.^2.*XX.*YY+phi.^2.*WW.^2.*XX.*YY+3.* ...

WW.^3.*XX.*YY+4.*phi.*WW.^3.*XX.*YY+(-2).*phi.^2.*WW.^3.*XX.*YY+phi.* ...

WW.^4.*XX.*YY+phi.^2.*WW.^4.*XX.*YY+phi.*WW.*ZZ+(-1).*phi.^2.*WW.*ZZ+( ...

-3).*WW.^2.*ZZ+4.*phi.*WW.^2.*ZZ+2.*phi.^2.*WW.^2.*ZZ+(-3).*WW.^3.*ZZ+( ...

-5).*phi.*WW.^3.*ZZ+(-1).*phi.^2.*WW.^3.*ZZ+2.*WW.*XX.*ZZ+(-6).*phi.* ...

WW.*XX.*ZZ+2.*phi.^2.*WW.*XX.*ZZ+5.*WW.^2.*XX.*ZZ+5.*phi.*WW.^2.*XX.*ZZ+ ...

(-5).*phi.^2.*WW.^2.*XX.*ZZ+(-1).*WW.^3.*XX.*ZZ+2.*phi.*WW.^3.*XX.*ZZ+ ...

4.*phi.^2.*WW.^3.*XX.*ZZ+(-1).*phi.*WW.^4.*XX.*ZZ+(-1).*phi.^2.*WW.^4.* ...

XX.*ZZ+(-2).*phi.*WW.^2.*YY.*ZZ+phi.^2.*WW.^2.*YY.*ZZ+3.*WW.^3.*YY.*ZZ+( ...

-2).*phi.*WW.^3.*YY.*ZZ+(-2).*phi.^2.*WW.^3.*YY.*ZZ+3.*WW.^4.*YY.*ZZ+4.* ...

phi.*WW.^4.*YY.*ZZ+phi.^2.*WW.^4.*YY.*ZZ+2.*phi.*WW.*XX.*YY.*ZZ+(-1).* ...

phi.^2.*WW.*XX.*YY.*ZZ+(-3).*WW.^2.*XX.*YY.*ZZ+2.*phi.*WW.^2.*XX.*YY.* ...

ZZ+2.*phi.^2.*WW.^2.*XX.*YY.*ZZ+(-3).*WW.^3.*XX.*YY.*ZZ+(-4).*phi.* ...

WW.^3.*XX.*YY.*ZZ+(-1).*phi.^2.*WW.^3.*XX.*YY.*ZZ)+2.*(3.*WW.^2+(-4).* ...

phi.*WW.^2+phi.^2.*WW.^2+3.*WW.^3+2.*phi.*WW.^3+(-2).*phi.^2.*WW.^3+2.* ...

phi.*WW.^4+phi.^2.*WW.^4+(-3).*WW.*XX+4.*phi.*WW.*XX+(-1).*phi.^2.*WW.* ...

XX+(-3).*WW.^2.*XX+(-2).*phi.*WW.^2.*XX+2.*phi.^2.*WW.^2.*XX+(-2).*phi.* ...

WW.^3.*XX+(-1).*phi.^2.*WW.^3.*XX+(-1).*phi.*WW.*YY+phi.^2.*WW.*YY+ ...

WW.^2.*YY+2.*phi.*WW.^2.*YY+(-4).*phi.^2.*WW.^2.*YY+(-5).*WW.^3.*YY+5.* ...

phi.*WW.^3.*YY+5.*phi.^2.*WW.^3.*YY+(-2).*WW.^4.*YY+(-6).*phi.*WW.^4.* ...

YY+(-2).*phi.^2.*WW.^4.*YY+3.*WW.^2.*XX.*YY+(-5).*phi.*WW.^2.*XX.*YY+ ...

phi.^2.*WW.^2.*XX.*YY+3.*WW.^3.*XX.*YY+4.*phi.*WW.^3.*XX.*YY+(-2).* ...

phi.^2.*WW.^3.*XX.*YY+phi.*WW.^4.*XX.*YY+phi.^2.*WW.^4.*XX.*YY+phi.*WW.* ...

ZZ+(-1).*phi.^2.*WW.*ZZ+(-3).*WW.^2.*ZZ+4.*phi.*WW.^2.*ZZ+2.*phi.^2.* ...

WW.^2.*ZZ+(-3).*WW.^3.*ZZ+(-5).*phi.*WW.^3.*ZZ+(-1).*phi.^2.*WW.^3.*ZZ+ ...

2.*WW.*XX.*ZZ+(-6).*phi.*WW.*XX.*ZZ+2.*phi.^2.*WW.*XX.*ZZ+5.*WW.^2.*XX.* ...

ZZ+5.*phi.*WW.^2.*XX.*ZZ+(-5).*phi.^2.*WW.^2.*XX.*ZZ+(-1).*WW.^3.*XX.* ...

ZZ+2.*phi.*WW.^3.*XX.*ZZ+4.*phi.^2.*WW.^3.*XX.*ZZ+(-1).*phi.*WW.^4.*XX.* ...

ZZ+(-1).*phi.^2.*WW.^4.*XX.*ZZ+(-2).*phi.*WW.^2.*YY.*ZZ+phi.^2.*WW.^2.* ...

YY.*ZZ+3.*WW.^3.*YY.*ZZ+(-2).*phi.*WW.^3.*YY.*ZZ+(-2).*phi.^2.*WW.^3.* ...

YY.*ZZ+3.*WW.^4.*YY.*ZZ+4.*phi.*WW.^4.*YY.*ZZ+phi.^2.*WW.^4.*YY.*ZZ+2.* ...

phi.*WW.*XX.*YY.*ZZ+(-1).*phi.^2.*WW.*XX.*YY.*ZZ+(-3).*WW.^2.*XX.*YY.* ...

ZZ+2.*phi.*WW.^2.*XX.*YY.*ZZ+2.*phi.^2.*WW.^2.*XX.*YY.*ZZ+(-3).*WW.^3.* ...

XX.*YY.*ZZ+(-4).*phi.*WW.^3.*XX.*YY.*ZZ+(-1).*phi.^2.*WW.^3.*XX.*YY.*ZZ) ...

.^3+(-9).*((-3).*WW.^3+2.*phi.*WW.^3+(-1).*WW.^4+(-2).*phi.*WW.^4+3.* ...

WW.^2.*XX+(-2).*phi.*WW.^2.*XX+WW.^3.*XX+2.*phi.*WW.^3.*XX+(-1).*WW.^2.* ...

YY+2.*phi.*WW.^2.*YY+WW.^3.*YY+(-6).*phi.*WW.^3.*YY+4.*WW.^4.*YY+4.* ...

phi.*WW.^4.*YY+(-3).*WW.^3.*XX.*YY+2.*phi.*WW.^3.*XX.*YY+(-1).*WW.^4.* ...

XX.*YY+(-2).*phi.*WW.^4.*XX.*YY+WW.^2.*ZZ+(-2).*phi.*WW.^2.*ZZ+3.* ...

WW.^3.*ZZ+2.*phi.*WW.^3.*ZZ+(-4).*WW.^2.*XX.*ZZ+4.*phi.*WW.^2.*XX.*ZZ+( ...

-1).*WW.^3.*XX.*ZZ+(-6).*phi.*WW.^3.*XX.*ZZ+WW.^4.*XX.*ZZ+2.*phi.* ...

WW.^4.*XX.*ZZ+(-1).*WW.^3.*YY.*ZZ+2.*phi.*WW.^3.*YY.*ZZ+(-3).*WW.^4.* ...

YY.*ZZ+(-2).*phi.*WW.^4.*YY.*ZZ+WW.^2.*XX.*YY.*ZZ+(-2).*phi.*WW.^2.*XX.* ...

YY.*ZZ+3.*WW.^3.*XX.*YY.*ZZ+2.*phi.*WW.^3.*XX.*YY.*ZZ).*(3.*WW.^2+(-4).* ...

phi.*WW.^2+phi.^2.*WW.^2+3.*WW.^3+2.*phi.*WW.^3+(-2).*phi.^2.*WW.^3+2.* ...

phi.*WW.^4+phi.^2.*WW.^4+(-3).*WW.*XX+4.*phi.*WW.*XX+(-1).*phi.^2.*WW.* ...

XX+(-3).*WW.^2.*XX+(-2).*phi.*WW.^2.*XX+2.*phi.^2.*WW.^2.*XX+(-2).*phi.* ...

WW.^3.*XX+(-1).*phi.^2.*WW.^3.*XX+(-1).*phi.*WW.*YY+phi.^2.*WW.*YY+ ...

WW.^2.*YY+2.*phi.*WW.^2.*YY+(-4).*phi.^2.*WW.^2.*YY+(-5).*WW.^3.*YY+5.* ...

phi.*WW.^3.*YY+5.*phi.^2.*WW.^3.*YY+(-2).*WW.^4.*YY+(-6).*phi.*WW.^4.* ...

YY+(-2).*phi.^2.*WW.^4.*YY+3.*WW.^2.*XX.*YY+(-5).*phi.*WW.^2.*XX.*YY+ ...

phi.^2.*WW.^2.*XX.*YY+3.*WW.^3.*XX.*YY+4.*phi.*WW.^3.*XX.*YY+(-2).* ...

phi.^2.*WW.^3.*XX.*YY+phi.*WW.^4.*XX.*YY+phi.^2.*WW.^4.*XX.*YY+phi.*WW.* ...

ZZ+(-1).*phi.^2.*WW.*ZZ+(-3).*WW.^2.*ZZ+4.*phi.*WW.^2.*ZZ+2.*phi.^2.* ...

WW.^2.*ZZ+(-3).*WW.^3.*ZZ+(-5).*phi.*WW.^3.*ZZ+(-1).*phi.^2.*WW.^3.*ZZ+ ...

2.*WW.*XX.*ZZ+(-6).*phi.*WW.*XX.*ZZ+2.*phi.^2.*WW.*XX.*ZZ+5.*WW.^2.*XX.* ...

ZZ+5.*phi.*WW.^2.*XX.*ZZ+(-5).*phi.^2.*WW.^2.*XX.*ZZ+(-1).*WW.^3.*XX.* ...

ZZ+2.*phi.*WW.^3.*XX.*ZZ+4.*phi.^2.*WW.^3.*XX.*ZZ+(-1).*phi.*WW.^4.*XX.* ...

ZZ+(-1).*phi.^2.*WW.^4.*XX.*ZZ+(-2).*phi.*WW.^2.*YY.*ZZ+phi.^2.*WW.^2.* ...

YY.*ZZ+3.*WW.^3.*YY.*ZZ+(-2).*phi.*WW.^3.*YY.*ZZ+(-2).*phi.^2.*WW.^3.* ...

YY.*ZZ+3.*WW.^4.*YY.*ZZ+4.*phi.*WW.^4.*YY.*ZZ+phi.^2.*WW.^4.*YY.*ZZ+2.* ...

phi.*WW.*XX.*YY.*ZZ+(-1).*phi.^2.*WW.*XX.*YY.*ZZ+(-3).*WW.^2.*XX.*YY.* ...

ZZ+2.*phi.*WW.^2.*XX.*YY.*ZZ+2.*phi.^2.*WW.^2.*XX.*YY.*ZZ+(-3).*WW.^3.* ...

XX.*YY.*ZZ+(-4).*phi.*WW.^3.*XX.*YY.*ZZ+(-1).*phi.^2.*WW.^3.*XX.*YY.*ZZ) ...

.*((-1).*WW+2.*phi.*WW+(-1).*phi.^2.*WW+(-3).*WW.^2+2.*phi.*WW.^2+ ...

phi.^2.*WW.^2+(-4).*phi.*WW.^3+phi.^2.*WW.^3+(-1).*phi.^2.*WW.^4+XX+(-2) ...

.*phi.*XX+phi.^2.*XX+3.*WW.*XX+(-2).*phi.*WW.*XX+(-1).*phi.^2.*WW.*XX+ ...

4.*phi.*WW.^2.*XX+(-1).*phi.^2.*WW.^2.*XX+phi.^2.*WW.^3.*XX+WW.^2.*YY+( ...

-4).*phi.*WW.^2.*YY+2.*phi.^2.*WW.^2.*YY+3.*WW.^3.*YY+2.*phi.*WW.^3.*YY+ ...

(-4).*phi.^2.*WW.^3.*YY+2.*phi.*WW.^4.*YY+2.*phi.^2.*WW.^4.*YY+(-1).* ...

WW.*XX.*YY+4.*phi.*WW.*XX.*YY+(-2).*phi.^2.*WW.*XX.*YY+(-3).*WW.^2.*XX.* ...

YY+(-2).*phi.*WW.^2.*XX.*YY+4.*phi.^2.*WW.^2.*XX.*YY+(-2).*phi.*WW.^3.* ...

XX.*YY+(-2).*phi.^2.*WW.^3.*XX.*YY+(-2).*phi.*WW.*ZZ+2.*phi.^2.*WW.*ZZ+ ...

3.*WW.^2.*ZZ+(-2).*phi.*WW.^2.*ZZ+(-4).*phi.^2.*WW.^2.*ZZ+WW.^3.*ZZ+4.* ...

phi.*WW.^3.*ZZ+2.*phi.^2.*WW.^3.*ZZ+2.*phi.*XX.*ZZ+(-2).*phi.^2.*XX.*ZZ+ ...

(-3).*WW.*XX.*ZZ+2.*phi.*WW.*XX.*ZZ+4.*phi.^2.*WW.*XX.*ZZ+(-1).*WW.^2.* ...

XX.*ZZ+(-4).*phi.*WW.^2.*XX.*ZZ+(-2).*phi.^2.*WW.^2.*XX.*ZZ+(-1).* ...

phi.^2.*WW.*YY.*ZZ+4.*phi.*WW.^2.*YY.*ZZ+phi.^2.*WW.^2.*YY.*ZZ+(-3).* ...

WW.^3.*YY.*ZZ+(-2).*phi.*WW.^3.*YY.*ZZ+phi.^2.*WW.^3.*YY.*ZZ+(-1).* ...

WW.^4.*YY.*ZZ+(-2).*phi.*WW.^4.*YY.*ZZ+(-1).*phi.^2.*WW.^4.*YY.*ZZ+ ...

phi.^2.*XX.*YY.*ZZ+(-4).*phi.*WW.*XX.*YY.*ZZ+(-1).*phi.^2.*WW.*XX.*YY.* ...

ZZ+3.*WW.^2.*XX.*YY.*ZZ+2.*phi.*WW.^2.*XX.*YY.*ZZ+(-1).*phi.^2.*WW.^2.* ...

XX.*YY.*ZZ+WW.^3.*XX.*YY.*ZZ+2.*phi.*WW.^3.*XX.*YY.*ZZ+phi.^2.*WW.^3.* ...

XX.*YY.*ZZ)+27.*(WW.^4+(-1).*WW.^3.*XX+WW.^3.*YY+(-2).*WW.^4.*YY+WW.^4.* ...

XX.*YY+(-1).*WW.^3.*ZZ+2.*WW.^3.*XX.*ZZ+(-1).*WW.^4.*XX.*ZZ+WW.^4.*YY.* ...

ZZ+(-1).*WW.^3.*XX.*YY.*ZZ).*((-1).*WW+2.*phi.*WW+(-1).*phi.^2.*WW+(-3) ...

.*WW.^2+2.*phi.*WW.^2+phi.^2.*WW.^2+(-4).*phi.*WW.^3+phi.^2.*WW.^3+(-1) ...

.*phi.^2.*WW.^4+XX+(-2).*phi.*XX+phi.^2.*XX+3.*WW.*XX+(-2).*phi.*WW.*XX+ ...

(-1).*phi.^2.*WW.*XX+4.*phi.*WW.^2.*XX+(-1).*phi.^2.*WW.^2.*XX+phi.^2.* ...

WW.^3.*XX+WW.^2.*YY+(-4).*phi.*WW.^2.*YY+2.*phi.^2.*WW.^2.*YY+3.*WW.^3.* ...

YY+2.*phi.*WW.^3.*YY+(-4).*phi.^2.*WW.^3.*YY+2.*phi.*WW.^4.*YY+2.* ...

phi.^2.*WW.^4.*YY+(-1).*WW.*XX.*YY+4.*phi.*WW.*XX.*YY+(-2).*phi.^2.*WW.* ...

XX.*YY+(-3).*WW.^2.*XX.*YY+(-2).*phi.*WW.^2.*XX.*YY+4.*phi.^2.*WW.^2.* ...

XX.*YY+(-2).*phi.*WW.^3.*XX.*YY+(-2).*phi.^2.*WW.^3.*XX.*YY+(-2).*phi.* ...

WW.*ZZ+2.*phi.^2.*WW.*ZZ+3.*WW.^2.*ZZ+(-2).*phi.*WW.^2.*ZZ+(-4).* ...

phi.^2.*WW.^2.*ZZ+WW.^3.*ZZ+4.*phi.*WW.^3.*ZZ+2.*phi.^2.*WW.^3.*ZZ+2.* ...

phi.*XX.*ZZ+(-2).*phi.^2.*XX.*ZZ+(-3).*WW.*XX.*ZZ+2.*phi.*WW.*XX.*ZZ+4.* ...

phi.^2.*WW.*XX.*ZZ+(-1).*WW.^2.*XX.*ZZ+(-4).*phi.*WW.^2.*XX.*ZZ+(-2).* ...

phi.^2.*WW.^2.*XX.*ZZ+(-1).*phi.^2.*WW.*YY.*ZZ+4.*phi.*WW.^2.*YY.*ZZ+ ...

phi.^2.*WW.^2.*YY.*ZZ+(-3).*WW.^3.*YY.*ZZ+(-2).*phi.*WW.^3.*YY.*ZZ+ ...

phi.^2.*WW.^3.*YY.*ZZ+(-1).*WW.^4.*YY.*ZZ+(-2).*phi.*WW.^4.*YY.*ZZ+(-1) ...

.*phi.^2.*WW.^4.*YY.*ZZ+phi.^2.*XX.*YY.*ZZ+(-4).*phi.*WW.*XX.*YY.*ZZ+( ...

-1).*phi.^2.*WW.*XX.*YY.*ZZ+3.*WW.^2.*XX.*YY.*ZZ+2.*phi.*WW.^2.*XX.*YY.* ...

ZZ+(-1).*phi.^2.*WW.^2.*XX.*YY.*ZZ+WW.^3.*XX.*YY.*ZZ+2.*phi.*WW.^3.*XX.* ...

YY.*ZZ+phi.^2.*WW.^3.*XX.*YY.*ZZ).^2).^2).^(1/2)).^(1/3)).^(1/2)+(1/2).* ...

((1/2).*((-1)+phi+(-1).*phi.*WW+(-1).*phi.*YY+WW.*YY+phi.*WW.*YY).^(-2) ...

.*((-1)+phi+(-1).*phi.*WW+(-1).*phi.*ZZ+WW.*ZZ+phi.*WW.*ZZ).^(-2).*((-1) ...

+2.*phi+(-1).*phi.^2+(-3).*WW+2.*phi.*WW+phi.^2.*WW+(-4).*phi.*WW.^2+ ...

phi.^2.*WW.^2+(-1).*phi.^2.*WW.^3+WW.*YY+(-4).*phi.*WW.*YY+2.*phi.^2.* ...

WW.*YY+3.*WW.^2.*YY+2.*phi.*WW.^2.*YY+(-4).*phi.^2.*WW.^2.*YY+2.*phi.* ...

WW.^3.*YY+2.*phi.^2.*WW.^3.*YY+(-2).*phi.*ZZ+2.*phi.^2.*ZZ+3.*WW.*ZZ+( ...

-2).*phi.*WW.*ZZ+(-4).*phi.^2.*WW.*ZZ+WW.^2.*ZZ+4.*phi.*WW.^2.*ZZ+2.* ...

phi.^2.*WW.^2.*ZZ+(-1).*phi.^2.*YY.*ZZ+4.*phi.*WW.*YY.*ZZ+phi.^2.*WW.* ...

YY.*ZZ+(-3).*WW.^2.*YY.*ZZ+(-2).*phi.*WW.^2.*YY.*ZZ+phi.^2.*WW.^2.*YY.* ...

ZZ+(-1).*WW.^3.*YY.*ZZ+(-2).*phi.*WW.^3.*YY.*ZZ+(-1).*phi.^2.*WW.^3.* ...

YY.*ZZ).^2+(-1).*(WW+(-1).*XX).^(-1).*((-1)+phi+(-1).*phi.*WW+(-1).* ...

phi.*YY+WW.*YY+phi.*WW.*YY).^(-1).*((-1)+phi+(-1).*phi.*WW+(-1).*phi.* ...

ZZ+WW.*ZZ+phi.*WW.*ZZ).^(-1).*(3.*WW.^2+(-4).*phi.*WW.^2+phi.^2.*WW.^2+ ...

3.*WW.^3+2.*phi.*WW.^3+(-2).*phi.^2.*WW.^3+2.*phi.*WW.^4+phi.^2.*WW.^4+( ...

-3).*WW.*XX+4.*phi.*WW.*XX+(-1).*phi.^2.*WW.*XX+(-3).*WW.^2.*XX+(-2).* ...

phi.*WW.^2.*XX+2.*phi.^2.*WW.^2.*XX+(-2).*phi.*WW.^3.*XX+(-1).*phi.^2.* ...

WW.^3.*XX+(-1).*phi.*WW.*YY+phi.^2.*WW.*YY+WW.^2.*YY+2.*phi.*WW.^2.*YY+( ...

-4).*phi.^2.*WW.^2.*YY+(-5).*WW.^3.*YY+5.*phi.*WW.^3.*YY+5.*phi.^2.* ...

WW.^3.*YY+(-2).*WW.^4.*YY+(-6).*phi.*WW.^4.*YY+(-2).*phi.^2.*WW.^4.*YY+ ...

3.*WW.^2.*XX.*YY+(-5).*phi.*WW.^2.*XX.*YY+phi.^2.*WW.^2.*XX.*YY+3.* ...

WW.^3.*XX.*YY+4.*phi.*WW.^3.*XX.*YY+(-2).*phi.^2.*WW.^3.*XX.*YY+phi.* ...

WW.^4.*XX.*YY+phi.^2.*WW.^4.*XX.*YY+phi.*WW.*ZZ+(-1).*phi.^2.*WW.*ZZ+( ...

-3).*WW.^2.*ZZ+4.*phi.*WW.^2.*ZZ+2.*phi.^2.*WW.^2.*ZZ+(-3).*WW.^3.*ZZ+( ...

-5).*phi.*WW.^3.*ZZ+(-1).*phi.^2.*WW.^3.*ZZ+2.*WW.*XX.*ZZ+(-6).*phi.* ...

WW.*XX.*ZZ+2.*phi.^2.*WW.*XX.*ZZ+5.*WW.^2.*XX.*ZZ+5.*phi.*WW.^2.*XX.*ZZ+ ...

(-5).*phi.^2.*WW.^2.*XX.*ZZ+(-1).*WW.^3.*XX.*ZZ+2.*phi.*WW.^3.*XX.*ZZ+ ...

4.*phi.^2.*WW.^3.*XX.*ZZ+(-1).*phi.*WW.^4.*XX.*ZZ+(-1).*phi.^2.*WW.^4.* ...

XX.*ZZ+(-2).*phi.*WW.^2.*YY.*ZZ+phi.^2.*WW.^2.*YY.*ZZ+3.*WW.^3.*YY.*ZZ+( ...

-2).*phi.*WW.^3.*YY.*ZZ+(-2).*phi.^2.*WW.^3.*YY.*ZZ+3.*WW.^4.*YY.*ZZ+4.* ...

phi.*WW.^4.*YY.*ZZ+phi.^2.*WW.^4.*YY.*ZZ+2.*phi.*WW.*XX.*YY.*ZZ+(-1).* ...

phi.^2.*WW.*XX.*YY.*ZZ+(-3).*WW.^2.*XX.*YY.*ZZ+2.*phi.*WW.^2.*XX.*YY.* ...

ZZ+2.*phi.^2.*WW.^2.*XX.*YY.*ZZ+(-3).*WW.^3.*XX.*YY.*ZZ+(-4).*phi.* ...

WW.^3.*XX.*YY.*ZZ+(-1).*phi.^2.*WW.^3.*XX.*YY.*ZZ)+(-1/3).*(WW+(-2).* ...

phi.*WW+phi.^2.*WW+2.*phi.*WW.^2+(-2).*phi.^2.*WW.^2+phi.^2.*WW.^3+(-1) ...

.*XX+2.*phi.*XX+(-1).*phi.^2.*XX+(-2).*phi.*WW.*XX+2.*phi.^2.*WW.*XX+( ...

-1).*phi.^2.*WW.^2.*XX+phi.*WW.*YY+(-1).*phi.^2.*WW.*YY+(-1).*WW.^2.*YY+ ...

2.*phi.^2.*WW.^2.*YY+(-1).*phi.*WW.^3.*YY+(-1).*phi.^2.*WW.^3.*YY+(-1).* ...

phi.*XX.*YY+phi.^2.*XX.*YY+WW.*XX.*YY+(-2).*phi.^2.*WW.*XX.*YY+phi.* ...

WW.^2.*XX.*YY+phi.^2.*WW.^2.*XX.*YY+phi.*WW.*ZZ+(-1).*phi.^2.*WW.*ZZ+( ...

-1).*WW.^2.*ZZ+2.*phi.^2.*WW.^2.*ZZ+(-1).*phi.*WW.^3.*ZZ+(-1).*phi.^2.* ...

WW.^3.*ZZ+(-1).*phi.*XX.*ZZ+phi.^2.*XX.*ZZ+WW.*XX.*ZZ+(-2).*phi.^2.*WW.* ...

XX.*ZZ+phi.*WW.^2.*XX.*ZZ+phi.^2.*WW.^2.*XX.*ZZ+phi.^2.*WW.*YY.*ZZ+(-2) ...

.*phi.*WW.^2.*YY.*ZZ+(-2).*phi.^2.*WW.^2.*YY.*ZZ+WW.^3.*YY.*ZZ+2.*phi.* ...

WW.^3.*YY.*ZZ+phi.^2.*WW.^3.*YY.*ZZ+(-1).*phi.^2.*XX.*YY.*ZZ+2.*phi.* ...

WW.*XX.*YY.*ZZ+2.*phi.^2.*WW.*XX.*YY.*ZZ+(-1).*WW.^2.*XX.*YY.*ZZ+(-2).* ...

phi.*WW.^2.*XX.*YY.*ZZ+(-1).*phi.^2.*WW.^2.*XX.*YY.*ZZ).^(-1).*(3.* ...

WW.^2+(-4).*phi.*WW.^2+phi.^2.*WW.^2+3.*WW.^3+2.*phi.*WW.^3+(-2).* ...

phi.^2.*WW.^3+2.*phi.*WW.^4+phi.^2.*WW.^4+(-3).*WW.*XX+4.*phi.*WW.*XX+( ...

-1).*phi.^2.*WW.*XX+(-3).*WW.^2.*XX+(-2).*phi.*WW.^2.*XX+2.*phi.^2.* ...

WW.^2.*XX+(-2).*phi.*WW.^3.*XX+(-1).*phi.^2.*WW.^3.*XX+(-1).*phi.*WW.* ...

YY+phi.^2.*WW.*YY+WW.^2.*YY+2.*phi.*WW.^2.*YY+(-4).*phi.^2.*WW.^2.*YY+( ...

-5).*WW.^3.*YY+5.*phi.*WW.^3.*YY+5.*phi.^2.*WW.^3.*YY+(-2).*WW.^4.*YY+( ...

-6).*phi.*WW.^4.*YY+(-2).*phi.^2.*WW.^4.*YY+3.*WW.^2.*XX.*YY+(-5).*phi.* ...

WW.^2.*XX.*YY+phi.^2.*WW.^2.*XX.*YY+3.*WW.^3.*XX.*YY+4.*phi.*WW.^3.*XX.* ...

YY+(-2).*phi.^2.*WW.^3.*XX.*YY+phi.*WW.^4.*XX.*YY+phi.^2.*WW.^4.*XX.*YY+ ...

phi.*WW.*ZZ+(-1).*phi.^2.*WW.*ZZ+(-3).*WW.^2.*ZZ+4.*phi.*WW.^2.*ZZ+2.* ...

phi.^2.*WW.^2.*ZZ+(-3).*WW.^3.*ZZ+(-5).*phi.*WW.^3.*ZZ+(-1).*phi.^2.* ...

WW.^3.*ZZ+2.*WW.*XX.*ZZ+(-6).*phi.*WW.*XX.*ZZ+2.*phi.^2.*WW.*XX.*ZZ+5.* ...

WW.^2.*XX.*ZZ+5.*phi.*WW.^2.*XX.*ZZ+(-5).*phi.^2.*WW.^2.*XX.*ZZ+(-1).* ...

WW.^3.*XX.*ZZ+2.*phi.*WW.^3.*XX.*ZZ+4.*phi.^2.*WW.^3.*XX.*ZZ+(-1).*phi.* ...

WW.^4.*XX.*ZZ+(-1).*phi.^2.*WW.^4.*XX.*ZZ+(-2).*phi.*WW.^2.*YY.*ZZ+ ...

phi.^2.*WW.^2.*YY.*ZZ+3.*WW.^3.*YY.*ZZ+(-2).*phi.*WW.^3.*YY.*ZZ+(-2).* ...

phi.^2.*WW.^3.*YY.*ZZ+3.*WW.^4.*YY.*ZZ+4.*phi.*WW.^4.*YY.*ZZ+phi.^2.* ...

WW.^4.*YY.*ZZ+2.*phi.*WW.*XX.*YY.*ZZ+(-1).*phi.^2.*WW.*XX.*YY.*ZZ+(-3).* ...

WW.^2.*XX.*YY.*ZZ+2.*phi.*WW.^2.*XX.*YY.*ZZ+2.*phi.^2.*WW.^2.*XX.*YY.* ...

ZZ+(-3).*WW.^3.*XX.*YY.*ZZ+(-4).*phi.*WW.^3.*XX.*YY.*ZZ+(-1).*phi.^2.* ...

WW.^3.*XX.*YY.*ZZ)+(-1/3).*2.^(1/3).*(WW+(-1).*XX).^(-1).*((-1)+phi+(-1) ...

.*phi.*WW+(-1).*phi.*YY+WW.*YY+phi.*WW.*YY).^(-1).*((-1)+phi+(-1).*phi.* ...

WW+(-1).*phi.*ZZ+WW.*ZZ+phi.*WW.*ZZ).^(-1).*(12.*(WW+(-2).*phi.*WW+ ...

phi.^2.*WW+2.*phi.*WW.^2+(-2).*phi.^2.*WW.^2+phi.^2.*WW.^3+(-1).*XX+2.* ...

phi.*XX+(-1).*phi.^2.*XX+(-2).*phi.*WW.*XX+2.*phi.^2.*WW.*XX+(-1).* ...

phi.^2.*WW.^2.*XX+phi.*WW.*YY+(-1).*phi.^2.*WW.*YY+(-1).*WW.^2.*YY+2.* ...

phi.^2.*WW.^2.*YY+(-1).*phi.*WW.^3.*YY+(-1).*phi.^2.*WW.^3.*YY+(-1).* ...

phi.*XX.*YY+phi.^2.*XX.*YY+WW.*XX.*YY+(-2).*phi.^2.*WW.*XX.*YY+phi.* ...

WW.^2.*XX.*YY+phi.^2.*WW.^2.*XX.*YY+phi.*WW.*ZZ+(-1).*phi.^2.*WW.*ZZ+( ...

-1).*WW.^2.*ZZ+2.*phi.^2.*WW.^2.*ZZ+(-1).*phi.*WW.^3.*ZZ+(-1).*phi.^2.* ...

WW.^3.*ZZ+(-1).*phi.*XX.*ZZ+phi.^2.*XX.*ZZ+WW.*XX.*ZZ+(-2).*phi.^2.*WW.* ...

XX.*ZZ+phi.*WW.^2.*XX.*ZZ+phi.^2.*WW.^2.*XX.*ZZ+phi.^2.*WW.*YY.*ZZ+(-2) ...

.*phi.*WW.^2.*YY.*ZZ+(-2).*phi.^2.*WW.^2.*YY.*ZZ+WW.^3.*YY.*ZZ+2.*phi.* ...

WW.^3.*YY.*ZZ+phi.^2.*WW.^3.*YY.*ZZ+(-1).*phi.^2.*XX.*YY.*ZZ+2.*phi.* ...

WW.*XX.*YY.*ZZ+2.*phi.^2.*WW.*XX.*YY.*ZZ+(-1).*WW.^2.*XX.*YY.*ZZ+(-2).* ...

phi.*WW.^2.*XX.*YY.*ZZ+(-1).*phi.^2.*WW.^2.*XX.*YY.*ZZ).*(WW.^4+(-1).* ...

WW.^3.*XX+WW.^3.*YY+(-2).*WW.^4.*YY+WW.^4.*XX.*YY+(-1).*WW.^3.*ZZ+2.* ...

WW.^3.*XX.*ZZ+(-1).*WW.^4.*XX.*ZZ+WW.^4.*YY.*ZZ+(-1).*WW.^3.*XX.*YY.*ZZ) ...

+(3.*WW.^2+(-4).*phi.*WW.^2+phi.^2.*WW.^2+3.*WW.^3+2.*phi.*WW.^3+(-2).* ...

phi.^2.*WW.^3+2.*phi.*WW.^4+phi.^2.*WW.^4+(-3).*WW.*XX+4.*phi.*WW.*XX+( ...

-1).*phi.^2.*WW.*XX+(-3).*WW.^2.*XX+(-2).*phi.*WW.^2.*XX+2.*phi.^2.* ...

WW.^2.*XX+(-2).*phi.*WW.^3.*XX+(-1).*phi.^2.*WW.^3.*XX+(-1).*phi.*WW.* ...

YY+phi.^2.*WW.*YY+WW.^2.*YY+2.*phi.*WW.^2.*YY+(-4).*phi.^2.*WW.^2.*YY+( ...

-5).*WW.^3.*YY+5.*phi.*WW.^3.*YY+5.*phi.^2.*WW.^3.*YY+(-2).*WW.^4.*YY+( ...

-6).*phi.*WW.^4.*YY+(-2).*phi.^2.*WW.^4.*YY+3.*WW.^2.*XX.*YY+(-5).*phi.* ...

WW.^2.*XX.*YY+phi.^2.*WW.^2.*XX.*YY+3.*WW.^3.*XX.*YY+4.*phi.*WW.^3.*XX.* ...

YY+(-2).*phi.^2.*WW.^3.*XX.*YY+phi.*WW.^4.*XX.*YY+phi.^2.*WW.^4.*XX.*YY+ ...

phi.*WW.*ZZ+(-1).*phi.^2.*WW.*ZZ+(-3).*WW.^2.*ZZ+4.*phi.*WW.^2.*ZZ+2.* ...

phi.^2.*WW.^2.*ZZ+(-3).*WW.^3.*ZZ+(-5).*phi.*WW.^3.*ZZ+(-1).*phi.^2.* ...

WW.^3.*ZZ+2.*WW.*XX.*ZZ+(-6).*phi.*WW.*XX.*ZZ+2.*phi.^2.*WW.*XX.*ZZ+5.* ...

WW.^2.*XX.*ZZ+5.*phi.*WW.^2.*XX.*ZZ+(-5).*phi.^2.*WW.^2.*XX.*ZZ+(-1).* ...

WW.^3.*XX.*ZZ+2.*phi.*WW.^3.*XX.*ZZ+4.*phi.^2.*WW.^3.*XX.*ZZ+(-1).*phi.* ...

WW.^4.*XX.*ZZ+(-1).*phi.^2.*WW.^4.*XX.*ZZ+(-2).*phi.*WW.^2.*YY.*ZZ+ ...

phi.^2.*WW.^2.*YY.*ZZ+3.*WW.^3.*YY.*ZZ+(-2).*phi.*WW.^3.*YY.*ZZ+(-2).* ...

phi.^2.*WW.^3.*YY.*ZZ+3.*WW.^4.*YY.*ZZ+4.*phi.*WW.^4.*YY.*ZZ+phi.^2.* ...

WW.^4.*YY.*ZZ+2.*phi.*WW.*XX.*YY.*ZZ+(-1).*phi.^2.*WW.*XX.*YY.*ZZ+(-3).* ...

WW.^2.*XX.*YY.*ZZ+2.*phi.*WW.^2.*XX.*YY.*ZZ+2.*phi.^2.*WW.^2.*XX.*YY.* ...

ZZ+(-3).*WW.^3.*XX.*YY.*ZZ+(-4).*phi.*WW.^3.*XX.*YY.*ZZ+(-1).*phi.^2.* ...

WW.^3.*XX.*YY.*ZZ).^2+(-3).*((-3).*WW.^3+2.*phi.*WW.^3+(-1).*WW.^4+(-2) ...

.*phi.*WW.^4+3.*WW.^2.*XX+(-2).*phi.*WW.^2.*XX+WW.^3.*XX+2.*phi.*WW.^3.* ...

XX+(-1).*WW.^2.*YY+2.*phi.*WW.^2.*YY+WW.^3.*YY+(-6).*phi.*WW.^3.*YY+4.* ...

WW.^4.*YY+4.*phi.*WW.^4.*YY+(-3).*WW.^3.*XX.*YY+2.*phi.*WW.^3.*XX.*YY+( ...

-1).*WW.^4.*XX.*YY+(-2).*phi.*WW.^4.*XX.*YY+WW.^2.*ZZ+(-2).*phi.*WW.^2.* ...

ZZ+3.*WW.^3.*ZZ+2.*phi.*WW.^3.*ZZ+(-4).*WW.^2.*XX.*ZZ+4.*phi.*WW.^2.* ...

XX.*ZZ+(-1).*WW.^3.*XX.*ZZ+(-6).*phi.*WW.^3.*XX.*ZZ+WW.^4.*XX.*ZZ+2.* ...

phi.*WW.^4.*XX.*ZZ+(-1).*WW.^3.*YY.*ZZ+2.*phi.*WW.^3.*YY.*ZZ+(-3).* ...

WW.^4.*YY.*ZZ+(-2).*phi.*WW.^4.*YY.*ZZ+WW.^2.*XX.*YY.*ZZ+(-2).*phi.* ...

WW.^2.*XX.*YY.*ZZ+3.*WW.^3.*XX.*YY.*ZZ+2.*phi.*WW.^3.*XX.*YY.*ZZ).*((-1) ...

.*WW+2.*phi.*WW+(-1).*phi.^2.*WW+(-3).*WW.^2+2.*phi.*WW.^2+phi.^2.* ...

WW.^2+(-4).*phi.*WW.^3+phi.^2.*WW.^3+(-1).*phi.^2.*WW.^4+XX+(-2).*phi.* ...

XX+phi.^2.*XX+3.*WW.*XX+(-2).*phi.*WW.*XX+(-1).*phi.^2.*WW.*XX+4.*phi.* ...

WW.^2.*XX+(-1).*phi.^2.*WW.^2.*XX+phi.^2.*WW.^3.*XX+WW.^2.*YY+(-4).* ...

phi.*WW.^2.*YY+2.*phi.^2.*WW.^2.*YY+3.*WW.^3.*YY+2.*phi.*WW.^3.*YY+(-4) ...

.*phi.^2.*WW.^3.*YY+2.*phi.*WW.^4.*YY+2.*phi.^2.*WW.^4.*YY+(-1).*WW.* ...

XX.*YY+4.*phi.*WW.*XX.*YY+(-2).*phi.^2.*WW.*XX.*YY+(-3).*WW.^2.*XX.*YY+( ...

-2).*phi.*WW.^2.*XX.*YY+4.*phi.^2.*WW.^2.*XX.*YY+(-2).*phi.*WW.^3.*XX.* ...

YY+(-2).*phi.^2.*WW.^3.*XX.*YY+(-2).*phi.*WW.*ZZ+2.*phi.^2.*WW.*ZZ+3.* ...

WW.^2.*ZZ+(-2).*phi.*WW.^2.*ZZ+(-4).*phi.^2.*WW.^2.*ZZ+WW.^3.*ZZ+4.* ...

phi.*WW.^3.*ZZ+2.*phi.^2.*WW.^3.*ZZ+2.*phi.*XX.*ZZ+(-2).*phi.^2.*XX.*ZZ+ ...

(-3).*WW.*XX.*ZZ+2.*phi.*WW.*XX.*ZZ+4.*phi.^2.*WW.*XX.*ZZ+(-1).*WW.^2.* ...

XX.*ZZ+(-4).*phi.*WW.^2.*XX.*ZZ+(-2).*phi.^2.*WW.^2.*XX.*ZZ+(-1).* ...

phi.^2.*WW.*YY.*ZZ+4.*phi.*WW.^2.*YY.*ZZ+phi.^2.*WW.^2.*YY.*ZZ+(-3).* ...

WW.^3.*YY.*ZZ+(-2).*phi.*WW.^3.*YY.*ZZ+phi.^2.*WW.^3.*YY.*ZZ+(-1).* ...

WW.^4.*YY.*ZZ+(-2).*phi.*WW.^4.*YY.*ZZ+(-1).*phi.^2.*WW.^4.*YY.*ZZ+ ...

phi.^2.*XX.*YY.*ZZ+(-4).*phi.*WW.*XX.*YY.*ZZ+(-1).*phi.^2.*WW.*XX.*YY.* ...

ZZ+3.*WW.^2.*XX.*YY.*ZZ+2.*phi.*WW.^2.*XX.*YY.*ZZ+(-1).*phi.^2.*WW.^2.* ...

XX.*YY.*ZZ+WW.^3.*XX.*YY.*ZZ+2.*phi.*WW.^3.*XX.*YY.*ZZ+phi.^2.*WW.^3.* ...

XX.*YY.*ZZ)).*(27.*(WW+(-2).*phi.*WW+phi.^2.*WW+2.*phi.*WW.^2+(-2).* ...

phi.^2.*WW.^2+phi.^2.*WW.^3+(-1).*XX+2.*phi.*XX+(-1).*phi.^2.*XX+(-2).* ...

phi.*WW.*XX+2.*phi.^2.*WW.*XX+(-1).*phi.^2.*WW.^2.*XX+phi.*WW.*YY+(-1).* ...

phi.^2.*WW.*YY+(-1).*WW.^2.*YY+2.*phi.^2.*WW.^2.*YY+(-1).*phi.*WW.^3.* ...

YY+(-1).*phi.^2.*WW.^3.*YY+(-1).*phi.*XX.*YY+phi.^2.*XX.*YY+WW.*XX.*YY+( ...

-2).*phi.^2.*WW.*XX.*YY+phi.*WW.^2.*XX.*YY+phi.^2.*WW.^2.*XX.*YY+phi.* ...

WW.*ZZ+(-1).*phi.^2.*WW.*ZZ+(-1).*WW.^2.*ZZ+2.*phi.^2.*WW.^2.*ZZ+(-1).* ...

phi.*WW.^3.*ZZ+(-1).*phi.^2.*WW.^3.*ZZ+(-1).*phi.*XX.*ZZ+phi.^2.*XX.*ZZ+ ...

WW.*XX.*ZZ+(-2).*phi.^2.*WW.*XX.*ZZ+phi.*WW.^2.*XX.*ZZ+phi.^2.*WW.^2.* ...

XX.*ZZ+phi.^2.*WW.*YY.*ZZ+(-2).*phi.*WW.^2.*YY.*ZZ+(-2).*phi.^2.*WW.^2.* ...

YY.*ZZ+WW.^3.*YY.*ZZ+2.*phi.*WW.^3.*YY.*ZZ+phi.^2.*WW.^3.*YY.*ZZ+(-1).* ...

phi.^2.*XX.*YY.*ZZ+2.*phi.*WW.*XX.*YY.*ZZ+2.*phi.^2.*WW.*XX.*YY.*ZZ+(-1) ...

.*WW.^2.*XX.*YY.*ZZ+(-2).*phi.*WW.^2.*XX.*YY.*ZZ+(-1).*phi.^2.*WW.^2.* ...

XX.*YY.*ZZ).*((-3).*WW.^3+2.*phi.*WW.^3+(-1).*WW.^4+(-2).*phi.*WW.^4+3.* ...

WW.^2.*XX+(-2).*phi.*WW.^2.*XX+WW.^3.*XX+2.*phi.*WW.^3.*XX+(-1).*WW.^2.* ...

YY+2.*phi.*WW.^2.*YY+WW.^3.*YY+(-6).*phi.*WW.^3.*YY+4.*WW.^4.*YY+4.* ...

phi.*WW.^4.*YY+(-3).*WW.^3.*XX.*YY+2.*phi.*WW.^3.*XX.*YY+(-1).*WW.^4.* ...

XX.*YY+(-2).*phi.*WW.^4.*XX.*YY+WW.^2.*ZZ+(-2).*phi.*WW.^2.*ZZ+3.* ...

WW.^3.*ZZ+2.*phi.*WW.^3.*ZZ+(-4).*WW.^2.*XX.*ZZ+4.*phi.*WW.^2.*XX.*ZZ+( ...

-1).*WW.^3.*XX.*ZZ+(-6).*phi.*WW.^3.*XX.*ZZ+WW.^4.*XX.*ZZ+2.*phi.* ...

WW.^4.*XX.*ZZ+(-1).*WW.^3.*YY.*ZZ+2.*phi.*WW.^3.*YY.*ZZ+(-3).*WW.^4.* ...

YY.*ZZ+(-2).*phi.*WW.^4.*YY.*ZZ+WW.^2.*XX.*YY.*ZZ+(-2).*phi.*WW.^2.*XX.* ...

YY.*ZZ+3.*WW.^3.*XX.*YY.*ZZ+2.*phi.*WW.^3.*XX.*YY.*ZZ).^2+(-72).*(WW+( ...

-2).*phi.*WW+phi.^2.*WW+2.*phi.*WW.^2+(-2).*phi.^2.*WW.^2+phi.^2.*WW.^3+ ...

(-1).*XX+2.*phi.*XX+(-1).*phi.^2.*XX+(-2).*phi.*WW.*XX+2.*phi.^2.*WW.* ...

XX+(-1).*phi.^2.*WW.^2.*XX+phi.*WW.*YY+(-1).*phi.^2.*WW.*YY+(-1).* ...

WW.^2.*YY+2.*phi.^2.*WW.^2.*YY+(-1).*phi.*WW.^3.*YY+(-1).*phi.^2.* ...

WW.^3.*YY+(-1).*phi.*XX.*YY+phi.^2.*XX.*YY+WW.*XX.*YY+(-2).*phi.^2.*WW.* ...

XX.*YY+phi.*WW.^2.*XX.*YY+phi.^2.*WW.^2.*XX.*YY+phi.*WW.*ZZ+(-1).* ...

phi.^2.*WW.*ZZ+(-1).*WW.^2.*ZZ+2.*phi.^2.*WW.^2.*ZZ+(-1).*phi.*WW.^3.* ...

ZZ+(-1).*phi.^2.*WW.^3.*ZZ+(-1).*phi.*XX.*ZZ+phi.^2.*XX.*ZZ+WW.*XX.*ZZ+( ...

-2).*phi.^2.*WW.*XX.*ZZ+phi.*WW.^2.*XX.*ZZ+phi.^2.*WW.^2.*XX.*ZZ+ ...

phi.^2.*WW.*YY.*ZZ+(-2).*phi.*WW.^2.*YY.*ZZ+(-2).*phi.^2.*WW.^2.*YY.*ZZ+ ...

WW.^3.*YY.*ZZ+2.*phi.*WW.^3.*YY.*ZZ+phi.^2.*WW.^3.*YY.*ZZ+(-1).*phi.^2.* ...

XX.*YY.*ZZ+2.*phi.*WW.*XX.*YY.*ZZ+2.*phi.^2.*WW.*XX.*YY.*ZZ+(-1).* ...

WW.^2.*XX.*YY.*ZZ+(-2).*phi.*WW.^2.*XX.*YY.*ZZ+(-1).*phi.^2.*WW.^2.*XX.* ...

YY.*ZZ).*(WW.^4+(-1).*WW.^3.*XX+WW.^3.*YY+(-2).*WW.^4.*YY+WW.^4.*XX.*YY+ ...

(-1).*WW.^3.*ZZ+2.*WW.^3.*XX.*ZZ+(-1).*WW.^4.*XX.*ZZ+WW.^4.*YY.*ZZ+(-1) ...

.*WW.^3.*XX.*YY.*ZZ).*(3.*WW.^2+(-4).*phi.*WW.^2+phi.^2.*WW.^2+3.*WW.^3+ ...

2.*phi.*WW.^3+(-2).*phi.^2.*WW.^3+2.*phi.*WW.^4+phi.^2.*WW.^4+(-3).*WW.* ...

XX+4.*phi.*WW.*XX+(-1).*phi.^2.*WW.*XX+(-3).*WW.^2.*XX+(-2).*phi.* ...

WW.^2.*XX+2.*phi.^2.*WW.^2.*XX+(-2).*phi.*WW.^3.*XX+(-1).*phi.^2.* ...

WW.^3.*XX+(-1).*phi.*WW.*YY+phi.^2.*WW.*YY+WW.^2.*YY+2.*phi.*WW.^2.*YY+( ...

-4).*phi.^2.*WW.^2.*YY+(-5).*WW.^3.*YY+5.*phi.*WW.^3.*YY+5.*phi.^2.* ...

WW.^3.*YY+(-2).*WW.^4.*YY+(-6).*phi.*WW.^4.*YY+(-2).*phi.^2.*WW.^4.*YY+ ...

3.*WW.^2.*XX.*YY+(-5).*phi.*WW.^2.*XX.*YY+phi.^2.*WW.^2.*XX.*YY+3.* ...

WW.^3.*XX.*YY+4.*phi.*WW.^3.*XX.*YY+(-2).*phi.^2.*WW.^3.*XX.*YY+phi.* ...

WW.^4.*XX.*YY+phi.^2.*WW.^4.*XX.*YY+phi.*WW.*ZZ+(-1).*phi.^2.*WW.*ZZ+( ...

-3).*WW.^2.*ZZ+4.*phi.*WW.^2.*ZZ+2.*phi.^2.*WW.^2.*ZZ+(-3).*WW.^3.*ZZ+( ...

-5).*phi.*WW.^3.*ZZ+(-1).*phi.^2.*WW.^3.*ZZ+2.*WW.*XX.*ZZ+(-6).*phi.* ...

WW.*XX.*ZZ+2.*phi.^2.*WW.*XX.*ZZ+5.*WW.^2.*XX.*ZZ+5.*phi.*WW.^2.*XX.*ZZ+ ...

(-5).*phi.^2.*WW.^2.*XX.*ZZ+(-1).*WW.^3.*XX.*ZZ+2.*phi.*WW.^3.*XX.*ZZ+ ...

4.*phi.^2.*WW.^3.*XX.*ZZ+(-1).*phi.*WW.^4.*XX.*ZZ+(-1).*phi.^2.*WW.^4.* ...

XX.*ZZ+(-2).*phi.*WW.^2.*YY.*ZZ+phi.^2.*WW.^2.*YY.*ZZ+3.*WW.^3.*YY.*ZZ+( ...

-2).*phi.*WW.^3.*YY.*ZZ+(-2).*phi.^2.*WW.^3.*YY.*ZZ+3.*WW.^4.*YY.*ZZ+4.* ...

phi.*WW.^4.*YY.*ZZ+phi.^2.*WW.^4.*YY.*ZZ+2.*phi.*WW.*XX.*YY.*ZZ+(-1).* ...

phi.^2.*WW.*XX.*YY.*ZZ+(-3).*WW.^2.*XX.*YY.*ZZ+2.*phi.*WW.^2.*XX.*YY.* ...

ZZ+2.*phi.^2.*WW.^2.*XX.*YY.*ZZ+(-3).*WW.^3.*XX.*YY.*ZZ+(-4).*phi.* ...

WW.^3.*XX.*YY.*ZZ+(-1).*phi.^2.*WW.^3.*XX.*YY.*ZZ)+2.*(3.*WW.^2+(-4).* ...

phi.*WW.^2+phi.^2.*WW.^2+3.*WW.^3+2.*phi.*WW.^3+(-2).*phi.^2.*WW.^3+2.* ...

phi.*WW.^4+phi.^2.*WW.^4+(-3).*WW.*XX+4.*phi.*WW.*XX+(-1).*phi.^2.*WW.* ...

XX+(-3).*WW.^2.*XX+(-2).*phi.*WW.^2.*XX+2.*phi.^2.*WW.^2.*XX+(-2).*phi.* ...

WW.^3.*XX+(-1).*phi.^2.*WW.^3.*XX+(-1).*phi.*WW.*YY+phi.^2.*WW.*YY+ ...

WW.^2.*YY+2.*phi.*WW.^2.*YY+(-4).*phi.^2.*WW.^2.*YY+(-5).*WW.^3.*YY+5.* ...

phi.*WW.^3.*YY+5.*phi.^2.*WW.^3.*YY+(-2).*WW.^4.*YY+(-6).*phi.*WW.^4.* ...

YY+(-2).*phi.^2.*WW.^4.*YY+3.*WW.^2.*XX.*YY+(-5).*phi.*WW.^2.*XX.*YY+ ...

phi.^2.*WW.^2.*XX.*YY+3.*WW.^3.*XX.*YY+4.*phi.*WW.^3.*XX.*YY+(-2).* ...

phi.^2.*WW.^3.*XX.*YY+phi.*WW.^4.*XX.*YY+phi.^2.*WW.^4.*XX.*YY+phi.*WW.* ...

ZZ+(-1).*phi.^2.*WW.*ZZ+(-3).*WW.^2.*ZZ+4.*phi.*WW.^2.*ZZ+2.*phi.^2.* ...

WW.^2.*ZZ+(-3).*WW.^3.*ZZ+(-5).*phi.*WW.^3.*ZZ+(-1).*phi.^2.*WW.^3.*ZZ+ ...

2.*WW.*XX.*ZZ+(-6).*phi.*WW.*XX.*ZZ+2.*phi.^2.*WW.*XX.*ZZ+5.*WW.^2.*XX.* ...

ZZ+5.*phi.*WW.^2.*XX.*ZZ+(-5).*phi.^2.*WW.^2.*XX.*ZZ+(-1).*WW.^3.*XX.* ...

ZZ+2.*phi.*WW.^3.*XX.*ZZ+4.*phi.^2.*WW.^3.*XX.*ZZ+(-1).*phi.*WW.^4.*XX.* ...

ZZ+(-1).*phi.^2.*WW.^4.*XX.*ZZ+(-2).*phi.*WW.^2.*YY.*ZZ+phi.^2.*WW.^2.* ...

YY.*ZZ+3.*WW.^3.*YY.*ZZ+(-2).*phi.*WW.^3.*YY.*ZZ+(-2).*phi.^2.*WW.^3.* ...

YY.*ZZ+3.*WW.^4.*YY.*ZZ+4.*phi.*WW.^4.*YY.*ZZ+phi.^2.*WW.^4.*YY.*ZZ+2.* ...

phi.*WW.*XX.*YY.*ZZ+(-1).*phi.^2.*WW.*XX.*YY.*ZZ+(-3).*WW.^2.*XX.*YY.* ...

ZZ+2.*phi.*WW.^2.*XX.*YY.*ZZ+2.*phi.^2.*WW.^2.*XX.*YY.*ZZ+(-3).*WW.^3.* ...

XX.*YY.*ZZ+(-4).*phi.*WW.^3.*XX.*YY.*ZZ+(-1).*phi.^2.*WW.^3.*XX.*YY.*ZZ) ...

.^3+(-9).*((-3).*WW.^3+2.*phi.*WW.^3+(-1).*WW.^4+(-2).*phi.*WW.^4+3.* ...

WW.^2.*XX+(-2).*phi.*WW.^2.*XX+WW.^3.*XX+2.*phi.*WW.^3.*XX+(-1).*WW.^2.* ...

YY+2.*phi.*WW.^2.*YY+WW.^3.*YY+(-6).*phi.*WW.^3.*YY+4.*WW.^4.*YY+4.* ...

phi.*WW.^4.*YY+(-3).*WW.^3.*XX.*YY+2.*phi.*WW.^3.*XX.*YY+(-1).*WW.^4.* ...

XX.*YY+(-2).*phi.*WW.^4.*XX.*YY+WW.^2.*ZZ+(-2).*phi.*WW.^2.*ZZ+3.* ...

WW.^3.*ZZ+2.*phi.*WW.^3.*ZZ+(-4).*WW.^2.*XX.*ZZ+4.*phi.*WW.^2.*XX.*ZZ+( ...

-1).*WW.^3.*XX.*ZZ+(-6).*phi.*WW.^3.*XX.*ZZ+WW.^4.*XX.*ZZ+2.*phi.* ...

WW.^4.*XX.*ZZ+(-1).*WW.^3.*YY.*ZZ+2.*phi.*WW.^3.*YY.*ZZ+(-3).*WW.^4.* ...

YY.*ZZ+(-2).*phi.*WW.^4.*YY.*ZZ+WW.^2.*XX.*YY.*ZZ+(-2).*phi.*WW.^2.*XX.* ...

YY.*ZZ+3.*WW.^3.*XX.*YY.*ZZ+2.*phi.*WW.^3.*XX.*YY.*ZZ).*(3.*WW.^2+(-4).* ...

phi.*WW.^2+phi.^2.*WW.^2+3.*WW.^3+2.*phi.*WW.^3+(-2).*phi.^2.*WW.^3+2.* ...

phi.*WW.^4+phi.^2.*WW.^4+(-3).*WW.*XX+4.*phi.*WW.*XX+(-1).*phi.^2.*WW.* ...

XX+(-3).*WW.^2.*XX+(-2).*phi.*WW.^2.*XX+2.*phi.^2.*WW.^2.*XX+(-2).*phi.* ...

WW.^3.*XX+(-1).*phi.^2.*WW.^3.*XX+(-1).*phi.*WW.*YY+phi.^2.*WW.*YY+ ...

WW.^2.*YY+2.*phi.*WW.^2.*YY+(-4).*phi.^2.*WW.^2.*YY+(-5).*WW.^3.*YY+5.* ...

phi.*WW.^3.*YY+5.*phi.^2.*WW.^3.*YY+(-2).*WW.^4.*YY+(-6).*phi.*WW.^4.* ...

YY+(-2).*phi.^2.*WW.^4.*YY+3.*WW.^2.*XX.*YY+(-5).*phi.*WW.^2.*XX.*YY+ ...

phi.^2.*WW.^2.*XX.*YY+3.*WW.^3.*XX.*YY+4.*phi.*WW.^3.*XX.*YY+(-2).* ...

phi.^2.*WW.^3.*XX.*YY+phi.*WW.^4.*XX.*YY+phi.^2.*WW.^4.*XX.*YY+phi.*WW.* ...

ZZ+(-1).*phi.^2.*WW.*ZZ+(-3).*WW.^2.*ZZ+4.*phi.*WW.^2.*ZZ+2.*phi.^2.* ...

WW.^2.*ZZ+(-3).*WW.^3.*ZZ+(-5).*phi.*WW.^3.*ZZ+(-1).*phi.^2.*WW.^3.*ZZ+ ...

2.*WW.*XX.*ZZ+(-6).*phi.*WW.*XX.*ZZ+2.*phi.^2.*WW.*XX.*ZZ+5.*WW.^2.*XX.* ...

ZZ+5.*phi.*WW.^2.*XX.*ZZ+(-5).*phi.^2.*WW.^2.*XX.*ZZ+(-1).*WW.^3.*XX.* ...

ZZ+2.*phi.*WW.^3.*XX.*ZZ+4.*phi.^2.*WW.^3.*XX.*ZZ+(-1).*phi.*WW.^4.*XX.* ...

ZZ+(-1).*phi.^2.*WW.^4.*XX.*ZZ+(-2).*phi.*WW.^2.*YY.*ZZ+phi.^2.*WW.^2.* ...

YY.*ZZ+3.*WW.^3.*YY.*ZZ+(-2).*phi.*WW.^3.*YY.*ZZ+(-2).*phi.^2.*WW.^3.* ...

YY.*ZZ+3.*WW.^4.*YY.*ZZ+4.*phi.*WW.^4.*YY.*ZZ+phi.^2.*WW.^4.*YY.*ZZ+2.* ...

phi.*WW.*XX.*YY.*ZZ+(-1).*phi.^2.*WW.*XX.*YY.*ZZ+(-3).*WW.^2.*XX.*YY.* ...

ZZ+2.*phi.*WW.^2.*XX.*YY.*ZZ+2.*phi.^2.*WW.^2.*XX.*YY.*ZZ+(-3).*WW.^3.* ...

XX.*YY.*ZZ+(-4).*phi.*WW.^3.*XX.*YY.*ZZ+(-1).*phi.^2.*WW.^3.*XX.*YY.*ZZ) ...

.*((-1).*WW+2.*phi.*WW+(-1).*phi.^2.*WW+(-3).*WW.^2+2.*phi.*WW.^2+ ...

phi.^2.*WW.^2+(-4).*phi.*WW.^3+phi.^2.*WW.^3+(-1).*phi.^2.*WW.^4+XX+(-2) ...

.*phi.*XX+phi.^2.*XX+3.*WW.*XX+(-2).*phi.*WW.*XX+(-1).*phi.^2.*WW.*XX+ ...

4.*phi.*WW.^2.*XX+(-1).*phi.^2.*WW.^2.*XX+phi.^2.*WW.^3.*XX+WW.^2.*YY+( ...

-4).*phi.*WW.^2.*YY+2.*phi.^2.*WW.^2.*YY+3.*WW.^3.*YY+2.*phi.*WW.^3.*YY+ ...

(-4).*phi.^2.*WW.^3.*YY+2.*phi.*WW.^4.*YY+2.*phi.^2.*WW.^4.*YY+(-1).* ...

WW.*XX.*YY+4.*phi.*WW.*XX.*YY+(-2).*phi.^2.*WW.*XX.*YY+(-3).*WW.^2.*XX.* ...

YY+(-2).*phi.*WW.^2.*XX.*YY+4.*phi.^2.*WW.^2.*XX.*YY+(-2).*phi.*WW.^3.* ...

XX.*YY+(-2).*phi.^2.*WW.^3.*XX.*YY+(-2).*phi.*WW.*ZZ+2.*phi.^2.*WW.*ZZ+ ...

3.*WW.^2.*ZZ+(-2).*phi.*WW.^2.*ZZ+(-4).*phi.^2.*WW.^2.*ZZ+WW.^3.*ZZ+4.* ...

phi.*WW.^3.*ZZ+2.*phi.^2.*WW.^3.*ZZ+2.*phi.*XX.*ZZ+(-2).*phi.^2.*XX.*ZZ+ ...

(-3).*WW.*XX.*ZZ+2.*phi.*WW.*XX.*ZZ+4.*phi.^2.*WW.*XX.*ZZ+(-1).*WW.^2.* ...

XX.*ZZ+(-4).*phi.*WW.^2.*XX.*ZZ+(-2).*phi.^2.*WW.^2.*XX.*ZZ+(-1).* ...

phi.^2.*WW.*YY.*ZZ+4.*phi.*WW.^2.*YY.*ZZ+phi.^2.*WW.^2.*YY.*ZZ+(-3).* ...

WW.^3.*YY.*ZZ+(-2).*phi.*WW.^3.*YY.*ZZ+phi.^2.*WW.^3.*YY.*ZZ+(-1).* ...

WW.^4.*YY.*ZZ+(-2).*phi.*WW.^4.*YY.*ZZ+(-1).*phi.^2.*WW.^4.*YY.*ZZ+ ...

phi.^2.*XX.*YY.*ZZ+(-4).*phi.*WW.*XX.*YY.*ZZ+(-1).*phi.^2.*WW.*XX.*YY.* ...

ZZ+3.*WW.^2.*XX.*YY.*ZZ+2.*phi.*WW.^2.*XX.*YY.*ZZ+(-1).*phi.^2.*WW.^2.* ...

XX.*YY.*ZZ+WW.^3.*XX.*YY.*ZZ+2.*phi.*WW.^3.*XX.*YY.*ZZ+phi.^2.*WW.^3.* ...

XX.*YY.*ZZ)+27.*(WW.^4+(-1).*WW.^3.*XX+WW.^3.*YY+(-2).*WW.^4.*YY+WW.^4.* ...

XX.*YY+(-1).*WW.^3.*ZZ+2.*WW.^3.*XX.*ZZ+(-1).*WW.^4.*XX.*ZZ+WW.^4.*YY.* ...

ZZ+(-1).*WW.^3.*XX.*YY.*ZZ).*((-1).*WW+2.*phi.*WW+(-1).*phi.^2.*WW+(-3) ...

.*WW.^2+2.*phi.*WW.^2+phi.^2.*WW.^2+(-4).*phi.*WW.^3+phi.^2.*WW.^3+(-1) ...

.*phi.^2.*WW.^4+XX+(-2).*phi.*XX+phi.^2.*XX+3.*WW.*XX+(-2).*phi.*WW.*XX+ ...

(-1).*phi.^2.*WW.*XX+4.*phi.*WW.^2.*XX+(-1).*phi.^2.*WW.^2.*XX+phi.^2.* ...

WW.^3.*XX+WW.^2.*YY+(-4).*phi.*WW.^2.*YY+2.*phi.^2.*WW.^2.*YY+3.*WW.^3.* ...

YY+2.*phi.*WW.^3.*YY+(-4).*phi.^2.*WW.^3.*YY+2.*phi.*WW.^4.*YY+2.* ...

phi.^2.*WW.^4.*YY+(-1).*WW.*XX.*YY+4.*phi.*WW.*XX.*YY+(-2).*phi.^2.*WW.* ...

XX.*YY+(-3).*WW.^2.*XX.*YY+(-2).*phi.*WW.^2.*XX.*YY+4.*phi.^2.*WW.^2.* ...

XX.*YY+(-2).*phi.*WW.^3.*XX.*YY+(-2).*phi.^2.*WW.^3.*XX.*YY+(-2).*phi.* ...

WW.*ZZ+2.*phi.^2.*WW.*ZZ+3.*WW.^2.*ZZ+(-2).*phi.*WW.^2.*ZZ+(-4).* ...

phi.^2.*WW.^2.*ZZ+WW.^3.*ZZ+4.*phi.*WW.^3.*ZZ+2.*phi.^2.*WW.^3.*ZZ+2.* ...

phi.*XX.*ZZ+(-2).*phi.^2.*XX.*ZZ+(-3).*WW.*XX.*ZZ+2.*phi.*WW.*XX.*ZZ+4.* ...

phi.^2.*WW.*XX.*ZZ+(-1).*WW.^2.*XX.*ZZ+(-4).*phi.*WW.^2.*XX.*ZZ+(-2).* ...

phi.^2.*WW.^2.*XX.*ZZ+(-1).*phi.^2.*WW.*YY.*ZZ+4.*phi.*WW.^2.*YY.*ZZ+ ...

phi.^2.*WW.^2.*YY.*ZZ+(-3).*WW.^3.*YY.*ZZ+(-2).*phi.*WW.^3.*YY.*ZZ+ ...

phi.^2.*WW.^3.*YY.*ZZ+(-1).*WW.^4.*YY.*ZZ+(-2).*phi.*WW.^4.*YY.*ZZ+(-1) ...

.*phi.^2.*WW.^4.*YY.*ZZ+phi.^2.*XX.*YY.*ZZ+(-4).*phi.*WW.*XX.*YY.*ZZ+( ...

-1).*phi.^2.*WW.*XX.*YY.*ZZ+3.*WW.^2.*XX.*YY.*ZZ+2.*phi.*WW.^2.*XX.*YY.* ...

ZZ+(-1).*phi.^2.*WW.^2.*XX.*YY.*ZZ+WW.^3.*XX.*YY.*ZZ+2.*phi.*WW.^3.*XX.* ...

YY.*ZZ+phi.^2.*WW.^3.*XX.*YY.*ZZ).^2+((-4).*(12.*(WW+(-2).*phi.*WW+ ...

phi.^2.*WW+2.*phi.*WW.^2+(-2).*phi.^2.*WW.^2+phi.^2.*WW.^3+(-1).*XX+2.* ...

phi.*XX+(-1).*phi.^2.*XX+(-2).*phi.*WW.*XX+2.*phi.^2.*WW.*XX+(-1).* ...

phi.^2.*WW.^2.*XX+phi.*WW.*YY+(-1).*phi.^2.*WW.*YY+(-1).*WW.^2.*YY+2.* ...

phi.^2.*WW.^2.*YY+(-1).*phi.*WW.^3.*YY+(-1).*phi.^2.*WW.^3.*YY+(-1).* ...

phi.*XX.*YY+phi.^2.*XX.*YY+WW.*XX.*YY+(-2).*phi.^2.*WW.*XX.*YY+phi.* ...

WW.^2.*XX.*YY+phi.^2.*WW.^2.*XX.*YY+phi.*WW.*ZZ+(-1).*phi.^2.*WW.*ZZ+( ...

-1).*WW.^2.*ZZ+2.*phi.^2.*WW.^2.*ZZ+(-1).*phi.*WW.^3.*ZZ+(-1).*phi.^2.* ...

WW.^3.*ZZ+(-1).*phi.*XX.*ZZ+phi.^2.*XX.*ZZ+WW.*XX.*ZZ+(-2).*phi.^2.*WW.* ...

XX.*ZZ+phi.*WW.^2.*XX.*ZZ+phi.^2.*WW.^2.*XX.*ZZ+phi.^2.*WW.*YY.*ZZ+(-2) ...

.*phi.*WW.^2.*YY.*ZZ+(-2).*phi.^2.*WW.^2.*YY.*ZZ+WW.^3.*YY.*ZZ+2.*phi.* ...

WW.^3.*YY.*ZZ+phi.^2.*WW.^3.*YY.*ZZ+(-1).*phi.^2.*XX.*YY.*ZZ+2.*phi.* ...

WW.*XX.*YY.*ZZ+2.*phi.^2.*WW.*XX.*YY.*ZZ+(-1).*WW.^2.*XX.*YY.*ZZ+(-2).* ...

phi.*WW.^2.*XX.*YY.*ZZ+(-1).*phi.^2.*WW.^2.*XX.*YY.*ZZ).*(WW.^4+(-1).* ...

WW.^3.*XX+WW.^3.*YY+(-2).*WW.^4.*YY+WW.^4.*XX.*YY+(-1).*WW.^3.*ZZ+2.* ...

WW.^3.*XX.*ZZ+(-1).*WW.^4.*XX.*ZZ+WW.^4.*YY.*ZZ+(-1).*WW.^3.*XX.*YY.*ZZ) ...

+(3.*WW.^2+(-4).*phi.*WW.^2+phi.^2.*WW.^2+3.*WW.^3+2.*phi.*WW.^3+(-2).* ...

phi.^2.*WW.^3+2.*phi.*WW.^4+phi.^2.*WW.^4+(-3).*WW.*XX+4.*phi.*WW.*XX+( ...

-1).*phi.^2.*WW.*XX+(-3).*WW.^2.*XX+(-2).*phi.*WW.^2.*XX+2.*phi.^2.* ...

WW.^2.*XX+(-2).*phi.*WW.^3.*XX+(-1).*phi.^2.*WW.^3.*XX+(-1).*phi.*WW.* ...

YY+phi.^2.*WW.*YY+WW.^2.*YY+2.*phi.*WW.^2.*YY+(-4).*phi.^2.*WW.^2.*YY+( ...

-5).*WW.^3.*YY+5.*phi.*WW.^3.*YY+5.*phi.^2.*WW.^3.*YY+(-2).*WW.^4.*YY+( ...

-6).*phi.*WW.^4.*YY+(-2).*phi.^2.*WW.^4.*YY+3.*WW.^2.*XX.*YY+(-5).*phi.* ...

WW.^2.*XX.*YY+phi.^2.*WW.^2.*XX.*YY+3.*WW.^3.*XX.*YY+4.*phi.*WW.^3.*XX.* ...

YY+(-2).*phi.^2.*WW.^3.*XX.*YY+phi.*WW.^4.*XX.*YY+phi.^2.*WW.^4.*XX.*YY+ ...

phi.*WW.*ZZ+(-1).*phi.^2.*WW.*ZZ+(-3).*WW.^2.*ZZ+4.*phi.*WW.^2.*ZZ+2.* ...

phi.^2.*WW.^2.*ZZ+(-3).*WW.^3.*ZZ+(-5).*phi.*WW.^3.*ZZ+(-1).*phi.^2.* ...

WW.^3.*ZZ+2.*WW.*XX.*ZZ+(-6).*phi.*WW.*XX.*ZZ+2.*phi.^2.*WW.*XX.*ZZ+5.* ...

WW.^2.*XX.*ZZ+5.*phi.*WW.^2.*XX.*ZZ+(-5).*phi.^2.*WW.^2.*XX.*ZZ+(-1).* ...

WW.^3.*XX.*ZZ+2.*phi.*WW.^3.*XX.*ZZ+4.*phi.^2.*WW.^3.*XX.*ZZ+(-1).*phi.* ...

WW.^4.*XX.*ZZ+(-1).*phi.^2.*WW.^4.*XX.*ZZ+(-2).*phi.*WW.^2.*YY.*ZZ+ ...

phi.^2.*WW.^2.*YY.*ZZ+3.*WW.^3.*YY.*ZZ+(-2).*phi.*WW.^3.*YY.*ZZ+(-2).* ...

phi.^2.*WW.^3.*YY.*ZZ+3.*WW.^4.*YY.*ZZ+4.*phi.*WW.^4.*YY.*ZZ+phi.^2.* ...

WW.^4.*YY.*ZZ+2.*phi.*WW.*XX.*YY.*ZZ+(-1).*phi.^2.*WW.*XX.*YY.*ZZ+(-3).* ...

WW.^2.*XX.*YY.*ZZ+2.*phi.*WW.^2.*XX.*YY.*ZZ+2.*phi.^2.*WW.^2.*XX.*YY.* ...

ZZ+(-3).*WW.^3.*XX.*YY.*ZZ+(-4).*phi.*WW.^3.*XX.*YY.*ZZ+(-1).*phi.^2.* ...

WW.^3.*XX.*YY.*ZZ).^2+(-3).*((-3).*WW.^3+2.*phi.*WW.^3+(-1).*WW.^4+(-2) ...

.*phi.*WW.^4+3.*WW.^2.*XX+(-2).*phi.*WW.^2.*XX+WW.^3.*XX+2.*phi.*WW.^3.* ...

XX+(-1).*WW.^2.*YY+2.*phi.*WW.^2.*YY+WW.^3.*YY+(-6).*phi.*WW.^3.*YY+4.* ...

WW.^4.*YY+4.*phi.*WW.^4.*YY+(-3).*WW.^3.*XX.*YY+2.*phi.*WW.^3.*XX.*YY+( ...

-1).*WW.^4.*XX.*YY+(-2).*phi.*WW.^4.*XX.*YY+WW.^2.*ZZ+(-2).*phi.*WW.^2.* ...

ZZ+3.*WW.^3.*ZZ+2.*phi.*WW.^3.*ZZ+(-4).*WW.^2.*XX.*ZZ+4.*phi.*WW.^2.* ...

XX.*ZZ+(-1).*WW.^3.*XX.*ZZ+(-6).*phi.*WW.^3.*XX.*ZZ+WW.^4.*XX.*ZZ+2.* ...

phi.*WW.^4.*XX.*ZZ+(-1).*WW.^3.*YY.*ZZ+2.*phi.*WW.^3.*YY.*ZZ+(-3).* ...

WW.^4.*YY.*ZZ+(-2).*phi.*WW.^4.*YY.*ZZ+WW.^2.*XX.*YY.*ZZ+(-2).*phi.* ...

WW.^2.*XX.*YY.*ZZ+3.*WW.^3.*XX.*YY.*ZZ+2.*phi.*WW.^3.*XX.*YY.*ZZ).*((-1) ...

.*WW+2.*phi.*WW+(-1).*phi.^2.*WW+(-3).*WW.^2+2.*phi.*WW.^2+phi.^2.* ...

WW.^2+(-4).*phi.*WW.^3+phi.^2.*WW.^3+(-1).*phi.^2.*WW.^4+XX+(-2).*phi.* ...

XX+phi.^2.*XX+3.*WW.*XX+(-2).*phi.*WW.*XX+(-1).*phi.^2.*WW.*XX+4.*phi.* ...

WW.^2.*XX+(-1).*phi.^2.*WW.^2.*XX+phi.^2.*WW.^3.*XX+WW.^2.*YY+(-4).* ...

phi.*WW.^2.*YY+2.*phi.^2.*WW.^2.*YY+3.*WW.^3.*YY+2.*phi.*WW.^3.*YY+(-4) ...

.*phi.^2.*WW.^3.*YY+2.*phi.*WW.^4.*YY+2.*phi.^2.*WW.^4.*YY+(-1).*WW.* ...

XX.*YY+4.*phi.*WW.*XX.*YY+(-2).*phi.^2.*WW.*XX.*YY+(-3).*WW.^2.*XX.*YY+( ...

-2).*phi.*WW.^2.*XX.*YY+4.*phi.^2.*WW.^2.*XX.*YY+(-2).*phi.*WW.^3.*XX.* ...

YY+(-2).*phi.^2.*WW.^3.*XX.*YY+(-2).*phi.*WW.*ZZ+2.*phi.^2.*WW.*ZZ+3.* ...

WW.^2.*ZZ+(-2).*phi.*WW.^2.*ZZ+(-4).*phi.^2.*WW.^2.*ZZ+WW.^3.*ZZ+4.* ...

phi.*WW.^3.*ZZ+2.*phi.^2.*WW.^3.*ZZ+2.*phi.*XX.*ZZ+(-2).*phi.^2.*XX.*ZZ+ ...

(-3).*WW.*XX.*ZZ+2.*phi.*WW.*XX.*ZZ+4.*phi.^2.*WW.*XX.*ZZ+(-1).*WW.^2.* ...

XX.*ZZ+(-4).*phi.*WW.^2.*XX.*ZZ+(-2).*phi.^2.*WW.^2.*XX.*ZZ+(-1).* ...

phi.^2.*WW.*YY.*ZZ+4.*phi.*WW.^2.*YY.*ZZ+phi.^2.*WW.^2.*YY.*ZZ+(-3).* ...

WW.^3.*YY.*ZZ+(-2).*phi.*WW.^3.*YY.*ZZ+phi.^2.*WW.^3.*YY.*ZZ+(-1).* ...

WW.^4.*YY.*ZZ+(-2).*phi.*WW.^4.*YY.*ZZ+(-1).*phi.^2.*WW.^4.*YY.*ZZ+ ...

phi.^2.*XX.*YY.*ZZ+(-4).*phi.*WW.*XX.*YY.*ZZ+(-1).*phi.^2.*WW.*XX.*YY.* ...

ZZ+3.*WW.^2.*XX.*YY.*ZZ+2.*phi.*WW.^2.*XX.*YY.*ZZ+(-1).*phi.^2.*WW.^2.* ...

XX.*YY.*ZZ+WW.^3.*XX.*YY.*ZZ+2.*phi.*WW.^3.*XX.*YY.*ZZ+phi.^2.*WW.^3.* ...

XX.*YY.*ZZ)).^3+(27.*(WW+(-2).*phi.*WW+phi.^2.*WW+2.*phi.*WW.^2+(-2).* ...

phi.^2.*WW.^2+phi.^2.*WW.^3+(-1).*XX+2.*phi.*XX+(-1).*phi.^2.*XX+(-2).* ...

phi.*WW.*XX+2.*phi.^2.*WW.*XX+(-1).*phi.^2.*WW.^2.*XX+phi.*WW.*YY+(-1).* ...

phi.^2.*WW.*YY+(-1).*WW.^2.*YY+2.*phi.^2.*WW.^2.*YY+(-1).*phi.*WW.^3.* ...

YY+(-1).*phi.^2.*WW.^3.*YY+(-1).*phi.*XX.*YY+phi.^2.*XX.*YY+WW.*XX.*YY+( ...

-2).*phi.^2.*WW.*XX.*YY+phi.*WW.^2.*XX.*YY+phi.^2.*WW.^2.*XX.*YY+phi.* ...

WW.*ZZ+(-1).*phi.^2.*WW.*ZZ+(-1).*WW.^2.*ZZ+2.*phi.^2.*WW.^2.*ZZ+(-1).* ...

phi.*WW.^3.*ZZ+(-1).*phi.^2.*WW.^3.*ZZ+(-1).*phi.*XX.*ZZ+phi.^2.*XX.*ZZ+ ...

WW.*XX.*ZZ+(-2).*phi.^2.*WW.*XX.*ZZ+phi.*WW.^2.*XX.*ZZ+phi.^2.*WW.^2.* ...

XX.*ZZ+phi.^2.*WW.*YY.*ZZ+(-2).*phi.*WW.^2.*YY.*ZZ+(-2).*phi.^2.*WW.^2.* ...

YY.*ZZ+WW.^3.*YY.*ZZ+2.*phi.*WW.^3.*YY.*ZZ+phi.^2.*WW.^3.*YY.*ZZ+(-1).* ...

phi.^2.*XX.*YY.*ZZ+2.*phi.*WW.*XX.*YY.*ZZ+2.*phi.^2.*WW.*XX.*YY.*ZZ+(-1) ...

.*WW.^2.*XX.*YY.*ZZ+(-2).*phi.*WW.^2.*XX.*YY.*ZZ+(-1).*phi.^2.*WW.^2.* ...

XX.*YY.*ZZ).*((-3).*WW.^3+2.*phi.*WW.^3+(-1).*WW.^4+(-2).*phi.*WW.^4+3.* ...

WW.^2.*XX+(-2).*phi.*WW.^2.*XX+WW.^3.*XX+2.*phi.*WW.^3.*XX+(-1).*WW.^2.* ...

YY+2.*phi.*WW.^2.*YY+WW.^3.*YY+(-6).*phi.*WW.^3.*YY+4.*WW.^4.*YY+4.* ...

phi.*WW.^4.*YY+(-3).*WW.^3.*XX.*YY+2.*phi.*WW.^3.*XX.*YY+(-1).*WW.^4.* ...

XX.*YY+(-2).*phi.*WW.^4.*XX.*YY+WW.^2.*ZZ+(-2).*phi.*WW.^2.*ZZ+3.* ...

WW.^3.*ZZ+2.*phi.*WW.^3.*ZZ+(-4).*WW.^2.*XX.*ZZ+4.*phi.*WW.^2.*XX.*ZZ+( ...

-1).*WW.^3.*XX.*ZZ+(-6).*phi.*WW.^3.*XX.*ZZ+WW.^4.*XX.*ZZ+2.*phi.* ...

WW.^4.*XX.*ZZ+(-1).*WW.^3.*YY.*ZZ+2.*phi.*WW.^3.*YY.*ZZ+(-3).*WW.^4.* ...

YY.*ZZ+(-2).*phi.*WW.^4.*YY.*ZZ+WW.^2.*XX.*YY.*ZZ+(-2).*phi.*WW.^2.*XX.* ...

YY.*ZZ+3.*WW.^3.*XX.*YY.*ZZ+2.*phi.*WW.^3.*XX.*YY.*ZZ).^2+(-72).*(WW+( ...

-2).*phi.*WW+phi.^2.*WW+2.*phi.*WW.^2+(-2).*phi.^2.*WW.^2+phi.^2.*WW.^3+ ...

(-1).*XX+2.*phi.*XX+(-1).*phi.^2.*XX+(-2).*phi.*WW.*XX+2.*phi.^2.*WW.* ...

XX+(-1).*phi.^2.*WW.^2.*XX+phi.*WW.*YY+(-1).*phi.^2.*WW.*YY+(-1).* ...

WW.^2.*YY+2.*phi.^2.*WW.^2.*YY+(-1).*phi.*WW.^3.*YY+(-1).*phi.^2.* ...

WW.^3.*YY+(-1).*phi.*XX.*YY+phi.^2.*XX.*YY+WW.*XX.*YY+(-2).*phi.^2.*WW.* ...

XX.*YY+phi.*WW.^2.*XX.*YY+phi.^2.*WW.^2.*XX.*YY+phi.*WW.*ZZ+(-1).* ...

phi.^2.*WW.*ZZ+(-1).*WW.^2.*ZZ+2.*phi.^2.*WW.^2.*ZZ+(-1).*phi.*WW.^3.* ...

ZZ+(-1).*phi.^2.*WW.^3.*ZZ+(-1).*phi.*XX.*ZZ+phi.^2.*XX.*ZZ+WW.*XX.*ZZ+( ...

-2).*phi.^2.*WW.*XX.*ZZ+phi.*WW.^2.*XX.*ZZ+phi.^2.*WW.^2.*XX.*ZZ+ ...

phi.^2.*WW.*YY.*ZZ+(-2).*phi.*WW.^2.*YY.*ZZ+(-2).*phi.^2.*WW.^2.*YY.*ZZ+ ...

WW.^3.*YY.*ZZ+2.*phi.*WW.^3.*YY.*ZZ+phi.^2.*WW.^3.*YY.*ZZ+(-1).*phi.^2.* ...

XX.*YY.*ZZ+2.*phi.*WW.*XX.*YY.*ZZ+2.*phi.^2.*WW.*XX.*YY.*ZZ+(-1).* ...

WW.^2.*XX.*YY.*ZZ+(-2).*phi.*WW.^2.*XX.*YY.*ZZ+(-1).*phi.^2.*WW.^2.*XX.* ...

YY.*ZZ).*(WW.^4+(-1).*WW.^3.*XX+WW.^3.*YY+(-2).*WW.^4.*YY+WW.^4.*XX.*YY+ ...

(-1).*WW.^3.*ZZ+2.*WW.^3.*XX.*ZZ+(-1).*WW.^4.*XX.*ZZ+WW.^4.*YY.*ZZ+(-1) ...

.*WW.^3.*XX.*YY.*ZZ).*(3.*WW.^2+(-4).*phi.*WW.^2+phi.^2.*WW.^2+3.*WW.^3+ ...

2.*phi.*WW.^3+(-2).*phi.^2.*WW.^3+2.*phi.*WW.^4+phi.^2.*WW.^4+(-3).*WW.* ...

XX+4.*phi.*WW.*XX+(-1).*phi.^2.*WW.*XX+(-3).*WW.^2.*XX+(-2).*phi.* ...

WW.^2.*XX+2.*phi.^2.*WW.^2.*XX+(-2).*phi.*WW.^3.*XX+(-1).*phi.^2.* ...

WW.^3.*XX+(-1).*phi.*WW.*YY+phi.^2.*WW.*YY+WW.^2.*YY+2.*phi.*WW.^2.*YY+( ...

-4).*phi.^2.*WW.^2.*YY+(-5).*WW.^3.*YY+5.*phi.*WW.^3.*YY+5.*phi.^2.* ...

WW.^3.*YY+(-2).*WW.^4.*YY+(-6).*phi.*WW.^4.*YY+(-2).*phi.^2.*WW.^4.*YY+ ...

3.*WW.^2.*XX.*YY+(-5).*phi.*WW.^2.*XX.*YY+phi.^2.*WW.^2.*XX.*YY+3.* ...

WW.^3.*XX.*YY+4.*phi.*WW.^3.*XX.*YY+(-2).*phi.^2.*WW.^3.*XX.*YY+phi.* ...

WW.^4.*XX.*YY+phi.^2.*WW.^4.*XX.*YY+phi.*WW.*ZZ+(-1).*phi.^2.*WW.*ZZ+( ...

-3).*WW.^2.*ZZ+4.*phi.*WW.^2.*ZZ+2.*phi.^2.*WW.^2.*ZZ+(-3).*WW.^3.*ZZ+( ...

-5).*phi.*WW.^3.*ZZ+(-1).*phi.^2.*WW.^3.*ZZ+2.*WW.*XX.*ZZ+(-6).*phi.* ...

WW.*XX.*ZZ+2.*phi.^2.*WW.*XX.*ZZ+5.*WW.^2.*XX.*ZZ+5.*phi.*WW.^2.*XX.*ZZ+ ...

(-5).*phi.^2.*WW.^2.*XX.*ZZ+(-1).*WW.^3.*XX.*ZZ+2.*phi.*WW.^3.*XX.*ZZ+ ...

4.*phi.^2.*WW.^3.*XX.*ZZ+(-1).*phi.*WW.^4.*XX.*ZZ+(-1).*phi.^2.*WW.^4.* ...

XX.*ZZ+(-2).*phi.*WW.^2.*YY.*ZZ+phi.^2.*WW.^2.*YY.*ZZ+3.*WW.^3.*YY.*ZZ+( ...

-2).*phi.*WW.^3.*YY.*ZZ+(-2).*phi.^2.*WW.^3.*YY.*ZZ+3.*WW.^4.*YY.*ZZ+4.* ...

phi.*WW.^4.*YY.*ZZ+phi.^2.*WW.^4.*YY.*ZZ+2.*phi.*WW.*XX.*YY.*ZZ+(-1).* ...

phi.^2.*WW.*XX.*YY.*ZZ+(-3).*WW.^2.*XX.*YY.*ZZ+2.*phi.*WW.^2.*XX.*YY.* ...

ZZ+2.*phi.^2.*WW.^2.*XX.*YY.*ZZ+(-3).*WW.^3.*XX.*YY.*ZZ+(-4).*phi.* ...

WW.^3.*XX.*YY.*ZZ+(-1).*phi.^2.*WW.^3.*XX.*YY.*ZZ)+2.*(3.*WW.^2+(-4).* ...

phi.*WW.^2+phi.^2.*WW.^2+3.*WW.^3+2.*phi.*WW.^3+(-2).*phi.^2.*WW.^3+2.* ...

phi.*WW.^4+phi.^2.*WW.^4+(-3).*WW.*XX+4.*phi.*WW.*XX+(-1).*phi.^2.*WW.* ...

XX+(-3).*WW.^2.*XX+(-2).*phi.*WW.^2.*XX+2.*phi.^2.*WW.^2.*XX+(-2).*phi.* ...

WW.^3.*XX+(-1).*phi.^2.*WW.^3.*XX+(-1).*phi.*WW.*YY+phi.^2.*WW.*YY+ ...

WW.^2.*YY+2.*phi.*WW.^2.*YY+(-4).*phi.^2.*WW.^2.*YY+(-5).*WW.^3.*YY+5.* ...

phi.*WW.^3.*YY+5.*phi.^2.*WW.^3.*YY+(-2).*WW.^4.*YY+(-6).*phi.*WW.^4.* ...

YY+(-2).*phi.^2.*WW.^4.*YY+3.*WW.^2.*XX.*YY+(-5).*phi.*WW.^2.*XX.*YY+ ...

phi.^2.*WW.^2.*XX.*YY+3.*WW.^3.*XX.*YY+4.*phi.*WW.^3.*XX.*YY+(-2).* ...

phi.^2.*WW.^3.*XX.*YY+phi.*WW.^4.*XX.*YY+phi.^2.*WW.^4.*XX.*YY+phi.*WW.* ...

ZZ+(-1).*phi.^2.*WW.*ZZ+(-3).*WW.^2.*ZZ+4.*phi.*WW.^2.*ZZ+2.*phi.^2.* ...

WW.^2.*ZZ+(-3).*WW.^3.*ZZ+(-5).*phi.*WW.^3.*ZZ+(-1).*phi.^2.*WW.^3.*ZZ+ ...

2.*WW.*XX.*ZZ+(-6).*phi.*WW.*XX.*ZZ+2.*phi.^2.*WW.*XX.*ZZ+5.*WW.^2.*XX.* ...

ZZ+5.*phi.*WW.^2.*XX.*ZZ+(-5).*phi.^2.*WW.^2.*XX.*ZZ+(-1).*WW.^3.*XX.* ...

ZZ+2.*phi.*WW.^3.*XX.*ZZ+4.*phi.^2.*WW.^3.*XX.*ZZ+(-1).*phi.*WW.^4.*XX.* ...

ZZ+(-1).*phi.^2.*WW.^4.*XX.*ZZ+(-2).*phi.*WW.^2.*YY.*ZZ+phi.^2.*WW.^2.* ...

YY.*ZZ+3.*WW.^3.*YY.*ZZ+(-2).*phi.*WW.^3.*YY.*ZZ+(-2).*phi.^2.*WW.^3.* ...

YY.*ZZ+3.*WW.^4.*YY.*ZZ+4.*phi.*WW.^4.*YY.*ZZ+phi.^2.*WW.^4.*YY.*ZZ+2.* ...

phi.*WW.*XX.*YY.*ZZ+(-1).*phi.^2.*WW.*XX.*YY.*ZZ+(-3).*WW.^2.*XX.*YY.* ...

ZZ+2.*phi.*WW.^2.*XX.*YY.*ZZ+2.*phi.^2.*WW.^2.*XX.*YY.*ZZ+(-3).*WW.^3.* ...

XX.*YY.*ZZ+(-4).*phi.*WW.^3.*XX.*YY.*ZZ+(-1).*phi.^2.*WW.^3.*XX.*YY.*ZZ) ...

.^3+(-9).*((-3).*WW.^3+2.*phi.*WW.^3+(-1).*WW.^4+(-2).*phi.*WW.^4+3.* ...

WW.^2.*XX+(-2).*phi.*WW.^2.*XX+WW.^3.*XX+2.*phi.*WW.^3.*XX+(-1).*WW.^2.* ...

YY+2.*phi.*WW.^2.*YY+WW.^3.*YY+(-6).*phi.*WW.^3.*YY+4.*WW.^4.*YY+4.* ...

phi.*WW.^4.*YY+(-3).*WW.^3.*XX.*YY+2.*phi.*WW.^3.*XX.*YY+(-1).*WW.^4.* ...

XX.*YY+(-2).*phi.*WW.^4.*XX.*YY+WW.^2.*ZZ+(-2).*phi.*WW.^2.*ZZ+3.* ...

WW.^3.*ZZ+2.*phi.*WW.^3.*ZZ+(-4).*WW.^2.*XX.*ZZ+4.*phi.*WW.^2.*XX.*ZZ+( ...

-1).*WW.^3.*XX.*ZZ+(-6).*phi.*WW.^3.*XX.*ZZ+WW.^4.*XX.*ZZ+2.*phi.* ...

WW.^4.*XX.*ZZ+(-1).*WW.^3.*YY.*ZZ+2.*phi.*WW.^3.*YY.*ZZ+(-3).*WW.^4.* ...

YY.*ZZ+(-2).*phi.*WW.^4.*YY.*ZZ+WW.^2.*XX.*YY.*ZZ+(-2).*phi.*WW.^2.*XX.* ...

YY.*ZZ+3.*WW.^3.*XX.*YY.*ZZ+2.*phi.*WW.^3.*XX.*YY.*ZZ).*(3.*WW.^2+(-4).* ...

phi.*WW.^2+phi.^2.*WW.^2+3.*WW.^3+2.*phi.*WW.^3+(-2).*phi.^2.*WW.^3+2.* ...

phi.*WW.^4+phi.^2.*WW.^4+(-3).*WW.*XX+4.*phi.*WW.*XX+(-1).*phi.^2.*WW.* ...

XX+(-3).*WW.^2.*XX+(-2).*phi.*WW.^2.*XX+2.*phi.^2.*WW.^2.*XX+(-2).*phi.* ...

WW.^3.*XX+(-1).*phi.^2.*WW.^3.*XX+(-1).*phi.*WW.*YY+phi.^2.*WW.*YY+ ...

WW.^2.*YY+2.*phi.*WW.^2.*YY+(-4).*phi.^2.*WW.^2.*YY+(-5).*WW.^3.*YY+5.* ...

phi.*WW.^3.*YY+5.*phi.^2.*WW.^3.*YY+(-2).*WW.^4.*YY+(-6).*phi.*WW.^4.* ...

YY+(-2).*phi.^2.*WW.^4.*YY+3.*WW.^2.*XX.*YY+(-5).*phi.*WW.^2.*XX.*YY+ ...

phi.^2.*WW.^2.*XX.*YY+3.*WW.^3.*XX.*YY+4.*phi.*WW.^3.*XX.*YY+(-2).* ...

phi.^2.*WW.^3.*XX.*YY+phi.*WW.^4.*XX.*YY+phi.^2.*WW.^4.*XX.*YY+phi.*WW.* ...

ZZ+(-1).*phi.^2.*WW.*ZZ+(-3).*WW.^2.*ZZ+4.*phi.*WW.^2.*ZZ+2.*phi.^2.* ...

WW.^2.*ZZ+(-3).*WW.^3.*ZZ+(-5).*phi.*WW.^3.*ZZ+(-1).*phi.^2.*WW.^3.*ZZ+ ...

2.*WW.*XX.*ZZ+(-6).*phi.*WW.*XX.*ZZ+2.*phi.^2.*WW.*XX.*ZZ+5.*WW.^2.*XX.* ...

ZZ+5.*phi.*WW.^2.*XX.*ZZ+(-5).*phi.^2.*WW.^2.*XX.*ZZ+(-1).*WW.^3.*XX.* ...

ZZ+2.*phi.*WW.^3.*XX.*ZZ+4.*phi.^2.*WW.^3.*XX.*ZZ+(-1).*phi.*WW.^4.*XX.* ...

ZZ+(-1).*phi.^2.*WW.^4.*XX.*ZZ+(-2).*phi.*WW.^2.*YY.*ZZ+phi.^2.*WW.^2.* ...

YY.*ZZ+3.*WW.^3.*YY.*ZZ+(-2).*phi.*WW.^3.*YY.*ZZ+(-2).*phi.^2.*WW.^3.* ...

YY.*ZZ+3.*WW.^4.*YY.*ZZ+4.*phi.*WW.^4.*YY.*ZZ+phi.^2.*WW.^4.*YY.*ZZ+2.* ...

phi.*WW.*XX.*YY.*ZZ+(-1).*phi.^2.*WW.*XX.*YY.*ZZ+(-3).*WW.^2.*XX.*YY.* ...

ZZ+2.*phi.*WW.^2.*XX.*YY.*ZZ+2.*phi.^2.*WW.^2.*XX.*YY.*ZZ+(-3).*WW.^3.* ...

XX.*YY.*ZZ+(-4).*phi.*WW.^3.*XX.*YY.*ZZ+(-1).*phi.^2.*WW.^3.*XX.*YY.*ZZ) ...

.*((-1).*WW+2.*phi.*WW+(-1).*phi.^2.*WW+(-3).*WW.^2+2.*phi.*WW.^2+ ...

phi.^2.*WW.^2+(-4).*phi.*WW.^3+phi.^2.*WW.^3+(-1).*phi.^2.*WW.^4+XX+(-2) ...

.*phi.*XX+phi.^2.*XX+3.*WW.*XX+(-2).*phi.*WW.*XX+(-1).*phi.^2.*WW.*XX+ ...

4.*phi.*WW.^2.*XX+(-1).*phi.^2.*WW.^2.*XX+phi.^2.*WW.^3.*XX+WW.^2.*YY+( ...

-4).*phi.*WW.^2.*YY+2.*phi.^2.*WW.^2.*YY+3.*WW.^3.*YY+2.*phi.*WW.^3.*YY+ ...

(-4).*phi.^2.*WW.^3.*YY+2.*phi.*WW.^4.*YY+2.*phi.^2.*WW.^4.*YY+(-1).* ...

WW.*XX.*YY+4.*phi.*WW.*XX.*YY+(-2).*phi.^2.*WW.*XX.*YY+(-3).*WW.^2.*XX.* ...

YY+(-2).*phi.*WW.^2.*XX.*YY+4.*phi.^2.*WW.^2.*XX.*YY+(-2).*phi.*WW.^3.* ...

XX.*YY+(-2).*phi.^2.*WW.^3.*XX.*YY+(-2).*phi.*WW.*ZZ+2.*phi.^2.*WW.*ZZ+ ...

3.*WW.^2.*ZZ+(-2).*phi.*WW.^2.*ZZ+(-4).*phi.^2.*WW.^2.*ZZ+WW.^3.*ZZ+4.* ...

phi.*WW.^3.*ZZ+2.*phi.^2.*WW.^3.*ZZ+2.*phi.*XX.*ZZ+(-2).*phi.^2.*XX.*ZZ+ ...

(-3).*WW.*XX.*ZZ+2.*phi.*WW.*XX.*ZZ+4.*phi.^2.*WW.*XX.*ZZ+(-1).*WW.^2.* ...

XX.*ZZ+(-4).*phi.*WW.^2.*XX.*ZZ+(-2).*phi.^2.*WW.^2.*XX.*ZZ+(-1).* ...

phi.^2.*WW.*YY.*ZZ+4.*phi.*WW.^2.*YY.*ZZ+phi.^2.*WW.^2.*YY.*ZZ+(-3).* ...

WW.^3.*YY.*ZZ+(-2).*phi.*WW.^3.*YY.*ZZ+phi.^2.*WW.^3.*YY.*ZZ+(-1).* ...

WW.^4.*YY.*ZZ+(-2).*phi.*WW.^4.*YY.*ZZ+(-1).*phi.^2.*WW.^4.*YY.*ZZ+ ...

phi.^2.*XX.*YY.*ZZ+(-4).*phi.*WW.*XX.*YY.*ZZ+(-1).*phi.^2.*WW.*XX.*YY.* ...

ZZ+3.*WW.^2.*XX.*YY.*ZZ+2.*phi.*WW.^2.*XX.*YY.*ZZ+(-1).*phi.^2.*WW.^2.* ...

XX.*YY.*ZZ+WW.^3.*XX.*YY.*ZZ+2.*phi.*WW.^3.*XX.*YY.*ZZ+phi.^2.*WW.^3.* ...

XX.*YY.*ZZ)+27.*(WW.^4+(-1).*WW.^3.*XX+WW.^3.*YY+(-2).*WW.^4.*YY+WW.^4.* ...

XX.*YY+(-1).*WW.^3.*ZZ+2.*WW.^3.*XX.*ZZ+(-1).*WW.^4.*XX.*ZZ+WW.^4.*YY.* ...

ZZ+(-1).*WW.^3.*XX.*YY.*ZZ).*((-1).*WW+2.*phi.*WW+(-1).*phi.^2.*WW+(-3) ...

.*WW.^2+2.*phi.*WW.^2+phi.^2.*WW.^2+(-4).*phi.*WW.^3+phi.^2.*WW.^3+(-1) ...

.*phi.^2.*WW.^4+XX+(-2).*phi.*XX+phi.^2.*XX+3.*WW.*XX+(-2).*phi.*WW.*XX+ ...

(-1).*phi.^2.*WW.*XX+4.*phi.*WW.^2.*XX+(-1).*phi.^2.*WW.^2.*XX+phi.^2.* ...

WW.^3.*XX+WW.^2.*YY+(-4).*phi.*WW.^2.*YY+2.*phi.^2.*WW.^2.*YY+3.*WW.^3.* ...

YY+2.*phi.*WW.^3.*YY+(-4).*phi.^2.*WW.^3.*YY+2.*phi.*WW.^4.*YY+2.* ...

phi.^2.*WW.^4.*YY+(-1).*WW.*XX.*YY+4.*phi.*WW.*XX.*YY+(-2).*phi.^2.*WW.* ...

XX.*YY+(-3).*WW.^2.*XX.*YY+(-2).*phi.*WW.^2.*XX.*YY+4.*phi.^2.*WW.^2.* ...

XX.*YY+(-2).*phi.*WW.^3.*XX.*YY+(-2).*phi.^2.*WW.^3.*XX.*YY+(-2).*phi.* ...

WW.*ZZ+2.*phi.^2.*WW.*ZZ+3.*WW.^2.*ZZ+(-2).*phi.*WW.^2.*ZZ+(-4).* ...

phi.^2.*WW.^2.*ZZ+WW.^3.*ZZ+4.*phi.*WW.^3.*ZZ+2.*phi.^2.*WW.^3.*ZZ+2.* ...

phi.*XX.*ZZ+(-2).*phi.^2.*XX.*ZZ+(-3).*WW.*XX.*ZZ+2.*phi.*WW.*XX.*ZZ+4.* ...

phi.^2.*WW.*XX.*ZZ+(-1).*WW.^2.*XX.*ZZ+(-4).*phi.*WW.^2.*XX.*ZZ+(-2).* ...

phi.^2.*WW.^2.*XX.*ZZ+(-1).*phi.^2.*WW.*YY.*ZZ+4.*phi.*WW.^2.*YY.*ZZ+ ...

phi.^2.*WW.^2.*YY.*ZZ+(-3).*WW.^3.*YY.*ZZ+(-2).*phi.*WW.^3.*YY.*ZZ+ ...

phi.^2.*WW.^3.*YY.*ZZ+(-1).*WW.^4.*YY.*ZZ+(-2).*phi.*WW.^4.*YY.*ZZ+(-1) ...

.*phi.^2.*WW.^4.*YY.*ZZ+phi.^2.*XX.*YY.*ZZ+(-4).*phi.*WW.*XX.*YY.*ZZ+( ...

-1).*phi.^2.*WW.*XX.*YY.*ZZ+3.*WW.^2.*XX.*YY.*ZZ+2.*phi.*WW.^2.*XX.*YY.* ...

ZZ+(-1).*phi.^2.*WW.^2.*XX.*YY.*ZZ+WW.^3.*XX.*YY.*ZZ+2.*phi.*WW.^3.*XX.* ...

YY.*ZZ+phi.^2.*WW.^3.*XX.*YY.*ZZ).^2).^2).^(1/2)).^(-1/3)+(-1/3).*2.^( ...

-1/3).*(WW+(-2).*phi.*WW+phi.^2.*WW+2.*phi.*WW.^2+(-2).*phi.^2.*WW.^2+ ...

phi.^2.*WW.^3+(-1).*XX+2.*phi.*XX+(-1).*phi.^2.*XX+(-2).*phi.*WW.*XX+2.* ...

phi.^2.*WW.*XX+(-1).*phi.^2.*WW.^2.*XX+phi.*WW.*YY+(-1).*phi.^2.*WW.*YY+ ...

(-1).*WW.^2.*YY+2.*phi.^2.*WW.^2.*YY+(-1).*phi.*WW.^3.*YY+(-1).*phi.^2.* ...

WW.^3.*YY+(-1).*phi.*XX.*YY+phi.^2.*XX.*YY+WW.*XX.*YY+(-2).*phi.^2.*WW.* ...

XX.*YY+phi.*WW.^2.*XX.*YY+phi.^2.*WW.^2.*XX.*YY+phi.*WW.*ZZ+(-1).* ...

phi.^2.*WW.*ZZ+(-1).*WW.^2.*ZZ+2.*phi.^2.*WW.^2.*ZZ+(-1).*phi.*WW.^3.* ...

ZZ+(-1).*phi.^2.*WW.^3.*ZZ+(-1).*phi.*XX.*ZZ+phi.^2.*XX.*ZZ+WW.*XX.*ZZ+( ...

-2).*phi.^2.*WW.*XX.*ZZ+phi.*WW.^2.*XX.*ZZ+phi.^2.*WW.^2.*XX.*ZZ+ ...

phi.^2.*WW.*YY.*ZZ+(-2).*phi.*WW.^2.*YY.*ZZ+(-2).*phi.^2.*WW.^2.*YY.*ZZ+ ...

WW.^3.*YY.*ZZ+2.*phi.*WW.^3.*YY.*ZZ+phi.^2.*WW.^3.*YY.*ZZ+(-1).*phi.^2.* ...

XX.*YY.*ZZ+2.*phi.*WW.*XX.*YY.*ZZ+2.*phi.^2.*WW.*XX.*YY.*ZZ+(-1).* ...

WW.^2.*XX.*YY.*ZZ+(-2).*phi.*WW.^2.*XX.*YY.*ZZ+(-1).*phi.^2.*WW.^2.*XX.* ...

YY.*ZZ).^(-1).*(27.*(WW+(-2).*phi.*WW+phi.^2.*WW+2.*phi.*WW.^2+(-2).* ...

phi.^2.*WW.^2+phi.^2.*WW.^3+(-1).*XX+2.*phi.*XX+(-1).*phi.^2.*XX+(-2).* ...

phi.*WW.*XX+2.*phi.^2.*WW.*XX+(-1).*phi.^2.*WW.^2.*XX+phi.*WW.*YY+(-1).* ...

phi.^2.*WW.*YY+(-1).*WW.^2.*YY+2.*phi.^2.*WW.^2.*YY+(-1).*phi.*WW.^3.* ...

YY+(-1).*phi.^2.*WW.^3.*YY+(-1).*phi.*XX.*YY+phi.^2.*XX.*YY+WW.*XX.*YY+( ...

-2).*phi.^2.*WW.*XX.*YY+phi.*WW.^2.*XX.*YY+phi.^2.*WW.^2.*XX.*YY+phi.* ...

WW.*ZZ+(-1).*phi.^2.*WW.*ZZ+(-1).*WW.^2.*ZZ+2.*phi.^2.*WW.^2.*ZZ+(-1).* ...

phi.*WW.^3.*ZZ+(-1).*phi.^2.*WW.^3.*ZZ+(-1).*phi.*XX.*ZZ+phi.^2.*XX.*ZZ+ ...

WW.*XX.*ZZ+(-2).*phi.^2.*WW.*XX.*ZZ+phi.*WW.^2.*XX.*ZZ+phi.^2.*WW.^2.* ...

XX.*ZZ+phi.^2.*WW.*YY.*ZZ+(-2).*phi.*WW.^2.*YY.*ZZ+(-2).*phi.^2.*WW.^2.* ...

YY.*ZZ+WW.^3.*YY.*ZZ+2.*phi.*WW.^3.*YY.*ZZ+phi.^2.*WW.^3.*YY.*ZZ+(-1).* ...

phi.^2.*XX.*YY.*ZZ+2.*phi.*WW.*XX.*YY.*ZZ+2.*phi.^2.*WW.*XX.*YY.*ZZ+(-1) ...

.*WW.^2.*XX.*YY.*ZZ+(-2).*phi.*WW.^2.*XX.*YY.*ZZ+(-1).*phi.^2.*WW.^2.* ...

XX.*YY.*ZZ).*((-3).*WW.^3+2.*phi.*WW.^3+(-1).*WW.^4+(-2).*phi.*WW.^4+3.* ...

WW.^2.*XX+(-2).*phi.*WW.^2.*XX+WW.^3.*XX+2.*phi.*WW.^3.*XX+(-1).*WW.^2.* ...

YY+2.*phi.*WW.^2.*YY+WW.^3.*YY+(-6).*phi.*WW.^3.*YY+4.*WW.^4.*YY+4.* ...

phi.*WW.^4.*YY+(-3).*WW.^3.*XX.*YY+2.*phi.*WW.^3.*XX.*YY+(-1).*WW.^4.* ...

XX.*YY+(-2).*phi.*WW.^4.*XX.*YY+WW.^2.*ZZ+(-2).*phi.*WW.^2.*ZZ+3.* ...

WW.^3.*ZZ+2.*phi.*WW.^3.*ZZ+(-4).*WW.^2.*XX.*ZZ+4.*phi.*WW.^2.*XX.*ZZ+( ...

-1).*WW.^3.*XX.*ZZ+(-6).*phi.*WW.^3.*XX.*ZZ+WW.^4.*XX.*ZZ+2.*phi.* ...

WW.^4.*XX.*ZZ+(-1).*WW.^3.*YY.*ZZ+2.*phi.*WW.^3.*YY.*ZZ+(-3).*WW.^4.* ...

YY.*ZZ+(-2).*phi.*WW.^4.*YY.*ZZ+WW.^2.*XX.*YY.*ZZ+(-2).*phi.*WW.^2.*XX.* ...

YY.*ZZ+3.*WW.^3.*XX.*YY.*ZZ+2.*phi.*WW.^3.*XX.*YY.*ZZ).^2+(-72).*(WW+( ...

-2).*phi.*WW+phi.^2.*WW+2.*phi.*WW.^2+(-2).*phi.^2.*WW.^2+phi.^2.*WW.^3+ ...

(-1).*XX+2.*phi.*XX+(-1).*phi.^2.*XX+(-2).*phi.*WW.*XX+2.*phi.^2.*WW.* ...

XX+(-1).*phi.^2.*WW.^2.*XX+phi.*WW.*YY+(-1).*phi.^2.*WW.*YY+(-1).* ...

WW.^2.*YY+2.*phi.^2.*WW.^2.*YY+(-1).*phi.*WW.^3.*YY+(-1).*phi.^2.* ...

WW.^3.*YY+(-1).*phi.*XX.*YY+phi.^2.*XX.*YY+WW.*XX.*YY+(-2).*phi.^2.*WW.* ...

XX.*YY+phi.*WW.^2.*XX.*YY+phi.^2.*WW.^2.*XX.*YY+phi.*WW.*ZZ+(-1).* ...

phi.^2.*WW.*ZZ+(-1).*WW.^2.*ZZ+2.*phi.^2.*WW.^2.*ZZ+(-1).*phi.*WW.^3.* ...

ZZ+(-1).*phi.^2.*WW.^3.*ZZ+(-1).*phi.*XX.*ZZ+phi.^2.*XX.*ZZ+WW.*XX.*ZZ+( ...

-2).*phi.^2.*WW.*XX.*ZZ+phi.*WW.^2.*XX.*ZZ+phi.^2.*WW.^2.*XX.*ZZ+ ...

phi.^2.*WW.*YY.*ZZ+(-2).*phi.*WW.^2.*YY.*ZZ+(-2).*phi.^2.*WW.^2.*YY.*ZZ+ ...

WW.^3.*YY.*ZZ+2.*phi.*WW.^3.*YY.*ZZ+phi.^2.*WW.^3.*YY.*ZZ+(-1).*phi.^2.* ...

XX.*YY.*ZZ+2.*phi.*WW.*XX.*YY.*ZZ+2.*phi.^2.*WW.*XX.*YY.*ZZ+(-1).* ...

WW.^2.*XX.*YY.*ZZ+(-2).*phi.*WW.^2.*XX.*YY.*ZZ+(-1).*phi.^2.*WW.^2.*XX.* ...

YY.*ZZ).*(WW.^4+(-1).*WW.^3.*XX+WW.^3.*YY+(-2).*WW.^4.*YY+WW.^4.*XX.*YY+ ...

(-1).*WW.^3.*ZZ+2.*WW.^3.*XX.*ZZ+(-1).*WW.^4.*XX.*ZZ+WW.^4.*YY.*ZZ+(-1) ...

.*WW.^3.*XX.*YY.*ZZ).*(3.*WW.^2+(-4).*phi.*WW.^2+phi.^2.*WW.^2+3.*WW.^3+ ...

2.*phi.*WW.^3+(-2).*phi.^2.*WW.^3+2.*phi.*WW.^4+phi.^2.*WW.^4+(-3).*WW.* ...

XX+4.*phi.*WW.*XX+(-1).*phi.^2.*WW.*XX+(-3).*WW.^2.*XX+(-2).*phi.* ...

WW.^2.*XX+2.*phi.^2.*WW.^2.*XX+(-2).*phi.*WW.^3.*XX+(-1).*phi.^2.* ...

WW.^3.*XX+(-1).*phi.*WW.*YY+phi.^2.*WW.*YY+WW.^2.*YY+2.*phi.*WW.^2.*YY+( ...

-4).*phi.^2.*WW.^2.*YY+(-5).*WW.^3.*YY+5.*phi.*WW.^3.*YY+5.*phi.^2.* ...

WW.^3.*YY+(-2).*WW.^4.*YY+(-6).*phi.*WW.^4.*YY+(-2).*phi.^2.*WW.^4.*YY+ ...

3.*WW.^2.*XX.*YY+(-5).*phi.*WW.^2.*XX.*YY+phi.^2.*WW.^2.*XX.*YY+3.* ...

WW.^3.*XX.*YY+4.*phi.*WW.^3.*XX.*YY+(-2).*phi.^2.*WW.^3.*XX.*YY+phi.* ...

WW.^4.*XX.*YY+phi.^2.*WW.^4.*XX.*YY+phi.*WW.*ZZ+(-1).*phi.^2.*WW.*ZZ+( ...

-3).*WW.^2.*ZZ+4.*phi.*WW.^2.*ZZ+2.*phi.^2.*WW.^2.*ZZ+(-3).*WW.^3.*ZZ+( ...

-5).*phi.*WW.^3.*ZZ+(-1).*phi.^2.*WW.^3.*ZZ+2.*WW.*XX.*ZZ+(-6).*phi.* ...

WW.*XX.*ZZ+2.*phi.^2.*WW.*XX.*ZZ+5.*WW.^2.*XX.*ZZ+5.*phi.*WW.^2.*XX.*ZZ+ ...

(-5).*phi.^2.*WW.^2.*XX.*ZZ+(-1).*WW.^3.*XX.*ZZ+2.*phi.*WW.^3.*XX.*ZZ+ ...

4.*phi.^2.*WW.^3.*XX.*ZZ+(-1).*phi.*WW.^4.*XX.*ZZ+(-1).*phi.^2.*WW.^4.* ...

XX.*ZZ+(-2).*phi.*WW.^2.*YY.*ZZ+phi.^2.*WW.^2.*YY.*ZZ+3.*WW.^3.*YY.*ZZ+( ...

-2).*phi.*WW.^3.*YY.*ZZ+(-2).*phi.^2.*WW.^3.*YY.*ZZ+3.*WW.^4.*YY.*ZZ+4.* ...

phi.*WW.^4.*YY.*ZZ+phi.^2.*WW.^4.*YY.*ZZ+2.*phi.*WW.*XX.*YY.*ZZ+(-1).* ...

phi.^2.*WW.*XX.*YY.*ZZ+(-3).*WW.^2.*XX.*YY.*ZZ+2.*phi.*WW.^2.*XX.*YY.* ...

ZZ+2.*phi.^2.*WW.^2.*XX.*YY.*ZZ+(-3).*WW.^3.*XX.*YY.*ZZ+(-4).*phi.* ...

WW.^3.*XX.*YY.*ZZ+(-1).*phi.^2.*WW.^3.*XX.*YY.*ZZ)+2.*(3.*WW.^2+(-4).* ...

phi.*WW.^2+phi.^2.*WW.^2+3.*WW.^3+2.*phi.*WW.^3+(-2).*phi.^2.*WW.^3+2.* ...

phi.*WW.^4+phi.^2.*WW.^4+(-3).*WW.*XX+4.*phi.*WW.*XX+(-1).*phi.^2.*WW.* ...

XX+(-3).*WW.^2.*XX+(-2).*phi.*WW.^2.*XX+2.*phi.^2.*WW.^2.*XX+(-2).*phi.* ...

WW.^3.*XX+(-1).*phi.^2.*WW.^3.*XX+(-1).*phi.*WW.*YY+phi.^2.*WW.*YY+ ...

WW.^2.*YY+2.*phi.*WW.^2.*YY+(-4).*phi.^2.*WW.^2.*YY+(-5).*WW.^3.*YY+5.* ...

phi.*WW.^3.*YY+5.*phi.^2.*WW.^3.*YY+(-2).*WW.^4.*YY+(-6).*phi.*WW.^4.* ...

YY+(-2).*phi.^2.*WW.^4.*YY+3.*WW.^2.*XX.*YY+(-5).*phi.*WW.^2.*XX.*YY+ ...

phi.^2.*WW.^2.*XX.*YY+3.*WW.^3.*XX.*YY+4.*phi.*WW.^3.*XX.*YY+(-2).* ...

phi.^2.*WW.^3.*XX.*YY+phi.*WW.^4.*XX.*YY+phi.^2.*WW.^4.*XX.*YY+phi.*WW.* ...

ZZ+(-1).*phi.^2.*WW.*ZZ+(-3).*WW.^2.*ZZ+4.*phi.*WW.^2.*ZZ+2.*phi.^2.* ...

WW.^2.*ZZ+(-3).*WW.^3.*ZZ+(-5).*phi.*WW.^3.*ZZ+(-1).*phi.^2.*WW.^3.*ZZ+ ...

2.*WW.*XX.*ZZ+(-6).*phi.*WW.*XX.*ZZ+2.*phi.^2.*WW.*XX.*ZZ+5.*WW.^2.*XX.* ...

ZZ+5.*phi.*WW.^2.*XX.*ZZ+(-5).*phi.^2.*WW.^2.*XX.*ZZ+(-1).*WW.^3.*XX.* ...

ZZ+2.*phi.*WW.^3.*XX.*ZZ+4.*phi.^2.*WW.^3.*XX.*ZZ+(-1).*phi.*WW.^4.*XX.* ...

ZZ+(-1).*phi.^2.*WW.^4.*XX.*ZZ+(-2).*phi.*WW.^2.*YY.*ZZ+phi.^2.*WW.^2.* ...

YY.*ZZ+3.*WW.^3.*YY.*ZZ+(-2).*phi.*WW.^3.*YY.*ZZ+(-2).*phi.^2.*WW.^3.* ...

YY.*ZZ+3.*WW.^4.*YY.*ZZ+4.*phi.*WW.^4.*YY.*ZZ+phi.^2.*WW.^4.*YY.*ZZ+2.* ...

phi.*WW.*XX.*YY.*ZZ+(-1).*phi.^2.*WW.*XX.*YY.*ZZ+(-3).*WW.^2.*XX.*YY.* ...

ZZ+2.*phi.*WW.^2.*XX.*YY.*ZZ+2.*phi.^2.*WW.^2.*XX.*YY.*ZZ+(-3).*WW.^3.* ...

XX.*YY.*ZZ+(-4).*phi.*WW.^3.*XX.*YY.*ZZ+(-1).*phi.^2.*WW.^3.*XX.*YY.*ZZ) ...

.^3+(-9).*((-3).*WW.^3+2.*phi.*WW.^3+(-1).*WW.^4+(-2).*phi.*WW.^4+3.* ...

WW.^2.*XX+(-2).*phi.*WW.^2.*XX+WW.^3.*XX+2.*phi.*WW.^3.*XX+(-1).*WW.^2.* ...

YY+2.*phi.*WW.^2.*YY+WW.^3.*YY+(-6).*phi.*WW.^3.*YY+4.*WW.^4.*YY+4.* ...

phi.*WW.^4.*YY+(-3).*WW.^3.*XX.*YY+2.*phi.*WW.^3.*XX.*YY+(-1).*WW.^4.* ...

XX.*YY+(-2).*phi.*WW.^4.*XX.*YY+WW.^2.*ZZ+(-2).*phi.*WW.^2.*ZZ+3.* ...

WW.^3.*ZZ+2.*phi.*WW.^3.*ZZ+(-4).*WW.^2.*XX.*ZZ+4.*phi.*WW.^2.*XX.*ZZ+( ...

-1).*WW.^3.*XX.*ZZ+(-6).*phi.*WW.^3.*XX.*ZZ+WW.^4.*XX.*ZZ+2.*phi.* ...

WW.^4.*XX.*ZZ+(-1).*WW.^3.*YY.*ZZ+2.*phi.*WW.^3.*YY.*ZZ+(-3).*WW.^4.* ...

YY.*ZZ+(-2).*phi.*WW.^4.*YY.*ZZ+WW.^2.*XX.*YY.*ZZ+(-2).*phi.*WW.^2.*XX.* ...

YY.*ZZ+3.*WW.^3.*XX.*YY.*ZZ+2.*phi.*WW.^3.*XX.*YY.*ZZ).*(3.*WW.^2+(-4).* ...

phi.*WW.^2+phi.^2.*WW.^2+3.*WW.^3+2.*phi.*WW.^3+(-2).*phi.^2.*WW.^3+2.* ...

phi.*WW.^4+phi.^2.*WW.^4+(-3).*WW.*XX+4.*phi.*WW.*XX+(-1).*phi.^2.*WW.* ...

XX+(-3).*WW.^2.*XX+(-2).*phi.*WW.^2.*XX+2.*phi.^2.*WW.^2.*XX+(-2).*phi.* ...

WW.^3.*XX+(-1).*phi.^2.*WW.^3.*XX+(-1).*phi.*WW.*YY+phi.^2.*WW.*YY+ ...

WW.^2.*YY+2.*phi.*WW.^2.*YY+(-4).*phi.^2.*WW.^2.*YY+(-5).*WW.^3.*YY+5.* ...

phi.*WW.^3.*YY+5.*phi.^2.*WW.^3.*YY+(-2).*WW.^4.*YY+(-6).*phi.*WW.^4.* ...

YY+(-2).*phi.^2.*WW.^4.*YY+3.*WW.^2.*XX.*YY+(-5).*phi.*WW.^2.*XX.*YY+ ...

phi.^2.*WW.^2.*XX.*YY+3.*WW.^3.*XX.*YY+4.*phi.*WW.^3.*XX.*YY+(-2).* ...

phi.^2.*WW.^3.*XX.*YY+phi.*WW.^4.*XX.*YY+phi.^2.*WW.^4.*XX.*YY+phi.*WW.* ...

ZZ+(-1).*phi.^2.*WW.*ZZ+(-3).*WW.^2.*ZZ+4.*phi.*WW.^2.*ZZ+2.*phi.^2.* ...

WW.^2.*ZZ+(-3).*WW.^3.*ZZ+(-5).*phi.*WW.^3.*ZZ+(-1).*phi.^2.*WW.^3.*ZZ+ ...

2.*WW.*XX.*ZZ+(-6).*phi.*WW.*XX.*ZZ+2.*phi.^2.*WW.*XX.*ZZ+5.*WW.^2.*XX.* ...

ZZ+5.*phi.*WW.^2.*XX.*ZZ+(-5).*phi.^2.*WW.^2.*XX.*ZZ+(-1).*WW.^3.*XX.* ...

ZZ+2.*phi.*WW.^3.*XX.*ZZ+4.*phi.^2.*WW.^3.*XX.*ZZ+(-1).*phi.*WW.^4.*XX.* ...

ZZ+(-1).*phi.^2.*WW.^4.*XX.*ZZ+(-2).*phi.*WW.^2.*YY.*ZZ+phi.^2.*WW.^2.* ...

YY.*ZZ+3.*WW.^3.*YY.*ZZ+(-2).*phi.*WW.^3.*YY.*ZZ+(-2).*phi.^2.*WW.^3.* ...

YY.*ZZ+3.*WW.^4.*YY.*ZZ+4.*phi.*WW.^4.*YY.*ZZ+phi.^2.*WW.^4.*YY.*ZZ+2.* ...

phi.*WW.*XX.*YY.*ZZ+(-1).*phi.^2.*WW.*XX.*YY.*ZZ+(-3).*WW.^2.*XX.*YY.* ...

ZZ+2.*phi.*WW.^2.*XX.*YY.*ZZ+2.*phi.^2.*WW.^2.*XX.*YY.*ZZ+(-3).*WW.^3.* ...

XX.*YY.*ZZ+(-4).*phi.*WW.^3.*XX.*YY.*ZZ+(-1).*phi.^2.*WW.^3.*XX.*YY.*ZZ) ...

.*((-1).*WW+2.*phi.*WW+(-1).*phi.^2.*WW+(-3).*WW.^2+2.*phi.*WW.^2+ ...

phi.^2.*WW.^2+(-4).*phi.*WW.^3+phi.^2.*WW.^3+(-1).*phi.^2.*WW.^4+XX+(-2) ...

.*phi.*XX+phi.^2.*XX+3.*WW.*XX+(-2).*phi.*WW.*XX+(-1).*phi.^2.*WW.*XX+ ...

4.*phi.*WW.^2.*XX+(-1).*phi.^2.*WW.^2.*XX+phi.^2.*WW.^3.*XX+WW.^2.*YY+( ...

-4).*phi.*WW.^2.*YY+2.*phi.^2.*WW.^2.*YY+3.*WW.^3.*YY+2.*phi.*WW.^3.*YY+ ...

(-4).*phi.^2.*WW.^3.*YY+2.*phi.*WW.^4.*YY+2.*phi.^2.*WW.^4.*YY+(-1).* ...

WW.*XX.*YY+4.*phi.*WW.*XX.*YY+(-2).*phi.^2.*WW.*XX.*YY+(-3).*WW.^2.*XX.* ...

YY+(-2).*phi.*WW.^2.*XX.*YY+4.*phi.^2.*WW.^2.*XX.*YY+(-2).*phi.*WW.^3.* ...

XX.*YY+(-2).*phi.^2.*WW.^3.*XX.*YY+(-2).*phi.*WW.*ZZ+2.*phi.^2.*WW.*ZZ+ ...

3.*WW.^2.*ZZ+(-2).*phi.*WW.^2.*ZZ+(-4).*phi.^2.*WW.^2.*ZZ+WW.^3.*ZZ+4.* ...

phi.*WW.^3.*ZZ+2.*phi.^2.*WW.^3.*ZZ+2.*phi.*XX.*ZZ+(-2).*phi.^2.*XX.*ZZ+ ...

(-3).*WW.*XX.*ZZ+2.*phi.*WW.*XX.*ZZ+4.*phi.^2.*WW.*XX.*ZZ+(-1).*WW.^2.* ...

XX.*ZZ+(-4).*phi.*WW.^2.*XX.*ZZ+(-2).*phi.^2.*WW.^2.*XX.*ZZ+(-1).* ...

phi.^2.*WW.*YY.*ZZ+4.*phi.*WW.^2.*YY.*ZZ+phi.^2.*WW.^2.*YY.*ZZ+(-3).* ...

WW.^3.*YY.*ZZ+(-2).*phi.*WW.^3.*YY.*ZZ+phi.^2.*WW.^3.*YY.*ZZ+(-1).* ...

WW.^4.*YY.*ZZ+(-2).*phi.*WW.^4.*YY.*ZZ+(-1).*phi.^2.*WW.^4.*YY.*ZZ+ ...

phi.^2.*XX.*YY.*ZZ+(-4).*phi.*WW.*XX.*YY.*ZZ+(-1).*phi.^2.*WW.*XX.*YY.* ...

ZZ+3.*WW.^2.*XX.*YY.*ZZ+2.*phi.*WW.^2.*XX.*YY.*ZZ+(-1).*phi.^2.*WW.^2.* ...

XX.*YY.*ZZ+WW.^3.*XX.*YY.*ZZ+2.*phi.*WW.^3.*XX.*YY.*ZZ+phi.^2.*WW.^3.* ...

XX.*YY.*ZZ)+27.*(WW.^4+(-1).*WW.^3.*XX+WW.^3.*YY+(-2).*WW.^4.*YY+WW.^4.* ...

XX.*YY+(-1).*WW.^3.*ZZ+2.*WW.^3.*XX.*ZZ+(-1).*WW.^4.*XX.*ZZ+WW.^4.*YY.* ...

ZZ+(-1).*WW.^3.*XX.*YY.*ZZ).*((-1).*WW+2.*phi.*WW+(-1).*phi.^2.*WW+(-3) ...

.*WW.^2+2.*phi.*WW.^2+phi.^2.*WW.^2+(-4).*phi.*WW.^3+phi.^2.*WW.^3+(-1) ...

.*phi.^2.*WW.^4+XX+(-2).*phi.*XX+phi.^2.*XX+3.*WW.*XX+(-2).*phi.*WW.*XX+ ...

(-1).*phi.^2.*WW.*XX+4.*phi.*WW.^2.*XX+(-1).*phi.^2.*WW.^2.*XX+phi.^2.* ...

WW.^3.*XX+WW.^2.*YY+(-4).*phi.*WW.^2.*YY+2.*phi.^2.*WW.^2.*YY+3.*WW.^3.* ...

YY+2.*phi.*WW.^3.*YY+(-4).*phi.^2.*WW.^3.*YY+2.*phi.*WW.^4.*YY+2.* ...

phi.^2.*WW.^4.*YY+(-1).*WW.*XX.*YY+4.*phi.*WW.*XX.*YY+(-2).*phi.^2.*WW.* ...

XX.*YY+(-3).*WW.^2.*XX.*YY+(-2).*phi.*WW.^2.*XX.*YY+4.*phi.^2.*WW.^2.* ...

XX.*YY+(-2).*phi.*WW.^3.*XX.*YY+(-2).*phi.^2.*WW.^3.*XX.*YY+(-2).*phi.* ...

WW.*ZZ+2.*phi.^2.*WW.*ZZ+3.*WW.^2.*ZZ+(-2).*phi.*WW.^2.*ZZ+(-4).* ...

phi.^2.*WW.^2.*ZZ+WW.^3.*ZZ+4.*phi.*WW.^3.*ZZ+2.*phi.^2.*WW.^3.*ZZ+2.* ...

phi.*XX.*ZZ+(-2).*phi.^2.*XX.*ZZ+(-3).*WW.*XX.*ZZ+2.*phi.*WW.*XX.*ZZ+4.* ...

phi.^2.*WW.*XX.*ZZ+(-1).*WW.^2.*XX.*ZZ+(-4).*phi.*WW.^2.*XX.*ZZ+(-2).* ...

phi.^2.*WW.^2.*XX.*ZZ+(-1).*phi.^2.*WW.*YY.*ZZ+4.*phi.*WW.^2.*YY.*ZZ+ ...

phi.^2.*WW.^2.*YY.*ZZ+(-3).*WW.^3.*YY.*ZZ+(-2).*phi.*WW.^3.*YY.*ZZ+ ...

phi.^2.*WW.^3.*YY.*ZZ+(-1).*WW.^4.*YY.*ZZ+(-2).*phi.*WW.^4.*YY.*ZZ+(-1) ...

.*phi.^2.*WW.^4.*YY.*ZZ+phi.^2.*XX.*YY.*ZZ+(-4).*phi.*WW.*XX.*YY.*ZZ+( ...

-1).*phi.^2.*WW.*XX.*YY.*ZZ+3.*WW.^2.*XX.*YY.*ZZ+2.*phi.*WW.^2.*XX.*YY.* ...

ZZ+(-1).*phi.^2.*WW.^2.*XX.*YY.*ZZ+WW.^3.*XX.*YY.*ZZ+2.*phi.*WW.^3.*XX.* ...

YY.*ZZ+phi.^2.*WW.^3.*XX.*YY.*ZZ).^2+((-4).*(12.*(WW+(-2).*phi.*WW+ ...

phi.^2.*WW+2.*phi.*WW.^2+(-2).*phi.^2.*WW.^2+phi.^2.*WW.^3+(-1).*XX+2.* ...

phi.*XX+(-1).*phi.^2.*XX+(-2).*phi.*WW.*XX+2.*phi.^2.*WW.*XX+(-1).* ...

phi.^2.*WW.^2.*XX+phi.*WW.*YY+(-1).*phi.^2.*WW.*YY+(-1).*WW.^2.*YY+2.* ...

phi.^2.*WW.^2.*YY+(-1).*phi.*WW.^3.*YY+(-1).*phi.^2.*WW.^3.*YY+(-1).* ...

phi.*XX.*YY+phi.^2.*XX.*YY+WW.*XX.*YY+(-2).*phi.^2.*WW.*XX.*YY+phi.* ...

WW.^2.*XX.*YY+phi.^2.*WW.^2.*XX.*YY+phi.*WW.*ZZ+(-1).*phi.^2.*WW.*ZZ+( ...

-1).*WW.^2.*ZZ+2.*phi.^2.*WW.^2.*ZZ+(-1).*phi.*WW.^3.*ZZ+(-1).*phi.^2.* ...

WW.^3.*ZZ+(-1).*phi.*XX.*ZZ+phi.^2.*XX.*ZZ+WW.*XX.*ZZ+(-2).*phi.^2.*WW.* ...

XX.*ZZ+phi.*WW.^2.*XX.*ZZ+phi.^2.*WW.^2.*XX.*ZZ+phi.^2.*WW.*YY.*ZZ+(-2) ...

.*phi.*WW.^2.*YY.*ZZ+(-2).*phi.^2.*WW.^2.*YY.*ZZ+WW.^3.*YY.*ZZ+2.*phi.* ...

WW.^3.*YY.*ZZ+phi.^2.*WW.^3.*YY.*ZZ+(-1).*phi.^2.*XX.*YY.*ZZ+2.*phi.* ...

WW.*XX.*YY.*ZZ+2.*phi.^2.*WW.*XX.*YY.*ZZ+(-1).*WW.^2.*XX.*YY.*ZZ+(-2).* ...

phi.*WW.^2.*XX.*YY.*ZZ+(-1).*phi.^2.*WW.^2.*XX.*YY.*ZZ).*(WW.^4+(-1).* ...

WW.^3.*XX+WW.^3.*YY+(-2).*WW.^4.*YY+WW.^4.*XX.*YY+(-1).*WW.^3.*ZZ+2.* ...

WW.^3.*XX.*ZZ+(-1).*WW.^4.*XX.*ZZ+WW.^4.*YY.*ZZ+(-1).*WW.^3.*XX.*YY.*ZZ) ...

+(3.*WW.^2+(-4).*phi.*WW.^2+phi.^2.*WW.^2+3.*WW.^3+2.*phi.*WW.^3+(-2).* ...

phi.^2.*WW.^3+2.*phi.*WW.^4+phi.^2.*WW.^4+(-3).*WW.*XX+4.*phi.*WW.*XX+( ...

-1).*phi.^2.*WW.*XX+(-3).*WW.^2.*XX+(-2).*phi.*WW.^2.*XX+2.*phi.^2.* ...

WW.^2.*XX+(-2).*phi.*WW.^3.*XX+(-1).*phi.^2.*WW.^3.*XX+(-1).*phi.*WW.* ...

YY+phi.^2.*WW.*YY+WW.^2.*YY+2.*phi.*WW.^2.*YY+(-4).*phi.^2.*WW.^2.*YY+( ...

-5).*WW.^3.*YY+5.*phi.*WW.^3.*YY+5.*phi.^2.*WW.^3.*YY+(-2).*WW.^4.*YY+( ...

-6).*phi.*WW.^4.*YY+(-2).*phi.^2.*WW.^4.*YY+3.*WW.^2.*XX.*YY+(-5).*phi.* ...

WW.^2.*XX.*YY+phi.^2.*WW.^2.*XX.*YY+3.*WW.^3.*XX.*YY+4.*phi.*WW.^3.*XX.* ...

YY+(-2).*phi.^2.*WW.^3.*XX.*YY+phi.*WW.^4.*XX.*YY+phi.^2.*WW.^4.*XX.*YY+ ...

phi.*WW.*ZZ+(-1).*phi.^2.*WW.*ZZ+(-3).*WW.^2.*ZZ+4.*phi.*WW.^2.*ZZ+2.* ...

phi.^2.*WW.^2.*ZZ+(-3).*WW.^3.*ZZ+(-5).*phi.*WW.^3.*ZZ+(-1).*phi.^2.* ...

WW.^3.*ZZ+2.*WW.*XX.*ZZ+(-6).*phi.*WW.*XX.*ZZ+2.*phi.^2.*WW.*XX.*ZZ+5.* ...

WW.^2.*XX.*ZZ+5.*phi.*WW.^2.*XX.*ZZ+(-5).*phi.^2.*WW.^2.*XX.*ZZ+(-1).* ...

WW.^3.*XX.*ZZ+2.*phi.*WW.^3.*XX.*ZZ+4.*phi.^2.*WW.^3.*XX.*ZZ+(-1).*phi.* ...

WW.^4.*XX.*ZZ+(-1).*phi.^2.*WW.^4.*XX.*ZZ+(-2).*phi.*WW.^2.*YY.*ZZ+ ...

phi.^2.*WW.^2.*YY.*ZZ+3.*WW.^3.*YY.*ZZ+(-2).*phi.*WW.^3.*YY.*ZZ+(-2).* ...

phi.^2.*WW.^3.*YY.*ZZ+3.*WW.^4.*YY.*ZZ+4.*phi.*WW.^4.*YY.*ZZ+phi.^2.* ...

WW.^4.*YY.*ZZ+2.*phi.*WW.*XX.*YY.*ZZ+(-1).*phi.^2.*WW.*XX.*YY.*ZZ+(-3).* ...

WW.^2.*XX.*YY.*ZZ+2.*phi.*WW.^2.*XX.*YY.*ZZ+2.*phi.^2.*WW.^2.*XX.*YY.* ...

ZZ+(-3).*WW.^3.*XX.*YY.*ZZ+(-4).*phi.*WW.^3.*XX.*YY.*ZZ+(-1).*phi.^2.* ...

WW.^3.*XX.*YY.*ZZ).^2+(-3).*((-3).*WW.^3+2.*phi.*WW.^3+(-1).*WW.^4+(-2) ...

.*phi.*WW.^4+3.*WW.^2.*XX+(-2).*phi.*WW.^2.*XX+WW.^3.*XX+2.*phi.*WW.^3.* ...

XX+(-1).*WW.^2.*YY+2.*phi.*WW.^2.*YY+WW.^3.*YY+(-6).*phi.*WW.^3.*YY+4.* ...

WW.^4.*YY+4.*phi.*WW.^4.*YY+(-3).*WW.^3.*XX.*YY+2.*phi.*WW.^3.*XX.*YY+( ...

-1).*WW.^4.*XX.*YY+(-2).*phi.*WW.^4.*XX.*YY+WW.^2.*ZZ+(-2).*phi.*WW.^2.* ...

ZZ+3.*WW.^3.*ZZ+2.*phi.*WW.^3.*ZZ+(-4).*WW.^2.*XX.*ZZ+4.*phi.*WW.^2.* ...

XX.*ZZ+(-1).*WW.^3.*XX.*ZZ+(-6).*phi.*WW.^3.*XX.*ZZ+WW.^4.*XX.*ZZ+2.* ...

phi.*WW.^4.*XX.*ZZ+(-1).*WW.^3.*YY.*ZZ+2.*phi.*WW.^3.*YY.*ZZ+(-3).* ...

WW.^4.*YY.*ZZ+(-2).*phi.*WW.^4.*YY.*ZZ+WW.^2.*XX.*YY.*ZZ+(-2).*phi.* ...

WW.^2.*XX.*YY.*ZZ+3.*WW.^3.*XX.*YY.*ZZ+2.*phi.*WW.^3.*XX.*YY.*ZZ).*((-1) ...

.*WW+2.*phi.*WW+(-1).*phi.^2.*WW+(-3).*WW.^2+2.*phi.*WW.^2+phi.^2.* ...

WW.^2+(-4).*phi.*WW.^3+phi.^2.*WW.^3+(-1).*phi.^2.*WW.^4+XX+(-2).*phi.* ...

XX+phi.^2.*XX+3.*WW.*XX+(-2).*phi.*WW.*XX+(-1).*phi.^2.*WW.*XX+4.*phi.* ...

WW.^2.*XX+(-1).*phi.^2.*WW.^2.*XX+phi.^2.*WW.^3.*XX+WW.^2.*YY+(-4).* ...

phi.*WW.^2.*YY+2.*phi.^2.*WW.^2.*YY+3.*WW.^3.*YY+2.*phi.*WW.^3.*YY+(-4) ...

.*phi.^2.*WW.^3.*YY+2.*phi.*WW.^4.*YY+2.*phi.^2.*WW.^4.*YY+(-1).*WW.* ...

XX.*YY+4.*phi.*WW.*XX.*YY+(-2).*phi.^2.*WW.*XX.*YY+(-3).*WW.^2.*XX.*YY+( ...

-2).*phi.*WW.^2.*XX.*YY+4.*phi.^2.*WW.^2.*XX.*YY+(-2).*phi.*WW.^3.*XX.* ...

YY+(-2).*phi.^2.*WW.^3.*XX.*YY+(-2).*phi.*WW.*ZZ+2.*phi.^2.*WW.*ZZ+3.* ...

WW.^2.*ZZ+(-2).*phi.*WW.^2.*ZZ+(-4).*phi.^2.*WW.^2.*ZZ+WW.^3.*ZZ+4.* ...

phi.*WW.^3.*ZZ+2.*phi.^2.*WW.^3.*ZZ+2.*phi.*XX.*ZZ+(-2).*phi.^2.*XX.*ZZ+ ...

(-3).*WW.*XX.*ZZ+2.*phi.*WW.*XX.*ZZ+4.*phi.^2.*WW.*XX.*ZZ+(-1).*WW.^2.* ...

XX.*ZZ+(-4).*phi.*WW.^2.*XX.*ZZ+(-2).*phi.^2.*WW.^2.*XX.*ZZ+(-1).* ...

phi.^2.*WW.*YY.*ZZ+4.*phi.*WW.^2.*YY.*ZZ+phi.^2.*WW.^2.*YY.*ZZ+(-3).* ...

WW.^3.*YY.*ZZ+(-2).*phi.*WW.^3.*YY.*ZZ+phi.^2.*WW.^3.*YY.*ZZ+(-1).* ...

WW.^4.*YY.*ZZ+(-2).*phi.*WW.^4.*YY.*ZZ+(-1).*phi.^2.*WW.^4.*YY.*ZZ+ ...

phi.^2.*XX.*YY.*ZZ+(-4).*phi.*WW.*XX.*YY.*ZZ+(-1).*phi.^2.*WW.*XX.*YY.* ...

ZZ+3.*WW.^2.*XX.*YY.*ZZ+2.*phi.*WW.^2.*XX.*YY.*ZZ+(-1).*phi.^2.*WW.^2.* ...

XX.*YY.*ZZ+WW.^3.*XX.*YY.*ZZ+2.*phi.*WW.^3.*XX.*YY.*ZZ+phi.^2.*WW.^3.* ...

XX.*YY.*ZZ)).^3+(27.*(WW+(-2).*phi.*WW+phi.^2.*WW+2.*phi.*WW.^2+(-2).* ...

phi.^2.*WW.^2+phi.^2.*WW.^3+(-1).*XX+2.*phi.*XX+(-1).*phi.^2.*XX+(-2).* ...

phi.*WW.*XX+2.*phi.^2.*WW.*XX+(-1).*phi.^2.*WW.^2.*XX+phi.*WW.*YY+(-1).* ...

phi.^2.*WW.*YY+(-1).*WW.^2.*YY+2.*phi.^2.*WW.^2.*YY+(-1).*phi.*WW.^3.* ...

YY+(-1).*phi.^2.*WW.^3.*YY+(-1).*phi.*XX.*YY+phi.^2.*XX.*YY+WW.*XX.*YY+( ...

-2).*phi.^2.*WW.*XX.*YY+phi.*WW.^2.*XX.*YY+phi.^2.*WW.^2.*XX.*YY+phi.* ...

WW.*ZZ+(-1).*phi.^2.*WW.*ZZ+(-1).*WW.^2.*ZZ+2.*phi.^2.*WW.^2.*ZZ+(-1).* ...

phi.*WW.^3.*ZZ+(-1).*phi.^2.*WW.^3.*ZZ+(-1).*phi.*XX.*ZZ+phi.^2.*XX.*ZZ+ ...

WW.*XX.*ZZ+(-2).*phi.^2.*WW.*XX.*ZZ+phi.*WW.^2.*XX.*ZZ+phi.^2.*WW.^2.* ...

XX.*ZZ+phi.^2.*WW.*YY.*ZZ+(-2).*phi.*WW.^2.*YY.*ZZ+(-2).*phi.^2.*WW.^2.* ...

YY.*ZZ+WW.^3.*YY.*ZZ+2.*phi.*WW.^3.*YY.*ZZ+phi.^2.*WW.^3.*YY.*ZZ+(-1).* ...

phi.^2.*XX.*YY.*ZZ+2.*phi.*WW.*XX.*YY.*ZZ+2.*phi.^2.*WW.*XX.*YY.*ZZ+(-1) ...

.*WW.^2.*XX.*YY.*ZZ+(-2).*phi.*WW.^2.*XX.*YY.*ZZ+(-1).*phi.^2.*WW.^2.* ...

XX.*YY.*ZZ).*((-3).*WW.^3+2.*phi.*WW.^3+(-1).*WW.^4+(-2).*phi.*WW.^4+3.* ...

WW.^2.*XX+(-2).*phi.*WW.^2.*XX+WW.^3.*XX+2.*phi.*WW.^3.*XX+(-1).*WW.^2.* ...

YY+2.*phi.*WW.^2.*YY+WW.^3.*YY+(-6).*phi.*WW.^3.*YY+4.*WW.^4.*YY+4.* ...

phi.*WW.^4.*YY+(-3).*WW.^3.*XX.*YY+2.*phi.*WW.^3.*XX.*YY+(-1).*WW.^4.* ...

XX.*YY+(-2).*phi.*WW.^4.*XX.*YY+WW.^2.*ZZ+(-2).*phi.*WW.^2.*ZZ+3.* ...

WW.^3.*ZZ+2.*phi.*WW.^3.*ZZ+(-4).*WW.^2.*XX.*ZZ+4.*phi.*WW.^2.*XX.*ZZ+( ...

-1).*WW.^3.*XX.*ZZ+(-6).*phi.*WW.^3.*XX.*ZZ+WW.^4.*XX.*ZZ+2.*phi.* ...

WW.^4.*XX.*ZZ+(-1).*WW.^3.*YY.*ZZ+2.*phi.*WW.^3.*YY.*ZZ+(-3).*WW.^4.* ...

YY.*ZZ+(-2).*phi.*WW.^4.*YY.*ZZ+WW.^2.*XX.*YY.*ZZ+(-2).*phi.*WW.^2.*XX.* ...

YY.*ZZ+3.*WW.^3.*XX.*YY.*ZZ+2.*phi.*WW.^3.*XX.*YY.*ZZ).^2+(-72).*(WW+( ...

-2).*phi.*WW+phi.^2.*WW+2.*phi.*WW.^2+(-2).*phi.^2.*WW.^2+phi.^2.*WW.^3+ ...

(-1).*XX+2.*phi.*XX+(-1).*phi.^2.*XX+(-2).*phi.*WW.*XX+2.*phi.^2.*WW.* ...

XX+(-1).*phi.^2.*WW.^2.*XX+phi.*WW.*YY+(-1).*phi.^2.*WW.*YY+(-1).* ...

WW.^2.*YY+2.*phi.^2.*WW.^2.*YY+(-1).*phi.*WW.^3.*YY+(-1).*phi.^2.* ...

WW.^3.*YY+(-1).*phi.*XX.*YY+phi.^2.*XX.*YY+WW.*XX.*YY+(-2).*phi.^2.*WW.* ...

XX.*YY+phi.*WW.^2.*XX.*YY+phi.^2.*WW.^2.*XX.*YY+phi.*WW.*ZZ+(-1).* ...

phi.^2.*WW.*ZZ+(-1).*WW.^2.*ZZ+2.*phi.^2.*WW.^2.*ZZ+(-1).*phi.*WW.^3.* ...

ZZ+(-1).*phi.^2.*WW.^3.*ZZ+(-1).*phi.*XX.*ZZ+phi.^2.*XX.*ZZ+WW.*XX.*ZZ+( ...

-2).*phi.^2.*WW.*XX.*ZZ+phi.*WW.^2.*XX.*ZZ+phi.^2.*WW.^2.*XX.*ZZ+ ...

phi.^2.*WW.*YY.*ZZ+(-2).*phi.*WW.^2.*YY.*ZZ+(-2).*phi.^2.*WW.^2.*YY.*ZZ+ ...

WW.^3.*YY.*ZZ+2.*phi.*WW.^3.*YY.*ZZ+phi.^2.*WW.^3.*YY.*ZZ+(-1).*phi.^2.* ...

XX.*YY.*ZZ+2.*phi.*WW.*XX.*YY.*ZZ+2.*phi.^2.*WW.*XX.*YY.*ZZ+(-1).* ...

WW.^2.*XX.*YY.*ZZ+(-2).*phi.*WW.^2.*XX.*YY.*ZZ+(-1).*phi.^2.*WW.^2.*XX.* ...

YY.*ZZ).*(WW.^4+(-1).*WW.^3.*XX+WW.^3.*YY+(-2).*WW.^4.*YY+WW.^4.*XX.*YY+ ...

(-1).*WW.^3.*ZZ+2.*WW.^3.*XX.*ZZ+(-1).*WW.^4.*XX.*ZZ+WW.^4.*YY.*ZZ+(-1) ...

.*WW.^3.*XX.*YY.*ZZ).*(3.*WW.^2+(-4).*phi.*WW.^2+phi.^2.*WW.^2+3.*WW.^3+ ...

2.*phi.*WW.^3+(-2).*phi.^2.*WW.^3+2.*phi.*WW.^4+phi.^2.*WW.^4+(-3).*WW.* ...

XX+4.*phi.*WW.*XX+(-1).*phi.^2.*WW.*XX+(-3).*WW.^2.*XX+(-2).*phi.* ...

WW.^2.*XX+2.*phi.^2.*WW.^2.*XX+(-2).*phi.*WW.^3.*XX+(-1).*phi.^2.* ...

WW.^3.*XX+(-1).*phi.*WW.*YY+phi.^2.*WW.*YY+WW.^2.*YY+2.*phi.*WW.^2.*YY+( ...

-4).*phi.^2.*WW.^2.*YY+(-5).*WW.^3.*YY+5.*phi.*WW.^3.*YY+5.*phi.^2.* ...

WW.^3.*YY+(-2).*WW.^4.*YY+(-6).*phi.*WW.^4.*YY+(-2).*phi.^2.*WW.^4.*YY+ ...

3.*WW.^2.*XX.*YY+(-5).*phi.*WW.^2.*XX.*YY+phi.^2.*WW.^2.*XX.*YY+3.* ...

WW.^3.*XX.*YY+4.*phi.*WW.^3.*XX.*YY+(-2).*phi.^2.*WW.^3.*XX.*YY+phi.* ...

WW.^4.*XX.*YY+phi.^2.*WW.^4.*XX.*YY+phi.*WW.*ZZ+(-1).*phi.^2.*WW.*ZZ+( ...

-3).*WW.^2.*ZZ+4.*phi.*WW.^2.*ZZ+2.*phi.^2.*WW.^2.*ZZ+(-3).*WW.^3.*ZZ+( ...

-5).*phi.*WW.^3.*ZZ+(-1).*phi.^2.*WW.^3.*ZZ+2.*WW.*XX.*ZZ+(-6).*phi.* ...

WW.*XX.*ZZ+2.*phi.^2.*WW.*XX.*ZZ+5.*WW.^2.*XX.*ZZ+5.*phi.*WW.^2.*XX.*ZZ+ ...

(-5).*phi.^2.*WW.^2.*XX.*ZZ+(-1).*WW.^3.*XX.*ZZ+2.*phi.*WW.^3.*XX.*ZZ+ ...

4.*phi.^2.*WW.^3.*XX.*ZZ+(-1).*phi.*WW.^4.*XX.*ZZ+(-1).*phi.^2.*WW.^4.* ...

XX.*ZZ+(-2).*phi.*WW.^2.*YY.*ZZ+phi.^2.*WW.^2.*YY.*ZZ+3.*WW.^3.*YY.*ZZ+( ...

-2).*phi.*WW.^3.*YY.*ZZ+(-2).*phi.^2.*WW.^3.*YY.*ZZ+3.*WW.^4.*YY.*ZZ+4.* ...

phi.*WW.^4.*YY.*ZZ+phi.^2.*WW.^4.*YY.*ZZ+2.*phi.*WW.*XX.*YY.*ZZ+(-1).* ...

phi.^2.*WW.*XX.*YY.*ZZ+(-3).*WW.^2.*XX.*YY.*ZZ+2.*phi.*WW.^2.*XX.*YY.* ...

ZZ+2.*phi.^2.*WW.^2.*XX.*YY.*ZZ+(-3).*WW.^3.*XX.*YY.*ZZ+(-4).*phi.* ...

WW.^3.*XX.*YY.*ZZ+(-1).*phi.^2.*WW.^3.*XX.*YY.*ZZ)+2.*(3.*WW.^2+(-4).* ...

phi.*WW.^2+phi.^2.*WW.^2+3.*WW.^3+2.*phi.*WW.^3+(-2).*phi.^2.*WW.^3+2.* ...

phi.*WW.^4+phi.^2.*WW.^4+(-3).*WW.*XX+4.*phi.*WW.*XX+(-1).*phi.^2.*WW.* ...

XX+(-3).*WW.^2.*XX+(-2).*phi.*WW.^2.*XX+2.*phi.^2.*WW.^2.*XX+(-2).*phi.* ...

WW.^3.*XX+(-1).*phi.^2.*WW.^3.*XX+(-1).*phi.*WW.*YY+phi.^2.*WW.*YY+ ...

WW.^2.*YY+2.*phi.*WW.^2.*YY+(-4).*phi.^2.*WW.^2.*YY+(-5).*WW.^3.*YY+5.* ...

phi.*WW.^3.*YY+5.*phi.^2.*WW.^3.*YY+(-2).*WW.^4.*YY+(-6).*phi.*WW.^4.* ...

YY+(-2).*phi.^2.*WW.^4.*YY+3.*WW.^2.*XX.*YY+(-5).*phi.*WW.^2.*XX.*YY+ ...

phi.^2.*WW.^2.*XX.*YY+3.*WW.^3.*XX.*YY+4.*phi.*WW.^3.*XX.*YY+(-2).* ...

phi.^2.*WW.^3.*XX.*YY+phi.*WW.^4.*XX.*YY+phi.^2.*WW.^4.*XX.*YY+phi.*WW.* ...

ZZ+(-1).*phi.^2.*WW.*ZZ+(-3).*WW.^2.*ZZ+4.*phi.*WW.^2.*ZZ+2.*phi.^2.* ...

WW.^2.*ZZ+(-3).*WW.^3.*ZZ+(-5).*phi.*WW.^3.*ZZ+(-1).*phi.^2.*WW.^3.*ZZ+ ...

2.*WW.*XX.*ZZ+(-6).*phi.*WW.*XX.*ZZ+2.*phi.^2.*WW.*XX.*ZZ+5.*WW.^2.*XX.* ...

ZZ+5.*phi.*WW.^2.*XX.*ZZ+(-5).*phi.^2.*WW.^2.*XX.*ZZ+(-1).*WW.^3.*XX.* ...

ZZ+2.*phi.*WW.^3.*XX.*ZZ+4.*phi.^2.*WW.^3.*XX.*ZZ+(-1).*phi.*WW.^4.*XX.* ...

ZZ+(-1).*phi.^2.*WW.^4.*XX.*ZZ+(-2).*phi.*WW.^2.*YY.*ZZ+phi.^2.*WW.^2.* ...

YY.*ZZ+3.*WW.^3.*YY.*ZZ+(-2).*phi.*WW.^3.*YY.*ZZ+(-2).*phi.^2.*WW.^3.* ...

YY.*ZZ+3.*WW.^4.*YY.*ZZ+4.*phi.*WW.^4.*YY.*ZZ+phi.^2.*WW.^4.*YY.*ZZ+2.* ...

phi.*WW.*XX.*YY.*ZZ+(-1).*phi.^2.*WW.*XX.*YY.*ZZ+(-3).*WW.^2.*XX.*YY.* ...

ZZ+2.*phi.*WW.^2.*XX.*YY.*ZZ+2.*phi.^2.*WW.^2.*XX.*YY.*ZZ+(-3).*WW.^3.* ...

XX.*YY.*ZZ+(-4).*phi.*WW.^3.*XX.*YY.*ZZ+(-1).*phi.^2.*WW.^3.*XX.*YY.*ZZ) ...

.^3+(-9).*((-3).*WW.^3+2.*phi.*WW.^3+(-1).*WW.^4+(-2).*phi.*WW.^4+3.* ...

WW.^2.*XX+(-2).*phi.*WW.^2.*XX+WW.^3.*XX+2.*phi.*WW.^3.*XX+(-1).*WW.^2.* ...

YY+2.*phi.*WW.^2.*YY+WW.^3.*YY+(-6).*phi.*WW.^3.*YY+4.*WW.^4.*YY+4.* ...

phi.*WW.^4.*YY+(-3).*WW.^3.*XX.*YY+2.*phi.*WW.^3.*XX.*YY+(-1).*WW.^4.* ...

XX.*YY+(-2).*phi.*WW.^4.*XX.*YY+WW.^2.*ZZ+(-2).*phi.*WW.^2.*ZZ+3.* ...

WW.^3.*ZZ+2.*phi.*WW.^3.*ZZ+(-4).*WW.^2.*XX.*ZZ+4.*phi.*WW.^2.*XX.*ZZ+( ...

-1).*WW.^3.*XX.*ZZ+(-6).*phi.*WW.^3.*XX.*ZZ+WW.^4.*XX.*ZZ+2.*phi.* ...

WW.^4.*XX.*ZZ+(-1).*WW.^3.*YY.*ZZ+2.*phi.*WW.^3.*YY.*ZZ+(-3).*WW.^4.* ...

YY.*ZZ+(-2).*phi.*WW.^4.*YY.*ZZ+WW.^2.*XX.*YY.*ZZ+(-2).*phi.*WW.^2.*XX.* ...

YY.*ZZ+3.*WW.^3.*XX.*YY.*ZZ+2.*phi.*WW.^3.*XX.*YY.*ZZ).*(3.*WW.^2+(-4).* ...

phi.*WW.^2+phi.^2.*WW.^2+3.*WW.^3+2.*phi.*WW.^3+(-2).*phi.^2.*WW.^3+2.* ...

phi.*WW.^4+phi.^2.*WW.^4+(-3).*WW.*XX+4.*phi.*WW.*XX+(-1).*phi.^2.*WW.* ...

XX+(-3).*WW.^2.*XX+(-2).*phi.*WW.^2.*XX+2.*phi.^2.*WW.^2.*XX+(-2).*phi.* ...

WW.^3.*XX+(-1).*phi.^2.*WW.^3.*XX+(-1).*phi.*WW.*YY+phi.^2.*WW.*YY+ ...

WW.^2.*YY+2.*phi.*WW.^2.*YY+(-4).*phi.^2.*WW.^2.*YY+(-5).*WW.^3.*YY+5.* ...

phi.*WW.^3.*YY+5.*phi.^2.*WW.^3.*YY+(-2).*WW.^4.*YY+(-6).*phi.*WW.^4.* ...

YY+(-2).*phi.^2.*WW.^4.*YY+3.*WW.^2.*XX.*YY+(-5).*phi.*WW.^2.*XX.*YY+ ...

phi.^2.*WW.^2.*XX.*YY+3.*WW.^3.*XX.*YY+4.*phi.*WW.^3.*XX.*YY+(-2).* ...

phi.^2.*WW.^3.*XX.*YY+phi.*WW.^4.*XX.*YY+phi.^2.*WW.^4.*XX.*YY+phi.*WW.* ...

ZZ+(-1).*phi.^2.*WW.*ZZ+(-3).*WW.^2.*ZZ+4.*phi.*WW.^2.*ZZ+2.*phi.^2.* ...

WW.^2.*ZZ+(-3).*WW.^3.*ZZ+(-5).*phi.*WW.^3.*ZZ+(-1).*phi.^2.*WW.^3.*ZZ+ ...

2.*WW.*XX.*ZZ+(-6).*phi.*WW.*XX.*ZZ+2.*phi.^2.*WW.*XX.*ZZ+5.*WW.^2.*XX.* ...

ZZ+5.*phi.*WW.^2.*XX.*ZZ+(-5).*phi.^2.*WW.^2.*XX.*ZZ+(-1).*WW.^3.*XX.* ...

ZZ+2.*phi.*WW.^3.*XX.*ZZ+4.*phi.^2.*WW.^3.*XX.*ZZ+(-1).*phi.*WW.^4.*XX.* ...

ZZ+(-1).*phi.^2.*WW.^4.*XX.*ZZ+(-2).*phi.*WW.^2.*YY.*ZZ+phi.^2.*WW.^2.* ...

YY.*ZZ+3.*WW.^3.*YY.*ZZ+(-2).*phi.*WW.^3.*YY.*ZZ+(-2).*phi.^2.*WW.^3.* ...

YY.*ZZ+3.*WW.^4.*YY.*ZZ+4.*phi.*WW.^4.*YY.*ZZ+phi.^2.*WW.^4.*YY.*ZZ+2.* ...

phi.*WW.*XX.*YY.*ZZ+(-1).*phi.^2.*WW.*XX.*YY.*ZZ+(-3).*WW.^2.*XX.*YY.* ...

ZZ+2.*phi.*WW.^2.*XX.*YY.*ZZ+2.*phi.^2.*WW.^2.*XX.*YY.*ZZ+(-3).*WW.^3.* ...

XX.*YY.*ZZ+(-4).*phi.*WW.^3.*XX.*YY.*ZZ+(-1).*phi.^2.*WW.^3.*XX.*YY.*ZZ) ...

.*((-1).*WW+2.*phi.*WW+(-1).*phi.^2.*WW+(-3).*WW.^2+2.*phi.*WW.^2+ ...

phi.^2.*WW.^2+(-4).*phi.*WW.^3+phi.^2.*WW.^3+(-1).*phi.^2.*WW.^4+XX+(-2) ...

.*phi.*XX+phi.^2.*XX+3.*WW.*XX+(-2).*phi.*WW.*XX+(-1).*phi.^2.*WW.*XX+ ...

4.*phi.*WW.^2.*XX+(-1).*phi.^2.*WW.^2.*XX+phi.^2.*WW.^3.*XX+WW.^2.*YY+( ...

-4).*phi.*WW.^2.*YY+2.*phi.^2.*WW.^2.*YY+3.*WW.^3.*YY+2.*phi.*WW.^3.*YY+ ...

(-4).*phi.^2.*WW.^3.*YY+2.*phi.*WW.^4.*YY+2.*phi.^2.*WW.^4.*YY+(-1).* ...

WW.*XX.*YY+4.*phi.*WW.*XX.*YY+(-2).*phi.^2.*WW.*XX.*YY+(-3).*WW.^2.*XX.* ...

YY+(-2).*phi.*WW.^2.*XX.*YY+4.*phi.^2.*WW.^2.*XX.*YY+(-2).*phi.*WW.^3.* ...

XX.*YY+(-2).*phi.^2.*WW.^3.*XX.*YY+(-2).*phi.*WW.*ZZ+2.*phi.^2.*WW.*ZZ+ ...

3.*WW.^2.*ZZ+(-2).*phi.*WW.^2.*ZZ+(-4).*phi.^2.*WW.^2.*ZZ+WW.^3.*ZZ+4.* ...

phi.*WW.^3.*ZZ+2.*phi.^2.*WW.^3.*ZZ+2.*phi.*XX.*ZZ+(-2).*phi.^2.*XX.*ZZ+ ...

(-3).*WW.*XX.*ZZ+2.*phi.*WW.*XX.*ZZ+4.*phi.^2.*WW.*XX.*ZZ+(-1).*WW.^2.* ...

XX.*ZZ+(-4).*phi.*WW.^2.*XX.*ZZ+(-2).*phi.^2.*WW.^2.*XX.*ZZ+(-1).* ...

phi.^2.*WW.*YY.*ZZ+4.*phi.*WW.^2.*YY.*ZZ+phi.^2.*WW.^2.*YY.*ZZ+(-3).* ...

WW.^3.*YY.*ZZ+(-2).*phi.*WW.^3.*YY.*ZZ+phi.^2.*WW.^3.*YY.*ZZ+(-1).* ...

WW.^4.*YY.*ZZ+(-2).*phi.*WW.^4.*YY.*ZZ+(-1).*phi.^2.*WW.^4.*YY.*ZZ+ ...

phi.^2.*XX.*YY.*ZZ+(-4).*phi.*WW.*XX.*YY.*ZZ+(-1).*phi.^2.*WW.*XX.*YY.* ...

ZZ+3.*WW.^2.*XX.*YY.*ZZ+2.*phi.*WW.^2.*XX.*YY.*ZZ+(-1).*phi.^2.*WW.^2.* ...

XX.*YY.*ZZ+WW.^3.*XX.*YY.*ZZ+2.*phi.*WW.^3.*XX.*YY.*ZZ+phi.^2.*WW.^3.* ...

XX.*YY.*ZZ)+27.*(WW.^4+(-1).*WW.^3.*XX+WW.^3.*YY+(-2).*WW.^4.*YY+WW.^4.* ...

XX.*YY+(-1).*WW.^3.*ZZ+2.*WW.^3.*XX.*ZZ+(-1).*WW.^4.*XX.*ZZ+WW.^4.*YY.* ...

ZZ+(-1).*WW.^3.*XX.*YY.*ZZ).*((-1).*WW+2.*phi.*WW+(-1).*phi.^2.*WW+(-3) ...

.*WW.^2+2.*phi.*WW.^2+phi.^2.*WW.^2+(-4).*phi.*WW.^3+phi.^2.*WW.^3+(-1) ...

.*phi.^2.*WW.^4+XX+(-2).*phi.*XX+phi.^2.*XX+3.*WW.*XX+(-2).*phi.*WW.*XX+ ...

(-1).*phi.^2.*WW.*XX+4.*phi.*WW.^2.*XX+(-1).*phi.^2.*WW.^2.*XX+phi.^2.* ...

WW.^3.*XX+WW.^2.*YY+(-4).*phi.*WW.^2.*YY+2.*phi.^2.*WW.^2.*YY+3.*WW.^3.* ...

YY+2.*phi.*WW.^3.*YY+(-4).*phi.^2.*WW.^3.*YY+2.*phi.*WW.^4.*YY+2.* ...

phi.^2.*WW.^4.*YY+(-1).*WW.*XX.*YY+4.*phi.*WW.*XX.*YY+(-2).*phi.^2.*WW.* ...

XX.*YY+(-3).*WW.^2.*XX.*YY+(-2).*phi.*WW.^2.*XX.*YY+4.*phi.^2.*WW.^2.* ...

XX.*YY+(-2).*phi.*WW.^3.*XX.*YY+(-2).*phi.^2.*WW.^3.*XX.*YY+(-2).*phi.* ...

WW.*ZZ+2.*phi.^2.*WW.*ZZ+3.*WW.^2.*ZZ+(-2).*phi.*WW.^2.*ZZ+(-4).* ...

phi.^2.*WW.^2.*ZZ+WW.^3.*ZZ+4.*phi.*WW.^3.*ZZ+2.*phi.^2.*WW.^3.*ZZ+2.* ...

phi.*XX.*ZZ+(-2).*phi.^2.*XX.*ZZ+(-3).*WW.*XX.*ZZ+2.*phi.*WW.*XX.*ZZ+4.* ...

phi.^2.*WW.*XX.*ZZ+(-1).*WW.^2.*XX.*ZZ+(-4).*phi.*WW.^2.*XX.*ZZ+(-2).* ...

phi.^2.*WW.^2.*XX.*ZZ+(-1).*phi.^2.*WW.*YY.*ZZ+4.*phi.*WW.^2.*YY.*ZZ+ ...

phi.^2.*WW.^2.*YY.*ZZ+(-3).*WW.^3.*YY.*ZZ+(-2).*phi.*WW.^3.*YY.*ZZ+ ...

phi.^2.*WW.^3.*YY.*ZZ+(-1).*WW.^4.*YY.*ZZ+(-2).*phi.*WW.^4.*YY.*ZZ+(-1) ...

.*phi.^2.*WW.^4.*YY.*ZZ+phi.^2.*XX.*YY.*ZZ+(-4).*phi.*WW.*XX.*YY.*ZZ+( ...

-1).*phi.^2.*WW.*XX.*YY.*ZZ+3.*WW.^2.*XX.*YY.*ZZ+2.*phi.*WW.^2.*XX.*YY.* ...

ZZ+(-1).*phi.^2.*WW.^2.*XX.*YY.*ZZ+WW.^3.*XX.*YY.*ZZ+2.*phi.*WW.^3.*XX.* ...

YY.*ZZ+phi.^2.*WW.^3.*XX.*YY.*ZZ).^2).^2).^(1/2)).^(1/3)+(-1/4).*((-1).* ...

((-1)+phi+(-1).*phi.*WW+(-1).*phi.*YY+WW.*YY+phi.*WW.*YY).^(-3).*((-1)+ ...

phi+(-1).*phi.*WW+(-1).*phi.*ZZ+WW.*ZZ+phi.*WW.*ZZ).^(-3).*((-1)+2.*phi+ ...

(-1).*phi.^2+(-3).*WW+2.*phi.*WW+phi.^2.*WW+(-4).*phi.*WW.^2+phi.^2.* ...

WW.^2+(-1).*phi.^2.*WW.^3+WW.*YY+(-4).*phi.*WW.*YY+2.*phi.^2.*WW.*YY+3.* ...

WW.^2.*YY+2.*phi.*WW.^2.*YY+(-4).*phi.^2.*WW.^2.*YY+2.*phi.*WW.^3.*YY+ ...

2.*phi.^2.*WW.^3.*YY+(-2).*phi.*ZZ+2.*phi.^2.*ZZ+3.*WW.*ZZ+(-2).*phi.* ...

WW.*ZZ+(-4).*phi.^2.*WW.*ZZ+WW.^2.*ZZ+4.*phi.*WW.^2.*ZZ+2.*phi.^2.* ...

WW.^2.*ZZ+(-1).*phi.^2.*YY.*ZZ+4.*phi.*WW.*YY.*ZZ+phi.^2.*WW.*YY.*ZZ+( ...

-3).*WW.^2.*YY.*ZZ+(-2).*phi.*WW.^2.*YY.*ZZ+phi.^2.*WW.^2.*YY.*ZZ+(-1).* ...

WW.^3.*YY.*ZZ+(-2).*phi.*WW.^3.*YY.*ZZ+(-1).*phi.^2.*WW.^3.*YY.*ZZ).^3+( ...

-8).*(WW+(-1).*XX).^(-1).*((-1)+phi+(-1).*phi.*WW+(-1).*phi.*YY+WW.*YY+ ...

phi.*WW.*YY).^(-1).*((-1)+phi+(-1).*phi.*WW+(-1).*phi.*ZZ+WW.*ZZ+phi.* ...

WW.*ZZ).^(-1).*((-3).*WW.^3+2.*phi.*WW.^3+(-1).*WW.^4+(-2).*phi.*WW.^4+ ...

3.*WW.^2.*XX+(-2).*phi.*WW.^2.*XX+WW.^3.*XX+2.*phi.*WW.^3.*XX+(-1).* ...

WW.^2.*YY+2.*phi.*WW.^2.*YY+WW.^3.*YY+(-6).*phi.*WW.^3.*YY+4.*WW.^4.*YY+ ...

4.*phi.*WW.^4.*YY+(-3).*WW.^3.*XX.*YY+2.*phi.*WW.^3.*XX.*YY+(-1).* ...

WW.^4.*XX.*YY+(-2).*phi.*WW.^4.*XX.*YY+WW.^2.*ZZ+(-2).*phi.*WW.^2.*ZZ+ ...

3.*WW.^3.*ZZ+2.*phi.*WW.^3.*ZZ+(-4).*WW.^2.*XX.*ZZ+4.*phi.*WW.^2.*XX.* ...

ZZ+(-1).*WW.^3.*XX.*ZZ+(-6).*phi.*WW.^3.*XX.*ZZ+WW.^4.*XX.*ZZ+2.*phi.* ...

WW.^4.*XX.*ZZ+(-1).*WW.^3.*YY.*ZZ+2.*phi.*WW.^3.*YY.*ZZ+(-3).*WW.^4.* ...

YY.*ZZ+(-2).*phi.*WW.^4.*YY.*ZZ+WW.^2.*XX.*YY.*ZZ+(-2).*phi.*WW.^2.*XX.* ...

YY.*ZZ+3.*WW.^3.*XX.*YY.*ZZ+2.*phi.*WW.^3.*XX.*YY.*ZZ)+4.*(WW+(-1).*XX) ...

.^(-1).*((-1)+phi+(-1).*phi.*WW+(-1).*phi.*YY+WW.*YY+phi.*WW.*YY).^(-2) ...

.*((-1)+phi+(-1).*phi.*WW+(-1).*phi.*ZZ+WW.*ZZ+phi.*WW.*ZZ).^(-2).*((-1) ...

+2.*phi+(-1).*phi.^2+(-3).*WW+2.*phi.*WW+phi.^2.*WW+(-4).*phi.*WW.^2+ ...

phi.^2.*WW.^2+(-1).*phi.^2.*WW.^3+WW.*YY+(-4).*phi.*WW.*YY+2.*phi.^2.* ...

WW.*YY+3.*WW.^2.*YY+2.*phi.*WW.^2.*YY+(-4).*phi.^2.*WW.^2.*YY+2.*phi.* ...

WW.^3.*YY+2.*phi.^2.*WW.^3.*YY+(-2).*phi.*ZZ+2.*phi.^2.*ZZ+3.*WW.*ZZ+( ...

-2).*phi.*WW.*ZZ+(-4).*phi.^2.*WW.*ZZ+WW.^2.*ZZ+4.*phi.*WW.^2.*ZZ+2.* ...

phi.^2.*WW.^2.*ZZ+(-1).*phi.^2.*YY.*ZZ+4.*phi.*WW.*YY.*ZZ+phi.^2.*WW.* ...

YY.*ZZ+(-3).*WW.^2.*YY.*ZZ+(-2).*phi.*WW.^2.*YY.*ZZ+phi.^2.*WW.^2.*YY.* ...

ZZ+(-1).*WW.^3.*YY.*ZZ+(-2).*phi.*WW.^3.*YY.*ZZ+(-1).*phi.^2.*WW.^3.* ...

YY.*ZZ).*(3.*WW.^2+(-4).*phi.*WW.^2+phi.^2.*WW.^2+3.*WW.^3+2.*phi.* ...

WW.^3+(-2).*phi.^2.*WW.^3+2.*phi.*WW.^4+phi.^2.*WW.^4+(-3).*WW.*XX+4.* ...

phi.*WW.*XX+(-1).*phi.^2.*WW.*XX+(-3).*WW.^2.*XX+(-2).*phi.*WW.^2.*XX+ ...

2.*phi.^2.*WW.^2.*XX+(-2).*phi.*WW.^3.*XX+(-1).*phi.^2.*WW.^3.*XX+(-1).* ...

phi.*WW.*YY+phi.^2.*WW.*YY+WW.^2.*YY+2.*phi.*WW.^2.*YY+(-4).*phi.^2.* ...

WW.^2.*YY+(-5).*WW.^3.*YY+5.*phi.*WW.^3.*YY+5.*phi.^2.*WW.^3.*YY+(-2).* ...

WW.^4.*YY+(-6).*phi.*WW.^4.*YY+(-2).*phi.^2.*WW.^4.*YY+3.*WW.^2.*XX.*YY+ ...

(-5).*phi.*WW.^2.*XX.*YY+phi.^2.*WW.^2.*XX.*YY+3.*WW.^3.*XX.*YY+4.*phi.* ...

WW.^3.*XX.*YY+(-2).*phi.^2.*WW.^3.*XX.*YY+phi.*WW.^4.*XX.*YY+phi.^2.* ...

WW.^4.*XX.*YY+phi.*WW.*ZZ+(-1).*phi.^2.*WW.*ZZ+(-3).*WW.^2.*ZZ+4.*phi.* ...

WW.^2.*ZZ+2.*phi.^2.*WW.^2.*ZZ+(-3).*WW.^3.*ZZ+(-5).*phi.*WW.^3.*ZZ+(-1) ...

.*phi.^2.*WW.^3.*ZZ+2.*WW.*XX.*ZZ+(-6).*phi.*WW.*XX.*ZZ+2.*phi.^2.*WW.* ...

XX.*ZZ+5.*WW.^2.*XX.*ZZ+5.*phi.*WW.^2.*XX.*ZZ+(-5).*phi.^2.*WW.^2.*XX.* ...

ZZ+(-1).*WW.^3.*XX.*ZZ+2.*phi.*WW.^3.*XX.*ZZ+4.*phi.^2.*WW.^3.*XX.*ZZ+( ...

-1).*phi.*WW.^4.*XX.*ZZ+(-1).*phi.^2.*WW.^4.*XX.*ZZ+(-2).*phi.*WW.^2.* ...

YY.*ZZ+phi.^2.*WW.^2.*YY.*ZZ+3.*WW.^3.*YY.*ZZ+(-2).*phi.*WW.^3.*YY.*ZZ+( ...

-2).*phi.^2.*WW.^3.*YY.*ZZ+3.*WW.^4.*YY.*ZZ+4.*phi.*WW.^4.*YY.*ZZ+ ...

phi.^2.*WW.^4.*YY.*ZZ+2.*phi.*WW.*XX.*YY.*ZZ+(-1).*phi.^2.*WW.*XX.*YY.* ...

ZZ+(-3).*WW.^2.*XX.*YY.*ZZ+2.*phi.*WW.^2.*XX.*YY.*ZZ+2.*phi.^2.*WW.^2.* ...

XX.*YY.*ZZ+(-3).*WW.^3.*XX.*YY.*ZZ+(-4).*phi.*WW.^3.*XX.*YY.*ZZ+(-1).* ...

phi.^2.*WW.^3.*XX.*YY.*ZZ)).*((1/4).*((-1)+phi+(-1).*phi.*WW+(-1).*phi.* ...

YY+WW.*YY+phi.*WW.*YY).^(-2).*((-1)+phi+(-1).*phi.*WW+(-1).*phi.*ZZ+WW.* ...

ZZ+phi.*WW.*ZZ).^(-2).*((-1)+2.*phi+(-1).*phi.^2+(-3).*WW+2.*phi.*WW+ ...

phi.^2.*WW+(-4).*phi.*WW.^2+phi.^2.*WW.^2+(-1).*phi.^2.*WW.^3+WW.*YY+( ...

-4).*phi.*WW.*YY+2.*phi.^2.*WW.*YY+3.*WW.^2.*YY+2.*phi.*WW.^2.*YY+(-4).* ...

phi.^2.*WW.^2.*YY+2.*phi.*WW.^3.*YY+2.*phi.^2.*WW.^3.*YY+(-2).*phi.*ZZ+ ...

2.*phi.^2.*ZZ+3.*WW.*ZZ+(-2).*phi.*WW.*ZZ+(-4).*phi.^2.*WW.*ZZ+WW.^2.* ...

ZZ+4.*phi.*WW.^2.*ZZ+2.*phi.^2.*WW.^2.*ZZ+(-1).*phi.^2.*YY.*ZZ+4.*phi.* ...

WW.*YY.*ZZ+phi.^2.*WW.*YY.*ZZ+(-3).*WW.^2.*YY.*ZZ+(-2).*phi.*WW.^2.*YY.* ...

ZZ+phi.^2.*WW.^2.*YY.*ZZ+(-1).*WW.^3.*YY.*ZZ+(-2).*phi.*WW.^3.*YY.*ZZ+( ...

-1).*phi.^2.*WW.^3.*YY.*ZZ).^2+(-1).*(WW+(-1).*XX).^(-1).*((-1)+phi+(-1) ...

.*phi.*WW+(-1).*phi.*YY+WW.*YY+phi.*WW.*YY).^(-1).*((-1)+phi+(-1).*phi.* ...

WW+(-1).*phi.*ZZ+WW.*ZZ+phi.*WW.*ZZ).^(-1).*(3.*WW.^2+(-4).*phi.*WW.^2+ ...

phi.^2.*WW.^2+3.*WW.^3+2.*phi.*WW.^3+(-2).*phi.^2.*WW.^3+2.*phi.*WW.^4+ ...

phi.^2.*WW.^4+(-3).*WW.*XX+4.*phi.*WW.*XX+(-1).*phi.^2.*WW.*XX+(-3).* ...

WW.^2.*XX+(-2).*phi.*WW.^2.*XX+2.*phi.^2.*WW.^2.*XX+(-2).*phi.*WW.^3.* ...

XX+(-1).*phi.^2.*WW.^3.*XX+(-1).*phi.*WW.*YY+phi.^2.*WW.*YY+WW.^2.*YY+ ...

2.*phi.*WW.^2.*YY+(-4).*phi.^2.*WW.^2.*YY+(-5).*WW.^3.*YY+5.*phi.* ...

WW.^3.*YY+5.*phi.^2.*WW.^3.*YY+(-2).*WW.^4.*YY+(-6).*phi.*WW.^4.*YY+(-2) ...

.*phi.^2.*WW.^4.*YY+3.*WW.^2.*XX.*YY+(-5).*phi.*WW.^2.*XX.*YY+phi.^2.* ...

WW.^2.*XX.*YY+3.*WW.^3.*XX.*YY+4.*phi.*WW.^3.*XX.*YY+(-2).*phi.^2.* ...

WW.^3.*XX.*YY+phi.*WW.^4.*XX.*YY+phi.^2.*WW.^4.*XX.*YY+phi.*WW.*ZZ+(-1) ...

.*phi.^2.*WW.*ZZ+(-3).*WW.^2.*ZZ+4.*phi.*WW.^2.*ZZ+2.*phi.^2.*WW.^2.*ZZ+ ...

(-3).*WW.^3.*ZZ+(-5).*phi.*WW.^3.*ZZ+(-1).*phi.^2.*WW.^3.*ZZ+2.*WW.*XX.* ...

ZZ+(-6).*phi.*WW.*XX.*ZZ+2.*phi.^2.*WW.*XX.*ZZ+5.*WW.^2.*XX.*ZZ+5.*phi.* ...

WW.^2.*XX.*ZZ+(-5).*phi.^2.*WW.^2.*XX.*ZZ+(-1).*WW.^3.*XX.*ZZ+2.*phi.* ...

WW.^3.*XX.*ZZ+4.*phi.^2.*WW.^3.*XX.*ZZ+(-1).*phi.*WW.^4.*XX.*ZZ+(-1).* ...

phi.^2.*WW.^4.*XX.*ZZ+(-2).*phi.*WW.^2.*YY.*ZZ+phi.^2.*WW.^2.*YY.*ZZ+3.* ...

WW.^3.*YY.*ZZ+(-2).*phi.*WW.^3.*YY.*ZZ+(-2).*phi.^2.*WW.^3.*YY.*ZZ+3.* ...

WW.^4.*YY.*ZZ+4.*phi.*WW.^4.*YY.*ZZ+phi.^2.*WW.^4.*YY.*ZZ+2.*phi.*WW.* ...

XX.*YY.*ZZ+(-1).*phi.^2.*WW.*XX.*YY.*ZZ+(-3).*WW.^2.*XX.*YY.*ZZ+2.*phi.* ...

WW.^2.*XX.*YY.*ZZ+2.*phi.^2.*WW.^2.*XX.*YY.*ZZ+(-3).*WW.^3.*XX.*YY.*ZZ+( ...

-4).*phi.*WW.^3.*XX.*YY.*ZZ+(-1).*phi.^2.*WW.^3.*XX.*YY.*ZZ)+(1/3).*(WW+ ...

(-2).*phi.*WW+phi.^2.*WW+2.*phi.*WW.^2+(-2).*phi.^2.*WW.^2+phi.^2.* ...

WW.^3+(-1).*XX+2.*phi.*XX+(-1).*phi.^2.*XX+(-2).*phi.*WW.*XX+2.*phi.^2.* ...

WW.*XX+(-1).*phi.^2.*WW.^2.*XX+phi.*WW.*YY+(-1).*phi.^2.*WW.*YY+(-1).* ...

WW.^2.*YY+2.*phi.^2.*WW.^2.*YY+(-1).*phi.*WW.^3.*YY+(-1).*phi.^2.* ...

WW.^3.*YY+(-1).*phi.*XX.*YY+phi.^2.*XX.*YY+WW.*XX.*YY+(-2).*phi.^2.*WW.* ...

XX.*YY+phi.*WW.^2.*XX.*YY+phi.^2.*WW.^2.*XX.*YY+phi.*WW.*ZZ+(-1).* ...

phi.^2.*WW.*ZZ+(-1).*WW.^2.*ZZ+2.*phi.^2.*WW.^2.*ZZ+(-1).*phi.*WW.^3.* ...

ZZ+(-1).*phi.^2.*WW.^3.*ZZ+(-1).*phi.*XX.*ZZ+phi.^2.*XX.*ZZ+WW.*XX.*ZZ+( ...

-2).*phi.^2.*WW.*XX.*ZZ+phi.*WW.^2.*XX.*ZZ+phi.^2.*WW.^2.*XX.*ZZ+ ...

phi.^2.*WW.*YY.*ZZ+(-2).*phi.*WW.^2.*YY.*ZZ+(-2).*phi.^2.*WW.^2.*YY.*ZZ+ ...

WW.^3.*YY.*ZZ+2.*phi.*WW.^3.*YY.*ZZ+phi.^2.*WW.^3.*YY.*ZZ+(-1).*phi.^2.* ...

XX.*YY.*ZZ+2.*phi.*WW.*XX.*YY.*ZZ+2.*phi.^2.*WW.*XX.*YY.*ZZ+(-1).* ...

WW.^2.*XX.*YY.*ZZ+(-2).*phi.*WW.^2.*XX.*YY.*ZZ+(-1).*phi.^2.*WW.^2.*XX.* ...

YY.*ZZ).^(-1).*(3.*WW.^2+(-4).*phi.*WW.^2+phi.^2.*WW.^2+3.*WW.^3+2.* ...

phi.*WW.^3+(-2).*phi.^2.*WW.^3+2.*phi.*WW.^4+phi.^2.*WW.^4+(-3).*WW.*XX+ ...

4.*phi.*WW.*XX+(-1).*phi.^2.*WW.*XX+(-3).*WW.^2.*XX+(-2).*phi.*WW.^2.* ...

XX+2.*phi.^2.*WW.^2.*XX+(-2).*phi.*WW.^3.*XX+(-1).*phi.^2.*WW.^3.*XX+( ...

-1).*phi.*WW.*YY+phi.^2.*WW.*YY+WW.^2.*YY+2.*phi.*WW.^2.*YY+(-4).* ...

phi.^2.*WW.^2.*YY+(-5).*WW.^3.*YY+5.*phi.*WW.^3.*YY+5.*phi.^2.*WW.^3.* ...

YY+(-2).*WW.^4.*YY+(-6).*phi.*WW.^4.*YY+(-2).*phi.^2.*WW.^4.*YY+3.* ...

WW.^2.*XX.*YY+(-5).*phi.*WW.^2.*XX.*YY+phi.^2.*WW.^2.*XX.*YY+3.*WW.^3.* ...

XX.*YY+4.*phi.*WW.^3.*XX.*YY+(-2).*phi.^2.*WW.^3.*XX.*YY+phi.*WW.^4.* ...

XX.*YY+phi.^2.*WW.^4.*XX.*YY+phi.*WW.*ZZ+(-1).*phi.^2.*WW.*ZZ+(-3).* ...

WW.^2.*ZZ+4.*phi.*WW.^2.*ZZ+2.*phi.^2.*WW.^2.*ZZ+(-3).*WW.^3.*ZZ+(-5).* ...

phi.*WW.^3.*ZZ+(-1).*phi.^2.*WW.^3.*ZZ+2.*WW.*XX.*ZZ+(-6).*phi.*WW.*XX.* ...

ZZ+2.*phi.^2.*WW.*XX.*ZZ+5.*WW.^2.*XX.*ZZ+5.*phi.*WW.^2.*XX.*ZZ+(-5).* ...

phi.^2.*WW.^2.*XX.*ZZ+(-1).*WW.^3.*XX.*ZZ+2.*phi.*WW.^3.*XX.*ZZ+4.* ...

phi.^2.*WW.^3.*XX.*ZZ+(-1).*phi.*WW.^4.*XX.*ZZ+(-1).*phi.^2.*WW.^4.*XX.* ...

ZZ+(-2).*phi.*WW.^2.*YY.*ZZ+phi.^2.*WW.^2.*YY.*ZZ+3.*WW.^3.*YY.*ZZ+(-2) ...

.*phi.*WW.^3.*YY.*ZZ+(-2).*phi.^2.*WW.^3.*YY.*ZZ+3.*WW.^4.*YY.*ZZ+4.* ...

phi.*WW.^4.*YY.*ZZ+phi.^2.*WW.^4.*YY.*ZZ+2.*phi.*WW.*XX.*YY.*ZZ+(-1).* ...

phi.^2.*WW.*XX.*YY.*ZZ+(-3).*WW.^2.*XX.*YY.*ZZ+2.*phi.*WW.^2.*XX.*YY.* ...

ZZ+2.*phi.^2.*WW.^2.*XX.*YY.*ZZ+(-3).*WW.^3.*XX.*YY.*ZZ+(-4).*phi.* ...

WW.^3.*XX.*YY.*ZZ+(-1).*phi.^2.*WW.^3.*XX.*YY.*ZZ)+(1/3).*2.^(1/3).*(WW+ ...

(-1).*XX).^(-1).*((-1)+phi+(-1).*phi.*WW+(-1).*phi.*YY+WW.*YY+phi.*WW.* ...

YY).^(-1).*((-1)+phi+(-1).*phi.*WW+(-1).*phi.*ZZ+WW.*ZZ+phi.*WW.*ZZ).^( ...

-1).*(12.*(WW+(-2).*phi.*WW+phi.^2.*WW+2.*phi.*WW.^2+(-2).*phi.^2.* ...

WW.^2+phi.^2.*WW.^3+(-1).*XX+2.*phi.*XX+(-1).*phi.^2.*XX+(-2).*phi.*WW.* ...

XX+2.*phi.^2.*WW.*XX+(-1).*phi.^2.*WW.^2.*XX+phi.*WW.*YY+(-1).*phi.^2.* ...

WW.*YY+(-1).*WW.^2.*YY+2.*phi.^2.*WW.^2.*YY+(-1).*phi.*WW.^3.*YY+(-1).* ...

phi.^2.*WW.^3.*YY+(-1).*phi.*XX.*YY+phi.^2.*XX.*YY+WW.*XX.*YY+(-2).* ...

phi.^2.*WW.*XX.*YY+phi.*WW.^2.*XX.*YY+phi.^2.*WW.^2.*XX.*YY+phi.*WW.*ZZ+ ...

(-1).*phi.^2.*WW.*ZZ+(-1).*WW.^2.*ZZ+2.*phi.^2.*WW.^2.*ZZ+(-1).*phi.* ...

WW.^3.*ZZ+(-1).*phi.^2.*WW.^3.*ZZ+(-1).*phi.*XX.*ZZ+phi.^2.*XX.*ZZ+WW.* ...

XX.*ZZ+(-2).*phi.^2.*WW.*XX.*ZZ+phi.*WW.^2.*XX.*ZZ+phi.^2.*WW.^2.*XX.* ...

ZZ+phi.^2.*WW.*YY.*ZZ+(-2).*phi.*WW.^2.*YY.*ZZ+(-2).*phi.^2.*WW.^2.*YY.* ...

ZZ+WW.^3.*YY.*ZZ+2.*phi.*WW.^3.*YY.*ZZ+phi.^2.*WW.^3.*YY.*ZZ+(-1).* ...

phi.^2.*XX.*YY.*ZZ+2.*phi.*WW.*XX.*YY.*ZZ+2.*phi.^2.*WW.*XX.*YY.*ZZ+(-1) ...

.*WW.^2.*XX.*YY.*ZZ+(-2).*phi.*WW.^2.*XX.*YY.*ZZ+(-1).*phi.^2.*WW.^2.* ...

XX.*YY.*ZZ).*(WW.^4+(-1).*WW.^3.*XX+WW.^3.*YY+(-2).*WW.^4.*YY+WW.^4.* ...

XX.*YY+(-1).*WW.^3.*ZZ+2.*WW.^3.*XX.*ZZ+(-1).*WW.^4.*XX.*ZZ+WW.^4.*YY.* ...

ZZ+(-1).*WW.^3.*XX.*YY.*ZZ)+(3.*WW.^2+(-4).*phi.*WW.^2+phi.^2.*WW.^2+3.* ...

WW.^3+2.*phi.*WW.^3+(-2).*phi.^2.*WW.^3+2.*phi.*WW.^4+phi.^2.*WW.^4+(-3) ...

.*WW.*XX+4.*phi.*WW.*XX+(-1).*phi.^2.*WW.*XX+(-3).*WW.^2.*XX+(-2).*phi.* ...

WW.^2.*XX+2.*phi.^2.*WW.^2.*XX+(-2).*phi.*WW.^3.*XX+(-1).*phi.^2.* ...

WW.^3.*XX+(-1).*phi.*WW.*YY+phi.^2.*WW.*YY+WW.^2.*YY+2.*phi.*WW.^2.*YY+( ...

-4).*phi.^2.*WW.^2.*YY+(-5).*WW.^3.*YY+5.*phi.*WW.^3.*YY+5.*phi.^2.* ...

WW.^3.*YY+(-2).*WW.^4.*YY+(-6).*phi.*WW.^4.*YY+(-2).*phi.^2.*WW.^4.*YY+ ...

3.*WW.^2.*XX.*YY+(-5).*phi.*WW.^2.*XX.*YY+phi.^2.*WW.^2.*XX.*YY+3.* ...

WW.^3.*XX.*YY+4.*phi.*WW.^3.*XX.*YY+(-2).*phi.^2.*WW.^3.*XX.*YY+phi.* ...

WW.^4.*XX.*YY+phi.^2.*WW.^4.*XX.*YY+phi.*WW.*ZZ+(-1).*phi.^2.*WW.*ZZ+( ...

-3).*WW.^2.*ZZ+4.*phi.*WW.^2.*ZZ+2.*phi.^2.*WW.^2.*ZZ+(-3).*WW.^3.*ZZ+( ...

-5).*phi.*WW.^3.*ZZ+(-1).*phi.^2.*WW.^3.*ZZ+2.*WW.*XX.*ZZ+(-6).*phi.* ...

WW.*XX.*ZZ+2.*phi.^2.*WW.*XX.*ZZ+5.*WW.^2.*XX.*ZZ+5.*phi.*WW.^2.*XX.*ZZ+ ...

(-5).*phi.^2.*WW.^2.*XX.*ZZ+(-1).*WW.^3.*XX.*ZZ+2.*phi.*WW.^3.*XX.*ZZ+ ...

4.*phi.^2.*WW.^3.*XX.*ZZ+(-1).*phi.*WW.^4.*XX.*ZZ+(-1).*phi.^2.*WW.^4.* ...

XX.*ZZ+(-2).*phi.*WW.^2.*YY.*ZZ+phi.^2.*WW.^2.*YY.*ZZ+3.*WW.^3.*YY.*ZZ+( ...

-2).*phi.*WW.^3.*YY.*ZZ+(-2).*phi.^2.*WW.^3.*YY.*ZZ+3.*WW.^4.*YY.*ZZ+4.* ...

phi.*WW.^4.*YY.*ZZ+phi.^2.*WW.^4.*YY.*ZZ+2.*phi.*WW.*XX.*YY.*ZZ+(-1).* ...

phi.^2.*WW.*XX.*YY.*ZZ+(-3).*WW.^2.*XX.*YY.*ZZ+2.*phi.*WW.^2.*XX.*YY.* ...

ZZ+2.*phi.^2.*WW.^2.*XX.*YY.*ZZ+(-3).*WW.^3.*XX.*YY.*ZZ+(-4).*phi.* ...

WW.^3.*XX.*YY.*ZZ+(-1).*phi.^2.*WW.^3.*XX.*YY.*ZZ).^2+(-3).*((-3).* ...

WW.^3+2.*phi.*WW.^3+(-1).*WW.^4+(-2).*phi.*WW.^4+3.*WW.^2.*XX+(-2).* ...

phi.*WW.^2.*XX+WW.^3.*XX+2.*phi.*WW.^3.*XX+(-1).*WW.^2.*YY+2.*phi.* ...

WW.^2.*YY+WW.^3.*YY+(-6).*phi.*WW.^3.*YY+4.*WW.^4.*YY+4.*phi.*WW.^4.*YY+ ...

(-3).*WW.^3.*XX.*YY+2.*phi.*WW.^3.*XX.*YY+(-1).*WW.^4.*XX.*YY+(-2).* ...

phi.*WW.^4.*XX.*YY+WW.^2.*ZZ+(-2).*phi.*WW.^2.*ZZ+3.*WW.^3.*ZZ+2.*phi.* ...

WW.^3.*ZZ+(-4).*WW.^2.*XX.*ZZ+4.*phi.*WW.^2.*XX.*ZZ+(-1).*WW.^3.*XX.*ZZ+ ...

(-6).*phi.*WW.^3.*XX.*ZZ+WW.^4.*XX.*ZZ+2.*phi.*WW.^4.*XX.*ZZ+(-1).* ...

WW.^3.*YY.*ZZ+2.*phi.*WW.^3.*YY.*ZZ+(-3).*WW.^4.*YY.*ZZ+(-2).*phi.* ...

WW.^4.*YY.*ZZ+WW.^2.*XX.*YY.*ZZ+(-2).*phi.*WW.^2.*XX.*YY.*ZZ+3.*WW.^3.* ...

XX.*YY.*ZZ+2.*phi.*WW.^3.*XX.*YY.*ZZ).*((-1).*WW+2.*phi.*WW+(-1).* ...

phi.^2.*WW+(-3).*WW.^2+2.*phi.*WW.^2+phi.^2.*WW.^2+(-4).*phi.*WW.^3+ ...

phi.^2.*WW.^3+(-1).*phi.^2.*WW.^4+XX+(-2).*phi.*XX+phi.^2.*XX+3.*WW.*XX+ ...

(-2).*phi.*WW.*XX+(-1).*phi.^2.*WW.*XX+4.*phi.*WW.^2.*XX+(-1).*phi.^2.* ...

WW.^2.*XX+phi.^2.*WW.^3.*XX+WW.^2.*YY+(-4).*phi.*WW.^2.*YY+2.*phi.^2.* ...

WW.^2.*YY+3.*WW.^3.*YY+2.*phi.*WW.^3.*YY+(-4).*phi.^2.*WW.^3.*YY+2.* ...

phi.*WW.^4.*YY+2.*phi.^2.*WW.^4.*YY+(-1).*WW.*XX.*YY+4.*phi.*WW.*XX.*YY+ ...

(-2).*phi.^2.*WW.*XX.*YY+(-3).*WW.^2.*XX.*YY+(-2).*phi.*WW.^2.*XX.*YY+ ...

4.*phi.^2.*WW.^2.*XX.*YY+(-2).*phi.*WW.^3.*XX.*YY+(-2).*phi.^2.*WW.^3.* ...

XX.*YY+(-2).*phi.*WW.*ZZ+2.*phi.^2.*WW.*ZZ+3.*WW.^2.*ZZ+(-2).*phi.* ...

WW.^2.*ZZ+(-4).*phi.^2.*WW.^2.*ZZ+WW.^3.*ZZ+4.*phi.*WW.^3.*ZZ+2.* ...

phi.^2.*WW.^3.*ZZ+2.*phi.*XX.*ZZ+(-2).*phi.^2.*XX.*ZZ+(-3).*WW.*XX.*ZZ+ ...

2.*phi.*WW.*XX.*ZZ+4.*phi.^2.*WW.*XX.*ZZ+(-1).*WW.^2.*XX.*ZZ+(-4).*phi.* ...

WW.^2.*XX.*ZZ+(-2).*phi.^2.*WW.^2.*XX.*ZZ+(-1).*phi.^2.*WW.*YY.*ZZ+4.* ...

phi.*WW.^2.*YY.*ZZ+phi.^2.*WW.^2.*YY.*ZZ+(-3).*WW.^3.*YY.*ZZ+(-2).*phi.* ...

WW.^3.*YY.*ZZ+phi.^2.*WW.^3.*YY.*ZZ+(-1).*WW.^4.*YY.*ZZ+(-2).*phi.* ...

WW.^4.*YY.*ZZ+(-1).*phi.^2.*WW.^4.*YY.*ZZ+phi.^2.*XX.*YY.*ZZ+(-4).*phi.* ...

WW.*XX.*YY.*ZZ+(-1).*phi.^2.*WW.*XX.*YY.*ZZ+3.*WW.^2.*XX.*YY.*ZZ+2.* ...

phi.*WW.^2.*XX.*YY.*ZZ+(-1).*phi.^2.*WW.^2.*XX.*YY.*ZZ+WW.^3.*XX.*YY.* ...

ZZ+2.*phi.*WW.^3.*XX.*YY.*ZZ+phi.^2.*WW.^3.*XX.*YY.*ZZ)).*(27.*(WW+(-2) ...

.*phi.*WW+phi.^2.*WW+2.*phi.*WW.^2+(-2).*phi.^2.*WW.^2+phi.^2.*WW.^3+( ...

-1).*XX+2.*phi.*XX+(-1).*phi.^2.*XX+(-2).*phi.*WW.*XX+2.*phi.^2.*WW.*XX+ ...

(-1).*phi.^2.*WW.^2.*XX+phi.*WW.*YY+(-1).*phi.^2.*WW.*YY+(-1).*WW.^2.* ...

YY+2.*phi.^2.*WW.^2.*YY+(-1).*phi.*WW.^3.*YY+(-1).*phi.^2.*WW.^3.*YY+( ...

-1).*phi.*XX.*YY+phi.^2.*XX.*YY+WW.*XX.*YY+(-2).*phi.^2.*WW.*XX.*YY+ ...

phi.*WW.^2.*XX.*YY+phi.^2.*WW.^2.*XX.*YY+phi.*WW.*ZZ+(-1).*phi.^2.*WW.* ...

ZZ+(-1).*WW.^2.*ZZ+2.*phi.^2.*WW.^2.*ZZ+(-1).*phi.*WW.^3.*ZZ+(-1).* ...

phi.^2.*WW.^3.*ZZ+(-1).*phi.*XX.*ZZ+phi.^2.*XX.*ZZ+WW.*XX.*ZZ+(-2).* ...

phi.^2.*WW.*XX.*ZZ+phi.*WW.^2.*XX.*ZZ+phi.^2.*WW.^2.*XX.*ZZ+phi.^2.*WW.* ...

YY.*ZZ+(-2).*phi.*WW.^2.*YY.*ZZ+(-2).*phi.^2.*WW.^2.*YY.*ZZ+WW.^3.*YY.* ...

ZZ+2.*phi.*WW.^3.*YY.*ZZ+phi.^2.*WW.^3.*YY.*ZZ+(-1).*phi.^2.*XX.*YY.*ZZ+ ...

2.*phi.*WW.*XX.*YY.*ZZ+2.*phi.^2.*WW.*XX.*YY.*ZZ+(-1).*WW.^2.*XX.*YY.* ...

ZZ+(-2).*phi.*WW.^2.*XX.*YY.*ZZ+(-1).*phi.^2.*WW.^2.*XX.*YY.*ZZ).*((-3) ...

.*WW.^3+2.*phi.*WW.^3+(-1).*WW.^4+(-2).*phi.*WW.^4+3.*WW.^2.*XX+(-2).* ...

phi.*WW.^2.*XX+WW.^3.*XX+2.*phi.*WW.^3.*XX+(-1).*WW.^2.*YY+2.*phi.* ...

WW.^2.*YY+WW.^3.*YY+(-6).*phi.*WW.^3.*YY+4.*WW.^4.*YY+4.*phi.*WW.^4.*YY+ ...

(-3).*WW.^3.*XX.*YY+2.*phi.*WW.^3.*XX.*YY+(-1).*WW.^4.*XX.*YY+(-2).* ...

phi.*WW.^4.*XX.*YY+WW.^2.*ZZ+(-2).*phi.*WW.^2.*ZZ+3.*WW.^3.*ZZ+2.*phi.* ...

WW.^3.*ZZ+(-4).*WW.^2.*XX.*ZZ+4.*phi.*WW.^2.*XX.*ZZ+(-1).*WW.^3.*XX.*ZZ+ ...

(-6).*phi.*WW.^3.*XX.*ZZ+WW.^4.*XX.*ZZ+2.*phi.*WW.^4.*XX.*ZZ+(-1).* ...

WW.^3.*YY.*ZZ+2.*phi.*WW.^3.*YY.*ZZ+(-3).*WW.^4.*YY.*ZZ+(-2).*phi.* ...

WW.^4.*YY.*ZZ+WW.^2.*XX.*YY.*ZZ+(-2).*phi.*WW.^2.*XX.*YY.*ZZ+3.*WW.^3.* ...

XX.*YY.*ZZ+2.*phi.*WW.^3.*XX.*YY.*ZZ).^2+(-72).*(WW+(-2).*phi.*WW+ ...

phi.^2.*WW+2.*phi.*WW.^2+(-2).*phi.^2.*WW.^2+phi.^2.*WW.^3+(-1).*XX+2.* ...

phi.*XX+(-1).*phi.^2.*XX+(-2).*phi.*WW.*XX+2.*phi.^2.*WW.*XX+(-1).* ...

phi.^2.*WW.^2.*XX+phi.*WW.*YY+(-1).*phi.^2.*WW.*YY+(-1).*WW.^2.*YY+2.* ...

phi.^2.*WW.^2.*YY+(-1).*phi.*WW.^3.*YY+(-1).*phi.^2.*WW.^3.*YY+(-1).* ...

phi.*XX.*YY+phi.^2.*XX.*YY+WW.*XX.*YY+(-2).*phi.^2.*WW.*XX.*YY+phi.* ...

WW.^2.*XX.*YY+phi.^2.*WW.^2.*XX.*YY+phi.*WW.*ZZ+(-1).*phi.^2.*WW.*ZZ+( ...

-1).*WW.^2.*ZZ+2.*phi.^2.*WW.^2.*ZZ+(-1).*phi.*WW.^3.*ZZ+(-1).*phi.^2.* ...

WW.^3.*ZZ+(-1).*phi.*XX.*ZZ+phi.^2.*XX.*ZZ+WW.*XX.*ZZ+(-2).*phi.^2.*WW.* ...

XX.*ZZ+phi.*WW.^2.*XX.*ZZ+phi.^2.*WW.^2.*XX.*ZZ+phi.^2.*WW.*YY.*ZZ+(-2) ...

.*phi.*WW.^2.*YY.*ZZ+(-2).*phi.^2.*WW.^2.*YY.*ZZ+WW.^3.*YY.*ZZ+2.*phi.* ...

WW.^3.*YY.*ZZ+phi.^2.*WW.^3.*YY.*ZZ+(-1).*phi.^2.*XX.*YY.*ZZ+2.*phi.* ...

WW.*XX.*YY.*ZZ+2.*phi.^2.*WW.*XX.*YY.*ZZ+(-1).*WW.^2.*XX.*YY.*ZZ+(-2).* ...

phi.*WW.^2.*XX.*YY.*ZZ+(-1).*phi.^2.*WW.^2.*XX.*YY.*ZZ).*(WW.^4+(-1).* ...

WW.^3.*XX+WW.^3.*YY+(-2).*WW.^4.*YY+WW.^4.*XX.*YY+(-1).*WW.^3.*ZZ+2.* ...

WW.^3.*XX.*ZZ+(-1).*WW.^4.*XX.*ZZ+WW.^4.*YY.*ZZ+(-1).*WW.^3.*XX.*YY.*ZZ) ...

.*(3.*WW.^2+(-4).*phi.*WW.^2+phi.^2.*WW.^2+3.*WW.^3+2.*phi.*WW.^3+(-2).* ...

phi.^2.*WW.^3+2.*phi.*WW.^4+phi.^2.*WW.^4+(-3).*WW.*XX+4.*phi.*WW.*XX+( ...

-1).*phi.^2.*WW.*XX+(-3).*WW.^2.*XX+(-2).*phi.*WW.^2.*XX+2.*phi.^2.* ...

WW.^2.*XX+(-2).*phi.*WW.^3.*XX+(-1).*phi.^2.*WW.^3.*XX+(-1).*phi.*WW.* ...

YY+phi.^2.*WW.*YY+WW.^2.*YY+2.*phi.*WW.^2.*YY+(-4).*phi.^2.*WW.^2.*YY+( ...

-5).*WW.^3.*YY+5.*phi.*WW.^3.*YY+5.*phi.^2.*WW.^3.*YY+(-2).*WW.^4.*YY+( ...

-6).*phi.*WW.^4.*YY+(-2).*phi.^2.*WW.^4.*YY+3.*WW.^2.*XX.*YY+(-5).*phi.* ...

WW.^2.*XX.*YY+phi.^2.*WW.^2.*XX.*YY+3.*WW.^3.*XX.*YY+4.*phi.*WW.^3.*XX.* ...

YY+(-2).*phi.^2.*WW.^3.*XX.*YY+phi.*WW.^4.*XX.*YY+phi.^2.*WW.^4.*XX.*YY+ ...

phi.*WW.*ZZ+(-1).*phi.^2.*WW.*ZZ+(-3).*WW.^2.*ZZ+4.*phi.*WW.^2.*ZZ+2.* ...

phi.^2.*WW.^2.*ZZ+(-3).*WW.^3.*ZZ+(-5).*phi.*WW.^3.*ZZ+(-1).*phi.^2.* ...

WW.^3.*ZZ+2.*WW.*XX.*ZZ+(-6).*phi.*WW.*XX.*ZZ+2.*phi.^2.*WW.*XX.*ZZ+5.* ...

WW.^2.*XX.*ZZ+5.*phi.*WW.^2.*XX.*ZZ+(-5).*phi.^2.*WW.^2.*XX.*ZZ+(-1).* ...

WW.^3.*XX.*ZZ+2.*phi.*WW.^3.*XX.*ZZ+4.*phi.^2.*WW.^3.*XX.*ZZ+(-1).*phi.* ...

WW.^4.*XX.*ZZ+(-1).*phi.^2.*WW.^4.*XX.*ZZ+(-2).*phi.*WW.^2.*YY.*ZZ+ ...

phi.^2.*WW.^2.*YY.*ZZ+3.*WW.^3.*YY.*ZZ+(-2).*phi.*WW.^3.*YY.*ZZ+(-2).* ...

phi.^2.*WW.^3.*YY.*ZZ+3.*WW.^4.*YY.*ZZ+4.*phi.*WW.^4.*YY.*ZZ+phi.^2.* ...

WW.^4.*YY.*ZZ+2.*phi.*WW.*XX.*YY.*ZZ+(-1).*phi.^2.*WW.*XX.*YY.*ZZ+(-3).* ...

WW.^2.*XX.*YY.*ZZ+2.*phi.*WW.^2.*XX.*YY.*ZZ+2.*phi.^2.*WW.^2.*XX.*YY.* ...

ZZ+(-3).*WW.^3.*XX.*YY.*ZZ+(-4).*phi.*WW.^3.*XX.*YY.*ZZ+(-1).*phi.^2.* ...

WW.^3.*XX.*YY.*ZZ)+2.*(3.*WW.^2+(-4).*phi.*WW.^2+phi.^2.*WW.^2+3.*WW.^3+ ...

2.*phi.*WW.^3+(-2).*phi.^2.*WW.^3+2.*phi.*WW.^4+phi.^2.*WW.^4+(-3).*WW.* ...

XX+4.*phi.*WW.*XX+(-1).*phi.^2.*WW.*XX+(-3).*WW.^2.*XX+(-2).*phi.* ...

WW.^2.*XX+2.*phi.^2.*WW.^2.*XX+(-2).*phi.*WW.^3.*XX+(-1).*phi.^2.* ...

WW.^3.*XX+(-1).*phi.*WW.*YY+phi.^2.*WW.*YY+WW.^2.*YY+2.*phi.*WW.^2.*YY+( ...

-4).*phi.^2.*WW.^2.*YY+(-5).*WW.^3.*YY+5.*phi.*WW.^3.*YY+5.*phi.^2.* ...

WW.^3.*YY+(-2).*WW.^4.*YY+(-6).*phi.*WW.^4.*YY+(-2).*phi.^2.*WW.^4.*YY+ ...

3.*WW.^2.*XX.*YY+(-5).*phi.*WW.^2.*XX.*YY+phi.^2.*WW.^2.*XX.*YY+3.* ...

WW.^3.*XX.*YY+4.*phi.*WW.^3.*XX.*YY+(-2).*phi.^2.*WW.^3.*XX.*YY+phi.* ...

WW.^4.*XX.*YY+phi.^2.*WW.^4.*XX.*YY+phi.*WW.*ZZ+(-1).*phi.^2.*WW.*ZZ+( ...

-3).*WW.^2.*ZZ+4.*phi.*WW.^2.*ZZ+2.*phi.^2.*WW.^2.*ZZ+(-3).*WW.^3.*ZZ+( ...

-5).*phi.*WW.^3.*ZZ+(-1).*phi.^2.*WW.^3.*ZZ+2.*WW.*XX.*ZZ+(-6).*phi.* ...

WW.*XX.*ZZ+2.*phi.^2.*WW.*XX.*ZZ+5.*WW.^2.*XX.*ZZ+5.*phi.*WW.^2.*XX.*ZZ+ ...

(-5).*phi.^2.*WW.^2.*XX.*ZZ+(-1).*WW.^3.*XX.*ZZ+2.*phi.*WW.^3.*XX.*ZZ+ ...

4.*phi.^2.*WW.^3.*XX.*ZZ+(-1).*phi.*WW.^4.*XX.*ZZ+(-1).*phi.^2.*WW.^4.* ...

XX.*ZZ+(-2).*phi.*WW.^2.*YY.*ZZ+phi.^2.*WW.^2.*YY.*ZZ+3.*WW.^3.*YY.*ZZ+( ...

-2).*phi.*WW.^3.*YY.*ZZ+(-2).*phi.^2.*WW.^3.*YY.*ZZ+3.*WW.^4.*YY.*ZZ+4.* ...

phi.*WW.^4.*YY.*ZZ+phi.^2.*WW.^4.*YY.*ZZ+2.*phi.*WW.*XX.*YY.*ZZ+(-1).* ...

phi.^2.*WW.*XX.*YY.*ZZ+(-3).*WW.^2.*XX.*YY.*ZZ+2.*phi.*WW.^2.*XX.*YY.* ...

ZZ+2.*phi.^2.*WW.^2.*XX.*YY.*ZZ+(-3).*WW.^3.*XX.*YY.*ZZ+(-4).*phi.* ...

WW.^3.*XX.*YY.*ZZ+(-1).*phi.^2.*WW.^3.*XX.*YY.*ZZ).^3+(-9).*((-3).* ...

WW.^3+2.*phi.*WW.^3+(-1).*WW.^4+(-2).*phi.*WW.^4+3.*WW.^2.*XX+(-2).* ...

phi.*WW.^2.*XX+WW.^3.*XX+2.*phi.*WW.^3.*XX+(-1).*WW.^2.*YY+2.*phi.* ...

WW.^2.*YY+WW.^3.*YY+(-6).*phi.*WW.^3.*YY+4.*WW.^4.*YY+4.*phi.*WW.^4.*YY+ ...

(-3).*WW.^3.*XX.*YY+2.*phi.*WW.^3.*XX.*YY+(-1).*WW.^4.*XX.*YY+(-2).* ...

phi.*WW.^4.*XX.*YY+WW.^2.*ZZ+(-2).*phi.*WW.^2.*ZZ+3.*WW.^3.*ZZ+2.*phi.* ...

WW.^3.*ZZ+(-4).*WW.^2.*XX.*ZZ+4.*phi.*WW.^2.*XX.*ZZ+(-1).*WW.^3.*XX.*ZZ+ ...

(-6).*phi.*WW.^3.*XX.*ZZ+WW.^4.*XX.*ZZ+2.*phi.*WW.^4.*XX.*ZZ+(-1).* ...

WW.^3.*YY.*ZZ+2.*phi.*WW.^3.*YY.*ZZ+(-3).*WW.^4.*YY.*ZZ+(-2).*phi.* ...

WW.^4.*YY.*ZZ+WW.^2.*XX.*YY.*ZZ+(-2).*phi.*WW.^2.*XX.*YY.*ZZ+3.*WW.^3.* ...

XX.*YY.*ZZ+2.*phi.*WW.^3.*XX.*YY.*ZZ).*(3.*WW.^2+(-4).*phi.*WW.^2+ ...

phi.^2.*WW.^2+3.*WW.^3+2.*phi.*WW.^3+(-2).*phi.^2.*WW.^3+2.*phi.*WW.^4+ ...

phi.^2.*WW.^4+(-3).*WW.*XX+4.*phi.*WW.*XX+(-1).*phi.^2.*WW.*XX+(-3).* ...

WW.^2.*XX+(-2).*phi.*WW.^2.*XX+2.*phi.^2.*WW.^2.*XX+(-2).*phi.*WW.^3.* ...

XX+(-1).*phi.^2.*WW.^3.*XX+(-1).*phi.*WW.*YY+phi.^2.*WW.*YY+WW.^2.*YY+ ...

2.*phi.*WW.^2.*YY+(-4).*phi.^2.*WW.^2.*YY+(-5).*WW.^3.*YY+5.*phi.* ...

WW.^3.*YY+5.*phi.^2.*WW.^3.*YY+(-2).*WW.^4.*YY+(-6).*phi.*WW.^4.*YY+(-2) ...

.*phi.^2.*WW.^4.*YY+3.*WW.^2.*XX.*YY+(-5).*phi.*WW.^2.*XX.*YY+phi.^2.* ...

WW.^2.*XX.*YY+3.*WW.^3.*XX.*YY+4.*phi.*WW.^3.*XX.*YY+(-2).*phi.^2.* ...

WW.^3.*XX.*YY+phi.*WW.^4.*XX.*YY+phi.^2.*WW.^4.*XX.*YY+phi.*WW.*ZZ+(-1) ...

.*phi.^2.*WW.*ZZ+(-3).*WW.^2.*ZZ+4.*phi.*WW.^2.*ZZ+2.*phi.^2.*WW.^2.*ZZ+ ...

(-3).*WW.^3.*ZZ+(-5).*phi.*WW.^3.*ZZ+(-1).*phi.^2.*WW.^3.*ZZ+2.*WW.*XX.* ...

ZZ+(-6).*phi.*WW.*XX.*ZZ+2.*phi.^2.*WW.*XX.*ZZ+5.*WW.^2.*XX.*ZZ+5.*phi.* ...

WW.^2.*XX.*ZZ+(-5).*phi.^2.*WW.^2.*XX.*ZZ+(-1).*WW.^3.*XX.*ZZ+2.*phi.* ...

WW.^3.*XX.*ZZ+4.*phi.^2.*WW.^3.*XX.*ZZ+(-1).*phi.*WW.^4.*XX.*ZZ+(-1).* ...

phi.^2.*WW.^4.*XX.*ZZ+(-2).*phi.*WW.^2.*YY.*ZZ+phi.^2.*WW.^2.*YY.*ZZ+3.* ...

WW.^3.*YY.*ZZ+(-2).*phi.*WW.^3.*YY.*ZZ+(-2).*phi.^2.*WW.^3.*YY.*ZZ+3.* ...

WW.^4.*YY.*ZZ+4.*phi.*WW.^4.*YY.*ZZ+phi.^2.*WW.^4.*YY.*ZZ+2.*phi.*WW.* ...

XX.*YY.*ZZ+(-1).*phi.^2.*WW.*XX.*YY.*ZZ+(-3).*WW.^2.*XX.*YY.*ZZ+2.*phi.* ...

WW.^2.*XX.*YY.*ZZ+2.*phi.^2.*WW.^2.*XX.*YY.*ZZ+(-3).*WW.^3.*XX.*YY.*ZZ+( ...

-4).*phi.*WW.^3.*XX.*YY.*ZZ+(-1).*phi.^2.*WW.^3.*XX.*YY.*ZZ).*((-1).*WW+ ...

2.*phi.*WW+(-1).*phi.^2.*WW+(-3).*WW.^2+2.*phi.*WW.^2+phi.^2.*WW.^2+(-4) ...

.*phi.*WW.^3+phi.^2.*WW.^3+(-1).*phi.^2.*WW.^4+XX+(-2).*phi.*XX+phi.^2.* ...

XX+3.*WW.*XX+(-2).*phi.*WW.*XX+(-1).*phi.^2.*WW.*XX+4.*phi.*WW.^2.*XX+( ...

-1).*phi.^2.*WW.^2.*XX+phi.^2.*WW.^3.*XX+WW.^2.*YY+(-4).*phi.*WW.^2.*YY+ ...

2.*phi.^2.*WW.^2.*YY+3.*WW.^3.*YY+2.*phi.*WW.^3.*YY+(-4).*phi.^2.* ...

WW.^3.*YY+2.*phi.*WW.^4.*YY+2.*phi.^2.*WW.^4.*YY+(-1).*WW.*XX.*YY+4.* ...

phi.*WW.*XX.*YY+(-2).*phi.^2.*WW.*XX.*YY+(-3).*WW.^2.*XX.*YY+(-2).*phi.* ...

WW.^2.*XX.*YY+4.*phi.^2.*WW.^2.*XX.*YY+(-2).*phi.*WW.^3.*XX.*YY+(-2).* ...

phi.^2.*WW.^3.*XX.*YY+(-2).*phi.*WW.*ZZ+2.*phi.^2.*WW.*ZZ+3.*WW.^2.*ZZ+( ...

-2).*phi.*WW.^2.*ZZ+(-4).*phi.^2.*WW.^2.*ZZ+WW.^3.*ZZ+4.*phi.*WW.^3.*ZZ+ ...

2.*phi.^2.*WW.^3.*ZZ+2.*phi.*XX.*ZZ+(-2).*phi.^2.*XX.*ZZ+(-3).*WW.*XX.* ...

ZZ+2.*phi.*WW.*XX.*ZZ+4.*phi.^2.*WW.*XX.*ZZ+(-1).*WW.^2.*XX.*ZZ+(-4).* ...

phi.*WW.^2.*XX.*ZZ+(-2).*phi.^2.*WW.^2.*XX.*ZZ+(-1).*phi.^2.*WW.*YY.*ZZ+ ...

4.*phi.*WW.^2.*YY.*ZZ+phi.^2.*WW.^2.*YY.*ZZ+(-3).*WW.^3.*YY.*ZZ+(-2).* ...

phi.*WW.^3.*YY.*ZZ+phi.^2.*WW.^3.*YY.*ZZ+(-1).*WW.^4.*YY.*ZZ+(-2).*phi.* ...

WW.^4.*YY.*ZZ+(-1).*phi.^2.*WW.^4.*YY.*ZZ+phi.^2.*XX.*YY.*ZZ+(-4).*phi.* ...

WW.*XX.*YY.*ZZ+(-1).*phi.^2.*WW.*XX.*YY.*ZZ+3.*WW.^2.*XX.*YY.*ZZ+2.* ...

phi.*WW.^2.*XX.*YY.*ZZ+(-1).*phi.^2.*WW.^2.*XX.*YY.*ZZ+WW.^3.*XX.*YY.* ...

ZZ+2.*phi.*WW.^3.*XX.*YY.*ZZ+phi.^2.*WW.^3.*XX.*YY.*ZZ)+27.*(WW.^4+(-1) ...

.*WW.^3.*XX+WW.^3.*YY+(-2).*WW.^4.*YY+WW.^4.*XX.*YY+(-1).*WW.^3.*ZZ+2.* ...

WW.^3.*XX.*ZZ+(-1).*WW.^4.*XX.*ZZ+WW.^4.*YY.*ZZ+(-1).*WW.^3.*XX.*YY.*ZZ) ...

.*((-1).*WW+2.*phi.*WW+(-1).*phi.^2.*WW+(-3).*WW.^2+2.*phi.*WW.^2+ ...

phi.^2.*WW.^2+(-4).*phi.*WW.^3+phi.^2.*WW.^3+(-1).*phi.^2.*WW.^4+XX+(-2) ...

.*phi.*XX+phi.^2.*XX+3.*WW.*XX+(-2).*phi.*WW.*XX+(-1).*phi.^2.*WW.*XX+ ...

4.*phi.*WW.^2.*XX+(-1).*phi.^2.*WW.^2.*XX+phi.^2.*WW.^3.*XX+WW.^2.*YY+( ...

-4).*phi.*WW.^2.*YY+2.*phi.^2.*WW.^2.*YY+3.*WW.^3.*YY+2.*phi.*WW.^3.*YY+ ...

(-4).*phi.^2.*WW.^3.*YY+2.*phi.*WW.^4.*YY+2.*phi.^2.*WW.^4.*YY+(-1).* ...

WW.*XX.*YY+4.*phi.*WW.*XX.*YY+(-2).*phi.^2.*WW.*XX.*YY+(-3).*WW.^2.*XX.* ...

YY+(-2).*phi.*WW.^2.*XX.*YY+4.*phi.^2.*WW.^2.*XX.*YY+(-2).*phi.*WW.^3.* ...

XX.*YY+(-2).*phi.^2.*WW.^3.*XX.*YY+(-2).*phi.*WW.*ZZ+2.*phi.^2.*WW.*ZZ+ ...

3.*WW.^2.*ZZ+(-2).*phi.*WW.^2.*ZZ+(-4).*phi.^2.*WW.^2.*ZZ+WW.^3.*ZZ+4.* ...

phi.*WW.^3.*ZZ+2.*phi.^2.*WW.^3.*ZZ+2.*phi.*XX.*ZZ+(-2).*phi.^2.*XX.*ZZ+ ...

(-3).*WW.*XX.*ZZ+2.*phi.*WW.*XX.*ZZ+4.*phi.^2.*WW.*XX.*ZZ+(-1).*WW.^2.* ...

XX.*ZZ+(-4).*phi.*WW.^2.*XX.*ZZ+(-2).*phi.^2.*WW.^2.*XX.*ZZ+(-1).* ...

phi.^2.*WW.*YY.*ZZ+4.*phi.*WW.^2.*YY.*ZZ+phi.^2.*WW.^2.*YY.*ZZ+(-3).* ...

WW.^3.*YY.*ZZ+(-2).*phi.*WW.^3.*YY.*ZZ+phi.^2.*WW.^3.*YY.*ZZ+(-1).* ...

WW.^4.*YY.*ZZ+(-2).*phi.*WW.^4.*YY.*ZZ+(-1).*phi.^2.*WW.^4.*YY.*ZZ+ ...

phi.^2.*XX.*YY.*ZZ+(-4).*phi.*WW.*XX.*YY.*ZZ+(-1).*phi.^2.*WW.*XX.*YY.* ...

ZZ+3.*WW.^2.*XX.*YY.*ZZ+2.*phi.*WW.^2.*XX.*YY.*ZZ+(-1).*phi.^2.*WW.^2.* ...

XX.*YY.*ZZ+WW.^3.*XX.*YY.*ZZ+2.*phi.*WW.^3.*XX.*YY.*ZZ+phi.^2.*WW.^3.* ...

XX.*YY.*ZZ).^2+((-4).*(12.*(WW+(-2).*phi.*WW+phi.^2.*WW+2.*phi.*WW.^2+( ...

-2).*phi.^2.*WW.^2+phi.^2.*WW.^3+(-1).*XX+2.*phi.*XX+(-1).*phi.^2.*XX+( ...

-2).*phi.*WW.*XX+2.*phi.^2.*WW.*XX+(-1).*phi.^2.*WW.^2.*XX+phi.*WW.*YY+( ...

-1).*phi.^2.*WW.*YY+(-1).*WW.^2.*YY+2.*phi.^2.*WW.^2.*YY+(-1).*phi.* ...

WW.^3.*YY+(-1).*phi.^2.*WW.^3.*YY+(-1).*phi.*XX.*YY+phi.^2.*XX.*YY+WW.* ...

XX.*YY+(-2).*phi.^2.*WW.*XX.*YY+phi.*WW.^2.*XX.*YY+phi.^2.*WW.^2.*XX.* ...

YY+phi.*WW.*ZZ+(-1).*phi.^2.*WW.*ZZ+(-1).*WW.^2.*ZZ+2.*phi.^2.*WW.^2.* ...

ZZ+(-1).*phi.*WW.^3.*ZZ+(-1).*phi.^2.*WW.^3.*ZZ+(-1).*phi.*XX.*ZZ+ ...

phi.^2.*XX.*ZZ+WW.*XX.*ZZ+(-2).*phi.^2.*WW.*XX.*ZZ+phi.*WW.^2.*XX.*ZZ+ ...

phi.^2.*WW.^2.*XX.*ZZ+phi.^2.*WW.*YY.*ZZ+(-2).*phi.*WW.^2.*YY.*ZZ+(-2).* ...

phi.^2.*WW.^2.*YY.*ZZ+WW.^3.*YY.*ZZ+2.*phi.*WW.^3.*YY.*ZZ+phi.^2.* ...

WW.^3.*YY.*ZZ+(-1).*phi.^2.*XX.*YY.*ZZ+2.*phi.*WW.*XX.*YY.*ZZ+2.* ...

phi.^2.*WW.*XX.*YY.*ZZ+(-1).*WW.^2.*XX.*YY.*ZZ+(-2).*phi.*WW.^2.*XX.* ...

YY.*ZZ+(-1).*phi.^2.*WW.^2.*XX.*YY.*ZZ).*(WW.^4+(-1).*WW.^3.*XX+WW.^3.* ...

YY+(-2).*WW.^4.*YY+WW.^4.*XX.*YY+(-1).*WW.^3.*ZZ+2.*WW.^3.*XX.*ZZ+(-1).* ...

WW.^4.*XX.*ZZ+WW.^4.*YY.*ZZ+(-1).*WW.^3.*XX.*YY.*ZZ)+(3.*WW.^2+(-4).* ...

phi.*WW.^2+phi.^2.*WW.^2+3.*WW.^3+2.*phi.*WW.^3+(-2).*phi.^2.*WW.^3+2.* ...

phi.*WW.^4+phi.^2.*WW.^4+(-3).*WW.*XX+4.*phi.*WW.*XX+(-1).*phi.^2.*WW.* ...

XX+(-3).*WW.^2.*XX+(-2).*phi.*WW.^2.*XX+2.*phi.^2.*WW.^2.*XX+(-2).*phi.* ...

WW.^3.*XX+(-1).*phi.^2.*WW.^3.*XX+(-1).*phi.*WW.*YY+phi.^2.*WW.*YY+ ...

WW.^2.*YY+2.*phi.*WW.^2.*YY+(-4).*phi.^2.*WW.^2.*YY+(-5).*WW.^3.*YY+5.* ...

phi.*WW.^3.*YY+5.*phi.^2.*WW.^3.*YY+(-2).*WW.^4.*YY+(-6).*phi.*WW.^4.* ...

YY+(-2).*phi.^2.*WW.^4.*YY+3.*WW.^2.*XX.*YY+(-5).*phi.*WW.^2.*XX.*YY+ ...

phi.^2.*WW.^2.*XX.*YY+3.*WW.^3.*XX.*YY+4.*phi.*WW.^3.*XX.*YY+(-2).* ...

phi.^2.*WW.^3.*XX.*YY+phi.*WW.^4.*XX.*YY+phi.^2.*WW.^4.*XX.*YY+phi.*WW.* ...

ZZ+(-1).*phi.^2.*WW.*ZZ+(-3).*WW.^2.*ZZ+4.*phi.*WW.^2.*ZZ+2.*phi.^2.* ...

WW.^2.*ZZ+(-3).*WW.^3.*ZZ+(-5).*phi.*WW.^3.*ZZ+(-1).*phi.^2.*WW.^3.*ZZ+ ...

2.*WW.*XX.*ZZ+(-6).*phi.*WW.*XX.*ZZ+2.*phi.^2.*WW.*XX.*ZZ+5.*WW.^2.*XX.* ...

ZZ+5.*phi.*WW.^2.*XX.*ZZ+(-5).*phi.^2.*WW.^2.*XX.*ZZ+(-1).*WW.^3.*XX.* ...

ZZ+2.*phi.*WW.^3.*XX.*ZZ+4.*phi.^2.*WW.^3.*XX.*ZZ+(-1).*phi.*WW.^4.*XX.* ...

ZZ+(-1).*phi.^2.*WW.^4.*XX.*ZZ+(-2).*phi.*WW.^2.*YY.*ZZ+phi.^2.*WW.^2.* ...

YY.*ZZ+3.*WW.^3.*YY.*ZZ+(-2).*phi.*WW.^3.*YY.*ZZ+(-2).*phi.^2.*WW.^3.* ...

YY.*ZZ+3.*WW.^4.*YY.*ZZ+4.*phi.*WW.^4.*YY.*ZZ+phi.^2.*WW.^4.*YY.*ZZ+2.* ...

phi.*WW.*XX.*YY.*ZZ+(-1).*phi.^2.*WW.*XX.*YY.*ZZ+(-3).*WW.^2.*XX.*YY.* ...

ZZ+2.*phi.*WW.^2.*XX.*YY.*ZZ+2.*phi.^2.*WW.^2.*XX.*YY.*ZZ+(-3).*WW.^3.* ...

XX.*YY.*ZZ+(-4).*phi.*WW.^3.*XX.*YY.*ZZ+(-1).*phi.^2.*WW.^3.*XX.*YY.*ZZ) ...

.^2+(-3).*((-3).*WW.^3+2.*phi.*WW.^3+(-1).*WW.^4+(-2).*phi.*WW.^4+3.* ...

WW.^2.*XX+(-2).*phi.*WW.^2.*XX+WW.^3.*XX+2.*phi.*WW.^3.*XX+(-1).*WW.^2.* ...

YY+2.*phi.*WW.^2.*YY+WW.^3.*YY+(-6).*phi.*WW.^3.*YY+4.*WW.^4.*YY+4.* ...

phi.*WW.^4.*YY+(-3).*WW.^3.*XX.*YY+2.*phi.*WW.^3.*XX.*YY+(-1).*WW.^4.* ...

XX.*YY+(-2).*phi.*WW.^4.*XX.*YY+WW.^2.*ZZ+(-2).*phi.*WW.^2.*ZZ+3.* ...

WW.^3.*ZZ+2.*phi.*WW.^3.*ZZ+(-4).*WW.^2.*XX.*ZZ+4.*phi.*WW.^2.*XX.*ZZ+( ...

-1).*WW.^3.*XX.*ZZ+(-6).*phi.*WW.^3.*XX.*ZZ+WW.^4.*XX.*ZZ+2.*phi.* ...

WW.^4.*XX.*ZZ+(-1).*WW.^3.*YY.*ZZ+2.*phi.*WW.^3.*YY.*ZZ+(-3).*WW.^4.* ...

YY.*ZZ+(-2).*phi.*WW.^4.*YY.*ZZ+WW.^2.*XX.*YY.*ZZ+(-2).*phi.*WW.^2.*XX.* ...

YY.*ZZ+3.*WW.^3.*XX.*YY.*ZZ+2.*phi.*WW.^3.*XX.*YY.*ZZ).*((-1).*WW+2.* ...

phi.*WW+(-1).*phi.^2.*WW+(-3).*WW.^2+2.*phi.*WW.^2+phi.^2.*WW.^2+(-4).* ...

phi.*WW.^3+phi.^2.*WW.^3+(-1).*phi.^2.*WW.^4+XX+(-2).*phi.*XX+phi.^2.* ...

XX+3.*WW.*XX+(-2).*phi.*WW.*XX+(-1).*phi.^2.*WW.*XX+4.*phi.*WW.^2.*XX+( ...

-1).*phi.^2.*WW.^2.*XX+phi.^2.*WW.^3.*XX+WW.^2.*YY+(-4).*phi.*WW.^2.*YY+ ...

2.*phi.^2.*WW.^2.*YY+3.*WW.^3.*YY+2.*phi.*WW.^3.*YY+(-4).*phi.^2.* ...

WW.^3.*YY+2.*phi.*WW.^4.*YY+2.*phi.^2.*WW.^4.*YY+(-1).*WW.*XX.*YY+4.* ...

phi.*WW.*XX.*YY+(-2).*phi.^2.*WW.*XX.*YY+(-3).*WW.^2.*XX.*YY+(-2).*phi.* ...

WW.^2.*XX.*YY+4.*phi.^2.*WW.^2.*XX.*YY+(-2).*phi.*WW.^3.*XX.*YY+(-2).* ...

phi.^2.*WW.^3.*XX.*YY+(-2).*phi.*WW.*ZZ+2.*phi.^2.*WW.*ZZ+3.*WW.^2.*ZZ+( ...

-2).*phi.*WW.^2.*ZZ+(-4).*phi.^2.*WW.^2.*ZZ+WW.^3.*ZZ+4.*phi.*WW.^3.*ZZ+ ...

2.*phi.^2.*WW.^3.*ZZ+2.*phi.*XX.*ZZ+(-2).*phi.^2.*XX.*ZZ+(-3).*WW.*XX.* ...

ZZ+2.*phi.*WW.*XX.*ZZ+4.*phi.^2.*WW.*XX.*ZZ+(-1).*WW.^2.*XX.*ZZ+(-4).* ...

phi.*WW.^2.*XX.*ZZ+(-2).*phi.^2.*WW.^2.*XX.*ZZ+(-1).*phi.^2.*WW.*YY.*ZZ+ ...

4.*phi.*WW.^2.*YY.*ZZ+phi.^2.*WW.^2.*YY.*ZZ+(-3).*WW.^3.*YY.*ZZ+(-2).* ...

phi.*WW.^3.*YY.*ZZ+phi.^2.*WW.^3.*YY.*ZZ+(-1).*WW.^4.*YY.*ZZ+(-2).*phi.* ...

WW.^4.*YY.*ZZ+(-1).*phi.^2.*WW.^4.*YY.*ZZ+phi.^2.*XX.*YY.*ZZ+(-4).*phi.* ...

WW.*XX.*YY.*ZZ+(-1).*phi.^2.*WW.*XX.*YY.*ZZ+3.*WW.^2.*XX.*YY.*ZZ+2.* ...

phi.*WW.^2.*XX.*YY.*ZZ+(-1).*phi.^2.*WW.^2.*XX.*YY.*ZZ+WW.^3.*XX.*YY.* ...

ZZ+2.*phi.*WW.^3.*XX.*YY.*ZZ+phi.^2.*WW.^3.*XX.*YY.*ZZ)).^3+(27.*(WW+( ...

-2).*phi.*WW+phi.^2.*WW+2.*phi.*WW.^2+(-2).*phi.^2.*WW.^2+phi.^2.*WW.^3+ ...

(-1).*XX+2.*phi.*XX+(-1).*phi.^2.*XX+(-2).*phi.*WW.*XX+2.*phi.^2.*WW.* ...

XX+(-1).*phi.^2.*WW.^2.*XX+phi.*WW.*YY+(-1).*phi.^2.*WW.*YY+(-1).* ...

WW.^2.*YY+2.*phi.^2.*WW.^2.*YY+(-1).*phi.*WW.^3.*YY+(-1).*phi.^2.* ...

WW.^3.*YY+(-1).*phi.*XX.*YY+phi.^2.*XX.*YY+WW.*XX.*YY+(-2).*phi.^2.*WW.* ...

XX.*YY+phi.*WW.^2.*XX.*YY+phi.^2.*WW.^2.*XX.*YY+phi.*WW.*ZZ+(-1).* ...

phi.^2.*WW.*ZZ+(-1).*WW.^2.*ZZ+2.*phi.^2.*WW.^2.*ZZ+(-1).*phi.*WW.^3.* ...

ZZ+(-1).*phi.^2.*WW.^3.*ZZ+(-1).*phi.*XX.*ZZ+phi.^2.*XX.*ZZ+WW.*XX.*ZZ+( ...

-2).*phi.^2.*WW.*XX.*ZZ+phi.*WW.^2.*XX.*ZZ+phi.^2.*WW.^2.*XX.*ZZ+ ...

phi.^2.*WW.*YY.*ZZ+(-2).*phi.*WW.^2.*YY.*ZZ+(-2).*phi.^2.*WW.^2.*YY.*ZZ+ ...

WW.^3.*YY.*ZZ+2.*phi.*WW.^3.*YY.*ZZ+phi.^2.*WW.^3.*YY.*ZZ+(-1).*phi.^2.* ...

XX.*YY.*ZZ+2.*phi.*WW.*XX.*YY.*ZZ+2.*phi.^2.*WW.*XX.*YY.*ZZ+(-1).* ...

WW.^2.*XX.*YY.*ZZ+(-2).*phi.*WW.^2.*XX.*YY.*ZZ+(-1).*phi.^2.*WW.^2.*XX.* ...

YY.*ZZ).*((-3).*WW.^3+2.*phi.*WW.^3+(-1).*WW.^4+(-2).*phi.*WW.^4+3.* ...

WW.^2.*XX+(-2).*phi.*WW.^2.*XX+WW.^3.*XX+2.*phi.*WW.^3.*XX+(-1).*WW.^2.* ...

YY+2.*phi.*WW.^2.*YY+WW.^3.*YY+(-6).*phi.*WW.^3.*YY+4.*WW.^4.*YY+4.* ...

phi.*WW.^4.*YY+(-3).*WW.^3.*XX.*YY+2.*phi.*WW.^3.*XX.*YY+(-1).*WW.^4.* ...

XX.*YY+(-2).*phi.*WW.^4.*XX.*YY+WW.^2.*ZZ+(-2).*phi.*WW.^2.*ZZ+3.* ...

WW.^3.*ZZ+2.*phi.*WW.^3.*ZZ+(-4).*WW.^2.*XX.*ZZ+4.*phi.*WW.^2.*XX.*ZZ+( ...

-1).*WW.^3.*XX.*ZZ+(-6).*phi.*WW.^3.*XX.*ZZ+WW.^4.*XX.*ZZ+2.*phi.* ...

WW.^4.*XX.*ZZ+(-1).*WW.^3.*YY.*ZZ+2.*phi.*WW.^3.*YY.*ZZ+(-3).*WW.^4.* ...

YY.*ZZ+(-2).*phi.*WW.^4.*YY.*ZZ+WW.^2.*XX.*YY.*ZZ+(-2).*phi.*WW.^2.*XX.* ...

YY.*ZZ+3.*WW.^3.*XX.*YY.*ZZ+2.*phi.*WW.^3.*XX.*YY.*ZZ).^2+(-72).*(WW+( ...

-2).*phi.*WW+phi.^2.*WW+2.*phi.*WW.^2+(-2).*phi.^2.*WW.^2+phi.^2.*WW.^3+ ...

(-1).*XX+2.*phi.*XX+(-1).*phi.^2.*XX+(-2).*phi.*WW.*XX+2.*phi.^2.*WW.* ...

XX+(-1).*phi.^2.*WW.^2.*XX+phi.*WW.*YY+(-1).*phi.^2.*WW.*YY+(-1).* ...

WW.^2.*YY+2.*phi.^2.*WW.^2.*YY+(-1).*phi.*WW.^3.*YY+(-1).*phi.^2.* ...

WW.^3.*YY+(-1).*phi.*XX.*YY+phi.^2.*XX.*YY+WW.*XX.*YY+(-2).*phi.^2.*WW.* ...

XX.*YY+phi.*WW.^2.*XX.*YY+phi.^2.*WW.^2.*XX.*YY+phi.*WW.*ZZ+(-1).* ...

phi.^2.*WW.*ZZ+(-1).*WW.^2.*ZZ+2.*phi.^2.*WW.^2.*ZZ+(-1).*phi.*WW.^3.* ...

ZZ+(-1).*phi.^2.*WW.^3.*ZZ+(-1).*phi.*XX.*ZZ+phi.^2.*XX.*ZZ+WW.*XX.*ZZ+( ...

-2).*phi.^2.*WW.*XX.*ZZ+phi.*WW.^2.*XX.*ZZ+phi.^2.*WW.^2.*XX.*ZZ+ ...

phi.^2.*WW.*YY.*ZZ+(-2).*phi.*WW.^2.*YY.*ZZ+(-2).*phi.^2.*WW.^2.*YY.*ZZ+ ...

WW.^3.*YY.*ZZ+2.*phi.*WW.^3.*YY.*ZZ+phi.^2.*WW.^3.*YY.*ZZ+(-1).*phi.^2.* ...

XX.*YY.*ZZ+2.*phi.*WW.*XX.*YY.*ZZ+2.*phi.^2.*WW.*XX.*YY.*ZZ+(-1).* ...

WW.^2.*XX.*YY.*ZZ+(-2).*phi.*WW.^2.*XX.*YY.*ZZ+(-1).*phi.^2.*WW.^2.*XX.* ...

YY.*ZZ).*(WW.^4+(-1).*WW.^3.*XX+WW.^3.*YY+(-2).*WW.^4.*YY+WW.^4.*XX.*YY+ ...

(-1).*WW.^3.*ZZ+2.*WW.^3.*XX.*ZZ+(-1).*WW.^4.*XX.*ZZ+WW.^4.*YY.*ZZ+(-1) ...

.*WW.^3.*XX.*YY.*ZZ).*(3.*WW.^2+(-4).*phi.*WW.^2+phi.^2.*WW.^2+3.*WW.^3+ ...

2.*phi.*WW.^3+(-2).*phi.^2.*WW.^3+2.*phi.*WW.^4+phi.^2.*WW.^4+(-3).*WW.* ...

XX+4.*phi.*WW.*XX+(-1).*phi.^2.*WW.*XX+(-3).*WW.^2.*XX+(-2).*phi.* ...

WW.^2.*XX+2.*phi.^2.*WW.^2.*XX+(-2).*phi.*WW.^3.*XX+(-1).*phi.^2.* ...

WW.^3.*XX+(-1).*phi.*WW.*YY+phi.^2.*WW.*YY+WW.^2.*YY+2.*phi.*WW.^2.*YY+( ...

-4).*phi.^2.*WW.^2.*YY+(-5).*WW.^3.*YY+5.*phi.*WW.^3.*YY+5.*phi.^2.* ...

WW.^3.*YY+(-2).*WW.^4.*YY+(-6).*phi.*WW.^4.*YY+(-2).*phi.^2.*WW.^4.*YY+ ...

3.*WW.^2.*XX.*YY+(-5).*phi.*WW.^2.*XX.*YY+phi.^2.*WW.^2.*XX.*YY+3.* ...

WW.^3.*XX.*YY+4.*phi.*WW.^3.*XX.*YY+(-2).*phi.^2.*WW.^3.*XX.*YY+phi.* ...

WW.^4.*XX.*YY+phi.^2.*WW.^4.*XX.*YY+phi.*WW.*ZZ+(-1).*phi.^2.*WW.*ZZ+( ...

-3).*WW.^2.*ZZ+4.*phi.*WW.^2.*ZZ+2.*phi.^2.*WW.^2.*ZZ+(-3).*WW.^3.*ZZ+( ...

-5).*phi.*WW.^3.*ZZ+(-1).*phi.^2.*WW.^3.*ZZ+2.*WW.*XX.*ZZ+(-6).*phi.* ...

WW.*XX.*ZZ+2.*phi.^2.*WW.*XX.*ZZ+5.*WW.^2.*XX.*ZZ+5.*phi.*WW.^2.*XX.*ZZ+ ...

(-5).*phi.^2.*WW.^2.*XX.*ZZ+(-1).*WW.^3.*XX.*ZZ+2.*phi.*WW.^3.*XX.*ZZ+ ...

4.*phi.^2.*WW.^3.*XX.*ZZ+(-1).*phi.*WW.^4.*XX.*ZZ+(-1).*phi.^2.*WW.^4.* ...

XX.*ZZ+(-2).*phi.*WW.^2.*YY.*ZZ+phi.^2.*WW.^2.*YY.*ZZ+3.*WW.^3.*YY.*ZZ+( ...

-2).*phi.*WW.^3.*YY.*ZZ+(-2).*phi.^2.*WW.^3.*YY.*ZZ+3.*WW.^4.*YY.*ZZ+4.* ...

phi.*WW.^4.*YY.*ZZ+phi.^2.*WW.^4.*YY.*ZZ+2.*phi.*WW.*XX.*YY.*ZZ+(-1).* ...

phi.^2.*WW.*XX.*YY.*ZZ+(-3).*WW.^2.*XX.*YY.*ZZ+2.*phi.*WW.^2.*XX.*YY.* ...

ZZ+2.*phi.^2.*WW.^2.*XX.*YY.*ZZ+(-3).*WW.^3.*XX.*YY.*ZZ+(-4).*phi.* ...

WW.^3.*XX.*YY.*ZZ+(-1).*phi.^2.*WW.^3.*XX.*YY.*ZZ)+2.*(3.*WW.^2+(-4).* ...

phi.*WW.^2+phi.^2.*WW.^2+3.*WW.^3+2.*phi.*WW.^3+(-2).*phi.^2.*WW.^3+2.* ...

phi.*WW.^4+phi.^2.*WW.^4+(-3).*WW.*XX+4.*phi.*WW.*XX+(-1).*phi.^2.*WW.* ...

XX+(-3).*WW.^2.*XX+(-2).*phi.*WW.^2.*XX+2.*phi.^2.*WW.^2.*XX+(-2).*phi.* ...

WW.^3.*XX+(-1).*phi.^2.*WW.^3.*XX+(-1).*phi.*WW.*YY+phi.^2.*WW.*YY+ ...

WW.^2.*YY+2.*phi.*WW.^2.*YY+(-4).*phi.^2.*WW.^2.*YY+(-5).*WW.^3.*YY+5.* ...

phi.*WW.^3.*YY+5.*phi.^2.*WW.^3.*YY+(-2).*WW.^4.*YY+(-6).*phi.*WW.^4.* ...

YY+(-2).*phi.^2.*WW.^4.*YY+3.*WW.^2.*XX.*YY+(-5).*phi.*WW.^2.*XX.*YY+ ...

phi.^2.*WW.^2.*XX.*YY+3.*WW.^3.*XX.*YY+4.*phi.*WW.^3.*XX.*YY+(-2).* ...

phi.^2.*WW.^3.*XX.*YY+phi.*WW.^4.*XX.*YY+phi.^2.*WW.^4.*XX.*YY+phi.*WW.* ...

ZZ+(-1).*phi.^2.*WW.*ZZ+(-3).*WW.^2.*ZZ+4.*phi.*WW.^2.*ZZ+2.*phi.^2.* ...

WW.^2.*ZZ+(-3).*WW.^3.*ZZ+(-5).*phi.*WW.^3.*ZZ+(-1).*phi.^2.*WW.^3.*ZZ+ ...

2.*WW.*XX.*ZZ+(-6).*phi.*WW.*XX.*ZZ+2.*phi.^2.*WW.*XX.*ZZ+5.*WW.^2.*XX.* ...

ZZ+5.*phi.*WW.^2.*XX.*ZZ+(-5).*phi.^2.*WW.^2.*XX.*ZZ+(-1).*WW.^3.*XX.* ...

ZZ+2.*phi.*WW.^3.*XX.*ZZ+4.*phi.^2.*WW.^3.*XX.*ZZ+(-1).*phi.*WW.^4.*XX.* ...

ZZ+(-1).*phi.^2.*WW.^4.*XX.*ZZ+(-2).*phi.*WW.^2.*YY.*ZZ+phi.^2.*WW.^2.* ...

YY.*ZZ+3.*WW.^3.*YY.*ZZ+(-2).*phi.*WW.^3.*YY.*ZZ+(-2).*phi.^2.*WW.^3.* ...

YY.*ZZ+3.*WW.^4.*YY.*ZZ+4.*phi.*WW.^4.*YY.*ZZ+phi.^2.*WW.^4.*YY.*ZZ+2.* ...

phi.*WW.*XX.*YY.*ZZ+(-1).*phi.^2.*WW.*XX.*YY.*ZZ+(-3).*WW.^2.*XX.*YY.* ...

ZZ+2.*phi.*WW.^2.*XX.*YY.*ZZ+2.*phi.^2.*WW.^2.*XX.*YY.*ZZ+(-3).*WW.^3.* ...

XX.*YY.*ZZ+(-4).*phi.*WW.^3.*XX.*YY.*ZZ+(-1).*phi.^2.*WW.^3.*XX.*YY.*ZZ) ...

.^3+(-9).*((-3).*WW.^3+2.*phi.*WW.^3+(-1).*WW.^4+(-2).*phi.*WW.^4+3.* ...

WW.^2.*XX+(-2).*phi.*WW.^2.*XX+WW.^3.*XX+2.*phi.*WW.^3.*XX+(-1).*WW.^2.* ...

YY+2.*phi.*WW.^2.*YY+WW.^3.*YY+(-6).*phi.*WW.^3.*YY+4.*WW.^4.*YY+4.* ...

phi.*WW.^4.*YY+(-3).*WW.^3.*XX.*YY+2.*phi.*WW.^3.*XX.*YY+(-1).*WW.^4.* ...

XX.*YY+(-2).*phi.*WW.^4.*XX.*YY+WW.^2.*ZZ+(-2).*phi.*WW.^2.*ZZ+3.* ...

WW.^3.*ZZ+2.*phi.*WW.^3.*ZZ+(-4).*WW.^2.*XX.*ZZ+4.*phi.*WW.^2.*XX.*ZZ+( ...

-1).*WW.^3.*XX.*ZZ+(-6).*phi.*WW.^3.*XX.*ZZ+WW.^4.*XX.*ZZ+2.*phi.* ...

WW.^4.*XX.*ZZ+(-1).*WW.^3.*YY.*ZZ+2.*phi.*WW.^3.*YY.*ZZ+(-3).*WW.^4.* ...

YY.*ZZ+(-2).*phi.*WW.^4.*YY.*ZZ+WW.^2.*XX.*YY.*ZZ+(-2).*phi.*WW.^2.*XX.* ...

YY.*ZZ+3.*WW.^3.*XX.*YY.*ZZ+2.*phi.*WW.^3.*XX.*YY.*ZZ).*(3.*WW.^2+(-4).* ...

phi.*WW.^2+phi.^2.*WW.^2+3.*WW.^3+2.*phi.*WW.^3+(-2).*phi.^2.*WW.^3+2.* ...

phi.*WW.^4+phi.^2.*WW.^4+(-3).*WW.*XX+4.*phi.*WW.*XX+(-1).*phi.^2.*WW.* ...

XX+(-3).*WW.^2.*XX+(-2).*phi.*WW.^2.*XX+2.*phi.^2.*WW.^2.*XX+(-2).*phi.* ...

WW.^3.*XX+(-1).*phi.^2.*WW.^3.*XX+(-1).*phi.*WW.*YY+phi.^2.*WW.*YY+ ...

WW.^2.*YY+2.*phi.*WW.^2.*YY+(-4).*phi.^2.*WW.^2.*YY+(-5).*WW.^3.*YY+5.* ...

phi.*WW.^3.*YY+5.*phi.^2.*WW.^3.*YY+(-2).*WW.^4.*YY+(-6).*phi.*WW.^4.* ...

YY+(-2).*phi.^2.*WW.^4.*YY+3.*WW.^2.*XX.*YY+(-5).*phi.*WW.^2.*XX.*YY+ ...

phi.^2.*WW.^2.*XX.*YY+3.*WW.^3.*XX.*YY+4.*phi.*WW.^3.*XX.*YY+(-2).* ...

phi.^2.*WW.^3.*XX.*YY+phi.*WW.^4.*XX.*YY+phi.^2.*WW.^4.*XX.*YY+phi.*WW.* ...

ZZ+(-1).*phi.^2.*WW.*ZZ+(-3).*WW.^2.*ZZ+4.*phi.*WW.^2.*ZZ+2.*phi.^2.* ...

WW.^2.*ZZ+(-3).*WW.^3.*ZZ+(-5).*phi.*WW.^3.*ZZ+(-1).*phi.^2.*WW.^3.*ZZ+ ...

2.*WW.*XX.*ZZ+(-6).*phi.*WW.*XX.*ZZ+2.*phi.^2.*WW.*XX.*ZZ+5.*WW.^2.*XX.* ...

ZZ+5.*phi.*WW.^2.*XX.*ZZ+(-5).*phi.^2.*WW.^2.*XX.*ZZ+(-1).*WW.^3.*XX.* ...

ZZ+2.*phi.*WW.^3.*XX.*ZZ+4.*phi.^2.*WW.^3.*XX.*ZZ+(-1).*phi.*WW.^4.*XX.* ...

ZZ+(-1).*phi.^2.*WW.^4.*XX.*ZZ+(-2).*phi.*WW.^2.*YY.*ZZ+phi.^2.*WW.^2.* ...

YY.*ZZ+3.*WW.^3.*YY.*ZZ+(-2).*phi.*WW.^3.*YY.*ZZ+(-2).*phi.^2.*WW.^3.* ...

YY.*ZZ+3.*WW.^4.*YY.*ZZ+4.*phi.*WW.^4.*YY.*ZZ+phi.^2.*WW.^4.*YY.*ZZ+2.* ...

phi.*WW.*XX.*YY.*ZZ+(-1).*phi.^2.*WW.*XX.*YY.*ZZ+(-3).*WW.^2.*XX.*YY.* ...

ZZ+2.*phi.*WW.^2.*XX.*YY.*ZZ+2.*phi.^2.*WW.^2.*XX.*YY.*ZZ+(-3).*WW.^3.* ...

XX.*YY.*ZZ+(-4).*phi.*WW.^3.*XX.*YY.*ZZ+(-1).*phi.^2.*WW.^3.*XX.*YY.*ZZ) ...

.*((-1).*WW+2.*phi.*WW+(-1).*phi.^2.*WW+(-3).*WW.^2+2.*phi.*WW.^2+ ...

phi.^2.*WW.^2+(-4).*phi.*WW.^3+phi.^2.*WW.^3+(-1).*phi.^2.*WW.^4+XX+(-2) ...

.*phi.*XX+phi.^2.*XX+3.*WW.*XX+(-2).*phi.*WW.*XX+(-1).*phi.^2.*WW.*XX+ ...

4.*phi.*WW.^2.*XX+(-1).*phi.^2.*WW.^2.*XX+phi.^2.*WW.^3.*XX+WW.^2.*YY+( ...

-4).*phi.*WW.^2.*YY+2.*phi.^2.*WW.^2.*YY+3.*WW.^3.*YY+2.*phi.*WW.^3.*YY+ ...

(-4).*phi.^2.*WW.^3.*YY+2.*phi.*WW.^4.*YY+2.*phi.^2.*WW.^4.*YY+(-1).* ...

WW.*XX.*YY+4.*phi.*WW.*XX.*YY+(-2).*phi.^2.*WW.*XX.*YY+(-3).*WW.^2.*XX.* ...

YY+(-2).*phi.*WW.^2.*XX.*YY+4.*phi.^2.*WW.^2.*XX.*YY+(-2).*phi.*WW.^3.* ...

XX.*YY+(-2).*phi.^2.*WW.^3.*XX.*YY+(-2).*phi.*WW.*ZZ+2.*phi.^2.*WW.*ZZ+ ...

3.*WW.^2.*ZZ+(-2).*phi.*WW.^2.*ZZ+(-4).*phi.^2.*WW.^2.*ZZ+WW.^3.*ZZ+4.* ...

phi.*WW.^3.*ZZ+2.*phi.^2.*WW.^3.*ZZ+2.*phi.*XX.*ZZ+(-2).*phi.^2.*XX.*ZZ+ ...

(-3).*WW.*XX.*ZZ+2.*phi.*WW.*XX.*ZZ+4.*phi.^2.*WW.*XX.*ZZ+(-1).*WW.^2.* ...

XX.*ZZ+(-4).*phi.*WW.^2.*XX.*ZZ+(-2).*phi.^2.*WW.^2.*XX.*ZZ+(-1).* ...

phi.^2.*WW.*YY.*ZZ+4.*phi.*WW.^2.*YY.*ZZ+phi.^2.*WW.^2.*YY.*ZZ+(-3).* ...

WW.^3.*YY.*ZZ+(-2).*phi.*WW.^3.*YY.*ZZ+phi.^2.*WW.^3.*YY.*ZZ+(-1).* ...

WW.^4.*YY.*ZZ+(-2).*phi.*WW.^4.*YY.*ZZ+(-1).*phi.^2.*WW.^4.*YY.*ZZ+ ...

phi.^2.*XX.*YY.*ZZ+(-4).*phi.*WW.*XX.*YY.*ZZ+(-1).*phi.^2.*WW.*XX.*YY.* ...

ZZ+3.*WW.^2.*XX.*YY.*ZZ+2.*phi.*WW.^2.*XX.*YY.*ZZ+(-1).*phi.^2.*WW.^2.* ...

XX.*YY.*ZZ+WW.^3.*XX.*YY.*ZZ+2.*phi.*WW.^3.*XX.*YY.*ZZ+phi.^2.*WW.^3.* ...

XX.*YY.*ZZ)+27.*(WW.^4+(-1).*WW.^3.*XX+WW.^3.*YY+(-2).*WW.^4.*YY+WW.^4.* ...

XX.*YY+(-1).*WW.^3.*ZZ+2.*WW.^3.*XX.*ZZ+(-1).*WW.^4.*XX.*ZZ+WW.^4.*YY.* ...

ZZ+(-1).*WW.^3.*XX.*YY.*ZZ).*((-1).*WW+2.*phi.*WW+(-1).*phi.^2.*WW+(-3) ...

.*WW.^2+2.*phi.*WW.^2+phi.^2.*WW.^2+(-4).*phi.*WW.^3+phi.^2.*WW.^3+(-1) ...

.*phi.^2.*WW.^4+XX+(-2).*phi.*XX+phi.^2.*XX+3.*WW.*XX+(-2).*phi.*WW.*XX+ ...

(-1).*phi.^2.*WW.*XX+4.*phi.*WW.^2.*XX+(-1).*phi.^2.*WW.^2.*XX+phi.^2.* ...

WW.^3.*XX+WW.^2.*YY+(-4).*phi.*WW.^2.*YY+2.*phi.^2.*WW.^2.*YY+3.*WW.^3.* ...

YY+2.*phi.*WW.^3.*YY+(-4).*phi.^2.*WW.^3.*YY+2.*phi.*WW.^4.*YY+2.* ...

phi.^2.*WW.^4.*YY+(-1).*WW.*XX.*YY+4.*phi.*WW.*XX.*YY+(-2).*phi.^2.*WW.* ...

XX.*YY+(-3).*WW.^2.*XX.*YY+(-2).*phi.*WW.^2.*XX.*YY+4.*phi.^2.*WW.^2.* ...

XX.*YY+(-2).*phi.*WW.^3.*XX.*YY+(-2).*phi.^2.*WW.^3.*XX.*YY+(-2).*phi.* ...

WW.*ZZ+2.*phi.^2.*WW.*ZZ+3.*WW.^2.*ZZ+(-2).*phi.*WW.^2.*ZZ+(-4).* ...

phi.^2.*WW.^2.*ZZ+WW.^3.*ZZ+4.*phi.*WW.^3.*ZZ+2.*phi.^2.*WW.^3.*ZZ+2.* ...

phi.*XX.*ZZ+(-2).*phi.^2.*XX.*ZZ+(-3).*WW.*XX.*ZZ+2.*phi.*WW.*XX.*ZZ+4.* ...

phi.^2.*WW.*XX.*ZZ+(-1).*WW.^2.*XX.*ZZ+(-4).*phi.*WW.^2.*XX.*ZZ+(-2).* ...

phi.^2.*WW.^2.*XX.*ZZ+(-1).*phi.^2.*WW.*YY.*ZZ+4.*phi.*WW.^2.*YY.*ZZ+ ...

phi.^2.*WW.^2.*YY.*ZZ+(-3).*WW.^3.*YY.*ZZ+(-2).*phi.*WW.^3.*YY.*ZZ+ ...

phi.^2.*WW.^3.*YY.*ZZ+(-1).*WW.^4.*YY.*ZZ+(-2).*phi.*WW.^4.*YY.*ZZ+(-1) ...

.*phi.^2.*WW.^4.*YY.*ZZ+phi.^2.*XX.*YY.*ZZ+(-4).*phi.*WW.*XX.*YY.*ZZ+( ...

-1).*phi.^2.*WW.*XX.*YY.*ZZ+3.*WW.^2.*XX.*YY.*ZZ+2.*phi.*WW.^2.*XX.*YY.* ...

ZZ+(-1).*phi.^2.*WW.^2.*XX.*YY.*ZZ+WW.^3.*XX.*YY.*ZZ+2.*phi.*WW.^3.*XX.* ...

YY.*ZZ+phi.^2.*WW.^3.*XX.*YY.*ZZ).^2).^2).^(1/2)).^(-1/3)+(1/3).*2.^( ...

-1/3).*(WW+(-2).*phi.*WW+phi.^2.*WW+2.*phi.*WW.^2+(-2).*phi.^2.*WW.^2+ ...

phi.^2.*WW.^3+(-1).*XX+2.*phi.*XX+(-1).*phi.^2.*XX+(-2).*phi.*WW.*XX+2.* ...

phi.^2.*WW.*XX+(-1).*phi.^2.*WW.^2.*XX+phi.*WW.*YY+(-1).*phi.^2.*WW.*YY+ ...

(-1).*WW.^2.*YY+2.*phi.^2.*WW.^2.*YY+(-1).*phi.*WW.^3.*YY+(-1).*phi.^2.* ...

WW.^3.*YY+(-1).*phi.*XX.*YY+phi.^2.*XX.*YY+WW.*XX.*YY+(-2).*phi.^2.*WW.* ...

XX.*YY+phi.*WW.^2.*XX.*YY+phi.^2.*WW.^2.*XX.*YY+phi.*WW.*ZZ+(-1).* ...

phi.^2.*WW.*ZZ+(-1).*WW.^2.*ZZ+2.*phi.^2.*WW.^2.*ZZ+(-1).*phi.*WW.^3.* ...

ZZ+(-1).*phi.^2.*WW.^3.*ZZ+(-1).*phi.*XX.*ZZ+phi.^2.*XX.*ZZ+WW.*XX.*ZZ+( ...

-2).*phi.^2.*WW.*XX.*ZZ+phi.*WW.^2.*XX.*ZZ+phi.^2.*WW.^2.*XX.*ZZ+ ...

phi.^2.*WW.*YY.*ZZ+(-2).*phi.*WW.^2.*YY.*ZZ+(-2).*phi.^2.*WW.^2.*YY.*ZZ+ ...

WW.^3.*YY.*ZZ+2.*phi.*WW.^3.*YY.*ZZ+phi.^2.*WW.^3.*YY.*ZZ+(-1).*phi.^2.* ...

XX.*YY.*ZZ+2.*phi.*WW.*XX.*YY.*ZZ+2.*phi.^2.*WW.*XX.*YY.*ZZ+(-1).* ...

WW.^2.*XX.*YY.*ZZ+(-2).*phi.*WW.^2.*XX.*YY.*ZZ+(-1).*phi.^2.*WW.^2.*XX.* ...

YY.*ZZ).^(-1).*(27.*(WW+(-2).*phi.*WW+phi.^2.*WW+2.*phi.*WW.^2+(-2).* ...

phi.^2.*WW.^2+phi.^2.*WW.^3+(-1).*XX+2.*phi.*XX+(-1).*phi.^2.*XX+(-2).* ...

phi.*WW.*XX+2.*phi.^2.*WW.*XX+(-1).*phi.^2.*WW.^2.*XX+phi.*WW.*YY+(-1).* ...

phi.^2.*WW.*YY+(-1).*WW.^2.*YY+2.*phi.^2.*WW.^2.*YY+(-1).*phi.*WW.^3.* ...

YY+(-1).*phi.^2.*WW.^3.*YY+(-1).*phi.*XX.*YY+phi.^2.*XX.*YY+WW.*XX.*YY+( ...

-2).*phi.^2.*WW.*XX.*YY+phi.*WW.^2.*XX.*YY+phi.^2.*WW.^2.*XX.*YY+phi.* ...

WW.*ZZ+(-1).*phi.^2.*WW.*ZZ+(-1).*WW.^2.*ZZ+2.*phi.^2.*WW.^2.*ZZ+(-1).* ...

phi.*WW.^3.*ZZ+(-1).*phi.^2.*WW.^3.*ZZ+(-1).*phi.*XX.*ZZ+phi.^2.*XX.*ZZ+ ...

WW.*XX.*ZZ+(-2).*phi.^2.*WW.*XX.*ZZ+phi.*WW.^2.*XX.*ZZ+phi.^2.*WW.^2.* ...

XX.*ZZ+phi.^2.*WW.*YY.*ZZ+(-2).*phi.*WW.^2.*YY.*ZZ+(-2).*phi.^2.*WW.^2.* ...

YY.*ZZ+WW.^3.*YY.*ZZ+2.*phi.*WW.^3.*YY.*ZZ+phi.^2.*WW.^3.*YY.*ZZ+(-1).* ...

phi.^2.*XX.*YY.*ZZ+2.*phi.*WW.*XX.*YY.*ZZ+2.*phi.^2.*WW.*XX.*YY.*ZZ+(-1) ...

.*WW.^2.*XX.*YY.*ZZ+(-2).*phi.*WW.^2.*XX.*YY.*ZZ+(-1).*phi.^2.*WW.^2.* ...

XX.*YY.*ZZ).*((-3).*WW.^3+2.*phi.*WW.^3+(-1).*WW.^4+(-2).*phi.*WW.^4+3.* ...

WW.^2.*XX+(-2).*phi.*WW.^2.*XX+WW.^3.*XX+2.*phi.*WW.^3.*XX+(-1).*WW.^2.* ...

YY+2.*phi.*WW.^2.*YY+WW.^3.*YY+(-6).*phi.*WW.^3.*YY+4.*WW.^4.*YY+4.* ...

phi.*WW.^4.*YY+(-3).*WW.^3.*XX.*YY+2.*phi.*WW.^3.*XX.*YY+(-1).*WW.^4.* ...

XX.*YY+(-2).*phi.*WW.^4.*XX.*YY+WW.^2.*ZZ+(-2).*phi.*WW.^2.*ZZ+3.* ...

WW.^3.*ZZ+2.*phi.*WW.^3.*ZZ+(-4).*WW.^2.*XX.*ZZ+4.*phi.*WW.^2.*XX.*ZZ+( ...

-1).*WW.^3.*XX.*ZZ+(-6).*phi.*WW.^3.*XX.*ZZ+WW.^4.*XX.*ZZ+2.*phi.* ...

WW.^4.*XX.*ZZ+(-1).*WW.^3.*YY.*ZZ+2.*phi.*WW.^3.*YY.*ZZ+(-3).*WW.^4.* ...

YY.*ZZ+(-2).*phi.*WW.^4.*YY.*ZZ+WW.^2.*XX.*YY.*ZZ+(-2).*phi.*WW.^2.*XX.* ...

YY.*ZZ+3.*WW.^3.*XX.*YY.*ZZ+2.*phi.*WW.^3.*XX.*YY.*ZZ).^2+(-72).*(WW+( ...

-2).*phi.*WW+phi.^2.*WW+2.*phi.*WW.^2+(-2).*phi.^2.*WW.^2+phi.^2.*WW.^3+ ...

(-1).*XX+2.*phi.*XX+(-1).*phi.^2.*XX+(-2).*phi.*WW.*XX+2.*phi.^2.*WW.* ...

XX+(-1).*phi.^2.*WW.^2.*XX+phi.*WW.*YY+(-1).*phi.^2.*WW.*YY+(-1).* ...

WW.^2.*YY+2.*phi.^2.*WW.^2.*YY+(-1).*phi.*WW.^3.*YY+(-1).*phi.^2.* ...

WW.^3.*YY+(-1).*phi.*XX.*YY+phi.^2.*XX.*YY+WW.*XX.*YY+(-2).*phi.^2.*WW.* ...

XX.*YY+phi.*WW.^2.*XX.*YY+phi.^2.*WW.^2.*XX.*YY+phi.*WW.*ZZ+(-1).* ...

phi.^2.*WW.*ZZ+(-1).*WW.^2.*ZZ+2.*phi.^2.*WW.^2.*ZZ+(-1).*phi.*WW.^3.* ...

ZZ+(-1).*phi.^2.*WW.^3.*ZZ+(-1).*phi.*XX.*ZZ+phi.^2.*XX.*ZZ+WW.*XX.*ZZ+( ...

-2).*phi.^2.*WW.*XX.*ZZ+phi.*WW.^2.*XX.*ZZ+phi.^2.*WW.^2.*XX.*ZZ+ ...

phi.^2.*WW.*YY.*ZZ+(-2).*phi.*WW.^2.*YY.*ZZ+(-2).*phi.^2.*WW.^2.*YY.*ZZ+ ...

WW.^3.*YY.*ZZ+2.*phi.*WW.^3.*YY.*ZZ+phi.^2.*WW.^3.*YY.*ZZ+(-1).*phi.^2.* ...

XX.*YY.*ZZ+2.*phi.*WW.*XX.*YY.*ZZ+2.*phi.^2.*WW.*XX.*YY.*ZZ+(-1).* ...

WW.^2.*XX.*YY.*ZZ+(-2).*phi.*WW.^2.*XX.*YY.*ZZ+(-1).*phi.^2.*WW.^2.*XX.* ...

YY.*ZZ).*(WW.^4+(-1).*WW.^3.*XX+WW.^3.*YY+(-2).*WW.^4.*YY+WW.^4.*XX.*YY+ ...

(-1).*WW.^3.*ZZ+2.*WW.^3.*XX.*ZZ+(-1).*WW.^4.*XX.*ZZ+WW.^4.*YY.*ZZ+(-1) ...

.*WW.^3.*XX.*YY.*ZZ).*(3.*WW.^2+(-4).*phi.*WW.^2+phi.^2.*WW.^2+3.*WW.^3+ ...

2.*phi.*WW.^3+(-2).*phi.^2.*WW.^3+2.*phi.*WW.^4+phi.^2.*WW.^4+(-3).*WW.* ...

XX+4.*phi.*WW.*XX+(-1).*phi.^2.*WW.*XX+(-3).*WW.^2.*XX+(-2).*phi.* ...

WW.^2.*XX+2.*phi.^2.*WW.^2.*XX+(-2).*phi.*WW.^3.*XX+(-1).*phi.^2.* ...

WW.^3.*XX+(-1).*phi.*WW.*YY+phi.^2.*WW.*YY+WW.^2.*YY+2.*phi.*WW.^2.*YY+( ...

-4).*phi.^2.*WW.^2.*YY+(-5).*WW.^3.*YY+5.*phi.*WW.^3.*YY+5.*phi.^2.* ...

WW.^3.*YY+(-2).*WW.^4.*YY+(-6).*phi.*WW.^4.*YY+(-2).*phi.^2.*WW.^4.*YY+ ...

3.*WW.^2.*XX.*YY+(-5).*phi.*WW.^2.*XX.*YY+phi.^2.*WW.^2.*XX.*YY+3.* ...

WW.^3.*XX.*YY+4.*phi.*WW.^3.*XX.*YY+(-2).*phi.^2.*WW.^3.*XX.*YY+phi.* ...

WW.^4.*XX.*YY+phi.^2.*WW.^4.*XX.*YY+phi.*WW.*ZZ+(-1).*phi.^2.*WW.*ZZ+( ...

-3).*WW.^2.*ZZ+4.*phi.*WW.^2.*ZZ+2.*phi.^2.*WW.^2.*ZZ+(-3).*WW.^3.*ZZ+( ...

-5).*phi.*WW.^3.*ZZ+(-1).*phi.^2.*WW.^3.*ZZ+2.*WW.*XX.*ZZ+(-6).*phi.* ...

WW.*XX.*ZZ+2.*phi.^2.*WW.*XX.*ZZ+5.*WW.^2.*XX.*ZZ+5.*phi.*WW.^2.*XX.*ZZ+ ...

(-5).*phi.^2.*WW.^2.*XX.*ZZ+(-1).*WW.^3.*XX.*ZZ+2.*phi.*WW.^3.*XX.*ZZ+ ...

4.*phi.^2.*WW.^3.*XX.*ZZ+(-1).*phi.*WW.^4.*XX.*ZZ+(-1).*phi.^2.*WW.^4.* ...

XX.*ZZ+(-2).*phi.*WW.^2.*YY.*ZZ+phi.^2.*WW.^2.*YY.*ZZ+3.*WW.^3.*YY.*ZZ+( ...

-2).*phi.*WW.^3.*YY.*ZZ+(-2).*phi.^2.*WW.^3.*YY.*ZZ+3.*WW.^4.*YY.*ZZ+4.* ...

phi.*WW.^4.*YY.*ZZ+phi.^2.*WW.^4.*YY.*ZZ+2.*phi.*WW.*XX.*YY.*ZZ+(-1).* ...

phi.^2.*WW.*XX.*YY.*ZZ+(-3).*WW.^2.*XX.*YY.*ZZ+2.*phi.*WW.^2.*XX.*YY.* ...

ZZ+2.*phi.^2.*WW.^2.*XX.*YY.*ZZ+(-3).*WW.^3.*XX.*YY.*ZZ+(-4).*phi.* ...

WW.^3.*XX.*YY.*ZZ+(-1).*phi.^2.*WW.^3.*XX.*YY.*ZZ)+2.*(3.*WW.^2+(-4).* ...

phi.*WW.^2+phi.^2.*WW.^2+3.*WW.^3+2.*phi.*WW.^3+(-2).*phi.^2.*WW.^3+2.* ...

phi.*WW.^4+phi.^2.*WW.^4+(-3).*WW.*XX+4.*phi.*WW.*XX+(-1).*phi.^2.*WW.* ...

XX+(-3).*WW.^2.*XX+(-2).*phi.*WW.^2.*XX+2.*phi.^2.*WW.^2.*XX+(-2).*phi.* ...

WW.^3.*XX+(-1).*phi.^2.*WW.^3.*XX+(-1).*phi.*WW.*YY+phi.^2.*WW.*YY+ ...

WW.^2.*YY+2.*phi.*WW.^2.*YY+(-4).*phi.^2.*WW.^2.*YY+(-5).*WW.^3.*YY+5.* ...

phi.*WW.^3.*YY+5.*phi.^2.*WW.^3.*YY+(-2).*WW.^4.*YY+(-6).*phi.*WW.^4.* ...

YY+(-2).*phi.^2.*WW.^4.*YY+3.*WW.^2.*XX.*YY+(-5).*phi.*WW.^2.*XX.*YY+ ...

phi.^2.*WW.^2.*XX.*YY+3.*WW.^3.*XX.*YY+4.*phi.*WW.^3.*XX.*YY+(-2).* ...

phi.^2.*WW.^3.*XX.*YY+phi.*WW.^4.*XX.*YY+phi.^2.*WW.^4.*XX.*YY+phi.*WW.* ...

ZZ+(-1).*phi.^2.*WW.*ZZ+(-3).*WW.^2.*ZZ+4.*phi.*WW.^2.*ZZ+2.*phi.^2.* ...

WW.^2.*ZZ+(-3).*WW.^3.*ZZ+(-5).*phi.*WW.^3.*ZZ+(-1).*phi.^2.*WW.^3.*ZZ+ ...

2.*WW.*XX.*ZZ+(-6).*phi.*WW.*XX.*ZZ+2.*phi.^2.*WW.*XX.*ZZ+5.*WW.^2.*XX.* ...

ZZ+5.*phi.*WW.^2.*XX.*ZZ+(-5).*phi.^2.*WW.^2.*XX.*ZZ+(-1).*WW.^3.*XX.* ...

ZZ+2.*phi.*WW.^3.*XX.*ZZ+4.*phi.^2.*WW.^3.*XX.*ZZ+(-1).*phi.*WW.^4.*XX.* ...

ZZ+(-1).*phi.^2.*WW.^4.*XX.*ZZ+(-2).*phi.*WW.^2.*YY.*ZZ+phi.^2.*WW.^2.* ...

YY.*ZZ+3.*WW.^3.*YY.*ZZ+(-2).*phi.*WW.^3.*YY.*ZZ+(-2).*phi.^2.*WW.^3.* ...

YY.*ZZ+3.*WW.^4.*YY.*ZZ+4.*phi.*WW.^4.*YY.*ZZ+phi.^2.*WW.^4.*YY.*ZZ+2.* ...

phi.*WW.*XX.*YY.*ZZ+(-1).*phi.^2.*WW.*XX.*YY.*ZZ+(-3).*WW.^2.*XX.*YY.* ...

ZZ+2.*phi.*WW.^2.*XX.*YY.*ZZ+2.*phi.^2.*WW.^2.*XX.*YY.*ZZ+(-3).*WW.^3.* ...

XX.*YY.*ZZ+(-4).*phi.*WW.^3.*XX.*YY.*ZZ+(-1).*phi.^2.*WW.^3.*XX.*YY.*ZZ) ...

.^3+(-9).*((-3).*WW.^3+2.*phi.*WW.^3+(-1).*WW.^4+(-2).*phi.*WW.^4+3.* ...

WW.^2.*XX+(-2).*phi.*WW.^2.*XX+WW.^3.*XX+2.*phi.*WW.^3.*XX+(-1).*WW.^2.* ...

YY+2.*phi.*WW.^2.*YY+WW.^3.*YY+(-6).*phi.*WW.^3.*YY+4.*WW.^4.*YY+4.* ...

phi.*WW.^4.*YY+(-3).*WW.^3.*XX.*YY+2.*phi.*WW.^3.*XX.*YY+(-1).*WW.^4.* ...

XX.*YY+(-2).*phi.*WW.^4.*XX.*YY+WW.^2.*ZZ+(-2).*phi.*WW.^2.*ZZ+3.* ...

WW.^3.*ZZ+2.*phi.*WW.^3.*ZZ+(-4).*WW.^2.*XX.*ZZ+4.*phi.*WW.^2.*XX.*ZZ+( ...

-1).*WW.^3.*XX.*ZZ+(-6).*phi.*WW.^3.*XX.*ZZ+WW.^4.*XX.*ZZ+2.*phi.* ...

WW.^4.*XX.*ZZ+(-1).*WW.^3.*YY.*ZZ+2.*phi.*WW.^3.*YY.*ZZ+(-3).*WW.^4.* ...

YY.*ZZ+(-2).*phi.*WW.^4.*YY.*ZZ+WW.^2.*XX.*YY.*ZZ+(-2).*phi.*WW.^2.*XX.* ...

YY.*ZZ+3.*WW.^3.*XX.*YY.*ZZ+2.*phi.*WW.^3.*XX.*YY.*ZZ).*(3.*WW.^2+(-4).* ...

phi.*WW.^2+phi.^2.*WW.^2+3.*WW.^3+2.*phi.*WW.^3+(-2).*phi.^2.*WW.^3+2.* ...

phi.*WW.^4+phi.^2.*WW.^4+(-3).*WW.*XX+4.*phi.*WW.*XX+(-1).*phi.^2.*WW.* ...

XX+(-3).*WW.^2.*XX+(-2).*phi.*WW.^2.*XX+2.*phi.^2.*WW.^2.*XX+(-2).*phi.* ...

WW.^3.*XX+(-1).*phi.^2.*WW.^3.*XX+(-1).*phi.*WW.*YY+phi.^2.*WW.*YY+ ...

WW.^2.*YY+2.*phi.*WW.^2.*YY+(-4).*phi.^2.*WW.^2.*YY+(-5).*WW.^3.*YY+5.* ...

phi.*WW.^3.*YY+5.*phi.^2.*WW.^3.*YY+(-2).*WW.^4.*YY+(-6).*phi.*WW.^4.* ...

YY+(-2).*phi.^2.*WW.^4.*YY+3.*WW.^2.*XX.*YY+(-5).*phi.*WW.^2.*XX.*YY+ ...

phi.^2.*WW.^2.*XX.*YY+3.*WW.^3.*XX.*YY+4.*phi.*WW.^3.*XX.*YY+(-2).* ...

phi.^2.*WW.^3.*XX.*YY+phi.*WW.^4.*XX.*YY+phi.^2.*WW.^4.*XX.*YY+phi.*WW.* ...

ZZ+(-1).*phi.^2.*WW.*ZZ+(-3).*WW.^2.*ZZ+4.*phi.*WW.^2.*ZZ+2.*phi.^2.* ...

WW.^2.*ZZ+(-3).*WW.^3.*ZZ+(-5).*phi.*WW.^3.*ZZ+(-1).*phi.^2.*WW.^3.*ZZ+ ...

2.*WW.*XX.*ZZ+(-6).*phi.*WW.*XX.*ZZ+2.*phi.^2.*WW.*XX.*ZZ+5.*WW.^2.*XX.* ...
[truncated: 431,031 more chars]
